# Unraveling the effect of topological structures of B/N‐doped conjugated macrocycles on spectral properties: A computational study

**DOI:** 10.1002/smo2.70066

**Published:** 2026-06-10

**Authors:** Wen‐Yu Guo, Yi Zeng, Yu‐Meng Xu, Pang‐Kuan Chen, Xiao‐Yan Zheng

**Affiliations:** ^1^ Key Laboratory of Cluster Science of Ministry of Education, State Key Laboratory of Environment Characteristics and Effects for Near‐space, Beijing Key Laboratory of Intelligent Molecular Materials and High‐throughput Manufacturing, School of Chemistry and Chemical Engineering Beijing Institute of Technology Beijing China

**Keywords:** donor‐acceptor effect, emission spectra, organic conjugated macrocycle, smart luminescent materials, topological structure

## Abstract

Organic conjugated macrocycles have attracted significant attention for their applications in smart luminescent materials, yet precise modulation of their spectral properties remains challenging. In this work, a series of fully B‐doped, fully N‐doped and hybrid B/N‐doped conjugated macrocycles were designed to systematically investigate the relationship between their topological structures and spectral properties. Three topological factors of heteroatom doped macrocycles were incorporated into the molecular design: ring size, number of triarylborane (Ar_3_B) and triarylamine (Ar_3_N) segments, and π‐linker between segments. For macrocycles with phenyl as the π‐linker, at a fixed ring size, both absorption (*λ*_abs_) and emission (*λ*_emi_) wavelength exhibit a first increase followed by a decrease as the number of Ar_3_N segments increases accompanied by the decrease of Ar_3_B segments. For each size of macrocycles, the reddest *λ*_emi_ usually contains a balanced ratio of Ar_3_B and Ar_3_N segments, with at least two Ar_3_B and Ar_3_N segments. Regardless of the ring size of macrocycles, the ratio of Ar_3_B to Ar_3_N segments plays a crucial role in tuning the spectral properties. A more balanced ratio enhances both electron‐donating and electron‐accepting abilities, thereby strengthening the intramolecular charge transfer effect and leading to a more redshifted emission spectra. A similar trend of *λ*_emi_ that first red‐shift then blue‐shift was also observed for macrocycles with fluorene or biphenyl as π‐linkers. Notably, at a given ring size and relative number of Ar_3_N and Ar_3_B, phenyl‐linked macrocycles exhibit a more pronounced red‐shift than those with fluorene or biphenyl linkers, and fluorene‐linked systems show slightly red‐shifted spectra relative to biphenyl‐linked analogs. These spectral trends are well explained by the electronic properties of the fundamental building blocks. This work provides important theoretical guidance for the development of smart luminescent materials based on conjugated macrocycles.

## Graphical abstract

Herein, we constructed a series of fully B‐doped, fully N‐doped and hybrid B/N‐doped conjugated macrocycles considering three key topological factors. At a fixed ring size, the spectra initially redshift then blueshift with varying B/N ratio, while ring size exhibits negligible impact on spectra. Notably, phenyl‐linked B/N‐doped macrocycles exhibit a more pronounced red‐shift emission than those with fluorene or biphenyl π‐linkers.

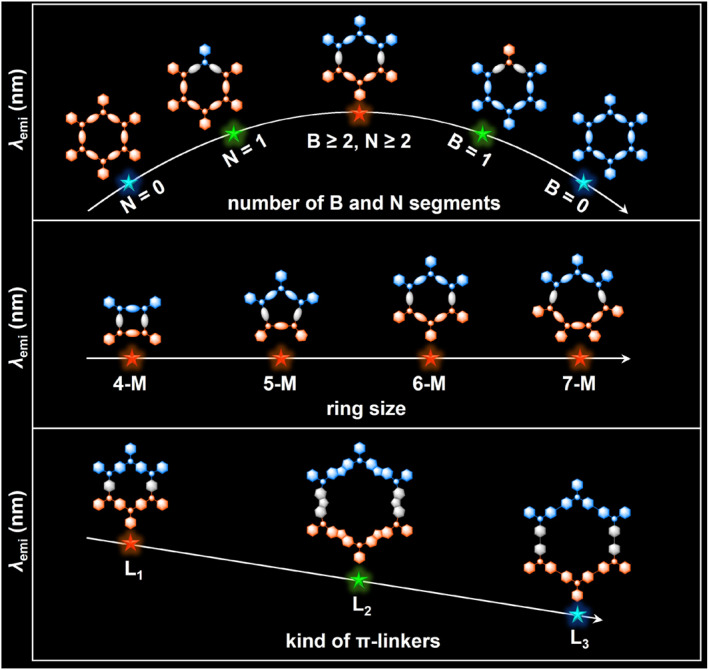

## 1 INTRODUCTION

Smart organic materials are pivotal to the advancement of modern technologies,[^1^, ^2^, ^3^, ^4^, ^5^] which have been widely used in nanomedicine and healthcare,^[6]^ media displays,^[7]^ environmental remediation,^[8]^ and so on. Macrocyclic architectures, characterized by inherent cyclic skeletons, intrinsic cavities, and specific host‐guest binding abilities[^9^, ^10^, ^11^, ^12^, ^13^] exhibit pronounced potential for the rational design and precise fabrication of diverse smart functional organic materials.[^14^, ^15^, ^16^, ^17^, ^18^] Organic conjugated macrocycles can be regarded as terminal‐less counterparts of linear π‐conjugated oligomers.[^19^, ^20^] According to their composition, the reported conjugated macrocycles can be broadly categorized into two types: the first type consists of macrocycles formed by multiple π‐repeating segments, while the second type comprises macrocycles constructed from electron‐donating (D) and electron‐accepting (A) segments.^[21]^ Compared to traditional macrocyclic structures, the fully conjugated framework endows their superior luminescent and charge transport properties. Most importantly, the rigid backbone of conjugated macrocycles can significantly restrict the conformational relaxation, thereby suppressing the nonradiative decay channels of the excited state, resulting in the enhanced emission.[^22^, ^23^, ^24^, ^25^] For example, a conjugated macrocycle constructed by carbazolyl‐*m*‐phenylene as D segments and phenyltriazine as A segments has achieved substantially higher fluorescence quantum efficiency (FQE) than its linear analog (77% and 49%, respectively) in toluene solvent.^[24]^ A similar trend is also observed in system composed of *N*,*N*′‐diphenyl‐*p*‐phenylenediamine and dibenzo[*a*,*j*]phenazine in toluene solution, where the cyclized structure demonstrates a higher FQE (28%) compared to its linear analog (20%).^[23]^ Therefore, conjugated macrocycles, which possess superior luminescent properties and inherent dynamic nature of host‐guest interactions, stand out as an ideal platform for the development of smart luminescent materials.[^25^, ^26^, ^27^, ^28^, ^29^, ^30^, ^31^, ^32^, ^33^]

The topological factors of conjugated macrocycles, including ring size, number of D and A segments, and π‐linkers between neighboring D or A segments, interplay together and have great impact on their luminescent properties. For instance, as ring size of cycloparaphenylene (CPP) increased by varying the number of phenyl units from 8 to 16, their absorption wavelength (*λ*_abs_) remains constant (around 340 nm), whereas the emission wavelength (*λ*_emi_) gradually blue‐shifts from 533 to 438 nm.[^34^, ^35^] The blue‐shifted emission of macrocycles with the increase of ring size also could extend to other series of CPP derivatives.[^36^, ^37^, ^38^, ^39^, ^40^] In contrast, for D–A typed macrocycle containing aniline as the D segment, and triazine as the A segment, the insertion of a phenyl spacer between D and A segments not only enlarges ring size, but also enhances π‐conjugation of macrocyclic backbone, thereby leading to red‐shifted emission from 450 to 530 nm.^[31]^ The composition of π‐linkers between D or A segments also influences the spectral properties. For macrocycles composed of six electron‐accepting triarylborane (Ar_3_B) segments, changing π‐linkers from phenyl group^[41]^ to fluorene group^[42]^ in THF solution results in a distinct emission redshift from 382 to 425 nm. Similarly, the macrocycle contains six electron‐donating triarylamine (Ar_3_N) segments with a phenyl group as π‐linker displays the *λ*_emi_ of 423 nm; it redshifted obviously to 467 and 433 nm, respectively, after changing π‐linkers into naphthalene and fluorene groups.^[43]^ Notably, even for both ring size and π‐linker constant, the change of relative number of D and A units in conjugated macrocycles lead to significant influence on their spectral properties. This is clearly demonstrated by six‐membered macrocycles containing Ar_3_B and Ar_3_N segments with phenyl as π‐linker, where the emission spectra exhibit gradual redshift in toluene from MC‐BN5 with one Ar_3_B and five Ar_3_N (*λ*_emi_ = 520 nm)^[44]^ to MC‐b‐B3N3 with three Ar_3_B and three Ar_3_N segments (*λ*_emi_ = 612 nm).^[45]^ Despite these advances, the relationship between topological structures and spectral properties of organic conjugated macrocycles remains elusive. In this contribution, we aim at filling this gap and systematically explore the influence of different topological factors of organic conjugated macrocycles on their spectral properties.

**FIGURE 1:**
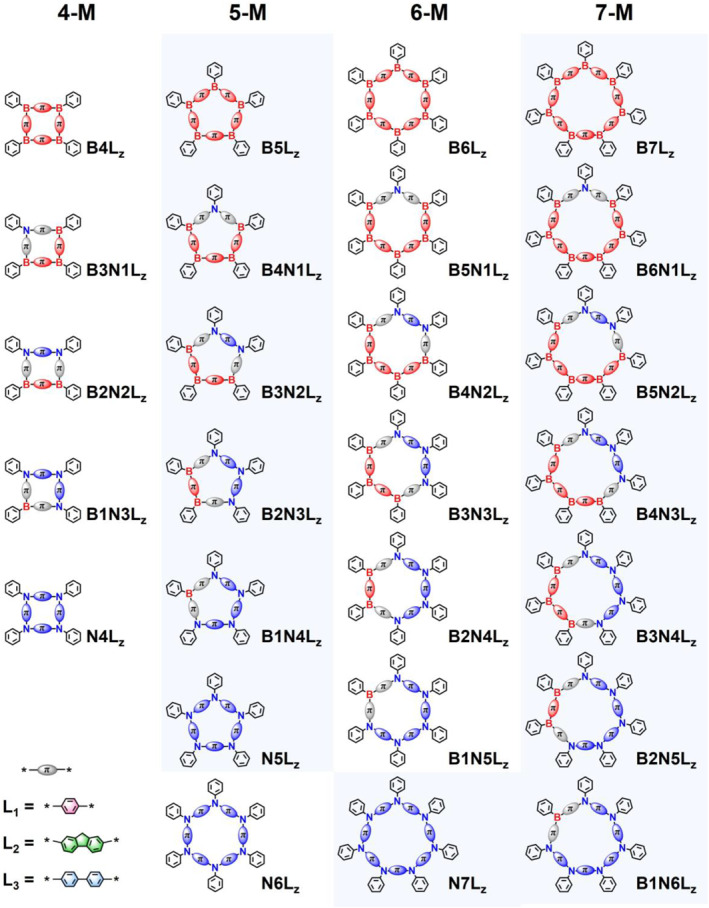
Molecular structures of the designed 78 molecules including fully B‐doped, hybrid B/N‐doped and fully N‐doped conjugated macrocycles, respectively, with ring size varying from 4‐M, 5‐M, 6‐M and 7‐M, and π‐linkers changing from L_1_, L_2_, to L_3_, respectively.

In this work, a series of 78 fully B‐doped, fully N‐doped, and hybrid B/N‐doped macrocycles were designed to elucidate the relationship between topological structures and spectral properties. Here three key topological factors, including ring size, number of Ar_3_B and Ar_3_N segments, as well as π‐linker between Ar_3_B and Ar_3_N segments, were considered in the molecular design of conjugated macrocycles (Figure 1). For L_1_‐based macrocycles, for a given ring size, both *λ*_abs_ and *λ*_emi_ first red‐shift and then blue‐shift as the number of Ar_3_N segments increases and the reddest *λ*_emi_ of each ring size usually contains a balanced ratio of Ar_3_B and Ar_3_N segments, with at least two Ar_3_B and Ar_3_N segments. In contrast, the influence of ring size on their spectra is not critical due to the reddest *λ*_emi_ of each size is comparable. Similar trend was also observed for L_2_‐ and L_3_ ‐based macrocycles. Regarding π‐linker effects, for B/N‐doped systems, at a given ring size and B/N ratio, L_1_‐based macrocycles display substantially red‐shifted emission compared to those with longer π‐linkers L_2_ and L_3_. Additionally, L_2_‐based macrocycles show slightly red‐shifted emission relative to L_3_‐based ones. This work establishes a rational molecular design strategy and provides important theoretical guidance for the spectral regulation of smart luminescent materials based on conjugated macrocyclic.

## 2 COMPUTATIONAL METHODS

In this work, the geometry optimizations of all 78 macrocycles at the ground state (S_0_) and the lowest singlet state (S_1_) were performed by using density functional theory (DFT) and time‐dependent DFT, respectively. The geometry optimizations at S_1_ were based on the S_0_‐optimized structure. For each optimized structure, the normal model analysis was performed at the same level to ensure no imaginary frequencies, and the solvation model based on solute electron density^[46]^ was used to simulate the toluene solvent condition. The corresponding absorption and emission spectra of macrocycles considering the S_0_ → S_1_ transition were calculated based on the optimized geometric structures at S_0_ and S_1_ respectively. All the calculations above were performed using the Gaussian 16 program.^[47]^

To accurately describe the spectral properties of designed conjugated macrocycles, the density functionals were first selected from a series of density functionals with different proportions of HF combined with 6‐31G(d) basis set, including B3LYP,^[48]^ PW6B95,^[49]^ PBE0‐1/3,^[50]^ PBE0‐3/8,^[51]^ BMK,^[52]^ and M06‐2X^[53]^ to well reproduce the experimental absorption and emission spectra of the reference macrocyclic molecule B3N3L_1_ and B1N5L_1_ in toluene,^[45]^ see Supporting Information S1: Table S1. It is found that the calculated *λ*_emi_ of B3N3L_1_ and B1N5L_1_ by PBE0‐1/3 (615 and 507 nm) show the best agreement with experimental results (612 and 520 nm), see Supporting Information S1: Table S1. It is necessary to mention that, the saturated alkyl groups of MC‐b‐B3N3 in the experiment were substituted by hydrogen, named as B3N3L_1_, and the calculated *λ*_emi_ of B3N3L_1_ (615 nm) was close to MC‐b‐B3N3 (639 nm), because of its neglectable effects on the emission properties of macrocyclic dyes (Supporting Information S1: Table S1). The influence of basis sets on the spectral properties of B/N‐doped macrocycles were also investigated. It is found that the spectral properties of B1N5L_1_ and B3N3L_1_ obtained by three basis sets (including 6‐31G (d), 6‐31G (d, p) and 6‐311G (d, p)) are highly consistent (see Supporting Information S1: Table S2). Therefore, the PBE0‐1/3/6‐31G(d) method can be reliably chosen to calculate the geometric structures and photophysical properties of B/N‐doped macrocycles. The Cartesian coordinates of all studied 78 macrocycles at both S_0_‐optimized and S_1_‐optimized structures were provided in the Supporting Information, see details in Structures S1‐S78.

To investigate the transition properties of the designed macrocycles, the natural transition orbital (NTO) analysis, the spatial overlap integral (*S*_r_) and centroid distance (*D*) between the hole and electron distributions^[54]^ based on optimized structures at S_0_ and S_1_ were calculated by using Multiwfn3.8(dev) package.^[55]^ The molecular planarity parameter (MPP)^[56]^ calculated using Multiwfn3.8(dev) package^[55]^ was used to quantify the planarity of the designed macrocycles. A plane was first fitted using the least‐squares method based on the atomic coordinates of the molecule, such that the plane passes through the geometric center of the atoms within the molecule. The MPP value is defined as the root‐mean‐square of the distances from each atom to this fitted plane: $\text{MPP}=\sqrt{\frac{1}{N}\sum_{i=1}^{N}d_{i}^{2}}$.

## 3 RESULTS AND DISCUSSIONS

### 3.1 Design of fully B‐doped, fully N‐doped and hybrid B/N‐doped conjugated macrocycles

To elucidate the relationship between topological structures and spectral properties, a series of conjugated macrocycles were systematically designed. These macrocycles vary in the number of Ar_3_B or Ar_3_N segments connected by different π‐linkers, as illustrated in Figure 1. The molecular design principle followed three main considerations. First, macrocycles of different ring sizes were constructed by varying the total number of incorporated Ar_3_B or Ar_3_N segments, ranging from four‐membered (4‐M) to seven‐membered (7‐M) rings. Second, for each ring size, fully B‐doped, fully N‐doped, and hybrid B/N doped macrocycles were designed by varying the relative numbers of Ar_3_B and Ar_3_N segments. Third, given the key impacts of π‐linkers on spectral properties, three representative π‐linkers including phenyl, fluorene, and biphenyl were incorporated into conjugated macrocycles. The fully B‐doped macrocycles were designated as B*x*L_*z*_, the fully N‐doped macrocycles were designated as N*y*L_*z*_, and the hybrid B/N‐doped macrocycles were designated as B*x*N*y*L_*z*_, respectively. Where “B”, “N” and “L” denote Ar_3_B segment, Ar_3_N segment and π‐linkers, respectively, and *x*, *y*, *z* indicate the corresponding counts or indices. For a given ring size, *x* and *y* each range from 1 to the total number of segments in the ring (e.g., 1–6 for 6‐M). The π‐linkers were specified as L_1_ for phenyl group, L_2_ for fluorene group, and L_3_ for biphenyl group, respectively. Considering the variation of ring size, B/N composition, and π‐linker, a total of 78 conjugated macrocycles were designed: 15 for 4‐M, 18 for 5‐M, 21 for 6‐M, and 24 for 7‐M. The chemical structures of all the systems were presented in Figure 1 and Supporting Information S1: Figures S2–S4 in Supporting Information.

### 3.2 Absorption and emission spectra of the designed macrocycles with L_1_ as π‐linker

**FIGURE 2:**
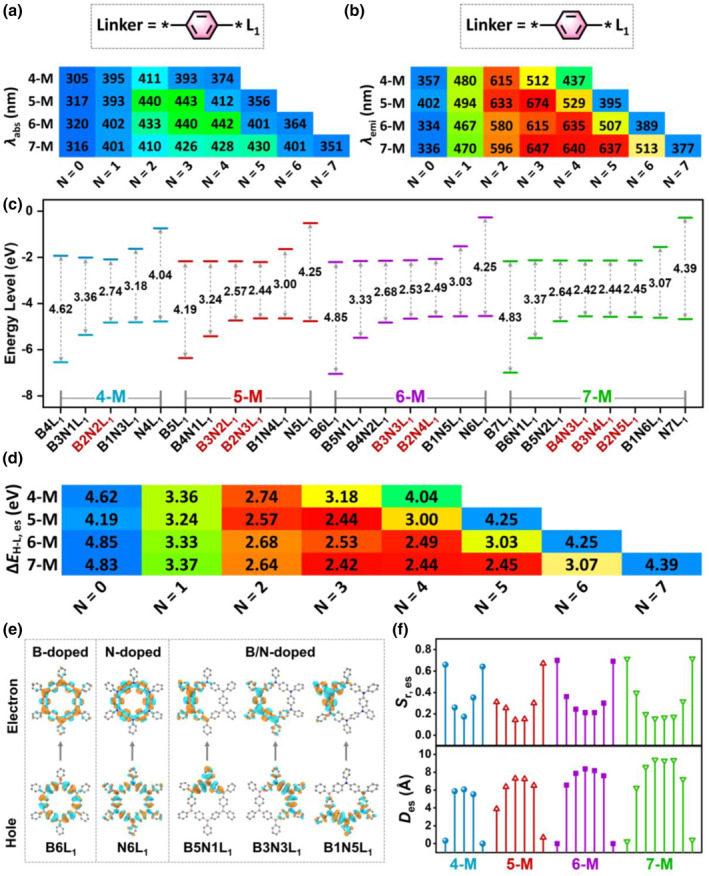
(a) *λ*_abs_, (b) *λ*_emi_ of the designed B/N doped macrocycles with L_1_ as the π‐linker. (c) HOMO and LUMO energy level, (d) HOMO‐LUMO energy gap (Δ*E*_H–L, es_) and (f) *S*_r, es_ and *D*_es_ indexes for L_1_‐based macrocycles at S_1_‐optimized structure. (e) NTOs of representative macrocycles: B6L_1_, N6L_1_, B5N1L_1_, B3N3L_1_ and B1N5L_1_ at S_1_‐optimized structure. HOMO, highest occupied molecular orbital; LUMO, lowest unoccupied molecular orbital.

For designed 26 macrocycles with the most commonly π‐linker L_1_, both the calculated *λ*_abs_ and *λ*_emi_ show strong dependence on the number of Ar_3_B or Ar_3_N segments, while the influence from ring size is neglectable (Figure 2a,b). At each ring size (4‐M to 7‐M), as the number of Ar_3_N segments increases (and Ar_3_B segments decreases accordingly), both *λ*_abs_ and *λ*_emi_ display a consistent trend of initial red‐shift followed by blue‐shift. Notably, *λ*_emi_ is more sensitive than *λ*_abs_ to the change of macrocyclic composition. The most pronounced red‐shifted emission for a given ring size occurs in macrocycles containing comparable numbers of Ar_3_N and Ar_3_B segments. For instance, within 4‐M the reddest *λ*_emi_ (>600 nm) is obtained for B2N2L_1_; for 5‐M, B3N2L_1_ and B2N3L_1_; for 6‐M, B3N3L_1_ and B2N4L_1_; and for 7‐M, B4N3L_1_, B3N4L_1_ and B2N5L_1_ are with the reddest emission, respectively (see Figure 2b). Therefore, across ring size from 4‐M to 7‐M, the reddest emission under a given ring size consistently corresponds to macrocycles that contain at least two Ar_3_B and Ar_3_N segments. Interestingly, the most significant changes in *λ*_emi_ are observed when the number of Ar_3_B segments increases from one to two, or similarly when the number of Ar_3_N segments rises from one to two. Taking the 6‐M macrocycles as an example, the emission has a significant red‐shift of 113 nm from B5N1L_1_ (467 nm) to B4N2L_1_ (580 nm). Likewise, a pronounced red‐shift of 128 nm (from 507 to 635 nm) occurs when moving from B1N5L_1_ to B2N4L_1_. While from B4N2L_1_ to B3N3L_1_, there is only a modest red‐shift of 35 nm (from 580 to 615 nm). This behavior is supported by the variation in energy levels of the highest occupied molecular orbital (HOMO) and the lowest unoccupied molecular orbital (LUMO) at S_1_‐optimized structures for L_1_‐based macrocycles (Figure 2c) due to the major contribution of HOMO → LUMO transition (Supporting Information S1: Table S3). For each ring size, as the number of Ar_3_N segments increases from 0 to 2, the HOMO energy rises significantly, indicating a marked enhancement in electron‐donating ability. Correspondingly, as the number of Ar_3_B segments increases from 0 to 2, the LUMO energy decreases considerably, reflecting a strong improvement in electron‐accepting ability. Beyond two segments of either type, however, the HOMO or LUMO energies approach plateaus. Consequently, when the numbers of Ar_3_B and Ar_3_N segments are both beyond two and approximately balanced, the macrocycles exhibit the reddest emission and the smallest energy gap (Δ*E*_H–L, es_) because of the simultaneously strong electron‐donating and electron‐accepting abilities (Figure 2c,2d).

Compared with hybrid B/N‐doped macrocycles, both *λ*_abs_ and *λ*_emi_ of fully B‐doped or fully N‐doped macrocycles are significantly blue‐shifted. And for each ring size of macrocycles, the *λ*_emi_ of fully N‐doped macrocycles consistently red‐shifts than that of the fully B‐doped counterparts, for example, *λ*_emi_ of N6L_1_ (389 nm) notably red‐shifts than B6L_1_ (334 nm). Moreover, for a given ring size (4‐M to 7‐M), varying the relative numbers of Ar_3_B and Ar_3_N segments enables broad tuning of *λ*_emi_ from below 400 nm to beyond 600 nm (Figure 2b). Thus, the spectral properties of hybrid B/N doped macrocycles with L_1_ π‐linkers are predominantly governed by the relative ratio of Ar_3_B to Ar_3_N segments, and the influence of ring size is negligible.

**FIGURE 3:**
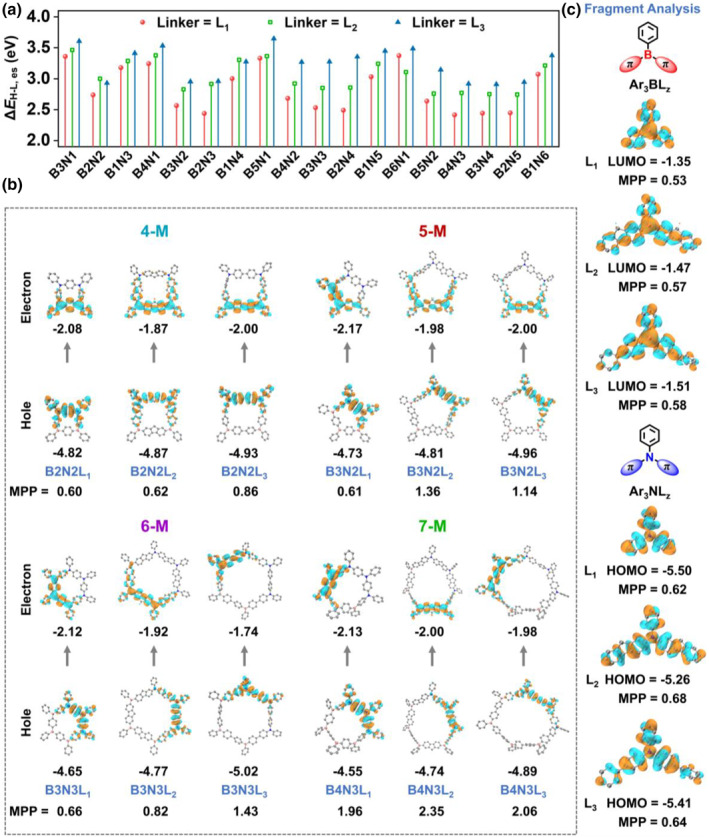
(a) Δ*E*_H–L, es_ values of hybrid B/N‐doped macrocycles with L_z_ (*z* = 1~3) as π‐linkers. (b) NTOs of B2N2L_z_, B3N2L_z_, B3N3L_z_, and B4N3L_z_ with three different π‐linkers at S_1_‐optimized structure. (c) The density distribution and energy level of LUMO for Ar_3_BL_z_ (*z* = 1~3), and density distribution and energy level of HOMO for Ar_3_NL_z_ (*z* = 1~3), as well as the corresponding MPP values. HOMO, highest occupied molecular orbital; LUMO, lowest unoccupied molecular orbital; MPP, molecular planarity parameter.

Natural transition orbital (NTO) analysis for representative L_1_‐based macrocycles at S_1_ was performed to further characterize the excited‐state properties (Figure 2e and Supporting Information S1: Figure S9). It is revealed that fully B‐doped (e.g., B6L_1_) and fully N‐doped (e.g., N6L_1_) macrocycles exhibit predominantly local excited character (Figure 2e). In contrast, hybrid B/N‐doped macrocycles clearly display an intramolecular charge transfer (ICT) feature with electron density distributed on Ar_3_B segments and hole density concentrated on Ar_3_N segments, as shown for B3N3L_1_, B1N5L_1_ and B5N1L_1_ in Figure 2e. However, the hole or electron of ICT‐type B/N‐doped macrocycles cannot fully delocalize across all Ar_3_N or Ar_3_B units because of their limited effective conjugation length. The maximum delocalization span for either Ar_3_N or Ar_3_B segments is approximately three units, which restricts the extent to which electron‐donating ability can be enhanced by increasing Ar_3_N segments, and similarly limits the improvement of electron‐accepting capacity through additional Ar_3_B segments. For instance, the electron of B5N1L_1_ is delocalized only about 2∼3 Ar_3_B segments, and the hole of B1N5L_1_ is distributed on only 2~3 Ar_3_N segments. As a result, both B5N1L_1_ and B1N5L_1_ exhibit relatively blue‐shifted emission compared with B3N3L_1_, where the hole and electron are well distributed across all available Ar_3_N or Ar_3_B segments, owing to its balanced numbers of both units (Figure 2e). This leads to both strong electron‐donating and electron‐accepting capabilities of B3N3L_1_. Notably, *λ*_emi_ of B3N4L_1_ in 7‐M is similar to that of B3N3L_1_ in 6‐M, implying that further increasing either Ar_3_N segments or ring size does not produce a substantial spectral red‐shift, supported by the limited spatial delocalization of hole and electron across the Ar_3_N and Ar_3_B segments of macrocycles, respectively (Figure 3b). The strength of ICT effect of the hybrid B/N‐doped macrocycles was further quantified using the spatial overlap integral (*S*_r_) and centroid distance (*D*) between the hole and electron distributions. Generally, a smaller *S*_r_ value coupled with a larger *D* value corresponds to the greater charge separation and an enhanced ICT effect. For each ring size of B/N doped macrocycles (4‐M to 7‐M), as the number of Ar_3_N segments increases (with a corresponding decrease in Ar_3_B segments), *S*_r, es_ displays an initial decrease followed by an increase, while *D*_es_ shows an opposite trend (Figure 2f). Thus, the red‐shifted spectra observed in macrocycles containing at least two Ar_3_B and Ar_3_N segments, relative to those with only one of each, are ascribed to an enhanced ICT effect.

Typically, the degree of orbital delocalization is closely related to molecular structural planarity, which can be quantified by the MPP.^[56]^ A larger MPP value indicates a lower molecular planarity. As shown in Supporting Information S1: Figure S10, the MPP values for 7‐M macrocycles are substantially higher than those of other ring sizes. For instance, considering macrocyles with the reddest *λ*_emi_ at each size B4N3L_1_ (7‐M), B3N3L_1_ (6‐M), B2N3L_1_ (5‐M), and B2N2L_1_ (4‐M), their MPP values at S_1_‐optimized structures are 1.96, 0.66, 0.62, and 0.60 Å, respectively. The relatively distorted geometry of the 7‐M series limits their orbital delocalization, thereby restricting the extent of red‐shifted emission in B4N3L_1_ compared to B3N3L_1_. The molecular planarity of macrocycles was also characterized by representative geometric parameters, such as the dihedral angles between four adjacent B/N heteroatoms, and the torsional angles between π‐linkers and exocyclic phenyls for macrocycles (Supporting Information S1: Figure S11). It was found that the planarity of the 4‐M to 6‐M macrocycles are quite good, because the dihedral angles among the four adjacent B/N heteroatoms of representative B2N2L_1_, B3N2L_1_, B3N3L_1_ are all below 10°. However, the corresponding dihedral angles of representative macrocycles B4N3L_1_ in 7‐M are much larger, indicating the weak planarity of 7‐M macrocycles.

**FIGURE 4:**
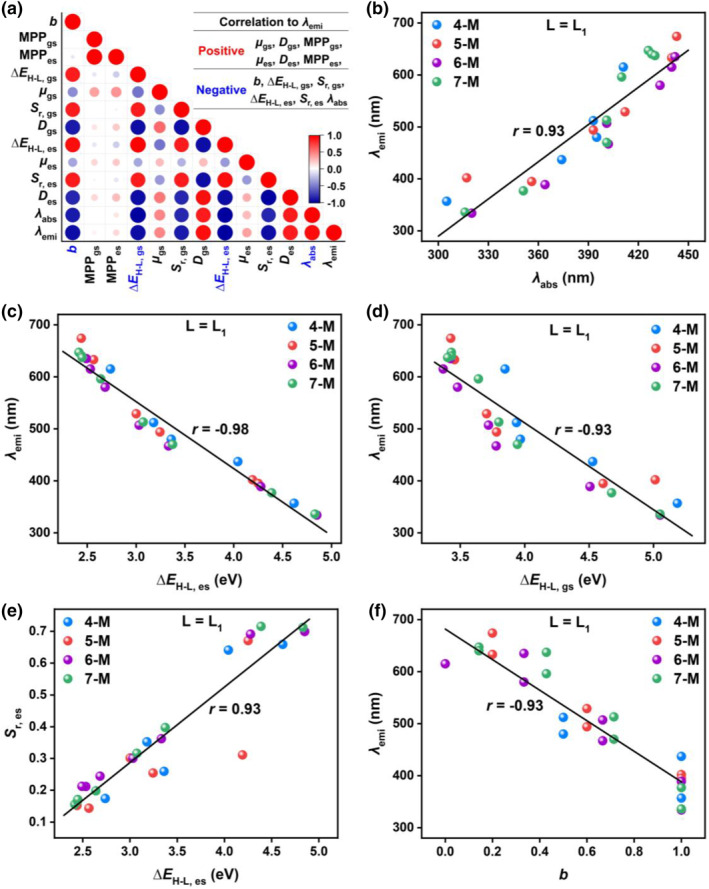
(a) Pearson's correlation coefficients matrix of the descriptors as well as *λ*_abs_ and *λ*_emi_ of the L_1_‐based macrocycles. The fitted line and linear correlation between (b) *λ*_abs_ and *λ*_emi_ (c) *λ*_emi_ and Δ*E*_H–L, es_, (d) *λ*_emi_ and Δ*E*_H–L, gs_, (e) *S*_r, es_ and Δ*E*_H–L, es_, (f) *λ*_emi_ and *b* of L_1_‐based macrocycles.

To better understand the variations in spectral properties of conjugated macrocycles, we evaluated the Pearson’s correlation coefficient (*r*) among a series of molecular descriptors, and between these descriptors and *λ*_abs_/*λ*_emi_ (Figure 4a and Supporting Information S1: Figure S12a), where a higher |*r*| indicates a stronger correlation. The descriptors fall into three categories: (1) a chemical‐component‐based descriptor *b*, defined as the excess index of Ar_3_B or Ar_3_N segments; (2) geometric‐structure‐based descriptors MPP_gs_/MPP_es_; and (3) electronic‐structure‐based descriptors, including Δ*E*_H–L, gs_/Δ*E*_H–L, es_, molecular dipole moment (*μ*_gs_/*μ*_es_), *S*_r, gs_/*S*_r, es_, and *D*_gs_/*D*_es_. All descriptors at both S_0_ (gs) and S_1_ (es) were calculated for the macrocycles, except for the *b* value. It is found that a strong positive linear relationship exists between *λ*_abs_ and *λ*_emi_ for L_1_‐based macrocycles (*r* = 0.93, Figure 4b). Among the selected descriptors, Δ*E*_H–L, es_ shows the strongest correlation with *λ*_emi_ (*r* = ‒ 0.98, Figure 4c). Given the strong *λ*_abs_ − *λ*_emi_ correlation, descriptors that correlate highly with *λ*_emi_ are largely consistent with those for *λ*_abs_, accordingly Δ*E*_H–L, gs_ also correlates strongly with *λ*_emi_ (*r* = ‒ 0.93, Figure 4d). Furthermore, Δ*E*_H–L, es_ exhibits a strong correlation with *S*_r, es_ (*r* = 0.93, Figure 4e), suggesting that the observed spectral variation of macrocycles originates from changes in ICT strength. For our designed macrocycles, both the orbital energy levels and ICT strength are modulated by the ratio of Ar_3_B to Ar_3_N segments, promoting us to propose a new descriptor *b* ‒ the excess index of Ar_3_B or Ar_3_N segments. The *b* index is defined as *b* = 2|*x*_B_ − 0.5|, where *x*_B_ is the ratio of the number of Ar_3_B segments to the total number of Ar_3_B and Ar_3_N segments in the macrocycle. The *b* index ranges from 0 to 1, and values approaching zero indicate a more balanced ratio of Ar_3_B to Ar_3_N segments. As shown in Figure 4f, *λ*_emi_ exhibits a strong negative correlation with *b* (*r* = − 0.93). Therefore, regardless of the ring size of macrocycles, the ratio of Ar_3_B to Ar_3_N segments plays a crucial role in tuning the spectral properties. A more balanced ratio (e.g., B3N3L_1_ and B4N3L_1_) enhances both electron‐donating and electron‐accepting abilities, thereby strengthening the ICT effect and leading to a more redshifted emission spectra.

### 3.3 Absorption and emission spectra of macrocycles with L_2_ and L_3_ as π‐linkers

**FIGURE 5:**
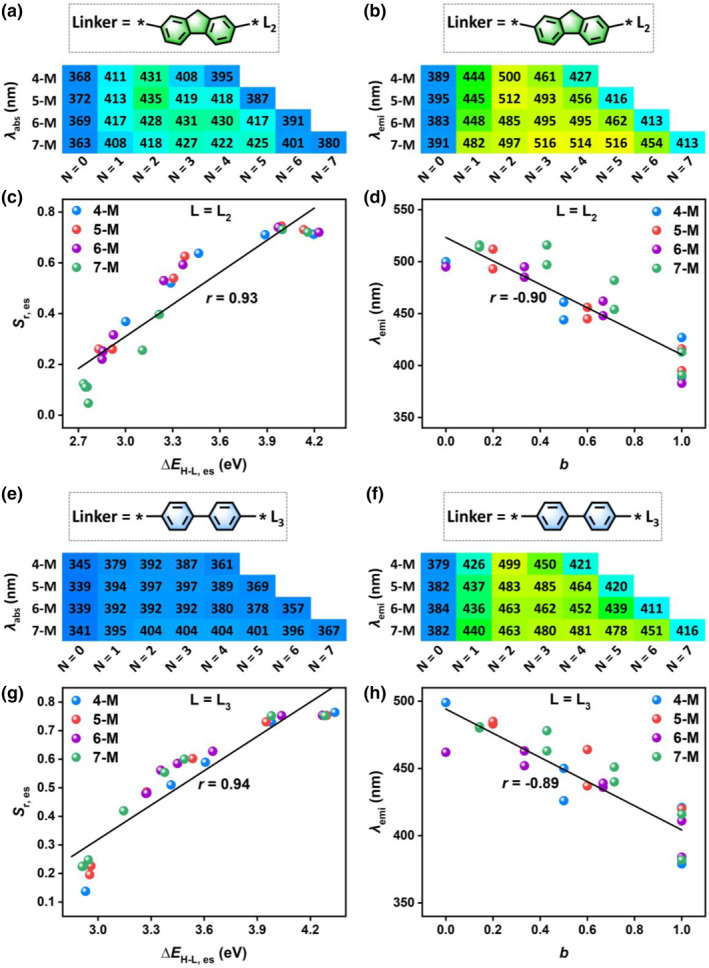
*λ*_abs_ and *λ*_emi_ of (a, b) L_2_‐based and (e, f) L_3_‐based macrocycles, respectively. The fitted line and linear correlation between *λ*_emi_ and Δ*E*_H–L, es_ of (c) L_2_‐based and (g) L_3_‐based macrocycles. The fitted line and linear correlation between *λ*_emi_ and *b* of (d) L_2_‐based and (h) L_3_‐based macrocycles.

The composition of π‐linkers significantly influences the spectral properties of conjugated macrocycles. To investigate this effect, two additional series of macrocycles were designed by replacing the π‐linker of L_1_‐based 26 macrocycles with π‐linkers L_2_ and L_3_, respectively. The corresponding *λ*_abs_ and *λ*_emi_ for these macrocycles are presented in Figure 5a,b (L_2_) and Figure 5e,f (L_3_). Consistent with the L_1_‐based macrocycles, fully B‐doped and fully N‐doped macrocycles containing L_2_ or L_3_ exhibit blue‐shifted spectra relative to their hybrid B/N doped counterparts. For the B/N doped macrocycles, both *λ*_abs_ and *λ*_emi_ are strongly governed by the relative numbers of Ar_3_B and Ar_3_N segments in the backbone. For each ring size of macrocycles, as the number of Ar_3_N segments increases (with a corresponding decrease in Ar_3_B segments), the emission spectra first red‐shift and then blue‐shift. The reddest *λ*_emi_ for each π‐linker consistently occurs in macrocycles with comparable numbers of Ar_3_B and Ar_3_N segments: for 4‐M are B2N2L_2/3_; for 5‐M are B3N2L_2/3_, for 6‐M are B3N3L_2/3_, for 7‐M are B4N3L_2/3_, B3N4L_2/3_ and B2N5L_2/3_, respectively. Also, the ring size of B/N doped macrocyles with L_2_ or L_3_ as π‐linkers has a negligible impact on emission spectra, as evidenced by the *λ*_emi_ values of the reddest‐emitting macrocyle at each ring size. For instance, *λ*_emi_ values for B2N2L_2_, B3N2L_2_, B3N3L_2_, and B4N3L_2_ are 500, 512, 495 and 516 nm, respectively.

Correlations between photophysical parameters and *λ*_abs_/*λ*_emi_ for macrocycles with L_2_ and L_3_ were also analyzed (Supporting Information S1: Figures S13a and S20a). For both types, *λ*_abs_ exhibits a strong positive linear correlation with *λ*_emi_ (*r* = 0.93 for L_2_ and *r* = 0.91 for L_3_, see Supporting Information S1: Figures S13b and S20b), and both Δ*E*_H–L, gs_ and Δ*E*_H–L, es_ exhibit strong correlation with *λ*_emi_ (|*r*| > 0.90, Supporting Information S1: Figures S13c,d and S20c,d). For each ring size of macrocycles with L_2_ or L_3_, the HOMO energy rises noticeably as the number of Ar_3_N segments increases from 0 to 2, while the LUMO energy decreases markedly as the number of Ar_3_B segments increases from 0 to 2 (Supporting Information S1: Figures S15a and S22a). However, beyond two units of either segments, the HOMO or LUMO energy largely plateaus (Supporting Information S1: Figures S15a and S22a). Moreover, Δ*E*_H–L, es_ exhibits a strong correlation with *S*_r, es_ (*r* = 0.93 for L_2_ and *r* = 0.94 for L_3_, Figure 5c,g), and the enhanced ICT effect further contributes to the observed spectral redshift, as evidenced by the strong correlation between *S*_r, es_ and *λ*_emi_ (*r* = −0.93 for L_2_ and *r* = −0.92 for L_3_, Supporting Information S1: Figures S13e and S20e). Specifically, as the number of Ar_3_N segments increases (and Ar_3_B segments decreases), *S*_r, es_ first decreases and then increases, while *D*_es_ follows the opposite trend (Supporting Information S1: Figures S17 and S24). Obviously, the variations in orbital energy levels and the ICT effect are both modulated by the ratio of Ar_3_B to Ar_3_N segments. The *b* index also exhibits a strong negative correlation with *λ*_emi_ (*r* = − 0.90 for L_2_ and *r* = − 0.89 for L_3_, Figure 5d,h); therefore, for macrocycles with L_2_ and L_3_ as π‐linkers, a relatively balanced ratio of Ar_3_B to Ar_3_N segments consistently induces a redshift in the emission spectra.

### 3.4 Effects of π‐linkers on spectral properties of macrocycles

A systematic understanding of how π‐linkers affect the spectral properties of fully B‐doped, fully N‐doped, and hybrid B/N‐doped macrocycles is essential for the rational design of high performance luminescent conjugated systems. For fully B‐doped macrocycles, the calculated *λ*_abs_ and *λ*_emi_ of the 6‐M system B6L_2_ (369 and 383 nm) are red‐shifted relative to those of B6L_1_ (320 and 334 nm), in good agree with experimental results.[^41^, ^42^] Similarly, for fully N‐doped macrocycles, the calculated *λ*_abs_ and *λ*_emi_ of N6L_2_ (391 and 413 nm) are also red‐shifted compared with N6L_1_ (364 and 389 nm) and N6L_3_ (357 and 411 nm), consistent with experimental results.^[43]^ In contrast, hybrid B/N doped macrocyles display the opposite trend. For a given ring size and fixed number of Ar_3_B and Ar_3_N segments, systems with L_1_ as π‐linkers exhibit significantly red‐shifted *λ*_emi_ relative to those with the longer π‐linkers L_2_ and L_3_. Moreover, L_2_‐based systems show a slight red‐shift compared with L_3_‐based analogs. For example, *λ*_emi_ values for B3N3L_1_, B3N3L_2_, and B3N3L_3_ are 615 nm, 495 and 462 nm, respectively (Figures 2b and 5b,f). This trend is strongly supported by the regular increase in Δ*E*_H–L,__es_ as the π‐linkers change from L_1_ to L_3_ (Figure 3a). Specifically, the HOMO energies of L_1_‐based macrocycles are higher, while their LUMO energies are lower than those of L_2_‐ and L_3_‐based systems (Figure 3b and Supporting Information S1: Figure S27). That indicates that both the electron‐donating and electron‐accepting capacities of hybrid B/N‐doped macrocycles are markedly stronger for those with L_1_ than for those with L_2_ or L_3_. Additionally, the degrees of hole and electron delocalization for L_1_‐based macrocycles at S_1_ are higher than those for L_2_ and L_3_ analogs (Figure 3b, Supporting Information S1: Figures S9, S19 and S26). In B3N3L_1_, both hole and electron densities are delocalized across all three Ar_3_N and three Ar_3_B segments, respectively. In contrast, for B3N3L_2_ and B3N3L_3_, the hole and electron distributions remain only partially localized on the respective Ar_3_N and Ar_3_B segments (Figure 3b). Thus, longer π‐linkers (such as L_2_ and L_3_) appear to restrict the delocalization of electrons and holes, thereby limiting the full expression of the electron‐donating and electron‐accepting capabilities of the connected Ar_3_B and Ar_3_N units. This constraint ultimately leads to the observed blue‐shifted emission spectra of L_2_‐based and L_3_‐based macrocycles.

All macrocycles examined here are constructed from repeating Ar_3_B and Ar_3_N segments bridged by different π‐linkers, thus their overall properties are likely closely related to the characteristics of the fundamental building blocks Ar_3_BL_z_ and Ar_3_NL_z_ (*z* = 1–3), as shown in Figure 3c. The MPP values for both Ar_3_BL_1_ and Ar_3_NL_1_ are lower than those of their L_2_ and L_3_ counterparts (Ar_3_BL_2_, Ar_3_NL_2_, Ar_3_BL_3_ and Ar_3_NL_3_). Since the conformations of these building blocks directly influence the overall molecular framework, hybrid B/N‐doped macrocycles with L_1_ as π‐linkers exhibit lower MPP values at S_1_ and hence greater planarity than those of L_2_ and L_3_‐based macrocycles (Figure 3b). For instance, the MPP values for B3N3L_1_, B3N3L_2_ and B3N3L_3_ are 0.66, 0.82 and 1.43 Å, respectively. Consequently, L_1_‐based hybrid B/N‐doped macrocycles adopt a more planar geometry and possess a higher degree of conjugation then those of L_2_‐based or L_3_‐based analogies, which facilitates the enhancement of electron and hole delocalization. The two phenyl rings within the biphenyl linker exhibit a large torsional angle, which further reduces the overall planarity of L_2_‐based macrocycle (Supporting Information S1: Figure S29). The macrocycles we designed can be regarded as the “D–π–A” structure, as shown in Supporting Information S1: Figure S28. Here, we also calculated the average values of the rotational angles of π‐linkers and exocyclic phenyls in the D segments, named *θ*_D_; the average values of the rotational angles of π‐linkers and exocyclic phenyls in the A segments, named *θ*_A_ (Supporting Information S1: Figures S28 and S29). It is found that at a constant ring size and relative number of Ar_3_B and Ar_3_N, *θ*_D_ and *θ*_A_ for L_1_‐based macrocycles are smaller than L_2_‐ and L_3_‐ based macrocycles, which induces a more planar structure. For example, *θ*_D_ for B3N3L_1_, B3N3L_2_, B3N3L_3_ are 38°, 40°, and 41°, respectively; *θ*_A_ for B3N3L_1_, B3N3L_2_, B3N3L_3_ are 29°, 31°, and 42°, respectively. Thus, such a geometric structure promotes the spectral redshift of L_1_‐based macrocycles. The bond lengths between each B/N atom and their adjacent C atoms in B2N2L_z_, B3N2L_z_, B3N3L_z_, and B4N3L_z_ (z = 1~3) at S_1_‐optimized structure are shown in Supporting Information S1: Figure S28 and Tables S6–S9. It is found that at a fixed number of Ar_3_B and Ar_3_N segments and ring size, the bond lengths change slightly with the variation of the π‐linker, which is evidenced by the difference between the maximum and minimum values of each bond length (Δ_max − min_) being less than 0.05 Å for macrocycles with different π‐linkers (Supporting Information S1: Tables S6–S9), indicating that the bond lengths have a negligible effect on the planarity of the macrocycles.

Moreover, L_2_‐based macrocycles generally exhibit slightly red‐shifted *λ*_emi_ compared to those of L_3_‐based macrocycles, supported by their lower Δ*E*_H–L,__es_ values. Further analysis of HOMO and LUMO energy levels at S_1_ reveals that L_2_‐ and L_3_‐based systems have comparable LUMO levels (e.g., −1.98 eV for B3N2L_2_ vs. −2.00 eV for B3N2L_3_), whereas the HOMO levels of L_2_‐based macrocycles are markedly higher (e.g., −4.81 eV for B3N2L_2_ vs. −4.96 eV for B3N2L_3_), see Figure 3b and Supporting Information S1: Figure S27. Thus, the reduced Δ*E*_H–L,__es_ of L_2_ series is primarily driven by the evaluation of the HOMO energy. This distinction can be traced back to the properties of fundamental building blocks. While the LUMO energies of Ar_3_BL_2_ and Ar_3_BL_3_ are similar, the HOMO energy of Ar_3_NL_2_ is significantly higher than that of Ar_3_NL_3_ (Figure 3c). Therefore, compared with the L_3_ π‐linker, the more rigid structure of L_2_ enhances the conjugation between adjacent Ar_3_N segments more effectively, while exerting a less influence on Ar_3_B segments. This selective enhancement ultimately modulates the emission characteristics of the entire macrocycle.

## 4 CONCLUSIONS

In summary, a series of 78 fully B‐doped, fully N‐doped, and hybrid B/N‐doped macrocycles were designed to elucidate the relationship between topological structures and spectral properties. These macrocycles differ in three key structural factors, including ring size, number of Ar_3_B and Ar_3_N segments, as well as π‐linkers connecting them. For L_1_‐based macrocycles, for a given ring size, both *λ*_abs_ and *λ*_emi_ first red‐shift and then blue‐shift as the number of Ar_3_N segments increases. In contrast, ring size exerts negligible influence on spectral properties, evidenced by the comparable maximum *λ*_emi_ across different ring sizes. Correlation analysis indicates that Δ*E*_H–L, es_ shows the strongest correlation with *λ*_emi_ (*r* = ‒ 0.98). Furthermore, Δ*E*_H–L, es_ exhibits a strong correlation with *S*_r, es_, suggesting that the observed spectral variation of macrocycles originates from changes in ICT strength. For our designed macrocycles, both the orbital energy levels and ICT strength are modulated by the ratio of Ar_3_B to Ar_3_N segments, promoting us to propose a new descriptor *b*‒ the excess index of Ar_3_B or Ar_3_N segments. *λ*_emi_ exhibits a strong negative correlation with *b* (*r* = ‒ 0.93). Therefore, regardless of the ring size of macrocycles, the ratio of Ar_3_B to Ar_3_N segments plays a crucial role in tuning the spectral properties. A more balanced ratio (e.g., B3N3L_1_ and B4N3L_1_) enhances both electron‐donating and electron‐accepting abilities, thereby strengthening the ICT effect and leading to a more redshifted emission spectra.

Similar spectral trends are observed for L_2_‐ and L_3_‐based macrocycles, where *λ*_emi_ initial red‐shift followed by blue‐shift with increasing Ar_3_N content, and ring size again shows minimal impact, consistent with L_1_‐based macrocycles. Regarding π‐linker effects, for hybrid B/N‐doped systems, at a given ring size and B/N ratio, L_1_‐based macrocycles display substantially red‐shifted emission compared to those with longer π‐linkers L_2_ and L_3_. This reversal stems from the more planar geometry and superior NTO delocalization in L_1_‐based macrocycles than those with L_2_‐ and L_3_‐based macrocycles. Additionally, L_2_‐based macrocycles show slightly red‐shifted emission relative to L_3_‐based ones. By clarifying the distinct roles of B/N composition, ring size, and π‐linker, this work establishes a rational design strategy for tuning the photophysical properties of conjugated macrocycles and provides valuable theoretical guidance for the development of smart materials with excellent luminescent performance based on conjugated macrocycles.

## CONFLICT OF INTEREST STATEMENT

The authors declare no conflicts of interest.

## ETHICS STATEMENT

No animal or human experiments were involved in this study.

## Supporting information

Supporting Information S1

## Data Availability

The data that support the findings of this study are available from the corresponding author upon reasonable request.
