# Supplementary material for: Unraveling the effect of topological structures of B/N‐doped conjugated macrocycles on spectral properties: A computational study
Source: Smart Mol. 2026 Jun 10:e70066. Online ahead of print. doi: 10.1002/smo2.70066 (PMC13399027; doi:10.1002/smo2.70066)
Supplement: Supplementary file 1 — Supporting Information S1 [file SMO2-9999-0-s001.pdf]

Supporting Information

Unraveling the Effect of Topological Structures of B/N-Doped Conjugated Macrocycles on Spectral Properties: A Computational Study

Wen-Yu Guo,[1] Yi Zeng,[1] Yu-Meng Xu,[1] Pang-Kuan Chen,[1] and Xiao-Yan Zheng*[1]

[1]Key Laboratory of Cluster Science of Ministry of Education, State Key Laboratory of Environment Characteristics and Effects for Near-space, Beijing Key Laboratory of Intelligent Molecular Materials and High-throughput Manufacturing, School of Chemistry and Chemical Engineering, Beijing Institute of Technology, Beijing 100081, P. R. China.

E-mail: xiaoyanzheng@bit.edu.cn

Table of Contents

1. Supplementary Figures 4

Figure S1. Chemical structures of **MC-b-B3N3** and **B3N3L1**. 4

Figure S2. Chemical structures of the fully B-doped, fully N-doped and B/N-doped macrocycles with $\mathbf{L_1}$ as π-linker. 5

Figure S3. Chemical structures of the fully B-doped, fully N-doped and B/N-doped macrocycles with $\mathbf{L_2}$ as π-linker. 6

Figure S4. Chemical structures of fully B-doped, fully N-doped and B/N-doped the macrocycles with $\mathbf{L_3}$ as π-linker. 7

Figure S5. The energy difference between S_2 and S_1 of all studied 78 macrocycles based on their S_1-optimized structures 8

Figure S6. (a) HOMO and LUMO energy levels, as well as (b) their energy gap ($\Delta E_{\text{H-L, gs}}$) of $\mathbf{L_1}$-based macrocycles at S_0-optimized structure. 9

Figure S7. $S_{\text{r, gs}}$ and D_{gs} values of $\mathbf{L_1}$-based macrocycles at S_0-optimized structure. The order of molecules is consistent with those in **Figure S5a**. 10

Figure S8. NTOs of $\mathbf{L_1}$-based macrocycles at S_0-optimized structure. 11

Figure S9. NTOs of $\mathbf{L_1}$-based macrocycles at S_1-optimized structure. 13

Figure S10. MPP values of $\mathbf{L_1}$-based macrocycles at both S_0 and S_1-optimized structures. The order of molecules is consistent with those in **Figure S6a** 15

Figure S11. The geometric parameters for macrocycles: **B2N2L1**, **B3N2L1**, **B3N3L1**, and **B4N3L1** at S_1-optimized structure. 16

Figure S12. (a) Pearson's correlation coefficients matrix of the descriptors as well as λ_{abs} and λ_{emi} of the $\mathbf{L_1}$-based macrocycles. The fitted line and linear correlation between (b) λ_{emi} and $S_{\text{r, es}}$, (c) λ_{emi} and $S_{\text{r, gs}}$ of $\mathbf{L_1}$-based macrocycles. 17

Figure S13. (a) Pearson's correlation coefficients matrix of the descriptors as well as λ_{abs} and λ_{emi} of $\mathbf{L_2}$-based macrocycles. The fitted line and linear correlation between (b) λ_{abs} and λ_{emi}, (c) λ_{emi} and $\Delta E_{\text{H-L, es}}$, (d) λ_{emi} and $\Delta E_{\text{H-L, gs}}$, (e) λ_{emi} and $S_{\text{r, es}}$, (f) λ_{emi} and $S_{\text{r, gs}}$ of $\mathbf{L_2}$-based macrocycles 18

Figure S14. (a) HOMO and LUMO energy levels, as well as (b) their energy gap ($\Delta E_{\text{H-L, gs}}$) of $\mathbf{L_2}$-based macrocycles at S_0-optimized structure. 19

Figure S15. (a) HOMO and LUMO energy levels, as well as (b) their energy gap ($\Delta E_{\text{H-L, es}}$) of $\mathbf{L_2}$-based macrocycles at S_1-optimized structure. 20

Figure S16. $S_{\text{r, gs}}$ and D_{gs} values of $\mathbf{L_2}$-based macrocycles at S_0-optimized structure. The order of molecules is consistent with those in **Figure S14a**. 21

Figure S17. $S_{\text{r, es}}$ and D_{es} values of $\mathbf{L_2}$-based macrocycles at S_1-optimized structure. The order of molecules is consistent with those in **Figure S14a**. 22

Figure S18. NTOs of $\mathbf{L_2}$-based macrocycles at S_0-optimized structure. 23

Figure S19. NTOs of $\mathbf{L_2}$-based macrocycles at S_1-optimized structure. 25

Figure S20. (a) Pearson's correlation coefficients matrix of the descriptors as well as λ_{abs} and λ_{emi} of $\mathbf{L_3}$-based macrocycles. The fitted line and linear correlation between (b) λ_{abs} and λ_{emi}, (c) λ_{emi} and $\Delta E_{\text{H-L, es}}$, (d) λ_{emi} and $\Delta E_{\text{H-L, gs}}$, (e) λ_{emi} and $S_{\text{r, es}}$, (f) λ_{emi} and $S_{\text{r, gs}}$ of $\mathbf{L_3}$-based macrocycles 27

Figure S21. (a) HOMO and LUMO energy levels, as well as (b) their energy gap ($\Delta E_{\text{H-L, gs}}$) of $\mathbf{L_3}$-based macrocycles at S_0-optimized structure. 28

Figure S22. (a) HOMO and LUMO energy levels, as well as (b) their energy gap ($\Delta E_{\text{H-L, es}}$) of $\mathbf{L_3}$-based macrocycles. 29

Figure S23. $S_{r,\,gs}$ and D_{gs} values of **L3**-based macrocycles at S_0-optimized structure. The order of molecules is consistent with those in **Figure S20a.** 30

Figure S24. $S_{r,\,es}$ and D_{es} values of **L3**-based macrocycles at S_1-optimized structure. The order of molecules is consistent with those in **Figure S20a**. 31

Figure S25. NTOs of **L3**-based macrocycles at S_0-optimized structure. 32

Figure S26. NTOs of **L3**-based macrocycles at S_1-optimized structure. 34

Figure S27. HOMO and LUMO energy levels of the B/N doped macrocycles with different π-linkers at S_1-optimized structure. 36

Figure S28. (a) The schematic diagram of D and A segments, as well as the define of θ_A and θ_D for the B/N-doped macrocycles we designed (taking **B3N3L1** as an example). (b) The schematic diagram of atomic serial numbers for **B2N2Ln**, **B3N2Ln**, **B3N3Ln** and **B4N3Ln** (n = 1 ~ 3). The statistical results of the corresponding bond lengths are shown in **Table S6 ~ S9**. 37

Figure S29. The rotational angles of π-linkers and exocyclic phenyls for macrocycles **B2N2Ln**, **B3N2Ln**, **B3N3Ln** and **B4N3Ln** (n = 1 ~ 3) with different π-linkers at S_1-optimized structure. ... 38

2. Supplementary Tables **39**

Table S1. The calculated λ_{abs} and λ_{emi} of **MC-b-B3N3**, **B3N3L1**, and **B1N5L1** in toluene by functionals with different HF proportions combined with 6-31G(d) basis set 39

Table S2. The calculated λ_{abs} and λ_{emi} of **B1N5L1** and **B3N3L1** in toluene by 6-31G (d), 6-31G (d, p) and 6-311G (d, p) basis sets combined with PBE0-1/3 functional. 40

Table S3. Transition properties of **L1**-based macrocycles. 41

Table S4. Transition properties of **L2**-based macrocycles. 42

Table S5. Transition properties of **L3**-based macrocycles. 43

Table S6. The bond lengths (Å) between each B/N atoms and their adjacent C atoms in **B2N2Ln** (n = 1 ~ 3) at S_1-optimized structure. 44

Table S7. The bond lengths (Å) between each B/N atoms and their adjacent C atoms in **B3N2Ln** (n = 1 ~ 3) at S_1-optimized structure. 45

Table S8. The bond lengths (Å) between each B/N atoms and their adjacent C atoms in **B3N3Ln** (n = 1 ~ 3) at S_1-optimized structure. 46

Table S9. The bond lengths (Å) between each B/N atoms and their adjacent C atoms in **B4N3Ln** (n = 1 ~ 3) at S_1-optimized structure. 47

3. The coordinates of both the optimized structures of the macrocycles at S_0 and S_1 in toluene. **48**

1. Supplementary Figures

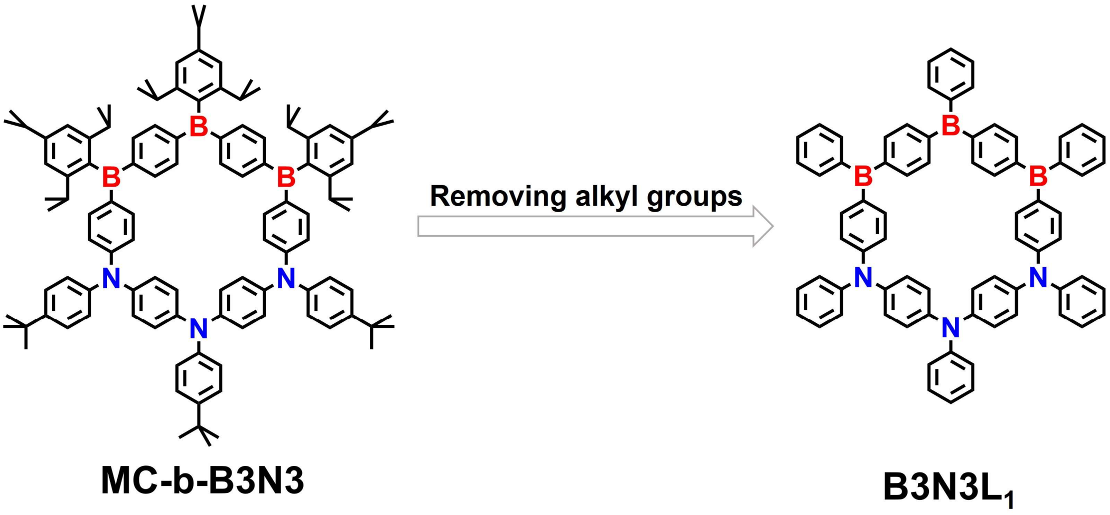

Figure S1. Chemical structures of **MC-b-B3N3** and **B3N3L1.**

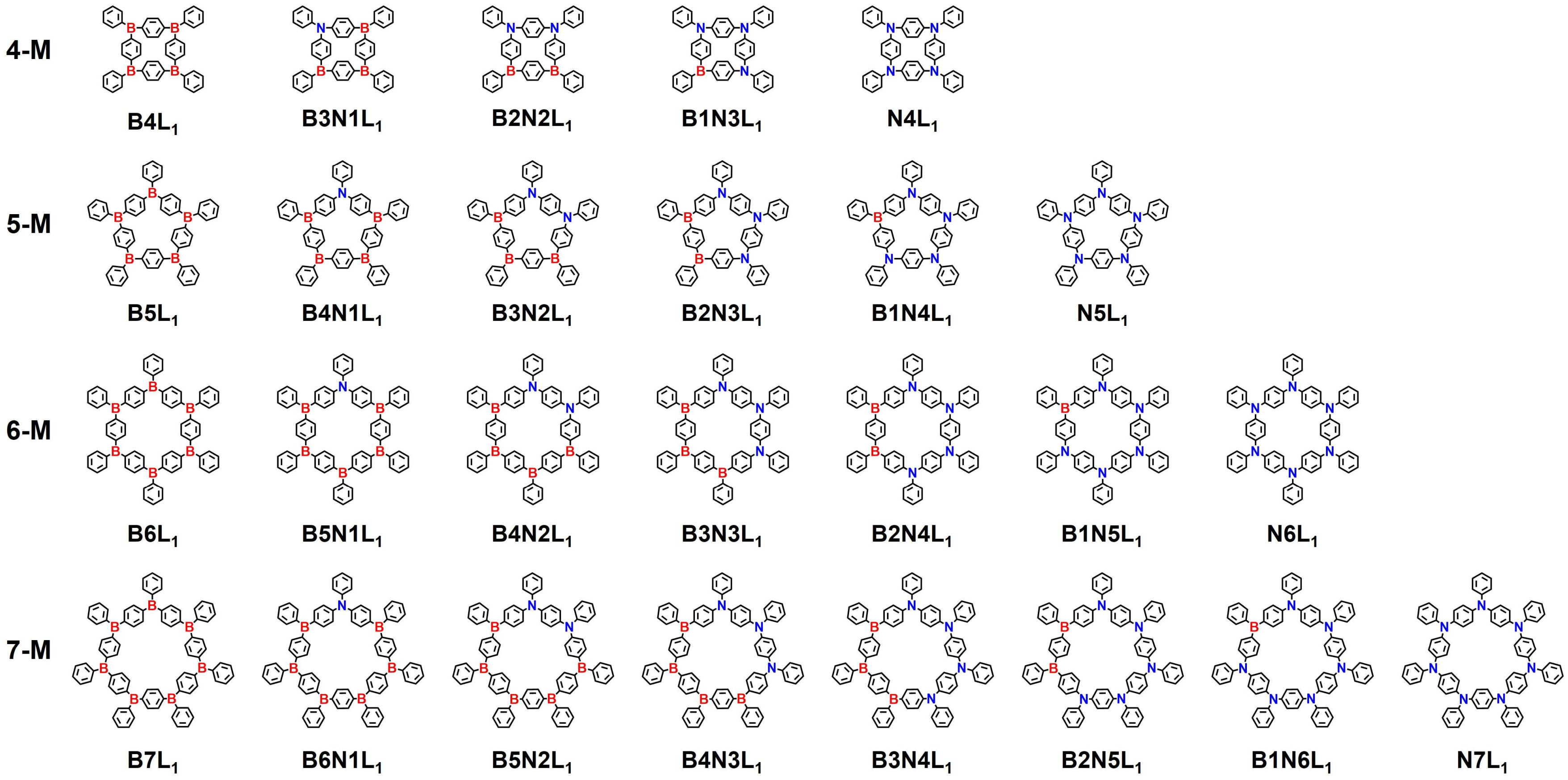

Figure S2. Chemical structures of the fully B-doped, fully N-doped and B/N-doped macrocycles with $\mathbf{L_1}$ as π-linker.

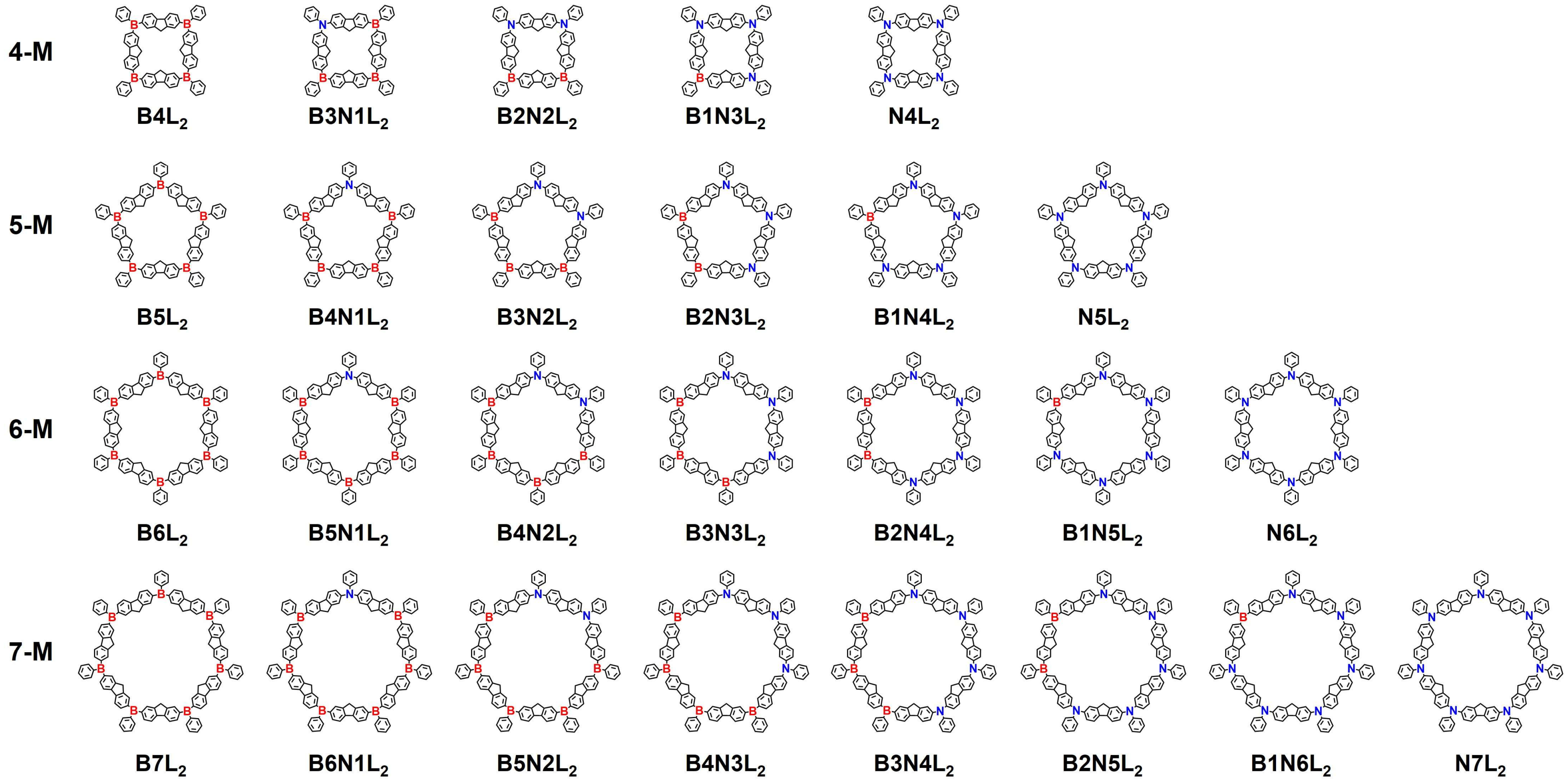

Figure S3. Chemical structures of the fully B-doped, fully N-doped and B/N-doped macrocycles with $\mathbf{L_2}$ as π-linker.

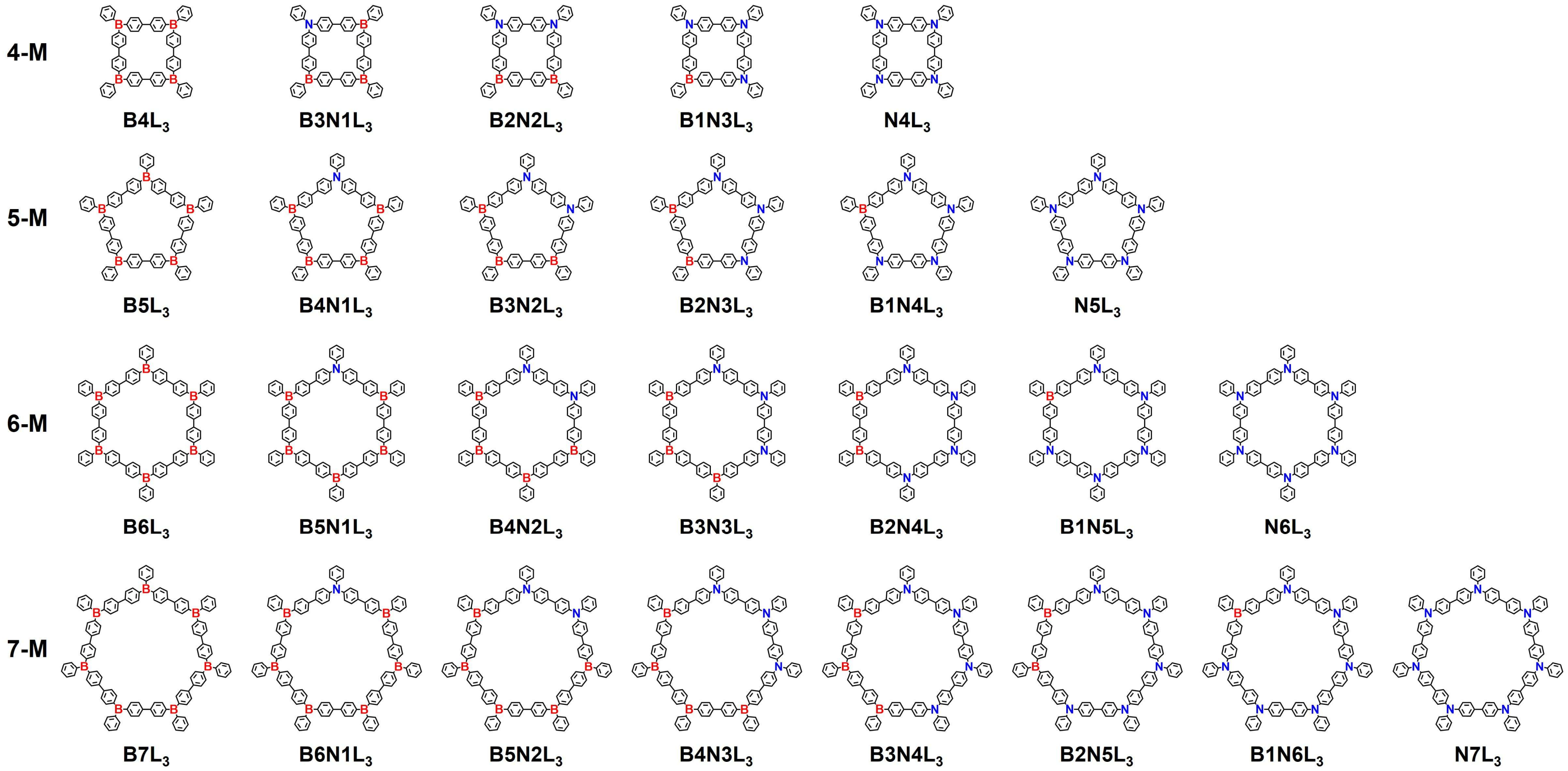

Figure S4. Chemical structures of fully B-doped, fully N-doped and B/N-doped the macrocycles with $\mathbf{L_3}$ as π-linker.

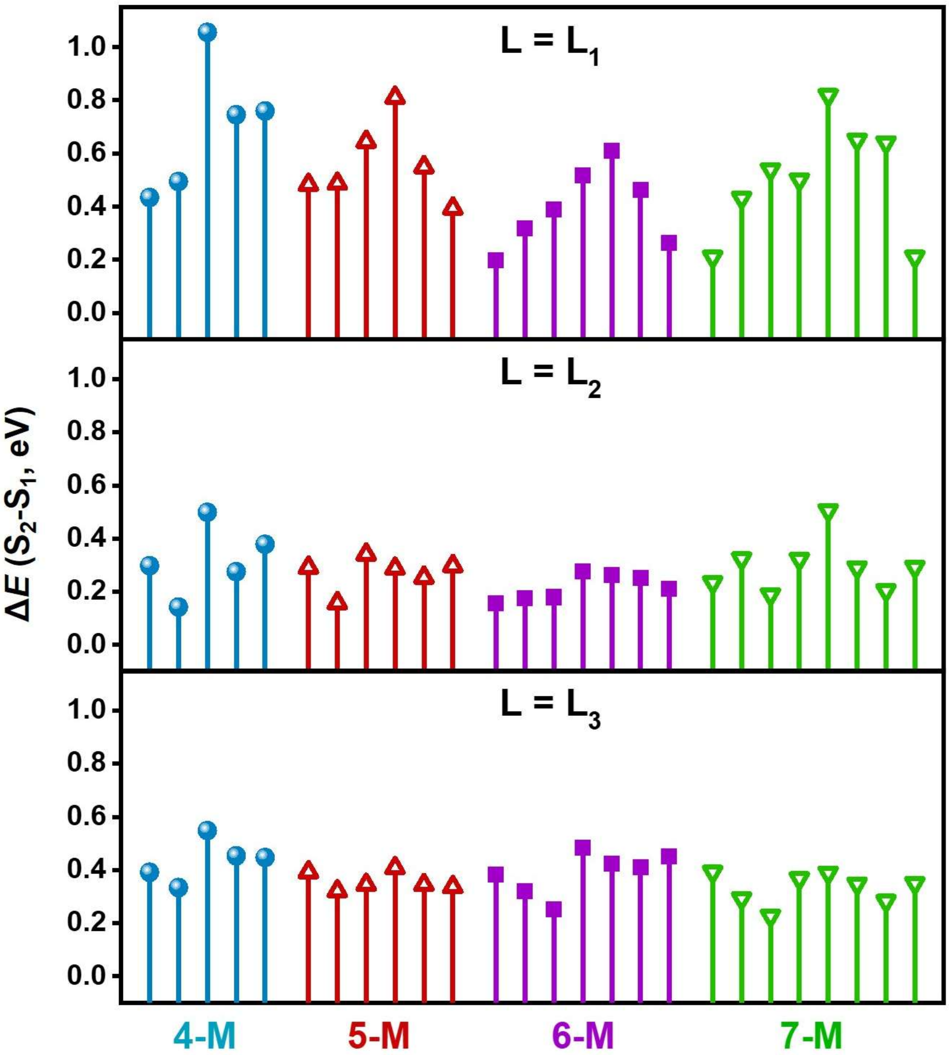

Figure S5. The energy difference between S_2 and S_1 of all studied 78 macrocycles based on their S_1-optimized structures.

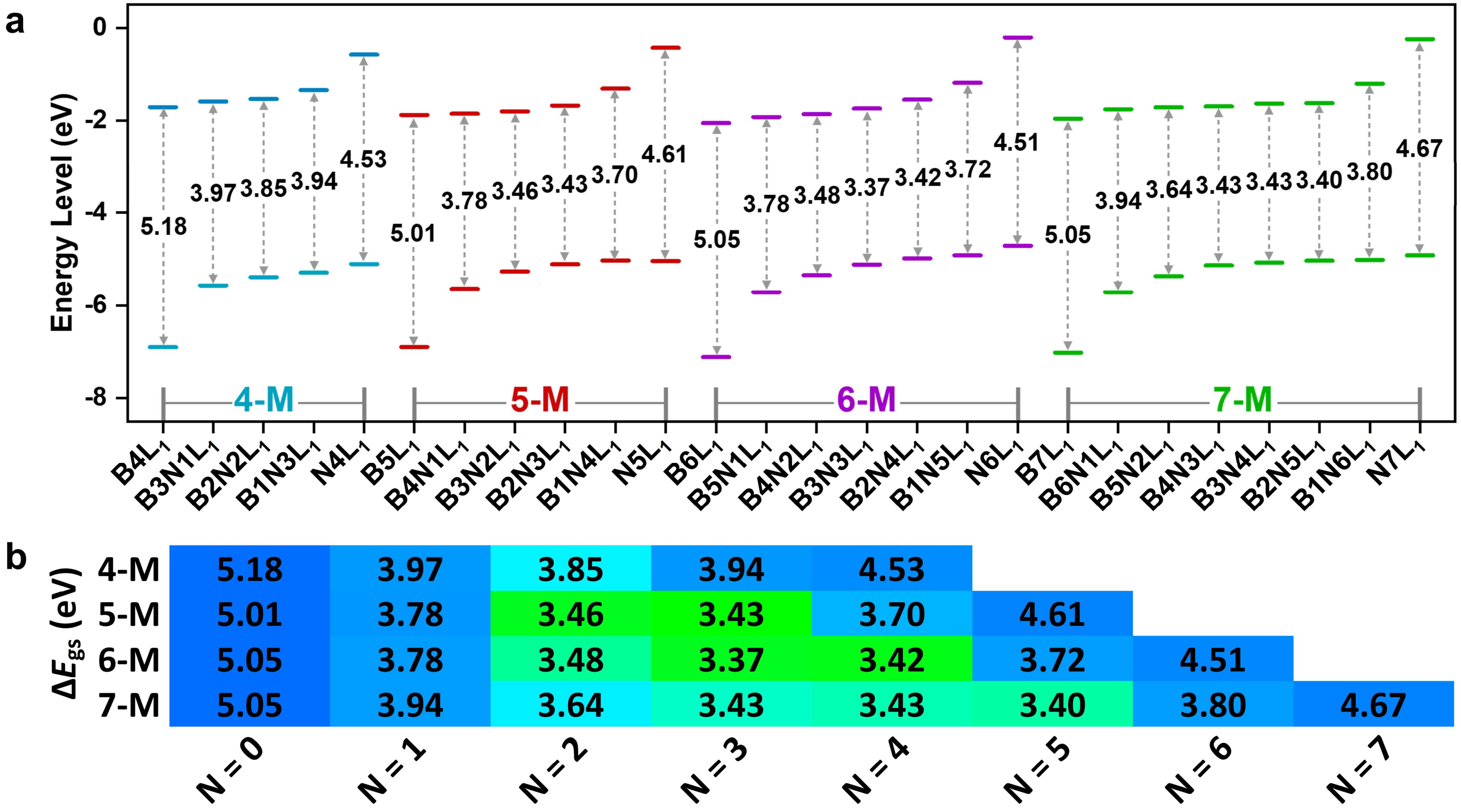

Figure S6. (a) HOMO and LUMO energy levels, as well as (b) their energy gap ($\Delta E_{\text{H-L, gs}}$) of **$L_1$-**based macrocycles at S_0-optimized structure.

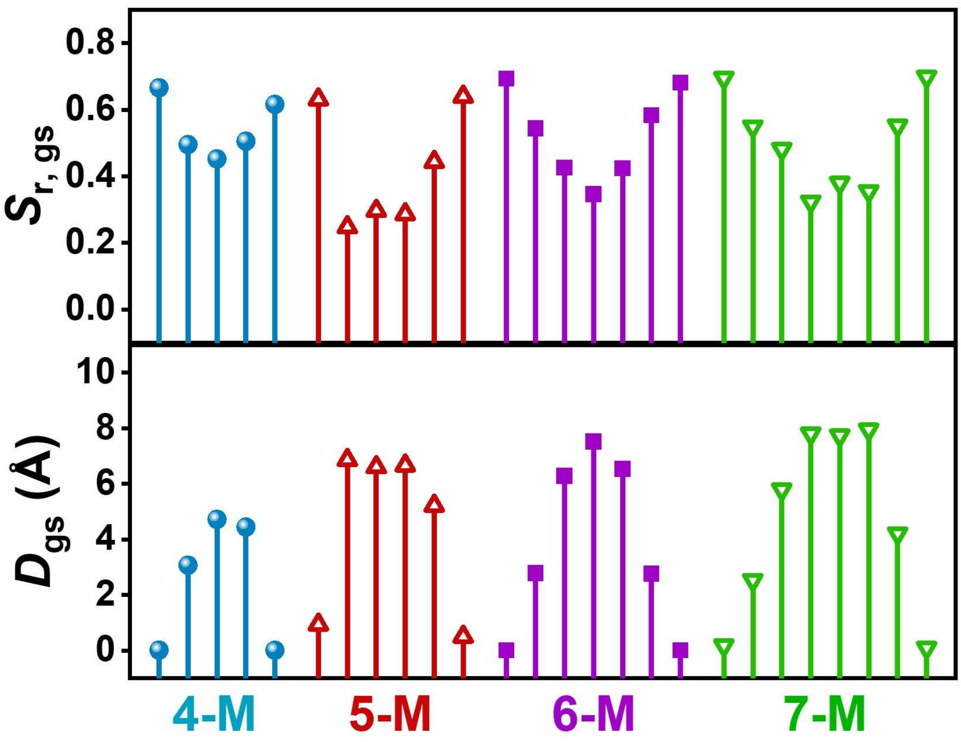

Figure S7. $S_{r,\ gs}$ and D_{gs} values of **L1**-based macrocycles at S_0-optimized structure. The order of molecules is consistent with those in **Figure S5a**.

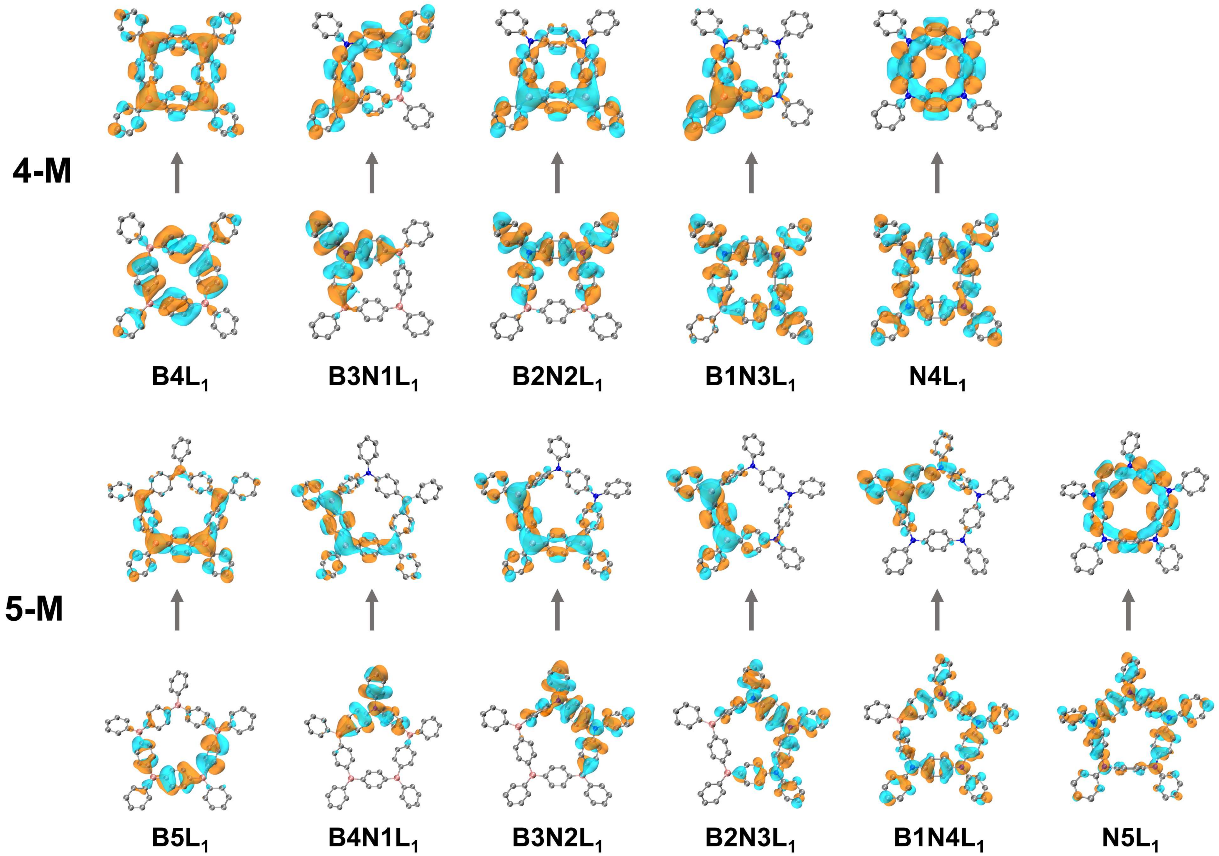

Figure S8. NTOs of **L_1**-based macrocycles at S_0-optimized structure.

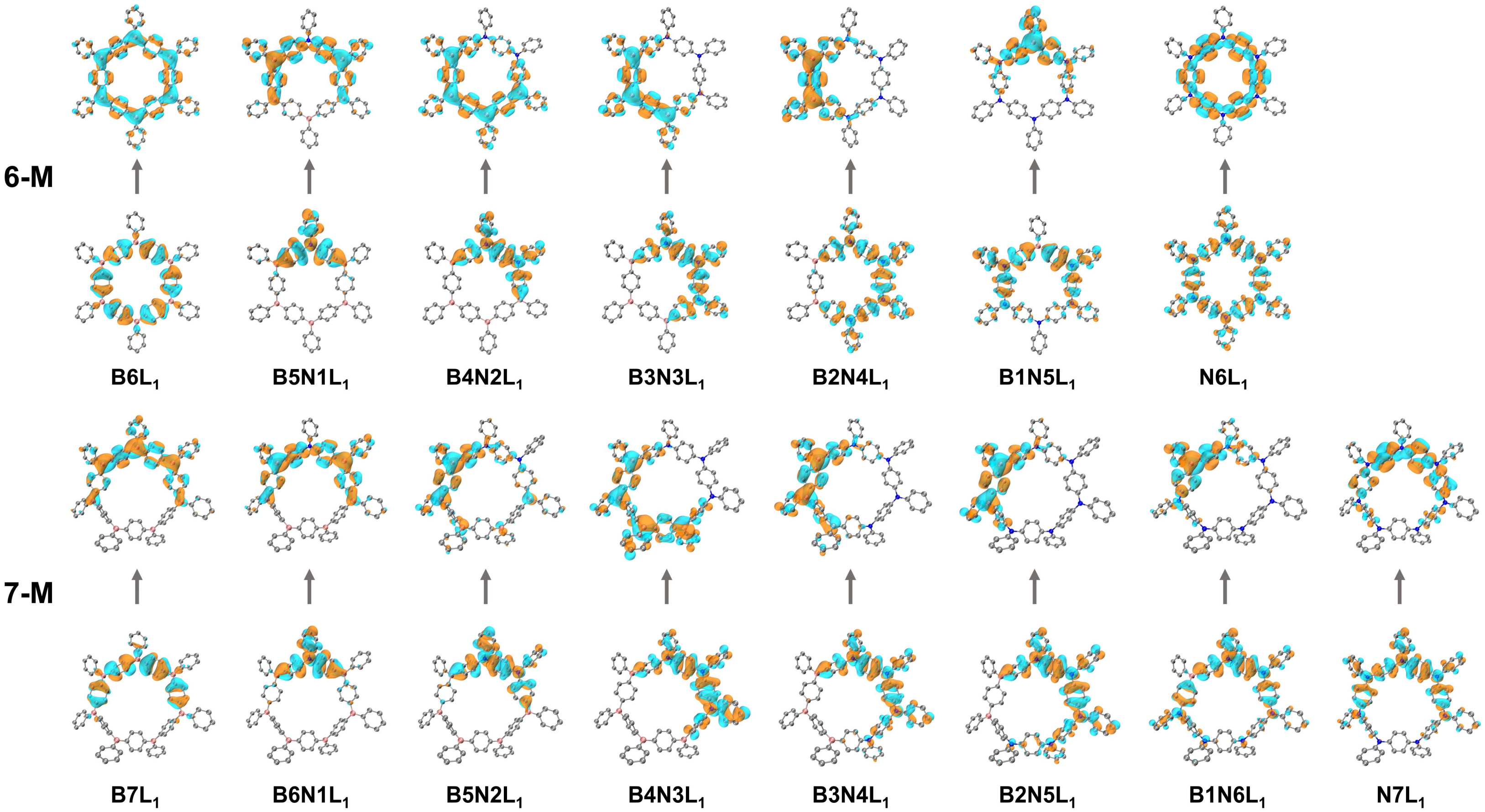

Figure S8. NTOs of $\mathbf{L_1}$**-**based macrocycles at S_0-optimized structure (continue).

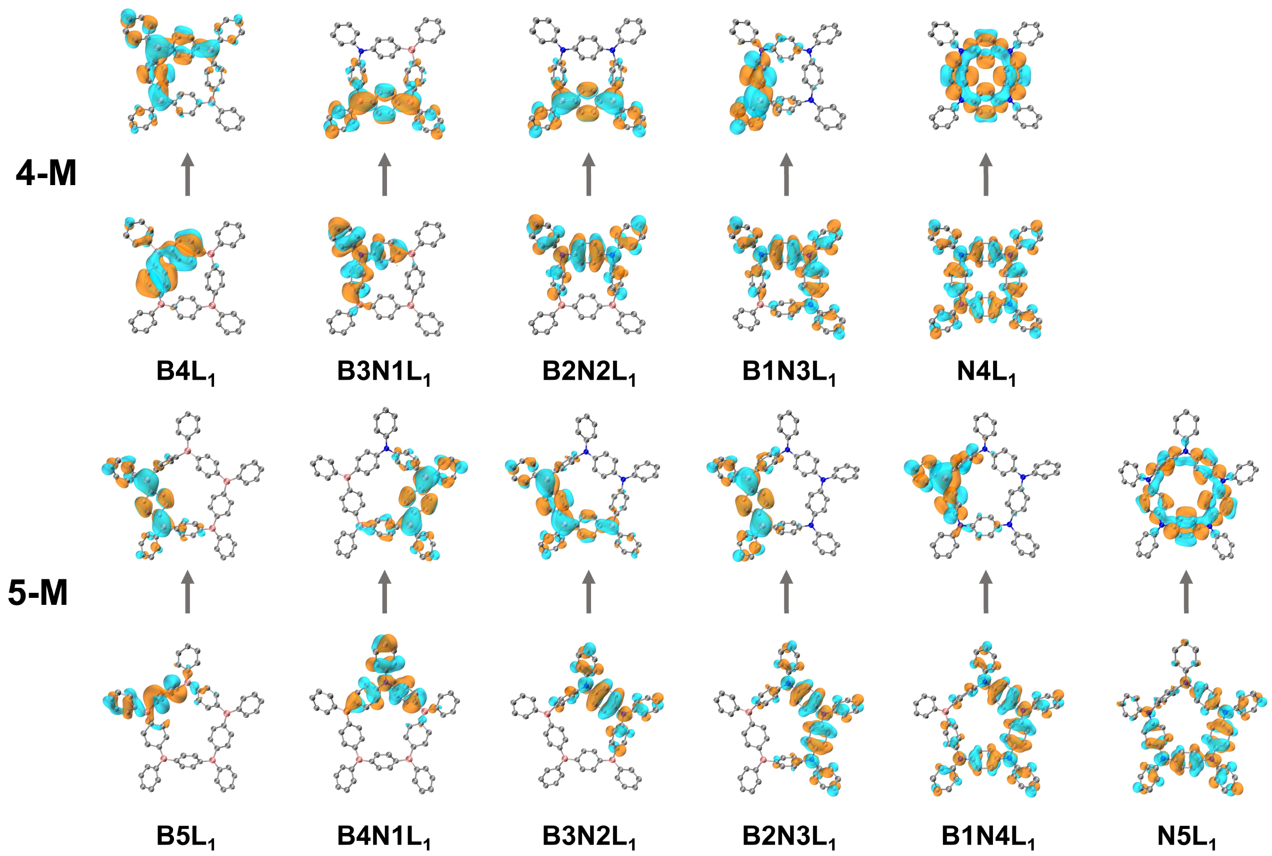

Figure S9. NTOs of $\mathbf{L_1}$-based macrocycles at S_1-optimized structure.

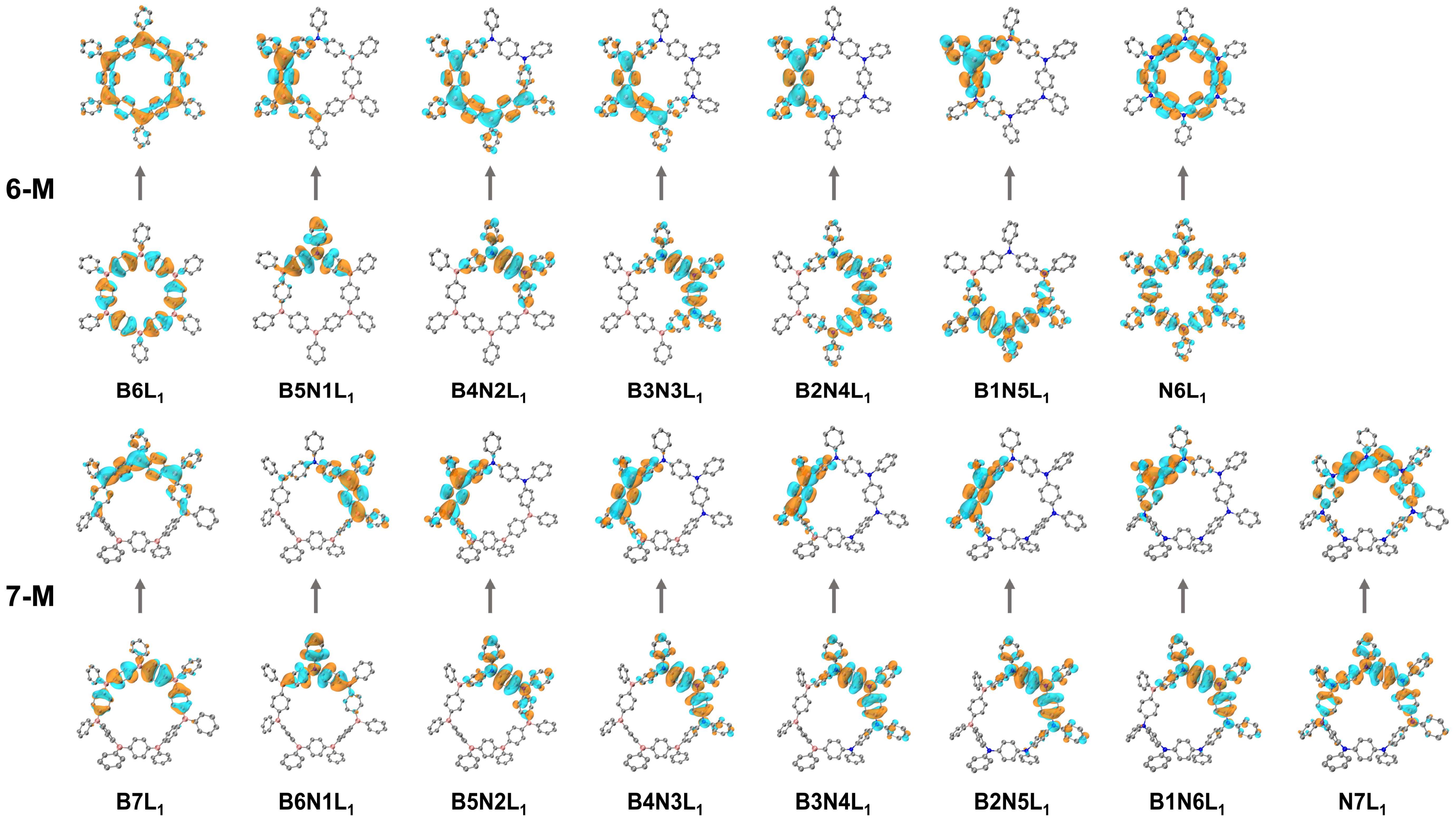

Figure S9. NTOs of **L_1-**based macrocycles at S_1-optimized structure (continue).

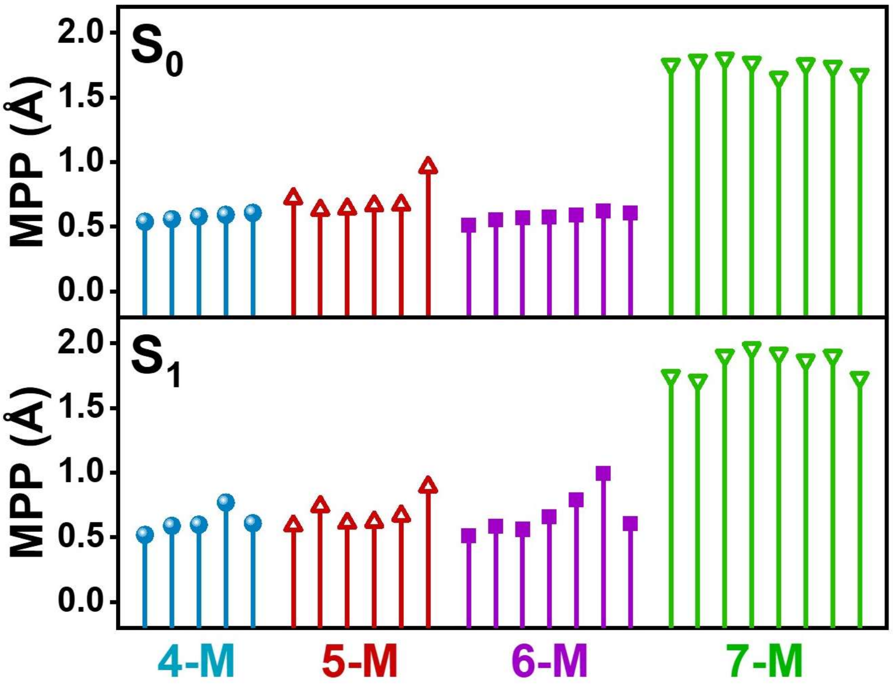

Figure S10. MPP values of **L_1-**based macrocycles at both S_0 and S_1-optimized structures. The order of molecules is consistent with those in **Figure S6a**

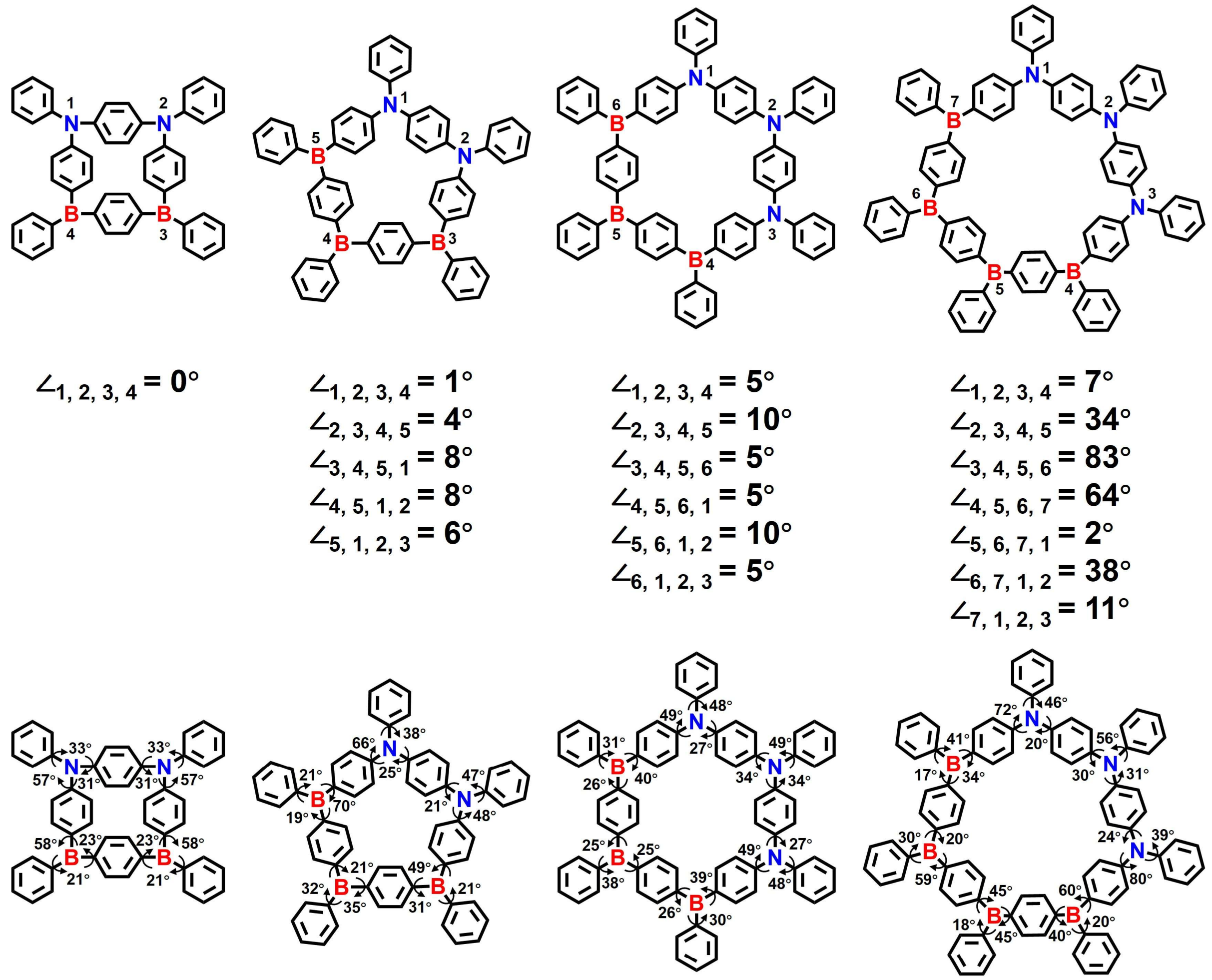

B2N2L$_1$ **B3N2L$_1$** **B3N3L$_1$** **B4N3L$_1$**

Figure S11. The geometric parameters for macrocycles: **B2N2L$_1$**, **B3N2L$_1$**, **B3N3L$_1$**, and **B4N3L$_1$** at S_1-optimized structure.

.

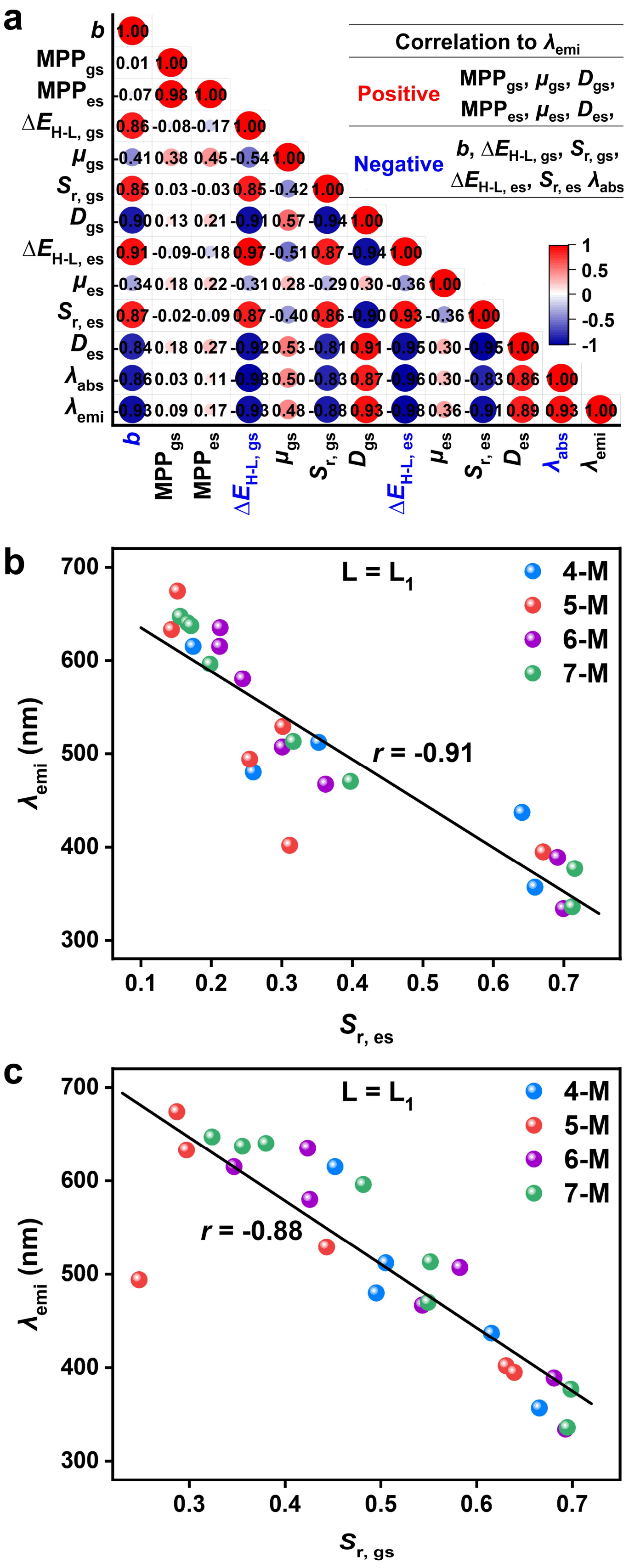

Figure S12. (a) Pearson's correlation coefficients matrix of the descriptors as well as λ_{abs} and λ_{emi} of the **L1**-based macrocycles. The fitted line and linear correlation between (b) λ_{emi} and $S_{r, es}$, (c) λ_{emi} and $S_{r, gs}$ of **L1**-based macrocycles.

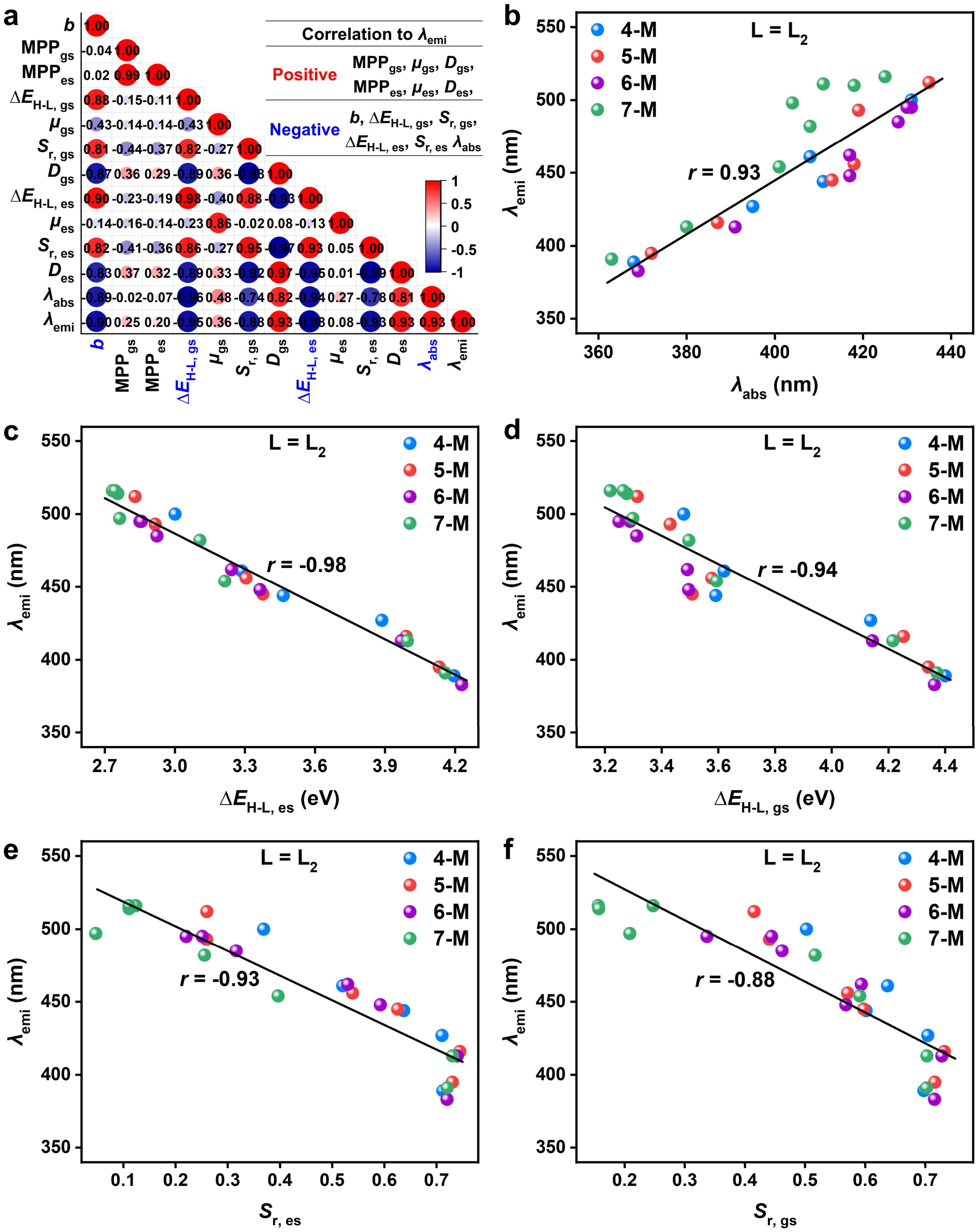

Figure S13. (a) Pearson's correlation coefficients matrix of the descriptors as well as λ_{abs} and λ_{emi} of **L2**-based macrocycles. The fitted line and linear correlation between (b) λ_{abs} and λ_{emi}, (c) λ_{emi} and $\Delta E_{H\text{-}L,\ es}$, (d) λ_{emi} and $\Delta E_{H\text{-}L,\ gs}$, (e) λ_{emi} and $S_{r,\ es}$, (f) λ_{emi} and $S_{r,\ gs}$ of **L2**-based macrocycles.

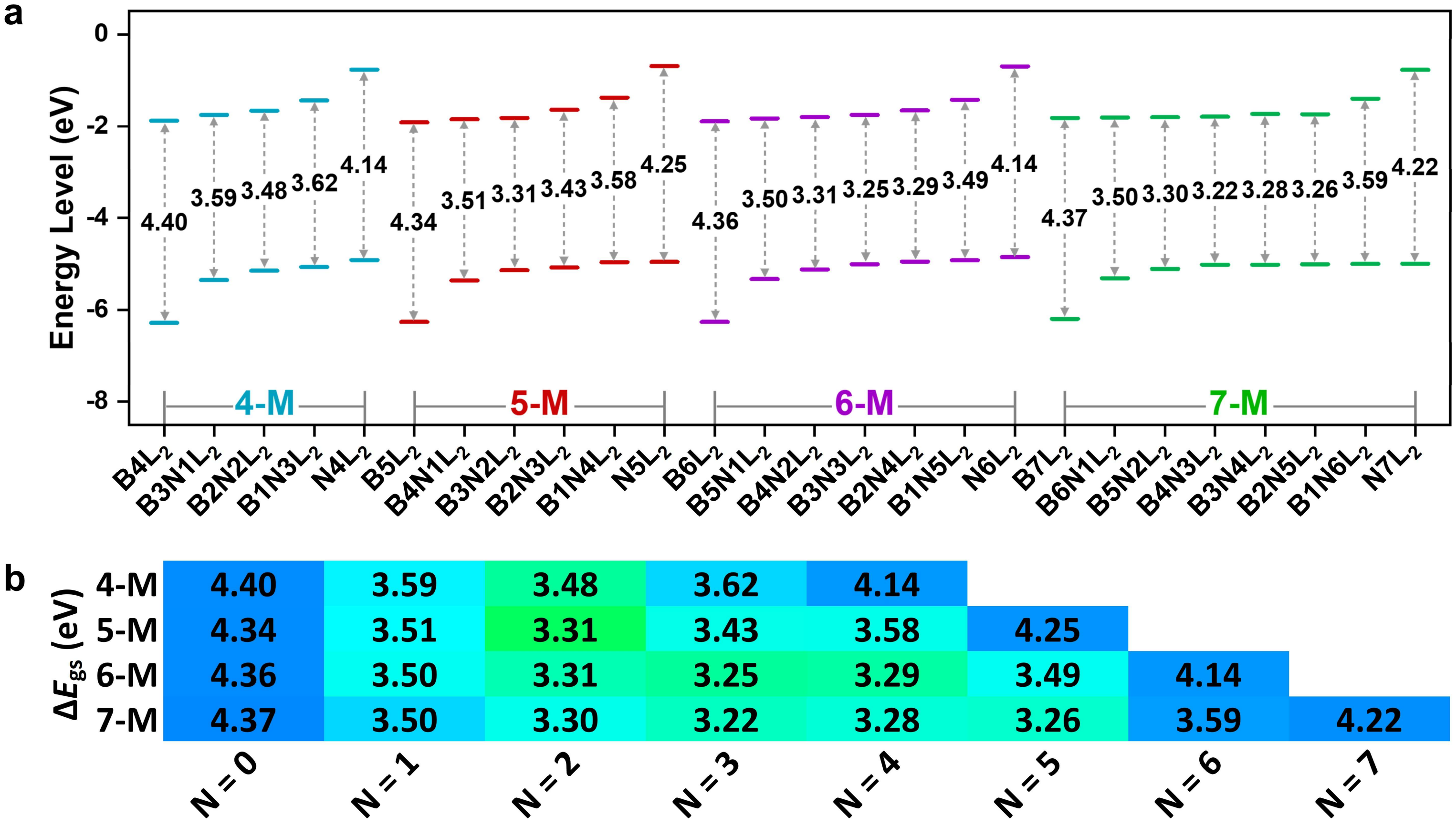

Figure S14. (a) HOMO and LUMO energy levels, as well as (b) their energy gap ($\Delta E_{\text{H-L, gs}}$) of $\mathbf{L_2}$-based macrocycles at S_0-optimized structure.

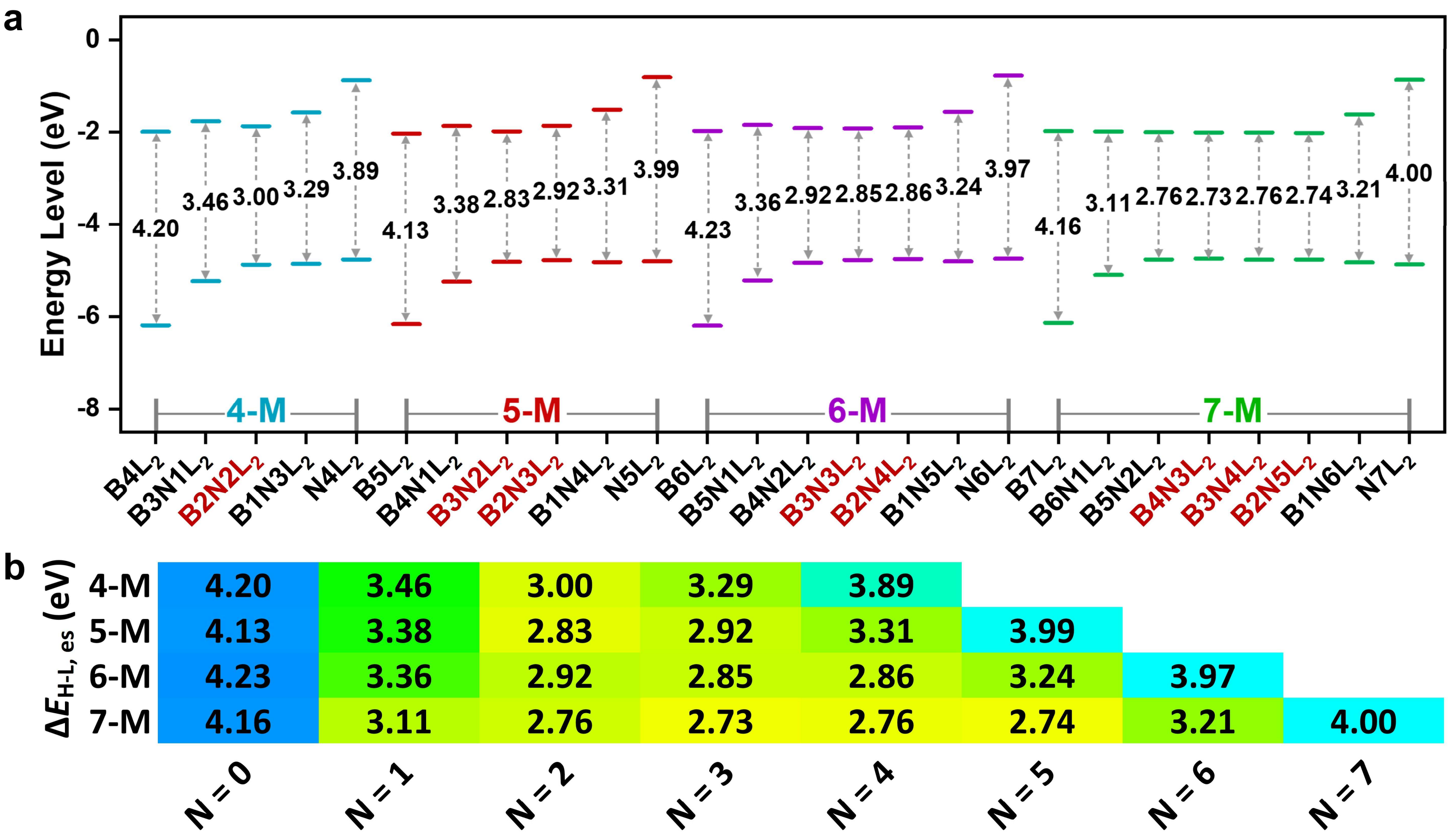

Figure S15. (a) HOMO and LUMO energy levels, as well as (b) their energy gap ($\Delta E_{\text{H-L, es}}$) of $\mathbf{L_2}$-based macrocycles at S_1-optimized structure.

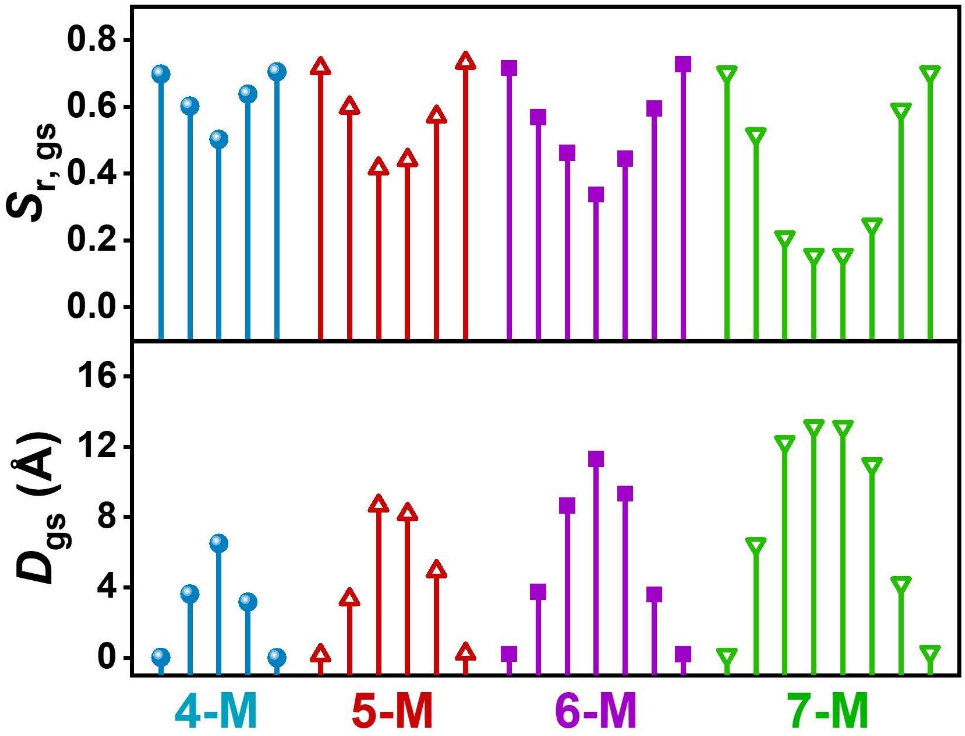

Figure S16. $S_{r,\ gs}$ and D_{gs} values of $\mathbf{L_2}$-based macrocycles at S_0-optimized structure. The order of molecules is consistent with those in **Figure S14a**.

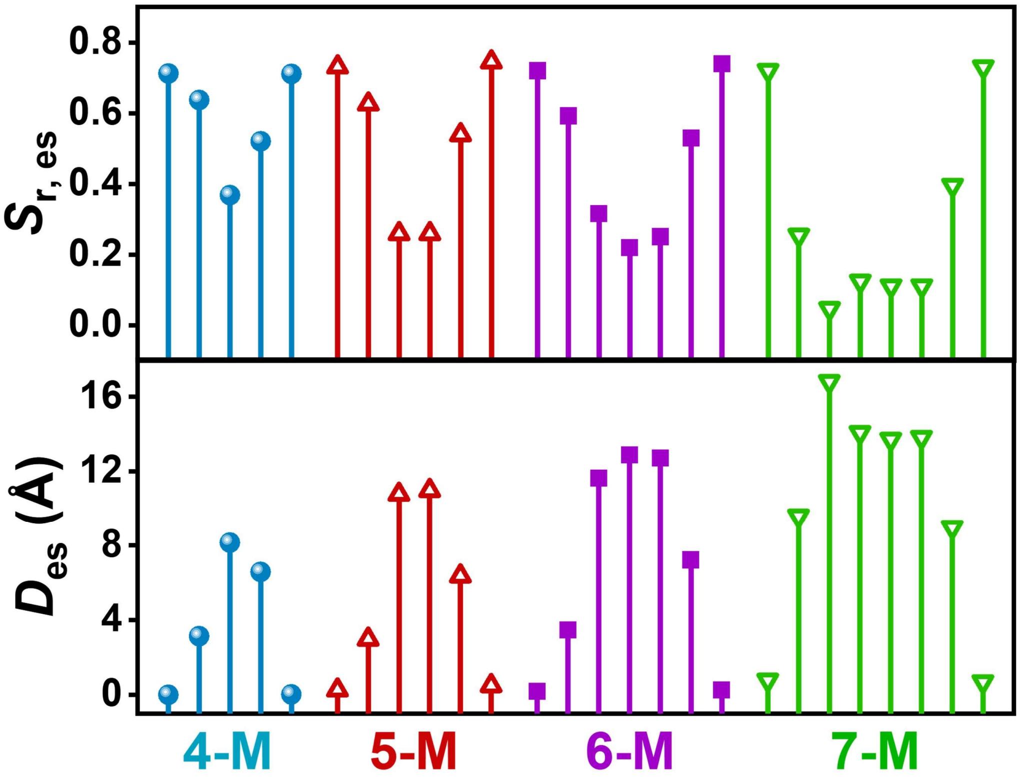

Figure S17. $S_{r,\ es}$ and D_{es} values of **L_2-**based macrocycles at S_1-optimized structure. The order of molecules is consistent with those in **Figure S14a**.

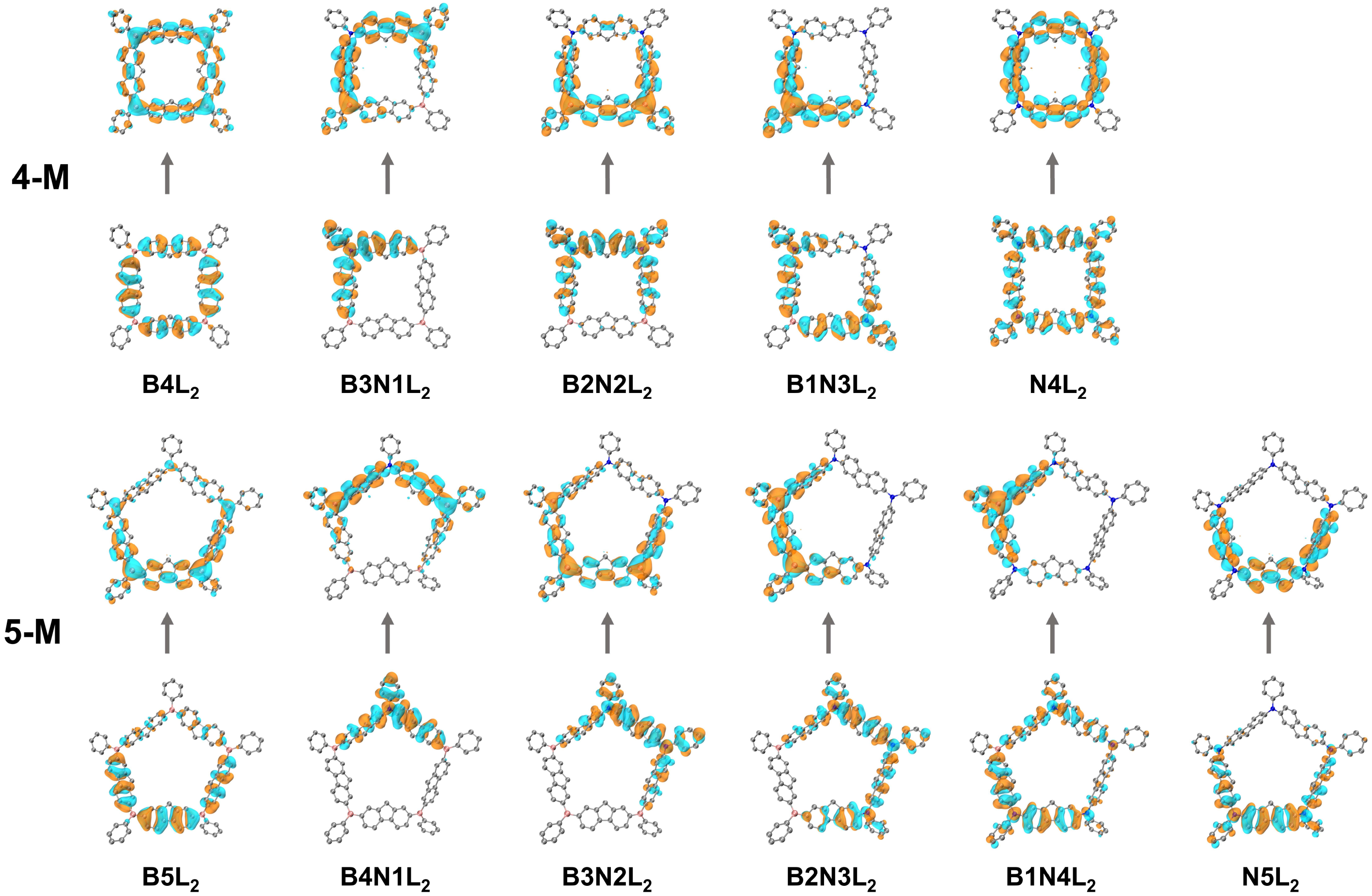

Figure S18. NTOs of $\mathbf{L_2}$-based macrocycles at S_0-optimized structure.

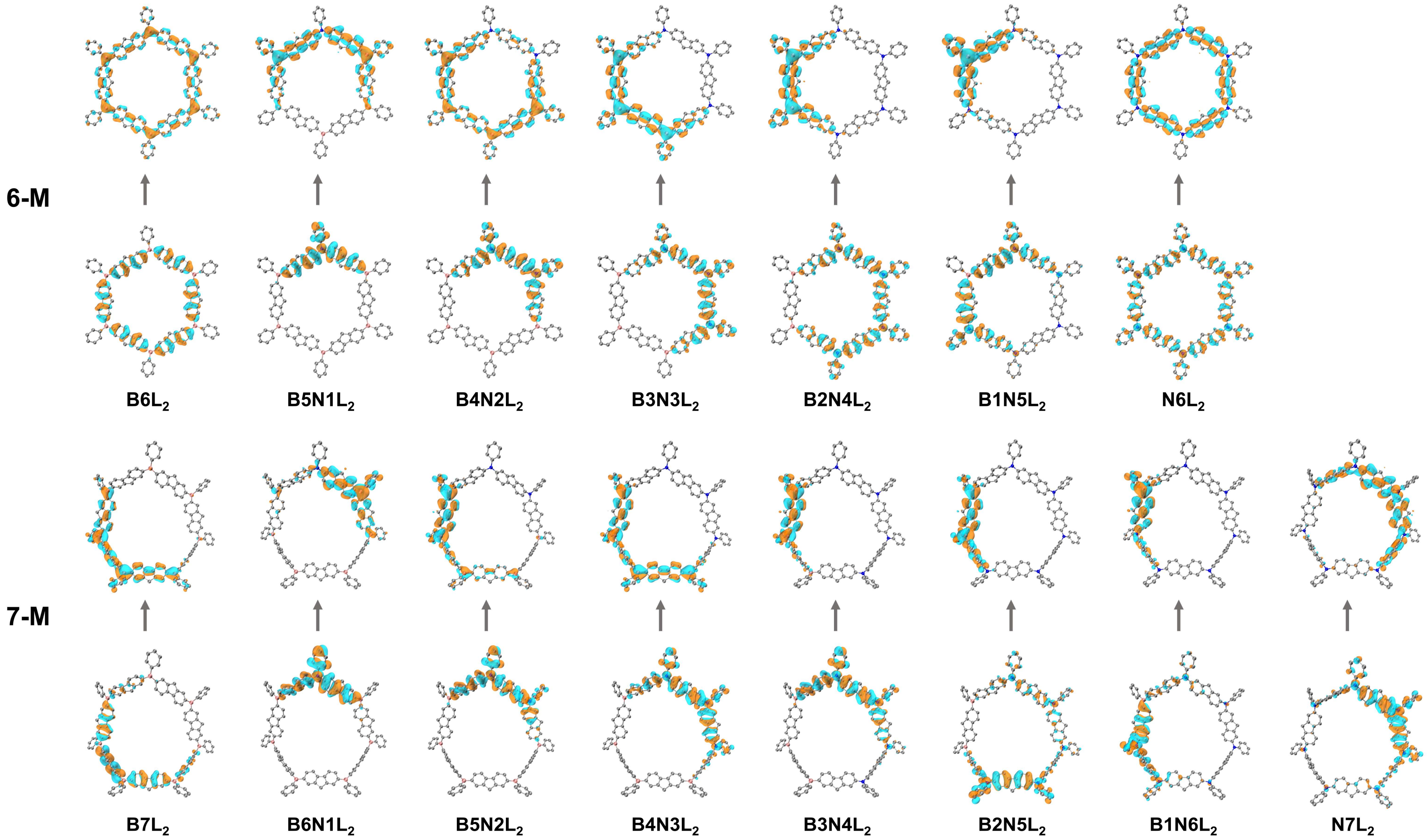

Figure S18. NTOs of $\mathbf{L_2}$-based macrocycles at S_0-optimized structure (continue).

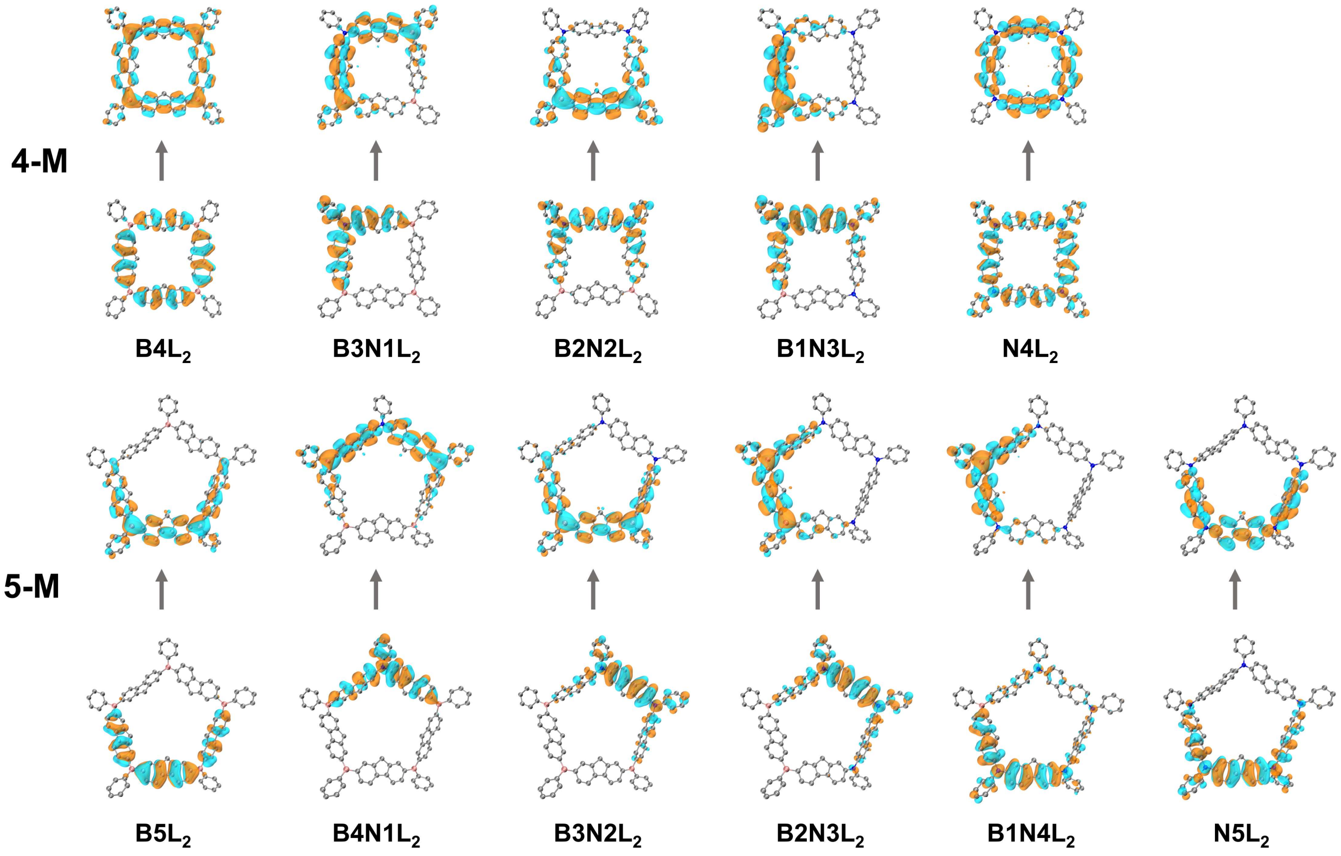

Figure S19. NTOs of $\mathbf{L_2}$**-**based macrocycles at S_1-optimized structure.

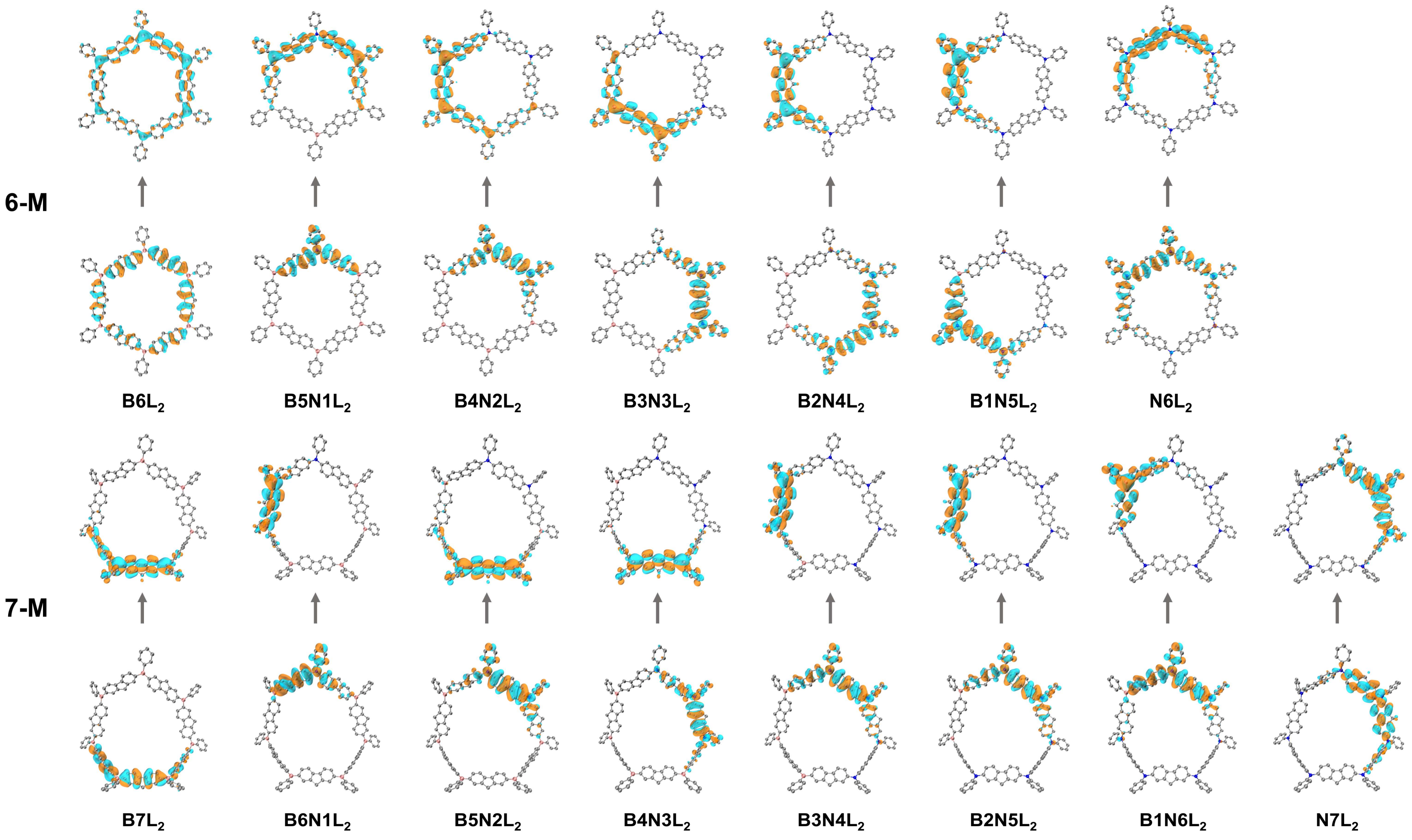

Figure S19. NTOs of **L**$_2$-based macrocycles at S_1-optimized structure (continue).

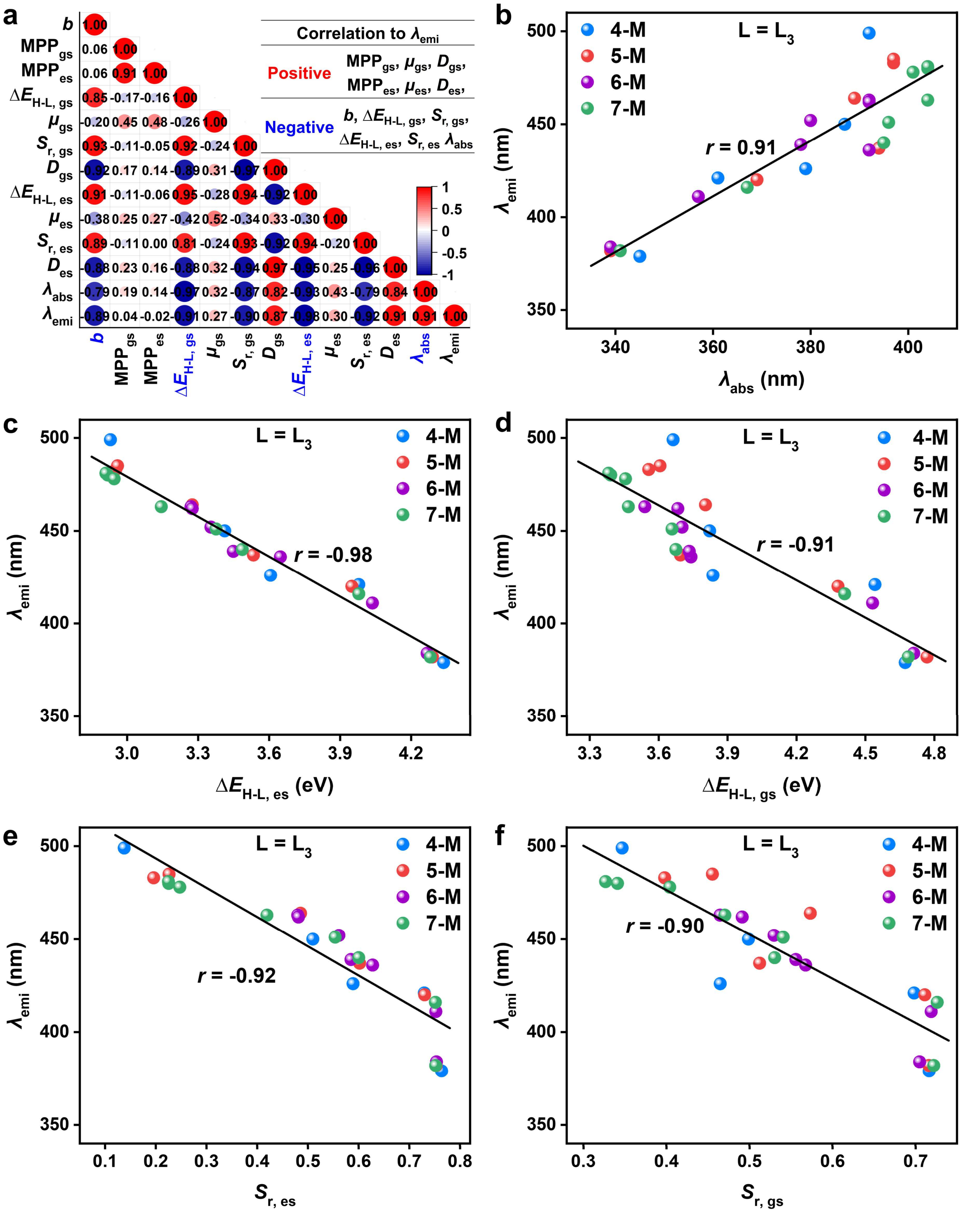

Figure S20. (a) Pearson's correlation coefficients matrix of the descriptors as well as λ_{abs} and λ_{emi} of **L3**-based macrocycles. The fitted line and linear correlation between (b) λ_{abs} and λ_{emi}, (c) λ_{emi} and $\Delta E_{H-L, es}$, (d) λ_{emi} and $\Delta E_{H-L, gs}$, (e) λ_{emi} and $S_{r, es}$, (f) λ_{emi} and $S_{r, gs}$ of **L3**-based macrocycles.

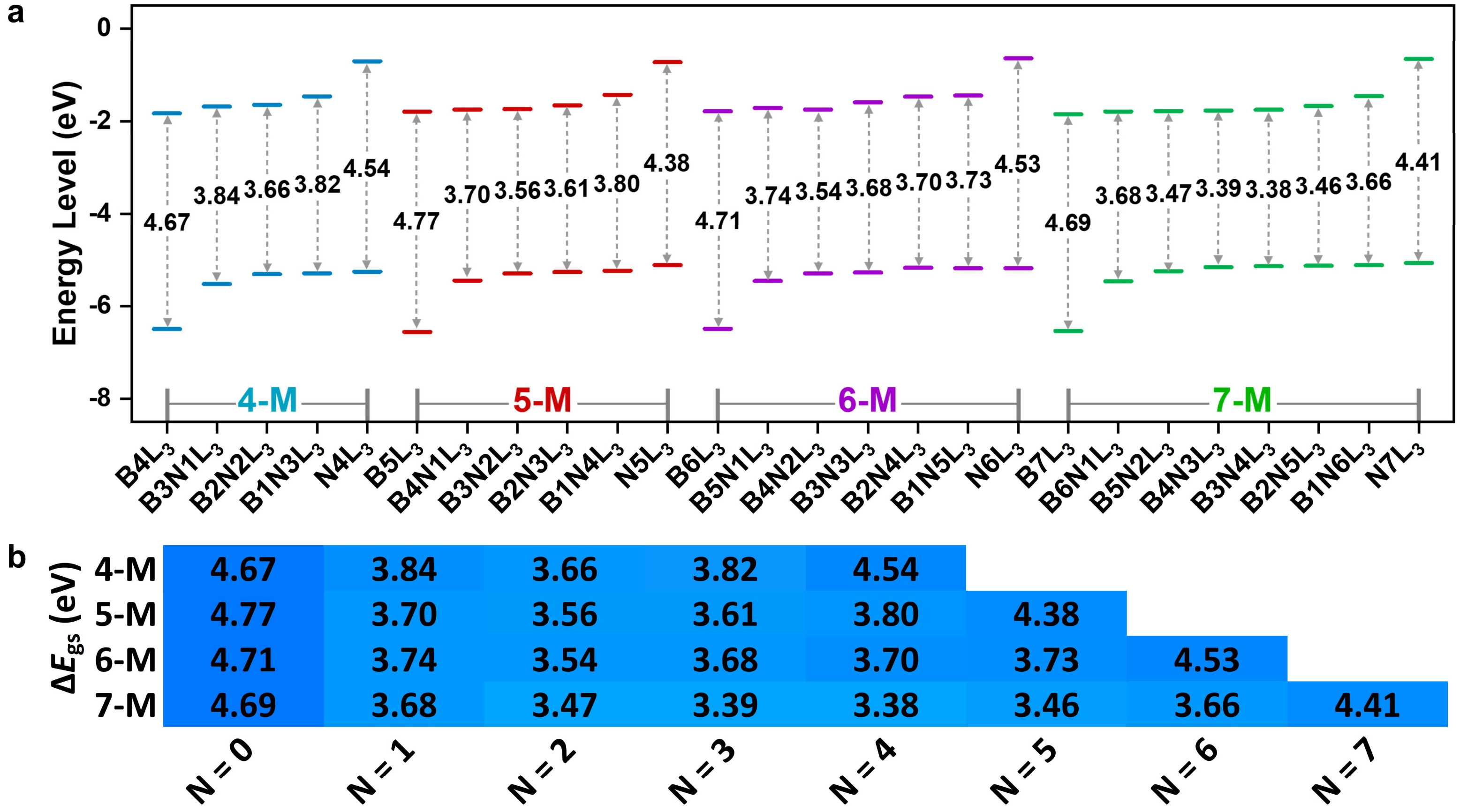

Figure S21. (a) HOMO and LUMO energy levels, as well as (b) their energy gap ($\Delta E_{\text{H-L, gs}}$) of **$L_3$-based** macrocycles at S_0-optimized structure.

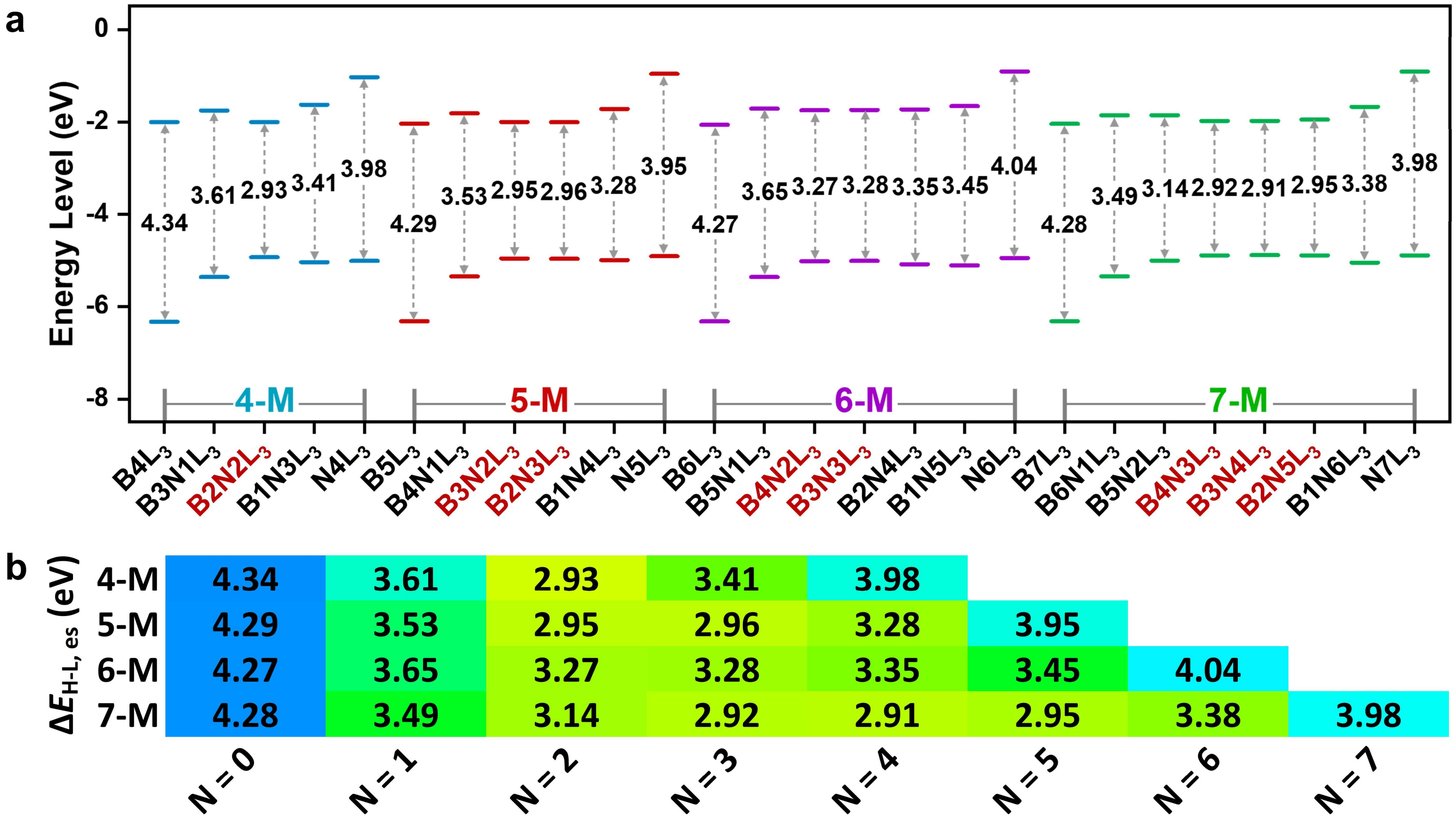

Figure S22. (a) HOMO and LUMO energy levels, as well as (b) their energy gap ($\Delta E_{\text{H-L, es}}$) of **$L_3$-based** macrocycles.

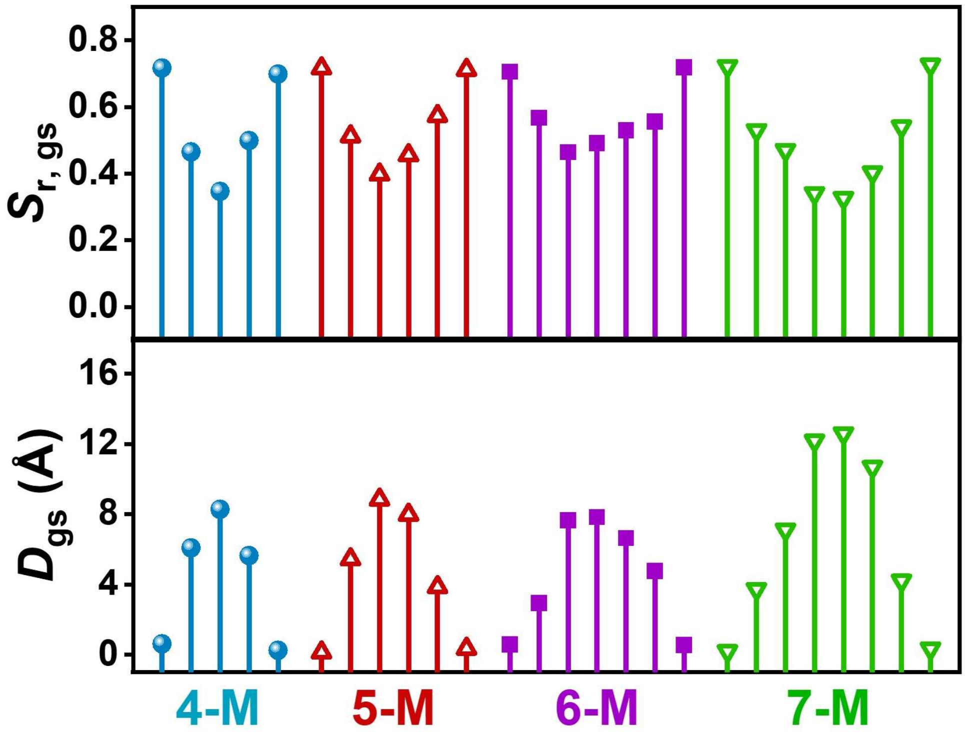

Figure S23. $S_{r,\, gs}$ and D_{gs} values of $\mathbf{L_3}$-based macrocycles at S_0-optimized structure. The order of molecules is consistent with those in **Figure S20a.**

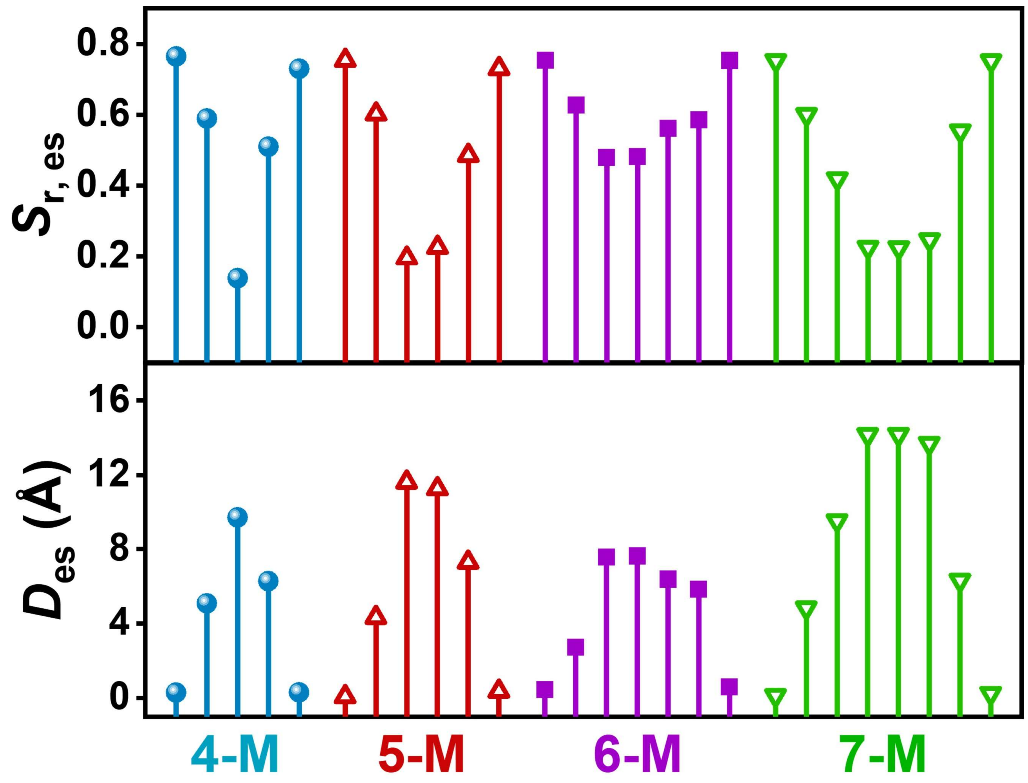

Figure S24. $S_{r,\ es}$ and D_{es} values of $\mathbf{L_3}$**-**based macrocycles at S_1-optimized structure. The order of molecules is consistent with those in **Figure S20a**.

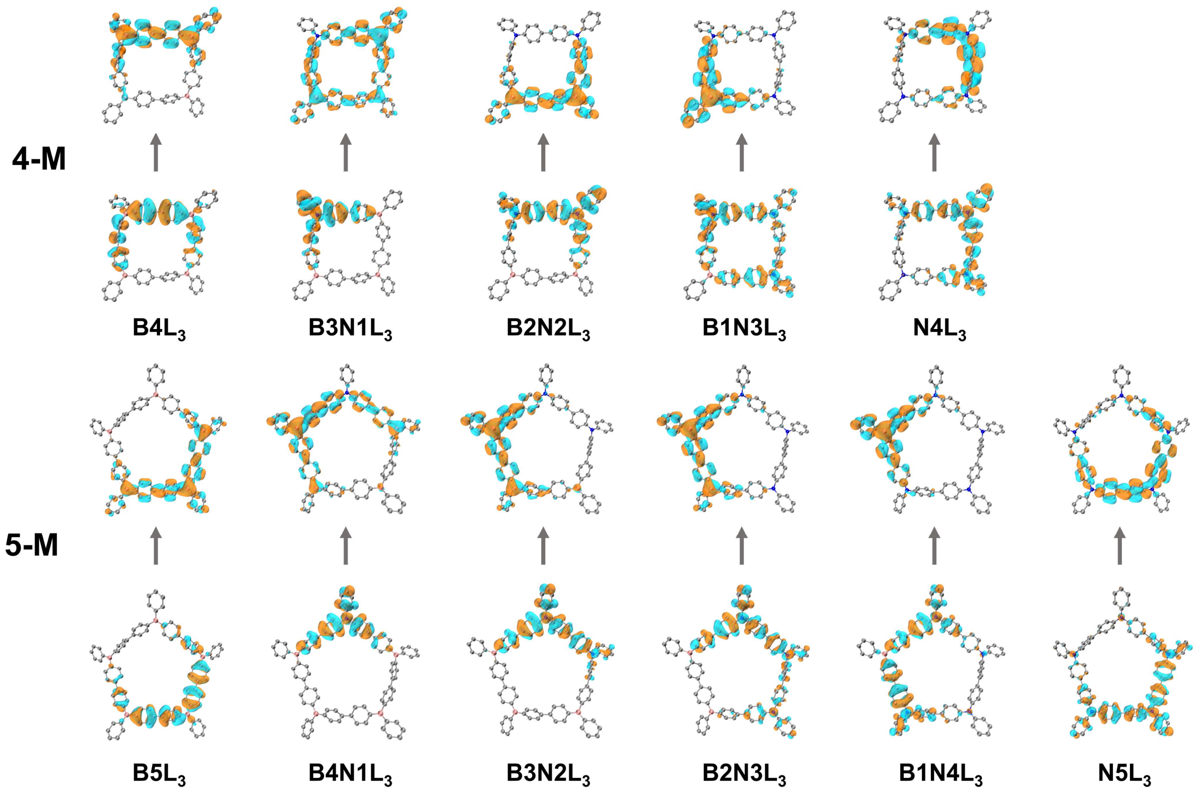

Figure S25. NTOs of $\mathbf{L_3}$-based macrocycles at S_0-optimized structure.

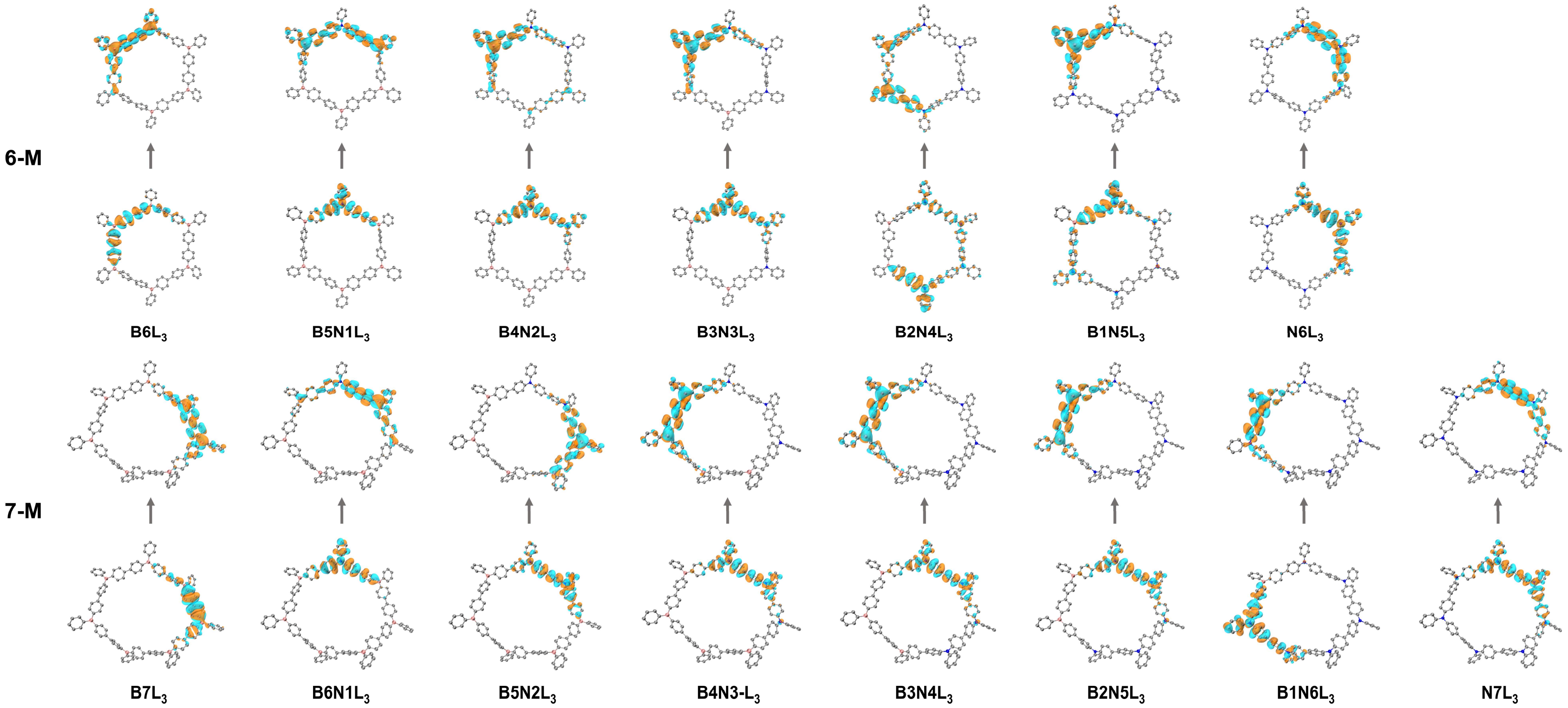

Figure S25. NTOs of **L_3-**based macrocycles at S_0-optimized structure (continue).

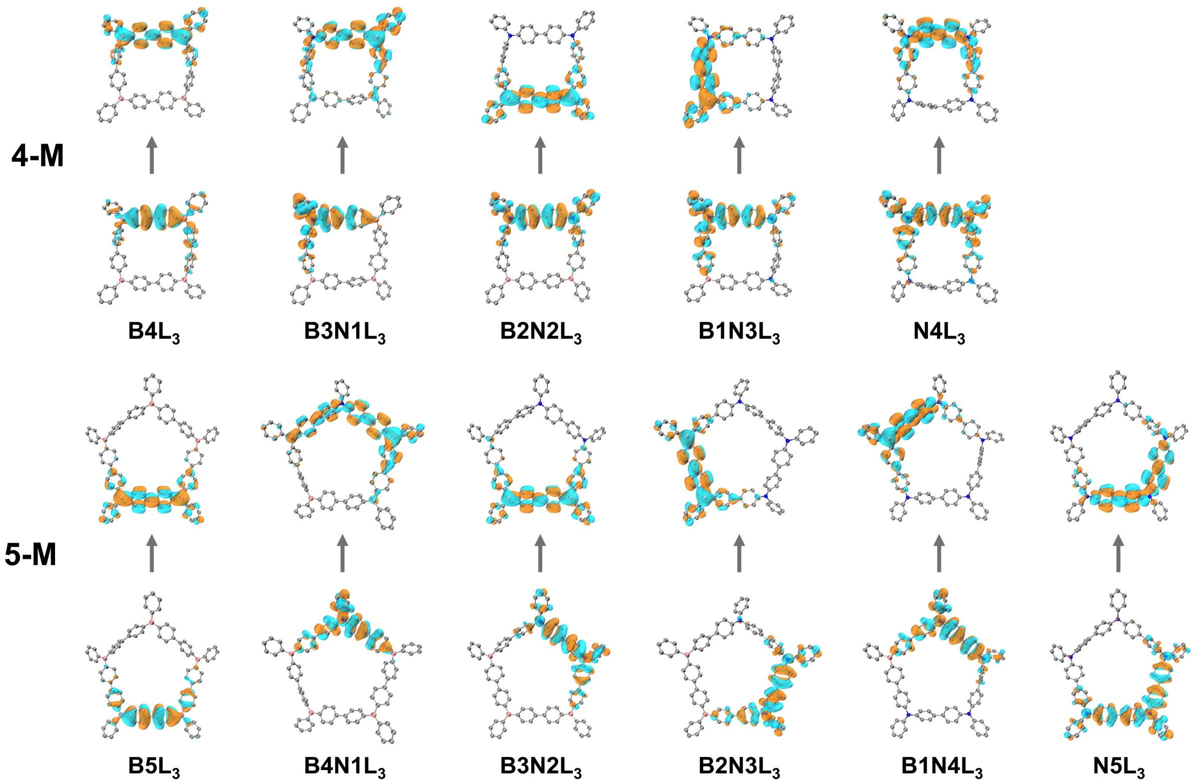

Figure S26. NTOs of $\mathbf{L_3}$-based macrocycles at S_1-optimized structure.

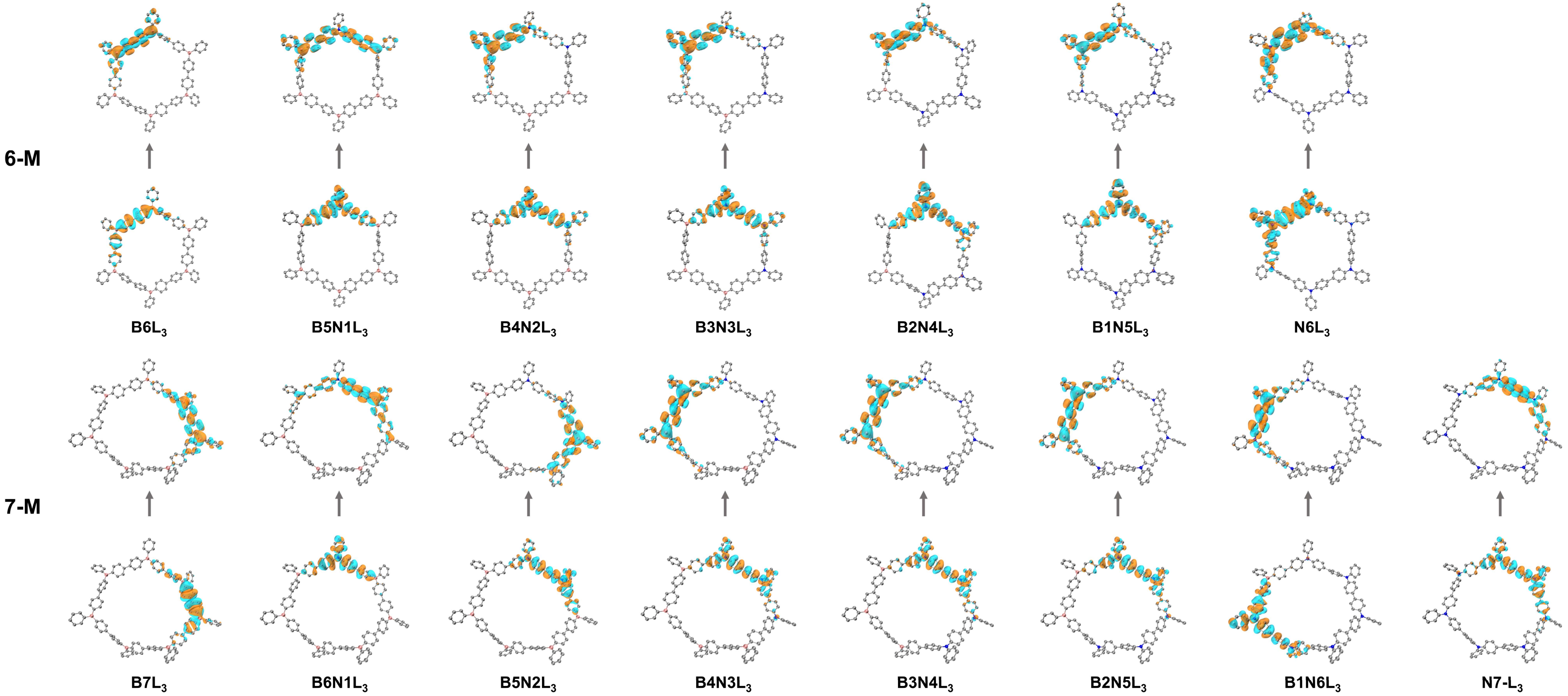

Figure S26. NTOs of **L_3-**based macrocycles at S_1-optimized structure (continue).

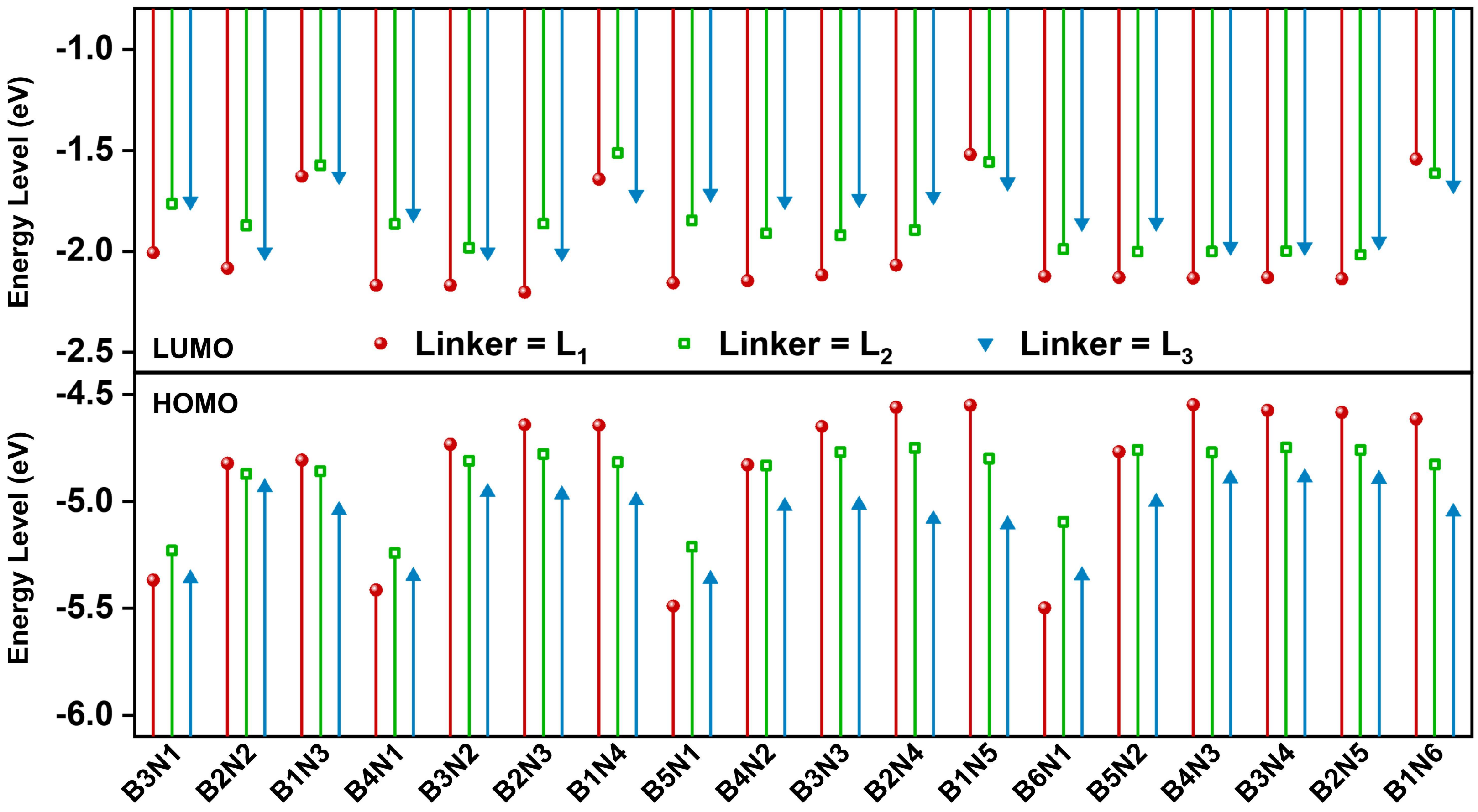

Figure S27. HOMO and LUMO energy levels of the B/N doped macrocycles with different π-linkers at S_1-optimized structure.

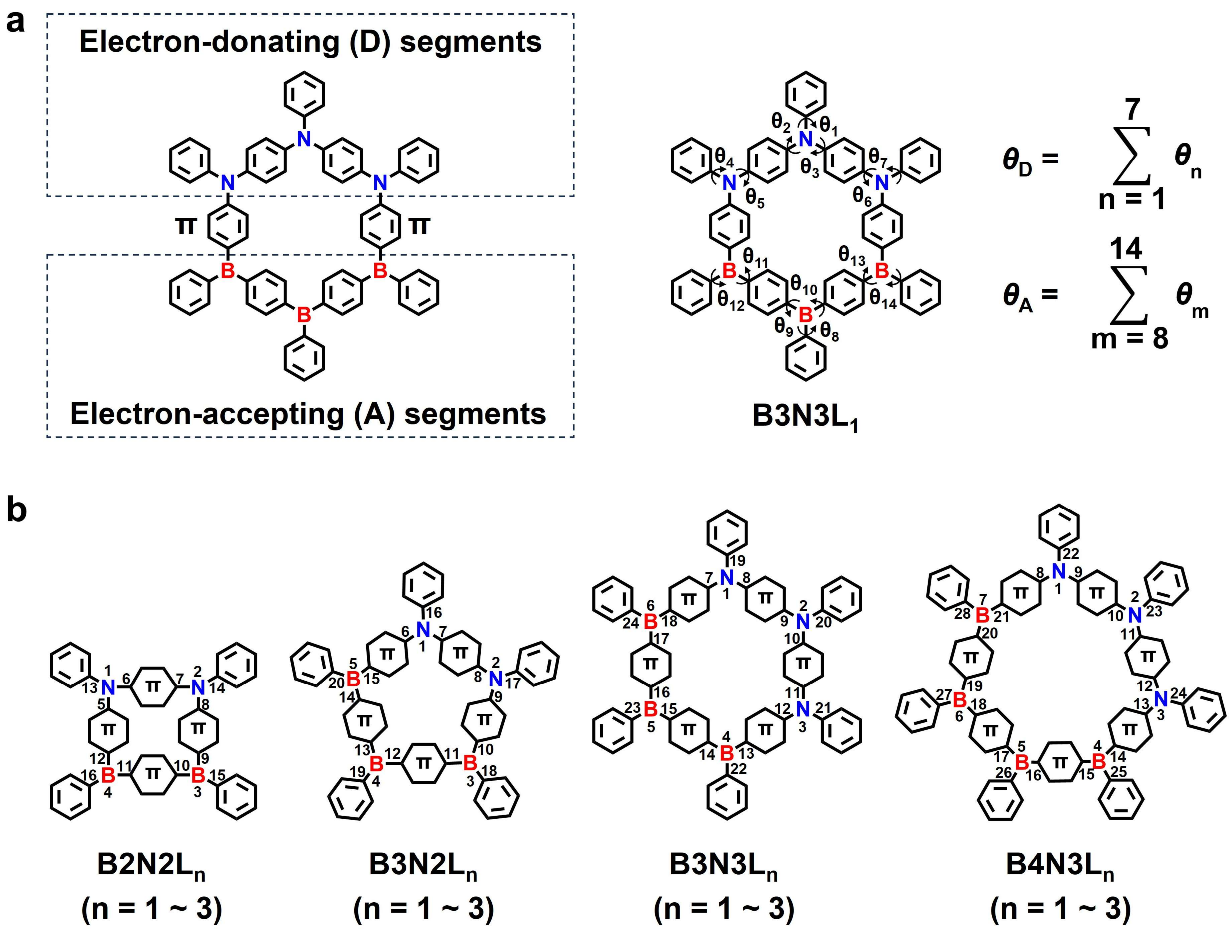

Figure S28. (a) The schematic diagram of D and A segments, as well as the define of θ_A and θ_D for the B/N-doped macrocycles we designed (taking **B3N3L$_1$** as an example). (b) The schematic diagram of atomic serial numbers for **B2N2L$_n$**, **B3N2L$_n$**, **B3N3L$_n$** and **B4N3L$_n$** (n = 1 ~ 3). The statistical results of the corresponding bond lengths are shown in **Table S6 ~ S9**.

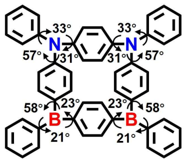

θ_D = 32°, θ_A = 22°
B2N2L$_1$

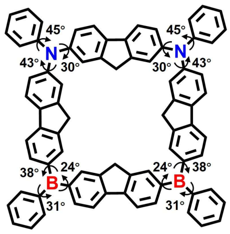

θ_D = 38°, θ_A = 28°
B2N2L$_2$

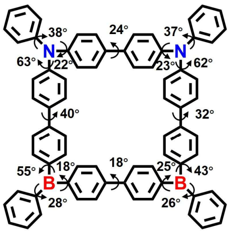

θ_D = 30°, θ_A = 24°
B2N2L$_3$

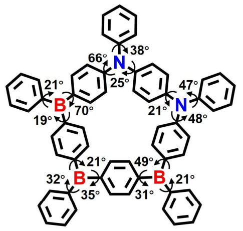

θ_D = 33°, θ_A = 26°
B3N2L$_1$

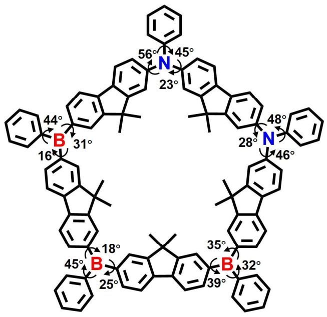

θ_D = 36°, θ_A = 31°
B3N2L$_2$

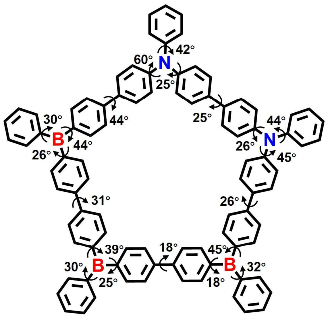

θ_D = 34°, θ_A = 28°
B3N2L$_3$

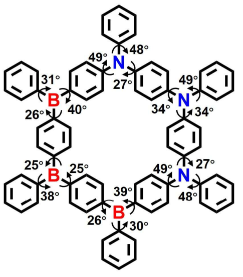

θ_D = 38°, θ_A = 29°
B3N3L$_1$

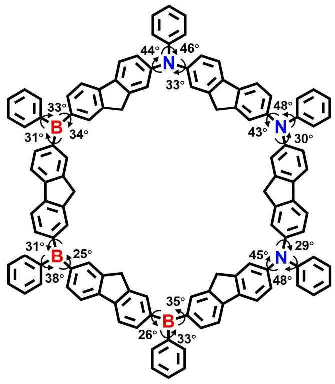

θ_D = 40°, θ_A = 31°
B3N3L$_2$

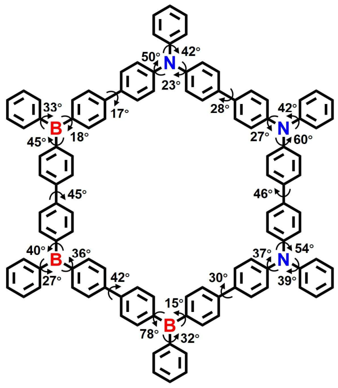

θ_D = 41°, θ_A = 42°
B3N3L$_3$

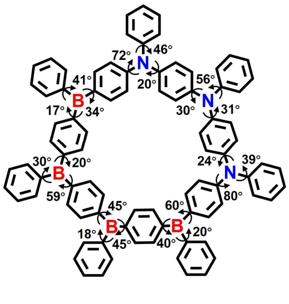

θ_D = 35°, θ_A = 34°
B4N3L$_1$

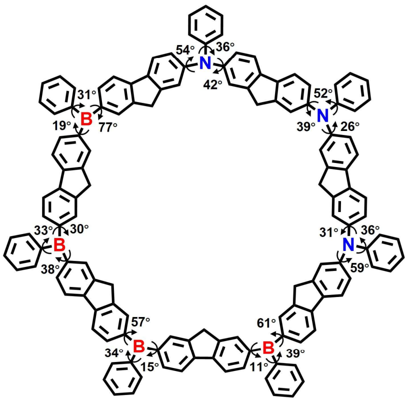

θ_D = 37°, θ_A = 31°
B4N3L$_2$

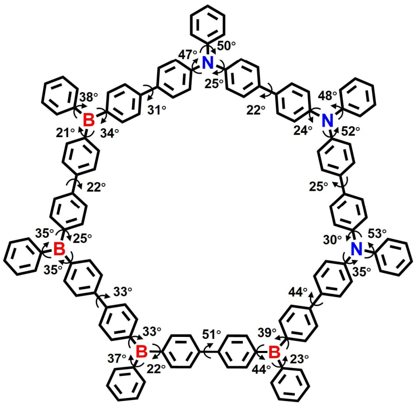

θ_D = 40°, θ_A = 31°
B4N3L$_3$

Figure S29. The rotational angles of π-linkers and exocyclic phenyls for macrocycles **B2N2L$_n$**, **B3N2L$_n$**, **B3N3L$_n$** and **B4N3L$_n$** (n = 1 ~ 3) with different π-linkers at S_1-optimized structure.

2. Supplementary Tables

Table S1. The calculated λ_{abs} and λ_{emi} of **MC-b-B3N3**, **B3N3L1**, and **B1N5L1** in toluene by functionals with different HF proportions combined with 6-31G(d) basis set.

		HF proportion	λ_{abs} (nm)	f	λ_{emi} (nm)	f
	exp.	---	408	---	520	---
	B3LYP	20%	475	0.2063	670	0.0689
	PW6B95	28%	445	0.2827	570	0.1253
**B1N5L1**	PBE0-1/3	33%	401	0.5384	507	0.1927
	PBE0-3/8	38%	385	0.5569	472	0.2736
	BMK	42%	395	0.6156	471	0.3831
	M06-2X	54%	365	0.5061	414	0.6088
	exp.	---	416	---	612	---
	B3LYP	20%	551	0.2624	847	0.1035
	PW6B95	28%	507	0.3516	712	0.1511
**B3N3L1**	PBE0-1/3	33%	440	0.4810	615	0.1464
	PBE0-3/8	38%	416	0.5852	559	0.1728
	BMK	42%	425	0.7003	555	0.2371
	M06-2X	54%	369	1.1667	447	0.3978
**MC-b-B3N3**	PBE0-1/3	33%	444	0.5302	639	0.1754

Table S2. The calculated λ_{abs} and λ_{emi} of **B1N5L$_1$** and **B3N3L$_1$** in toluene by 6-31G (d), 6-31G (d, p) and 6-311G (d, p) basis sets combined with PBE0-1/3 functional.

		λ_{abs} (nm)	f	λ_{emi} (nm)	f
**B1N5L$_1$**	6-31G (d)	401	0.5384	507	0.1927
	6-31G (d, p)	401	0.5369	508	0.1912
	6-311G (d, p)	401	0.5333	511	0.1923
**B3N3L$_1$**	6-31G (d)	440	0.4810	615	0.1464
	6-31G (d, p)	441	0.4810	615	0.1460
	6-311G (d, p)	437	0.5015	622	0.1352

Table S3. Transition properties of **L_1**-based macrocycles.

		Absorption				Emission			
		λ_{abs} (nm)	E_{vert} (eV)	assignment	*f*	λ_{emi} (nm)	E_{vert} (eV)	assignment	*f*
**4-M**	**B4L₁**	305	4.06	H→L 78.4%	0.9266	357	3.48	H→L 92.5%	0.3162
	**B3N1L₁**	395	3.14	H→L+1 93.8%	0.2991	480	2.58	H→L 95.8%	0.0457
	**B2N2L₁**	411	3.02	H→L 91.2%	0.0320	615	2.02	H→L 99.0%	0.0028
	**B1N3L₁**	393	3.15	H→L 94.0%	0.3887	512	2.42	H→L 95.5%	0.1154
	**N4L₁**	374	3.32	H→L 93.0%	0.0000	437	2.84	H→L 95.7%	0.0000
**5-M**	**B5L₁**	317	3.91	H→ L 39.5%<br>H-2→L 11.6%<br>H→L+2 10.7%<br>H-1→L 9.4%<br>H→L+1 9.1%	0.1031	402	3.08	H→L 88.1%<br>H→L+1 3.7%	0.0334
	**B4N1L₁**	393	3.16	H→L 93.8%	0.0594	494	2.51	H→L 94.3	0.0675
	**B3N2L₁**	440	2.82	H→L 94.2%	0.0589	633	1.96	H→L 99.1%	0.0059
	**B2N3L₁**	443	2.80	H→L 96.3%	0.1891	674	1.84	H→L 99.4%	0.0395
	**B1N4L₁**	412	3.01	H→L 87.1%	0.1512	529	2.35	H→ L 96.0%	0.0443
	**N5L₁**	356	3.48	H→L 81.1%<br>H→L+2 6.2%	0.0855	395	3.14	H→L 84.8%<br>H→L+1 6.0%	0.1892
**6-M**	**B6L₁**	320	3.88	H→L 63.7%<br>H-1→L+2 6.3%<br>H-2→L+1 6.3%	0.0000	334	3.71	H→L 68.9%<br>H-1→L+1 9.1%<br>H-2→L+2 9.1%	0.0000
	**B5N1L₁**	402	3.08	H→L+1 86.3%<br>H→L+3 9.4%	0.6720	467	2.65	H→L 85.4%<br>H→L+1 9.7%	0.2542
	**B4N2L₁**	433	2.86	H→L 86.7%<br>H→L+2 8.1%	0.1668	580	2.14	H→L 96.9%	0.0561
	**B3N3L₁**	440	2.82	H→L 95.1%	0.4810	615	2.02	H→L 99.0%	0.1464
	**B2N4L₁**	442	2.80	H→L 90.4%	0.0424	635	1.95	H→L 98.3%	0.0106
	**B1N5L₁**	401	3.09	H-1→L 85.6%	0.5384	507	2.45	H→L 93.7%	0.1927
	**N6L₁**	364	3.40	H→L 80.0%<br>H-1→L+2 7.3%<br>H-2→L+3 7.3%	0.0000	389	3.19	H→L 82.8%<br>H-2→L+2 6.9%<br>H-1→L+3 6.9%	0.0000
**7-M**	**B7L₁**	316	3.93	H-2→L 57.0%<br>H-3→L+1 12.9%<br>H→L 5.6%	0.4725	336	3.69	H→L 59.5%<br>H-2→L 15.8%<br>H-3→L+1 7.6%	0.9136
	**B6N1L₁**	401	3.09	H→L+1 83.9%<br>H→L+3 9.8%	0.8451	470	2.64	H→L 93.3%	0.3882
	**B5N2L₁**	410	3.03	H→L 65.7%<br>H→L+2 12.5%<br>H→L+1 11.2%	0.4592	596	2.08	H→L 96.3%	0.0921
	**B4N3L₁**	426	2.91	H→ L 89.3%	0.4218	647	1.92	H→L 97.5%	0.0609
	**B3N4L₁**	428	2.90	H→L 85.3%	0.1893	640	1.94	H→L 97.5%	0.0612
	**B2N5L₁**	430	2.89	H→L 87.0%	0.3575	637	1.95	H→L 97.5%	0.0764
	**B1N6L₁**	401	3.09	H→L 67.6%<br>H-2→L 23.0%	0.6207	513	2.42	H→L 93.6%	0.2069
	**N7L₁**	351	3.53	H→L 49.7%<br>H-1→L+1 25.4%<br>H→L+2 7.6%<br>H→L+3 5.1%	0.1034	377	3.29	H→L 70.2%<br>H→L+2 11.6%<br>H-1→L+1 8.8%	0.4990

Table S4. Transition properties of **L₂**-based macrocycles.

		Absorption				Emission			
		λ_{abs} (nm)	E_{vert} (eV)	assignment	*f*	λ_{emi} (nm)	E_{vert} (eV)	assignment	*f*
4-M	**B4L$_2$**	368	3.37	H→L 72.9%<br>H-1→L+2 11.6%<br>H-2→L+1 11.6%	0.0000	389	3.19	H→L 76.2%<br>H-1→L+2 10.5%<br>H-2→L+1 10.5%	0.0000
	**B3N1L$_2$**	411	3.02	H→L+1 84.5%<br>H→L+3 6.4%	0.4523	444	2.79	H→L+1 87.4%<br>H→L+3 5.6%	0.6337
	**B2N2L$_2$**	431	2.88	H→L 84.6%<br>H→L+2 5.8%	0.0110	500	2.48	H→L 93.8%	0.0077
	**B1N3L$_2$**	408	3.04	H-1→L 73.1%<br>H→L+1 17.4%	0.1728	461	2.69	H→L 79.5%<br>H-1→L 8.8%<br>H→L+1 5.4%	0.5091
	**N4L$_2$**	395	3.14	H→L 79.8%<br>H-1→L+2 7.8%<br>H-2→L+1 7.8%	0.0000	427	2.90	H→L 82.9%<br>H-1→L+2 6.2%<br>H-2→L+1 6.2%	0.0000
5-M	**B5L$_2$**	372	3.33	H→L 63.8%<br>H-2→L+1 14.8%<br>H-1→L+2 14.2%	0.1068	395	3.14	H→L 75.7%<br>H-1→L+2 9.4%<br>H-2→L+1 6.1%	0.6655
	**B4N1L$_2$**	413	3.01	H→L+1 77.1%<br>H→L+3 11.5%	0.7067	445	2.78	H→L+1 80.7%<br>H→L+3 10.4%	0.9488
	**B3N2L$_2$**	435	2.85	H→L 83.7%<br>H→L+2 6.0%	0.0657	511	2.42	H→L 95.1%	0.0538
	**B2N3L$_2$**	419	2.96	H→L 81.5%<br>H-1→L+1 8.2%	1.0358	493	2.51	H→L 95.1%	0.2788
	**B1N4L$_2$**	418	2.97	H→L 69.1%<br>H-2→L 13.9%	0.2883	456	2.72	H→L 79.9%<br>H-2→L 8.9%	0.3884
	**N5L$_2$**	387	3.20	H-1→L 61.7%<br>H→L+1 25.0%	0.1177	416	2.98	H→L 80.3%<br>H-1→L+1 9.6%	0.6496
6-M	**B6L$_2$**	369	3.36	H→L 51.2%<br>H-2→L+1 19.5%<br>H-1→L+2 19.4%	0.0000	383	3.24	H→L 53.6%<br>H-2→L+2 13.1%<br>H-1→L+1 13.1%	0.0333
	**B5N1L$_2$**	417	2.97	H→L+1 69.8%<br>H→L+3 19.3%	0.9136	448	2.77	H→L+1 72.6%<br>H→L+3 17.6%	1.1162
	**B4N2L$_2$**	428	2.90	H→L 69.4%<br>H→L+2 15.3%<br>H-1→L+1 6.9%	0.2859	485	2.55	H→L 87.7%<br>H→L+2 6.0%	0.2097
	**B3N3L$_2$**	431	2.88	H→L 84.5%<br>H-1→L+1 7.7%	0.7574	495	2.50	H→L 93.5%	0.2289
	**B2N4L$_2$**	430	2.88	H→L 74.7%<br>H-2→L 9.8%<br>H-1→L+1 7.4%	0.0894	495	2.51	H→L 93.3%	0.0942
	**B1N5L$_2$**	417	2.98	H-1→L 69.6%<br>H-3→L 13.9%<br>H-2→L+3 7.4%	0.6539	462	2.68	H→L 65.3%<br>H-1→L 17.9%<br>H-3→L 5.7%	0.7748
	**N6L$_2$**	391	3.17	H→L 57.2%<br>H-1→L+1 14.2%<br>H-2→L+2 14.1%	0.0000	413	3.00	H→L 65.5%<br>H-2→L+2 8.6%<br>H-1→L+1 6.6%	0.5011
7-M	**B7L$_2$**	363	3.41	H-1→L 18.5%<br>H-2→L+1 14.4%<br>H→L 14.3%<br>H-2→L 10.2%<br>H-1→L+2 6.0%	0.1128	391	3.17	H→L 73.4%	1.5839
	**B6N1L$_2$**	408	3.04	H→L+1 38.9%<br>H→L+2 35.1%<br>H→L+3 12.3%	0.7993	482	2.57	H→L 89.1%	0.1582
	**B5N2L$_2$**	418	2.97	H→L 73.5%<br>H→L+1 12.7%	0.0651	497	2.50	H→L 99.2%	0.0057
	**B4N3L$_2$**	427	2.90	H→L 90.7%	0.1045	516	2.40	H→L 96.8%	0.0488
	**B3N4L$_2$**	422	2.94	H→L 86.1%<br>H-1→L 5.4%	0.0227	514	2.41	H→L 96.1%	0.0348
	**B2N5L$_2$**	425	2.92	H→L 65.4%<br>H-1→L 21.7%	0.2098	516	2.40	H→L 96.4%	0.0350
	**B1N6L$_2$**	401	3.09	H-2→L 31.9%<br>H→L 22.4%<br>H-3→L 21.0%<br>H-1→L 7.5%	0.7964	454	2.73	H→L 80.5%<br>H-2→L 9.8%	0.4160
	**N7L$_2$**	380	3.26	H→L 38.5%<br>H-1→L+2 14.6%<br>H-2→L+1 11.0%<br>H-1→L 10.6%	0.1081	413	3.00	H→L 78.0%	1.4413

Table S5. Transition properties of **L_3**-based macrocycles.

		Absorption				Emission			
		λ_{abs} (nm)	E_{vert} (eV)	assignment	f	λ_{emi} (nm)	E_{vert} (eV)	assignment	f
**4-M**	**B4L$_3$**	345	3.59	H→L 55.7%<br>H-1→L+1 11.8%<br>H-1→L 9.3%<br>H-3→L 7.3%	0.3288	379	3.27	H→L 91.1%	1.1804
	**B3N1L$_3$**	379	3.27	H→L 70.5%<br>H→L+2 14.9%<br>H→L+1 5.3%	0.3027	426	2.91	H→L 66.3%<br>H→L+1 16.4%<br>H→L+2 9.1%	0.7417
	**B2N2L$_3$**	392	3.16	H→L 86.3%	0.3765	499	2.49	H→L 99.1%	0.0649
	**B1N3L$_3$**	387	3.21	H→L 75.9%<br>H-2→L 8.6%	0.1442	450	2.75	H→L 85.7%	0.4004
	**N4L$_3$**	361	3.44	H→L 34.6%<br>H-1→L 25.1%<br>H→L+1 21.5%<br>H-1→L+1 8.7%	0.1182	421	2.94	H→L 90.2%	0.7688
**5-M**	**B5L$_3$**	339	3.66	H→L 57.2%<br>H-1→L+1 24.9%<br>H-3→L+2 5.4%	0.2947	382	3.24	H→L 89.5%	1.3962
	**B4N1L$_3$**	394	3.15	H→L 55.0%<br>H→L+2 33.8%	0.6367	437	2.84	H→L 62.1%<br>H→L+2 26.5%	0.9952
	**B3N2L$_3$**	397	3.12	H→L 67.7%<br>H→L+1 12.9%<br>H→L+2 5.4%	0.4930	483	2.57	H→L 96.1%	0.1224
	**B2N3L$_3$**	397	3.12	H→L 72.9%<br>H-1→L+1 8.7%<br>H-2→L 6.1%	0.1020	485	2.56	H→L 95.5%	0.0829
	**B1N4L$_3$**	389	3.19	H-1→L 75.6%<br>H-3→L 7.5%<br>H→L+1 5.7%	0.5093	464	2.67	H→L 82.8%	0.6003
	**N5L$_3$**	369	3.36	H→L 64.9%<br>H-1→L+2 15.1%<br>H-2→L+1 10.9%	0.2391	420	2.95	H→L 83.4%<br>H-1→L+1 7.5%	0.9791
**6-M**	**B6L$_3$**	339	3.66	H→L 56.4%<br>H-2→L 11.5%	0.9591	384	3.23	H→L 87.3%	1.7499
	**B5N1L$_3$**	392	3.16	H→L+2 40.7%<br>H→L+1 38.4%<br>H→L 5.6%	1.0797	436	2.84	H→L+1 42.4%<br>H→L 37.9%<br>H→L+2 8.2%	1.4203
	**B4N2L$_3$**	392	3.16	H→L+1 50.7%<br>H→L 17.7%<br>H→L+2 13.9%<br>H-1→L+1 5.5%	0.5555	463	2.68	H→L 45.5%<br>H→L+1 37.3%<br>H→L+2 5.3%	0.7087
**6-M**	**B3N3L$_3$**	392	3.16	H→L 61.0%<br>H→L+2 11.5%<br>H→L+3 5.0%	0.5886	462	2.68	H→L 82.1%	0.7014
	**B2N4L$_3$**	380	3.26	H-2→L 28.3%<br>H→L 25.4%<br>H-1→L+1 17.5%<br>H→L+1 8.1%	0.1380	452	2.74	H→L 55.9%<br>H-1→L 24.2%<br>H-2→L 9.6%	0.9933
	**B1N5L$_3$**	378	3.28	H-2→L 63.9%<br>H→L 15.9%	0.7486	439	2.82	H→L 46.7%<br>H-1→L 30.2%<br>H-2→L 9.6%	1.0820
	**N6L$_3$**	357	3.47	H→L 63.7%<br>H-1→L+1 5.4%<br>H-1→L 5.3%	0.7239	411	3.01	H→L 89.8%	1.5457
**7-M**	**B7L$_3$**	341	3.64	H→L 44.0%<br>H-1→L+1 23.4%<br>H-2→L+2 13.1%	0.3756	382	3.25	H→L 88.0%	1.8307
	**B6N1L$_3$**	395	3.14	H→L+1 57.6%<br>H→L+3 27.0%	1.0655	440	2.82	H→L 61.2%<br>H→L+3 18.4%<br>H→L+1 8.6%	1.4116
	**B5N2L$_3$**	404	3.07	H→L 39.0%<br>H→L+2 27.0%<br>H→L+1 11.2%<br>H-1→L+1 7.9%	0.6386	463	2.68	H→L 73.8%<br>H→L+1 6.3%<br>H→L+2 5.9%	0.6287
	**B4N3L$_3$**	404	3.07	H→L 65.2%<br>H→L+1 10.0%<br>H-1→L+1 5.4%	0.4868	480	2.58	H→L 92.0%	0.2140
	**B3N4L$_3$**	404	3.07	H→L 71.6%<br>H-1→L+1 7.4%	0.2261	481	2.58	H→L 92.1%	0.1992
	**B2N5L$_3$**	401	3.09	H→L 54.3%<br>H-1→L 16.5%<br>H→L+1 5.7%<br>H-2→L 5.4%	0.5332	478	2.59	H→L 91.5%	0.2482
	**B1N6L$_3$**	396	3.13	H→L 37.8%<br>H-2→L 33.8%<br>H-4→L 8.4%<br>H-1→L 5.9%	0.8042	451	2.75	H→L 67.3%<br>H-2→L 8.9%<br>H-1→L 6.7%<br>H-4→L 5.4%	0.9587
	**N7L$_3$**	367	3.38	H→L 44.4%<br>H-1→L+1 21.1%<br>H-2→L+2 8.4%	0.3028	416	2.98	H→L 84.4%	1.8702

Table S6. The bond lengths (Å) between each B/N atoms and their adjacent C atoms in **$B2N2L_n$** (n = 1 ~ 3) at S_1-optimized structure.

	**$B2N2L_1$**	**$B2N2L_2$**	**$B2N2L_3$**	**$\Delta_{max - min}$**
N_1-C_5	1.432	1.415	1.428	0.017
N_1-C_6	1.378	1.384	1.376	0.008
N_1-C_{13}	1.402	1.415	1.409	0.013
N_2-C_7	1.378	1.384	1.375	0.009
N_2-C_8	1.432	1.415	1.428	0.017
N_2-C_{14}	1.402	1.415	1.411	0.013
B_3-C_9	1.599	1.574	1.586	0.025
B_3-C_{10}	1.537	1.545	1.538	0.008
B_3-C_{15}	1.557	1.569	1.565	0.012
B_4-C_{11}	1.537	1.545	1.541	0.008
B_4-C_{12}	1.599	1.574	1.582	0.025
B_4-C_{16}	1.557	1.569	1.564	0.012

Table S7. The bond lengths (Å) between each B/N atoms and their adjacent C atoms in **B3N2L$_n$** (n = 1 ~ 3) at S_1-optimized structure.

	**B3N2L$_1$**	**B3N2L$_2$**	**B3N2L$_3$**	**$\Delta_{max-min}$**
N_1-C_6	1.431	1.422	1.424	0.009
N_1-C_7	1.369	1.373	1.379	0.010
N_1-C_{16}	1.409	1.417	1.414	0.008
N_2-C_8	1.371	1.378	1.377	0.007
N_2-C_9	1.423	1.416	1.415	0.008
N_2-C_{17}	1.418	1.420	1.415	0.005
B_3-C_{10}	1.583	1.568	1.579	0.015
B_3-C_{11}	1.550	1.555	1.538	0.017
B_3-C_{18}	1.557	1.567	1.569	0.012
B_4-C_{12}	1.572	1.567	1.543	0.029
B_4-C_{13}	1.543	1.548	1.574	0.031
B_4-C_{19}	1.572	1.580	1.569	0.011
B_5-C_{14}	1.537	1.543	1.558	0.021
B_5-C_{15}	1.592	1.571	1.572	0.021
B_5-C_{20}	1.567	1.579	1.562	0.017

Table S8. The bond lengths (Å) between each B/N atoms and their adjacent C atoms in **B3N3L$_n$** (n = 1 ~ 3) at S_1-optimized structure.

	**B3N3L$_1$**	**B3N3L$_2$**	**B3N3L$_3$**	**$\Delta_{max - min}$**
N_1-C_7	1.423	1.414	1.405	0.018
N_1-C_8	1.378	1.398	1.375	0.023
N_1-C_{19}	1.417	1.415	1.409	0.008
N_2-C_9	1.392	1.411	1.385	0.026
N_2-C_{10}	1.392	1.384	1.421	0.037
N_2-C_{20}	1.419	1.419	1.414	0.005
N_3-C_{11}	1.378	1.380	1.413	0.035
N_3-C_{12}	1.423	1.417	1.405	0.018
N_3-C_{21}	1.417	1.417	1.412	0.005
B_4-C_{13}	1.579	1.572	1.537	0.042
B_4-C_{14}	1.541	1.543	1.574	0.033
B_4-C_{22}	1.568	1.573	1.573	0.005
B_5-C_{15}	1.555	1.546	1.561	0.015
B_5-C_{16}	1.554	1.567	1.565	0.013
B_5-C_{23}	1.577	1.576	1.564	0.013
B_6-C_{17}	1.541	1.556	1.576	0.035
B_6-C_{18}	1.579	1.568	1.560	0.019
B_6-C_{24}	1.568	1.569	1.566	0.003

Table S9. The bond lengths (Å) between each B/N atoms and their adjacent C atoms in **B4N3L$_n$** (n = 1 ~ 3) at S_1-optimized structure.

	**B4N3L$_1$**	**B4N3L$_2$**	**B4N3L$_3$**	**$\Delta_{max - min}$**
N_1-C_8	1.432	1.419	1.416	0.016
N_1-C_9	1.367	1.401	1.373	0.034
N_1-C_{22}	1.418	1.407	1.421	0.014
N_2-C_{10}	1.384	1.410	1.377	0.033
N_2-C_{11}	1.397	1.383	1.415	0.032
N_2-C_{23}	1.426	1.422	1.420	0.006
N_3-C_{12}	1.381	1.381	1.399	0.018
N_3-C_{13}	1.429	1.424	1.409	0.020
N_3-C_{24}	1.416	1.410	1.419	0.009
B_4-C_{14}	1.575	1.583	1.564	0.019
B_4-C_{15}	1.559	1.529	1.565	0.036
B_4-C_{25}	1.557	1.575	1.560	0.018
B_5-C_{16}	1.572	1.534	1.566	0.038
B_5-C_{17}	1.557	1.579	1.560	0.022
B_5-C_{26}	1.560	1.575	1.568	0.015
B_6-C_{18}	1.581	1.560	1.571	0.021
B_6-C_{19}	1.531	1.568	1.544	0.037
B_6-C_{27}	1.572	1.566	1.573	0.007
B_7-C_{20}	1.531	1.560	1.539	0.029
B_7-C_{21}	1.580	1.578	1.570	0.010
B_7-C_{28}	1.579	1.567	1.577	0.012

3. The coordinates of both the optimized structures of the macrocycles at S_0 and S_1 in toluene.

Structure S1. The coordinates of both the optimized structures of $B4L_1$ at S_0 and S_1 in toluene.

S_0-optimized structure				S_1-optimized structure			
B	-0.62221200	4.13781500	-0.00220700	B	0.02214300	-4.03852500	-0.05131900
C	-1.76980500	3.07965100	-0.20334200	C	-1.25290600	-3.14701700	0.12417000
C	-1.50712100	1.96993400	-1.02484000	C	-1.13572800	-1.90829200	0.76928300
C	-2.96050100	3.05447100	0.53477100	C	-2.54578100	-3.44821800	-0.37667200
C	-2.32700800	0.86002400	-1.02400500	C	-2.20794600	-1.02001900	0.85331300
H	-0.60257100	1.95437000	-1.62400700	H	-0.20243200	-1.63957900	1.25012200
C	-3.78974100	1.93178300	0.53568200	C	-3.60447300	-2.53162900	-0.35577300
H	-3.21314000	3.89645100	1.17245700	H	-2.70442700	-4.40581500	-0.86294300
C	-3.46437400	0.78562700	-0.20164200	C	-3.46009200	-1.25740100	0.19675200
H	-2.04677700	-0.00050800	-1.62266600	H	-2.10887200	-0.14878100	1.49282300
H	-4.66823500	1.92636700	1.17412300	H	-4.52982700	-2.79719600	-0.85625400
C	0.78557200	3.46410800	0.20108400	C	1.34525900	-3.21197100	-0.24801100
C	0.85995500	2.32683400	1.02354800	C	1.31642800	-2.08573300	-1.09066000
C	1.93174400	3.78941400	-0.53626200	C	2.51494300	-3.40717400	0.50063100
C	1.96997400	1.50706100	1.02465100	C	2.32882400	-1.14861200	-1.08083800
H	-0.00063400	2.04654100	1.62210900	H	0.44815600	-1.91545300	-1.71926300
C	3.05447000	2.96026400	-0.53516100	C	3.54129800	-2.46246100	0.50702200
H	1.92632100	4.66785800	-1.17478100	H	2.59524400	-4.27665600	1.14624300
C	3.07972300	1.76971600	0.20319800	C	3.44409300	-1.27706200	-0.23495600
H	1.95441600	0.60262000	1.62399200	H	2.22925700	-0.25947500	-1.69533500
H	3.89645200	3.21286500	-1.17286300	H	4.39560900	-2.61704900	1.15955800
C	-0.85303600	5.67235000	-0.00422500	C	-0.00762500	-5.59143300	-0.01497900
C	-2.03908600	6.22944700	-0.51025600	C	-1.05794900	-6.30320800	0.59132500
C	0.11619100	6.55539100	0.49977400	C	1.03473000	-6.34572000	-0.58236100
C	-2.24701200	7.60110500	-0.51922800	C	-1.07022400	-7.68989000	0.62833500
H	-2.80231000	5.57079800	-0.91137200	H	-1.86913300	-5.75537400	1.06000500
C	-0.08897300	7.92749000	0.50480300	C	1.02383700	-7.73271600	-0.56003900
H	1.03929300	6.15178100	0.90252900	H	1.86115500	-5.82735900	-1.05745400
C	-1.27137600	8.45101100	-0.00822100	C	-0.02978300	-8.40817400	0.04752400
H	-3.16698300	8.01059900	-0.92193100	H	-1.88831600	-8.21381000	1.11083400
H	0.66947600	8.59084500	0.90595600	H	1.83612100	-8.29018800	-1.01387000
H	-1.43288100	9.52376500	-0.00975900	H	-0.03885900	-9.49265300	0.07097600
B	4.13800100	0.62223600	0.00216100	B	4.33294500	0.00064100	-0.00014300
C	3.46454700	-0.78570300	-0.20080700	C	3.44364800	1.27806300	0.23459500
C	3.78958600	-1.93173600	0.53687400	C	3.54051700	2.46354700	-0.50729000
C	2.32745500	-0.86026900	-1.02354600	C	2.32837200	1.14920400	1.08040500
C	2.96035800	-3.05440300	0.53585300	C	2.51387000	3.40794600	-0.50087700
H	4.66785500	-1.92625300	1.17562600	H	4.39481600	2.61846700	-1.15976200
C	1.50753100	-1.97016300	-1.02446000	C	1.31567700	2.08600900	1.09023600
H	2.04741300	0.00014100	-1.62248000	H	2.22904600	0.26000900	1.69485600
C	1.76992800	-3.07975100	-0.20268200	C	1.34420600	3.21232200	0.24767800
H	3.21281900	-3.89625900	1.17377400	H	2.59395700	4.27750700	-1.14641000
H	0.60317300	-1.95465200	-1.62391900	H	0.44743600	1.91541400	1.71879600
C	-3.07978500	-1.76988200	0.20246400	C	-3.46048400	1.25631000	-0.19713900
C	-3.05426400	-2.96032500	-0.53605500	C	-3.60522600	2.53041200	0.35560700
C	-1.97032800	-1.50740000	1.02438300	C	-2.20833400	1.01940900	-0.85384300
C	-1.93151500	-3.78945900	-0.53691700	C	-2.54684100	3.44734400	0.37651400
H	-3.89605700	-3.21287900	-1.17402300	H	-4.53061300	2.79557400	0.85624100
C	-0.86033900	-2.32720500	1.02361000	C	-1.13641200	1.90803600	-0.76983100
H	-1.95496200	-0.60303200	1.62382600	H	-2.10902000	0.14822800	-1.49339400
C	-0.78561700	-3.46432300	0.20093700	C	-1.25393000	3.14663100	-0.12452700
H	-1.92584100	-4.66775500	-1.17563500	H	-2.70572700	4.40481900	0.86294600
H	-0.00004700	-2.04711500	1.62268600	H	-0.20307200	1.63970700	-1.25080100
B	0.62230300	-4.13786700	-0.00186900	B	0.02083300	4.03850400	0.05111300
C	-5.67254600	-0.85304600	0.00406100	C	-5.91789600	-0.00087800	-0.00008700
C	-6.22948200	-2.03929100	0.50983900	C	-6.66180900	1.10035200	-0.47837800
C	-6.55577000	0.11646900	-0.49904800	C	-6.66144100	-1.10232700	0.47827300
C	-7.60115300	-2.24709000	0.51949200	C	-8.04677000	1.10234500	-0.48018700
H	-5.57069300	-2.80277200	0.91023100	H	-6.13214300	1.96137000	-0.87328900
C	-7.92788500	-0.08858800	-0.50343900	C	-8.04640000	-1.10471100	0.48027000
H	-6.15229300	1.03971100	-0.90160600	H	-6.13148100	-1.96320800	0.87308800
C	-8.45124700	-1.27116300	0.00936000	C	-8.74860200	-0.00127600	0.00009700
H	-8.01051800	-3.16722300	0.92197100	H	-8.58678300	1.96301800	-0.86105400
H	-8.59140100	0.67010000	-0.90387800	H	-8.58612300	-1.96553900	0.86119800
H	-9.52402200	-1.43254000	0.01140700	H	-9.83321000	-0.00142100	0.00017200
C	0.85312800	-5.67238200	-0.00406600	C	-0.00944600	5.59141500	0.01522700
C	-0.11639300	-6.55551000	0.49922100	C	-1.06006100	6.30299400	-0.59080900
C	2.03942500	-6.22940300	-0.50959700	C	1.03266500	6.34589700	0.58278400
C	0.08871200	-7.92762100	0.50399400	C	-1.07285400	7.68968400	-0.62736800
H	-1.03967100	-6.15197500	0.90163300	H	-1.87106600	5.75500600	-1.05961500
C	2.24730000	-7.60106100	-0.51882500	C	1.02124700	7.73289700	0.56092500
H	2.80290700	-5.57068500	-0.91011100	H	1.85931300	5.82768400	1.05765400
C	1.27135400	-8.45106200	-0.00856100	C	-0.03265500	8.40816100	-0.04636200
H	-0.66999400	-8.59105600	0.90453100	H	-1.89115800	8.21345900	-1.10966300
H	3.16749800	-8.01049000	-0.92108400	H	1.83334500	8.29052500	1.01489600
H	1.43279600	-9.52382900	-0.01031300	H	-0.04213200	9.49264500	-0.06945000
C	5.67243300	0.85323600	0.00421700	C	5.88286600	0.00090700	-0.00005500
C	6.55556500	-0.11611800	-0.49943500	C	6.61066000	1.09539400	0.49589200
C	6.22949000	2.03943500	0.50994100	C	6.61110400	-1.09332100	-0.49592500
C	7.92765600	0.08905700	-0.50433900	C	7.99782700	1.09767200	0.50158800
H	6.15202500	-1.03931700	-0.90202800	H	6.07284700	1.95102200	0.89091300
C	7.60112600	2.24743500	0.51896100	C	7.99827100	-1.09509300	-0.50148300
H	5.57077400	2.80275500	0.91078000	H	6.07363900	-1.94915000	-0.89099000
C	8.45110300	1.27163800	0.00836600	C	8.69241500	0.00141600	0.00008600
H	8.59107100	-0.66951200	-0.90516300	H	8.54033000	1.95042300	0.89492400
H	8.01055800	3.16755200	0.92139500	H	8.54112500	-1.94764800	-0.89476100
H	9.52386200	1.43314400	0.00999600	H	9.77728800	0.00160800	0.00013500
B	-4.13798900	-0.62226300	0.00148000	B	-4.37808500	-0.00068300	-0.00016400

Structure S2. The coordinates of both the optimized structures of B3N1L$_1$ at S$_0$ and S$_1$ in toluene.

S_0-optimized structure				S_1-optimized structure			
B	4.14403900	0.13472400	-0.03578600	B	-4.12671400	-0.63134300	0.15410900
C	3.21399400	1.38420600	-0.23812900	C	-3.08526200	-1.82668900	-0.07199700
C	2.08087100	1.24540200	-1.05793500	C	-2.10500800	-1.62373200	-1.06809400
C	3.30016300	2.56253500	0.51808200	C	-2.92136200	-2.95629600	0.75346700
C	1.06423300	2.17664000	-1.06165100	C	-0.93511800	-2.34344500	-1.09329900
H	1.97931200	0.36355600	-1.68147300	H	-2.23208200	-0.80774600	-1.76976100
C	2.25334600	3.48036600	0.57201300	C	-1.77739300	-3.73661200	0.71301800
H	4.16464000	2.72806000	1.15360400	H	-3.67187800	-3.17537500	1.50529700
C	1.09743900	3.26033900	-0.17843400	C	-0.73793300	-3.37773600	-0.16374800
H	0.18825400	2.02801400	-1.68203700	H	-0.15018500	-2.10825400	-1.80213800
H	2.30627000	4.33011200	1.24343500	H	-1.62818700	-4.54023300	1.42512700
C	3.31914500	-1.19371500	0.15597000	C	-3.41546400	0.69744400	0.44958100
C	2.19769400	-1.14376900	1.00193700	C	-2.09066400	0.67914500	0.97446700
C	3.49691500	-2.36544200	-0.59085500	C	-3.87162400	1.99171100	0.06803000
C	1.25641500	-2.15242200	1.01574000	C	-1.26828000	1.77180800	0.95411100
H	2.03046000	-0.26107800	1.61048400	H	-1.70259000	-0.24986300	1.38023000
C	2.54391200	-3.38566200	-0.57777300	C	-3.04364600	3.09856500	0.06221000
H	4.35841300	-2.45798800	-1.24567900	H	-4.87646700	2.09899400	-0.32902100
C	1.37235300	-3.27877000	0.18339500	C	-1.67255300	3.02986500	0.42887500
H	0.37191500	-2.03686300	1.63368500	H	-0.25812500	1.66905400	1.33656600
H	2.68554900	-4.24663600	-1.22441100	H	-3.43525800	4.03251000	-0.33113900
C	5.69402900	0.20155700	-0.00027700	C	-5.64901300	-0.85313800	-0.05907100
C	6.45735300	-0.86160600	0.50961300	C	-6.61437100	0.04493800	0.44047100
C	6.38539900	1.32682900	-0.47863900	C	-6.14269200	-1.98708900	-0.73520600
C	7.84295600	-0.80325300	0.54795200	C	-7.97317300	-0.16444700	0.26182300
H	5.94872000	-1.74136600	0.88994800	H	-6.28401400	0.91384400	0.99941700
C	7.77115400	1.38864700	-0.45343900	C	-7.50022900	-2.20120000	-0.92140300
H	5.82066700	2.15896000	-0.88598700	H	-5.43791800	-2.71200500	-1.13190700
C	8.50067800	0.32286200	0.06355100	C	-8.42594700	-1.28793000	-0.42526100
H	8.41272800	-1.63243300	0.95297000	H	-8.68633900	0.54736000	0.66517700
H	8.28519000	2.26425300	-0.83465600	H	-7.84141300	-3.08266400	-1.45513200
H	9.58423200	0.36965800	0.08828600	H	-9.48874100	-1.45302000	-0.56603200
B	0.10061000	-4.18803300	-0.00218800	B	-0.59826700	4.09750600	0.14390500
C	-1.21322400	-3.33978700	-0.18328900	C	0.83452300	3.47504300	-0.15694200
C	-2.37757700	-3.50505000	0.57843700	C	2.00808200	3.73104000	0.56305300
C	-1.15256900	-2.20724200	-1.01310900	C	0.89734700	2.43458000	-1.10272700
C	-3.37880600	-2.53214300	0.59481100	C	3.13015000	2.90746500	0.45180100
H	-2.47699000	-4.37357700	1.22286200	H	2.02526100	4.54508700	1.28210400
C	-2.14198900	-1.24585500	-0.99665800	C	1.99433700	1.60340700	-1.19939900
H	-0.27516200	-2.04747900	-1.63133000	H	0.02256500	2.22466000	-1.70939900
C	-3.25855300	-1.35145900	-0.14940600	C	3.12651500	1.78010400	-0.38411200
H	-4.23403500	-2.66757000	1.25038400	H	3.98876900	3.09790900	1.08949600
H	-2.01842300	-0.35499200	-1.60374500	H	1.95331300	0.75694900	-1.87892800
C	-1.25123300	3.20407600	0.19233100	C	1.58655400	-2.96064700	0.01056800
C	-2.41815700	3.36960800	-0.55530300	C	2.71882900	-3.02337800	-0.80648700
C	-1.16431800	2.12100700	1.07296900	C	1.39670400	-1.86749300	0.86335300
C	-3.41944500	2.40238300	-0.50118700	C	3.59712700	-1.94528900	-0.82890400
H	-2.51394700	4.21707900	-1.22478700	H	2.86613100	-3.87162100	-1.46504700
C	-2.13452300	1.14165700	1.06849900	C	2.27597600	-0.80929300	0.81890300
H	-0.28130900	2.01367400	1.69187600	H	0.54114800	-1.85008600	1.52718800
C	-3.27504000	1.22759000	0.25158800	C	3.37159800	-0.79238000	-0.06066200
H	-4.29233000	2.52769700	-1.13442500	H	4.44104900	-1.97924100	-1.50955300
H	-1.98926500	0.26426700	1.68966700	H	2.08445900	0.05154500	1.44821900
B	-4.14499900	-0.06396000	0.04750800	B	4.12955900	0.59488800	-0.21108000
C	-0.13059100	5.39914900	-0.00597700	C	0.78271000	-5.28112700	0.06972100
C	-1.14082200	6.09318600	0.66978600	C	1.92925200	-5.74695800	0.73445700
C	0.84379500	6.13057200	-0.69467100	C	-0.13131200	-6.19662400	-0.47860800
C	-1.17531800	7.47932700	0.64470900	C	2.14806300	-7.10647900	0.84468700
H	-1.89469300	5.54195700	1.21891600	H	2.61581800	-5.03994500	1.18226500
C	0.80682400	7.51694100	-0.69710400	C	0.10058400	-7.55273500	-0.35055300
H	1.62526500	5.60809700	-1.23319200	H	-0.99253800	-5.83241900	-1.02437800
C	-0.20243900	8.20435300	-0.03334100	C	1.23778500	-8.01473900	0.30777200
H	-1.96792500	7.99607600	1.17503100	H	3.02681100	-7.46331600	1.36801300
H	1.57242600	8.06309400	-1.23756700	H	-0.60093700	-8.25593800	-0.78254200
H	-0.23027800	9.28768700	-0.04416400	H	1.41584700	-9.07917400	0.40035100
C	-5.69653100	-0.07037400	0.01414300	C	5.66860700	0.73328300	-0.10580500
C	-6.44004500	1.01887300	0.49766900	C	6.47203900	-0.31205700	0.37928000
C	-6.40912500	-1.16657900	-0.49912000	C	6.31162200	1.91630000	-0.50718000
C	-7.82720200	1.01473700	0.47414700	C	7.85013100	-0.18287300	0.46750900
H	-5.91513100	1.87536200	0.90781800	H	6.00246000	-1.23580700	0.70184600
C	-7.79597100	-1.17404000	-0.53596200	C	7.69030300	2.04910600	-0.43184200
H	-5.85965200	-2.01969800	-0.88329700	H	5.71423500	2.73560700	-0.89262600
C	-8.50592900	-0.08243100	-0.04635900	C	8.45983100	0.99909400	0.05873700
H	-8.38176900	1.86342900	0.85940600	H	8.45179500	-0.99885000	0.85260600
H	-8.32611500	-2.02782400	-0.94368300	H	8.16769500	2.96821500	-0.75306700
H	-9.59053400	-0.08704800	-0.06975600	H	9.53783400	1.10186400	0.12216700
C	0.13792300	-5.73900000	-0.00762100	C	-0.84898500	5.63624100	0.08397400
C	1.25719400	-6.44191800	0.46800300	C	-1.95286000	6.23647000	0.71634500
C	-0.94561400	-6.49169300	-0.48982800	C	0.04107400	6.49627100	-0.58386500
C	1.29317900	-7.82875800	0.46809500	C	-2.16303300	7.60743300	0.67577700
H	2.10571300	-5.88525600	0.85190800	H	-2.64801700	5.61001000	1.26540900
C	-0.91291300	-7.87856900	-0.50330000	C	-0.16393600	7.86793000	-0.63466300
H	-1.82063000	-5.97402100	-0.86883000	H	0.90871500	6.07130000	-1.07903600
C	0.20732200	-8.54781500	-0.02105500	C	-1.26974900	8.42967800	-0.00446600
H	2.16453400	-8.35178300	0.84677200	H	-3.02209100	8.03938800	1.17888500
H	-1.75747100	-8.44031600	-0.88718300	H	0.53849900	8.50272000	-1.16517300
H	0.23410100	-9.63233700	-0.02638500	H	-1.43179300	9.50190900	-0.03833900
N	-0.09558800	4.00151800	0.00710800	N	0.54892500	-3.91969000	-0.04971200

Structure S3. The coordinates of both the optimized structures of B2N2L$_1$ at S$_0$ and S$_1$ in toluene.

S_0-optimized structure				S_1-optimized structure			
B	2.87778000	-2.99101700	-0.04277800	B	-2.90588300	2.96887700	-0.09073500
C	3.17277100	-1.46323700	-0.25504400	C	-1.35972700	3.34027300	0.08090100
C	2.30685300	-0.72979600	-1.08428800	C	-0.66247700	2.71014500	1.12539500
C	4.09433100	-0.71972700	0.49746100	C	-0.58478700	4.03474500	-0.85962500
C	2.30452800	0.64820900	-1.10294900	C	0.71634000	2.61924000	1.13372000
H	1.59160500	-1.25777900	-1.70589200	H	-1.22206400	2.19602300	1.89922400
C	4.06304500	0.67243400	0.53560100	C	0.80664700	3.95293800	-0.87489000
H	4.79838400	-1.23632700	1.14253300	H	-1.07819300	4.57361000	-1.66229900
C	3.12287900	1.36925600	-0.22700300	C	1.44983300	3.17977900	0.08878500
H	1.60568400	1.18398400	-1.73408500	H	1.23018600	2.05018700	1.90084200
H	4.72396600	1.21328700	1.20351900	H	1.37876600	4.40466600	-1.67802800
C	1.33795600	-3.27233300	0.13949400	C	-3.11850200	1.46100300	-0.30118400
C	0.62379800	-2.40911800	0.98833200	C	-2.04230000	0.68347700	-0.81470700
C	0.58499900	-4.17464300	-0.62269600	C	-4.25146400	0.69124500	0.09171000
C	-0.75517400	-2.37140400	0.98770500	C	-2.04248600	-0.68291300	-0.81476900
H	1.16718900	-1.70336800	1.60821800	H	-1.17607400	1.19897400	-1.21494200
C	-0.81115900	-4.13673700	-0.62304600	C	-4.25164500	-0.69016700	0.09165600
H	1.09218000	-4.87467200	-1.28029500	H	-5.12100900	1.20464900	0.49180900
C	-1.51447800	-3.19484100	0.13870900	C	-3.11888400	-1.46019200	-0.30128800
H	-1.26004300	-1.63677300	1.60666800	H	-1.17639300	-1.19860600	-1.21504000
H	-1.35530500	-4.80825000	-1.28080600	H	-5.12132000	-1.20337300	0.49172600
C	3.97082200	-4.09085000	0.01596300	C	-4.01780500	4.05351600	0.01867700
C	5.27502400	-3.84859000	-0.44564400	C	-3.76007900	5.30655500	0.60597000
C	3.69056500	-5.36723200	0.53100600	C	-5.31592500	3.85299100	-0.48850000
C	6.25283500	-4.83168800	-0.39874800	C	-4.73609100	6.28788100	0.70347400
H	5.51628800	-2.87406200	-0.85740800	H	-2.76864000	5.50644700	1.00126300
C	4.66535600	-6.35268900	0.59058700	C	-6.29660300	4.82997500	-0.39922200
H	2.69191300	-5.58012500	0.89808700	H	-5.54845600	2.91452800	-0.98041000
C	5.94789800	-6.08481300	0.12293800	C	-6.01274600	6.05283700	0.20232600
H	7.25184200	-4.62547300	-0.76717700	H	-4.50378200	7.23983600	1.17025100
H	4.42942200	-7.32923000	0.99919400	H	-7.28563000	4.64236400	-0.80482700
H	6.71131200	-6.85449000	0.16435000	H	-6.77824300	6.81822300	0.27373500
B	-3.03676300	-2.83078500	-0.04349000	B	-2.90664200	-2.96813400	-0.09091000
C	-3.24712300	-1.28917000	-0.25572000	C	-1.36058800	-3.33989700	0.08081800
C	-4.12919000	-0.49737900	0.49461900	C	-0.58578800	-4.03460600	-0.85964400
C	-2.33940500	-0.60335000	-1.08109700	C	-0.66321900	-2.70986200	1.12529000
C	-4.02246700	0.89115900	0.53413800	C	0.80566700	-3.95312300	-0.87487900
H	-4.86250500	-0.97547500	1.13680100	H	-1.07930100	-4.57339800	-1.66230200
C	-2.26208000	0.77241900	-1.09834900	C	0.71562200	-2.61930400	1.13366100
H	-1.65182000	-1.16903800	-1.70072400	H	-1.22271000	-2.19554400	1.89906000
C	-3.04330600	1.53641600	-0.22459100	C	1.44901100	-3.18009100	0.08878600
H	-4.65510200	1.46638900	1.20071400	H	1.37770200	-4.40503300	-1.67797400
H	-1.53316300	1.26994600	-1.72673100	H	1.22958500	-2.05033000	1.90076300
C	1.48205800	3.05849100	0.08419500	C	2.98788900	1.41037900	-0.21198300
C	0.80074900	3.89277400	-0.80335800	C	4.08088700	0.68784400	0.32240100
C	0.75641100	2.37744700	1.06226400	C	1.96823600	0.68068900	-0.86433300
C	-0.58982000	3.93035500	-0.80201600	C	4.08072800	-0.68884200	0.32240200
H	1.35502300	4.44688100	-1.55251000	H	4.87937800	1.21683800	0.82708000
C	-0.62448000	2.41461700	1.06351800	C	1.96808700	-0.68119600	-0.86434900
H	1.28546400	1.76171000	1.78020500	H	1.17658500	1.21612800	-1.36942600
C	-1.31371400	3.13395500	0.08667800	C	2.98756400	-1.41113000	-0.21198800
H	-1.11483900	4.51339000	-1.55028300	H	4.87909000	-1.21801200	0.82709900
H	-1.18467700	1.82816100	1.78236600	H	1.17631800	-1.21644900	-1.36944900
C	3.86000600	3.72473700	0.00567500	C	3.86560200	3.69258300	0.02009800
C	5.11455700	3.51076300	-0.57822000	C	3.71697800	4.84713600	0.79441800
C	3.61883600	4.94289400	0.65193500	C	5.04827800	3.48560100	-0.69823900
C	6.09745800	4.48601100	-0.50243800	C	4.74830400	5.76966000	0.85860700
H	5.31313500	2.58149200	-1.09838800	H	2.79914800	5.00700800	1.34649600
C	4.60638900	5.91526800	0.70628200	C	6.07556300	4.41167200	-0.61552800
H	2.65646200	5.12136100	1.11632400	H	5.14604900	2.61747700	-1.33879700
C	5.85468500	5.69670600	0.13600900	C	5.93306800	5.55582600	0.16193800
H	7.06163800	4.29772500	-0.96236300	H	4.62660500	6.65971400	1.46472900
H	4.39616300	6.85107100	1.21292600	H	6.98622800	4.24648400	-1.17956900
H	6.62489300	6.45736400	0.18673400	H	6.73641700	6.28079600	0.21694300
C	-3.65127100	3.92773300	0.01220200	C	3.86464100	-3.69356800	0.02046200
C	-3.34270300	5.13184700	0.65578000	C	5.04745200	-3.48712300	-0.69780800
C	-4.91746400	3.78086500	-0.56742700	C	3.71558200	-4.84789600	0.79502900
C	-4.27619700	6.15624700	0.71147800	C	6.07445100	-4.41348200	-0.61476200
H	-2.37060700	5.25897100	1.11684900	H	5.14555400	-2.61918800	-1.33857300
C	-5.84606400	4.80775200	-0.49031700	C	4.74662500	-5.77071400	0.85955400
H	-5.16742700	2.86276300	-1.08530900	H	2.79763700	-5.00736000	1.34703300
C	-5.53620200	6.00461800	0.14534300	C	5.93153000	-5.55740400	0.16296700
H	-4.01404500	7.08000300	1.21602200	H	6.98522800	-4.24871100	-1.17874400
H	-6.82044600	4.67102300	-0.94695500	H	4.62458400	-6.66058700	1.46587400
H	-6.26402700	6.80585900	0.19703100	H	6.73465600	-6.28260100	0.21823700
C	-4.18799700	-3.86951100	0.01223300	C	-4.01884100	-4.05250400	0.01831100
C	-5.47666300	-3.55503900	-0.44976500	C	-3.76152500	-5.30561400	0.60563100
C	-3.97817700	-5.16093800	0.52307500	C	-5.31680800	-3.85166400	-0.48913300
C	-6.50653100	-4.48371400	-0.40733500	C	-4.73778600	-6.28671600	0.70291200
H	-5.66402900	-2.56751400	-0.85843600	H	-2.77021400	-5.50574300	1.00112600
C	-5.00508700	-6.09219400	0.57804200	C	-6.29773000	-4.82842500	-0.40008200
H	-2.99307600	-5.42894000	0.89072800	H	-5.54901100	-2.91314100	-0.98108200
C	-6.27064300	-5.75340800	0.11002900	C	-6.01428000	-6.05136600	0.20149900
H	-7.49245300	-4.22217000	-0.77584100	H	-4.50579400	-7.23873500	1.16971500
H	-4.82304900	-7.08165200	0.98310000	H	-7.28662800	-4.64058200	-0.80589500
H	-7.07462400	-6.48079400	0.14772100	H	-6.77996700	-6.81657900	0.27272500
N	2.86182400	2.75045700	-0.06512600	N	2.81372900	2.76844700	-0.05595300
N	-2.70767200	2.90102100	-0.06101600	N	2.81303500	-2.76913800	-0.05591100

Structure S4. The coordinates of both the optimized structures of B1N3L$_1$ at S$_0$ and S$_1$ in toluene.

S_0-optimized structure				S_1-optimized structure			
B	4.11256600	0.00119600	-0.00027900	B	-2.06312600	-3.57111300	-0.11898700
C	3.22750200	1.28057900	-0.22517200	C	-0.52756700	-3.43627000	-0.32505300
C	2.09858900	1.15362200	-1.05315600	C	-0.00235400	-2.45183000	-1.20735600
C	3.33456000	2.47387200	0.50363200	C	0.44373800	-4.09086700	0.48048700
C	1.10502500	2.10752900	-1.08781300	C	1.32017600	-2.07520300	-1.20158100
H	1.98062300	0.26118100	-1.65836900	H	-0.67843800	-1.95161400	-1.89276200
C	2.30956500	3.41797000	0.52729800	C	1.77115600	-3.69726000	0.52568700
H	4.19663900	2.63429300	1.14394200	H	0.11920900	-4.86491100	1.16796800
C	1.15345800	3.21044400	-0.22865200	C	2.21725400	-2.64800000	-0.28960000
H	0.23229700	1.96303600	-1.71346400	H	1.67278400	-1.29247200	-1.86627000
H	2.37948000	4.27951900	1.18179500	H	2.45322500	-4.15376700	1.23600300
C	3.22827000	-1.27867100	0.22490700	C	-2.79554900	-2.14810800	-0.20446800
C	2.09947000	-1.15242000	1.05313900	C	-2.64499100	-1.30609100	0.90923500
C	3.33587800	-2.47186600	-0.50399100	C	-3.39368600	-1.57569800	-1.33696900
C	1.10646600	-2.10690600	1.08793800	C	-2.92423600	0.04925000	0.85196200
H	1.98109700	-0.26008600	1.65842900	H	-2.22272700	-1.71433700	1.82277200
C	2.31144900	-3.41658400	-0.52746800	C	-3.69511400	-0.21582700	-1.41112400
H	4.19792400	-2.63173700	-1.14448700	H	-3.57267900	-2.19277800	-2.21231600
C	1.15538100	-3.20976200	0.22873600	C	-3.39906200	0.61068300	-0.33140100
H	0.23376800	-1.96294200	1.71375100	H	-2.71824900	0.69476400	1.69959200
H	2.38174200	-4.27808900	-1.18198400	H	-4.08716400	0.21624700	-2.32621800
C	5.66461100	0.00164100	-0.00051300	C	-2.80383300	-4.83544400	0.35487700
C	6.39326100	-1.08419100	0.51204900	C	-4.14388800	-4.77917200	0.81098300
C	6.39250200	1.08788400	-0.51329300	C	-2.22695600	-6.12931200	0.31984100
C	7.78065700	-1.08583500	0.51784900	C	-4.83613600	-5.90570500	1.22199300
H	5.85583400	-1.93347200	0.92114000	H	-4.64303400	-3.81526600	0.84163700
C	7.77989400	1.09031400	-0.51945100	C	-2.91521400	-7.25851000	0.72837300
H	5.85448700	1.93684600	-0.92228300	H	-1.21709100	-6.24212500	-0.06153800
C	8.47491900	0.00244000	-0.00088900	C	-4.22803700	-7.16005900	1.19063500
H	8.32345400	-1.93201200	0.92485800	H	-5.86050300	-5.81087600	1.57005000
H	8.32210500	1.93678900	-0.92662100	H	-2.43152900	-8.22966200	0.68003800
H	9.55979900	0.00275600	-0.00103800	H	-4.76882300	-8.04446700	1.50928900
C	-1.19482100	-3.17903500	-0.09065300	C	-2.11222400	2.58970500	-0.41598900
C	-2.26742500	-3.23170900	0.80070800	C	-1.74634800	3.67923400	0.39543400
C	-1.19234300	-2.20029500	-1.08465300	C	-1.10387200	1.93472000	-1.14245800
C	-3.24182500	-2.23922300	0.78488600	C	-0.41185800	3.99038500	0.59677400
H	-2.29387900	-4.00201400	1.56332100	H	-2.50608000	4.21786100	0.94846000
C	-2.15916600	-1.21395400	-1.10013300	C	0.21525400	2.25151900	-0.95375300
H	-0.38259700	-2.18091400	-1.80479500	H	-1.37554900	1.14341900	-1.82810400
C	-3.15463800	-1.18114700	-0.12257300	C	0.59098700	3.23246900	-0.02315200
H	-4.02603100	-2.24118100	1.53361400	H	-0.14395400	4.76416800	1.30663900
H	-2.11302500	-0.41847800	-1.83461600	H	0.98254100	1.71886100	-1.50156200
C	-1.19665100	3.17829100	0.09128000	C	3.32604700	-0.63990200	0.28435100
C	-2.26951900	3.23033100	-0.79978900	C	4.16073000	0.38612500	-0.21412000
C	-1.19335500	2.19953500	1.08526700	C	2.20432500	-0.26426900	1.05556000
C	-3.24337700	2.23731900	-0.78369300	C	3.77734100	1.70941600	-0.11968100
H	-2.29662400	4.00060800	-1.56241000	H	5.04517400	0.13109500	-0.78420100
C	-2.15962600	1.21266700	1.10103400	C	1.83378200	1.04663100	1.15641100
H	-0.38341300	2.18062400	1.80520300	H	1.60643700	-1.03400800	1.52497900
C	-3.15535800	1.17927900	0.12374100	C	2.56060800	2.05385900	0.49319600
H	-4.02777500	2.23887200	-1.53221900	H	4.36521000	2.47204700	-0.61688100
H	-2.11283100	0.41722600	1.83551300	H	0.94430600	1.31530100	1.71278200
C	-0.05250400	5.36661400	-0.01175700	C	4.68840600	-2.58580700	-0.33151200
C	-1.11059400	6.03283300	0.61770800	C	5.84941900	-2.19015200	0.34437800
C	0.97061100	6.12740900	-0.59099500	C	4.75711100	-3.63568200	-1.25349600
C	-1.13985900	7.41886300	0.66065000	C	7.05656300	-2.81529600	0.07529900
H	-1.90642800	5.45958300	1.07783100	H	5.79530200	-1.41214800	1.09666600
C	0.93562800	7.51201200	-0.52676900	C	5.96920200	-4.25785700	-1.50557100
H	1.78791000	5.62937400	-1.09846800	H	3.85708100	-3.95081500	-1.76655700
C	-0.11805400	8.17194400	0.09515400	C	7.12664500	-3.85056500	-0.85060000
H	-1.97004300	7.91250400	1.15433900	H	7.94637900	-2.50220700	0.60999700
H	1.73944300	8.07995600	-0.98277800	H	6.00829900	-5.06728000	-2.22582300
H	-0.14280600	9.25456100	0.13683000	H	8.07131200	-4.34227100	-1.05116800
C	-5.32861500	-0.00154800	-0.00018000	C	2.64304900	4.51243500	0.45903800
C	-6.04407500	1.08084700	0.52713400	C	3.72664800	4.60933700	1.33837500
C	-6.04283900	-1.08431300	-0.52838000	C	2.25825700	5.62814100	-0.29164400
C	-7.43075200	1.07626100	0.51609900	C	4.41598100	5.80561200	1.45408200
H	-5.50896000	1.92164400	0.95182700	H	4.00895600	3.75272600	1.93857800
C	-7.42954300	-1.08037500	-0.51921400	C	2.94794800	6.82193700	-0.15435100
H	-5.50676600	-1.92486800	-0.95234700	H	1.43355400	5.54801000	-0.98940600
C	-8.13796700	-0.00222900	-0.00204100	C	4.03058900	6.91798800	0.71353800
H	-7.96225000	1.92621700	0.93058800	H	5.25132500	5.87216100	2.14158300
H	-7.96006800	-1.93059900	-0.93439600	H	2.64490400	7.68024900	-0.74287300
H	-9.22164900	-0.00248400	-0.00276700	H	4.56903100	7.85297300	0.81288400
C	-0.04935600	-5.36665900	0.01232900	C	-4.56979300	2.79266600	-0.38582800
C	0.97437500	-6.12674100	0.59141500	C	-5.73802000	2.23228000	0.14226800
C	-1.10725300	-6.03360700	-0.61667900	C	-4.60870200	4.10146600	-0.88333200
C	0.94014000	-7.51137800	0.52754200	C	-6.90959300	2.97271600	0.17966300
H	1.79153700	-5.62811900	1.09853000	H	-5.71985100	1.21906700	0.52405600
C	-1.13577200	-7.41966400	-0.65925600	C	-5.78371800	4.83425300	-0.82613400
H	-1.90355100	-5.46090400	-1.07668300	H	-3.72238300	4.53348100	-1.33260200
C	-0.11337600	-8.17204100	-0.09388600	C	-6.94262100	4.27859700	-0.29542100
H	1.74441900	-8.07876400	0.98342700	H	-7.80464500	2.52175500	0.59351200
H	-1.96582700	-7.91389300	-1.15257200	H	-5.79526500	5.84530600	-1.21819600
H	-0.13754600	-9.25468000	-0.13529500	H	-7.86068600	4.85319900	-0.26007700
N	-0.02842200	3.97244700	-0.07251100	N	3.45956000	-1.97274400	-0.06126200
N	-3.93473100	-0.00117200	0.00072000	N	1.94915400	3.30213400	0.33359800
N	-0.02607700	-3.97247900	0.07284200	N	-3.39411700	2.04099000	-0.44848900

Structure S5. The coordinates of both the optimized structures of N4L$_1$ at S$_0$ and S$_1$ in toluene.

S_0-optimized structure				S_1-optimized structure			
C	-2.01491900	2.69967100	0.14500200	C	1.53170800	2.99183000	-0.20441500
C	-1.17487500	2.17077200	1.12530800	C	0.77332300	2.17452900	-1.06745600
C	-1.49608100	3.63853800	-0.74919000	C	0.85181000	3.96225700	0.56327500
C	0.17527800	2.46152400	1.12537500	C	-0.59440000	2.23028000	-1.06773300
H	-1.58038000	1.47277700	1.84822400	H	1.29055900	1.47264900	-1.71099700
C	-0.13636800	3.93100300	-0.74952800	C	-0.52775500	4.01839000	0.56316400
H	-2.14147000	4.07835400	-1.50137700	H	1.41616300	4.62050900	1.21483300
C	0.72273100	3.28939600	0.14482500	C	-1.28404100	3.10650900	-0.20485000
H	0.83212200	1.99239100	1.84853400	H	-1.16673200	1.57299500	-1.71177600
H	0.27161400	4.59756000	-1.50116100	H	-1.03707600	4.72038000	1.21439300
C	-3.28917900	0.72306200	-0.14637500	C	3.10646100	1.28404100	0.20518400
C	-2.46084500	0.17653500	-1.12717600	C	2.23016600	0.59438200	1.06799000
C	-3.93136100	-0.13698200	0.74669000	C	4.01838700	0.52776400	-0.56277400
C	-2.17000300	-1.17369700	-1.12830900	C	2.17442600	-0.77333800	1.06770000
H	-1.99150900	0.83404900	-1.84959200	H	1.57283200	1.16670200	1.71199300
C	-3.63875800	-1.49676700	0.74523600	C	3.96226600	-0.85180400	-0.56290000
H	-4.59846900	0.27022000	1.49827100	H	4.72041800	1.03708700	-1.21395700
C	-2.69923800	-2.01469600	-0.14891400	C	2.99179000	-1.53172400	0.20471100
H	-1.47174800	-1.57847300	-1.85139400	H	1.47249700	-1.29058200	1.71117900
H	-4.07908100	-2.14290400	1.49649200	H	4.62057100	-1.41613400	-1.21442600
C	-4.48121400	2.89065600	-0.00247600	C	3.90914800	3.60407700	0.00070600
C	-4.49659700	4.18808600	0.52532400	C	3.75492400	4.81877900	-0.67802400
C	-5.66859300	2.36811700	-0.53118900	C	5.10694700	3.35118200	0.67986300
C	-5.66427200	4.93576600	0.51447800	C	4.77507600	5.75697700	-0.66590300
H	-3.59119400	4.60408200	0.95033300	H	2.83843800	5.01261700	-1.22205600
C	-6.83170500	3.12314700	-0.52150300	C	6.12456000	4.29216300	0.66975100
H	-5.67397800	1.37125200	-0.95534200	H	5.22526800	2.42150000	1.22329400
C	-6.84313400	4.41308200	-0.00400700	C	5.96769900	5.50206600	0.00239200
H	-5.65034600	5.93805700	0.92915300	H	4.63936400	6.69223000	-1.19797000
H	-7.73819900	2.69606200	-0.93689800	H	7.04532400	4.08049800	1.20225800
H	-7.75408900	4.99995700	-0.00445800	H	6.76467400	6.23655800	0.00286800
C	-0.72241900	-3.28924000	0.14143100	C	1.28402700	-3.10649600	-0.20495100
C	0.13700400	-3.93172500	-0.75187100	C	0.52773500	-4.01836000	0.56307500
C	-0.17543600	-2.46069700	1.12169500	C	0.59440800	-2.23025300	-1.06783000
C	1.49670100	-3.63917100	-0.75120200	C	-0.85182900	-3.96223100	0.56315900
H	-0.27072900	-4.59878500	-1.50319600	H	1.03706100	-4.72034200	1.21431000
C	1.17476200	-2.17001100	1.12206900	C	-0.77331700	-2.17450300	-1.06757800
H	-0.83256500	-1.99102800	1.84421700	H	1.16675400	-1.57295500	-1.71184800
C	2.01506200	-2.69960200	0.14245700	C	-1.53170900	-2.99181800	-0.20456400
H	2.14253100	-4.07945100	-1.50273400	H	-1.41620700	-4.62047700	1.21470200
H	1.58001500	-1.47140900	1.84453600	H	-1.29054600	-1.47261100	-1.71111100
C	2.69948400	2.01486000	-0.14596200	C	-2.99179300	1.53170300	0.20472000
C	3.63808600	1.49560700	0.74828700	C	-3.96223000	0.85183200	-0.56297300
C	2.17116500	1.17530400	-1.12710700	C	-2.17447400	0.77327900	1.06771200
C	3.93091700	0.13588200	0.74793900	C	-4.01836900	-0.52773500	-0.56291300
H	4.07749500	2.14067800	1.50099500	H	-4.62049300	1.41620500	-1.21450500
C	2.46217900	-0.17491300	-1.12784900	C	-2.23023800	-0.59444300	1.06794600
H	1.47346800	1.58121600	-1.85008100	H	-1.47257800	1.29048500	1.71125900
C	3.28981600	-0.72290900	-0.14720500	C	-3.10649000	-1.28406100	0.20506400
H	4.59721600	-0.27234000	1.49966700	H	-4.72037500	-1.03701700	-1.21415500
H	1.99364000	-0.83138400	-1.85171300	H	-1.57293600	-1.16679800	1.71195100
C	2.89081100	4.48102300	0.00360500	C	-3.60409200	3.90913800	-0.00029700
C	4.18838900	4.49629300	-0.52379700	C	-4.81879200	3.75484800	0.67842300
C	2.36907800	5.66789200	0.53432200	C	-3.35124100	5.10696700	-0.67941600
C	4.93692600	5.66342200	-0.51089800	C	-5.75702400	4.77496900	0.66633900
H	4.60375900	3.59124900	-0.95021100	H	-5.01260400	2.83833200	1.22241600
C	3.12490300	6.83050400	0.52662200	C	-4.29226000	6.12454800	-0.66927200
H	1.37230700	5.67317500	0.95866800	H	-2.42157100	5.22534100	-1.22285400
C	4.41483900	6.84190200	0.00907500	C	-5.50215500	5.96762600	-0.00191500
H	5.93929700	5.64945200	-0.92536100	H	-6.69227200	4.63920100	1.19839900
H	2.69848200	7.73660300	0.94357800	H	-4.08062700	7.04533400	-1.20175300
H	5.00234600	7.75244700	0.01104500	H	-6.23667500	6.76457400	-0.00236500
C	4.48119600	-2.89102100	-0.00292200	C	-3.90916700	-3.60408200	0.00028500
C	5.66920800	-2.36984700	-0.53167000	C	-5.10709100	-3.35125900	0.67925300
C	4.49542900	-4.18769700	0.52659100	C	-3.75479800	-4.81874700	-0.67847700
C	6.83188600	-3.12543800	-0.52011300	C	-6.12469100	-4.29225000	0.66889000
H	5.67525600	-1.37375300	-0.95757400	H	-5.22552900	-2.42162200	1.22273500
C	5.66275500	-4.93598100	0.51767600	C	-4.77494400	-5.75695500	-0.66661000
H	3.58944200	-4.60251300	0.95152600	H	-2.83820400	-5.01254500	-1.22234300
C	6.84227700	-4.41452000	-0.00045500	C	-5.96769500	-5.50210800	0.00148000
H	7.73887700	-2.69954800	-0.93565000	H	-7.04555200	-4.08063300	1.20124800
H	5.64805600	-5.93769400	0.93368800	H	-4.63911400	-6.69217300	-1.19870800
H	7.75294000	-5.00185100	0.00062600	H	-6.76466000	-6.23661000	0.00176200
C	-2.89073600	-4.48059800	0.00164100	C	3.60410900	-3.90912800	-0.00070200
C	-2.36863700	-5.66691900	0.53310300	C	3.35114200	-5.10685800	-0.67995000
C	-4.18886600	-4.49619400	-0.52433700	C	4.81893300	-3.75493100	0.67782300
C	-3.12478000	-6.82932200	0.52774400	C	4.29218000	-6.12442500	-0.67014600
H	-1.37131000	-5.67203100	0.95611700	H	2.42136700	-5.22515800	-1.22322300
C	-4.93760500	-5.66312000	-0.50925000	C	5.75717800	-4.77503300	0.66539600
H	-4.60455500	-3.59147500	-0.95114600	H	5.01283200	-2.83850000	1.22192700
C	-4.41535200	-6.84099300	0.01191500	C	5.50220000	-5.96758900	-0.00299900
H	-2.69800600	-7.73500900	0.94520500	H	4.08045900	-7.04513200	-1.20272800
H	-5.94046400	-5.64939100	-0.92255200	H	6.69252600	-4.63934000	1.19729900
H	-5.00311600	-7.75136500	0.01578400	H	6.23673200	-6.76452600	-0.00371500
N	-3.31058000	2.13590600	-0.00011200	N	2.88230900	2.65737400	0.00057100
N	2.13567300	3.31055300	-0.00007300	N	-2.65736400	2.88232400	-0.00019400
N	3.31070300	-2.13596200	-0.00325700	N	-2.88233500	-2.65738100	0.00041100
N	-2.13538200	-3.31036600	-0.00468100	N	2.65736700	-2.88232700	-0.00025300

Structure S6. The coordinates of both the optimized structures of B5L$_1$ at S$_0$ and S$_1$ in toluene.

S_0-optimized structure				S_1-optimized structure			
C	-3.49398700	2.67422400	-0.33559200	C	-4.46602400	-1.41796900	0.06610100
C	-3.49491500	3.82965900	0.46316900	C	-4.61230800	-0.63102700	1.24563900
C	-2.27758900	2.32882400	-0.94325000	C	-4.46193700	-0.73551200	-1.18750100
C	-2.34395100	4.58661800	0.65040000	C	-4.49143000	0.73289800	1.19152200
H	-4.40619600	4.12623100	0.97249400	H	-4.72311300	-1.12971700	2.20101300
C	-1.13527200	3.08844400	-0.76647800	C	-4.32284400	0.62901700	-1.23854200
H	-2.21366000	1.43468600	-1.55094200	H	-4.47535400	-1.31983500	-2.09948100
C	-1.12758700	4.23348300	0.04654100	C	-4.32129000	1.40232900	-0.05118400
H	-2.38469000	5.45648600	1.29841800	H	-4.51614800	1.31223800	2.10768700
H	-0.21756100	2.77633900	-1.25293800	H	-4.20903800	1.12384800	-2.19632400
C	-4.51957100	0.19308200	-0.39034500	C	-2.71962200	-3.45312200	0.08955300
C	-3.84832600	-0.51403900	-1.39868800	C	-1.64284400	-2.55437500	-0.16891500
C	-4.87606600	-0.52476800	0.75894100	C	-2.32506500	-4.80041100	0.34364400
C	-3.46128100	-1.83244000	-1.21990600	C	-0.33205700	-2.95563200	-0.17690900
H	-3.59666400	-0.01942700	-2.33163700	H	-1.86517300	-1.51203000	-0.38069200
C	-4.54091600	-1.86650000	0.91117200	C	-1.00811600	-5.19430000	0.33884500
H	-5.40641700	-0.02290300	1.56313100	H	-3.08941300	-5.53303300	0.58383700
C	-3.78483700	-2.54245000	-0.05461100	C	0.06481900	-4.29513700	0.08481900
H	-2.88341000	-2.32091900	-1.99866000	H	0.43141600	-2.22038500	-0.40698400
H	-4.83350100	-2.38335400	1.82031900	H	-0.77556800	-6.22833400	0.57415800
C	-6.19698500	2.33264600	-0.62078300	C	-5.45897900	-3.83475300	0.16775700
C	-7.33047300	1.51615100	-0.46768100	C	-5.52771200	-5.02234800	-0.58743900
C	-6.41488000	3.67653100	-0.97020200	C	-6.61511500	-3.46452100	0.88255600
C	-8.61394800	2.01978400	-0.61770800	C	-6.66046400	-5.82144200	-0.57660900
H	-7.19776700	0.46689700	-0.22773300	H	-4.67248200	-5.31937900	-1.18392900
C	-7.69502600	4.18428500	-1.13905400	C	-7.75148800	-4.26053700	0.89076700
H	-5.56328800	4.32861900	-1.12863800	H	-6.61975400	-2.54794000	1.46276700
C	-8.79734300	3.35708400	-0.95360800	C	-7.77937700	-5.44498400	0.16324600
H	-9.47230200	1.37056500	-0.48405700	H	-6.67496700	-6.74100200	-1.15225600
H	-7.83584900	5.22322000	-1.41642700	H	-8.61937000	-3.95633900	1.46639600
H	-9.79964500	3.75212200	-1.08073300	H	-8.66746600	-6.06736400	0.16505700
C	-1.64143700	-4.11312500	0.04709500	C	2.64631000	-3.58176100	0.23825600
C	-0.98925000	-5.19484300	-0.56441300	C	3.81015100	-3.58596100	-0.54666000
C	-0.83434400	-3.08210700	0.55164300	C	2.46138100	-2.47002300	1.07811000
C	0.39485000	-5.23012900	-0.68286900	C	4.68951200	-2.50927300	-0.55845200
H	-1.57703800	-6.01067100	-0.97397300	H	4.00900100	-4.43701300	-1.19102500
C	0.54565900	-3.13084800	0.45585700	C	3.31735900	-1.38386500	1.04600000
H	-1.30161600	-2.22495700	1.02510800	H	1.59913900	-2.44466800	1.73641500
C	1.20348000	-4.19769300	-0.17812800	C	4.44221000	-1.35686300	0.20410100
H	0.85974100	-6.06861900	-1.19159500	H	5.55602700	-2.53960300	-1.21242600
H	1.12988700	-2.31644000	0.86942300	H	3.10060000	-0.52171800	1.66925500
C	-4.09996800	-5.16431400	0.63668100	C	2.01034800	-6.20329600	-0.01147500
C	-3.56741600	-6.30845600	1.25395400	C	3.14082600	-6.66845400	0.68040100
C	-5.48909300	-5.12820100	0.42956100	C	1.34426800	-7.13012700	-0.83005600
C	-4.37914400	-7.36258100	1.64783600	C	3.56729400	-7.98663000	0.58597200
H	-2.49991900	-6.36201900	1.43881600	H	3.69150800	-5.97891100	1.31261800
C	-6.30543900	-6.18524000	0.80556400	C	1.77507500	-8.44551400	-0.94734900
H	-5.92902800	-4.25709900	-0.04439300	H	0.47939600	-6.80499700	-1.39902000
C	-5.74983900	-7.30326400	1.41875900	C	2.88519100	-8.88071600	-0.23213600
H	-3.94667800	-8.23133500	2.13211900	H	4.43650300	-8.31702000	1.14535900
H	-7.37395800	-6.13935300	0.62539900	H	1.24451100	-9.13343700	-1.59766000
H	-6.38601800	-8.12874000	1.72004700	H	3.22041400	-9.90915400	-0.31623500
B	0.21623700	5.00514900	0.29076500	B	-4.12473700	2.96443500	-0.09873600
C	1.52793300	4.14472000	0.33066000	C	-2.65289700	3.47101300	0.00241200
C	1.51895300	2.87081200	0.92139300	C	-1.68286800	2.73776800	0.70622200
C	2.71580800	4.54771200	-0.29845200	C	-2.22315300	4.63009600	-0.66533000
C	2.61395600	2.02868100	0.84040200	C	-0.35995500	3.14374500	0.74453500
H	0.62352300	2.52575400	1.42762600	H	-1.96458100	1.82907300	1.22814900
C	3.81523800	3.70063000	-0.38147400	C	-0.89564200	5.03438500	-0.62422100
H	2.76624600	5.52768200	-0.76274400	H	-2.93402800	5.20941200	-1.24452300
C	3.78490100	2.40760700	0.16304200	C	0.07369200	4.29815000	0.07361400
H	2.55478500	1.04222700	1.28780700	H	0.35958900	2.54409900	1.29102300
H	4.70227500	4.03523000	-0.91024200	H	-0.59947600	5.92919500	-1.16193100
C	0.25483800	6.55396500	0.46220000	C	-5.36397200	3.88489100	-0.24734400
C	-0.73625200	7.38007800	-0.09337500	C	-6.58020100	3.38930600	-0.74892700
C	1.28840100	7.17912300	1.17938300	C	-5.32655800	5.23004600	0.15790500
C	-0.69463500	8.75979100	0.05066200	C	-7.69456400	4.20414600	-0.87408900
H	-1.54399300	6.92976000	-0.66050500	H	-6.65032800	2.35041900	-1.05505200
C	1.32421300	8.55619500	1.34787000	C	-6.44510300	6.04383600	0.05580900
H	2.06883300	6.56911500	1.62196000	H	-4.41264600	5.63480600	0.57760600
C	0.33378900	9.34908500	0.77815200	C	-7.62742500	5.53334800	-0.46913300
H	-1.46428400	9.37714600	-0.39976600	H	-8.61833600	3.80457300	-1.27698700
H	2.12534800	9.01393800	1.91794800	H	-6.39747500	7.07582300	0.38462400
H	0.36400800	10.42654800	0.90034700	H	-8.50096200	6.17025400	-0.55536100
C	3.46312000	-2.77389100	-0.40982300	C	4.39773700	1.28263800	-0.02839900
C	2.85878600	-1.70685800	-1.09487900	C	3.18188800	1.26517900	-0.73057500
C	4.64363000	-2.49148500	0.29408200	C	4.73292100	2.46940700	0.63938700
C	3.36374100	-0.42067000	-1.01907000	C	2.33134700	2.35632300	-0.73271300
H	1.95414900	-1.88641100	-1.66643900	H	2.89117100	0.36669100	-1.26424500
C	5.15403300	-1.20005100	0.36624700	C	3.87519400	3.56339100	0.64365800
H	5.14817300	-3.29054100	0.82811200	H	5.66569900	2.52526400	1.19210200
C	4.50790900	-0.12392700	-0.25984300	C	2.64099300	3.53007100	-0.02606800
H	2.84375000	0.38189700	-1.53166400	H	1.39404700	2.29246600	-1.27529500
H	6.04758700	-1.01579900	0.95452300	H	4.15238900	4.45058900	1.20416000
B	4.94252900	1.36708600	-0.03532600	B	1.59888500	4.69186400	0.06402300
C	6.44267100	1.78619100	0.00281400	C	2.03410200	6.18612700	0.14837700
C	7.42712600	1.02848500	-0.65310400	C	3.24701200	6.62419200	-0.40907900
C	6.86531800	2.93371800	0.69414300	C	1.22912300	7.14915900	0.77952100
C	8.76337900	1.40253600	-0.63242500	C	3.63271400	7.95602200	-0.34991000
H	7.13298900	0.13787100	-1.19838800	H	3.88891800	5.90739000	-0.90989400
C	8.20254800	3.30226100	0.73783600	C	1.62007900	8.47792400	0.86508400
H	6.13080500	3.53703800	1.21702300	H	0.28701700	6.84434000	1.22309500
C	9.15306600	2.53876700	0.06858200	C	2.82147400	8.88395400	0.29411300
H	9.50309800	0.80809500	-1.15759200	H	4.56720100	8.27176800	-0.80071700

H	8.50563500	4.18554900	1.28946600	H	0.98818500	9.19952700	1.37137500
H	10.19800600	2.82902100	0.09387600	H	3.12529000	9.92375200	0.35056600
C	3.60623400	-5.49113800	-0.33571500	C	6.80698400	-0.01406100	-0.11487800
C	3.17413300	-6.65210000	0.32642500	C	7.59326400	-1.07747100	0.35871500
C	4.83733500	-5.55389200	-1.00927100	C	7.47498900	1.06384800	-0.71903900
C	3.93380100	-7.81379500	0.32368800	C	8.97621500	-1.06353700	0.24404000
H	2.23324600	-6.63538500	0.86614200	H	7.10646200	-1.92219800	0.83441300
C	5.59038600	-6.71941200	-1.03827700	C	8.85611000	1.07738500	-0.85352400
H	5.20123600	-4.67336000	-1.52840200	H	6.89589300	1.89827800	-1.10054100
C	5.14085300	-7.85070200	-0.36596600	C	9.60893300	0.01374000	-0.36711300
H	3.58509300	-8.69252800	0.85536700	H	9.56224900	-1.89204200	0.62680500
H	6.53055700	-6.74601200	-1.57842400	H	9.34829400	1.91579700	-1.33448600
H	5.73194800	-8.76024700	-0.37761400	H	10.68934000	0.02435200	-0.46476400
B	-4.75996600	1.74919100	-0.46802400	B	-4.14558800	-2.96533000	0.14020600
B	-3.19789000	-3.97446500	0.19808600	B	1.54559700	-4.70629600	0.11304700
B	2.76522000	-4.17685000	-0.32361400	B	5.25631800	-0.03509100	0.03119800

Structure S7. The coordinates of both the optimized structures of B4N1L$_1$ at S$_0$ and S$_1$ in toluene.

S_0-optimized structure				S_1-optimized structure			
C	2.64461000	-3.43627900	-0.07287800	C	-2.68375300	-3.34520100	0.00298700
C	2.31524700	-4.69106000	0.46091600	C	-2.38114300	-4.55395900	-0.63388700
C	1.60430700	-2.61443400	-0.52534400	C	-1.65947500	-2.56073200	0.54474100
C	0.99215200	-5.09254000	0.53704300	C	-1.05897600	-4.95616900	-0.72787700
H	3.10010900	-5.33717300	0.83595300	H	-3.17479800	-5.14679400	-1.07304600
C	0.29073900	-3.02595200	-0.42176200	C	-0.35011900	-2.98703000	0.44315700
H	1.83468200	-1.64799000	-0.95609600	H	-1.90219500	-1.64152700	1.06367400
C	-0.07263200	-4.27474400	0.11381400	C	-0.00584200	-4.18831500	-0.19853000
H	0.77454100	-6.05929700	0.97918700	H	-0.83364600	-5.88146600	-1.24603900
H	-0.48300600	-2.36006800	-0.78633400	H	0.42763200	-2.37474700	0.88243700
C	4.16032300	-1.56690400	0.00796200	C	-4.23480900	-1.52422500	-0.09026900
C	4.55087700	-0.78964000	-1.07880900	C	-4.93285300	-0.75494400	0.85762000
C	3.89153700	-0.94920200	1.22651900	C	-3.67830000	-0.90604800	-1.22172400
C	4.60227200	0.59377600	-0.95910400	C	-4.95123600	0.61783100	0.72456100
H	4.77505700	-1.26962000	-2.02504200	H	-5.35408000	-1.23547300	1.73287000
C	3.95055600	0.43256100	1.33640800	C	-3.78972500	0.45803400	-1.37189300
H	3.61077000	-1.55887300	2.07817700	H	-3.18501400	-1.51324000	-1.97172900
C	4.27184800	1.24184200	0.23878000	C	-4.34582600	1.27858200	-0.36583000
H	4.86785100	1.18000900	-1.83358900	H	-5.39427000	1.20841700	1.51884700
H	3.72780900	0.88587900	2.29734300	H	-3.38272900	0.92013500	-2.26403600
C	5.06598000	-3.80273900	-0.38587600	C	-5.03753200	-3.72631300	0.54523900
C	6.32424900	-3.47146300	0.12700100	C	-6.35287200	-3.47063300	0.12601600
C	4.94266200	-4.94725700	-1.18116900	C	-4.76794800	-4.81673100	1.38723600
C	7.42577500	-4.26885400	-0.14725500	C	-7.37778600	-4.29551800	0.55004300
H	6.43393600	-2.58683800	0.74272100	H	-6.54943100	-2.64921800	-0.55134700
C	6.04835000	-5.74521800	-1.43489200	C	-5.80462000	-5.63342300	1.79671700
H	3.98072900	-5.20460900	-1.60731800	H	-3.75869200	-4.99087300	1.73746600
C	7.29778900	-5.41422600	-0.92342900	C	-7.11056300	-5.37863500	1.38317300
H	8.39191600	-3.99326200	0.26132800	H	-8.38974700	-4.10300400	0.21538700
H	5.93065500	-6.62755400	-2.05458800	H	-5.59666500	-6.46582700	2.45795100
H	8.15960300	-6.03792300	-1.13053100	H	-7.91752600	-6.02330400	1.70960800
C	2.69929700	3.41107600	0.24240900	C	-2.72556700	3.36158500	-0.14669500
C	2.39023000	4.56925200	-0.48991900	C	-2.39841900	4.67948200	0.29237400
C	1.62689500	2.74642900	0.85690600	C	-1.59586900	2.50973900	-0.30568000
C	1.08335000	5.02769800	-0.60821600	C	-1.10318700	5.09639200	0.50201800
H	3.18298600	5.10447100	-1.00243300	H	-3.20383400	5.37321300	0.51086400
C	0.32783700	3.20961300	0.74919900	C	-0.30549800	2.93373600	-0.11687700
H	1.80863500	1.83429200	1.41334000	H	-1.74495300	1.48415600	-0.62239000
C	0.01276300	4.35585600	0.00122000	C	0.02422800	4.25206500	0.29552100
H	0.88630000	5.91157100	-1.20663300	H	-0.93929600	6.10463500	0.87029800
H	-0.46882200	2.65819800	1.23652000	H	0.49698600	2.23205000	-0.31426700
C	5.42245000	3.71067300	0.36897800	C	-5.40267000	3.76479200	-0.64330500
C	5.35735900	5.05766600	0.76492700	C	-5.27933000	5.06923200	-1.16310600
C	6.69358500	3.18346000	0.08462800	C	-6.71887500	3.30437500	-0.43897400
C	6.49811500	5.84210600	0.85701900	C	-6.38361700	5.86737100	-1.42303700
H	4.39698400	5.49000900	1.02254800	H	-4.29204400	5.45411700	-1.39124300
C	7.83619300	3.96638500	0.15419100	C	-7.82845200	4.09831700	-0.68930800
H	6.78257300	2.13966800	-0.19673600	H	-6.87877700	2.29616000	-0.07082300
C	7.73882000	5.29872900	0.54191200	C	-7.66788100	5.39042300	-1.17900300
H	6.42282600	6.87629500	1.17463800	H	-6.24315600	6.86516800	-1.82614700
H	8.80433800	3.53866300	-0.08208300	H	-8.82456000	3.70769800	-0.50659400
H	8.63177000	5.91117900	0.60737400	H	-8.53280700	6.01335500	-1.38002000
B	-1.57283400	-4.67223900	0.24543700	B	1.51161200	-4.61365000	-0.30509300
C	-2.62999800	-3.51240700	0.33713000	C	2.57647500	-3.47393600	-0.37878800
C	-2.35566900	-2.34270800	1.06449500	C	2.29833100	-2.25593200	-1.02208300
C	-3.83013900	-3.54201900	-0.38882700	C	3.80043100	-3.56718200	0.30408400
C	-3.18881200	-1.23951500	1.00171700	C	3.15893700	-1.17645300	-0.93031200
H	-1.45006800	-2.28798800	1.65971200	H	1.37461600	-2.14512900	-1.58098600
C	-4.67291500	-2.43721100	-0.44488900	C	4.66843800	-2.48404100	0.38932200
H	-4.08844800	-4.43364700	-0.95170700	H	4.05790000	-4.49217600	0.81020700
C	-4.35555100	-1.24319000	0.21958000	C	4.35487000	-1.25053800	-0.19749200
H	-2.91639200	-0.34046300	1.54488900	H	2.88816200	-0.24164200	-1.40944200
H	-5.57211300	-2.48695100	-1.05122600	H	5.58986800	-2.58738600	0.95405100
C	-2.03959200	-6.16430400	0.25328100	C	1.92094000	-6.11642800	-0.27937400
C	-1.34205900	-7.16447500	-0.44335600	C	1.15618500	-7.08622800	0.39044500
C	-3.19200600	-6.55441400	0.95480900	C	3.08527500	-6.55207100	-0.93430200
C	-1.77200300	-8.48464400	-0.44284100	C	1.53755400	-8.42019600	0.41528700
H	-0.45692500	-6.89599700	-1.01030100	H	0.25729900	-6.78428300	0.91798200
C	-3.61456000	-7.87641600	0.98104100	C	3.45822200	-7.88871200	-0.93674600
H	-3.75908600	-5.80494500	1.49726100	H	3.69827800	-5.82806500	-1.46031600
C	-2.90594500	-8.84408800	0.27722900	C	2.68671100	-8.82415500	-0.25599500
H	-1.22284000	-9.23518500	-1.00121200	H	0.93964700	-9.14737400	0.95377100
H	-4.49998600	-8.15304600	1.54328600	H	4.35330400	-8.20193700	-1.46274100
H	-3.23940200	-9.87632900	0.28661300	H	2.98191000	-9.86779700	-0.24684800
C	-2.54865100	3.63296200	-0.25215400	C	2.60019400	3.56485000	0.55486300
C	-2.23646300	2.42454200	-0.89603000	C	2.39523400	2.36202900	1.25516100
C	-3.79136400	3.70727700	0.39582700	C	3.79377200	3.65899800	-0.17979000
C	-3.08973600	1.33667300	-0.84478800	C	3.25105600	1.28454100	1.11974500
H	-1.29123800	2.32984500	-1.42007800	H	1.51244800	2.26047000	1.87818300
C	-4.64971200	2.61436500	0.44574100	C	4.67385800	2.58909100	-0.29206500
H	-4.07374100	4.62538900	0.90153900	H	4.01214800	4.57890400	-0.71379600
C	-4.30900100	1.39043500	-0.14938600	C	4.40200400	1.35100400	0.31395900
H	-2.79558400	0.41202500	-1.32994000	H	3.01565400	0.35604600	1.63122200
H	-5.58565400	2.70070200	0.98896200	H	5.55913700	2.69530800	-0.91214100
B	-5.16503700	0.08426500	0.01362700	B	5.21352300	0.05754200	-0.00077300
C	-6.72129600	0.10330600	-0.04166300	C	6.76388800	0.04325700	-0.15367800
C	-7.41835500	1.13188900	-0.69709600	C	7.56022300	1.04195800	0.43112700
C	-7.48340900	-0.91101400	0.56158300	C	7.42213700	-0.96881500	-0.87171600
C	-8.80469000	1.14377500	-0.75736200	C	8.94276900	1.02958100	0.31116500
H	-6.85878200	1.92686000	-1.17859100	H	7.08160800	1.83304700	0.99840600
C	-8.87047400	-0.89529100	0.52340600	C	8.80277700	-0.97885000	-1.01112700
H	-6.97465700	-1.71765900	1.07877300	H	6.83551700	-1.75360400	-1.33793000
C	-9.53267200	0.13122000	-0.14156500	C	9.56546600	0.02021600	-0.41534700
H	-9.31989400	1.94219400	-1.28026500	H	9.53631600	1.80681800	0.78018600

H	-9.43710800	-1.68348300	1.00713300	H	9.28704600	-1.76476300	-1.58068300
H	-10.61676900	0.14189400	-0.18019800	H	10.64554100	0.01167600	-0.51677700
C	-1.90497200	6.28435300	-0.25632300	C	1.93045200	6.19124000	0.54954600
C	-1.13353700	7.29464400	0.34164900	C	1.29147400	7.19680700	-0.19566500
C	-3.07419300	6.67775800	-0.92836400	C	3.01992500	6.59170900	1.34192600
C	-1.51353200	8.62812000	0.28208000	C	1.70641400	8.52149500	-0.14737200
H	-0.22835900	7.02424900	0.87503200	H	0.46199100	6.92745600	-0.84106500
C	-3.44814100	8.01158100	-1.01285300	C	3.43216100	7.91592400	1.40997100
H	-3.69114600	5.92189100	-1.40276600	H	3.54984300	5.84340000	1.92321700
C	-2.66963200	8.98863800	-0.40176500	C	2.77578900	8.88798600	0.66252400
H	-0.90847200	9.38827400	0.76421000	H	1.19625500	9.27049400	-0.74452600
H	-4.34810400	8.29118000	-1.54972900	H	4.26990600	8.19133700	2.04258700
H	-2.96439700	10.03111200	-0.45776600	H	3.09917100	9.92272800	0.70640300
B	4.15134700	2.81400900	0.29777900	B	-4.15234800	2.85930000	-0.38764300
B	-1.48682600	4.78481700	-0.17791500	B	1.48693500	4.68414400	0.48744200
N	3.96384700	-2.97141700	-0.12641700	N	-4.00989400	-2.88588600	0.12336600

Structure S8. The coordinates of both the optimized structures of B3N2L$_1$ at S$_0$ and S$_1$ in toluene.

S_0-optimized structure				S_1-optimized structure			
C	0.97770000	-4.22574500	-0.24071200	C	2.95840700	3.23600800	0.19709700
C	0.21965900	-5.15938200	0.48555000	C	2.68774300	4.55342400	-0.26880100
C	0.27134600	-3.17985400	-0.85421700	C	1.79997700	2.49108800	0.54451900
C	-1.16123700	-5.04549600	0.59969200	C	1.40886200	5.05745900	-0.37887300
H	0.72291700	-5.97270500	0.99812000	H	3.51460300	5.17236400	-0.60197900
C	-1.10344400	-3.07257100	-0.74972900	C	0.52900700	2.99990700	0.45018100
H	0.81043400	-2.41795600	-1.40473200	H	1.91515300	1.47886800	0.91465400
C	-1.86172100	-3.99204800	-0.00674200	C	0.25300000	4.30346700	-0.03959400
H	-1.70340500	-5.77387400	1.19458500	H	1.27909400	6.05613900	-0.78507400
H	-1.60366000	-2.24091400	-1.23375100	H	-0.29910600	2.37823200	0.77182500
C	3.30121000	-2.88536900	-0.23738800	C	4.40295200	1.00962100	0.23164600
C	3.31505300	-2.00653300	-1.32853000	C	3.99020000	0.25558700	1.34056800
C	3.90124900	-2.43827200	0.94796000	C	4.71758800	0.29403100	-0.93452800
C	3.84005700	-0.72655300	-1.22487800	C	3.78719300	-1.11381000	1.26208500
H	2.90203400	-2.32062100	-2.28213400	H	3.77538800	0.75916000	2.27733500
C	4.43198600	-1.15929900	1.06180700	C	4.55871800	-1.08237600	-1.02403800
H	3.92274900	-3.08705400	1.81845300	H	5.04593400	0.83307200	-1.81740200
C	4.37552400	-0.28119500	-0.01861600	C	4.04701100	-1.77757100	0.06682100
H	3.81999200	-0.05286700	-2.07435700	H	3.41269900	-1.67076200	2.11429700
H	4.86459200	-0.82500200	1.99848100	H	4.76761300	-1.60745600	-1.94969400
C	3.34149600	-5.61014800	-0.34737400	C	5.70158300	3.40606300	0.23850100
C	4.71469900	-5.65081200	-0.05210600	C	6.91162800	2.83348100	-0.19564000
C	2.73285500	-6.81314300	-0.74458500	C	5.77940100	4.71882700	0.74101000
C	5.43534200	-6.83434200	-0.11074400	C	8.10871300	3.53441800	-0.17566300
H	5.22315000	-4.73481600	0.22858100	H	6.91388200	1.81095400	-0.55879700
C	3.45133000	-7.99740200	-0.82625700	C	6.97473800	5.42316800	0.77809200
H	1.68206800	-6.81385600	-1.01173700	H	4.88391600	5.18685800	1.13390900
C	4.80278100	-8.01074600	-0.49909600	C	8.14610100	4.83778300	0.30934300
H	6.49181600	-6.84088000	0.13405300	H	9.01856200	3.06190800	-0.53178100
H	2.96021500	-8.91031300	-1.14491900	H	6.99446200	6.43149700	1.17869700
H	5.36582700	-8.93620100	-0.55622800	H	9.08119400	5.38715400	0.33352600
C	3.76713500	2.04664300	0.05419400	C	2.37101300	-3.48838700	-0.02726500
C	3.85337400	3.16225700	-0.78168500	C	1.90919200	-4.75466500	0.40770300
C	2.61158500	1.87435200	0.81745800	C	1.40582900	-2.53219900	-0.42446500
C	2.79843000	4.05809700	-0.87786900	C	0.56954800	-5.03746100	0.45640700
H	4.74237900	3.31132800	-1.38382900	H	2.61978500	-5.48767300	0.76681200
C	1.56443100	2.77389800	0.73220900	C	0.07124800	-2.82265000	-0.39711700
H	2.53575500	1.02395100	1.48464400	H	1.73270000	-1.57250000	-0.80077700
C	1.63155200	3.86611700	-0.13463100	C	-0.39546500	-4.07897500	0.05952000
H	2.86894000	4.90003600	-1.55671200	H	0.24403800	-5.98970400	0.85363100
H	0.67778800	2.62337300	1.33609900	H	-0.63441100	-2.08819400	-0.75915400
C	6.12271700	1.39551800	0.37965500	C	4.72770700	-4.11285400	-0.20862400
C	6.46727500	2.61142800	0.98448500	C	4.56201900	-5.20656000	-1.06378200
C	7.14837200	0.49738400	0.05575300	C	5.93847000	-3.93333900	0.46489600
C	7.79552400	2.91585600	1.24260900	C	5.59067500	-6.12272100	-1.21667900
H	5.69065700	3.31458500	1.25867700	H	3.64159000	-5.31944000	-1.62375800
C	8.47071000	0.81051500	0.33183500	C	6.96074100	-4.85331600	0.29790300
H	6.90397400	-0.44543100	-0.41821000	H	6.06638400	-3.07993300	1.11904600
C	8.81027200	2.02174200	0.92277900	C	6.79162900	-5.95280700	-0.53698700
H	8.03471000	3.86375200	1.71296200	H	5.45864200	-6.96336300	-1.88780700
H	9.24512500	0.09750600	0.06966900	H	7.89502700	-4.71095900	0.82806100
H	9.84586400	2.26373800	1.13118300	H	7.59539400	-6.66802500	-0.66517400
B	-3.40396900	-3.75653200	0.17330900	B	-1.19797400	4.80315600	-0.20231800
C	-3.86826300	-2.26219200	0.27393000	C	-2.34318100	3.73084900	-0.29890600
C	-3.07303000	-1.32587200	0.95426000	C	-2.11948700	2.49532100	-0.94125100
C	-4.99856000	-1.77044600	-0.39686100	C	-3.60846300	3.88608300	0.30237400
C	-3.35323100	0.02800800	0.91010700	C	-3.02764100	1.45987900	-0.87539400
H	-2.19827700	-1.66649600	1.49830900	H	-1.18677800	2.34442900	-1.47415600
C	-5.27859500	-0.40867500	-0.44250800	C	-4.53768100	2.85695800	0.34403800
H	-5.64534400	-2.46213200	-0.92780100	H	-3.84422900	4.82620400	0.79199800
C	-4.44342100	0.53031300	0.18067100	C	-4.26140000	1.58800500	-0.20422600
H	-2.69428900	0.72035000	1.42324800	H	-2.78212700	0.51670500	-1.35399600
H	-6.13997600	-0.06390300	-1.00635600	H	-5.47523800	3.01541700	0.86877800
C	-4.41770200	-4.93846000	0.22251200	C	-1.55954700	6.33213900	-0.24475900
C	-4.13173400	-6.17752900	-0.37438300	C	-0.82801600	7.29432500	0.47373700
C	-5.65840500	-4.80347000	0.86745300	C	-2.64942800	6.80731400	-0.99548500
C	-5.04002300	-7.22617700	-0.33839400	C	-1.15803600	8.64289100	0.44402100
H	-3.18535900	-6.31219800	-0.88739200	H	0.01066400	6.96995600	1.08122900
C	-6.56264400	-5.85452700	0.92709000	C	-2.98095300	8.15498800	-1.04118400
H	-5.90850500	-3.85952400	1.34018200	H	-3.24361900	6.09870500	-1.56421900
C	-6.25544300	-7.06684400	0.31866800	C	-2.23561900	9.08085000	-0.31871300
H	-4.80171600	-8.16909700	-0.81849100	H	-0.57529100	9.35538900	1.01923900
H	-7.50877900	-5.72968300	1.44240900	H	-3.82314500	8.48573700	-1.64074100
H	-6.96390300	-7.88754600	0.35570800	H	-2.49411600	10.13420500	-0.34743200
C	-0.76565200	4.18545000	-0.29940700	C	-2.67401300	-3.27089900	0.18029500
C	-0.97940100	2.96386300	-0.95089600	C	-2.46976900	-2.21849700	1.07260800
C	-1.84914400	4.79672100	0.34282500	C	-3.77032800	-3.25009400	-0.68051500
C	-2.20388900	2.33316200	-0.86658600	C	-3.29520200	-1.10942800	1.01811100
H	-0.16293900	2.49605100	-1.48743400	H	-1.64431600	-2.25682400	1.77425300
C	-3.08645200	4.17073700	0.37297400	C	-4.61508800	-2.14768300	-0.68559000
H	-1.70942300	5.74562800	0.84692500	H	-3.93710300	-4.07525300	-1.36313200
C	-3.29879200	2.90117700	-0.18977900	C	-4.37307900	-1.02883400	0.12319100
H	-2.31956800	1.36661500	-1.34465000	H	-3.08574100	-0.27229500	1.67445800
H	-3.89674800	4.65806800	0.90571200	H	-5.45150800	-2.13656600	-1.37655100
C	-6.01673600	2.76016500	0.06348200	C	-6.71359300	0.37810000	0.06503500
C	-6.26534300	3.98137600	-0.58436500	C	-7.44300200	-0.66126700	0.66721000
C	-7.08715900	2.16667300	0.75248500	C	-7.44791000	1.44375500	-0.48324000
C	-7.51834000	4.57776500	-0.55291500	C	-8.82848600	-0.63257500	0.73695100
H	-5.46249700	4.46070500	-1.13460200	H	-6.90796200	-1.50132600	1.09855400
C	-8.33749300	2.76678200	0.80637700	C	-8.83424600	1.47313600	-0.43053600
H	-6.92820700	1.22285200	1.26357600	H	-6.91702900	2.25372100	-0.97156700
C	-8.55510000	3.97269000	0.14868200	C	-9.52742800	0.43606500	0.18502700
H	-7.68801800	5.51488500	-1.07210600	H	-9.36649000	-1.44239400	1.21816900
H	-9.14448600	2.29422800	1.35586400	H	-9.37674100	2.30375200	-0.86906400

H	-9.53403100	4.43987200	0.18140100	H	-10.61101700	0.45903400	0.23176300
C	0.72134900	6.13362600	-0.26185900	C	-2.23911100	-5.66923900	0.11770400
C	1.68251800	6.71355300	0.56972200	C	-1.77569700	-6.58863800	-0.82411800
C	-0.03650800	6.95546600	-1.10038800	C	-3.22284800	-6.03901400	1.03475400
C	1.88189300	8.08655600	0.55514400	C	-2.28386500	-7.87924700	-0.83084300
H	2.26988500	6.08382300	1.22735100	H	-1.03610100	-6.28352400	-1.55489100
C	0.15775000	8.32892600	-1.09405700	C	-3.72717200	-7.33004100	1.01475600
H	-0.77492200	6.51116600	-1.75738600	H	-3.57931700	-5.31700400	1.75940800
C	1.11961900	8.90379300	-0.27120100	C	-3.25841400	-8.25402900	0.08683300
H	2.63205800	8.52019100	1.20730600	H	-1.92644900	-8.58931200	-1.56739900
H	-0.43939500	8.95195300	-1.75083700	H	-4.48668800	-7.61663400	1.73247300
H	1.27435900	9.97654400	-0.27515100	H	-3.65652400	-9.26176400	0.07499800
B	2.54760000	-4.27057700	-0.28892800	B	4.35741900	2.60136800	0.23675700
N	0.52438900	4.73757700	-0.26521200	N	-1.73911800	-4.34211800	0.12877500
N	4.79192100	1.07284500	0.10635000	N	3.70050400	-3.16076700	-0.05180500
B	-4.61194000	2.08388900	0.01275900	B	-5.15858900	0.33882500	-0.01205700

Structure S9. The coordinates of both the optimized structures of B2N3L$_1$ at S$_0$ and S$_1$ in toluene.

S$_0$-optimized structure				S$_1$-optimized structure			
C	1.52550000	-3.94351500	0.03011400	C	3.23813300	-2.78217300	-0.05744700
C	0.84917300	-4.89196700	0.80145400	C	3.06039200	-4.04476500	0.54636700
C	0.77180100	-2.99903500	-0.66694000	C	2.10619700	-2.15425500	-0.61682800
C	-0.53505500	-4.88393500	0.88512500	C	1.81345300	-4.62141800	0.62546500
H	1.41580800	-5.62796900	1.36045500	H	3.90401800	-4.54106200	1.00860100
C	-0.60964900	-2.99430800	-0.59110800	C	0.86956700	-2.74333400	-0.56281200
H	1.27525300	-2.26005100	-1.27879300	H	2.22131900	-1.20649800	-1.12535500
C	-1.28780100	-3.92869400	0.19459700	C	0.68318800	-3.97780400	0.08683300
H	-1.03713000	-5.61268200	1.51068100	H	1.69615400	-5.56049500	1.15150500
H	-1.17095700	-2.25463000	-1.14877100	H	0.03222000	-2.25691300	-1.04580900
C	3.53114500	-2.61150800	0.09409200	C	4.42181800	-0.70159500	-0.04994700
C	4.13857600	-1.99124900	-0.99611400	C	4.68411200	0.04670100	-1.19199600
C	3.44388000	-1.92267300	1.30155900	C	4.02009200	-0.06552100	1.12034100
C	4.58351900	-0.67932300	-0.89169200	C	4.45554700	1.41854600	-1.17566300
H	4.22856500	-2.52877700	-1.93377400	H	5.00539700	-0.45262100	-2.09990500
C	3.89401800	-0.61374400	1.39561800	C	3.82761700	1.30808600	1.12394100
H	2.99783700	-2.41497100	2.15858500	H	3.83326900	-0.65555200	2.01144000
C	4.44183000	0.05571500	0.29345700	C	3.98582400	2.08331100	-0.03393600
H	5.01270800	-0.20679000	-1.77014400	H	4.59780800	1.97587800	-2.09638700
H	3.80542500	-0.10374300	2.34985400	H	3.49046700	1.78446500	2.03908100
C	3.70981600	-5.00959100	-0.37614000	C	5.69217300	-2.80285800	-0.10447500
C	5.07735200	-5.04104000	-0.07176600	C	6.80168900	-2.23190200	0.52521600
C	3.14876700	-6.10922800	-1.03796200	C	5.84052700	-4.01434000	-0.78833900
C	5.85139200	-6.13672700	-0.42196800	C	8.02928400	-2.87455400	0.48427000
H	5.52904900	-4.20314100	0.44531500	H	6.69524800	-1.28819700	1.04544900
C	3.93298900	-7.20418800	-1.37008700	C	7.07107700	-4.65228900	-0.81203100
H	2.09763400	-6.10124400	-1.29803800	H	4.99748000	-4.44151600	-1.31798000
C	5.28941500	-7.23083300	-1.06948200	C	8.17189500	-4.08933000	-0.17642200
H	6.90737500	-6.13528600	-0.17350200	H	8.88015200	-2.42101300	0.97965000
H	3.47354000	-8.04220100	-1.88349200	H	7.17265600	-5.58828900	-1.34970200
H	5.89742400	-8.08757000	-1.33561700	H	9.13358700	-4.58791700	-0.20406500
C	3.52694600	2.56955000	0.26239600	C	1.98647100	3.82957700	-0.07920400
C	3.52690100	3.74970400	-0.50005000	C	1.34893300	5.07828200	-0.34932600
C	2.32355600	2.23035900	0.89907000	C	1.07798500	2.75057900	0.12351200
C	2.38455200	4.53158200	-0.63205500	C	-0.01766300	5.23402400	-0.35884800
H	4.42783900	4.04390200	-1.02874200	H	1.96485600	5.93801100	-0.59260600
C	1.19106200	3.01437600	0.77724800	C	-0.28341600	2.90632100	0.11472100
H	2.26250800	1.31895000	1.48184200	H	1.47716100	1.76076000	0.31497800
C	1.17863700	4.17796000	-0.00891600	C	-0.92195100	4.15862800	-0.11259800
H	2.42215400	5.42042500	-1.25431300	H	-0.42428100	6.21127000	-0.60132800
H	0.28186700	2.70542500	1.28134800	H	-0.90006900	2.03608500	0.31258400
C	6.23127700	2.11630600	0.35547400	C	4.58045900	4.73870500	-0.00703500
C	6.54231400	3.44034800	0.70944700	C	4.33714500	5.98682000	0.59620600
C	7.30585600	1.25403400	0.07954500	C	5.87681900	4.53860500	-0.51631200
C	7.85508600	3.88582400	0.76861900	C	5.30848300	6.97681100	0.65960800
H	5.74010600	4.12539700	0.96005100	H	3.37030900	6.17427500	1.04960000
C	8.61976600	1.69655300	0.11689800	C	6.84998800	5.52673700	-0.47093100
H	7.10209200	0.21800800	-0.16874400	H	6.12802600	3.58075800	-0.96061900
C	8.89554700	3.01545400	0.46241400	C	6.56952000	6.75626500	0.11623200
H	8.06951600	4.91007100	1.05337000	H	5.08274900	7.92344400	1.14018700
H	9.43084000	1.01392900	-0.11179100	H	7.83408400	5.33636400	-0.88749400
H	9.92256300	3.36240900	0.50258900	H	7.32927400	7.52939700	0.16076500
C	-3.30641500	-2.59979700	0.29157600	C	-1.71619200	-3.71004100	0.24425300
C	-2.84298500	-1.60251900	1.15244200	C	-1.66657900	-2.46697400	0.89616900
C	-4.32830400	-2.28890700	-0.60774000	C	-2.91106000	-4.08563000	-0.39051100
C	-3.33816200	-0.31422000	1.06792100	C	-2.72773200	-1.59907800	0.83119100
H	-2.07276300	-1.84120500	1.87630300	H	-0.78437500	-2.19054400	1.46008000
C	-4.82303400	-0.99520100	-0.69382600	C	-3.98375000	-3.22192700	-0.44217300
H	-4.70493300	-3.05439600	-1.27612600	H	-2.97157400	-5.03917400	-0.90038300
C	-4.30778100	0.01436800	0.11859300	C	-3.90047000	-1.93616000	0.12643600
H	-2.95613800	0.45696400	1.72650000	H	-2.66785600	-0.64262000	1.33352400
H	-5.58224400	-0.75601000	-1.42951300	H	-4.87158600	-3.51068500	-0.99009000
C	-3.48080400	-5.03875300	0.27352800	C	-0.74943900	-5.93950500	0.23782700
C	-3.08775000	-6.15941000	-0.46497500	C	-0.08911900	-6.74447600	-0.69105100
C	-4.68435100	-5.08851800	0.98476900	C	-1.57870500	-6.51409800	1.20155000
C	-3.87660800	-7.30066400	-0.47834600	C	-0.25655000	-8.12025000	-0.64737100
H	-2.16443700	-6.13150700	-1.03084900	H	0.53721800	-6.28756200	-1.44783500
C	-5.47337600	-6.22845200	0.94987500	C	-1.74250600	-7.89053800	1.23210800
H	-4.99490500	-4.22915800	1.56722600	H	-2.07742100	-5.88138500	1.92604100
C	-5.07535200	-7.34494000	0.22355400	C	-1.08235900	-8.69702400	0.31136600
H	-3.55382500	-8.15888100	-1.05775600	H	0.25119400	-8.74282700	-1.37468800
H	-6.40252000	-6.24577400	1.50910000	H	-2.38129100	-8.33508100	1.98621700
H	-5.69069400	-8.23694400	0.20497900	H	-1.21263500	-9.77236200	0.33992200
C	-1.46505300	4.09833400	-0.30476900	C	-3.28959500	2.94058000	-0.19197200
C	-1.42408200	2.82211000	-0.89436000	C	-2.98777600	1.97235900	-1.16284600
C	-2.68667700	4.46978700	0.28206000	C	-4.28002100	2.60142800	0.74311200
C	-2.50381100	1.96381700	-0.87037400	C	-3.56296200	0.71259900	-1.14759100
H	-0.51538600	2.49386600	-1.38698700	H	-2.25294900	2.20378200	-1.92662200
C	-3.76645100	3.60246200	0.35895000	C	-4.85240700	1.33504700	0.79001800
H	-2.77607000	5.44494200	0.75003900	H	-4.57002000	3.33095800	1.49263600
C	-3.67847900	2.31916900	-0.19430500	C	-4.46117000	0.37405500	-0.13793100
H	-2.43397400	0.99106000	-1.34145400	H	-3.29057800	-0.03053500	-1.88946400
H	-4.66767200	3.90284700	0.88031000	H	-5.56481400	1.07617100	1.56591400
C	-6.06091700	1.71671100	-0.04654500	C	-6.25340400	-1.30728700	-0.17364800
C	-6.53560300	2.74177300	-0.87068400	C	-7.07136800	-0.53461500	-1.00260300
C	-6.96657200	1.02616000	0.76402900	C	-6.81229100	-2.37392400	0.53795500
C	-7.88230400	3.07395000	-0.86769200	C	-8.41939800	-0.83554900	-1.12273500
H	-5.84613300	3.27227100	-1.51652000	H	-6.64487800	0.29698300	-1.54987800
C	-8.31411200	1.35516400	0.74554700	C	-8.15911800	-2.67241000	0.39913300
H	-6.60867100	0.23301800	1.40956300	H	-6.19335800	-2.95379900	1.21230100
C	-8.78212400	2.38308800	-0.06427100	C	-8.97127100	-1.90712300	-0.42998600
H	-8.23135700	3.87200100	-1.51385400	H	-9.04124100	-0.22819700	-1.77061100
H	-9.00099800	0.80796200	1.38191300	H	-8.57945800	-3.49947600	0.96031400
H	-9.83472800	2.64118300	-0.07103000	H	-10.02538000	-2.13838100	-0.52871800

C	-0.20478800	6.52070100	-0.25294300	C	-3.21816300	5.64029500	0.06485300
C	0.78729700	7.29594900	0.36960900	C	-2.69921200	6.72360700	0.79646900
C	-1.22789800	7.20592000	-0.92874900	C	-4.49554900	5.81943200	-0.49608800
C	0.75423300	8.68298600	0.33253300	C	-3.40122700	7.91198500	0.94773100
H	1.59076700	6.79801400	0.90223700	H	-1.73005100	6.62038700	1.27274800
C	-1.25652400	8.59234200	-0.98884200	C	-5.19975200	7.00823900	-0.36191500
H	-2.00493900	6.63637300	-1.42767500	H	-4.94053500	5.00454200	-1.05905800
C	-0.26676500	9.33332500	-0.35221100	C	-4.65432600	8.06281200	0.36294200
H	1.52544500	9.25895000	0.83255400	H	-2.97186300	8.72325800	1.52697200
H	-2.05067200	9.09719000	-1.52824600	H	-6.17808200	7.11362000	-0.82009700
H	-0.29043100	10.41722500	-0.39049000	H	-5.20342000	8.99167900	0.47610600
B	4.76192300	1.60048900	0.32095900	B	3.49860100	3.60183700	-0.05019400
B	-0.17055800	4.96158800	-0.20310300	B	-2.44727500	4.28260800	-0.09461900
N	2.93884500	-3.89863000	-0.02283100	N	4.45557300	-2.13346500	-0.08310900
N	-2.69412900	-3.87420300	0.30375900	N	-0.58685000	-4.53166600	0.20021300
N	-4.69861200	1.36747900	-0.04394100	N	-4.89227800	-0.98454800	-0.03187300

Structure S10. The coordinates of both the optimized structures of B1N4L$_1$ at S_0 and S_1 in toluene.

S_0-optimized structure				S_1-optimized structure			
C	3.05544300	-2.98005100	0.00444100	C	-3.97249600	-1.61148200	-0.09189500
C	2.79906500	-4.11628200	0.77566600	C	-4.19896400	-2.85538600	-0.71029500
C	2.00545700	-2.42995000	-0.73187300	C	-2.74296200	-1.41546400	0.56239800
C	1.52742600	-4.66778100	0.82746000	C	-3.22589900	-3.83468200	-0.71061100
H	3.59782200	-4.55461900	1.36311800	H	-5.12780200	-3.03052200	-1.23859400
C	0.73862100	-2.98380200	-0.68832700	C	-1.78537200	-2.39984000	0.58094900
H	2.18478400	-1.55510800	-1.34525300	H	-2.55919100	-0.48051800	1.07519800
C	0.47279600	-4.10202500	0.10455200	C	-1.99225000	-3.62054500	-0.07907500
H	1.34429200	-5.53118000	1.45633700	H	-3.40496200	-4.76312400	-1.23948700
H	-0.05874200	-2.53919400	-1.27131500	H	-0.86249500	-2.23138000	1.12158600
C	4.32251800	-0.93809200	0.11422300	C	-4.34160400	0.75207100	-0.18727100
C	4.60328700	-0.12128100	-0.97906200	C	-4.43192000	1.61031800	0.90960500
C	3.95155000	-0.35025900	1.32085100	C	-3.62904300	1.16056500	-1.31218100
C	4.43478300	1.25424100	-0.88084500	C	-3.74286400	2.81358500	0.89729400
H	4.91274300	-0.57218800	-1.91576600	H	-4.99392500	1.30369100	1.78571300
C	3.79395600	1.02590100	1.40976300	C	-2.97184900	2.37994000	-1.31472800
H	3.75905900	-0.98444400	2.17920900	H	-3.59433800	0.51193500	-2.18179200
C	3.99144600	1.86090300	0.30252200	C	-2.94648800	3.23246000	-0.19042800
H	4.60940300	1.86108800	-1.76435200	H	-3.77509700	3.43127200	1.78925600
H	3.48734600	1.45299900	2.35988000	H	-2.46790700	2.68307900	-2.22560500
C	5.50205300	-3.04637000	-0.30372100	C	-6.27563700	-0.75680400	-0.14717000
C	6.74532100	-2.49245100	0.03038400	C	-7.10033000	0.17989600	-0.77933900
C	5.47818600	-4.29278500	-0.94299400	C	-6.86271800	-1.84782900	0.50492100
C	7.91954300	-3.16661200	-0.26795500	C	-8.47677000	0.01637700	-0.76853800
H	6.78526000	-1.53186600	0.52946300	H	-6.65409800	1.03180700	-1.27713900
C	6.66091400	-4.96078400	-1.22408200	C	-8.23998700	-2.00581800	0.49768300
H	4.53076300	-4.73395700	-1.22647100	H	-6.23941600	-2.55907000	1.03317900
C	7.89209400	-4.40764100	-0.89338100	C	-9.05744600	-1.07816600	-0.13805500
H	8.86795900	-2.71493700	0.00293300	H	-9.09957800	0.75188300	-1.26532000
H	6.61389100	-5.92382900	-1.72139800	H	-8.67772500	-2.85453600	1.01166500
H	8.81256400	-4.93306200	-1.11959400	H	-10.13399200	-1.20254600	-0.13467000
C	2.06877700	3.71055200	0.24966400	C	-0.46401400	4.23842900	-0.16688600
C	1.54712000	4.85804700	-0.37640200	C	0.47088600	4.87260100	0.69342000
C	1.11665400	2.79573300	0.73293500	C	0.10605900	3.26606800	-1.02713500
C	0.18704700	5.06763200	-0.53723000	C	1.81824600	4.55461700	0.72219100
H	2.22743000	5.58812200	-0.80181000	H	0.10950900	5.60099400	1.41080300
C	-0.24110100	3.01076800	0.63141800	C	1.45038400	2.95576900	-1.03095200
H	1.44936800	1.88320300	1.21291100	H	-0.52746500	2.73305400	-1.72319200
C	-0.73747400	4.13955000	-0.03674300	C	2.32812500	3.57968000	-0.14056000
H	-0.16370500	5.94073700	-1.07409100	H	2.47904600	5.03918600	1.43379100
H	-0.93035700	2.28544100	1.04406800	H	1.83508800	2.20986800	-1.71914300
C	4.69091000	4.49881200	0.36298500	C	-2.59936800	5.88321500	0.32684100
C	4.41414800	5.79502400	0.82912500	C	-1.83415700	7.07702100	0.30687800
C	6.01581700	4.22223000	-0.01278400	C	-3.97751400	6.06920300	0.60337000
C	5.40117500	6.76854300	0.89972700	C	-2.37618000	8.32032600	0.58685700
H	3.41125400	6.03598800	1.16384600	H	-0.78586400	7.02445100	0.03458200
C	7.00309100	5.19574000	0.03189600	C	-4.52516700	7.30845600	0.88962700
H	6.27401700	3.22264700	-0.34574500	H	-4.63810900	5.20938000	0.57973800
C	6.69629500	6.47279300	0.48977200	C	-3.72881500	8.45242700	0.89630300
H	5.16251800	7.75791600	1.27471600	H	-1.74055300	9.20042600	0.55440200
H	8.01523700	4.95815100	-0.27712100	H	-5.58730100	7.38808700	1.10230400
H	7.46859300	7.23310400	0.53769500	H	-4.15535800	9.42469600	1.11758100
C	-1.90953500	-3.68409300	0.20004600	C	0.35574300	-4.14962800	-0.16552100
C	-1.84709900	-2.56116200	1.02793600	C	0.69544900	-3.02045000	-0.92946700
C	-3.01276700	-3.83146200	-0.64408400	C	1.36614600	-4.78404400	0.57543300
C	-2.82568500	-1.58612300	0.96839600	C	1.95971200	-2.48899700	-0.87173300
H	-1.01211400	-2.44805800	1.70916700	H	-0.05102500	-2.56264800	-1.56634700
C	-3.99574100	-2.85382500	-0.70141400	C	2.63770900	-4.25170400	0.63144000
H	-3.08042400	-4.69878600	-1.29043000	H	1.12932500	-5.66173700	1.16418600
C	-3.89924300	-1.70371200	0.08326300	C	2.94651600	-3.06261000	-0.05036000
H	-2.75567700	-0.71219000	1.60547200	H	2.20223400	-1.61491700	-1.46260400
H	-4.82469300	-2.96393400	-1.39122000	H	3.38375600	-4.71822600	1.26281200
C	-1.09456300	-5.99215300	0.18241800	C	-1.27322200	-5.95660900	-0.02495500
C	-0.28926400	-6.86555000	-0.55559000	C	-2.24847700	-6.40861400	0.86624600
C	-2.16547000	-6.51754400	0.91294500	C	-0.59286200	-6.87079500	-0.83190400
C	-0.54683500	-8.22869100	-0.55053500	C	-2.53896300	-7.76235200	0.94169300
H	0.53671900	-6.47106600	-1.13508600	H	-2.76473400	-5.69859800	1.50113500
C	-2.42409500	-7.88003200	0.89662900	C	-0.88383200	-8.22340200	-0.73964300
H	-2.79108200	-5.85181900	1.49563400	H	0.15089600	-6.51438800	-1.53467500
C	-1.61605100	-8.74726300	0.17037000	C	-1.85818300	-8.67507800	0.14346100
H	0.08964100	-8.88863700	-1.12999900	H	-3.29379600	-8.10641800	1.63933300
H	-3.25920700	-8.26666300	1.47063100	H	-0.35501000	-8.92626900	-1.37310800
H	-1.81699400	-9.81228300	0.16616300	H	-2.08635300	-9.73246600	0.20870400
C	-2.90941300	3.10862800	-0.26940300	C	3.92739700	1.79978600	0.05821400
C	-2.56863000	2.07676900	-1.14420400	C	3.03949600	1.02557000	0.82636300
C	-3.98756200	2.93063900	0.59675800	C	4.98361300	1.13929100	-0.59543100
C	-3.23523000	0.86658600	-1.09482200	C	3.15674600	-0.34302500	0.87567200
H	-1.75327100	2.21912100	-1.84379600	H	2.24960400	1.52005900	1.37672500
C	-4.66160500	1.71831500	0.64277000	C	5.09179500	-0.23812200	-0.55335900
H	-4.26796400	3.72957200	1.27355200	H	5.68442300	1.70874900	-1.19299200
C	-4.26529500	0.65739300	-0.17441300	C	4.15872200	-1.00451800	0.15306600
H	-2.94449300	0.06026700	-1.75769700	H	2.46280700	-0.91897500	1.47632700
H	-5.46740700	1.57631300	1.35353600	H	5.87587600	-0.73085400	-1.11654000
C	-6.20327800	-0.84216900	0.03239400	C	5.37031700	-3.12962100	0.26677400
C	-7.08867500	0.02704800	-0.61581300	C	6.34758200	-2.67563900	1.15604700
C	-6.72815800	-1.92211600	0.75126000	C	5.59842800	-4.29171300	-0.47510500
C	-8.45782300	-0.17802000	-0.53547900	C	7.53457600	-3.37819800	1.29625100
H	-6.69838800	0.86053500	-1.18739400	H	6.16709400	-1.78025700	1.73842900
C	-8.09904400	-2.12512100	0.81006100	C	6.78392100	-4.99292300	-0.31609100
H	-6.05783600	-2.59881400	1.26711600	H	4.85121500	-4.63189900	-1.18228500
C	-8.97637100	-1.25556300	0.17317800	C	7.75850600	-4.54031000	0.56628600
H	-9.12443500	0.50846900	-1.04625600	H	8.28461200	-3.01915800	1.99170300
H	-8.48283300	-2.96871100	1.37362600	H	6.95262700	-5.89106900	-0.89926600
H	-10.04687400	-1.41557700	0.22718600	H	8.68667000	-5.08743800	0.68230100

C	-2.73957800	5.54443500	-0.32096900	C	4.72213600	4.09051300	-0.28968100
C	-2.32984300	6.61322500	0.48080100	C	4.51570500	5.20107300	-1.11442500
C	-3.80444700	5.72890700	-1.20630500	C	5.95625600	3.94342700	0.35437600
C	-2.96384300	7.84324100	0.38109500	C	5.52703000	6.13203700	-1.29585500
H	-1.51762600	6.47481900	1.18446600	H	3.56019500	5.32601600	-1.60857100
C	-4.44304000	6.95787800	-1.28562900	C	6.96375000	4.87486900	0.15512900
H	-4.12631100	4.90461500	-1.83172600	H	6.11552800	3.10582700	1.02316500
C	-4.02489400	8.02433200	-0.49846000	C	6.75841900	5.97534700	-0.66950100
H	-2.63314200	8.66204000	1.01064400	H	5.34943300	6.98563000	-1.94065800
H	-5.26736900	7.08329700	-1.97901400	H	7.91221900	4.74650000	0.66524100
H	-4.52187600	8.98501700	-0.56765400	H	7.54564700	6.70579600	-0.81582600
B	3.59198200	3.38950100	0.32070800	B	-2.00415500	4.49663300	-0.04832000
N	4.32542900	-2.35621600	-0.00423800	N	-4.88572200	-0.56804900	-0.14139100
N	-0.84183800	-4.60946900	0.19180900	N	-0.97037400	-4.57914800	-0.11228000
N	-4.81978800	-0.63532800	-0.04274500	N	4.16741200	-2.41274300	0.11058200
N	-2.11534100	4.28406200	-0.22666100	N	3.68939200	3.16109100	-0.08552400

Structure S11. The coordinates of both the optimized structures of $N5L_1$ at S_0 and S_1 in toluene.

S_0-optimized structure				S_1-optimized structure			
C	-4.15980100	-1.04682500	-0.21268900	C	4.05803200	1.38482800	-0.19860100
C	-4.16936500	-0.50971300	1.07320800	C	4.08168500	0.85830300	1.09422900
C	-4.11391300	-0.18905600	-1.31099800	C	4.10847800	0.51498400	-1.29511200
C	-4.06890500	0.86088600	1.26306300	C	4.10813300	-0.51159000	1.29553400
H	-4.22396000	-1.17999600	1.92335200	H	4.06029900	1.53751400	1.93918300
C	-3.99477300	1.18014500	-1.12117500	C	4.08314700	-0.85493500	-1.09381100
H	-4.13981800	-0.60354300	-2.31258900	H	4.12640000	0.92415100	-2.29908700
C	-3.93931200	1.71198000	0.16575300	C	4.05915800	-1.38148900	0.19900000
H	-4.05783900	1.27611500	2.26459300	H	4.12585000	-0.92074100	2.29951900
H	-3.91242700	1.84822400	-1.97072700	H	4.06288700	-1.53416100	-1.93878100
C	-2.84060400	-3.05807100	-0.22048500	C	2.57393100	3.26003300	-0.21751400
C	-1.74129900	-2.55595900	-0.91501600	C	1.51127800	2.60356400	-0.85103800
C	-2.63779400	-4.11745100	0.66545200	C	2.27716400	4.35189800	0.61596000
C	-0.47545500	-3.07525300	-0.71460200	C	0.20814300	3.00447500	-0.65890000
H	-1.87754000	-1.74283500	-1.61775600	H	1.71966000	1.76891300	-1.50923000
C	-1.36833800	-4.63766900	0.87407400	C	0.97013900	4.75255900	0.81995400
H	-3.48021100	-4.51776200	1.21845900	H	3.08362300	4.86884800	1.12362900
C	-0.26272100	-4.11187100	0.19736600	C	-0.09335800	4.08624000	0.18811100
H	0.36294500	-2.66622100	-1.26475600	H	-0.59384600	2.49118900	-1.17420300
H	-1.22908500	-5.43813100	1.59117700	H	0.76143600	5.57618500	1.49296900
C	-5.20907600	-3.15208700	-0.90857200	C	4.94130400	3.58608100	-0.83030100
C	-5.06259400	-4.42383000	-1.47868700	C	4.70641900	4.84372100	-1.40384700
C	-6.48918600	-2.58160600	-0.87429300	C	6.26586100	3.14226400	-0.71207300
C	-6.16432000	-5.09811100	-1.98424000	C	5.76709100	5.62899700	-1.82887100
H	-4.08239800	-4.88064100	-1.53187800	H	3.69028000	5.19777700	-1.52437100
C	-7.57865800	-3.26477600	-1.39296000	C	7.31431500	3.93445600	-1.15377100
H	-6.62796100	-1.60106900	-0.43540200	H	6.46762300	2.17533900	-0.26766300
C	-7.43185300	-4.52966000	-1.94962000	C	7.07954400	5.18618400	-1.71092200
H	-6.02143900	-6.08088400	-2.42104000	H	5.55863400	6.59773200	-2.27061800
H	-8.55749700	-2.79879500	-1.35219200	H	8.32981900	3.56690300	-1.05120800
H	-8.28762600	-5.06088100	-2.34937800	H	7.90338400	5.80390200	-2.04881300
C	2.08084900	-3.59313000	0.42820100	C	-2.38322600	3.40078500	0.46803800
C	3.20145000	-3.72945300	-0.39277000	C	-3.60903200	3.49285100	-0.22948500
C	1.94520200	-2.43182900	1.19033400	C	-2.07655100	2.18667300	1.12228000
C	4.12473700	-2.69914600	-0.50453800	C	-4.44524100	2.40675100	-0.33402200
H	3.32558000	-4.62907600	-0.98453800	H	-3.86775900	4.41848900	-0.73083500
C	2.86442200	-1.40521200	1.07910500	C	-2.90752500	1.10167800	1.01691400
H	1.09563500	-2.32899000	1.85522800	H	-1.17101600	2.11924500	1.71362400
C	3.94720100	-1.50839400	0.20260400	C	-4.09710300	1.16775700	0.25681500
H	4.96462800	-2.80072500	-1.18207400	H	-5.35488200	2.48306500	-0.91844300
H	2.73554700	-0.49933000	1.65956500	H	-2.65929100	0.17818000	1.52656200
C	1.35293700	-5.92498700	0.60340600	C	-1.83207100	5.77216100	0.54436000
C	2.41491700	-6.31664500	1.42543000	C	-2.86173900	6.12076100	1.42385800
C	0.61582000	-6.91132800	-0.06032500	C	-1.22044700	6.76964900	-0.22159900
C	2.73015500	-7.65921800	1.57223900	C	-3.26883800	7.44167200	1.53064600
H	2.98915300	-5.56247600	1.95035700	H	-3.32890900	5.35045800	2.02568100
C	0.92967100	-8.25212100	0.10801500	C	-1.62780900	8.08905500	-0.09703200
H	-0.20025500	-6.62351800	-0.71205700	H	-0.43460100	6.50244800	-0.91759500
C	1.98916000	-8.63818000	0.92054400	C	-2.65421500	8.43446900	0.77542400
H	3.55740800	-7.94051900	2.21481600	H	-4.06527200	7.69748000	2.22065500
H	0.34549500	-9.00076100	-0.41613200	H	-1.14651200	8.85166200	-0.69926300
H	2.23419500	-9.68670500	1.04366500	H	-2.97252500	9.46665700	0.86486500
C	-2.31021200	3.47871200	0.18629700	C	2.57660400	-3.25795000	0.21768000
C	-1.31220100	2.79807400	0.88115500	C	1.51342000	-2.60281300	0.85166800
C	-1.93397400	4.49900800	-0.68799400	C	2.28077900	-4.34959200	-0.61637400
C	0.02245500	3.10718900	0.69226900	C	0.21062400	-3.00478800	0.65936000
H	-1.58405600	2.01176200	1.57509900	H	1.72109400	-1.76836000	1.51033000
C	-0.59571100	4.80813700	-0.88608200	C	0.97409200	-4.75132800	-0.82051800
H	-2.69829300	5.03505600	-1.23949900	H	3.08768300	-4.86553300	-1.12436700
C	0.40564000	4.10337100	-0.20955600	C	-0.08994200	-4.08630300	-0.18824700
H	0.78040000	2.56330900	1.24227000	H	-0.59180900	-2.49248600	1.17495700
H	-0.32320200	5.58185700	-1.59404900	H	0.76608500	-5.57477600	-1.49396700
C	-4.61848900	3.94724600	0.91220200	C	4.94423000	-3.58211800	0.83049600
C	-5.97882300	3.60966800	0.87808100	C	6.26842400	-3.13710100	0.71270600
C	-4.24647900	5.15138700	1.52392300	C	4.71034200	-4.84019900	1.40348700
C	-6.92706500	4.45104400	1.43934900	C	7.31748500	-3.92855500	1.15429700
H	-6.29015500	2.68668400	0.40419100	H	6.46943800	-2.16982100	0.26873300
C	-5.20871400	5.98701300	2.07226600	C	5.77161400	-5.62470400	1.82842900
H	-3.20125200	5.42917900	1.57456500	H	3.69449100	-5.19521000	1.52363500
C	-6.55580300	5.64863500	2.03936500	C	7.08370600	-5.18069800	1.71092100
H	-7.97268400	4.16487400	1.39798500	H	8.33268900	-3.56007500	1.05207600
H	-4.89238700	6.91283200	2.54114100	H	5.56392000	-6.59380000	2.26974300
H	-7.30166300	6.30450800	2.47283100	H	7.90801900	-5.79782900	2.04873200
C	4.14114500	0.85358900	-0.20126600	C	-4.09605400	-1.17101100	-0.25645100
C	3.06067000	0.92842900	-1.08379200	C	-2.90655400	-1.10386100	-1.01656600
C	4.50377400	1.99888100	0.51069800	C	-4.44309500	-2.41030700	0.33431800
C	2.31966200	2.09004200	-1.19663000	C	-2.07469600	-2.18818000	-1.12207500
H	2.79032100	0.05670300	-1.66782000	H	-2.65908300	-0.18010500	-1.52611500
C	3.75872600	3.16445600	0.39759600	C	-3.60599900	-3.49573100	0.22961700
H	5.34471400	1.96249900	1.19358200	H	-5.35261500	-2.48742700	0.91882000
C	2.63598000	3.21250600	-0.42992600	C	-2.38034800	-3.40259600	-0.46798700
H	1.46842500	2.12665900	-1.86636900	H	-1.16920900	-2.11991900	-1.71339900
H	4.02245300	4.03063000	0.99353800	H	-3.86394700	-4.42162500	0.73089400
C	2.29456300	5.63094100	-0.61080000	C	-1.82733000	-5.77355100	-0.54492700
C	3.41036600	5.84288700	-1.42752600	C	-2.85699900	-6.12273100	-1.42420500
C	1.72157700	6.72644600	0.04373900	C	-1.21464700	-6.77081000	0.22049100
C	3.93716300	7.11691300	-1.57889800	C	-3.26304700	-7.44394100	-1.53129900
H	3.85954300	5.00368100	-1.94516600	H	-3.32500800	-5.35263100	-2.02563500
C	2.24691100	7.99868900	-0.12903300	C	-1.62095700	-8.09051100	0.09560500
H	0.86625900	6.57680500	0.69148900	H	-0.42879300	-6.50322200	0.91632900
C	3.35873000	8.20570400	-0.93699000	C	-2.64735800	-8.43649400	-0.77662700
H	4.80234800	7.25836600	-2.21751600	H	-4.05950000	-7.70016100	-2.22113500
H	1.78707600	8.83395700	0.38797700	H	-1.13881800	-8.85291800	0.69741700
H	3.76904000	9.20078600	-1.06361800	H	-2.96483500	-9.46891700	-0.86632000

C	6.18265500	-0.50222500	0.00966900	C	-6.22373600	-0.00257000	0.00025200
C	6.81321600	-1.48844400	0.77683300	C	-6.93001700	0.84050700	0.86448900
C	6.97424700	0.36682500	-0.74968500	C	-6.92937100	-0.84623800	-0.86393900
C	8.19543700	-1.60257900	0.77316700	C	-8.31597500	0.83946600	0.85538900
H	6.21538000	-2.16171700	1.37921500	H	-6.38436400	1.48108300	1.54654900
C	8.35636900	0.25251400	-0.73172400	C	-8.31532600	-0.84640900	-0.85470100
H	6.50130000	1.12850500	-1.35779600	H	-6.38323300	-1.48633600	-1.54605700
C	8.97980300	-0.73317500	0.02431400	C	-9.01753200	-0.00377600	0.00038000
H	8.66225900	-2.37438400	1.37557200	H	-8.85151500	1.49507100	1.53294300
H	8.94967700	0.93653600	-1.32888600	H	-8.85036200	-1.50247500	-1.53220800
H	10.05982700	-0.82250400	0.02974100	H	-10.10139200	-0.00424300	0.00043200
N	1.04958700	-4.56280400	0.44684700	N	-1.43387000	4.42751200	0.42611400
N	4.78595100	-0.38779700	0.00162000	N	-4.81824900	-0.00194500	0.00019000
N	1.77477400	4.33642600	-0.44994200	N	-1.43019800	-4.42863100	-0.42639200
N	-3.67065300	3.09873500	0.33884700	N	3.89314300	-2.78083100	0.38862200
N	-4.11807600	-2.45997900	-0.38340100	N	3.89085800	2.78401300	-0.38829300

Structure S12. The coordinates of both the optimized structures of $B6L_1$ at S_0 and S_1 in toluene.

S_0-optimized structure				S_1-optimized structure			
C	5.53860000	2.81436900	0.67309800	C	-4.18526200	-4.66450900	0.59018600
C	4.35242600	3.15292900	0.00226100	C	-2.91421600	-4.52485400	-0.01022000
C	3.67785300	2.11762300	-0.66468000	C	-2.63838100	-3.28231700	-0.62408300
C	4.16425000	0.81895000	-0.66478500	C	-3.55747000	-2.25335900	-0.62422400
C	5.35301600	0.48138500	0.00198600	C	-4.82334200	-2.38771900	-0.01080000
C	6.02496300	1.51571200	0.67303000	C	-5.10512700	-3.63487600	0.58983000
H	6.08400500	3.58428200	1.20900900	H	-4.43636000	-5.60030100	1.07774900
H	2.75849700	2.33917300	-1.19686100	H	-1.68083200	-3.13720800	-1.11290000
H	3.61661500	0.04806300	-1.19710100	H	-3.30555900	-1.31826400	-1.11318900
H	6.94195400	1.29339100	1.20886800	H	-6.06338300	-3.77912100	1.07705900
C	4.90659500	-2.19233900	-0.00371300	C	-5.37577900	0.26151700	0.01248200
C	5.20592200	-3.38862000	-0.67525300	C	-6.13187200	1.29223200	-0.58875800
C	3.67297200	-2.12575800	0.66368200	C	-4.16221300	0.64413100	0.62704400
C	4.32435800	-4.45909700	-0.67495100	C	-5.70006000	2.60371800	-0.58860600
H	6.14514300	-3.47594900	-1.21160200	H	-7.06734400	1.04156100	-1.07714000
C	2.79138600	-3.19625300	0.66398300	C	-3.73065700	1.95462700	0.62706000
H	3.40568000	-1.21899600	1.19645100	H	-3.55807300	-0.11244500	1.11646200
C	3.09298300	-4.39439000	-0.00326200	C	-4.47952200	2.98348700	0.01272700
H	4.58993900	-5.36430100	-1.21113600	H	-6.30359400	3.36126800	-1.07677000
H	1.85026400	-3.10749100	1.19684400	H	-2.79525900	2.20421100	1.11660700
C	7.44659200	-1.24231100	-0.00042000	C	-7.39497400	-1.54177000	-0.00088300
C	8.33664300	-0.35409900	-0.62663100	C	-7.91245600	-2.65081000	-0.69154500
C	7.99931200	-2.37135400	0.62649700	C	-8.31385300	-0.73206000	0.68821600
C	9.70500100	-0.58687300	-0.64069700	C	-9.27219800	-2.93036600	-0.70519900
H	7.94423400	0.52697100	-1.12324800	H	-7.23511800	-3.29683300	-1.24037600
C	9.36908000	-2.59554500	0.64213600	C	-9.67213200	-1.01885500	0.69868700
H	7.34160100	-3.07704900	1.12279400	H	-7.95222400	0.13055700	1.23816700
C	10.22412800	-1.70586200	0.00112000	C	-10.15565400	-2.11691300	-0.00419900
H	10.36864700	0.10627400	-1.14618400	H	-9.64412400	-3.78572700	-1.25900600
H	9.77115700	-3.46658100	1.14813100	H	-10.35594200	-0.38348900	1.25132400
C	-3.09299800	4.39437000	0.00375800	C	4.47954800	-2.98353900	-0.01298900
C	-4.32462000	4.45905300	0.67501800	C	5.70024700	-2.60375600	0.58806900
C	-2.79118300	3.19631100	-0.66353100	C	3.73052600	-1.95466900	-0.62716800
C	-5.20621600	3.38859000	0.67488900	C	6.13204500	-1.29229000	0.58810600
H	-4.59035000	5.36421000	1.21119400	H	6.30391900	-3.36132700	1.07602500
C	-3.67280500	2.12584000	-0.66366700	C	4.16204200	-0.64419200	-0.62721800
H	-1.84991800	3.10762900	-1.19613900	H	2.79498800	-2.20426400	-1.11644000
C	-4.90660800	2.19233700	0.00341200	C	5.37577700	-0.26156800	-0.01294900
H	-6.14564200	3.47587100	1.21087700	H	7.06766800	-1.04160700	1.07618600
H	-3.40538200	1.21915600	-1.19649100	H	3.55775400	0.11240400	-1.11641800
C	0.55429300	-5.34511300	0.00254200	C	-2.46153200	4.78679500	-0.01209500
C	-0.00507100	-4.24343600	-0.66463600	C	-1.52364400	3.92692400	-0.62671100
C	-0.33193100	-6.20286200	0.67372000	C	-1.94669700	5.95712300	0.58854800
C	-1.37297600	-4.01537400	-0.66480400	C	-0.17299600	4.20849800	-0.62744000
H	0.64637100	-3.55832000	-1.19729400	H	-1.87689700	3.02521200	-1.11567100
C	-1.69980300	-5.97481700	0.67354600	C	-0.59503300	6.23892300	0.58779700
H	0.06224700	-7.05991300	1.20989900	H	-2.63131600	6.64214700	1.07689000
C	-2.25966000	-4.87594600	0.00223100	C	0.34386100	5.37161200	-0.01362900
H	-1.76684800	-3.15593100	-1.19745400	H	0.51071900	3.52304900	-1.11693800
H	-2.35072500	-6.65766100	1.20965600	H	-0.24056100	7.14052600	1.07556500
C	-5.35316800	-0.48140400	-0.00263700	C	4.82331100	2.38765500	0.01028000
C	-4.16436800	-0.81891400	0.66412100	C	3.55738300	2.25324700	0.62354500
C	-6.02517600	-1.51577500	-0.67355000	C	5.10521400	3.63487000	-0.59014600
C	-3.67789300	-2.11756500	0.66399300	C	2.63832900	3.28223500	0.62341600
H	-3.61671900	-0.04798500	1.19635100	H	3.30538800	1.31809200	1.11235100
C	-5.53873000	-2.81441100	-0.67365600	C	4.18537200	4.66452300	-0.59048000
H	-6.94221800	-1.29348600	-1.20930500	H	6.06355200	3.77915700	-1.07720100
C	-4.35249500	-3.15289400	-0.00287900	C	2.91428900	4.52484000	0.00978300
H	-2.75848800	-2.33908000	1.19609300	H	1.68071300	3.13708200	1.11208700
H	-6.08410800	-3.58436500	-1.20952500	H	4.43656300	5.60035700	-1.07791200
C	-7.44666000	1.24236400	-0.00014900	C	7.39491600	1.54177300	0.00095400
C	-7.99961600	2.37104900	-0.62751100	C	8.31415100	0.73204500	-0.68764300
C	-8.33647800	0.35440300	0.62676200	C	7.91198800	2.65095400	0.69169000
C	-9.36940400	2.59514300	-0.64287400	C	9.67241200	1.01893900	-0.69752500
C	-9.70484600	0.58710600	0.64110300	C	9.27170200	2.93061700	0.70591400
C	-10.22420200	1.70574400	-0.00113500	C	10.15553100	2.11712900	0.00543200
H	-9.77170500	3.46586400	-1.14923100	H	10.35652300	0.38353200	-1.24974300
H	-10.36832900	-0.10580500	1.14712700	H	9.64331000	3.78611000	1.25973100
C	-4.79895100	-5.82770800	-0.00082900	C	2.36277900	7.17518200	-0.00322300
C	-6.05282300	-5.74295300	0.62677700	C	3.52355300	7.56564500	0.68590300
C	-4.47463600	-7.04178900	-0.62861800	C	1.66109300	8.17818700	-0.69347500
C	-6.93147100	-6.81745200	0.64158500	C	3.95445000	8.88526400	0.69685700
C	-5.36011100	-8.11065800	-0.64367400	C	2.09903400	9.49546800	-0.70668100
C	-6.58846400	-8.00187300	-0.00111400	C	3.24521900	9.85344000	-0.00546100
H	-7.88663800	-6.73104200	1.14815200	H	4.84667600	9.15952900	1.24949500
H	-5.09162100	-9.03129600	-1.15038200	H	1.54433100	10.24557300	-1.26014600
C	2.64751200	-7.06924300	0.00087100	C	-5.03329300	5.63317400	0.00294300
C	3.86133800	-7.39488100	0.62845900	C	-6.25294900	5.52524000	0.69261400
C	1.94701400	-8.11326900	-0.62568900	C	-4.79207900	6.83449100	-0.68513500
C	4.34453300	-8.69604700	0.64413400	C	-7.17590100	6.56212100	0.70626500
H	4.42738900	-6.61382300	1.12491600	H	-6.47339500	4.61505800	1.24064400
C	2.43825400	-9.41144400	-0.63952200	C	-5.72053900	7.86651500	-0.69563500
H	1.00713600	-7.89744900	-1.12268300	H	-3.86396800	6.95377000	-1.23440800
C	3.63588800	-9.70599600	0.00275200	C	-6.91380700	7.73455100	0.00604100
H	5.27640300	-8.92327600	1.15047700	H	-8.10295400	6.45534300	1.25929000
H	1.88571300	-10.19593600	-1.14518700	H	-5.51263200	8.77694200	-1.24753300
C	2.25969100	4.87605500	-0.00242700	C	-0.34381000	-5.37161600	0.01331700
C	1.69967200	5.97521600	-0.67311800	C	0.59516300	-6.23910800	-0.58779100
C	1.37318600	4.01524800	0.66453600	C	0.17300000	-4.20834200	0.62693000
C	0.33180300	6.20328200	-0.67282800	C	1.94680400	-5.95731300	-0.58847700
H	2.35047900	6.65821900	-1.20917000	H	0.24072600	-7.14084300	-1.07533500
C	0.00528100	4.24328800	0.66478600	C	1.52362400	-3.92678900	0.62630000
H	1.76722600	3.15565000	1.19681600	H	-0.51078300	-3.52276000	1.11614400
C	-0.55427200	5.34519500	-0.00187000	C	2.46159300	-4.78683100	0.01197200

H	-0.06246700	7.06062600	-1.20846700	H	2.63149400	-6.64246600	-1.07654100
H	-0.64599800	3.55791600	1.19730400	H	1.87684400	-3.02493500	1.11501800
C	4.79904700	5.82768400	-0.00073000	C	-2.36272800	-7.17516500	0.00310200
C	4.47505000	7.04206000	0.62663500	C	-1.66110100	-8.17807100	0.69355400
C	6.05274600	5.74253400	-0.62862200	C	-3.52350200	-7.56569500	-0.68598700
C	5.36059900	8.11087500	0.64092600	C	-2.09908000	-9.49533700	0.70695000
H	3.51613700	7.14220900	1.12404000	H	-0.76284100	-7.91472000	1.24229800
C	6.93148100	6.81694300	-0.64416400	C	-3.95444500	-8.88530000	-0.69672600
H	6.33491700	4.82041700	-1.12563000	H	-4.08959100	-6.82092100	-1.23571000
C	6.58876100	8.00170700	-0.00193900	C	-3.24525800	-9.85338600	0.00575700
H	5.09230000	9.03177600	1.14725800	H	-1.54440300	-10.24537500	1.26053200
H	7.88650800	6.73016600	-1.15093300	H	-4.84668400	-9.15961800	-1.24931800
C	-2.64752300	7.06925900	0.00112400	C	5.03330900	-5.63321100	-0.00281200
C	-3.86126100	7.39536600	-0.62639700	C	6.25269700	-5.52567100	-0.69301300
C	-1.94705900	8.11281300	0.62851300	C	4.79235800	-6.83411500	0.68607000
C	-4.34446500	8.69654900	-0.64112500	C	7.17563100	-6.56257100	-0.70642600
C	-2.43834600	9.41096100	0.64333500	C	5.72080600	-7.86614800	0.69678500
C	-3.63590700	9.70598700	0.00114600	C	6.91379900	-7.73460200	-0.00543500
H	-5.27624900	8.92421000	-1.14743400	H	8.10248100	-6.45610700	-1.25985400
H	-1.88590600	10.19506300	1.14971500	H	5.51308600	-8.77626600	1.24926300
B	-3.80542400	-4.62092700	-0.00051300	B	1.87258700	5.68736800	-0.00225600
B	-5.90485200	0.98490300	-0.00007000	B	5.86140400	1.22193300	-0.00084800
B	-2.09915600	5.60545000	0.00100600	B	3.98910400	-4.46543800	-0.00120600
H	4.01657700	-10.72175300	0.00338900	H	-7.63716500	8.54283700	0.00716400
H	-4.01657800	10.72175200	0.00118100	H	7.63714700	-8.54289600	-0.00638000
H	-1.00721900	7.89662500	1.12541200	H	3.86445300	-6.95307100	1.23576800
H	-4.42730700	6.61467900	-1.12343300	H	6.47295600	-4.61578500	-1.24161300
H	7.27826500	8.83913700	-0.00233100	H	-3.58474200	-10.88359700	0.00675800
H	11.29413800	-1.88425900	0.00178200	H	-11.21753100	-2.33825200	-0.00546500
H	-7.34208400	3.07656100	-1.12430300	H	7.95283200	-0.13069300	-1.23760800
H	-11.29422400	1.88408000	-0.00158200	H	11.21739100	2.33854500	0.00714600
H	-6.33524800	-4.82106900	1.12406100	H	4.08966500	6.82081700	1.23553300
H	-7.27788500	-8.83937200	-0.00126400	H	3.58467400	10.88366200	-0.00630800
H	-3.51557000	-7.14163200	-1.12577500	H	0.76283200	7.91489900	-1.24224500
H	-7.94388900	-0.52647300	1.12358100	H	7.23433000	3.29703200	1.24006000
B	2.09913600	-5.60543800	-0.00002200	B	-3.98907900	4.46541200	0.00110500
B	5.90479000	-0.98487600	-0.00086000	B	-5.86144700	-1.22199500	0.00030300
B	3.80542500	4.62098400	-0.00024900	B	-1.87250800	-5.68735600	0.00186900

Structure S13. The coordinates of both the optimized structures of B5N1L$_1$ at S$_0$ and S$_1$ in toluene.

S_0-optimized structure				S_1-optimized structure			
C	5.01574200	-3.52391800	-0.69387700	C	4.30579800	-4.29381400	-0.60565600
C	3.77994200	-3.70632600	-0.04927200	C	3.04472500	-4.23811200	0.01545500
C	3.25656100	-2.58443400	0.61744400	C	2.71113800	-3.04602000	0.68207900
C	3.92030800	-1.37192700	0.65917700	C	3.58165700	-1.97471700	0.74706700
C	5.13624400	-1.20985200	-0.01662300	C	4.83329700	-2.06265900	0.12001900
C	5.67402800	-2.30720500	-0.69989700	C	5.18799400	-3.23250400	-0.56823600
H	5.45886500	-4.35281700	-1.23543300	H	4.59147900	-5.18786000	-1.14774200
H	2.31348000	-2.67436500	1.14593900	H	1.75370400	-2.96693300	1.18371400
H	3.50071300	-0.54261100	1.21557400	H	3.31932900	-1.08306700	1.30294200
H	6.60820600	-2.19729600	-1.23725600	H	6.13803700	-3.28699300	-1.08516900
C	5.12883400	1.24066800	0.01665700	C	5.26169300	0.32521700	0.11497800
C	5.65971600	2.34112700	0.70032500	C	5.87154700	1.33278100	0.88595700
C	3.91223900	1.39549500	-0.65964000	C	4.18878400	0.65234900	-0.73401600
C	4.99410800	3.55384700	0.69422900	C	5.40900400	2.62780300	0.79964700
H	6.59433200	2.23675600	1.23802600	H	6.67339500	1.07600200	1.56762300
C	3.24117100	2.60396700	-0.61798800	C	3.75421600	1.95865800	-0.80654700
H	3.49789000	0.56372500	-1.21630600	H	3.74222200	-0.11420600	-1.35579100
C	3.75746500	3.72887900	0.04918200	C	4.33821000	2.99136600	-0.04352200
H	5.43198700	4.38532300	1.23610000	H	5.87865100	3.39098500	1.40997200
H	2.29781000	2.68831500	-1.14690400	H	2.94355200	2.20210100	-1.48349500
C	7.23527800	0.02178100	0.00014800	C	7.10418900	-1.23252500	0.31528500
C	7.93952700	-0.74930800	0.92482100	C	7.55495900	-2.26306800	1.15121400
C	7.93489500	0.79713700	-0.92446700	C	8.02308400	-0.44150700	-0.38753600
C	9.32730900	-0.74888900	0.91618500	C	8.91278100	-2.49175600	1.27942400
H	7.39405300	-1.34607200	1.64662000	H	6.84041600	-2.85096900	1.71346600
C	9.32265300	0.80519600	-0.91568500	C	9.37773400	-0.68575900	-0.25058600
H	7.38586700	1.39056300	-1.64632100	H	7.66596100	0.33366300	-1.05373900
C	10.02487000	0.03029500	0.00028700	C	9.82750600	-1.70745100	0.58105300
H	9.86503800	-1.35188700	1.63933500	H	9.26133600	-3.27728900	1.93889100
H	9.85676200	1.41146800	-1.63878100	H	10.08647700	-0.08321500	-0.80542800
H	11.10886100	0.03360700	0.00033700	H	10.88982500	-1.89263800	0.68540600
C	-3.76023500	-3.82622000	0.10549900	C	-4.40810100	-3.09052800	-0.09151300
C	-4.99218300	-3.71881600	-0.55939800	C	-5.60892500	-2.71410300	-0.71642700
C	-3.27404600	-2.67155800	0.73956200	C	-3.72307000	-2.09356700	0.62273100
C	-5.69669400	-2.52450500	-0.59041100	C	-6.09519400	-1.41797100	-0.63048300
H	-5.39869000	-4.58702500	-1.06780100	H	-6.16413500	-3.45097200	-1.28767700
C	-3.97887000	-1.47776900	0.70944900	C	-4.20831200	-0.79754500	0.70556400
H	-2.32727700	-2.71454700	1.26807500	H	-2.79521500	-2.34386700	1.12692500
C	-5.21018300	-1.36962100	0.04289100	C	-5.40725800	-0.42156700	0.07947100
H	-6.64079400	-2.48033100	-1.12348500	H	-7.02518000	-1.16654100	-1.13038900
H	-3.57078000	-0.60880200	1.21527000	H	-3.65034000	-0.05629300	1.26820900
C	1.42755900	5.10179100	0.01168700	C	2.33852600	4.79300100	-0.07866600
C	0.66020800	4.11601500	0.65212200	C	1.39321000	3.84466200	0.40252900
C	0.72928400	6.10493400	-0.67909800	C	1.77708700	6.02457500	-0.51801900
C	-0.72573000	4.13330400	0.60667300	C	0.04205400	4.09586600	0.43470800
H	1.16248800	3.32701700	1.20241500	H	1.75189800	2.89240100	0.78216100
C	-0.65663500	6.12049900	-0.72772100	C	0.42487000	6.27348800	-0.48448400
H	1.28635700	6.88326300	-1.19068100	H	2.43563500	6.78060900	-0.93296000
C	-1.42477100	5.13661100	-0.08355300	C	-0.51825600	5.32148400	-0.01353200
H	-1.28210200	3.35756900	1.12256100	H	-0.62009600	3.33761600	0.84043200
H	-1.15827400	6.90818400	-1.28035400	H	0.06013700	7.22158000	-0.86696900
C	-5.21834000	1.33821100	-0.04268300	C	-4.94558700	2.23978000	0.12014900
C	-3.98774500	1.45372500	-0.70933000	C	-3.72571100	2.13852700	-0.57476600
C	-5.71183500	2.49022700	0.59043400	C	-5.19751600	3.46537900	0.76563100
C	-3.29015700	2.65174300	-0.73963100	C	-2.82333100	3.18714300	-0.62077000
H	-3.57442100	0.58716300	-1.21502900	H	-3.49405600	1.21946800	-1.10400900
C	-5.01455500	3.68877400	0.55923900	C	-4.28701400	4.50831700	0.72892300
H	-6.65566100	2.44043000	1.12349900	H	-6.12001300	3.58930300	1.32417900
C	-3.78328700	3.80352700	-0.10569300	C	-3.06265700	4.40607700	0.04160100
H	-2.34368800	2.70038800	-1.26819500	H	-1.90758700	3.07039400	-1.19110400
H	-5.42630500	4.55458400	1.06751700	H	-4.51907400	5.42934500	1.25432100
C	-7.56460600	-0.02274800	0.00020400	C	-7.48758400	1.29426600	0.32159900
C	-8.29303800	-1.02645300	0.65991900	C	-8.30948000	0.37787600	0.99815200
C	-8.29895600	0.97666000	-0.65948100	C	-8.10828800	2.42985300	-0.22439900
C	-9.68100600	-1.02570900	0.67467900	C	-9.67368300	0.59106200	1.14137700
H	-7.75839300	-1.81274300	1.18224800	H	-7.86481200	-0.51254800	1.43047000
C	-9.68689500	0.96767300	-0.67429900	C	-9.47624200	2.63736800	-0.10859700
H	-7.76896600	1.76610500	-1.18179600	H	-7.50584600	3.15479200	-0.76131100
C	-10.38053300	-0.03108400	0.00017900	C	-10.26111900	1.72060800	0.58196300
H	-10.21890600	-1.80349500	1.20584400	H	-10.28165800	-0.12547800	1.68342300
H	-10.22937900	1.74226500	-1.20547000	H	-9.93101700	3.51649300	-0.55246500
H	-11.46534500	-0.03429300	0.00018300	H	-11.32867600	1.88535700	0.68274100
C	-3.76248800	6.51325200	-0.17788200	C	-2.59513400	7.06476200	-0.02609600
C	-4.99596800	6.62600700	-0.84050500	C	-3.76670600	7.38839000	-0.73165400
C	-3.25501200	7.66952400	0.43745600	C	-1.96527800	8.11587000	0.66166900
C	-5.68003400	7.83228000	-0.90142400	C	-4.26940800	8.68248000	-0.76755900
H	-5.41736900	5.75301900	-1.32766400	H	-4.28900300	6.60486400	-1.27174600
C	-3.94740300	8.87223700	0.40607700	C	-2.47003500	9.40989800	0.64726400
H	-2.30704700	7.61798600	0.96240200	H	-1.06574500	7.90707300	1.23168800
C	-5.15894000	8.95688000	-0.27106100	C	-3.62298600	9.69996500	-0.07395500
H	-6.62269300	7.89582500	-1.43414000	H	-5.16940600	8.89862100	-1.33428400
H	-3.54098100	9.74602300	0.90382500	H	-1.96331300	10.19476400	1.19960800
H	-5.69700400	9.89813700	-0.30681500	H	-4.01671200	10.71081400	-0.09281000
C	3.77653100	6.43834400	0.18133100	C	4.93763700	5.59687300	-0.31962100
C	5.04404900	6.60064300	-0.40145800	C	6.12694800	5.34379300	-1.02828700
C	3.23349200	7.54435400	0.85548800	C	4.80926100	6.87803200	0.24834400
C	5.73048000	7.80508200	-0.32495000	C	7.11174600	6.30903700	-1.18437100
H	5.49046000	5.77010500	-0.93813800	H	6.27466100	4.36760400	-1.48062100
C	3.92567200	8.74301300	0.96096700	C	5.79606700	7.84474200	0.11044700
H	2.25475000	7.45566500	1.31524900	H	3.92175700	7.11088800	0.82680900
C	5.17463000	8.87697500	0.36443600	C	6.95111400	7.56738000	-0.61310900
H	6.70102000	7.90818600	-0.79824700	H	8.00867800	6.08152400	-1.75165900
H	3.48965400	9.57613200	1.50165800	H	5.66510500	8.81855300	0.57107000
H	5.71301100	9.81610000	0.43496900	H	7.72095500	8.32310500	-0.72706000

C	1.45837400	-5.09325900	-0.01183500	C	0.51893400	-5.22260500	-0.08547900
C	0.76607600	-6.10058200	0.67888500	C	-0.37801100	-6.11477900	0.52572300
C	0.68517400	-4.11200900	-0.65218300	C	-0.03386000	-4.10711800	-0.73677000
C	-0.61973400	-6.12443700	0.72745000	C	-1.74784200	-5.90329600	0.48678300
H	1.32775800	-6.87558800	1.19045800	H	0.00914300	-6.98335300	1.04799900
C	-0.70065000	-4.13758700	-0.60678700	C	-1.40383900	-3.89629500	-0.77468000
H	1.18274300	-3.31996600	-1.20237000	H	0.62268200	-3.39687500	-1.22933800
C	-1.39370900	-5.14512100	0.08331800	C	-2.29912500	-4.78831100	-0.16320700
H	-1.11667500	-6.91512700	1.28003100	H	-2.40749600	-6.61256200	0.97576100
H	-1.26161600	-3.36515600	-1.12264800	H	-1.79223500	-3.02394200	-1.28975000
C	3.81546300	-6.41560500	-0.18142300	C	2.63977100	-6.91344800	-0.00025600
C	3.27950000	-7.52469800	-0.85618700	C	1.99180000	-7.96649500	-0.66809400
C	5.08364800	-6.57038300	0.40196100	C	3.82702600	-7.22040800	0.68590300
C	3.97901200	-8.71909000	-0.96169100	C	2.50883500	-9.25440900	-0.66480200
H	2.30047700	-7.44182400	-1.31642600	H	1.07458000	-7.76506700	-1.21087800
C	5.77744900	-7.77058700	0.32539300	C	4.33354300	-8.51191800	0.71887200
H	5.52471100	-5.73733300	0.93916900	H	4.35168100	-6.43432400	1.21915200
C	5.22847500	-8.84562800	-0.36460100	C	3.67764100	-9.53051700	0.03597200
H	3.54833400	-9.55467400	-1.50285900	H	1.99785400	-10.04572100	-1.20212000
H	6.74836000	-7.86793000	0.79914900	H	5.24143600	-8.72623700	1.27213300
H	5.77259400	-9.78144000	-0.43515900	H	4.07679800	-10.53891800	0.05053200
C	-3.72314600	-6.53574300	0.17772400	C	-4.82027700	-5.76725900	-0.31182500
C	-4.95594600	-6.65579900	0.84032600	C	-6.08923200	-5.75678000	0.29052600
C	-3.20875600	-7.68901800	-0.43749100	C	-4.45504700	-6.92254600	-1.02253200
C	-5.63280200	-7.86613000	0.90133300	C	-6.94493000	-6.84603900	0.19929900
H	-5.38256700	-5.78530200	1.32740100	H	-6.40181900	-4.88300800	0.85222300
C	-3.89395300	-8.89584000	-0.40602100	C	-5.31688900	-8.00385300	-1.14327700
H	-2.26108500	-7.63188000	-0.96239000	H	-3.48196700	-6.96531500	-1.50056800
C	-5.10498800	-8.98765400	0.27108300	C	-6.56226400	-7.96924600	-0.52548600
H	-6.57507400	-7.93525200	1.43404400	H	-7.91303300	-6.81819000	0.68747700
H	-3.48230500	-9.76722500	-0.90368500	H	-5.01722900	-8.87687800	-1.71296400
H	-5.63742400	-9.93210400	0.30690000	H	-7.23381900	-8.81714200	-0.60824100
N	5.81621000	0.01746000	0.00008200	N	5.72787000	-0.98724700	0.18357400
B	-2.98988800	5.15514800	-0.12588100	B	-2.03293800	5.59361700	0.00201300
B	-6.00146900	-0.01808100	0.00023300	B	-5.94761200	1.05261200	0.17324600
B	-2.95868500	-5.17303200	0.12557000	B	-3.85158500	-4.54647700	-0.19123400
B	3.02616800	-5.06795000	-0.07809100	B	2.05953000	-5.46486000	-0.02614200
B	2.99546100	5.08591300	0.07799100	B	3.83457000	4.49506800	-0.14497200

Structure S14. The coordinates of both the optimized structures of B4N2L$_1$ at S$_0$ and S$_1$ in toluene.

S$_0$-optimized structure				S$_1$-optimized structure			
C	-5.81088100	0.69745100	-0.93052900	C	0.44458000	-6.07352300	0.54243500
C	-4.98492800	1.40840000	-0.05864300	C	1.21545500	-5.02478600	-0.01348100
C	-4.16281300	0.69587100	0.81694900	C	0.52872200	-3.92331000	-0.57724300
C	-4.16408700	-0.68884400	0.81694200	C	-0.83899400	-3.87015700	-0.57694300
C	-4.98750800	-1.39986100	-0.05865400	C	-1.60927400	-4.91492200	-0.01314000
C	-5.81216200	-0.68739600	-0.93053400	C	-0.92216500	-6.02036000	0.54276500
H	-6.44913200	1.23601800	-1.62100500	H	0.94312600	-6.90573700	1.02102600
H	-3.53084600	1.23254900	1.51474200	H	1.09076000	-3.13185700	-1.05462300
H	-3.53310800	-1.22670000	1.51472200	H	-1.33819000	-3.03710900	-1.05329300
H	-6.45140400	-1.22477900	-1.62101300	H	-1.48306000	-6.81108700	1.02261800
C	-3.81813400	-3.54588300	-0.01817500	C	-3.67188100	-3.62307900	-0.03437300
C	-3.76521600	-4.80253600	0.60148000	C	-4.79404400	-3.48038600	-0.85226400
C	-2.64782700	-3.04278900	-0.60375100	C	-3.26019300	-2.56116600	0.77274400
C	-2.58775300	-5.52659500	0.61326100	C	-5.49137300	-2.28416300	-0.85249400
H	-4.65187500	-5.20230700	1.07826200	H	-5.11070500	-4.30381200	-1.48163000
C	-1.47272900	-3.76893100	-0.55146800	C	-3.95347800	-1.36252900	0.73168600
H	-2.66954300	-2.08247200	-1.10438300	H	-2.41206100	-2.68327900	1.43678300
C	-1.39533100	-5.03945700	0.04762800	C	-5.09010600	-1.18620000	-0.07350600
H	-2.58309000	-6.49181300	1.10826800	H	-6.36501600	-2.19160600	-1.48832600
H	-0.58829600	-3.34946200	-1.01895400	H	-3.61820700	-0.54552200	1.36056400
C	-6.26069900	-3.47092500	-0.07253100	C	-3.74913000	-6.05130800	0.06817500
C	-7.29330300	-3.04205700	0.76299600	C	-3.48150700	-7.10573200	-0.80556100
C	-6.48452900	-4.54044500	-0.94188900	C	-4.78906700	-6.15035700	0.99233300
C	-8.52901600	-3.67225100	0.72430800	C	-4.24573100	-8.26124300	-0.74057500
H	-7.12128300	-2.21439600	1.44108500	H	-2.68971400	-7.00774500	-1.53906200
C	-7.71783400	-5.17587200	-0.96152200	C	-5.54875200	-7.30866400	1.04513600
H	-5.68865800	-4.86859500	-1.60015100	H	-4.98995000	-5.32573400	1.66535600
C	-8.74747700	-4.74425600	-0.13304100	C	-5.27930600	-8.36663500	0.18308800
H	-9.32204600	-3.32874200	1.37929700	H	-4.04102300	-9.07585100	-1.42530200
H	-7.87774500	-6.00511400	-1.64174700	H	-6.35216600	-7.38648800	1.76812200
H	-9.71191400	-5.23836600	-0.15651200	H	-5.87749000	-9.26904900	0.22765500
C	1.32022400	5.10460800	0.05733500	C	5.20288500	1.10365700	-0.04181400
C	2.42486400	5.63536900	-0.62760800	C	5.84055100	2.24569600	0.49002700
C	1.50761500	3.87847000	0.71489900	C	3.89635500	1.30121300	-0.53778200
C	3.64418300	4.97494300	-0.65960700	C	5.21526800	3.47787500	0.53393000
H	2.32106600	6.58146700	-1.14907700	H	6.83983600	2.14767800	0.90231700
C	2.72601500	3.21695700	0.68202700	C	3.27904900	2.53631700	-0.50594800
H	0.68170800	3.44021100	1.26577800	H	3.36828300	0.46328600	-0.98243100
C	3.82947600	3.74524800	-0.00720800	C	3.90611500	3.67470400	0.04418900
H	4.47069200	5.41474100	-1.20836700	H	5.74089900	4.31812000	0.97625200
H	2.82981400	2.27200700	1.20537100	H	2.28637300	2.63875200	-0.93107600
C	1.31116400	-5.10677800	0.05731200	C	-5.09802500	1.50997400	-0.03842500
C	1.50067600	-3.88096300	0.71486400	C	-3.78357700	1.60602600	-0.54221100
C	2.41486600	-5.63945900	-0.62765500	C	-5.64099600	2.69510400	0.50316300
C	2.72020400	-3.22153800	0.68192600	C	-3.07147200	2.78930400	-0.51012300
H	0.67555100	-3.44130400	1.26579500	H	-3.32564100	0.73086100	-0.99290800
C	3.63531800	-4.98112000	-0.65971900	C	-4.92081700	3.87460700	0.54773700
H	2.30941000	-6.58538100	-1.14910400	H	-6.64237100	2.67349100	0.92146100
C	3.82272000	-3.75171700	-0.00737000	C	-3.60449300	3.97030000	0.04830100
H	2.82565800	-2.27676400	1.20525000	H	-2.07655500	2.81603300	-0.94150200
H	4.46104900	-5.42233600	-1.20850400	H	-5.37614800	4.75159000	0.99667800
C	5.21364200	1.42204800	-0.06398100	C	1.64870500	5.15718500	0.14872400
C	4.20822500	0.68962400	-0.71541500	C	0.85293700	4.13631100	0.71662400
C	6.22017600	0.68820400	0.58384300	C	0.93802000	6.25937700	-0.37823500
C	4.20698600	-0.69670000	-0.71547500	C	-0.52579600	4.19079500	0.71595300
H	3.41501900	1.22030300	-1.23177500	H	1.34306500	3.28804900	1.18369300
C	6.21892700	-0.69897200	0.58381900	C	-0.44238900	6.31434500	-0.37823300
H	7.01144100	1.21728700	1.10496000	H	1.49531600	7.07447000	-0.82901300
C	5.21107700	-1.43095900	-0.06405000	C	-1.23803100	5.27166700	0.14828500
H	3.41288900	-1.22591000	-1.23195600	H	-1.08201500	3.38386400	1.18230000
H	7.00921600	-1.22949400	1.10494600	H	-0.93322100	7.17095900	-0.82942000
C	6.54301600	3.78794400	-0.07977800	C	4.06455000	6.37885400	0.05050200
C	6.66361800	5.03870500	0.54813000	C	5.24390200	6.46770400	-0.70850800
C	7.67979000	3.28398800	-0.73321400	C	3.69709800	7.52306600	0.77897500
C	7.85924500	5.74383100	0.53818000	C	6.00564000	7.62826900	-0.74872700
H	5.80631300	5.45679600	1.06498400	H	5.56149100	5.60821800	-1.29030100
C	8.87053400	3.99627300	-0.77218000	C	4.46185200	8.68224600	0.76019400
H	7.62167500	2.32187600	-1.23114200	H	2.79479400	7.49535800	1.38138500
C	8.96364700	5.22574800	-0.12923900	C	5.61886900	8.74061700	-0.00897200
H	7.93010700	6.70013600	1.04501900	H	6.90376800	7.66615800	-1.35676700
H	9.72869900	3.59164100	-1.29779900	H	4.15413200	9.54350200	1.34434000
H	9.89612900	5.77972700	-0.14819400	H	6.21505200	9.64680800	-0.03200500
C	6.53618700	-3.79924300	-0.07968900	C	-3.55059000	6.67905600	0.05246000
C	7.67390100	-3.29729400	-0.73303100	C	-3.09068800	7.79285600	0.77564100
C	6.65452500	-5.05018100	0.54829400	C	-4.72320200	6.85796400	-0.70113100
C	8.86339600	-4.01167500	-0.77186200	C	-3.76179000	9.00860200	0.75636300
H	7.61751800	-2.33509800	-1.23099800	H	-2.19089500	7.69571100	1.37457400
C	7.84892000	-5.75740100	0.53847600	C	-5.39123700	8.07481900	-0.74173400
H	5.79644700	-5.46673800	1.06509700	H	-5.11072200	6.02444500	-1.27827400
C	8.95428500	-5.24128800	-0.12887200	C	-4.91444300	9.15542400	-0.00754900
H	9.72232900	-3.60856900	-1.29740400	H	-3.38457200	9.84464800	1.33634400
H	7.91806400	-6.71382000	1.04533900	H	-6.28671900	8.18151700	-1.34547100
H	9.88579300	-5.79690600	-0.14772600	H	-5.43741700	10.10574800	-0.03105200
C	-0.09317900	-7.41644700	0.16238400	C	-7.44720300	0.13718000	-0.12288400
C	-1.08906300	-8.17075800	-0.47900400	C	-8.17262500	-0.91426700	0.46346900
C	0.89181600	-8.12460200	0.86990300	C	-8.19052500	1.14340800	-0.76375100
C	-1.09911700	-9.55818800	-0.42417000	C	-9.55971900	-0.95171000	0.43193600
H	-1.86016300	-7.65793600	-1.04432700	H	-7.63520500	-1.71129100	0.96744900
C	0.87278600	-9.51022300	0.95338000	C	-9.57705700	1.10206800	-0.81932100
H	1.68017600	-7.57465900	1.37325400	H	-7.66601100	1.96488500	-1.23979200
C	-0.12129300	-10.23054200	0.30029100	C	-10.26646600	0.05656500	-0.21484600
H	-1.87159400	-10.11633100	-0.94229000	H	-10.09231200	-1.76811400	0.90833000
H	1.63699600	-10.03054000	1.52074700	H	-10.12214000	1.88745200	-1.33197200
H	-0.13227500	-11.31399100	0.35361100	H	-11.35031200	0.02670200	-0.24912300

C	-3.81175500	3.55236700	-0.01801600	C	3.37408200	-3.89943900	-0.03479100
C	-3.75660600	4.80882100	0.60184000	C	4.50418100	-3.84668400	-0.85256500
C	-2.64232600	3.04729700	-0.60367700	C	3.04949300	-2.80856900	0.77348300
C	-2.57789400	5.53085100	0.61360300	C	5.29467300	-2.70980100	-0.85295900
H	-4.64251500	5.21005000	1.07878000	H	4.75376900	-4.69237300	-1.48259600
C	-1.46597600	3.77139800	-0.55137400	C	3.83651000	-1.66934400	0.73274100
H	-2.66573100	2.08708200	-1.10442600	H	2.19520700	-2.86230900	1.43866900
C	-1.38636000	5.04175200	0.04779900	C	4.98256800	-1.58330800	-0.07382100
H	-2.57153700	6.49599700	1.10873700	H	6.17270300	-2.68737700	-1.48912900
H	-0.58225800	3.35043000	-1.01887400	H	3.56837800	-0.82875400	1.36275200
C	-6.25446600	3.48169400	-0.07205000	C	3.25906100	-6.32515800	0.07304300
C	-6.47659800	4.55180900	-0.94111900	C	4.28326900	-6.50277300	1.00275100
C	-7.28765100	3.05443400	0.76357400	C	2.91458300	-7.35668800	-0.80103400
C	-7.70877800	5.18942100	-0.96034400	C	4.95039600	-7.71647300	1.06120700
H	-5.68028000	4.87869000	-1.59946900	H	4.54372400	-5.69442000	1.67525800
C	-8.52225600	3.68682500	0.72529400	C	3.58703600	-8.56768800	-0.73095700
H	-7.11695900	2.22630400	1.44142800	H	2.13657900	-7.19823200	-1.53891800
C	-8.73900500	4.75942100	-0.13174500	C	4.60443200	-8.75139900	0.19848600
H	-7.86736700	6.01910000	-1.64034400	H	5.74145700	-7.85559100	1.78865200
H	-9.31576500	3.34455600	1.38035200	H	3.32391700	-9.36481300	-1.41627700
H	-9.70257600	5.25523600	-0.15490000	H	5.13109100	-9.69718900	0.24663800
C	-0.08029400	7.41660800	0.16217300	C	7.43659100	-0.45031400	-0.12470400
C	0.90609900	8.12324000	0.86926600	C	8.25792400	0.49228900	-0.76709300
C	-1.07510900	8.17249700	-0.47903600	C	8.07607000	-1.55518900	0.46355700
C	0.88939700	9.50890000	0.95256500	C	9.63682900	0.34108100	-0.82142900
H	1.69371100	7.57205200	1.37243400	H	7.80066200	1.35209100	-1.24486300
C	-1.08284200	9.55994500	-0.42439900	C	9.45584400	-1.70266400	0.43311100
H	-1.84722300	7.66087300	-1.04406000	H	7.47684100	-2.30625700	0.96849500
C	-0.10368700	10.23077500	0.29968000	C	10.24087400	-0.75478900	-0.21475800
H	1.65463500	10.02801600	1.51964100	H	10.24278200	1.07985300	-1.33509100
H	-1.85452100	10.11929700	-0.94240200	H	9.92150300	-2.55813400	0.91113400
H	-0.11284900	11.31424900	0.35284500	H	11.31895400	-0.87060200	-0.24826300
N	-5.00768200	-2.81319900	-0.04943800	N	-2.97846100	-4.86064800	0.00037500
B	5.19990900	2.98903900	-0.05091900	B	3.19719400	5.06664100	0.08810900
N	-5.00258500	2.82177600	-0.04944500	N	2.58446700	-5.07784900	0.00043700
B	-0.06044700	5.85179700	0.08810800	B	5.88033200	-0.28582300	-0.08110700
B	5.19449900	-2.99792100	-0.05101300	B	-2.78847200	5.30345700	0.08976100
B	-0.07074100	-5.85166000	0.08813300	B	-5.88298100	0.17754000	-0.07917100

Structure S15. The coordinates of both the optimized structures of B3N3L$_1$ at S$_0$ and S$_1$ in toluene.

S$_0$-optimized structure				S$_1$-optimized structure			
C	4.68069800	-3.46280700	-0.88266100	C	-3.63150800	4.73015400	0.78131500
C	3.57154800	-3.65997800	-0.05809100	C	-3.77914200	3.48867200	0.12945500
C	3.17078500	-2.61901000	0.78179700	C	-2.66977600	2.97547300	-0.57330500
C	3.85878300	-1.41699700	0.79581700	C	-1.48072500	3.66363600	-0.61885600
C	4.97298200	-1.22172000	-0.02390700	C	-1.33600900	4.89648200	0.04056400
C	5.37126300	-2.26293100	-0.86583400	C	-2.44124900	5.41504700	0.73717100
H	4.99839800	-4.25540700	-1.54982400	H	-4.45415100	5.13290500	1.35729500
H	2.32294700	-2.75871300	1.44206300	H	-2.76619500	2.04369500	-1.11461100
H	3.54311100	-0.62598000	1.46589800	H	-0.65927500	3.26771800	-1.20304100
H	6.22449300	-2.12560700	-1.51950300	H	-2.34300200	6.34683400	1.27979300
N	2.89219700	-4.90013600	-0.06038100	N	-4.96866800	2.79420200	0.18027600
C	4.97466100	1.21649700	0.02509900	C	1.09539700	4.95395200	-0.04102200
C	5.37296600	2.25779400	0.86695300	C	2.17410200	5.52184800	-0.74052900
C	3.86181200	1.41260900	-0.79631500	C	1.29907200	3.73199800	0.62290700
C	4.68376400	3.45847900	0.88205500	C	3.39575500	4.89441900	-0.78288100
H	6.22506100	2.11991200	1.52197500	H	2.03129400	6.44589700	-1.28645800
C	3.17520300	2.61542900	-0.78396100	C	2.51961900	3.10102800	0.57884500
H	3.54613100	0.62157100	-1.46636200	H	0.49815200	3.29968300	1.20987600
C	3.57600400	3.65641500	0.05585300	C	3.60264300	3.66367300	-0.12699900
H	5.00143900	4.25112700	1.54918100	H	4.19792600	5.33420800	-1.36068300
H	2.32839500	2.75573800	-1.44543100	H	2.66053300	2.17687200	1.12355100
C	7.09036700	-0.00431000	0.00432900	C	-0.16966900	7.02060300	-0.00494100
C	7.79886600	-0.96899000	0.72703000	C	-1.01325300	7.69371100	-0.88928400
C	7.80355800	0.95900900	-0.71558500	C	0.64225000	7.73740200	0.87467200
C	9.18600600	-0.97123900	0.71905400	C	-1.04181400	9.07996100	-0.88850900
H	7.25670700	-1.71523800	1.29547200	H	-1.62918900	7.12840000	-1.57841200
C	9.19065100	0.95865700	-0.70200100	C	0.60809800	9.12348400	0.86358000
H	7.26510200	1.70625800	-1.28622500	H	1.28342700	7.20557000	1.56744400
C	9.89276100	-0.00694300	0.00995300	C	-0.23251800	9.79834400	-0.01502700
H	9.71724900	-1.72795800	1.28604900	H	-1.69186200	9.60047700	-1.58196000
H	9.72558900	1.71436000	-1.26687500	H	1.23398700	9.67801100	1.55288000
N	2.89798700	4.89737500	0.05647700	N	4.82394200	3.02638100	-0.17698600
C	-3.72632200	-3.78582300	0.01891700	C	-3.75437500	-3.79100300	-0.24613700
C	-4.95429100	-3.67458400	-0.65256400	C	-3.64948000	-5.12203600	0.22289000
C	-3.26608900	-2.64612500	0.69732200	C	-2.54096300	-3.20050200	-0.67154300
C	-5.67564800	-2.48954400	-0.65376900	C	-2.43994900	-5.78340200	0.29347000
H	-5.34489000	-4.53321700	-1.18930200	H	-4.54357200	-5.62570300	0.57742900
C	-3.98484000	-1.46054200	0.69367300	C	-1.33724000	-3.87008900	-0.61962100
H	-2.32742500	-2.69466000	1.23958700	H	-2.56203300	-2.19369900	-1.07798000
C	-5.20930900	-1.34689000	0.01606800	C	-1.22097100	-5.18531900	-0.10838400
H	-6.61581100	-2.44200500	-1.19382900	H	-2.41749200	-6.78993800	0.69919300
H	-3.59323100	-0.60191900	1.22936100	H	-0.44870700	-3.37655200	-0.99884800
C	1.50243900	4.95680800	0.04979500	C	4.91221500	1.61271400	-0.04464700
C	0.73485200	3.94463800	0.64377700	C	4.08663700	0.78404000	-0.80324600
C	0.83262500	6.03040000	-0.55473400	C	5.84946200	1.04821700	0.81924900
C	-0.64562500	4.00195500	0.60694900	C	4.18430000	-0.59211100	-0.66956100
H	1.22960100	3.11563300	1.13489700	H	3.37674600	1.21982800	-1.49747500
C	-0.54860600	6.08643000	-0.54759500	C	5.95515200	-0.33153800	0.91231900
H	1.40339000	6.81495100	-1.03625200	H	6.49197000	1.69209600	1.40920100
C	-1.34284400	5.07368500	0.01981200	C	5.12090700	-1.19511100	0.18476500
H	-1.20577700	3.19994000	1.07583500	H	3.52923900	-1.22136100	-1.26187500
H	-1.03120900	6.92898900	-1.03141500	H	6.69719400	-0.75209100	1.58278500
C	-5.20768800	1.35278700	-0.01418800	C	1.47268000	-5.12324600	0.12488500
C	-3.98372000	1.46484200	-0.69297000	C	1.52762400	-3.80854800	0.64733300
C	-5.67202000	2.49613500	0.65586500	C	2.71710000	-5.65865600	-0.28680300
C	-3.26355800	2.64956600	-0.69754600	C	2.69760200	-3.08139500	0.69884400
H	-3.59364400	0.60564500	-1.22885200	H	0.61782400	-3.36175300	1.03409700
C	-4.94927700	3.68032400	0.65368700	C	3.89375700	-4.94022400	-0.21502300
H	-6.61171100	2.44982100	1.19685100	H	2.74150000	-6.66143400	-0.70161700
C	-3.72181500	3.78996300	-0.01898400	C	3.93572300	-3.60929400	0.26327000
H	-2.32533700	2.69687300	-1.24068700	H	2.67172200	-2.07748300	1.11216500
H	-5.33836100	4.53955000	1.19057900	H	4.80988500	-5.39714100	-0.57669600
C	-7.56562800	0.00435900	0.00240400	C	0.18106000	-7.49820100	-0.00508200
C	-8.29716700	-1.02410100	0.61911900	C	-0.67000400	-8.25397500	-0.83029100
C	-8.29680800	1.03370700	-0.61325600	C	1.06996400	-8.22509400	0.80605900
C	-9.68511300	-1.01951200	0.63443800	C	-0.63200900	-9.64217200	-0.85485800
C	-9.68477800	1.03085600	-0.62648800	C	1.10642900	-9.61350700	0.80037500
C	-10.38149100	0.00611000	0.00450500	C	0.25613200	-10.33008100	-0.03498600
H	-10.22552700	-1.81772800	1.13168700	H	-1.29714400	-10.19019600	-1.51479000
H	-10.22494100	1.82973600	-1.12294200	H	1.80012500	-10.13935500	1.44885600
C	-3.65163300	6.49645300	-0.05640200	C	6.64801100	-3.42310900	0.37433700
C	-4.86595800	6.63968700	-0.74722000	C	6.86001800	-4.62176200	1.07734700
C	-3.15146300	7.63086000	0.60324000	C	7.77397300	-2.83360200	-0.22599800
C	-5.53635000	7.85440800	-0.79566500	C	8.11911100	-5.19840500	1.17662400
C	-3.82993900	8.84219100	0.58317700	C	9.03306700	-3.41304100	-0.14856900
C	-5.02136900	8.95777900	-0.12408200	C	9.21033800	-4.59800800	0.55788900
H	-6.46453400	7.94064900	-1.35035700	H	8.25056900	-6.11880000	1.73592800
H	-3.42866600	9.69822200	1.11504900	H	9.87924900	-2.94022500	-0.63636800
C	3.66839800	6.08521900	0.04332400	C	6.02131800	3.75047800	-0.40089500
C	4.74227100	6.21606600	-0.83850100	C	6.29366200	4.90794900	0.33068300
C	3.37524500	7.12889400	0.92321200	C	6.94827700	3.28792500	-1.33565100
C	5.51042200	7.37179700	-0.83517100	C	7.47444500	5.60262100	0.11234700
H	4.97003900	5.40824500	-1.52401300	H	5.58437900	5.25139900	1.07476800
C	4.13785100	8.28801100	0.90801500	C	8.13062600	3.98385800	-1.53838000
H	2.54884500	7.02529800	1.61670200	H	6.73608300	2.38665900	-1.89820500
C	5.21079900	8.41507600	0.03310400	C	8.39767100	5.14437500	-0.82049400
H	6.34154400	7.46023700	-1.52607600	H	7.68041400	6.49791500	0.68794800
H	3.89895100	9.09097200	1.59654000	H	8.84416600	3.61862200	-2.26805500
C	1.49654000	-4.95815900	-0.05320900	C	-4.98811500	1.37737800	0.05139300
C	0.82593100	-6.03129800	0.55118900	C	-5.89193800	0.76579600	-0.81595500
C	0.72976500	-3.94506600	-0.64663300	C	-4.12709200	0.59214100	0.81669200
C	-0.55537200	-6.08592100	0.54463500	C	-5.92886600	-0.61770900	-0.90627900
H	1.39613200	-6.81659400	1.03216400	H	-6.56209700	1.37602700	-1.41091700
C	-0.65075800	-4.00095700	-0.60919900	C	-4.15638600	-0.78750100	0.68620500

H	1.22517400	-3.11643500	-1.13772500	H	-3.44320000	1.06442300	1.51319000
C	-1.34879400	-5.07214300	-0.02204400	C	-5.05714100	-1.43763900	-0.17212300
H	-1.03863700	-6.92815500	1.02835800	H	-6.64556200	-1.07582500	-1.57969400
H	-1.21030400	-3.19822900	-1.07758500	H	-3.47453700	-1.38254500	1.28363300
C	3.66114000	-6.08896400	-0.04851500	C	-6.19959100	3.46038000	0.40074500
C	3.36677000	-7.13113000	-0.92977400	C	-7.10812800	2.95149600	1.32959700
C	4.73455200	-6.22239700	0.83348000	C	-6.52301300	4.60622000	-0.32852200
C	4.12775400	-8.29132800	-0.91580400	C	-8.32273000	3.59041900	1.52905500
H	2.54069500	-7.02551700	-1.62334700	H	-6.85667500	2.05913100	1.89014300
C	5.50109900	-7.37919000	0.82892400	C	-7.73606900	5.24383400	-0.11337900
H	4.96321400	-5.41575500	1.52008200	H	-5.82768200	4.98496700	-1.06865600
C	5.20027600	-8.42096200	-0.04074700	C	-8.64087300	4.73961800	0.81378600
H	3.88793500	-9.09311400	-1.60537600	H	-9.02166900	3.18968700	2.25423400
H	6.33188400	-7.46965900	1.51997100	H	-7.98122000	6.13030500	-0.68728600
C	-3.65907100	-6.49243600	0.05503300	C	-6.47271500	-3.73697800	-0.36554500
C	-4.87304000	-6.63470600	0.74667700	C	-6.62614900	-4.94282700	-1.07156800
C	-3.16058500	-7.62705500	-0.60551300	C	-7.62692300	-3.20299100	0.23318800
C	-5.54469300	-7.84873600	0.79502400	C	-7.85624200	-5.57830100	-1.17513300
C	-3.84035000	-8.83766500	-0.58555300	C	-8.85696300	-3.84128400	0.15142500
C	-5.03139300	-8.95232200	0.12251000	C	-8.97623000	-5.03183200	-0.55782300
H	-6.47255600	-7.93425400	1.35036600	H	-7.94249100	-6.50260600	-1.73679300
H	-3.44038700	-9.69386400	-1.11814100	H	-9.72578200	-3.41011400	0.63803700
N	5.68159400	-0.00306900	0.00150000	N	-0.13643400	5.60175300	-0.00098600
B	-2.89587300	5.12500500	-0.01612900	B	5.22652400	-2.76771400	0.28333900
B	-6.00284700	0.00343600	0.00140400	B	0.14339800	-5.92147600	0.00562500
B	-2.90188000	-5.12178000	0.01486500	B	-5.08454800	-3.01383200	-0.26980700
H	5.80900100	9.31899100	0.02920600	H	9.32284600	5.68492400	-0.98289400
H	-5.56005300	-9.89925200	0.14828400	H	-9.93733000	-5.52976400	-0.63106500
H	-2.23008300	-7.55150900	-1.15835500	H	-7.55218900	-2.27189700	0.78605700
H	-5.29022100	-5.77771000	1.26517400	H	-5.76154300	-5.38007200	-1.55956000
H	5.79722100	-9.32570900	-0.03779200	H	-9.59104600	5.23577300	0.97355600
H	10.97662700	-0.00794300	0.01211700	H	-0.25657200	10.88172500	-0.01903200
H	-7.76460700	-1.83353100	1.10710000	H	-1.37085100	-7.73488100	-1.47665800
H	-11.46633200	0.00679000	0.00532700	H	0.28559600	-11.41463300	-0.04706600
H	-5.28443800	5.78288100	-1.26498500	H	6.01824200	-5.10059500	1.56619000
H	-5.54903300	9.90526100	-0.14994200	H	10.19419100	-5.04984200	0.62779300
H	-2.22065900	7.55457300	1.15547000	H	7.65361700	-1.90620600	-0.77701100
H	-7.76397400	1.84247100	-1.10203900	H	1.74317100	-7.68339800	1.46313000

Structure S16. The coordinates of both the optimized structures of B2N4L$_1$ at S$_0$ and S$_1$ in toluene.

S_0-optimized structure				S_1-optimized structure			
C	-5.35893600	-2.30601400	-0.80787100	C	-5.43121500	-2.46464200	-0.88141400
C	-4.98421700	-1.25079000	0.02478100	C	-5.04044600	-1.26649700	-0.25560900
C	-3.87727500	-1.41864200	0.85886900	C	-3.87918100	-1.28733000	0.53858000
C	-3.16276300	-2.60504100	0.85581100	C	-3.14671600	-2.44400100	0.69438200
C	-3.53783900	-3.66280900	0.02322800	C	-3.53696000	-3.63015300	0.06138500
C	-4.64740100	-3.49381900	-0.80914100	C	-4.69614400	-3.61981200	-0.72359000
H	-6.20927400	-2.18887900	-1.46944300	H	-6.30457100	-2.47577400	-1.52058300
H	-3.58241200	-0.61624500	1.52493600	H	-3.57046600	-0.38820500	1.05535500
H	-2.31425900	-2.72245100	1.51955300	H	-2.27517900	-2.44009400	1.33842800
H	-4.94652900	-4.29737900	-1.47153300	H	-5.00361100	-4.52248500	-1.23782600
C	-1.40457300	-4.85116000	0.04082900	C	-1.41183400	-4.80365900	0.22760900
C	-0.68776700	-5.75845500	0.82540000	C	-0.68751900	-5.76183900	0.96222300
C	-0.68978800	-3.93203800	-0.73043600	C	-0.68754300	-3.84468300	-0.50609900
C	0.69639100	-5.75745400	0.82536300	C	0.68635700	-5.76197700	0.96219300
H	-1.22277100	-6.46689900	1.44682400	H	-1.21845000	-6.48858700	1.56351900
C	0.69565100	-3.93102000	-0.73046100	C	0.68670500	-3.84482000	-0.50612700
H	-1.22496700	-3.22501200	-1.35339100	H	-1.21869500	-3.12224000	-1.11257000
C	1.41182900	-4.84909100	0.04077400	C	1.41083500	-4.80394000	0.22755100
H	1.23245600	-6.46510600	1.44677500	H	1.21716700	-6.48883200	1.56346900
H	1.22976100	-3.22320400	-1.35343300	H	1.21797700	-3.12248300	-1.11262000
C	-3.50027200	-6.10235100	-0.03437400	C	-3.49876100	-6.03551800	0.38572700
C	-4.67776800	-6.29230800	0.69500700	C	-4.55335100	-6.11342500	1.29576600
C	-3.01550900	-7.15166500	-0.82057800	C	-3.14618200	-7.15339000	-0.37152800
C	-5.35522900	-7.50097800	0.62897700	C	-5.24524700	-7.30571700	1.44669700
H	-5.05720700	-5.48794900	1.31402100	H	-4.81794800	-5.24266800	1.88370300
C	-3.69095200	-8.36264300	-0.86566400	C	-3.83991300	-8.34312800	-0.20696900
H	-2.10815300	-7.01213200	-1.39608400	H	-2.33940100	-7.08031900	-1.09139600
C	-4.86631100	-8.54598200	-0.14655800	C	-4.89078600	-8.42395700	0.69986600
H	-6.26698300	-7.62926200	1.20215000	H	-6.05985900	-7.36272400	2.15937100
H	-3.29945500	-9.16477800	-1.48179600	H	-3.56574800	-9.20712200	-0.80130900
H	-5.39471900	-9.49131200	-0.18984900	H	-5.43285100	-9.35427800	0.82248300
C	-1.43160700	5.09070900	-0.03980100	C	-1.45760600	5.02271800	0.20382300
C	-0.69830000	6.06512400	-0.73574800	C	-0.68687400	6.19711900	-0.02868000
C	-0.69628300	4.12613300	0.66690800	C	-0.68632800	3.85306500	0.45405900
C	0.68915400	6.06612700	-0.73588300	C	0.68817400	6.19698100	-0.02860500
H	-1.22893400	6.83053800	-1.29315500	H	-1.20638000	7.12363800	-0.25324200
C	0.69023000	4.12713800	0.66677200	C	0.68710700	3.85292900	0.45414200
H	-1.22525700	3.36431100	1.23029400	H	-1.20512300	2.92749500	0.68405500
C	1.42401800	5.09279800	-0.04006000	C	1.45864800	5.02242900	0.20400100
H	1.21857200	6.83233000	-1.29336700	H	1.20788700	7.12339500	-0.25312200
H	1.22041900	3.36608500	1.23005800	H	1.20569100	2.92725600	0.68419900
C	3.54329600	-3.65751400	0.02311700	C	3.53619000	-3.63085500	0.06126900
C	3.16654400	-2.60024200	0.85557100	C	3.14621900	-2.44464300	0.69432000
C	4.65268700	-3.48693500	-0.80915000	C	4.69534400	-3.62072900	-0.72375500
C	3.87926500	-1.41277300	0.85859700	C	3.87891700	-1.28811800	0.53852300
H	2.31814100	-2.71887000	1.51922700	H	2.27471300	-2.44057700	1.33840700
C	5.36244800	-2.29806600	-0.80789700	C	5.43064600	-2.46570600	-0.88157300
H	4.95308700	-4.29009900	-1.47144600	H	5.00260500	-4.52345100	-1.23802800
C	4.98605000	-1.24333000	0.02461500	C	5.04015200	-1.26750000	-0.25571200
H	3.58312700	-0.61076300	1.52456200	H	3.57041300	-0.38894600	1.05534400
H	6.21269000	-2.17970800	-1.46937500	H	6.30397800	-2.47700000	-1.52077200
C	3.74322300	3.71322800	-0.04630200	C	3.76242000	3.64022800	-0.00726700
C	3.18126000	2.55847500	-0.62033200	C	3.30060000	2.64108900	-0.88247000
C	5.00985100	3.55245800	0.54329900	C	4.94953400	3.35138900	0.68868400
C	3.83050500	1.33854100	-0.62282200	C	3.95665100	1.43051700	-1.03834500
H	2.21318800	2.62650000	-1.10509600	H	2.40744000	2.82822100	-1.46866300
C	5.65922800	2.33245700	0.58684100	C	5.60540600	2.13541500	0.56765200
H	5.48554400	4.40493300	1.01669600	H	5.35644300	4.09551800	1.36564000
C	5.08056200	1.19915000	-0.00286100	C	5.10465700	1.16081200	-0.29416900
H	3.37306400	0.48300800	-1.10436300	H	3.58353900	0.68647800	-1.73404100
H	6.61628600	2.24684900	1.08669200	H	6.50200800	1.93174100	1.14300400
C	3.78347600	6.42698700	-0.03579900	C	3.84543400	6.31569700	0.35090800
C	3.27738000	7.56389100	0.61504400	C	3.45189500	7.35885000	1.20777600
C	5.02211100	6.55760000	-0.68465300	C	5.06108600	6.49495100	-0.33287000
C	3.97769400	8.76242000	0.63557800	C	4.21584600	8.50752800	1.36886100
H	2.32126100	7.50035300	1.12401600	H	2.52997700	7.25548200	1.77069600
C	5.71614100	7.76020400	-0.69207700	C	5.82512800	7.64567500	-0.19042600
H	5.43902200	5.70230400	-1.20603800	H	5.40820000	5.71261900	-1.00096900
C	5.19753700	8.86393100	-0.02423500	C	5.40568400	8.65935600	0.66486300
H	3.57046100	9.62050800	1.15951600	H	3.88324800	9.28717400	2.04685500
H	6.66360000	7.83726600	-1.21466100	H	6.75150200	7.75319100	-0.74593000
H	5.74205500	9.80219200	-0.01976200	H	6.00244300	9.55759400	0.78429800
C	7.13957800	-0.11737200	0.07851400	C	7.14110300	-0.10705500	-0.72121300
C	7.92318500	0.65570000	-0.78079600	C	7.65191800	0.77842600	-1.67249900
C	7.76368200	-0.98889500	0.97265900	C	8.01231700	-0.96977900	-0.05118900
C	9.30693600	0.56549700	-0.73281200	C	9.01042500	0.79141100	-1.95146600
H	7.44214000	1.32457800	-1.48481000	H	6.97924300	1.45411700	-2.18692700
C	9.14762300	-1.08539600	1.00197600	C	9.36752100	-0.95832200	-0.34738000
H	7.15804800	-1.58850600	1.64203100	H	7.62492600	-1.63950400	0.70766800
C	9.92711100	-0.30677000	0.15448200	C	9.87484500	-0.07867300	-1.29673200
H	9.90283800	1.17199700	-1.40587800	H	9.39327100	1.48380300	-2.69275900
H	9.61849700	-1.76652000	1.70216800	H	10.03308100	-1.63078500	0.18242500
H	11.00814300	-0.38013300	0.18383100	H	10.93551300	-0.06649300	-1.51930800
C	3.50942800	-6.09711100	-0.03416400	C	3.49752100	-6.03621700	0.38558400
C	3.02641400	-7.14723600	-0.82035500	C	3.14467900	-7.15401700	-0.37165400
C	4.68707500	-6.28519600	0.69545700	C	4.55213700	-6.11434000	1.29557500
C	3.70371700	-8.35718400	-0.86520200	C	3.83817400	-8.34389700	-0.20712500
H	2.11895000	-7.00914200	-1.39604000	H	2.33788000	-7.08078200	-1.09148400
C	5.36639700	-7.49283400	0.62966900	C	5.24379500	-7.30677300	1.44647500
H	5.06516600	-5.48019100	1.31445600	H	4.81693900	-5.24363600	1.88350000
C	4.87922100	-8.53865800	-0.14585800	C	4.88907100	-8.42494100	0.69966200
H	3.31356200	-9.15997300	-1.48133300	H	3.56380300	-9.20783600	-0.80145100
H	6.27824200	-7.61966600	1.20302000	H	6.05842800	-7.36394500	2.15911300
H	5.40908700	-9.48317800	-0.18897100	H	5.43095000	-9.35537400	0.82225600

C	-5.08231000	1.19153700	-0.00255900	C	-5.10442600	1.16182500	-0.29417700
C	-5.66263900	2.32394900	0.58722800	C	-5.60495200	2.13659600	0.56758400
C	-3.83242600	1.33280100	-0.62243900	C	-3.95636900	1.43122900	-1.03838400
C	-5.01507400	3.54491600	0.54376200	C	-4.94882000	3.35243900	0.68851700
H	-6.61957600	2.23690200	1.08706500	H	-6.50159400	1.93315800	1.14295800
C	-3.18498600	2.55370100	-0.61987600	C	-3.30005400	2.64166900	-0.88260200
H	-3.37369200	0.47796200	-1.10398900	H	-3.58342900	0.68706200	-1.73403600
C	-3.74868600	3.70759700	-0.04583200	C	-3.76165300	3.64097300	-0.00747000
H	-5.49203800	4.39665300	1.01720500	H	-5.35556700	4.09670900	1.36541600
H	-2.21700300	2.62317000	-1.10461000	H	-2.40686500	2.82857000	-1.46882500
C	-7.13941500	-0.12800700	0.07844500	C	-7.14115700	-0.10562100	-0.72111300
C	-7.92402500	0.64405100	-0.78086800	C	-7.65179500	0.77992400	-1.67243500
C	-7.76238400	-1.00063900	0.97229900	C	-8.01254700	-0.96812300	-0.05103300
C	-9.30764500	0.55175100	-0.73316600	C	-9.01030400	0.79318900	-1.95138200
H	-7.44386200	1.31377800	-1.48467600	H	-6.97898100	1.45544300	-2.18690800
C	-9.14618300	-1.09924400	1.00132000	C	-9.36775300	-0.95638800	-0.34720500
H	-7.15598200	-1.59947900	1.64166400	H	-7.62528900	-1.63789600	0.70784900
C	-9.92668200	-0.32162400	0.15383600	C	-9.87490100	-0.07667500	-1.29659100
H	-9.90432800	1.15747800	-1.40623700	H	-9.39301200	1.48562700	-2.69270300
H	-9.61615800	-1.78122400	1.70128500	H	-10.03345000	-1.62868100	0.18264400
H	-11.00760800	-0.39662100	0.18295400	H	-10.93557000	-0.06427700	-1.51915100
C	-3.79312100	6.42129000	-0.03534900	C	-3.84411300	6.31648300	0.35055200
C	-3.28867500	7.55900000	0.61537000	C	-3.45017900	7.35969100	1.20717000
C	-5.03206600	6.54995900	-0.68399600	C	-5.05986600	6.49587600	-0.33300800
C	-3.99085700	8.75643700	0.63600200	C	-4.21387000	8.50854400	1.36824300
H	-2.33237800	7.49695600	1.12418800	H	-2.52814700	7.25623400	1.76988900
C	-5.72797400	7.75148000	-0.69131900	C	-5.82365100	7.64677200	-0.19057500
H	-5.44773500	5.69400800	-1.20529400	H	-5.40727100	5.71351400	-1.00092000
C	-5.21097300	8.85602900	-0.02359600	C	-5.40382900	8.66049700	0.66447800
H	-3.58486300	9.61517400	1.15984100	H	-3.88097000	9.28823100	2.04604100
H	-6.67565100	7.82705700	-1.21372500	H	-6.75011900	7.75438900	-0.74590300
H	-5.75696100	9.79343500	-0.01904400	H	-6.00038400	9.55887300	0.78390000
N	-2.81478600	-4.87210600	0.02424300	N	-2.79663600	-4.81399800	0.22233500
B	2.99302700	5.07499900	-0.04404300	B	2.98994300	5.00461000	0.18172600
N	2.82206000	-4.86790100	0.02418600	N	2.79563500	-4.81455600	0.22222600
N	-5.72751000	-0.04670200	0.03213000	N	-5.76042500	-0.09531100	-0.42096600
N	5.72755700	-0.03814500	0.03196900	N	5.76038000	-0.09646100	-0.42104000
B	-3.00058700	5.07052700	-0.04364000	B	-2.98889800	5.00521700	0.18137900

Structure S17. The coordinates of both the optimized structures of B1N5L_1 at S_0 and S_1 in toluene.

S_0-optimized structure				S_1-optimized structure			
C	-3.51491500	-4.63244000	-0.72645000	C	3.13337700	-4.85900500	1.16299400
C	-3.68037800	-3.49063900	0.06058100	C	3.39966200	-3.73787500	0.36094800
C	-2.59553000	-3.04280300	0.81728200	C	2.35926700	-3.21810200	-0.42600200
C	-1.38691200	-3.71938200	0.79525300	C	1.10965900	-3.79283500	-0.41335400
C	-1.22375400	-4.86673700	0.01477500	C	0.83882000	-4.91916900	0.38587400
C	-2.30723700	-5.30866200	-0.74962900	C	1.88351800	-5.43464300	1.17713900
H	-4.33950700	-4.98663100	-1.33396700	H	3.91145100	-5.25729100	1.80243900
H	-2.70663100	-2.16550700	1.44386200	H	2.55257200	-2.37804900	-1.08154900
H	-0.56410900	-3.36503200	1.40488600	H	0.33448100	-3.39451100	-1.05501600
H	-2.19705800	-6.18583900	-1.37611100	H	1.69566600	-6.27882700	1.82786400
C	1.22178000	-4.86714400	-0.01482900	C	-1.56841500	-4.72502500	0.09077800
C	2.30499000	-5.31006900	0.74936700	C	-2.49932600	-5.17695900	-0.84526500
C	1.38548700	-3.71930800	-0.79446400	C	-1.80224500	-3.50974900	0.73445100
C	3.51294500	-4.63432200	0.72682600	C	-3.62936200	-4.43524900	-1.13380100
H	2.19437100	-6.18766900	1.37518400	H	-2.32545400	-6.11199900	-1.36522300
C	2.59438200	-3.04320200	-0.81588100	C	-2.93098000	-2.76437900	0.44935100
H	0.56289800	-3.36419500	-1.40394300	H	-1.10061500	-3.15279200	1.47979800
C	3.67897000	-3.49201400	-0.05937200	C	-3.87330800	-3.21215500	-0.48911200
H	4.33731200	-4.98929000	1.33419000	H	-4.32378400	-4.79677000	-1.88142200
H	2.70589800	-2.16551800	-1.44183800	H	-3.09833200	-1.83165900	0.97225100
C	-0.00131100	-6.97811900	-0.00149500	C	-0.59415800	-6.86954400	0.69301300
C	-0.89426800	-7.68841100	0.80574300	C	0.23090300	-7.83491600	0.11232000
C	0.89148100	-7.68732200	-0.80986400	C	-1.61834100	-7.26564600	1.55447100
C	-0.89670800	-9.07563500	0.79434200	C	0.04103700	-9.17707500	0.40728900
H	-1.58468900	-7.14675100	1.44146700	H	1.01135900	-7.52971700	-0.57473100
C	0.89353300	-9.07456700	-0.80076100	C	-1.80748400	-8.61102700	1.83334600
H	1.58205600	-7.14480100	-1.44468400	H	-2.25838100	-6.51529300	2.00295400
C	-0.00169000	-9.77877600	-0.00379800	C	-0.97810300	-9.57212000	1.26634000
H	-1.59665100	-9.60977300	1.42763500	H	0.68499800	-9.91905600	-0.05116100
H	1.59334100	-9.60785100	-1.43492200	H	-2.60381800	-8.90786200	2.50631600
H	-0.00184200	-10.86268500	-0.00469300	H	-1.12781700	-10.62216100	1.48893600
C	-3.79767500	3.54574100	-0.08728100	C	4.02049400	3.17322800	-0.30474600
C	-3.68300600	4.79063000	-0.72173800	C	4.09022300	4.31625000	0.49627300
C	-2.67811000	3.03836500	0.58595000	C	2.81540000	2.86307300	-0.94535800
C	-2.49424800	5.49618200	-0.66785700	C	2.97752100	5.12246600	0.64954000
H	-4.52975300	5.19742200	-1.26118800	H	5.01983000	4.56024900	1.00027900
C	-1.48765200	3.74148000	0.59236100	C	1.70525800	3.66202600	-0.75263500
H	-2.74931100	2.08923300	1.10292700	H	2.76268800	1.99551400	-1.59552600
C	-1.34762900	4.99627400	-0.02607900	C	1.72733500	4.83288300	0.04558700
H	-2.44274800	6.45608400	-1.17122200	H	3.05879500	5.99745800	1.28572600
H	-0.64185300	3.31592200	1.12198100	H	0.78343100	3.39698600	-1.26001500
C	4.94099600	-1.40497800	-0.03054300	C	-4.97013800	-1.04345400	-0.55258600
C	4.08920700	-0.71255000	0.83458100	C	-4.05915500	-0.23371300	-1.22894100
C	5.81243600	-0.66822300	-0.83745600	C	-5.84126200	-0.45449100	0.36063500
C	4.10805100	0.67085900	0.88980800	C	-4.00432500	1.12719400	-0.98355900
H	3.41794500	-1.26653800	1.48004700	H	-3.38917800	-0.67744300	-1.95710300
C	5.82794800	0.71537700	-0.78315800	C	-5.78920400	0.90631000	0.61162900
H	6.47255600	-1.18512400	-1.52373700	H	-6.55177200	-1.07402300	0.89648500
C	4.97390800	1.40535400	0.07788100	C	-4.86110400	1.72355400	-0.04790100
H	3.45037100	1.18999200	1.57723000	H	-3.29031900	1.74169600	-1.51736900
H	6.49971600	1.27057800	-1.42743000	H	-6.45741900	1.33887000	1.34580000
C	1.34948500	4.99574500	0.02552700	C	-0.94716100	5.02610500	0.20387800
C	1.48905100	3.74060900	-0.59231300	C	-1.14137300	3.73645100	0.74506700
C	2.49627900	5.49551800	0.66710400	C	-2.10913700	5.62931100	-0.32492000
C	2.67924200	3.03704900	-0.58554600	C	-2.37472000	3.10999200	0.77784300
H	0.64310400	3.31512200	-1.12175700	H	-0.29226600	3.22471000	1.18745900
C	3.68476700	4.78953900	0.72135200	C	-3.34355800	5.00020400	-0.33948000
H	2.44513100	6.45567700	1.17001200	H	-2.03051800	6.61740800	-0.76732600
C	3.79898700	3.54430900	0.08747500	C	-3.49706900	3.72938100	0.22071500
H	2.75009000	2.08765200	-1.10208400	H	-2.47726000	2.13344700	1.23902700
H	4.53164900	5.19626400	1.26063800	H	-4.20135900	5.49298500	-0.78491000
C	0.00140100	7.34534700	-0.00085600	C	0.57745700	7.26237000	0.43681600
C	-1.00442300	8.07832600	0.64939400	C	1.68993300	8.00848300	-0.01590700
C	1.00750400	8.07762900	-0.65145600	C	-0.42410200	8.01300600	1.09452100
C	-1.00078300	9.46684100	0.66296800	C	1.79855400	9.37730200	0.17451200
H	-1.79651900	7.54571000	1.16544700	H	2.48247200	7.49212900	-0.54852000
C	1.00439700	9.46613700	-0.66570000	C	-0.32286500	9.38159800	1.29119400
H	1.79938900	7.54446000	-1.16726200	H	-1.29822900	7.49459200	1.47567600
C	0.00194000	10.16418800	-0.00153300	C	0.79201100	10.08036200	0.83329500
H	-1.78246100	10.00682400	1.18667600	H	2.67067600	9.90509200	-0.20032000
H	1.78627900	10.00556900	-1.18967100	H	-1.11594600	9.91002900	1.81213500
H	0.00214800	11.24907500	-0.00179200	H	0.87400400	11.15137000	0.98493000
C	6.24329000	3.47536200	0.21171700	C	-5.90070500	3.82211300	0.63866600
C	6.51667000	4.59079100	-0.58358200	C	-5.81778500	4.73733100	1.69172100
C	7.22995100	2.99833800	1.07716800	C	-7.12296500	3.66101800	-0.02148800
C	7.74929200	5.22217000	-0.49982300	C	-6.93266200	5.47033100	2.07170500
H	5.75965000	4.95813800	-1.26642800	H	-4.87311700	4.87278000	2.20440800
C	8.46587800	3.62568000	1.14147500	C	-8.23631800	4.38646800	0.37651600
H	7.02123000	2.13473300	1.69759800	H	-7.19278400	2.96654500	-0.85065800
C	8.73218000	4.74298000	0.35869900	C	-8.15004800	5.29796600	1.42279400
H	7.94624600	6.08720900	-1.12351000	H	-6.84782900	6.17631100	2.89072900
H	9.22108200	3.24333500	1.81937600	H	-9.17514500	4.24826600	-0.14896300
H	9.69640800	5.23474800	0.41593000	H	-9.01945700	5.87040400	1.72525300
C	6.11555100	-3.54112700	-0.20191900	C	-6.20684600	-3.02442300	-1.23218700
C	6.18541200	-4.67312700	-1.01940000	C	-6.68148500	-4.22583200	-0.69779200
C	7.25540200	-3.14779600	0.50599300	C	-6.96621100	-2.36799100	-2.20398100
C	7.36624700	-5.39466700	-1.11721700	C	-7.87897600	-4.76481100	-1.14547300
H	5.31011000	-4.98250600	-1.57807600	H	-6.11317500	-4.73131400	0.07403400
C	8.43605700	-3.86625200	0.38757100	C	-8.16966800	-2.90739400	-2.63384500
H	7.20970400	-2.27757900	1.15000100	H	-6.60705100	-1.43287800	-2.61695100
C	8.50069500	-4.99602700	-0.41988700	C	-8.63189400	-4.11065700	-2.11367300
H	7.40048600	-6.26966200	-1.75719500	H	-8.23304200	-5.69687900	-0.71871700
H	9.30929000	-3.54545800	0.94515300	H	-8.74510100	-2.38429400	-3.38963600
H	9.42322400	-5.55869600	-0.50407300	H	-9.57058900	-4.53134700	-2.45514800

C	-4.94155700	-1.40310700	0.03209000	C	4.82494000	-1.78781300	0.16741100
C	-5.81268700	-0.66582400	0.83887300	C	5.86635300	-1.27303300	-0.62415100
C	-4.08948400	-0.71119400	-0.83316200	C	3.93651900	-0.88276100	0.77250400
C	-5.82766100	0.71776200	0.78426800	C	5.99464600	0.07890000	-0.82828600
H	-6.47299700	-1.18232100	1.52527700	H	6.54771900	-1.95376600	-1.11965500
C	-4.10779300	0.67221300	-0.88869400	C	4.06407400	0.47001600	0.57659200
H	-3.41842900	-1.26557500	-1.47850700	H	3.15605600	-1.25611700	1.42448900
C	-4.97337500	1.40722400	-0.07695200	C	5.08993600	0.99462400	-0.24280200
H	-6.49920700	1.27337100	1.42842100	H	6.77195300	0.44153100	-1.48734800
H	-3.44990700	1.19093300	-1.57623100	H	3.38199600	1.14875100	1.06976700
C	-6.11690800	-3.53883800	0.20381600	C	5.81782000	-3.98391000	0.43981700
C	-7.25684700	-3.14491600	-0.50362800	C	6.87244200	-3.59796300	1.26924100
C	-6.18688000	-4.67113800	1.02088400	C	5.90107600	-5.17240400	-0.28820600
C	-8.43767800	-3.86307600	-0.38513800	C	8.00067300	-4.39794700	1.36501900
H	-7.21109800	-2.27447700	-1.14733100	H	6.79551700	-2.68143400	1.84175600
C	-7.36789400	-5.39236900	1.11876200	C	7.03128100	-5.96782600	-0.17858100
H	-5.31150900	-4.98098600	1.57919100	H	5.08794900	-5.45757900	-0.94476700
C	-8.50242800	-4.99313600	0.42190800	C	8.08444200	-5.58469700	0.64501500
H	-9.31097100	-3.54181200	-0.94235700	H	8.81364100	-4.09699300	2.01546400
H	-7.40219900	-6.26759900	1.75841600	H	7.09393800	-6.88600000	-0.75104300
H	-9.42509900	-5.55556500	0.50614300	H	8.96716600	-6.20818500	0.72451600
C	-6.24199800	3.47766800	-0.21141900	C	6.35390500	2.95037100	-0.96079400
C	-6.51489300	4.59360300	0.58334100	C	6.28488400	3.88171600	-1.99892600
C	-7.22888700	3.00063200	-1.07660400	C	7.58765500	2.66845500	-0.36834900
C	-7.74725600	5.22544800	0.49931800	C	7.43958600	4.50232500	-2.44926000
H	-5.75769100	4.96097600	1.26597200	H	5.32415600	4.11329600	-2.44243500
C	-8.46456000	3.62845200	-1.14116900	C	8.73786000	3.29121200	-0.83073000
H	-7.02055200	2.13664300	-1.69662700	H	7.63648900	1.97494400	0.46317300
C	-8.73037600	4.74624300	-0.35893100	C	8.67122100	4.20863500	-1.87281800
H	-7.94382600	6.09087500	1.12258700	H	7.37510200	5.22013600	-3.25912500
H	-9.21994300	3.24608600	-1.81886000	H	9.68903700	3.06986000	-0.35958300
H	-9.69440400	5.23837800	-0.41636500	H	9.57041500	4.69968800	-2.22639400
N	-0.00112200	-5.56754600	-0.00033800	N	-0.41832400	-5.49727200	0.39192700
B	0.00108700	5.77666200	-0.00047300	B	0.45730800	5.71959400	0.22403400
N	4.91557400	-2.81165700	-0.08993400	N	-5.00088200	-2.44805900	-0.77976300
N	-4.91670900	-2.80976900	0.09180100	N	4.66987700	-3.16246100	0.33423400
N	4.99420600	2.81942300	0.13012100	N	-4.76559400	3.09011100	0.23492000
N	-4.99317200	2.82128300	-0.12954400	N	5.16862900	2.34233900	-0.48455500

Structure S18. The coordinates of both the optimized structures of N6L$_1$ at S$_0$ and S$_1$ in toluene.

S$_0$-optimized structure				S$_1$-optimized structure			
C	4.88657000	3.13481200	0.81002500	C	5.66830500	-1.47353100	-0.75091900
C	3.77794500	3.39243800	-0.00026100	C	4.64120400	-2.06259900	0.00967300
C	3.28907900	2.35988100	-0.80380600	C	3.80320200	-1.21730400	0.75898700
C	3.89262300	1.11325400	-0.80235400	C	3.99073400	0.14790600	0.75867400
C	5.00512100	0.85778900	0.00267700	C	5.02561900	0.73543400	0.00900800
C	5.48988600	1.88880000	0.81149800	C	5.85579300	-0.10924300	-0.75111700
H	5.27108000	3.91680700	1.45418900	H	6.30950100	-2.10165400	-1.35805000
H	2.43673200	2.54185300	-1.44784600	H	3.01563200	-1.65026100	1.36436600
H	3.50727200	0.33059700	-1.44524100	H	3.34919400	0.77738100	1.36397600
H	6.34111700	1.70673700	1.45691300	H	6.64263200	0.32250800	-1.35837500
C	4.82730100	-1.57524400	-0.00170300	C	4.10781400	2.98733900	-0.01109600
C	5.15814700	-2.66446600	-0.81172600	C	4.11072900	4.17223600	0.74819400
C	3.68862700	-1.66750500	0.80186700	C	2.95666400	2.68285500	-0.75962300
C	4.38029100	-3.80966500	-0.81286200	C	3.02233300	5.01598100	0.74822900
H	6.02754500	-2.60685700	-1.45603400	H	4.97519500	4.41463800	1.35497500
C	2.91033700	-2.81320000	0.80072300	C	1.86749100	3.52711500	-0.75951200
H	3.42047300	-0.83809800	1.44573100	H	2.93803900	1.78366500	-1.36403100
C	3.24502900	-3.90471800	-0.00397800	C	1.87561200	4.71795000	-0.01112900
H	4.64780700	-4.63819900	-1.45804600	H	3.04152100	5.91380000	1.35471700
H	2.03995700	-2.87040300	1.44377100	H	1.00149400	3.28488800	-1.36410700
C	7.02665000	-0.51329700	0.00496300	C	6.51810300	2.65644300	0.00014600
C	7.80717700	0.37649700	-0.73965900	C	7.51372200	2.08492100	0.79729400
C	7.66511100	-1.50757500	0.75274900	C	6.83212200	3.76163800	-0.79564000
C	9.19061500	0.27572700	-0.72607100	C	8.79802400	2.60826800	0.79021100
H	7.32311600	1.14572900	-1.32952500	H	7.27078000	1.23454200	1.42314600
C	9.04843100	-1.61058800	0.74535700	C	8.11629600	4.28519300	-0.78561000
H	7.07085800	-2.19734000	1.34016700	H	6.06506300	4.20021600	-1.42261600
C	9.82225000	-0.71919600	0.01120500	C	9.10765000	3.71197500	0.00315300
H	9.77801300	0.97530000	-1.31083800	H	9.55916500	2.15619000	1.41659200
H	9.52435200	-2.38852300	1.33247700	H	8.34564000	5.14113800	-1.41076000
C	-3.24509400	3.90473800	0.00396900	C	-1.87561400	-4.71795100	0.01113800
C	-4.38022300	3.80944700	0.81301200	C	-3.02233500	-5.01598300	-0.74822000
C	-2.91052200	2.81344900	-0.80109500	C	-1.86749300	-3.52711400	0.75952000
C	-5.15807400	2.66424400	0.81167000	C	-4.11073000	-4.17223800	-0.74818700
H	-4.64763800	4.63779400	1.45847900	H	-3.04152200	-5.91380200	-1.35470700
C	-3.68880300	1.66774700	-0.80243500	C	-2.95666600	-2.68285400	0.75962900
H	-2.04024000	2.87083800	-1.44425800	H	-1.00149700	-3.28488700	1.36411500
C	-4.82735300	1.57525800	0.00128000	C	-4.10781500	-2.98733900	0.01110000
H	-6.02737400	2.60644500	1.45609400	H	-4.97519500	-4.41464000	-1.35496900
H	-3.42074800	0.83852100	-1.44657500	H	-2.93804100	-1.78366400	1.36403500
C	1.04906800	-4.96751600	0.00116000	C	-0.53385100	5.04865500	0.00751800
C	0.39947900	-4.02750900	-0.80212000	C	-0.84650400	3.89961900	0.75598400
C	0.27147400	-5.79908200	0.81102000	C	-1.55811700	5.64442800	-0.75169700
C	-0.98189800	-3.92678600	-0.80088000	C	-2.12272700	3.37972800	0.75628900
H	0.98345800	-3.38017900	-1.44579600	H	-0.07741500	3.43329000	1.36033000
C	-1.10925200	-5.69838000	0.81235100	C	-2.83348800	5.12485500	-0.75142700
H	0.75625600	-6.52334900	1.45502900	H	-1.33514800	6.51424800	-1.35831400
C	-1.75955000	-4.76270300	0.00379900	C	-3.14962100	3.98314800	0.00823300
H	-1.46683200	-3.20150500	-1.44366500	H	-2.34662300	2.50878400	1.36088600
H	-1.69275600	-6.34469500	1.45740800	H	-3.60115100	5.59101200	-1.35777700
C	-5.00516600	-0.85776800	-0.00351100	C	-5.02562000	-0.73543500	-0.00900700
C	-3.89264900	-1.11333500	0.80145400	C	-3.99073300	-0.14790700	-0.75866900
C	-5.48991900	-1.88867000	-0.81248100	C	-5.85579700	0.10924400	0.75111500
C	-3.28912400	-2.35997100	0.80275900	C	-3.80320000	1.21730400	-0.75898200
H	-3.50728300	-0.33076500	1.44443900	H	-3.34919100	-0.77738100	-1.36396900
C	-4.88660800	-3.13468500	-0.81117600	C	-5.66830800	1.47353200	0.75091700
H	-6.34115200	-1.70652400	-1.45787000	H	-6.64263700	-0.32250800	1.35837100
C	-3.77800400	-3.39243800	-0.00089800	C	-4.64120500	2.06259900	-0.00967200
H	-2.43678200	-2.54202200	1.44678000	H	-3.01562900	1.65026100	-1.36436000
H	-5.27112800	-3.91658300	-1.45545100	H	-6.30950700	2.10165500	1.35804600
C	-7.02670600	0.51333600	-0.00508400	C	-6.51810400	-2.65644300	-0.00014900
C	-7.66540500	1.50778700	-0.75243700	C	-6.83212700	-3.76163700	0.79563600
C	-7.80699800	-0.37657400	0.73964800	C	-7.51372000	-2.08492100	-0.79730200
C	-9.04872000	1.61084800	-0.74452100	C	-8.11629900	-4.28519300	0.78559900
C	-9.19043600	-0.27575300	0.72658300	C	-8.79802100	-2.60826900	-0.79022500
C	-9.82230600	0.71933600	-0.01026900	C	-9.10765100	-3.71197500	-0.00316800
H	-9.52482700	2.38891800	-1.33131100	H	-8.34564600	-5.14113800	1.41074800
H	-9.77764300	-0.97541800	1.31143100	H	-9.55915900	-2.15619200	-1.41661100
C	-3.95749400	-5.82807200	0.00499600	C	-5.55984700	4.31507900	-0.00012200
C	-5.13772600	-5.88466500	0.75282900	C	-6.67484000	4.03184900	-0.79377700
C	-3.57708800	-6.94818300	-0.74069900	C	-5.56264200	5.46468400	0.79472200
C	-5.91857400	-7.03115300	0.74430700	C	-7.77115600	4.88113700	-0.78380100
C	-4.35609500	-8.09589500	-0.72832100	C	-6.65876700	6.31427700	0.78738100
C	-5.53350400	-8.14622600	0.00897300	C	-7.77029600	6.02792000	0.00254100
H	-6.83023400	-7.05498700	1.33141200	H	-8.62780500	4.64960600	-1.40715600
H	-4.04393400	-8.95378500	-1.31394600	H	-6.64771400	7.20077400	1.41190600
C	3.06866200	-6.34082000	-0.00436300	C	0.95736200	6.97061900	-0.00112400
C	4.22833000	-6.57132000	0.74225800	C	1.94984000	7.54863700	-0.79746300
C	2.52815100	-7.39129000	-0.75250500	C	0.15696200	7.79360000	0.79596500
C	4.83308200	-7.81966300	0.73017800	C	2.13844400	8.92255000	-0.78799700
H	4.65121100	-5.76714600	1.33255500	H	2.56504000	6.91420500	-1.42426200
C	3.13092300	-8.64061700	-0.74371000	C	0.34526300	9.16755200	0.78827100
H	1.63446000	-7.22198900	-1.34127500	H	-0.60632200	7.34736700	1.42214500
C	4.28868900	-8.86450900	-0.00761900	C	1.33733800	9.74099500	0.00062400
H	5.73168200	-7.97812600	1.31651900	H	2.91055400	9.35685400	-1.41352200
H	2.69631500	-9.44209700	-1.33119500	H	-0.28145800	9.79289400	1.41454400
C	1.75949900	4.76270300	-0.00474700	C	3.14962000	-3.98314700	-0.00823000
C	1.10899500	5.69858900	-0.81289800	C	2.83348700	-5.12485400	0.75143200
C	0.98204900	3.92654400	0.79987700	C	2.12272400	-3.37972700	-0.75628400
C	-0.27173100	5.79928600	-0.81118900	C	1.55811700	-5.64442700	0.75170500
H	1.69233500	6.34508700	-1.45792000	H	3.60115100	-5.59101000	1.35778200
C	-0.39932800	4.02726200	0.80148600	C	0.84650100	-3.89961800	-0.75597600
H	1.46714700	3.20109000	1.44234000	H	2.34661900	-2.50878400	-1.36088200
C	-1.04911700	4.96749300	-0.00136500	C	0.53384900	-5.04865400	-0.00750900

H	-0.75668300	6.52374000	-1.45486000	H	1.33514800	-6.51424600	1.35832300
H	-0.98314700	3.37975600	1.44513100	H	0.07741100	-3.43329000	-1.36032100
C	3.95750400	5.82803000	-0.00631600	C	5.55984600	-4.31507900	0.00011500
C	3.57698200	6.94834800	0.73901100	C	5.56263600	-5.46468200	-0.79473200
C	5.13801900	5.88429600	-0.75372100	C	6.67484400	-4.03185100	0.79376400
C	4.35613700	8.09596000	0.72664800	C	6.65876300	-6.31427400	-0.78739900
H	2.66858400	6.91331400	1.32855400	H	4.70416800	-5.68143900	-1.41912200
C	5.91901300	7.03068600	-0.74517800	C	7.77116000	-4.88113800	0.78378000
H	5.43838500	5.02512800	-1.34158800	H	6.67139000	-3.14686000	1.41875300
C	5.53381300	8.14597500	-0.01024000	C	7.77029600	-6.02791900	-0.00256400
H	4.04388100	8.95401800	1.31197500	H	6.64770700	-7.20077000	-1.41192700
H	6.83089200	7.05426700	-1.33195200	H	8.62781200	-4.64960700	1.40713100
C	-3.06866400	6.34083700	0.00589100	C	-0.95736000	-6.97062000	0.00113400
C	-4.22891000	6.57187800	-0.73966300	C	-1.94983400	-7.54864200	0.79747500
C	-2.52751400	7.39079100	0.75429700	C	-0.15696000	-7.79359600	-0.79595800
C	-4.83360100	7.82023900	-0.72627300	C	-2.13843400	-8.92255600	0.78800700
C	-3.13023800	8.64014900	0.74680600	C	-0.34525700	-9.16755000	-0.78826700
C	-4.28857900	8.86457500	0.01178100	C	-1.33732700	-9.74099700	-0.00061800
H	-5.73266100	7.97912800	-1.31179200	H	-2.91054000	-9.35686300	1.41353400
H	-2.69513200	9.44122000	1.33447900	H	0.28146500	-9.79288800	-1.41454200
H	4.76036000	-9.84036100	-0.00886100	H	1.48466300	10.81484600	0.00136000
H	-4.76020600	9.84044600	0.01404100	H	-1.48465000	-10.81484800	-0.00135600
H	-1.63336800	7.22105700	1.34225200	H	0.60632200	-7.34736000	-1.42213900
H	-4.65229800	5.76811000	-1.33015000	H	-2.56503400	-6.91421300	1.42427700
H	6.14334600	9.04219700	-0.01170700	H	8.62718300	-6.69173200	-0.00366000
H	10.90317700	-0.79885800	0.01356500	H	10.11130600	4.12124100	0.00439700
H	-7.07134200	2.19765500	-1.33992600	H	-6.06507000	-4.20021500	1.42261500
H	-10.90323000	0.79903700	-0.01221700	H	-10.11130600	-4.12124200	-0.00441700
H	-5.43798700	-5.02567900	1.34101400	H	-6.67138300	3.14685700	-1.41876300
H	-6.14291800	-9.04252900	0.01046200	H	-8.62718300	6.69173400	0.00363000
H	-2.66889600	-6.91291000	-1.33054600	H	-4.70417600	5.68144200	1.41911600
H	-7.32275100	-1.14593500	1.32919300	H	-7.27077500	-1.23454200	-1.42315400
N	-2.45544100	5.07308800	0.00277100	N	-0.76547200	-5.57248200	0.00184300
N	3.16614100	4.66315100	-0.00404700	N	4.44504700	-3.44977000	0.00059200
N	5.62215400	-0.41022100	0.00176700	N	5.21118700	2.12405900	-0.00092600
N	2.45539000	-5.07308300	-0.00254500	N	0.76547100	5.57248200	-0.00183000
N	-3.16619900	-4.66315300	0.00275300	N	-4.44504900	3.44977000	-0.00059100
N	-5.62220700	0.41023700	-0.00239100	N	-5.21118800	-2.12405900	0.00092900

Structure S19. The coordinates of both the optimized structures of B7L$_1$ at S$_0$ and S$_1$ in toluene.

S$_0$-optimized structure				S$_1$-optimized structure			
C	0.99504500	-5.94033000	-0.08383700	C	1.04584800	5.95361200	0.08311900
C	2.02160000	-6.52829400	-0.83972400	C	2.13891300	6.61441300	0.71087200
C	1.31501200	-4.77525700	0.63130400	C	1.33765000	4.69796800	-0.51940800
C	3.30166000	-5.99970400	-0.85531700	C	3.40145100	6.07643300	0.72665300
H	1.81120000	-7.42290400	-1.41640900	H	1.96050400	7.56720800	1.19622100
C	2.60234700	-4.25323100	0.62584500	C	2.60631500	4.16751200	-0.51610200
H	0.54651000	-4.28692600	1.22239700	H	0.53820900	4.16041100	-1.01875200
C	3.63589700	-4.85743600	-0.10888800	C	3.70013100	4.83410100	0.10442000
H	4.06747500	-6.48817600	-1.44819100	H	4.19869600	6.61089400	1.23018100
H	2.81704100	-3.37043500	1.21954100	H	2.78522600	3.22384900	-1.01973200
C	-1.73459300	-5.77457400	0.08736900	C	-1.67104100	5.81445700	-0.06968600
C	-2.82475600	-6.23576500	0.84220200	C	-2.82065300	6.36099000	-0.70594800
C	-1.91085700	-4.57700800	-0.62393200	C	-1.83816600	4.53660600	0.53243400
C	-4.03087600	-5.55517500	0.86089500	C	-4.02197000	5.69718500	-0.73140900
H	-2.72461900	-7.15120100	1.41586800	H	-2.73621100	7.32723300	-1.19015900
C	-3.12494400	-3.90203200	-0.61535900	C	-3.04660700	3.88004200	0.51989700
H	-1.08901900	-4.18396200	-1.21432600	H	-0.99222600	4.08324800	1.03870500
C	-4.22378600	-4.37794500	0.11893300	C	-4.19713600	4.43151200	-0.11057500
H	-4.85011800	-5.94860200	1.45305300	H	-4.86525100	6.14778200	-1.24195400
H	-3.23074900	-2.99753700	-1.20577200	H	-3.13283700	2.92340400	1.02345600
C	-5.79282200	-2.12891400	0.01798500	C	-5.76893200	2.19059300	-0.04010500
C	-6.99410800	-1.55822500	-0.43827200	C	-6.99792700	1.63388000	0.38283800
C	-4.78111300	-1.24134800	0.41941200	C	-4.76275000	1.27406200	-0.41488600
C	-7.19487300	-0.18691200	-0.43730600	C	-7.22054000	0.27030500	0.38526500
H	-7.79114600	-2.20741300	-0.78408400	H	-7.79215700	2.29857600	0.70357800
C	-4.96669500	0.13336100	0.38435200	C	-4.96982000	-0.09231700	-0.36866200
H	-3.83832400	-1.63793800	0.78120000	H	-3.80713100	1.64854200	-0.76530200
C	-6.18966600	0.69463700	-0.01312600	C	-6.21591800	-0.63646700	-0.00082700
H	-8.14965900	0.21078300	-0.76676900	H	-8.18893400	-0.10988800	0.69438900
H	-4.15955100	0.78716000	0.69956500	H	-4.16537200	-0.76249200	-0.65451000
C	5.47089300	-2.81911600	0.00224000	C	5.49622300	2.76820000	0.01884100
C	4.57821000	-1.81150000	-0.39808400	C	4.59482300	1.75005200	0.39915600
C	6.73382900	-2.40365300	0.45989700	C	6.77511000	2.34423900	-0.41109500
C	4.93454500	-0.47077800	-0.36209600	C	4.94522000	0.41335800	0.35170800
H	3.59335900	-2.08671200	-0.76057000	H	3.60708800	2.02148800	0.75541700
C	7.10480700	-1.06836300	0.45967600	C	7.14115000	1.01221300	-0.41377900
H	7.44318800	-3.14782100	0.80530300	H	7.49256700	3.08908200	-0.73651900
C	6.21830900	-0.06754300	0.03528600	C	6.24045900	0.00412100	-0.02184900
H	4.21607000	0.27925600	-0.67758300	H	4.21812600	-0.33826600	0.64220600
H	8.10207500	-0.79371100	0.78888000	H	8.14309700	0.73669600	-0.72699400
C	6.27039900	-5.39038000	-0.25609900	C	6.34047800	5.31609000	0.26836700
C	7.40954500	-5.13315500	-1.03578100	C	7.43043400	5.06074300	1.11806200
C	6.19967300	-6.63784900	0.38486600	C	6.35351800	6.53162500	-0.43562400
C	8.41980800	-6.07500000	-1.17922600	C	8.46719300	5.97257500	1.26950300
H	7.49637800	-4.18252700	-1.55148100	H	7.45638400	4.13437500	1.68270100
C	7.22181600	-7.57091300	0.27452000	C	7.39853900	7.43700700	-0.30887600
H	5.33075500	-6.87422900	0.99020900	H	5.53103600	6.76630900	-1.10385000
C	8.33189200	-7.29330900	-0.51531100	C	8.45750300	7.16193200	0.55019100
H	9.27987700	-5.85679400	-1.80312600	H	9.28640000	5.75309400	1.94598300
H	7.14952500	-8.51908100	0.79632700	H	7.38524200	8.36202000	-0.87548100
H	9.12581400	-8.02577300	-0.61497700	H	9.27011900	7.87215700	0.65880700
C	-6.90405700	-4.58284600	0.26820400	C	-6.87238400	4.63686300	-0.29816400
C	-8.00244400	-4.18997900	1.04982200	C	-7.92437600	4.26678100	-1.15347700
C	-6.98821600	-5.82694300	-0.37771700	C	-7.01605200	5.84672900	0.40109300
C	-9.12130100	-5.00030100	1.19034200	C	-9.04916000	5.06546100	-1.31473600
H	-7.97099900	-3.23818000	1.56968100	H	-7.85040700	3.34088200	-1.71459500
C	-8.11778700	-6.62697400	-0.27022600	C	-8.14870400	6.63807400	0.26471400
H	-6.15537700	-6.16630200	-0.98481300	H	-6.22648100	6.16757000	1.07316700
C	-9.18484300	-6.21746100	0.52151600	C	-9.16776900	6.25155500	-0.59968600
H	-9.94761700	-4.68025500	1.81592800	H	-9.83685300	4.76006500	-1.99527200
H	-8.16335200	-7.57464800	-0.79593400	H	-8.23522300	7.56128300	0.82779000
H	-10.06315000	-6.84660400	0.61873200	H	-10.04915300	6.87300400	-0.71582600
C	-5.28932800	3.21562500	-0.38581800	C	-5.33503000	-3.15904900	0.38345300
C	-4.80336100	4.24024400	0.43955600	C	-4.85812000	-4.18850500	-0.44213200
C	-4.72465200	3.09245100	-1.66309600	C	-4.76703700	-3.04055900	1.66011800
C	-3.76462100	5.06128900	0.02723900	C	-3.82420100	-5.01655200	-0.03173500
H	-5.22268200	4.37002900	1.43250200	H	-5.28099400	-4.31628200	-1.43381700
C	-3.76670800	3.98965000	-2.11662100	C	-3.81432600	-3.94390600	2.11173400
H	-5.06115300	2.29668200	-2.32069500	H	-5.09807400	-2.24360400	2.31907200
C	-3.24270600	4.97779300	-1.27149800	C	-3.29883200	-4.93669600	1.26606200
H	-3.36307500	5.79717600	0.71718100	H	-3.42997600	-5.75608300	-0.72204600
H	-3.39078500	3.89915600	-3.13122200	H	-3.43609900	-3.85606900	3.12573000
C	5.64684500	2.55038400	0.39843200	C	5.63702600	-2.60158000	-0.39500700
C	5.29724300	3.62439400	-0.43316000	C	5.27858300	-3.67274200	0.43744200
C	5.07029000	2.50807500	1.67558800	C	5.05518200	-2.55273000	-1.66993100
C	4.37234600	4.57434700	-0.02683800	C	4.33980200	-4.61081500	0.03536100
H	5.73037700	3.69377700	-1.42622200	H	5.71637300	-3.74846200	1.42798300
C	4.23544300	3.52368800	2.12307700	C	4.20543700	-3.55731900	-2.11347000
H	5.30155700	1.67951400	2.33809800	H	5.29507800	-1.72798600	-2.33415200
C	3.84348600	4.56590900	1.27164000	C	3.80452200	-4.59562200	-1.26063900
H	4.06879600	5.35164100	-0.72145800	H	4.03083800	-5.38513900	0.73095600
H	3.85096800	3.48819900	3.13790200	H	3.81695300	-3.51694700	-3.12660200
B	2.81202400	5.66704700	1.70169300	B	2.76036800	-5.68460300	-1.68994400
C	-2.18529300	6.78928900	-3.00717200	C	-2.26080400	-6.75657300	3.00325000
C	-1.04796600	7.33671000	-3.62448200	C	-1.12967100	-7.31386700	3.62321300
C	-3.43031600	7.03712900	-3.60946500	C	-3.50921000	-6.99326100	3.60314400
C	-1.14642100	8.09012000	-4.78544700	C	-1.23719100	-8.06589400	4.78426700
H	-0.07050900	7.15334100	-3.19158700	H	-0.14969200	-7.13948900	3.19230600
C	-3.53867800	7.80337500	-4.76095500	C	-3.62668100	-7.75789300	4.75479900
H	-4.32677700	6.62654800	-3.15716100	H	-4.40118400	-6.57530400	3.14872600
C	-2.39418500	8.32828300	-5.35201500	C	-2.48814100	-8.29271300	5.34847600
H	-0.25321600	8.49316200	-5.25019400	H	-0.34851900	-8.47669200	5.25092000
H	-4.51193800	7.98922700	-5.20196300	H	-4.60248300	-7.93488600	5.19383300
H	-2.47433900	8.92154600	-6.25670700	H	-2.57542700	-8.88484800	6.25324200

C	-0.80139600	5.94932200	-0.80673200	C	-0.86443000	-5.93091500	0.80608300
C	-0.10868400	7.11156100	-0.43576700	C	-0.18206600	-7.10006700	0.43720000
C	1.00648600	7.05170300	0.39040700	C	0.93707600	-7.05127100	-0.38412100
C	1.53754800	5.82135000	0.80545400	C	1.48321400	-5.82619000	-0.79558100
C	0.87155500	4.65807500	0.39179300	C	0.82772700	-4.65631700	-0.38345600
C	-0.29871000	4.72351500	-0.34517200	C	-0.34650100	-4.71004000	0.34806800
H	-0.46600800	8.07815600	-0.77736100	H	-0.55095900	-8.06310100	0.77650300
H	1.49221400	7.97347000	0.69563200	H	1.41408900	-7.97783700	-0.68851600
H	1.26598500	3.68868200	0.67910000	H	1.23372300	-3.69097600	-0.66836600
H	-0.82042900	3.80599200	-0.59758000	H	-0.85969500	-3.78735500	0.59925800
C	8.00732200	1.83016500	-0.71723400	C	8.02175800	-1.91676200	0.68771700
C	8.58430100	3.08779000	-0.47193300	C	8.57864100	-3.17948700	0.41943000
C	8.71243600	0.94971600	-1.55512600	C	8.75080300	-1.05739300	1.52806900
C	9.80782400	3.44418200	-1.02079600	C	9.80508400	-3.55869100	0.94556300
H	8.06551300	3.79091600	0.17071500	H	8.04139800	-3.86775900	-0.22408900
C	9.92628400	1.30675000	-2.12473700	C	9.96862400	-1.43700000	2.07343100
H	8.29160400	-0.02590000	-1.77268300	H	8.34571900	-0.08013200	1.76722500
C	10.47833700	2.55394600	-1.85273600	C	10.50046600	-2.68789600	1.77782700
H	10.23829000	4.41613500	-0.80566200	H	10.21894300	-4.53361200	0.71194500
H	10.44480300	0.61415800	-2.77876100	H	10.50605200	-0.75930200	2.72783400
H	11.43096100	2.83276800	-2.29048300	H	11.45585200	-2.98453700	2.19738500
C	3.03887400	6.53214700	2.97524000	C	2.97722600	-6.55005300	-2.96517500
C	4.30821600	6.62176900	3.57109300	C	4.24568400	-6.65408800	-3.56070100
C	1.98934700	7.24579700	3.57811100	C	1.91940300	-7.24970800	-3.57006100
C	4.52435300	7.39448900	4.70285000	C	4.45288400	-7.42702700	-4.69396600
H	5.13823300	6.08087600	3.12916900	H	5.08215100	-6.12448000	-3.11726300
C	2.19415200	8.00819900	4.71911300	C	2.11544000	-8.01219100	-4.71254100
H	0.99456900	7.18730900	3.14980400	H	0.92516200	-7.18003400	-3.14218400
C	3.46469200	8.08663700	5.27977000	C	3.38515500	-8.10499100	-5.27286900
H	5.51532300	7.45628700	5.13917000	H	5.44326200	-7.50004100	-5.12991000
H	1.36640100	8.54193700	5.17317100	H	1.28143200	-8.53488200	-5.16801100
H	3.62885300	8.68606900	6.16893000	H	3.54239300	-8.70455200	-6.16318600
C	-7.72319000	2.80835900	0.73126500	C	-7.77697400	-2.74218300	-0.70892400
C	-8.54027300	2.02713200	1.56587600	C	-8.59685100	-1.96896100	-1.54925700
C	-8.12979900	4.13092400	0.48581100	C	-8.18984000	-4.05925000	-0.44167400
C	-9.69896500	2.53946600	2.13182600	C	-9.76475000	-2.48150700	-2.09509900
H	-8.25153700	1.00463700	1.78364300	H	-8.30304200	-0.95257000	-1.78796300
C	-9.29763300	4.64416100	1.03165200	C	-9.36594200	-4.57263100	-0.96887600
H	-7.52130000	4.76063700	-0.15426500	H	-7.57943200	-4.68380200	0.20158600
C	-10.08181800	3.84860700	1.86013300	C	-10.15384000	-3.78416700	-1.80075800
H	-10.30623900	1.92001400	2.78282100	H	-10.37431700	-1.86735100	-2.74899600
H	-9.59605600	5.66447600	0.81676500	H	-9.66832800	-5.58793300	-0.73637500
H	-10.99108700	4.24944300	2.29535000	H	-11.06999800	-4.18515900	-2.22105100
C	-0.51099400	-8.17751300	-0.00191700	C	-0.43371300	8.19945900	0.01701200
C	0.49713200	-8.97288700	0.56779600	C	0.49902400	8.98953000	-0.67653600
C	-1.60840200	-8.84316600	-0.57301400	C	-1.44626900	8.87938200	0.71551600
C	0.40818100	-10.35804200	0.58292200	C	0.42179600	10.37628900	-0.68130200
H	1.35891800	-8.49327300	1.01945700	H	1.29381000	8.50481200	-1.23429000
C	-1.68930300	-10.22878300	-0.58981900	C	-1.51988600	10.26627400	0.72951100
H	-2.40519400	-8.26136700	-1.02394200	H	-2.18402000	8.30768100	1.26925700
C	-0.68318300	-10.98888100	-0.00384900	C	-0.58697700	11.02037700	0.02650100
H	1.19238000	-10.94730900	1.04575800	H	1.15253400	10.95638700	-1.23501100
H	-2.53939000	-10.71743600	-1.05349500	H	-2.30892100	10.76014000	1.28680500
H	-0.74956300	-12.07163100	-0.00448700	H	-0.64576300	12.10342400	0.02999700
B	6.63679200	1.43809400	-0.08317900	B	6.64589200	-1.50188200	0.07980400
B	-2.07878700	5.93312700	-1.71144300	B	-2.14412500	-5.90214600	1.70710800
B	-6.41373000	2.24153700	0.10074300	B	-6.45634600	-2.17777200	-0.09930200
B	-5.62895700	-3.68128300	0.12317700	B	-5.59151600	3.73872900	-0.13567700
B	-0.41584000	-6.61461000	0.00041200	B	-0.35054400	6.62721800	0.01073200
B	5.11610300	-4.33885200	-0.10851500	B	5.15841900	4.28920100	0.11679700

Structure S20. The coordinates of both the optimized structures of B6N1L$_1$ at S$_0$ and S$_1$ in toluene.

S_0-optimized structure				S_1-optimized structure			
C	0.84484300	5.90626400	0.10571900	C	2.77019200	-5.11190300	0.42932000
C	1.84022300	6.52827000	0.86869700	C	2.19544600	-6.02361300	1.33555200
C	1.16692000	4.73166700	-0.58623000	C	1.95201100	-4.46019600	-0.51217900
C	3.12236900	6.01548200	0.89389900	C	0.85419700	-6.31532100	1.24675900
H	1.60152700	7.42334000	1.43016300	H	2.81300700	-6.48006800	2.09976700
C	2.45288000	4.21931900	-0.52707300	C	0.61214300	-4.78632700	-0.58844400
H	0.41322200	4.24155100	-1.19136600	H	2.39303400	-3.76576600	-1.21749300
C	3.48269200	4.84865300	0.19563900	C	0.01906200	-5.74543300	0.26032600
H	3.87021800	6.52829700	1.48836200	H	0.42905900	-7.02774100	1.94444000
H	2.67533700	3.32384200	-1.09786500	H	0.00738800	-4.32240000	-1.35941500
C	-1.60818400	5.74988800	-0.09111400	C	4.71763400	-3.66579200	0.16359200
C	-2.67023500	6.24278500	-0.85825900	C	5.86566700	-3.58559100	-0.63720900
C	-1.78180500	4.54202300	0.59644000	C	4.16885700	-2.50050400	0.71612300
C	-3.87612900	5.57030400	-0.89343600	C	6.46264000	-2.36037700	-0.85009100
H	-2.54421400	7.16325500	-1.41530200	H	6.26407000	-4.48142000	-1.09739200
C	-2.99147300	3.86988700	0.52791000	C	4.79241900	-1.28546000	0.49444100
H	-0.97527000	4.15055600	1.20531500	H	3.29372900	-2.56665200	1.35131100
C	-4.08841400	4.36444300	-0.20083300	C	5.95923100	-1.17370000	-0.28477500
H	-4.67959000	5.98523900	-1.49166800	H	7.34392800	-2.31558200	-1.47889100
H	-3.10130600	2.95170500	1.09536900	H	4.38075100	-0.40141400	0.96721900
C	-5.66523600	2.12312700	-0.06638700	C	5.95803500	1.54983200	-0.55779100
C	-6.87988600	1.59488800	0.40498100	C	6.63479400	2.75115600	-0.27838400
C	-4.66840000	1.19991100	-0.42061100	C	4.59073200	1.65049600	-0.86939000
C	-7.10527400	0.22885400	0.46662200	C	5.99321600	3.97849200	-0.33513700
H	-7.66938500	2.27380000	0.70895800	H	7.68609000	2.71771100	-0.01434400
C	-4.87984300	-0.16865300	-0.32593500	C	3.93872400	2.87354600	-0.88541700
H	-3.71775600	1.56189600	-0.79799500	H	4.02987800	0.75405700	-1.11232800
C	-6.11479500	-0.69014500	0.08864900	C	4.62721900	4.07093100	-0.63741800
H	-8.06930500	-0.13578000	0.80724700	H	6.55772300	4.88335200	-0.13316000
H	-4.08429000	-0.85038700	-0.60995100	H	2.87783800	2.90763000	-1.11195800
C	5.33353700	2.82917700	0.04155700	C	-2.66233800	-5.35728300	-0.11201700
C	4.46817500	1.78491800	0.40494800	C	-2.61368400	-3.94440200	0.06588800
C	6.60165300	2.46236000	-0.44213400	C	-3.93444000	-5.86301300	-0.50682100
C	4.85476000	0.45516500	0.30894500	C	-3.72046300	-3.14124200	-0.08353300
H	3.48225800	2.02122900	0.79141500	H	-1.67227700	-3.48344400	0.35129000
C	7.00192400	1.13710800	-0.50547700	C	-5.04160600	-5.06173100	-0.63630900
H	7.29337800	3.23758400	-0.75387100	H	-4.02843800	-6.91979000	-0.73241100
C	6.14322600	0.09787500	-0.11664300	C	-5.00619000	-3.66095100	-0.39657100
H	4.15743100	-0.32371900	0.60125200	H	-3.61353500	-2.07160400	0.06821400
H	8.00204200	0.90043500	-0.85492900	H	-5.97340900	-5.51341200	-0.96006600
C	6.10788500	5.37334800	0.48878200	C	-1.61309600	-7.84872600	0.15682900
C	7.19918000	5.06500800	1.31662600	C	-2.66532000	-8.47446300	0.84894600
C	6.09174700	6.64718900	-0.10142900	C	-0.67172600	-8.70022300	-0.44941000
C	8.21187000	5.98394200	1.55816000	C	-2.77581700	-9.85702800	0.92521100
H	7.24704000	4.08956500	1.78954800	H	-3.40470600	-7.85934200	1.35100200
C	7.11742900	7.55977500	0.10827100	C	-0.78400000	-10.08265900	-0.39470900
H	5.26436800	6.92100000	-0.74794100	H	0.16238200	-8.26393100	-0.99141400
C	8.17688400	7.23208200	0.94656500	C	-1.83842500	-10.66967100	0.29728900
H	9.03358600	5.72469700	2.21717000	H	-3.59715200	-10.30248100	1.47727700
H	7.08747300	8.53003000	-0.37588800	H	-0.04744600	-10.70640500	-0.89136200
H	8.97335200	7.94722700	1.12253400	H	-1.92690000	-11.74962400	0.34884100
C	-6.75667100	4.54809800	-0.51559600	C	8.26494800	0.14358000	-0.73519600
C	-7.79024500	4.10603100	-1.35672300	C	8.89678500	0.96972500	-1.67927100
C	-6.91070600	5.81048900	0.07927200	C	9.08226300	-0.72586700	0.00593600
C	-8.90980400	4.88859300	-1.60676900	C	10.26911300	0.92103900	-1.88376000
H	-7.70701600	3.13485900	-1.83353600	H	8.29726400	1.65105500	-2.27362700
C	-8.04282900	6.58477200	-0.13886800	C	10.45898700	-0.75559600	-0.16889700
H	-6.13230900	6.18531200	0.73589700	H	8.63028800	-1.37918900	0.74547900
C	-9.04212700	6.12786700	-0.99048600	C	11.05457400	0.06373900	-1.12156000
H	-9.68404700	4.52926000	-2.27621400	H	10.72831900	1.55726700	-2.63246800
H	-8.14318300	7.54845000	0.34895200	H	11.06867100	-1.42153400	0.43213900
H	-9.92182900	6.73565100	-1.17345400	H	12.12853300	0.03369500	-1.27013000
C	-5.24727800	-3.21564000	0.51185100	C	2.49499300	5.57712700	0.00200100
C	-4.80271500	-4.26452600	-0.30636000	C	1.37672000	6.14908200	-0.62314500
C	-4.64015500	-3.07451300	1.76746100	C	2.33001500	5.10197300	1.31160200
C	-3.76045200	-5.08977600	0.08812500	C	0.14379200	6.18991900	0.01107900
H	-5.25685600	-4.40979900	-1.28170900	H	1.46790800	6.53053900	-1.63535000
C	-3.67849500	-3.97457300	2.20795900	C	1.12392000	5.24284800	1.98284800
H	-4.94487600	-2.26116300	2.41902800	H	3.16924600	4.63553000	1.81843500
C	-3.19361100	-4.98516300	1.36621000	C	-0.00779800	5.76237800	1.33823400
H	-3.39062200	-5.84417300	-0.59960600	H	-0.71768100	6.57498800	-0.52547400
H	-3.26829400	-3.86816900	3.20767500	H	1.04484300	4.90903200	3.01285900
C	5.61429700	-2.52322100	-0.52353200	C	-6.12957700	-1.20705300	-0.63338800
C	5.32086200	-3.61972400	0.30042600	C	-5.71277100	-0.42546700	0.45468800
C	4.98434400	-2.46917700	-1.77461500	C	-6.45664900	-0.51916400	-1.81267700
C	4.39678600	-4.57932000	-0.08440700	C	-5.62787800	0.95602300	0.36861500
H	5.79748700	-3.69940000	1.27258600	H	-5.45200900	-0.91239300	1.38985800
C	4.14975700	-3.49217800	-2.20605800	C	-6.41886200	0.86559300	-1.88497700
H	5.17223600	-1.62443100	-2.43039800	H	-6.74834500	-1.08110100	-2.69524300
C	3.81223700	-4.55619300	-1.35848900	C	-6.00093600	1.64781400	-0.79596100
H	4.13707300	-5.37459600	0.60771500	H	-5.28644600	1.51862800	1.23211600
H	3.72232400	-3.44535800	-3.20314200	H	-6.69870600	1.35408900	-2.81353500
B	2.77577300	-5.66288800	-1.76234300	B	-5.94025400	3.20249300	-0.85484200
C	-2.09173700	-6.78696100	3.08574500	C	-1.55444500	6.41085400	3.47391200
C	-0.93966100	-7.33712000	3.67246800	C	-2.61102000	6.04565000	4.32492700
C	-3.31988200	-7.02418000	3.72576100	C	-0.62039400	7.33778700	3.96634300
C	-1.00806400	-8.08369200	4.83995800	C	-2.72839200	6.57456000	5.60264000
H	0.02561700	-7.16128000	3.21012100	H	-3.34230700	5.32213000	3.98137100
C	-3.39849000	-7.78320300	4.88440600	C	-0.74289000	7.88731900	5.23460700
H	-4.22677400	-6.61054700	3.29763600	H	0.20942700	7.63804500	3.33525000
C	-2.23994400	-8.31149200	5.44432300	C	-1.79682300	7.50212800	6.05638100
H	-0.10386100	-8.48932800	5.28053600	H	-3.54628200	6.26704600	6.24511800
H	-4.35925400	-7.96084000	5.35520400	H	-0.01605200	8.61122900	5.58681800
H	-2.29666100	-8.89928700	6.35435000	H	-1.89083400	7.92293700	7.05185800

C	-0.76540400	-5.95538200	0.84575900	C	-2.62315600	5.19302400	1.27648500
C	-0.08026300	-7.11629500	0.45714700	C	-3.92640200	5.71251200	1.33293200
C	1.01200500	-7.05450400	-0.39910400	C	-4.96863500	5.12006300	0.63364600
C	1.52846900	-5.82327600	-0.82960500	C	-4.77941700	3.93425300	-0.09306200
C	0.87175100	-4.66123800	-0.39800500	C	-3.48249300	3.40091500	-0.12574800
C	-0.27791500	-4.72879800	0.37028000	C	-2.42549800	4.03750800	0.50423100
H	-0.42600900	-8.08352500	0.80876100	H	-4.11866800	6.60786300	1.91586600
H	1.49134400	-7.97546000	-0.71664900	H	-5.95642400	5.56871900	0.67059300
H	1.25626100	-3.69114000	-0.69615200	H	-3.30186500	2.48085400	-0.67181500
H	-0.79484500	-3.81229900	0.63588600	H	-1.42836300	3.61707700	0.42412700
C	8.00226300	-1.77994600	0.51094400	C	-7.71287100	-3.38717900	-0.45745200
C	8.59809800	-3.01313300	0.19653200	C	-8.80287500	-2.80254600	-1.12500000
C	8.71766900	-0.91594600	1.35710100	C	-8.00037800	-4.50907900	0.34072500
C	9.84908800	-3.36065200	0.68606800	C	-10.08459000	-3.32881400	-1.04462100
H	8.07135400	-3.70400900	-0.45295400	H	-8.64481900	-1.91086100	-1.72133500
C	9.95969600	-1.26521300	1.86798800	C	-9.28355600	-5.03004100	0.44579900
H	8.28292200	0.03981800	1.62865400	H	-7.20045900	-4.97015400	0.90953300
C	10.52980000	-2.48711200	1.52734900	C	-10.33180900	-4.44861700	-0.25782500
H	10.29297500	-4.31317200	0.41774300	H	-10.89702200	-2.85740300	-1.58820400
H	10.48595000	-0.58598600	2.52984100	H	-9.46618100	-5.89128800	1.08031700
H	11.50406400	-2.75950900	1.91911800	H	-11.33459000	-4.85605300	-0.18407200
C	2.96999800	-6.52271800	-3.04455700	C	-6.99780400	4.02892500	-1.65660200
C	4.21926200	-6.59309700	-3.68384600	C	-8.29960600	3.54348500	-1.86306900
C	1.91043600	-7.24983500	-3.61276300	C	-6.68946700	5.29040700	-2.19180700
C	4.40718200	-7.36026500	-4.82435800	C	-9.24767400	4.28100400	-2.55912800
H	5.05607400	-6.04094400	-3.26947900	H	-8.57317800	2.57582400	-1.45638100
C	2.08677800	-8.00754700	-4.76159300	C	-7.62445800	6.02363200	-2.90944500
H	0.93012700	-7.20577200	-3.15072100	H	-5.69276400	5.69548300	-2.05132000
C	3.33826700	-8.06672200	-5.36580800	C	-8.90861600	5.52095900	-3.08934900
H	5.38311400	-7.40666000	-5.29509400	H	-10.25019900	3.88881100	-2.69223600
H	1.25160800	-8.55236800	-5.18806400	H	-7.35590900	6.98916300	-3.32458400
H	3.48014300	-8.66211200	-6.26150000	H	-9.64490400	6.09575600	-3.64117900
C	-7.70648300	-2.80025100	-0.53940500	C	4.48032500	6.63332000	-1.52754200
C	-8.52625200	-2.04319200	-1.39329100	C	5.45850800	6.42708100	-2.51513000
C	-8.13448000	-4.10017700	-0.22071300	C	4.07099900	7.95667500	-1.29081500
C	-9.70833600	-2.55711600	-1.90728900	C	5.99342700	7.48394200	-3.23749800
H	-8.22117200	-1.03942100	-1.66837600	H	5.79317100	5.41711300	-2.72633600
C	-9.32582800	-4.61337000	-0.71331400	C	4.61564300	9.02077100	-1.99529100
H	-7.52341300	-4.71179800	0.43437100	H	3.32115700	8.15067600	-0.53153000
C	-10.11271200	-3.84216700	-1.56198200	C	5.57494000	8.78395100	-2.97403700
H	-10.31723700	-1.95710300	-2.57479200	H	6.73782500	7.29803300	-4.00402500
H	-9.64028000	-5.61519300	-0.44162100	H	4.29177100	10.03443600	-1.78614000
H	-11.04037900	-4.24345800	-1.95602500	H	5.99676700	9.61311100	-3.53200400
C	-0.51620900	7.91284000	0.01732500	C	4.99860400	-6.01010300	0.67839700
C	0.24483200	8.66893500	-0.87380500	C	4.68894800	-7.27138100	0.15065000
C	-1.36887100	8.55638800	0.91382500	C	6.15321400	-5.83488400	1.45355700
C	0.15691500	10.05398000	-0.86033800	C	5.53014900	-8.34050700	0.40101400
H	0.90244300	8.16563700	-1.57304100	H	3.81085000	-7.39272000	-0.47076500
C	-1.46263200	9.94110200	0.91095000	C	6.98095500	-6.91518400	1.69800400
H	-1.95502000	7.96601300	1.60851500	H	6.37104900	-4.86423200	1.88097400
C	-0.69859100	10.69560400	0.02797000	C	6.67486900	-8.16897700	1.17431700
H	0.75222400	10.63308800	-1.55740700	H	5.29729900	-9.31110400	-0.01968600
H	-2.12818700	10.43212600	1.61208700	H	7.86348400	-6.78272000	2.31201200
H	-0.76949700	11.77724700	0.03202400	H	7.32807900	-9.01095500	1.36929600
B	6.59901600	-1.39936000	-0.05682600	B	-6.25827300	-2.77990900	-0.51122000
B	-2.01924100	-5.93839700	1.78302600	B	-1.40902800	5.80976900	2.04263700
B	-6.36950600	-2.23439100	0.03325000	B	3.87310200	5.44476700	-0.72369000
B	-5.48214900	3.66952700	-0.24829000	B	6.71423400	0.18725800	-0.52304400
B	4.95442600	4.33882400	0.22944700	B	-1.46790900	-6.28032900	0.09012000
N	-0.42432100	6.49469400	0.01158600	N	4.14989600	-4.91843400	0.43122000

Structure S21. The coordinates of both the optimized structures of B5N2L$_1$ at S$_0$ and S$_1$ in toluene.

S_0-optimized structure				S_1-optimized structure			
C	2.94490200	5.05099800	0.07813800	C	-0.59149200	5.78016900	0.58798000
C	3.90623100	5.13294100	1.08500100	C	0.31426800	6.56566500	1.34586400
C	2.97571500	3.95916900	-0.78980800	C	-0.05795200	4.71841800	-0.18625100
C	4.89078500	4.16905500	1.20142500	C	1.66021800	6.35059600	1.27529100
H	3.88416500	5.96749800	1.77604100	H	-0.06816400	7.32065900	2.01897800
C	3.96854200	2.99593000	-0.68117500	C	1.29409400	4.50886900	-0.26068600
H	2.24086000	3.88566400	-1.58343100	H	-0.72727200	4.10214200	-0.77106700
C	4.95114200	3.09543500	0.30814700	C	2.19880200	5.33650400	0.44590400
H	5.63113300	4.25278300	1.98751000	H	2.32434900	6.94155900	1.89128500
H	4.00153600	2.18126100	-1.39461900	H	1.67334400	3.73123100	-0.91039100
C	0.64012600	5.87286800	-0.16317800	C	-2.88444100	5.05461200	0.19123800
C	-0.18351100	6.73847500	-0.89740900	C	-3.65678700	5.29062500	-0.94306400
C	0.04296300	4.79247600	0.50261700	C	-3.09974800	3.91476600	0.96204400
C	-1.55123900	6.55611500	-0.91180200	C	-4.63954400	4.38201700	-1.29547600
H	0.25964800	7.55847400	-1.44900800	H	-3.48582100	6.18353800	-1.53470500
C	-1.32974900	4.61643100	0.45720400	C	-4.09650400	3.02239000	0.59366000
H	0.65951000	4.11243000	1.07862000	H	-2.51123600	3.75087200	1.85869500
C	-2.18362300	5.49734800	-0.23244900	C	-4.90258500	3.21921500	-0.54342100
H	-2.15470600	7.24927400	-1.48724900	H	-5.23360100	4.57848700	-2.18112100
H	-1.75802300	3.78826300	1.01214800	H	-4.27511400	2.15877600	1.22408200
C	-4.47641700	3.99841000	-0.05626200	C	-5.99027000	0.72944200	-0.72679300
C	-5.78993800	3.96554800	0.44390200	C	-7.13899600	-0.10949400	-0.80122100
C	-3.90782600	2.76775100	-0.42146200	C	-4.77684000	0.04361600	-0.43965200
C	-6.51030100	2.78434600	0.52436000	C	-7.07626800	-1.47388000	-0.64418200
H	-6.26041900	4.89219500	0.75491100	H	-8.10716600	0.35047800	-0.97072600
C	-4.61518400	1.57878300	-0.30773100	C	-4.71823300	-1.31820800	-0.25927700
H	-2.90021900	2.74560700	-0.82318300	H	-3.85194100	0.60998500	-0.39638700
C	-5.94597700	1.55957600	0.13691400	C	-5.85930900	-2.15635600	-0.37655800
H	-7.53312000	2.80959900	0.88713700	H	-7.99614900	-2.04820200	-0.69808600
H	-4.14033500	0.64775400	-0.60117600	H	-3.75488400	-1.77907200	-0.06282000
C	5.89977600	0.81239300	0.20087800	C	4.19496800	3.98923500	-0.04379800
C	4.67341100	0.14988900	0.35704400	C	3.77860100	2.75712900	0.46296700
C	7.02737700	0.04543900	-0.13573700	C	5.31254000	4.05282700	-0.87977000
C	4.59666900	-1.22259000	0.19672300	C	4.49476900	1.61110800	0.14758200
H	3.78553800	0.71018500	0.62214100	H	2.92480200	2.70325400	1.12832900
C	6.93867600	-1.32912300	-0.23397900	C	6.03355800	2.90445900	-1.15080400
H	7.97841500	0.53544800	-0.30119600	H	5.62243200	5.00573600	-1.29126900
C	5.72530800	-2.01854300	-0.06598300	C	5.65929000	1.65369700	-0.63410300
H	3.63190400	-1.70224600	0.32636100	H	4.16715800	0.66389300	0.56149100
H	7.83860400	-1.88698500	-0.47116500	H	6.91498500	2.97949800	-1.77769300
C	7.30163100	2.72762400	0.73093800	C	4.40486600	6.30220700	0.63779100
C	7.97690600	2.28476700	1.86764200	C	5.42570800	6.15406600	1.57453100
C	7.86994300	3.71854700	-0.06737200	C	4.21988600	7.51756500	-0.01901100
C	9.21398700	2.82363000	2.19366200	C	6.25381500	7.22964600	1.85843200
H	7.52762900	1.51805700	2.48831600	H	5.55929100	5.20337300	2.07691800
C	9.10103600	4.26297800	0.27185700	C	5.05265000	8.58797700	0.27460100
H	7.34025400	4.05746300	-0.95026200	H	3.43589500	7.61362900	-0.76112600
C	9.77880300	3.81585700	1.40015100	C	6.06915400	8.44741000	1.21227900
H	9.73255300	2.47397800	3.07938400	H	7.04293300	7.11676600	2.59254100
H	9.53616200	5.03324600	-0.35499500	H	4.91265400	9.53107900	-0.24050900
H	10.74218500	4.23910900	1.66058700	H	6.71941400	9.28453400	1.43732700
C	-4.60037700	6.64967100	-0.51976700	C	-7.33605600	2.89659600	-1.63925700
C	-5.72464700	6.61562100	-1.35977700	C	-7.98022500	2.30684700	-2.74108600
C	-4.28484400	7.88021900	0.07765500	C	-7.86898800	4.11712600	-1.18774400
C	-6.48232400	7.75259900	-1.60832600	C	-9.08295300	2.89183300	-3.35088000
H	-6.00196600	5.68107300	-1.83657600	H	-7.59607300	1.37121200	-3.13442100
C	-5.05724500	9.01413700	-0.13874000	C	-8.98030000	4.70271200	-1.77957700
H	-3.42350500	7.94441800	0.73462800	H	-7.40255200	4.61359600	-0.34195200
C	-6.15402500	8.95425700	-0.99078200	C	-9.59325200	4.09214100	-2.86882200
H	-7.33469500	7.70076800	-2.27735500	H	-9.54630400	2.40947600	-4.20573800
H	-4.79979900	9.94725100	0.35102700	H	-9.37071400	5.63838000	-1.39158800
H	-6.75172700	9.84111500	-1.17251000	H	-10.45831800	4.54850000	-3.33842900
C	-6.07998900	-1.10817400	0.55788600	C	-4.46989400	-4.30862200	0.42436800
C	-6.07837700	-2.24285000	-0.26662300	C	-3.63073100	-5.20601400	-0.25492000
C	-5.43710900	-1.21174500	1.79910700	C	-4.10739700	-3.97514500	1.73846700
C	-5.41107500	-3.39989400	0.10619900	C	-2.45610200	-5.67159500	0.31399700
H	-6.57569900	-2.20257600	-1.23083500	H	-3.88728600	-5.50746700	-1.26627700
C	-4.87198800	-2.40862200	2.22048000	C	-2.98591400	-4.52257700	2.34688000
H	-5.40164200	-0.34741000	2.45536900	H	-4.72671000	-3.27767200	2.29471300
C	-4.81741300	-3.52152600	1.37029100	C	-2.10726400	-5.35786400	1.63873700
H	-5.36466900	-4.23343500	-0.58811800	H	-1.79588900	-6.30411700	-0.27226500
H	-4.43058400	-2.46892500	3.21076500	H	-2.75869900	-4.26219500	3.37666700
C	4.29434700	-4.25938800	-0.54856000	C	5.91084900	-1.03401300	-0.94648700
C	3.61723900	-5.16463100	0.28107700	C	6.50045400	-2.15145600	-0.33489900
C	3.74130600	-4.00502300	-1.81089000	C	4.72519700	-1.25461500	-1.66391000
C	2.41395400	-5.73342000	-0.10764500	C	5.92067600	-3.40917200	-0.40890200
H	4.02459100	-5.39844500	1.26001900	H	7.42127500	-2.02705000	0.22587000
C	2.59840500	-4.66575300	-2.24444600	C	4.17165300	-2.52169900	-1.78105900
H	4.22939900	-3.29694600	-2.47388100	H	4.23784000	-0.41932400	-2.15768900
C	1.88992300	-5.52296100	-1.39143700	C	4.74356500	-3.62817500	-1.13829800
H	1.87883900	-6.37232100	0.58842500	H	6.39404300	-4.23812800	0.10910900
H	2.22797600	-4.48471800	-3.24907400	H	3.27629500	-2.64861100	-2.38150000
B	0.53077500	-6.18708500	-1.80294000	B	4.10077500	-5.06773200	-1.15735000
C	-4.45291300	-5.64335200	3.03866500	C	-0.66598300	-6.37514700	3.71321200
C	-3.58907800	-6.59829400	3.60083800	C	0.53535300	-6.34320500	4.44063800
C	-5.68377900	-5.42662800	3.68071200	C	-1.79378800	-6.91125400	4.35698700
C	-3.93427600	-7.30110900	4.74625100	C	0.60779100	-6.81380600	5.74438200
H	-2.62542300	-6.77881300	3.13692800	H	1.42396600	-5.92806100	3.97689600
C	-6.04262400	-6.13623100	4.81752300	C	-1.72551700	-7.40094000	5.65390700
H	-6.37061900	-4.69333000	3.27156300	H	-2.73627000	-6.95154300	3.82142400
C	-5.16510300	-7.07339300	5.35284400	C	-0.52344800	-7.34893100	6.35139200
H	-3.24675200	-8.02619500	5.16791100	H	1.54510100	-6.76686800	6.28845700
H	-7.00233500	-5.95866800	5.29048600	H	-2.60874100	-7.82001800	6.12383900
H	-5.43964700	-7.62514300	6.24565600	H	-0.46839400	-7.72441800	7.36777800

C	-2.89378400	-5.28169300	0.82146600	C	0.52334000	-5.71388000	1.34993900
C	-2.66668100	-6.59767200	0.39132500	C	1.55840600	-6.65921800	1.30507800
C	-1.61809900	-6.90295600	-0.46737200	C	2.66217300	-6.48280200	0.48124000
C	-0.69227300	-5.92494600	-0.86049500	C	2.82781400	-5.31447500	-0.28140500
C	-0.89281900	-4.61983000	-0.38681100	C	1.81599900	-4.34726600	-0.19300100
C	-1.99541100	-4.29646000	0.38531700	C	0.67462800	-4.56378900	0.55973900
H	-3.33876700	-7.38802100	0.71132100	H	1.48601900	-7.55866100	1.90866200
H	-1.49841800	-7.92343300	-0.81816300	H	3.42702500	-7.25231600	0.45153100
H	-0.18560600	-3.84209200	-0.65633900	H	1.91344700	-3.41512500	-0.73793100
H	-2.15223600	-3.26461200	0.68274200	H	-0.11014200	-3.81479700	0.56179400
C	6.80706800	-4.46756700	0.40724100	C	8.11184300	0.53270500	-0.85638500
C	6.95360900	-5.77979300	-0.07208500	C	8.90861800	-0.35615500	-1.59739700
C	7.74903100	-4.01885600	1.34754200	C	8.77215000	1.55225000	-0.15069700
C	7.99925800	-6.59300400	0.34201400	C	10.28945500	-0.22468000	-1.64704600
H	6.23624000	-6.16249000	-0.79045300	H	8.43138400	-1.15558300	-2.15393900
C	8.78373800	-4.83432000	1.78568100	C	10.15469600	1.67261800	-0.17200600
H	7.65529200	-3.01819500	1.75546900	H	8.19096800	2.25230800	0.44019300
C	8.91507900	-6.12141800	1.27632400	C	10.91542400	0.78741200	-0.92789900
H	8.09726500	-7.59741900	-0.05563400	H	10.87964900	-0.91447900	-2.24035400
H	9.48931200	-4.46750700	2.52343400	H	10.64093500	2.45820500	0.39618300
H	9.72716500	-6.75822200	1.61088400	H	11.99531900	0.88547900	-0.95534000
C	0.40247300	-7.02624900	-3.10812800	C	4.75590300	-6.20573200	-1.99895900
C	1.54103700	-7.53943200	-3.75190200	C	6.04918000	-6.06959900	-2.53184300
C	-0.84680800	-7.29508400	-3.69212200	C	4.05745600	-7.38771100	-2.30031200
C	1.43924700	-8.29378500	-4.91187900	C	6.63041900	-7.07273800	-3.29282800
H	2.51984100	-7.34769500	-3.32531300	H	6.60704100	-5.15898100	-2.34250500
C	-0.95573500	-8.03488100	-4.86092100	C	4.62485500	-8.38827400	-3.07651800
H	-1.74439000	-6.90284400	-3.22630000	H	3.04537300	-7.51448200	-1.93302000
C	0.18879200	-8.53941900	-5.46933400	C	5.91709900	-8.23531600	-3.56625100
H	2.33184900	-8.68791200	-5.38539300	H	7.63464100	-6.94752100	-3.68288600
H	-1.93040000	-8.22003700	-5.29915700	H	4.06087400	-9.28679000	-3.30158900
H	0.10643300	-9.12277300	-6.38027200	H	6.36516700	-9.01782600	-4.16934500
C	-8.22847700	0.20204000	-0.43690300	C	-6.79798900	-4.66814900	-0.88606200
C	-8.72467400	1.21108200	-1.27938100	C	-7.55043400	-4.35927900	-2.03271800
C	-9.10497800	-0.84243200	-0.09726000	C	-7.01166300	-5.93614600	-0.31741900
C	-10.02510800	1.17792800	-1.76268500	C	-8.46741900	-5.25252700	-2.57114700
H	-8.07211300	2.02705700	-1.57020700	H	-7.39716100	-3.40329600	-2.52186500
C	-10.41321500	-0.87147600	-0.55892400	C	-7.93765200	-6.82961300	-0.83846900
H	-8.75244400	-1.63856400	0.54967600	H	-6.43959200	-6.22072000	0.56013700
C	-10.87344700	0.13828800	-1.39721600	C	-8.67072300	-6.49049600	-1.97085600
H	-10.38016700	1.96231000	-2.42225300	H	-9.02394700	-4.98420500	-3.46346500
H	-11.07374900	-1.68226400	-0.27142600	H	-8.08553800	-7.79498400	-0.36503200
H	-11.89291700	0.11400200	-1.76728100	H	-9.39013500	-7.18840500	-2.38634600
C	2.52749300	7.42189400	-0.16721200	C	-2.44469600	7.29448200	1.00878700
C	3.64015100	7.69324900	-0.96512900	C	-1.88441000	8.47724000	0.52032600
C	1.94904400	8.45654800	0.57152100	C	-3.55578400	7.34342800	1.85182100
C	4.16336400	8.97744900	-1.02018100	C	-2.41951700	9.69936000	0.89832400
H	4.09131000	6.89272200	-1.53957300	H	-1.05020100	8.43299800	-0.17004500
C	2.46865300	9.74077500	0.49772300	C	-4.08087700	8.57091400	2.22255900
H	1.09260100	8.24819200	1.20187000	H	-3.99736800	6.42237300	2.21148100
C	3.57939800	10.00886000	-0.29445200	C	-3.51521900	9.75155500	1.75199300
H	5.02772700	9.17330900	-1.64507100	H	-1.98734500	10.61374600	0.50883800
H	2.00893700	10.53400500	1.07688400	H	-4.93859900	8.60387700	2.88394500
H	3.98670100	11.01212600	-0.34401800	H	-3.93441600	10.70809900	2.04083500
B	5.62319500	-3.56699600	-0.07974000	B	6.55630100	0.37857100	-0.82765200
B	-4.06743300	-4.84387100	1.76050400	B	-0.75812300	-5.84325100	2.24789300
B	-6.76286700	0.22435200	0.10001000	B	-5.73796600	-3.68064200	-0.27589000
B	-3.73249400	5.36532700	-0.25693500	B	-6.07247400	2.24127800	-0.95676800
N	2.01341900	6.10448000	-0.09045400	N	-1.92746800	6.03620900	0.60810000
N	6.02874500	2.18805200	0.39266600	N	3.55767400	5.19542600	0.33724100

Structure S22. The coordinates of both the optimized structures of B4N3L$_1$ at S$_0$ and S$_1$ in toluene.

S$_0$-optimized structure				S$_1$-optimized structure			
C	-3.79694900	4.38160600	-0.04267000	C	-1.37613600	5.59564800	-0.58394500
C	-4.89045400	4.33151300	-0.90823500	C	-2.47704500	5.98884600	-1.37996200
C	-3.51902400	3.26333900	0.74561300	C	-1.54164100	4.47289500	0.25953900
C	-5.70300600	3.21400400	-0.95872000	C	-3.67677500	5.33571400	-1.29635100
H	-5.10900400	5.18338000	-1.54127300	H	-2.36190000	6.79725600	-2.08925700
C	-4.33587500	2.14276100	0.69824400	C	-2.75085100	3.82651500	0.35005900
H	-2.67902300	3.28500000	1.43044900	H	-0.71770200	4.15366300	0.88373000
C	-5.45241800	2.10788200	-0.14203100	C	-3.85668600	4.24933300	-0.41451300
H	-6.54959200	3.19805400	-1.63435100	H	-4.49067500	5.63934700	-1.94169200
H	-4.12341300	1.30041200	1.34603900	H	-2.86395900	3.01497600	1.05787800
C	-1.65479400	5.59568700	0.07957900	C	1.03036600	5.67053200	-0.19595700
C	-0.97338600	6.61957800	0.75355100	C	1.66337100	6.13863600	0.95190800
C	-0.89659400	4.62306800	-0.58936800	C	1.62537300	4.66974500	-0.95985700
C	0.40383200	6.69180300	0.70706900	C	2.88800000	5.60643800	1.32084100
H	-1.53513300	7.36261900	1.30590600	H	1.19729000	6.92207700	1.53999500
C	0.48623200	4.69952100	-0.59938800	C	2.85499800	4.15426500	-0.57529500
H	-1.39971500	3.82457200	-1.12180500	H	1.13711400	4.32371900	-1.86498800
C	1.19155000	5.74317500	0.02820600	C	3.53287300	4.60084600	0.57428500
H	0.89246100	7.50382000	1.23404300	H	3.36978100	5.98542800	2.21550700
H	1.03651700	3.94360300	-1.15018400	H	3.31965000	3.40149300	-1.20206800
C	3.71615700	4.67964200	-0.15356500	C	5.38515900	2.61351800	0.74590200
C	5.00900000	4.85392500	-0.67799300	C	6.74871800	2.20560800	0.81004700
C	3.38586800	3.39241400	0.29929900	C	4.47094500	1.56588300	0.44074400
C	5.93507700	3.82302700	-0.69232700	C	7.14614000	0.90263800	0.62610500
H	5.29807900	5.82739900	-1.05939000	H	7.50809000	2.95956600	0.99160300
C	4.30003800	2.34874200	0.25129800	C	4.87081200	0.26684600	0.23550800
H	2.40180600	3.21310700	0.71961700	H	3.40987000	1.79123800	0.40367700
C	5.60994500	2.54559600	-0.21163800	C	6.22723500	-0.14439100	0.34237300
H	6.93371800	4.01128200	-1.07409200	H	8.20569300	0.66979800	0.67049000
H	4.00704800	1.36823600	0.61355200	H	4.11654800	-0.48617200	0.02743400
C	-5.91560300	-0.31651300	-0.14018900	C	-5.31302500	2.31829600	0.02703800
C	-4.66584000	-0.69047100	-0.64553000	C	-4.40056600	1.30805100	-0.31198400
C	-6.75393300	-1.32104200	0.36209300	C	-6.50136600	1.94937600	0.68011800
C	-4.28753000	-2.02365700	-0.67774600	C	-4.68119600	-0.01551000	-0.04342500
H	-3.99693100	0.06170700	-1.04509200	H	-3.48769900	1.55643400	-0.83898300
C	-6.37497900	-2.65091400	0.31667500	C	-6.78978900	0.63029500	0.93177100
H	-7.71397200	-1.05553400	0.78662800	H	-7.19538300	2.71436600	1.00390400
C	-5.14173200	-3.02344400	-0.21592500	C	-5.90123100	-0.39223800	0.54719400
H	-3.32562300	-2.29691000	-1.09689000	H	-3.97606600	-0.77811400	-0.34788700
H	-7.04271100	-3.41560200	0.69725600	H	-7.70781300	0.37598300	1.44528800
C	-7.73245800	1.30269200	-0.26552000	C	-6.25050800	4.45967100	-0.58274000
C	-8.50204900	0.71322700	-1.26959900	C	-7.16401300	4.05805800	-1.55565800
C	-8.33463800	2.19229000	0.62482500	C	-6.44950200	5.64053700	0.12990100
C	-9.85556900	1.00317400	-1.37062800	C	-8.27653900	4.84512000	-1.81381600
H	-8.03426600	0.02740400	-1.96636100	H	-6.99147600	3.14169700	-2.10743400
C	-9.68500700	2.49015800	0.50733700	C	-7.56594600	6.41986400	-0.13598600
H	-7.73658000	2.64753600	1.40580300	H	-5.73714200	5.93260400	0.89235700
C	-10.45295100	1.89468000	-0.48671800	C	-8.48003800	6.02523400	-1.10651000
H	-10.44281900	0.53845900	-2.15489800	H	-8.98257200	4.53846200	-2.57645900
H	-10.14092600	3.18315800	1.20572100	H	-7.72557800	7.33419600	0.42325800
H	-11.50898900	2.12372900	-0.57235000	H	-9.35097400	6.63644200	-1.31187200
C	3.35724300	7.33487900	0.14687100	C	5.92537400	5.08789200	1.70512600
C	4.49832400	7.56575800	0.93145700	C	6.72626000	4.72370800	2.80167200
C	2.78903300	8.44497100	-0.49798400	C	6.02215200	6.42532100	1.28156600
C	5.03422500	8.83825400	1.08038900	C	7.57020100	5.62958300	3.43206300
H	4.96650900	6.73212500	1.44466100	H	6.67599400	3.70518000	3.17301900
C	3.33815900	9.71532100	-0.38107200	C	6.87377300	7.33517300	1.89427400
H	1.90593900	8.30505600	-1.11291300	H	5.41903700	6.75549600	0.44103400
C	4.45889800	9.91595200	0.41655500	C	7.65256100	6.94110600	2.97739500
H	5.90583400	8.98949500	1.70826600	H	8.16618500	5.31126300	4.28160000
H	2.88764900	10.55120500	-0.90561500	H	6.93078600	8.35530800	1.52754800
H	4.88285400	10.90905900	0.52049300	H	8.31520100	7.64964600	3.46329600
C	6.24271900	-0.07280700	-0.44681900	C	5.62192200	-2.62492500	-0.48539600
C	6.42756900	-1.12561900	0.46112400	C	5.09177100	-3.72274100	0.21032400
C	5.65832800	-0.38203200	-1.68248900	C	5.19753800	-2.45263800	-1.81179500
C	5.98901600	-2.40908100	0.17120600	C	4.13152200	-4.54496700	-0.35673800
H	6.88776800	-0.92553900	1.42392100	H	5.40995000	-3.90405000	1.23288900
C	5.32868500	-1.68934700	-2.01693900	C	4.31638000	-3.33921500	-2.41718900
H	5.48266400	0.41096700	-2.40314500	H	5.57704500	-1.60899000	-2.38085900
C	5.45566900	-2.72802800	-1.08488300	C	3.73013100	-4.39046000	-1.69470200
H	6.07877600	-3.18392800	0.92667500	H	3.68924400	-5.33641300	0.24144800
H	4.92953500	-1.90094800	-3.00444300	H	4.03975600	-3.18779000	-3.45675000
C	-3.54839900	-4.80050700	0.23722000	C	-5.19977500	-2.70978400	0.84528000
C	-2.57527400	-5.41139600	-0.55819700	C	-4.91033700	-3.52922900	-0.24169300
C	-3.28056400	-4.57893700	1.58944500	C	-4.52202300	-2.88147300	2.04854600
C	-1.35459900	-5.76358200	-0.00945900	C	-3.92926700	-4.50279600	-0.12361900
H	-2.77664900	-5.58074200	-1.60986900	H	-5.44518900	-3.38827000	-1.17438700
C	-2.07468600	-4.99031800	2.13760400	C	-3.59213200	-3.90613500	2.17341700
H	-4.03306500	-4.10070300	2.20636500	H	-4.75270000	-2.23438000	2.88765200
C	-1.07290500	-5.58246900	1.35435100	C	-3.26760400	-4.73255000	1.09033500
H	-0.59561200	-6.19207100	-0.65604700	H	-3.68168900	-5.10720400	-0.99008400
H	-1.90079600	-4.83352700	3.19746100	H	-3.10438300	-4.05958800	3.13050800
B	0.32399800	-5.98991800	1.92408200	B	-2.19096700	-5.87677300	1.19641400
C	5.56289100	-5.01778200	-2.55266500	C	2.72993600	-5.91167800	-3.72735400
C	4.90966900	-6.14721000	-3.07405600	C	1.59767700	-6.35305100	-4.43212300
C	6.78840700	-4.65281200	-3.13496500	C	3.97610700	-6.04514400	-4.36224600
C	5.45120100	-6.87671900	-4.12267200	C	1.70010600	-6.89118500	-5.70720600
H	3.95406400	-6.44615500	-2.65718400	H	0.61923000	-6.25966100	-3.97268800
C	7.34400700	-5.38620400	-4.17325100	C	4.08907000	-6.59681200	-5.63072900
H	7.31350700	-3.78205000	-2.75686900	H	4.86977900	-5.71775800	-3.84191300
C	6.67299100	-6.49895500	-4.66962900	C	2.94878800	-7.01732900	-6.30693000
H	4.92392800	-7.73932300	-4.51527500	H	0.80928900	-7.21505300	-6.23478900
H	8.29619000	-5.09088500	-4.60029000	H	5.06420900	-6.69758700	-6.09487000
H	7.10130600	-7.07049200	-5.48618600	H	3.03327000	-7.44347100	-7.30107800

C	3.79842300	-4.72876600	-0.48349900	C	1.34319400	-5.53598100	-1.40845300
C	3.73196900	-6.03210600	0.03212100	C	0.72520600	-6.78050300	-1.21534200
C	2.66990100	-6.43672300	0.83082300	C	-0.37380300	-6.91690100	-0.37750300
C	1.57951700	-5.58849500	1.07575700	C	-0.96484600	-5.80059600	0.23595700
C	1.63124200	-4.29846400	0.52710000	C	-0.38183200	-4.54793800	-0.01004500
C	2.73525000	-3.86306900	-0.18608600	C	0.77139300	-4.42718400	-0.76672900
H	4.53720300	-6.73027500	-0.17486800	H	1.13227700	-7.65965200	-1.70540700
H	2.67061100	-7.43875100	1.24877700	H	-0.79783100	-7.90275800	-0.21333600
H	0.80025600	-3.61932200	0.68785000	H	-0.82023000	-3.66010200	0.43464200
H	2.76518900	-2.84043800	-0.54811200	H	1.23445800	-3.45266500	-0.88117700
C	-5.52910300	-5.26006000	-1.12163500	C	-7.56605400	-2.17563300	0.73791600
C	-5.49411200	-6.64214800	-0.89458000	C	-7.95864700	-3.18547300	1.61780700
C	-6.34008700	-4.77237600	-2.15297500	C	-8.49513400	-1.65935600	-0.16954900
C	-6.23906300	-7.50344100	-1.68501800	C	-9.26243900	-3.65923200	1.59591700
H	-4.88399400	-7.03699100	-0.09122800	H	-7.24002700	-3.59737100	2.31577500
C	-7.09218200	-5.64474600	-2.92688100	C	-9.79935900	-2.13078600	-0.17409200
H	-6.37648600	-3.70802700	-2.34910900	H	-8.18949900	-0.89718200	-0.87635700
C	-7.04752600	-7.01550700	-2.70559800	C	-10.19116600	-3.13237800	0.70673600
H	-6.19510300	-8.56933400	-1.48841400	H	-9.55344000	-4.44299300	2.28600100
H	-7.71182000	-5.24233400	-3.72118000	H	-10.50884800	-1.72324600	-0.88553700
H	-7.63348600	-7.69236500	-3.31641300	H	-11.20926300	-3.50342400	0.69497900
C	0.46720000	-6.73274500	3.28697800	C	-2.35667500	-7.00930000	2.25161400
C	-0.60129900	-7.46147700	3.83565600	C	-3.60886900	-7.28796200	2.82517800
C	1.66938100	-6.70466400	4.01336300	C	-1.26388400	-7.78301700	2.67793000
C	-0.47565800	-8.13588600	5.04191200	C	-3.76854100	-8.29900700	3.76148100
H	-1.54139600	-7.50770500	3.29629600	H	-4.47207500	-6.70590600	2.51970000
C	1.79718800	-7.36089200	5.22943800	C	-1.41215300	-8.78298900	3.62847400
H	2.51218200	-6.14631000	3.62006200	H	-0.28042500	-7.58272600	2.26721600
C	0.72446500	-8.08201900	5.74282200	C	-2.66724000	-9.04601300	4.16666000
H	-1.31166600	-8.70143500	5.43894800	H	-4.74758400	-8.50398200	4.18076800
H	2.73213600	-7.31439500	5.77721500	H	-0.55130400	-9.35855700	3.95048500
H	0.82385500	-8.60226600	6.68957100	H	-2.78679400	-9.83160700	4.90515300
C	8.10013400	1.68113200	0.44570900	C	7.95540700	-2.20985100	0.79547200
C	8.39405200	2.82674500	1.20425500	C	8.58516500	-1.68488200	1.93774000
C	9.15646200	0.79137700	0.18671700	C	8.56400300	-3.33093200	0.20379300
C	9.67318900	3.07031600	1.68405400	C	9.75512800	-2.23170900	2.44815100
H	7.59998800	3.52942700	1.43207800	H	8.13431300	-0.83894700	2.44544100
C	10.44263000	1.03838500	0.64509500	C	9.74148500	-3.87581600	0.69746300
H	8.96230800	-0.10389500	-0.39414600	H	8.10183500	-3.77807600	-0.67083600
C	10.70119000	2.17803300	1.39911900	C	10.34374300	-3.32675700	1.82471000
H	9.87179400	3.95571900	2.27816700	H	10.20842000	-1.80496500	3.33722100
H	11.24345800	0.34225800	0.42060900	H	10.18952400	-4.73374200	0.20626700
H	11.70375300	2.36971300	1.76656700	H	11.26045900	-3.75336500	2.21832800
C	-3.77506500	6.79147500	0.17319600	C	-0.11245200	7.61578500	-1.09203500
C	-4.82344600	6.90373900	1.08697400	C	-1.02889200	8.57337500	-0.65093100
C	-3.46389600	7.87642600	-0.64829500	C	0.92267200	7.99233400	-1.94852200
C	-5.54946200	8.08338900	1.17357800	C	-0.92284700	9.88571600	-1.08722500
H	-5.06509700	6.06211800	1.72555000	H	-1.80971700	8.29024900	0.04516900
C	-4.18410500	9.05788000	-0.54420400	C	1.02268500	9.30833200	-2.37353000
H	-2.65602300	7.78742900	-1.36526100	H	1.64396800	7.25295800	-2.27416600
C	-5.23186700	9.16709300	0.36319600	C	0.10031800	10.25909500	-1.95074100
H	-6.36158800	8.15776100	1.88831200	H	-1.63480600	10.62308800	-0.73416500
H	-3.93225600	9.89330200	-1.18805400	H	1.82794400	9.59035000	-3.04191500
H	-5.79711800	10.08907200	0.43670300	H	0.18461900	11.28674100	-2.28434400
N	-6.34728600	1.01783200	-0.15237500	N	-5.10357300	3.65758600	-0.31040200
N	-4.78047900	-4.39102200	-0.31591000	N	-6.22556000	-1.72058100	0.74010200
N	-3.04951700	5.57723600	0.06751900	N	-0.19198500	6.27659300	-0.63238900
B	4.95794900	-4.18699600	-1.38411300	B	2.61948200	-5.30196000	-2.29547000
B	6.66153100	1.39202700	-0.08651900	B	6.61963200	-1.61836300	0.21430100
B	2.73850400	5.89779200	-0.00750400	B	4.95599800	4.06174200	0.99783300

Structure S23. The coordinates of both the optimized structures of B3N4L$_1$ at S_0 and S_1 in toluene.

S_0-optimized structure				S_1-optimized structure			
C	0.53976200	5.78127900	0.07852800	C	0.94308100	5.70672000	0.52315300
C	1.46015300	6.33442500	0.96949800	C	2.00780600	6.18611100	1.32086400
C	0.94530500	4.70908800	-0.71806500	C	1.19201800	4.58646100	-0.30294300
C	2.75646600	5.85851700	1.03499500	C	3.24817200	5.61097200	1.26137900
H	1.15556300	7.15327900	1.61060800	H	1.83205800	6.99690500	2.01487000
C	2.24680000	4.23214200	-0.65582500	C	2.44187400	4.02004200	-0.37130400
H	0.24717100	4.26917800	-1.42106700	H	0.39765700	4.20529700	-0.93083900
C	3.18015700	4.81457100	0.20695700	C	3.50860200	4.52358000	0.40060400
H	3.45595400	6.30639500	1.73022400	H	4.03202900	5.97695900	1.91147600
H	2.55079200	3.42411200	-1.31080600	H	2.61616400	3.20794700	-1.06576600
C	-1.91236200	5.59912300	-0.07125500	C	-1.46316900	5.59621300	0.14768000
C	-3.04428700	6.06771400	-0.75416000	C	-2.16061100	6.00626200	-0.98515500
C	-2.00097900	4.36615500	0.59225100	C	-1.95503600	4.55240700	0.92772700
C	-4.22348500	5.35111300	-0.72773400	C	-3.34634800	5.37429600	-1.32230300
H	-2.99133000	7.00144400	-1.30014000	H	-1.77513200	6.82279700	-1.58642000
C	-3.18631500	3.65078600	0.58239300	C	-3.14681400	3.93611000	0.57423300
H	-1.14253100	3.98685800	1.13387400	H	-1.41905800	4.25203800	1.82211900
C	-4.34771600	4.11805200	-0.06061900	C	-3.88831100	4.32229600	-0.55789200
H	-5.07778800	5.74987900	-1.26360600	H	-3.88012800	5.70965200	-2.20483000
H	-3.22278600	2.71331000	1.12762000	H	-3.53267800	3.14999100	1.21343000
C	-5.80404200	1.80146400	0.06869800	C	-5.58119900	2.19434300	-0.66768500
C	-6.96375800	1.18901800	0.57511200	C	-6.91082400	1.68176500	-0.67913100
C	-4.78079200	0.94879500	-0.37515000	C	-4.57803600	1.22164000	-0.39390900
C	-7.11454100	-0.18878500	0.58720700	C	-7.19988600	0.35406600	-0.47333100
H	-7.77091500	1.81114200	0.94696400	H	-7.73176800	2.37429200	-0.83533600
C	-4.91484400	-0.43174500	-0.32906800	C	-4.86858500	-0.10310600	-0.17170600
H	-3.87187000	1.37738900	-0.78406300	H	-3.53691500	1.52873400	-0.39612400
C	-6.09761900	-1.03642000	0.12255300	C	-6.19285100	-0.61841000	-0.22325100
H	-8.03985100	-0.61896900	0.95764200	H	-8.23968500	0.04179700	-0.47308900
H	-4.10032700	-1.05716000	-0.68090400	H	-4.05112200	-0.79464300	0.00927500
C	4.92619300	3.06878800	0.14486500	C	5.08823900	2.68101200	0.00277800
C	4.12739300	2.03026300	0.63366300	C	4.23430200	1.61881700	0.33414100
C	6.16992500	2.74144200	-0.40993800	C	6.30959400	2.38232900	-0.62412900
C	4.57838200	0.71891000	0.60806500	C	4.60084100	0.31324400	0.07959900
H	3.16116200	2.25184800	1.07041100	H	3.29923300	1.81364600	0.84456000
C	6.61825900	1.43336800	-0.42682400	C	6.68312800	1.08165800	-0.86131500
H	6.79318300	3.52348300	-0.82549700	H	6.96195100	3.18689200	-0.93858000
C	5.83796300	0.40350400	0.09946600	C	5.85105300	0.00946700	-0.48786400
H	3.95744800	-0.06799300	1.02120800	H	3.93926000	-0.49018300	0.37706600
H	7.58990500	1.20273800	-0.84856800	H	7.62550700	0.88060500	-1.35416800
C	5.51970000	5.41469100	0.40162800	C	5.88157500	4.87820100	0.61683200
C	6.48262000	5.29691200	1.40515700	C	6.78699100	4.54242300	1.62141400
C	5.53193600	6.53546700	-0.42963500	C	6.03375300	6.05861000	-0.10761900
C	7.44713100	6.28205700	1.56450300	C	7.84447000	5.39561000	1.90023700
H	6.47057700	4.43011600	2.05573300	H	6.65054800	3.62472700	2.18106400
C	6.48933100	7.52455900	-0.25345600	C	7.09544500	6.90447400	0.17890100
H	4.78617300	6.62508200	-1.21103100	H	5.32845200	6.29860500	-0.89450700
C	7.45389400	7.40149300	0.74004400	C	8.00095700	6.57607100	1.18159500
H	8.18976600	6.17868200	2.34796100	H	8.54367900	5.14066000	2.68785000
H	6.48783500	8.39044200	-0.90629100	H	7.21913500	7.81912300	-0.38885700
H	8.20480700	8.17215200	0.87132700	H	8.82866300	7.23947900	1.40317600
C	-7.03434400	4.18422700	-0.23093400	C	-6.34355700	4.61055600	-1.62228400
C	-8.08994800	3.72070300	-1.03208600	C	-7.14205900	4.17496400	-2.69437900
C	-7.21554300	5.41697200	0.41601900	C	-6.53608500	5.93907800	-1.20357800
C	-9.25730400	4.45514800	-1.19456200	C	-8.07234400	5.00550100	-3.30667700
H	-7.98696100	2.77129300	-1.54738000	H	-7.01954800	3.16075400	-3.06078500
C	-8.39282300	6.14214100	0.28509800	C	-7.47402100	6.77312800	-1.79790600
H	-6.42094500	5.80686500	1.04385300	H	-5.93925500	6.32310400	-0.38165300
C	-9.41434100	5.66541000	-0.52842000	C	-8.24728000	6.30977700	-2.85731700
H	-10.04901200	4.08064200	-1.83468400	H	-8.66343500	4.63396400	-4.13785500
H	-8.51167000	7.08324700	0.81141000	H	-7.60291200	7.78822800	-1.43540500
H	-10.33044100	6.23504000	-0.64280700	H	-8.97730800	6.95928100	-3.32894900
C	-5.06624400	-3.51600000	0.39707000	C	-5.34921100	-3.04116600	0.56576300
C	-4.60517900	-4.51524400	-0.47217300	C	-4.74157100	-4.05747100	-0.18699300
C	-4.39942300	-3.36965100	1.62167200	C	-4.89690100	-2.87368300	1.88303400
C	-3.49548400	-5.28585400	-0.15667300	C	-3.68788900	-4.80252600	0.31838500
H	-5.10241400	-4.66411500	-1.42580500	H	-5.07781500	-4.23526300	-1.20446300
C	-3.36271300	-4.21929100	1.98460500	C	-3.91201800	-3.68963900	2.42552100
H	-4.71657700	-2.59354300	2.31165600	H	-5.33511900	-2.09138800	2.49593100
C	-2.86517200	-5.17645500	1.09031700	C	-3.25362900	-4.65326100	1.64624000
H	-3.12151300	-6.00069800	-0.88355700	H	-3.19562900	-5.52904200	-0.32166600
H	-2.90762300	-4.11413800	2.96496000	H	-3.61458200	-3.54790000	3.46075100
C	5.53048200	-1.97758900	-0.33079200	C	5.29911900	-2.34405100	-0.81543900
C	5.28198200	-3.09568100	0.46935300	C	5.04338900	-3.21029400	0.24429600
C	4.97075100	-1.92319700	-1.60960600	C	4.65257900	-2.53143900	-2.03513200
C	4.50682800	-4.13913800	-0.00588000	C	4.15260900	-4.25873500	0.08346200
H	5.70889300	-3.14487800	1.46414700	H	5.55670300	-3.06617500	1.18818200
C	4.18517600	-2.96474300	-2.07939500	C	3.77219800	-3.58958600	-2.20086200
H	5.15697200	-1.05712000	-2.23410300	H	4.85017100	-1.84880000	-2.85387100
C	3.94992300	-4.08471400	-1.28321600	C	3.51726400	-4.46479500	-1.14284000
H	4.31683600	-5.00476200	0.61844700	H	3.95011600	-4.93672500	0.90405800
H	3.75633000	-2.91902000	-3.07402100	H	3.27587900	-3.74405900	-3.15167000
C	-1.66809600	-7.07252700	2.62208000	C	-2.06654100	-6.15389500	3.58581500
C	-0.49966600	-7.56563200	3.22638900	C	-0.88607700	-6.47725500	4.27490700
C	-2.89747000	-7.49718400	3.15290000	C	-3.28381000	-6.46383000	4.21455900
C	-0.55386800	-8.43999700	4.30267200	C	-0.91616700	-7.07359200	5.52785400
H	0.46639800	-7.24168800	2.85464600	H	0.07162500	-6.24264500	3.82212400
C	-2.96042200	-8.38535200	4.21704600	C	-3.32298500	-7.07608900	5.45966700
H	-3.81772600	-7.12653800	2.71380100	H	-4.21313400	-6.22664400	3.70779900
C	-1.78623800	-8.85623600	4.79501700	C	-2.13714300	-7.37873000	6.12009100
H	0.36234600	-8.79804600	4.75956800	H	0.00993500	-7.30325400	6.04407100
H	-3.92214900	-8.70704100	4.60166200	H	-4.27652600	-7.31444400	5.91844800
H	-1.83152000	-9.54398800	5.63279200	H	-2.16432300	-7.85111800	7.09638700
C	-0.35611400	-5.90096900	0.52987300	C	-0.77822400	-5.56029000	1.25473100

C	0.49902900	-6.94805000	0.14908200	C	0.00669600	-6.71087600	1.08139700
C	1.62567000	-6.72964000	-0.62757100	C	1.10818000	-6.72582800	0.23805900
C	1.96657300	-5.42964600	-1.01806800	C	1.48452500	-5.56251600	-0.43754900
C	1.13322100	-4.37117500	-0.64598900	C	0.73243600	-4.40041300	-0.26623100
C	-0.01439100	-4.61349700	0.08560700	C	-0.38720600	-4.41341700	0.54734100
H	0.27397800	-7.96201100	0.46402500	H	-0.25685200	-7.62065300	1.61141700
H	2.26221100	-7.55851600	-0.91518100	H	1.68841600	-7.63196500	0.10388700
H	1.38679200	-3.36310900	-0.95258200	H	1.01523800	-3.49845400	-0.79718700
H	-0.66177000	-3.77839900	0.33199100	H	-0.98080900	-3.50991900	0.63519400
C	7.56505600	-1.18993400	0.76486800	C	7.62435700	-1.67048700	-0.62548800
C	8.34984800	-2.27077100	0.34535200	C	8.10852500	-2.64534100	-1.49926100
C	8.03683800	-0.38352200	1.80655400	C	8.48882700	-1.10478300	0.31564800
C	9.56383300	-2.53792300	0.95981800	C	9.43816100	-3.03665000	-1.43726400
H	8.00512400	-2.89769600	-0.46820200	H	7.44091700	-3.09441000	-2.22453400
C	9.26055100	-0.65188700	2.40270100	C	9.81934100	-1.49350400	0.36031500
H	7.43931400	0.45240600	2.14907300	H	8.11264400	-0.36948400	1.01702700
C	10.03284000	-1.73096600	1.99010400	C	10.30207900	-2.46069300	-0.51378000
H	10.15468600	-3.38043400	0.61681400	H	9.80087900	-3.79345000	-2.12354400
H	9.60496600	-0.01368700	3.20937700	H	10.47828600	-1.04855800	1.09745500
H	10.98545200	-1.93945900	2.46306500	H	11.34052400	-2.76750300	-0.47074000
C	3.55384800	-5.94170200	-2.82795200	C	2.75938000	-6.45766800	-2.33919400
C	4.91017600	-6.04309200	-3.15711500	C	4.03159400	-6.77166500	-2.83085100
C	2.61838600	-6.61820500	-3.62063700	C	1.64346000	-7.07399400	-2.91533200
C	5.31289900	-6.80034400	-4.24773100	C	4.17665300	-7.67109700	-3.87652200
H	5.64809100	-5.52878700	-2.55372100	H	4.90647400	-6.31353400	-2.38512600
C	3.03601000	-7.38409800	-4.69821000	C	1.80407400	-7.98483800	-3.94921200
H	1.56246500	-6.53493600	-3.39383600	H	0.65071800	-6.83434900	-2.55476600
C	4.38398700	-7.48132100	-5.02457700	C	3.06744200	-8.28866300	-4.44222500
H	6.36989400	-6.86403600	-4.48290300	H	5.17208300	-7.90043400	-4.24134600
H	2.29266100	-7.89824100	-5.29793000	H	0.92505700	-8.45034100	-4.38148300
H	4.70404800	-8.07577300	-5.87235100	H	3.18572800	-8.99660000	-5.25422100
C	-7.59406900	-3.20922700	-0.52716000	C	-7.78543300	-2.81461100	-0.55814200
C	-8.49373300	-2.46621700	-1.30987600	C	-8.52572700	-2.35726600	-1.66245900
C	-7.93240500	-4.54438600	-0.24929700	C	-8.26277100	-3.97327100	0.07988500
C	-9.66656700	-3.02666100	-1.79581500	C	-9.67665900	-3.00403500	-2.09362300
H	-8.25921900	-1.43523000	-1.55152300	H	-8.17680400	-1.48576100	-2.20589400
C	-9.11319200	-5.10587800	-0.71399900	C	-9.42048300	-4.61789500	-0.33361700
H	-7.25882900	-5.14579300	0.35172100	H	-7.71126300	-4.37121500	0.92604500
C	-9.98034600	-4.34721500	-1.49289200	C	-10.13435700	-4.13451800	-1.42521800
H	-10.33830700	-2.43559200	-2.40881800	H	-10.21732100	-2.62742900	-2.95612100
H	-9.35722000	-6.13526200	-0.47485100	H	-9.76547700	-5.50275600	0.19179800
H	-10.90012400	-4.78570900	-1.86522500	H	-11.03578900	-4.63911700	-1.75698400
C	-0.81421200	7.77325000	-0.15469900	C	-0.46085300	7.64287500	0.98494400
C	0.01057100	8.44852700	-1.05503200	C	0.39197100	8.65115800	0.52910700
C	-1.68815500	8.50346300	0.65278700	C	-1.52565600	7.96571700	1.82723600
C	-0.03802900	9.83277700	-1.14197500	C	0.19383400	9.96187200	0.93695600
H	0.68857400	7.88237700	-1.68297800	H	1.19492500	8.40726700	-0.15654000
C	-1.74193600	9.88603900	0.54855700	C	-1.71761300	9.28029200	2.22374100
H	-2.32251400	7.98147100	1.35967900	H	-2.19704200	7.18560400	2.16438900
C	-0.91602400	10.55833400	-0.34530400	C	-0.85870100	10.28258200	1.78626900
H	0.60796200	10.34507100	-1.84637200	H	0.85676400	10.73859900	0.57294500
H	-2.42489200	10.44135900	1.18189800	H	-2.54479700	9.52092900	2.88145700
H	-0.95546200	11.63906200	-0.41902000	H	-1.01520000	11.30887300	2.09717800
B	-1.62014700	-6.07689200	1.42302400	B	-2.04183400	-5.47788300	2.17792300
B	-6.26666200	-2.58928600	0.01128900	B	-6.46506600	-2.11536800	-0.06832600
B	-5.70607500	3.36173400	-0.05986300	B	-5.27553300	3.66953800	-0.93885800
N	-0.74489900	6.36101800	-0.04706500	N	-0.28657000	6.30360500	0.55477700
N	4.53010600	4.41335900	0.22889500	N	4.79013000	4.00808000	0.32495200
N	6.33462700	-0.92080000	0.14333300	N	6.25911200	-1.29878900	-0.66905100
N	3.14079900	-5.15776300	-1.74246500	N	2.61539300	-5.54160400	-1.28589300

Structure S24. The coordinates of both the optimized structures of B2N5L_1 at S_0 and S_1 in toluene.

S_0-optimized structure				S_1-optimized structure			
C	1.74136900	5.46469100	0.08206500	C	3.36247500	4.77199700	0.44559300
C	2.71406300	5.80722800	1.02135400	C	4.52562000	4.78609000	1.24811400
C	1.95500400	4.34239800	-0.71909900	C	3.13449000	3.63623400	-0.36330600
C	3.88185600	5.07531100	1.13131100	C	5.41566900	3.74545700	1.21945700
H	2.55389700	6.66351600	1.66608600	H	4.69879500	5.61262300	1.92405500
C	3.12770800	3.60861500	-0.61395700	C	4.03733300	2.60065600	-0.40178700
H	1.21311400	4.06311700	-1.45857100	H	2.25993400	3.60683500	-0.99970600
C	4.12192500	3.97726900	0.29832100	C	5.20498300	2.62873300	0.38550200
H	4.62460900	5.36184400	1.86557500	H	6.27617700	3.76790900	1.87534700
H	3.28845400	2.76380300	-1.27308400	H	3.86589800	1.77641000	-1.08257500
C	-0.68978500	5.78804900	-0.12618000	C	1.11436100	5.63864900	0.07520100
C	-1.69116300	6.46220200	-0.84095200	C	0.61088600	6.30434200	-1.03898900
C	-1.03800700	4.61011500	0.55198400	C	0.26532300	4.86689600	0.86539800
C	-2.99091600	5.99845000	-0.83022100	C	-0.73726700	6.20547300	-1.34249100
H	-1.44065000	7.35518200	-1.40005500	H	1.27557500	6.90571100	-1.65002800
C	-2.34315900	4.14935100	0.52777200	C	-1.08175800	4.78206500	0.54405900
H	-0.28340300	4.07528100	1.11649200	H	0.65777100	4.36729100	1.74499600
C	-3.37463300	4.82922200	-0.14672500	C	-1.63630500	5.45214400	-0.56222600
H	-3.73668100	6.55042700	-1.39165600	H	-1.11406900	6.73815600	-2.20881900
H	-2.57841100	3.24929400	1.08631500	H	-1.73382600	4.20693600	1.19193200
C	-5.26892900	2.85856600	0.01253600	C	-4.06113500	4.21596000	-0.58072300
C	-6.52831500	2.50766100	0.52922700	C	-5.48011800	4.31096200	-0.49620900
C	-4.43621000	1.80434500	-0.39498100	C	-3.54593500	2.90539900	-0.36978900
C	-6.94865900	1.18821000	0.59238400	C	-6.28665600	3.22558400	-0.25204500
H	-7.19646300	3.29098000	0.87105300	H	-5.94118200	5.28751800	-0.60472400
C	-4.84178400	0.48058700	-0.30017700	C	-4.35264500	1.82089000	-0.11879000
H	-3.46034400	2.02909600	-0.81245600	H	-2.47472800	2.74664900	-0.44898100
C	-6.11909100	0.13850000	0.16967200	C	-5.77108500	1.91339700	-0.06228300
H	-7.94037800	0.96410600	0.97282800	H	-7.35829800	3.38074700	-0.17442700
H	-4.16809400	-0.30600000	-0.62539700	H	-3.89192100	0.84490100	0.00161200
C	5.47035500	1.91269900	0.20006300	C	5.84762500	0.27749700	0.08702600
C	4.47909300	1.03213500	0.64135200	C	4.60539600	-0.28753700	0.41699400
C	6.61922000	1.37397000	-0.39070000	C	6.82623500	-0.55454300	-0.48413400
C	4.65000000	-0.33949900	0.52877200	C	4.36158100	-1.62716300	0.20348300
H	3.58093800	1.42082800	1.10628500	H	3.84304000	0.31782600	0.89113000
C	6.78923100	0.00550400	-0.49643300	C	6.59093100	-1.89460300	-0.67635400
H	7.38808200	2.03745200	-0.76729700	H	7.77367700	-0.13347900	-0.79466200
C	5.81510500	-0.87451000	-0.02179900	C	5.35862400	-2.47126800	-0.31721600
H	3.88182800	-1.00610800	0.90379800	H	3.40753600	-2.04697200	0.49579500
H	7.69008000	-0.39116900	-0.95031500	H	7.35755700	-2.51018200	-1.12903200
C	6.52302700	4.05663800	0.65095200	C	7.50607400	1.95109400	0.62945700
C	7.39268600	3.65690700	1.66695300	C	8.18861400	1.29526600	1.65243300
C	6.81461600	5.20641800	-0.08480400	C	8.13975800	2.93747600	-0.12380300
C	8.53875600	4.39272600	1.93364400	C	9.50767300	1.63146000	1.91886100
H	7.16488000	2.76739600	2.24273000	H	7.68055000	0.53701200	2.23631200
C	7.95363100	5.94644500	0.19930000	C	9.45918300	3.26618600	0.15133000
H	6.14282800	5.51503100	-0.87734000	H	7.59967500	3.43112900	-0.92299200
C	8.82356900	5.54233200	1.20562500	C	10.14507500	2.61555500	1.17090000
H	9.20584100	4.07099700	2.72575500	H	10.03611300	1.12810600	2.71992200
H	8.16824300	6.83768000	-0.38001000	H	9.95448100	4.02805500	-0.43903900
H	9.71614900	6.11847600	1.42087400	H	11.17575600	2.87479500	1.38254600
C	-5.99134500	5.42858800	-0.37760700	C	-3.79826100	6.73585200	-1.52701400
C	-7.10942200	5.15821600	-1.18255500	C	-4.76216200	6.67310000	-2.54868000
C	-5.93052800	6.69317900	0.22918700	C	-3.40174200	8.02349400	-1.12444500
C	-8.10501900	6.10444200	-1.38725100	C	-5.29706300	7.81592700	-3.12982500
H	-7.19257800	4.19060300	-1.66666100	H	-5.09035600	5.69999800	-2.89976500
C	-6.93788000	7.63349100	0.05583600	C	-3.94118900	9.17272400	-1.68740600
H	-5.08175100	6.93730400	0.85968000	H	-2.65683500	8.12328700	-0.34063100
C	-8.02480000	7.34310500	-0.76076300	C	-4.89196000	9.07420500	-2.69794500
H	-8.94860400	5.87387500	-2.02913200	H	-6.03274200	7.72503900	-3.92275800
H	-6.87253400	8.59604500	0.55182600	H	-3.61938300	10.14907200	-1.33863800
H	-8.80756400	8.07958200	-0.90807000	H	-5.31180500	9.96914200	-3.14521800
C	-5.58171300	-2.47392900	0.55656100	C	-6.03209100	-0.65565200	0.70742400
C	-5.38635100	-3.62020400	-0.22737600	C	-6.23687300	-1.88170600	0.05619200
C	-4.81275900	-2.35451200	1.72440500	C	-5.22923600	-0.68177100	1.85880200
C	-4.44387100	-4.57897300	0.11211500	C	-5.63079100	-3.05284500	0.48717200
H	-5.97153100	-3.75471200	-1.13133500	H	-6.87225800	-1.91285100	-0.82337900
C	-3.91205100	-3.33629300	2.10705800	C	-4.64432700	-1.85248100	2.32385000
H	-4.92278400	-1.47176100	2.34622000	H	-5.05100500	0.24329200	2.39777600
C	-3.70421200	-4.44603400	1.28612200	C	-4.82236700	-3.04185500	1.62126100
H	-4.27797600	-5.44336000	-0.52101800	H	-5.77159300	-3.98317700	-0.05284100
H	-3.33846400	-3.23609300	3.02180500	H	-4.02589400	-1.84852300	3.21536500
C	4.96520900	-3.10082600	-0.52461600	C	3.81572100	-4.30637300	-0.71553700
C	4.50705700	-4.15477800	0.26882200	C	3.13417800	-5.02938900	0.26064500
C	4.36100900	-2.87440100	-1.76298200	C	3.21650400	-4.08468700	-1.95368000
C	3.47395400	-4.96492000	-0.17009700	C	1.86885700	-5.52668000	0.00004200
H	4.97172000	-4.33882000	1.23049400	H	3.60592900	-5.20837900	1.22016500
C	3.31654700	-3.67707200	-2.19460700	C	1.95451400	-4.59087500	-2.21900400
H	4.71174500	-2.05738600	-2.38300200	H	3.74704400	-3.51683200	-2.70975200
C	2.86291400	-4.73241900	-1.40245400	C	1.26684500	-5.32183000	-1.24531000
H	3.11995000	-5.77985300	0.45090300	H	1.33640000	-6.08746000	0.75892300
H	2.84860600	-3.49254800	-3.15495200	H	1.49291700	-4.42171600	-3.18449400
C	-2.74224700	-6.09049100	2.82254300	C	-4.49449800	-4.88803200	3.19751300
C	-1.56742200	-6.55007400	3.42958300	C	-3.57502400	-5.67947400	3.89854800
C	-3.96450000	-6.32785000	3.46473200	C	-5.78379100	-4.73964800	3.72432400
C	-1.62231900	-7.23317200	4.63594000	C	-3.94597200	-6.31355000	5.07490800
H	-0.61030800	-6.36598800	2.95770700	H	-2.56441600	-5.78876100	3.52478500
C	-4.00270600	-6.99883700	4.67728000	C	-6.13681800	-5.36871600	4.90863200
H	-4.88481200	-5.98776600	3.00546000	H	-6.50747800	-4.12882000	3.19888000
C	-2.83544200	-7.46230000	5.27333300	C	-5.22702100	-6.16572400	5.59317500
H	-0.69792000	-7.57846500	5.08651900	H	-3.21422400	-6.91978600	5.59848900
H	-4.96192400	-7.16984500	5.15409500	H	-7.14240100	-5.23728500	5.29360400
H	-2.87103900	-7.99241400	6.21788400	H	-5.50988800	-6.65958900	6.51551800
C	-1.59060200	-5.46524200	0.74163500	C	-3.09378500	-4.66074200	1.18139500

C	-1.09807300	-6.68193500	0.26338700	C	-2.89602300	-6.01206600	0.87238100
C	0.00515100	-6.71532500	-0.57459400	C	-1.88049000	-6.39916600	0.01253900
C	0.62800600	-5.53037700	-0.97659800	C	-1.05061700	-5.44733700	-0.57994400
C	0.12223900	-4.31590000	-0.51284000	C	-1.25022400	-4.10151900	-0.28002400
C	-0.96394300	-4.28436000	0.34355100	C	-2.24855600	-3.71366200	0.59489600
H	-1.57720500	-7.60626100	0.56537700	H	-3.54695100	-6.76130600	1.30699800
H	0.38602500	-7.66586900	-0.93001400	H	-1.74163200	-7.44831000	-0.22338700
H	0.59733300	-3.39066500	-0.81719000	H	-0.61531000	-3.35229600	-0.73935000
H	-1.34037600	-3.33357700	0.70215400	H	-2.39504400	-2.66289800	0.81228600
C	7.20027700	-2.84186000	0.41971100	C	6.17428500	-4.77763600	-0.35298300
C	7.68857000	-4.03962600	-0.11557100	C	6.17887800	-5.90632200	-1.17521700
C	7.91323500	-2.23147700	1.45764400	C	7.18072000	-4.62496800	0.60518000
C	8.85146000	-4.60868500	0.38113700	C	7.17992700	-6.85763000	-1.04489800
H	7.15335200	-4.51979000	-0.92596500	H	5.39760200	-6.03374900	-1.91459500
C	9.08401100	-2.80239400	1.93506000	C	8.18364500	-5.57593900	0.71777400
H	7.54485800	-1.30962200	1.89076900	H	7.16716400	-3.76758500	1.26756200
C	9.56231400	-3.99523700	1.40627600	C	8.19018100	-6.69711100	-0.10420400
H	9.21090100	-5.53664900	-0.05042100	H	7.17168300	-7.72786100	-1.69139500
H	9.61911200	-2.31162800	2.74094500	H	8.95573600	-5.44630100	1.46790700
H	10.47453400	-4.43990800	1.78671700	H	8.97168400	-7.44162100	-0.00751500
C	1.80003200	-6.23463900	-3.02079300	C	-0.34067100	-6.46433800	-2.69405100
C	3.02115500	-6.62776000	-3.58278800	C	0.61493100	-7.25234200	-3.34702200
C	0.61791400	-6.54858900	-3.70271200	C	-1.61507500	-6.34731900	-3.25804000
C	3.05208300	-7.30866200	-4.79044700	C	0.30279300	-7.89061400	-4.53755300
H	3.94570700	-6.40090100	-3.06599100	H	1.60064600	-7.36883500	-2.91223700
C	0.66316200	-7.24288400	-4.90258500	C	-1.91936600	-7.00448000	-4.44180000
H	-0.33623000	-6.24097200	-3.29285600	H	-2.36515400	-5.73847000	-2.76866500
C	1.87668900	-7.62708500	-5.46043600	C	-0.96602000	-7.77614100	-5.09441300
H	4.01070600	-7.60271600	-5.20430100	H	1.05839600	-8.49688800	-5.02554400
H	-0.26647000	-7.47295800	-5.41210600	H	-2.91383100	-6.89864200	-4.86142700
H	1.90591700	-8.16473300	-6.40094100	H	-1.20832600	-8.28326400	-6.02104700
C	-8.02155500	-1.72824700	-0.36131300	C	-8.18533600	0.66036900	-0.23367300
C	-8.75881000	-0.87274200	-1.19708300	C	-8.70778000	1.38176900	-1.32134500
C	-8.62459000	-2.94127100	0.01178600	C	-9.09877300	-0.12127900	0.49543100
C	-10.02747700	-1.21264900	-1.64515600	C	-10.05558100	1.33703700	-1.65376800
H	-8.32054000	0.06862300	-1.51037600	H	-8.03518300	1.97742700	-1.92935400
C	-9.90151100	-3.27849600	-0.41412400	C	-10.45068800	-0.16016600	0.18153800
H	-8.08323400	-3.62396000	0.65807400	H	-8.73601700	-0.70575200	1.33532100
C	-10.60300700	-2.41494800	-1.24859700	C	-10.93603400	0.57054900	-0.89789400
H	-10.57070200	-0.54065800	-2.30066900	H	-10.42084900	1.90032300	-2.50660800
H	-10.34997200	-4.21498500	-0.10070300	H	-11.12825000	-0.76430900	0.77655700
H	-11.59804800	-2.67934800	-1.59042200	H	-11.99043700	0.53785300	-1.15166300
C	0.83694400	7.68815600	-0.22120100	C	2.86882300	7.12190700	0.86003400
C	1.81993100	8.15401000	-1.09527900	C	4.06153300	7.68384600	0.39766400
C	0.10438500	8.60529800	0.53504200	C	2.02698400	7.87014200	1.68450700
C	2.06476900	9.51539800	-1.20805200	C	4.41572100	8.96902400	0.78028600
H	2.38929900	7.44323900	-1.68274700	H	4.69788100	7.11860700	-0.27315000
C	0.34467800	9.96559100	0.40469800	C	2.38807300	9.15638400	2.05513700
H	-0.65169000	8.24560800	1.22313600	H	1.09419800	7.44029200	2.02826700
C	1.32699400	10.42833900	-0.46365100	C	3.58340300	9.71056300	1.61066000
H	2.83087400	9.86366800	-1.89195300	H	5.34007400	9.39847100	0.41085900
H	-0.23137800	10.66679300	0.99834000	H	1.72919300	9.72778600	2.69873700
H	1.51708200	11.49136500	-0.55761400	H	3.85935900	10.71747700	1.90126200
B	-6.58921800	-1.35520700	0.13421600	B	-6.65683100	0.68203700	0.14233800
B	-4.85729900	4.36312300	-0.15827100	B	-3.18280600	5.43253300	-0.88307000
N	0.60723300	6.29605300	-0.08658200	N	2.48190500	5.82026600	0.45648100
N	5.35512900	3.30470400	0.36796800	N	6.14924300	1.61402200	0.35071700
N	6.01903300	-2.27137300	-0.08208100	N	5.13457800	-3.82762200	-0.46692300
N	1.77066400	-5.53999500	-1.80640900	N	-0.02437100	-5.82665100	-1.48401500
N	-2.71443100	-5.41232200	1.59847200	N	-4.14148100	-4.23268300	2.01370700

Structure S25. The coordinates of both the optimized structures of B1N6L$_1$ at S$_0$ and S$_1$ in toluene.

S_0-optimized structure				S_1-optimized structure			
C	2.44435700	5.18165700	0.06079300	C	2.34501000	5.26282800	0.41375100
C	3.41107700	5.42595000	1.03616800	C	3.50552700	5.50225500	1.19036900
C	2.56109700	4.03079700	-0.71910900	C	2.34723700	4.13109600	-0.43796500
C	4.48115100	4.56646800	1.20478300	C	4.60016300	4.68751200	1.09426100
H	3.32366100	6.30492800	1.66420600	H	3.51371800	6.31697800	1.90150000
C	3.63640100	3.16982800	-0.55664300	C	3.44942100	3.31847600	-0.53395800
H	1.82223300	3.82710900	-1.48585500	H	1.47801600	3.93272500	-1.05030200
C	4.62654500	3.43448100	0.39549000	C	4.60978700	3.57601400	0.22462500
H	5.21995200	4.77670100	1.96827900	H	5.45741500	4.87519100	1.72766200
H	3.72575500	2.30226700	-1.19940900	H	3.43831600	2.49080000	-1.23216000
C	0.06980000	5.75791700	-0.21223200	C	-0.03949900	5.63867700	0.07262900
C	-0.83835500	6.49398300	-0.98662000	C	-0.72116200	6.34710200	-0.92305300
C	-0.41539400	4.66204300	0.51546600	C	-0.68331600	4.57493300	0.71865400
C	-2.18146700	6.17416600	-0.98165100	C	-2.01809000	6.00752100	-1.24496200
H	-0.48231800	7.32483400	-1.58335500	H	-0.22290000	7.16622300	-1.43184000
C	-1.76298800	4.34266800	0.48391700	C	-1.97904000	4.24236300	0.37457300
H	0.26640000	4.08153400	1.12582700	H	-0.16847400	4.03872800	1.50994000
C	-2.70189000	5.09237800	-0.24741500	C	-2.72583900	4.95159400	-0.60432200
H	-2.85403100	6.77508400	-1.58404600	H	-2.51830800	6.56898000	-2.02642800
H	-2.10493800	3.50652300	1.08517500	H	-2.46112200	3.43208800	0.91116700
C	-4.83023800	3.38147400	-0.03657000	C	-4.81168800	3.21734100	-0.64589400
C	-6.15006400	3.20618300	0.42106200	C	-6.16866500	3.02708200	-0.30061700
C	-4.12410800	2.20369400	-0.33617700	C	-4.05718500	2.03053900	-0.76378900
C	-6.73283600	1.96080000	0.55507900	C	-6.72411300	1.77781800	-0.08616400
H	-6.73375900	4.07829300	0.69480900	H	-6.80309400	3.89916600	-0.17914500
C	-4.69381800	0.94668600	-0.22462500	C	-4.60272500	0.77053500	-0.58415900
H	-3.10858600	2.27552200	-0.71097600	H	-3.01237600	2.10136400	-1.04994000
C	-6.01893400	0.80598500	0.20769600	C	-5.95009000	0.62408100	-0.24188000
H	-7.74762000	1.87163400	0.92417400	H	-7.76773500	1.68472600	0.19527400
H	-4.12549400	0.06629900	-0.50043800	H	-3.98965800	-0.11324000	-0.72538300
C	5.71695400	1.22383300	0.35493800	C	5.69565000	1.40725500	-0.12846500
C	4.61023500	0.47049700	0.75513300	C	4.63398600	0.60500500	0.30845300
C	6.80911000	0.55098000	-0.20423300	C	6.76421400	0.79351900	-0.79834800
C	4.60924000	-0.90961700	0.62438300	C	4.65504700	-0.76122700	0.11069000
H	3.75392700	0.96468400	1.19829700	H	3.80792400	1.04835500	0.85093600
C	6.80777700	-0.82681900	-0.32832400	C	6.79290400	-0.57075400	-0.98082900
H	7.66781300	1.11642500	-0.54517800	H	7.57384300	1.39891200	-1.18633400
C	5.71285100	-1.58115900	0.09673100	C	5.75041600	-1.38516600	-0.50681700
H	3.75067200	-1.47802100	0.96316700	H	3.83819900	-1.36421400	0.48667500
H	7.66531400	-1.32839800	-0.76175900	H	7.62611900	-1.02016600	-1.50592300
C	7.00198700	3.22342600	0.86422600	C	7.00835400	3.37752900	0.38610300
C	7.77441200	2.71846500	1.91150100	C	7.86472700	2.82472300	1.33764500
C	7.46219800	4.32971800	0.14800900	C	7.38949800	4.51341700	-0.32715200
C	8.99020300	3.30768000	2.22878000	C	9.09919400	3.41169800	1.57166800
H	7.41625800	1.86288300	2.47216500	H	7.55497500	1.94720200	1.89248800
C	8.67075400	4.92395100	0.48251100	C	8.62528000	5.09447800	-0.08285200
H	6.86604600	4.71970200	-0.66877800	H	6.72101500	4.92594700	-1.07365300
C	9.44303600	4.41481600	1.52038400	C	9.48260300	4.54637700	0.86453600
H	9.58006000	2.90526500	3.04491400	H	9.76079300	2.98538800	2.31669300
H	9.01595700	5.78311800	-0.08194300	H	8.92224300	5.97325300	-0.64325600
H	10.38982000	4.87713400	1.77497900	H	10.44811400	5.00189400	1.05082200
C	-5.21849200	6.00767600	-0.49625200	C	-5.14487700	5.75296200	-1.51455600
C	-6.34448200	5.87951100	-1.32451600	C	-6.19100000	5.44934400	-2.41060200
C	-5.00607100	7.25874000	0.10391500	C	-5.00705000	7.11686400	-1.18219100
C	-7.20563000	6.94497300	-1.55404900	C	-7.02421800	6.42371700	-2.94178400
H	-6.54059800	4.92793300	-1.80804200	H	-6.34197100	4.41508100	-2.70412300
C	-5.87982300	8.32014200	-0.09413500	C	-5.83872000	8.09859700	-1.70214600
H	-4.14350100	7.39671400	0.74789700	H	-4.23135100	7.40786600	-0.48052400
C	-6.97923500	8.16707300	-0.93119800	C	-6.85500900	7.76031100	-2.59139000
H	-8.05804200	6.82102400	-2.21358600	H	-7.80921800	6.14112600	-3.63690300
H	-5.69959400	9.27016800	0.39775700	H	-5.69944600	9.13455700	-1.40761100
H	-7.65690200	8.99756200	-1.09828400	H	-7.50589000	8.52498800	-3.00208000
C	-5.80741600	-1.58867000	0.58263000	C	-5.75969800	-1.66276400	0.55474500
C	-5.63718800	-2.59609100	-0.36578400	C	-5.58208700	-2.91516800	-0.04098700
C	-5.18715500	-1.70712100	1.82587600	C	-5.12758200	-1.40469300	1.77578900
C	-4.85805500	-3.70382900	-0.07510300	C	-4.77885600	-3.87016300	0.55807500
H	-6.12477600	-2.50715900	-1.32975800	H	-6.06767100	-3.13086600	-0.98520800
C	-4.40243700	-2.81272400	2.11509000	C	-4.31205400	-2.35693300	2.36513000
H	-5.31925900	-0.92203400	2.56158700	H	-5.26238000	-0.43776800	2.24596300
C	-4.23032200	-3.82116100	1.16551300	C	-4.11957100	-3.59679600	1.75541600
H	-4.72235000	-4.48613800	-0.81285800	H	-4.63052400	-4.83174500	0.07957800
H	-3.91904500	-2.90171500	3.08114000	H	-3.81258600	-2.14018300	3.30285500
C	4.58104600	-3.65405300	-0.48429500	C	4.59400100	-3.51119700	-0.75465100
C	3.95853700	-4.65069600	0.27009800	C	4.15593400	-4.30463800	0.30335200
C	4.04549600	-3.31306400	-1.72797900	C	3.85960200	-3.47290400	-1.93814700
C	2.82999100	-5.29201400	-0.21131800	C	2.99821200	-5.05493200	0.17724700
H	4.36926500	-4.92339400	1.23526300	H	4.73436200	-4.33857600	1.21952900
C	2.90665400	-3.94501800	-2.20190900	C	2.70592400	-4.22996500	-2.06786100
H	4.52427400	-2.53988300	-2.31775700	H	4.20100200	-2.84975500	-2.75701900
C	2.28701000	-4.94222600	-1.44788000	C	2.26590400	-5.03121300	-1.01141700
H	2.34796200	-6.06264200	0.37926000	H	2.65467400	-5.67400200	0.99764800
H	2.49285400	-3.67088000	-3.16568400	H	2.13870900	-4.20693800	-2.99091800
C	-3.61843700	-5.73641100	2.55936800	C	-3.43775300	-5.11440900	3.55722000
C	-2.55965600	-6.39433200	3.19611600	C	-2.37774500	-5.62308800	4.31898400
C	-4.91055400	-5.88864100	3.07711200	C	-4.72985900	-5.16237400	4.09544500
C	-2.79505300	-7.18620300	4.31057600	C	-2.61250000	-6.17312900	5.57021200
H	-1.55105300	-6.27926700	2.81867900	H	-1.36748800	-5.57823700	3.93102700
C	-5.12940200	-6.67069600	4.20104700	C	-4.94775700	-5.70232900	5.35407700
H	-5.74321800	-5.39492600	2.59075800	H	-5.56266400	-4.77602400	3.52079700
C	-4.07747800	-7.33011600	4.82615500	C	-3.89585700	-6.21748000	6.10211100
H	-1.95831300	-7.68526200	4.78748800	H	-1.77421100	-6.55933500	6.14034600
H	-6.13948400	-6.77349000	4.58284000	H	-5.95840400	-5.72758500	5.74728800
H	-4.25444500	-7.94561400	5.70041300	H	-4.07236700	-6.64335500	7.08292300
C	-2.27592200	-5.11849500	0.59470100	C	-2.10523900	-4.88652300	1.49802600

C	-1.95238100	-6.37175900	0.06802700	C	-1.71773100	-6.21380600	1.28630300
C	-0.84450700	-6.52891400	-0.74902500	C	-0.65741700	-6.51325900	0.44595000
C	-0.04690200	-5.43152000	-1.08508000	C	0.02690500	-5.49817400	-0.22561700
C	-0.38271600	-4.17795300	-0.57257500	C	-0.35449400	-4.17462900	-0.00908500
C	-1.47311300	-4.02585500	0.26541200	C	-1.39648200	-3.87264800	0.84941200
H	-2.56704000	-7.22954500	0.31571400	H	-2.25517000	-7.01378800	1.78197900
H	-0.59742000	-7.50870200	-1.14128100	H	-0.37088600	-7.54623200	0.28404800
H	0.22879200	-3.31921100	-0.82368400	H	0.16940500	-3.37635500	-0.52212000
H	-1.71452200	-3.04735000	0.66375600	H	-1.68758300	-2.84019100	1.00168200
C	6.81062000	-3.73130100	0.50598200	C	7.01971000	-3.48086000	-0.59714300
C	7.13839400	-4.97074900	-0.05610400	C	7.18680700	-4.61991400	-1.38825200
C	7.58165700	-3.25316900	1.57141800	C	8.05322500	-3.09019700	0.25974500
C	8.20159500	-5.70888300	0.44153200	C	8.36767500	-5.34544100	-1.32652600
H	6.55736400	-5.35020800	-0.88806900	H	6.38905300	-4.93313500	-2.05077500
C	8.65281200	-3.99373800	2.04975200	C	9.23513100	-3.81446700	0.30233300
H	7.33643500	-2.30087200	2.02546900	H	7.92414000	-2.22477600	0.89863300
C	8.97091200	-5.22738400	1.49448700	C	9.40110500	-4.94609300	-0.48787300
H	8.43702400	-6.66609100	-0.01110100	H	8.48088100	-6.22640000	-1.94836100
H	9.23563600	-3.60351300	2.87718200	H	10.02593000	-3.49902300	0.97388200
H	9.80540900	-5.80430900	1.87574000	H	10.32363000	-5.51320000	-0.44617900
C	1.05965100	-6.23135300	-3.13053700	C	0.88121500	-6.63610600	-2.22505800
C	2.22540000	-6.78232300	-3.67718500	C	1.96870200	-7.28663400	-2.82068400
C	-0.13709400	-6.35192300	-3.84759400	C	-0.39844700	-6.85141300	-2.74766900
C	2.18925000	-7.42812000	-4.90385400	C	1.77662500	-8.11857100	-3.91308300
H	3.15922900	-6.70568500	-3.13366100	H	2.96430800	-7.14247700	-2.41814600
C	-0.16145000	-7.01355400	-5.06637900	C	-0.57865800	-7.69930100	-3.83119000
H	-1.04714400	-5.92011800	-3.44964700	H	-1.25077200	-6.35013400	-2.30615300
C	0.99805700	-7.55489400	-5.60862300	C	0.50315700	-8.33644700	-4.42636400
H	3.10581100	-7.84699800	-5.30526200	H	2.63397300	-8.61252800	-4.35755800
H	-1.10093200	-7.09274900	-5.60287900	H	-1.57985500	-7.84998500	-4.22017400
H	0.97392600	-8.06606200	-6.56391800	H	0.35651200	-8.99348400	-5.27563200
C	-7.97542400	-0.66590200	-0.00508100	C	-7.72663600	-0.99484300	-0.71711300
C	-8.61950200	0.06552200	-1.00786900	C	-8.08785700	-0.40011000	-1.93086200
C	-8.69587700	-1.64318300	0.68720400	C	-8.59678000	-1.92906800	-0.14231400
C	-9.95661000	-0.16787600	-1.29345400	C	-9.28758200	-0.73079200	-2.54389900
H	-8.06818300	0.81365700	-1.56450600	H	-7.42442500	0.32320800	-2.38859500
C	-10.02834900	-1.87994800	0.38179100	C	-9.78567900	-2.26388700	-0.77243400
H	-8.20610700	-2.21500300	1.46640800	H	-8.33622700	-2.38665600	0.80486500
C	-10.67056900	-1.14207100	-0.60503200	C	-10.14438800	-1.66618000	-1.97551900
H	-10.43862500	0.40925400	-2.07502500	H	-9.54692700	-0.25658000	-3.48442100
H	-10.56991500	-2.64259600	0.93068800	H	-10.44475900	-2.98922800	-0.30732100
H	-11.71337500	-1.32516000	-0.83686700	H	-11.07893200	-1.92321100	-2.46082600
C	1.79622400	7.48152000	-0.32500100	C	1.33615100	7.38342700	1.04106400
C	2.87465200	7.81059500	-1.14822000	C	2.34249200	8.26170600	0.62984400
C	1.12096100	8.49938800	0.35264700	C	0.36347200	7.81918800	1.94318300
C	3.26912700	9.13374400	-1.28757700	C	2.38797800	9.55037500	1.14126600
H	3.40034700	7.02342700	-1.67585300	H	3.07153800	7.93697900	-0.10330500
C	1.51176600	9.82116400	0.19465200	C	0.41877900	9.10887300	2.44684900
H	0.29033900	8.24822900	1.00170300	H	-0.42975300	7.14349700	2.23850000
C	2.58882300	10.14670000	-0.62213000	C	1.43067300	9.97916600	2.05309000
H	4.10829300	9.37351900	-1.93129700	H	3.16723100	10.22746400	0.80997200
H	0.97785000	10.60040600	0.72735800	H	-0.33667900	9.43627200	3.15180600
H	2.89577500	11.17988200	-0.73724300	H	1.46478100	10.98894600	2.44520200
B	-4.23352200	4.80503900	-0.24928400	B	-4.21112500	4.63664400	-0.91782700
N	1.41464500	6.12808900	-0.16224200	N	1.25338700	6.07575500	0.50095500
N	5.76440300	2.61927400	0.53034400	N	5.73947200	2.78311400	0.13797700
N	5.72854400	-2.99061800	0.00377200	N	5.80272700	-2.76626400	-0.64127900
N	1.09976700	-5.57198600	-1.89659300	N	1.08391700	-5.79609000	-1.11918000
N	-3.40202300	-4.93890700	1.42776900	N	-3.21841700	-4.55365700	2.29541500
N	-6.61316400	-0.45705200	0.29101600	N	-6.52220100	-0.66220800	-0.07465000

Structure S26. The coordinates of both the optimized structures of N7L$_1$ at S$_0$ and S$_1$ in toluene.

S$_0$-optimized structure				S$_1$-optimized structure			
C	1.23209900	5.52208500	0.03190900	C	1.21263900	5.48165000	0.12532600
C	2.27408800	6.00412900	0.82892100	C	2.20110000	5.96590600	1.01928100
C	1.46688900	4.38724200	-0.74892000	C	1.50659000	4.32935700	-0.62934800
C	3.51817800	5.40173700	0.81112900	C	3.43069000	5.36246800	1.09471900
H	2.10569600	6.86580100	1.46370400	H	1.97472800	6.81101200	1.65772700
C	2.71695000	3.78487500	-0.76847100	C	2.74447900	3.72955700	-0.56029700
H	0.67478000	3.99499900	-1.37625100	H	0.76108800	3.94204800	-1.31473900
C	3.76852300	4.29480200	-0.00341400	C	3.75411900	4.25311600	0.28712600
H	4.31256900	5.79666300	1.43317700	H	4.16805700	5.74147400	1.79242600
H	2.88647000	2.92806800	-1.41033500	H	2.96448900	2.88088900	-1.19627800
C	-1.23137400	5.52223200	-0.03191200	C	-1.21445900	5.48125300	-0.12530000
C	-2.27327700	6.00444300	-0.82893200	C	-2.20308500	5.96523900	-1.01926800
C	-1.46632800	4.38738200	0.74885500	C	-1.50807300	4.32885500	0.62936500
C	-3.51744300	5.40220600	-0.81121100	C	-3.43247600	5.36143000	-1.09471500
H	-2.10475400	6.86612600	-1.46366800	H	-1.97695600	6.81040700	-1.65771700
C	-2.71646400	3.78516800	0.76833700	C	-2.74576100	3.72866600	0.56029900
H	-0.67429000	3.99500900	1.37619400	H	-0.76245800	3.94177000	1.31476100
C	-3.76795300	4.29526200	0.00326900	C	-3.75558000	4.25195800	-0.28712900
H	-4.31176300	5.79726200	-1.43326700	H	-4.16995100	5.74020100	-1.79243800
H	-2.88611100	2.92835100	1.41015400	H	-2.96552100	2.87992600	1.19626800
C	-5.31636600	2.37562100	0.02174500	C	-5.30941600	2.35321300	-0.18628400
C	-6.47984700	1.84807300	0.59804100	C	-6.51908200	1.92027600	0.38382500
C	-4.43289100	1.49195600	-0.60760800	C	-4.38939600	1.37696600	-0.61302900
C	-6.76807400	0.49809000	0.50824400	C	-6.82433500	0.57965600	0.47301200
H	-7.16540500	2.50497300	1.11863600	H	-7.22202500	2.65208600	0.76278200
C	-4.72317200	0.13860300	-0.68664400	C	-4.69805300	0.03305200	-0.51510100
H	-3.52664900	1.86985300	-1.06472400	H	-3.45266700	1.68061100	-1.06356400
C	-5.90091000	-0.37575900	-0.14691600	C	-5.92432700	-0.39404900	0.00728900
H	-7.67846400	0.11099900	0.95186400	H	-7.76584900	0.27017900	0.91123000
H	-4.03816100	-0.52666000	-1.20030300	H	-3.99235300	-0.70378000	-0.88177700
C	5.31668600	2.37496900	-0.02188900	C	5.30858000	2.35484700	0.18620100
C	4.43309400	1.49146500	0.60753100	C	4.38898100	1.37828900	0.61305400
C	6.48007600	1.84722800	-0.59818900	C	6.51833700	1.92234600	-0.38401800
C	4.72317500	0.13807400	0.68661600	C	4.69809900	0.03447300	0.51510400
H	3.52692400	1.86952400	1.06465500	H	3.45218100	1.68159700	1.06367100
C	6.76810300	0.49720300	-0.50834000	C	6.82405100	0.58182200	-0.47323900
H	7.16571800	2.50400400	-1.11882700	H	7.22099400	2.65440300	-0.76302800
C	5.90082100	-0.37648300	0.14687000	C	5.92446000	-0.39218800	-0.00742100
H	4.03808800	-0.52707100	1.20032500	H	3.99269300	-0.70260200	0.88185600
H	7.67842700	0.10995600	-0.95196100	H	7.76563500	0.27268100	-0.91154600
C	6.16062000	4.66164600	-0.08787500	C	6.12968400	4.60952600	0.52675300
C	7.21601900	4.53345600	0.81725600	C	7.06685700	4.35636700	1.53027000
C	6.17661900	5.70894900	-1.01038100	C	6.25651800	5.75198500	-0.26458400
C	8.27318200	5.43202400	0.78787300	C	8.12207300	5.23515900	1.72996100
H	7.20285100	3.72618700	1.54032000	H	6.95808400	3.47389000	2.14958200
C	7.22890800	6.61352400	-1.02225000	C	7.31084000	6.62823900	-0.05348700
H	5.35863800	5.80779600	-1.71457100	H	5.52708200	5.94038000	-1.04350000
C	8.28438600	6.47821000	-0.12772400	C	8.24866000	6.37399400	0.94253600
H	9.08636500	5.32036400	1.49660900	H	8.84241700	5.03387700	2.51504300
H	7.22823300	7.42224700	-1.74473900	H	7.40580800	7.50919300	-0.67843400
H	9.10849000	7.18219800	-0.14336500	H	9.07177000	7.06049900	1.10452300
C	-6.16001100	4.66239700	0.08762100	C	-6.13123700	4.60762500	-0.52678400
C	-7.21540600	4.53429500	-0.81752400	C	-7.06830100	4.35418800	-1.53033400
C	-6.17590900	5.70972800	1.01009800	C	-6.25843200	5.75003400	0.26456700
C	-8.27247400	5.43297900	-0.78818500	C	-8.12367600	5.23266400	-1.73004300
H	-7.20231200	3.72700300	-1.54056400	H	-6.95922800	3.47175600	-2.14965700
C	-7.22810100	6.61441700	1.02192400	C	-7.31302300	6.62596000	0.05346100
H	-5.35792900	5.80850300	1.71429800	H	-5.52906900	5.93863600	1.04350000
C	-8.28357800	6.47919000	0.12738300	C	-8.25073400	6.37144300	-0.94259700
H	-9.08565800	5.32138700	-1.49693000	H	-8.84403100	5.03118200	-2.51515500
H	-7.22735200	7.42316000	1.74439000	H	-7.40828500	7.50687200	0.67842300
H	-9.10760800	7.18326700	0.14299200	H	-9.07404800	7.05770000	-1.10459800
C	-5.28237500	-2.70470800	0.17126600	C	-5.27258500	-2.68320100	0.47871700
C	-4.85302400	-3.73693000	-0.66663900	C	-4.90854000	-3.75623400	-0.34082100
C	-4.75327800	-2.61883900	1.46098700	C	-4.64184500	-2.52821400	1.71593800
C	-3.92368700	-4.66144200	-0.22123900	C	-3.93560000	-4.65015000	0.07099900
H	-5.25697100	-3.81319800	-1.66941500	H	-5.39534800	-3.88279900	-1.30078300
C	-3.81134600	-3.53572100	1.89967800	C	-3.66545900	-3.42115800	2.12417600
H	-5.07939200	-1.81902000	2.11582400	H	-4.92083900	-1.69589100	2.35189700
C	-3.38743900	-4.56848100	1.06318800	C	-3.29914000	-4.49142900	1.30266400
H	-3.59135300	-5.45802100	-0.87710300	H	-3.64801100	-5.47604000	-0.56961300
H	-3.40141100	-3.45815200	2.90028500	H	-3.17885800	-3.29552400	3.08468300
C	5.28197800	-2.70535700	-0.17125500	C	5.27350500	-2.68159400	-0.47880100
C	4.85263900	-3.73765400	0.66656900	C	4.90990000	-3.75474000	0.34077300
C	4.75273200	-2.61929000	-1.46090700	C	4.64263100	-2.52685400	-1.71598100
C	3.92317400	-4.66203300	0.22116000	C	3.93724700	-4.64901000	-0.07096300
H	5.25668300	-3.81408500	1.66929400	H	5.39682100	-3.88111900	1.30070200
C	3.81068000	-3.53604600	-1.89960000	C	3.66652100	-3.42014500	-2.12413200
H	5.07883200	-1.81942200	-2.11569000	H	4.92129100	-1.69444800	-2.35197700
C	3.38678300	-4.56887000	-1.06318800	C	3.30064000	-4.49052500	-1.30258000
H	3.59084700	-5.45866100	0.87697000	H	3.64999500	-5.47499300	0.56967900
H	3.40063700	-3.45832600	-2.90015100	H	3.17980300	-3.29469500	-3.08460400
C	-2.57502500	-6.25067300	2.64345100	C	-2.32378800	-6.09139400	2.87921700
C	-1.48181600	-6.67580400	3.40827200	C	-1.15901900	-6.49187700	3.54573300
C	-3.86378900	-6.59634100	3.06911400	C	-3.56347500	-6.41285400	3.44688900
C	-1.67865700	-7.43134300	4.55479300	C	-1.23981200	-7.19984900	4.73571100
H	-0.47736200	-6.40702900	3.10523000	H	-0.19022600	-6.24079400	3.13188900
C	-4.04671000	-7.33952200	4.22541800	C	-3.62888300	-7.10854700	4.64447600
H	-4.72121800	-6.28244600	2.48624100	H	-4.47513200	-6.11861300	2.94123700
C	-2.95904100	-7.76828600	4.97723900	C	-2.47118000	-7.51283700	5.29902900
H	-0.81536800	-7.74736700	5.13067900	H	-0.32306000	-7.49765200	5.23337400
H	-5.05514300	-7.59464000	4.53313500	H	-4.60118300	-7.34586800	5.06286000
H	-3.10684900	-8.35408900	5.87696800	H	-2.52761900	-8.06162900	6.23183900
C	-1.18831100	-5.51307400	0.75105900	C	-1.11815500	-5.40712300	0.84799400

C	-0.59020100	-6.71540700	0.36335600	C	-0.55498500	-6.61039300	0.41354700
C	0.58917000	-6.71547600	-0.36355100	C	0.55715900	-6.61026100	-0.41323100
C	1.18753200	-5.51320500	-0.75107300	C	1.11994200	-5.40686100	-0.84781600
C	0.58092300	-4.31405700	-0.37506400	C	0.54844100	-4.20661700	-0.42179500
C	-0.58145700	-4.31399600	0.37524100	C	-0.54704100	-4.20674400	0.42183400
H	-1.04851100	-7.65580100	0.64710700	H	-0.98788200	-7.54992700	0.73747900
H	1.04728200	-7.65592300	-0.64744200	H	0.99035800	-7.54969300	-0.73705600
H	1.03516400	-3.37372400	-0.66445900	H	0.97868500	-3.26710100	-0.74825900
H	-1.03551200	-3.37361500	0.66477800	H	-0.97758800	-3.26733000	0.74819000
C	7.39836700	-2.14421200	0.92466400	C	7.50618200	-2.21677500	0.38211100
C	8.00476500	-3.37065100	0.62407600	C	8.07277500	-3.36874000	-0.17635400
C	8.00255600	-1.31614600	1.87829000	C	8.21148300	-1.53632000	1.38193700
C	9.17097500	-3.75478900	1.26820900	C	9.30626400	-3.82625500	0.26105400
H	7.56032800	-4.01926900	-0.12104300	H	7.54275200	-3.89907800	-0.95828300
C	9.17733300	-1.70619300	2.50453600	C	9.45154600	-1.99584200	1.79963400
H	7.54539100	-0.36704100	2.12952100	H	7.77957200	-0.65153600	1.83316300
C	9.77098100	-2.92776100	2.21093600	C	10.00888000	-3.14352600	1.24787400
H	9.62147400	-4.70903600	1.01680400	H	9.72660800	-4.72067200	-0.18590100
H	9.62484700	-1.04726400	3.24095200	H	9.97989900	-1.45495200	2.57737000
H	10.68586300	-3.23003000	2.70711800	H	10.97599000	-3.50098100	1.58193100
C	2.57426000	-6.25105900	-2.64338000	C	2.32579000	-6.09094800	-2.87898000
C	3.86302700	-6.59687500	-3.06890600	C	3.56557600	-6.41203000	-3.44664700
C	1.48107500	-6.67615600	-3.40825700	C	1.16114700	-6.49189500	-3.54543800
C	4.04597800	-7.34017300	-4.22513300	C	3.63120300	-7.10780800	-4.64417300
H	4.72043500	-6.28300200	-2.48599200	H	4.47714300	-6.11742600	-2.94104200
C	1.67794300	-7.43181000	-4.55469500	C	1.24216200	-7.19994500	-4.73535400
H	0.47662000	-6.40726200	-3.10532300	H	0.19227400	-6.24111000	-3.13159800
C	2.95833300	-7.76890500	-4.97700500	C	2.47362700	-7.51255800	-5.29866800
H	5.05441300	-7.59540400	-4.53274600	H	4.60357700	-7.34483000	-5.06255400
H	0.81467500	-7.74780500	-5.13062800	H	0.32550400	-7.49811000	-5.23297200
H	3.10616300	-8.35479800	-5.87667200	H	2.53023800	-8.06141300	-6.23143000
C	-7.39870600	-2.14333900	-0.92461500	C	-7.50537100	-2.21919200	-0.38233400
C	-8.00287500	-1.31522400	-1.87821400	C	-8.21088800	-1.53895500	-1.38215900
C	-8.00515900	-3.36975900	-0.62405000	C	-8.07155500	-3.37138900	0.17606900
C	-9.17768300	-1.70519900	-2.50444600	C	-9.45076300	-1.99892500	-1.79992000
H	-7.54566800	-0.36613800	-2.12944000	H	-7.77928500	-0.65399700	-1.83333600
C	-9.17140200	-3.75382100	-1.26816800	C	-9.30485200	-3.82935000	-0.26140900
H	-7.56073800	-4.01842300	0.12103700	H	-7.54136300	-3.90155500	0.95800000
C	-9.77138900	-2.92674200	-2.21086400	C	-10.00768500	-3.14684400	-1.24823100
H	-9.62517400	-1.04622900	-3.24084100	H	-9.97929000	-1.45820400	-2.57765500
H	-9.62194100	-4.70805300	-1.01677800	H	-9.72487900	-4.72394400	0.18549100
H	-10.68629500	-3.22895500	-2.70703500	H	-10.97464600	-3.50464800	-1.58234200
C	0.00048600	7.61783600	0.00015100	C	-0.00126800	7.57115800	0.00005800
C	0.88572500	8.32653300	-0.81610800	C	1.00845800	8.27632100	-0.66142400
C	-0.88466800	8.32650600	0.81652700	C	-1.01127800	8.27593700	0.66151600
C	0.88823600	9.71388900	-0.80620300	C	1.00590400	9.66291200	-0.65384500
H	1.57067100	7.78325600	-1.45645100	H	1.78227400	7.72945700	-1.18623700
C	-0.88700100	9.71386300	0.80685800	C	-1.00926600	9.66253100	0.65391100
H	-1.56968500	7.78320700	1.45677500	H	-1.78488100	7.72877600	1.18633100
C	0.00066300	10.41691000	0.00038800	C	-0.00182000	10.36385900	0.00002700
H	1.58179400	10.24810100	-1.44637800	H	1.79281000	10.19880600	-1.17282000
H	-1.58049200	10.24805600	1.44712100	H	-1.79638200	10.19812600	1.17287500
H	0.00073200	11.50085400	0.00048300	H	-0.00203400	11.44783500	0.00001300
N	0.00040400	6.20431300	0.00003500	N	-0.00102700	6.15733300	0.00003400
N	5.07218000	3.75457200	-0.06633400	N	5.04337200	3.72076700	0.32213200
N	6.22022300	-1.75123900	0.27514900	N	6.25459300	-1.75774400	-0.05824900
N	2.39540700	-5.49165000	-1.48080500	N	2.26442700	-5.38153300	-1.67469000
N	-2.39619500	-5.49139400	1.48079100	N	-2.26264700	-5.38206600	1.67486300
N	-6.22051600	-1.75046800	-0.27513400	N	-6.25397000	-1.75970800	0.05808300
N	-5.07167600	3.75519400	0.06613000	N	-5.04464900	3.71920400	-0.32216200

Structure S27. The coordinates of both the optimized structures of B4L$_2$ at S$_0$ and S$_1$ in toluene.

S$_0$-optimized structure				S$_1$-optimized structure			
B	-4.48650300	-5.33460700	0.83549800	B	6.67674500	2.50442900	0.58178500
C	-5.21352600	-3.98455000	0.50455000	C	6.58216000	0.96662000	0.28353700
C	-4.89114600	-3.25678200	-0.65100100	C	5.91672500	0.48300700	-0.85402600
C	-6.07584900	-3.36573200	1.42207500	C	6.99433100	0.01566200	1.22989200
C	-5.35250300	-1.96794800	-0.85162800	C	5.63250300	-0.86149500	-1.01403200
H	-4.22840600	-3.69621400	-1.38937700	H	5.57906400	1.18342000	-1.61112900
C	-6.50008900	-2.05346800	1.25260000	C	6.65407700	-1.32465000	1.10877600
H	-6.37610100	-3.90305600	2.31615000	H	7.54218000	0.34151900	2.10851500
C	-6.11887500	-1.32077300	0.12522500	C	5.94034100	-1.78336300	-0.00401600
H	-5.04099600	-1.41887500	-1.73346300	H	5.07894300	-1.19358000	-1.88564500
H	-7.12121800	-1.58678200	2.01007600	H	6.93546800	-2.02449700	1.88863400
C	-6.34436700	0.13434200	-0.00180500	C	5.37122100	-3.14051200	-0.07423300
C	-6.12610000	0.97314900	1.09805000	C	4.73023300	-3.67883800	1.05176400
C	-6.59712200	0.73073900	-1.23961600	C	5.29770700	-3.85698200	-1.27444600
C	-6.00267900	2.34301500	0.92886700	C	3.93294600	-4.80342000	0.93981600
H	-5.94620800	0.53595700	2.07449600	H	4.78215400	-3.14871000	1.99684900
C	-6.52126300	2.10858300	-1.39149400	C	4.54385200	-5.01771600	-1.36276200
H	-6.82328600	0.10717400	-2.09816600	H	5.82035700	-3.48664200	-2.15029500
C	-6.13929500	2.94401900	-0.33138300	C	3.77063600	-5.48029900	-0.28233400
H	-5.72923300	2.95210200	1.78488800	H	3.37465500	-5.13726200	1.80892600
H	-6.68911100	2.53813500	-2.37407700	H	4.49039300	-5.53897000	-2.31326600
C	-2.93623800	-5.36638900	0.60587300	C	5.34836800	3.32142500	0.43282000
C	-2.20511600	-6.51308900	0.25280800	C	5.27367800	4.67846500	0.07528000
C	-2.20194200	-4.17882500	0.75672700	C	4.12833000	2.67229900	0.68312000
C	-0.83237000	-6.47320000	0.04686000	C	4.05858200	5.34068100	-0.03928800
H	-2.72529200	-7.45632100	0.12075300	H	6.18752300	5.22510900	-0.13396200
C	-0.83189700	-4.13640500	0.57728500	C	2.91689900	3.33203400	0.59821900
H	-2.72007100	-3.26403000	1.02062200	H	4.13103500	1.62212300	0.95176000
C	-0.11658300	-5.28175400	0.21144600	C	2.85401100	4.68006300	0.22871100
H	-0.30538300	-7.38190700	-0.22453600	H	4.04398200	6.38853600	-0.32005700
H	-0.31261900	-3.19071600	0.68173800	H	1.99953500	2.78364600	0.77868300
C	1.34011900	-5.17788200	-0.01555300	C	1.53029900	5.32224600	0.09548100
C	2.09660500	-4.26844400	0.73317800	C	0.48025200	4.92960900	0.93503900
C	1.99550500	-5.90656200	-1.01324300	C	1.25028000	6.25456100	-0.90805000
C	3.42376400	-4.02949700	0.42946200	C	-0.81189300	5.36210400	0.70413500
H	1.63096700	-3.72885200	1.54996900	H	0.67837600	4.25535500	1.76059600
C	3.34336700	-5.69628900	-1.28030600	C	-0.04470000	6.72088100	-1.10509300
H	1.43477300	-6.61087000	-1.61851700	H	2.04133800	6.57575000	-1.57738100
C	4.08590200	-4.72232200	-0.59656100	C	-1.12087300	6.24840000	-0.34003000
H	3.96251800	-3.27842500	0.99729300	H	-1.60956200	4.98737900	1.33696700
H	3.81381100	-6.26394400	-2.07699300	H	-0.23205000	7.41944400	-1.91454400
B	5.54565700	-4.29993600	-0.99049700	B	-2.62159200	6.53023300	-0.70778300
B	-5.60329800	4.37441200	-0.69719900	B	2.62639600	-6.52807800	-0.52868400
C	-4.12549200	4.70776600	-0.29291000	C	1.18829100	-6.09078700	-0.12264000
C	-3.53435400	5.98052700	-0.39273300	C	0.07223700	-6.96490200	0.03865700
C	-3.27445100	3.66726500	0.11441100	C	0.90503400	-4.70643400	0.04379400
C	-2.17928900	6.18405100	-0.17083700	C	-1.19415400	-6.50681200	0.29640500
H	-4.14683000	6.83273400	-0.66641700	H	0.23259000	-8.03480600	-0.03199100
C	-1.93195000	3.86714300	0.36600100	C	-0.34703300	-4.23358500	0.31001500
H	-3.66983000	2.66508900	0.21924300	H	1.70898600	-3.99172700	-0.07769000
C	-1.33869600	5.12181100	0.18799900	C	-1.46995800	-5.10988500	0.43349600
H	-1.76558700	7.17630000	-0.31304100	H	-1.99477400	-7.22439600	0.42848200
H	-1.33777900	3.02754300	0.70395600	H	-0.48831300	-3.16372400	0.36988600
C	0.12544700	5.26287100	0.33983900	C	-2.77586900	-4.57899000	0.63856600
C	0.93831100	4.13437100	0.18909800	C	-2.95305600	-3.20303100	0.98648200
C	0.76150300	6.48020900	0.61764400	C	-3.97265600	-5.34066600	0.45175200
C	2.30827100	4.21153800	0.33838800	C	-4.19094300	-2.63179100	1.02405500
H	0.49968500	3.18044500	-0.07393100	H	-2.09552700	-2.59508900	1.23951500
C	2.14184700	6.55255200	0.75201100	C	-5.21172700	-4.75287400	0.51132000
H	0.17124900	7.38022100	0.74961500	H	-3.90236700	-6.39116200	0.19707900
C	2.96489000	5.41932900	0.62521300	C	-5.38231800	-3.35997700	0.74972000
H	2.88658100	3.30275600	0.23116400	H	-4.26973100	-1.58103100	1.27507100
H	2.59228300	7.51821500	0.95623000	H	-6.08774400	-5.36160700	0.31530700
C	5.81013000	-2.75450300	-1.01522400	C	-3.55432500	5.27132200	-0.71446000
C	6.95223100	-2.14222300	-0.47622900	C	-4.89192900	5.25847700	-0.28413000
C	4.80786600	-1.90150900	-1.50282900	C	-3.01404400	4.03785200	-1.11391100
C	7.05085600	-0.76118700	-0.35293900	C	-5.62408300	4.08109500	-0.20217000
H	7.76242400	-2.76047600	-0.10252900	H	-5.36014300	6.18705600	0.02674200
C	4.92024800	-0.52614000	-1.42672600	C	-3.74628700	2.86853100	-1.06696200
H	3.90486200	-2.32894300	-1.92514100	H	-1.98492100	3.99340600	-1.45196100
C	6.02462600	0.07206800	-0.80878500	C	-5.06081000	2.85608300	-0.58214800
H	7.93043800	-0.32498200	0.10896500	H	-6.64704300	4.11241300	0.15768900
H	4.10345200	0.09505900	-1.77633000	H	-3.26904300	1.93671200	-1.34610000
C	5.97490100	1.52610500	-0.54737600	C	-5.73597100	1.55669100	-0.40861100
C	5.36118800	2.39414000	-1.46040800	C	-5.40958800	0.48075900	-1.24798900
C	6.36734400	2.04854200	0.68717900	C	-6.60166900	1.30233500	0.66141300
C	5.00343500	3.67655700	-1.08508600	C	-5.80617700	-0.80484400	-0.93964300
H	5.10948500	2.03567300	-2.45274100	H	-4.80487700	0.65390800	-2.13109000
C	6.04182200	3.35290900	1.04175300	C	-7.03432500	0.01071100	0.93907200
H	6.85365300	1.40382000	1.41158000	H	-6.87951000	2.11019300	1.33005200
C	5.27828900	4.17283400	0.19925200	C	-6.58707700	-1.09230600	0.19390400
H	4.44716000	4.29049200	-1.78638200	H	-5.47276100	-1.61959700	-1.57435700
H	6.30744200	3.70753300	2.03267200	H	-7.66444900	-0.15438500	1.80748800
B	4.52263100	5.40523100	0.81317400	B	-6.73062100	-2.57700900	0.69141600
C	-5.26615200	-6.54465600	1.43467900	C	8.00498300	3.14420500	1.08763000
C	-4.64069400	-7.50491200	2.24699500	C	8.02222000	4.31330700	1.86636900
C	-6.63914400	-6.70639700	1.18531400	C	9.24296900	2.55317800	0.78430000
C	-5.34844700	-8.57046300	2.78558600	C	9.21249200	4.86461100	2.31966700
H	-3.58480300	-7.40162300	2.47321700	H	7.08355100	4.78559300	2.13545400
C	-7.34908400	-7.78101600	1.70218900	C	10.43848000	3.10952800	1.21667100
H	-7.15234500	-5.97866300	0.56530100	H	9.26264700	1.64564900	0.18982300
C	-6.70364800	-8.71318000	2.50759200	C	10.42360000	4.26621100	1.98875600
H	-4.84565600	-9.29147900	3.42100900	H	9.19872400	5.76094900	2.93024300
H	-8.40588700	-7.89053500	1.48408500	H	11.38178300	2.64058400	0.95853200

H	-7.25742500	-9.54926000	2.92123800	H	11.35565900	4.69915600	2.33604100
C	-6.47416400	5.31723900	-1.58826800	C	2.93156300	-7.85625600	-1.28697100
C	-7.86885000	5.33318000	-1.42326400	C	4.21048100	-8.43973900	-1.19877100
C	-5.93447300	6.14918600	-2.58245100	C	1.99061300	-8.51409100	-2.10220600
C	-8.68150200	6.16365000	-2.18240200	C	4.52317200	-9.62044300	-1.85592000
H	-8.31912600	4.68438200	-0.67885800	H	4.96681700	-7.95599700	-0.58919200
C	-6.74374100	6.96090700	-3.36609400	C	2.30051300	-9.68721200	-2.77402400
H	-4.86501200	6.14034200	-2.76256900	H	1.00377500	-8.08217400	-2.23025200
C	-8.11854800	6.97790800	-3.15895900	C	3.56738100	-10.24947300	-2.64746700
H	-9.75427700	6.16844100	-2.02260100	H	5.51378600	-10.05116700	-1.75538400
H	-6.30322200	7.58180100	-4.13871700	H	1.55582500	-10.16464100	-3.40217900
H	-8.75135800	7.61853600	-3.76383200	H	3.80973000	-11.16982900	-3.16791300
C	5.28356900	6.45574300	1.67889900	C	-8.08998800	-3.17316400	1.15016000
C	6.63045300	6.74757800	1.40736500	C	-9.30431500	-2.59106500	0.73767500
C	4.68163900	7.13062200	2.75346500	C	-8.17688400	-4.29079900	2.00272300
C	7.33397000	7.68630300	2.14898600	C	-10.53030000	-3.10367400	1.13395200
H	7.12764600	6.23117500	0.59262100	H	-9.27639000	-1.72699500	0.08193500
C	5.38583800	8.05246500	3.51577900	C	-9.39910200	-4.80300300	2.41013300
H	3.65051200	6.91134800	3.00871300	H	-7.26485700	-4.75281100	2.36597500
C	6.71152500	8.33840100	3.20790000	C	-10.58165900	-4.21247100	1.97340400
H	8.36913700	7.90422200	1.90955800	H	-11.44903700	-2.64031700	0.79032700
H	4.90260700	8.54984300	4.34976800	H	-9.43421100	-5.66149800	3.07246500
H	7.26110100	9.06491700	3.79691400	H	-11.53904200	-4.61323700	2.28866700
C	6.64946100	-5.34240000	-1.33297700	C	-3.12643300	7.95577700	-1.07768800
C	6.55807300	-6.67298900	-0.89180200	C	-2.43655800	9.10125000	-0.64683100
C	7.77585100	-4.98967400	-2.09477600	C	-4.27949100	8.15101400	-1.85613200
C	7.54489500	-7.60318800	-1.18609200	C	-2.88134000	10.37724600	-0.96255900
H	5.70251600	-6.97488600	-0.29683800	H	-1.54235900	8.98324800	-0.04369900
C	8.75803400	-5.91837200	-2.40847600	C	-4.72056400	9.42333700	-2.19135300
H	7.87185800	-3.97129800	-2.45638400	H	-4.82783500	7.28688600	-2.21602400
C	8.64492300	-7.22648100	-1.94939800	C	-4.02365000	10.53901500	-1.73938400
H	7.45807700	-8.62199200	-0.82438200	H	-2.33798400	11.24648900	-0.60820600
H	9.61325600	-5.62518200	-3.00761500	H	-5.60739000	9.54815500	-2.80316100
H	9.41479600	-7.95295000	-2.18676300	H	-4.36984500	11.53501500	-1.99430000

Structure S28. The coordinates of both the optimized structures of B3N1L$_2$ at S$_0$ and S$_1$ in toluene.

S_0-optimized structure				S_1-optimized structure			
B	3.62452200	6.01003700	0.55785800	B	3.32137900	6.14582500	0.58900200
C	4.46223200	4.72219000	0.23502100	C	4.27742200	4.93271700	0.29504500
C	4.18204100	3.93572700	-0.89255200	C	4.21168200	4.20992000	-0.90624400
C	5.39005400	4.20867700	1.15253300	C	5.09520700	4.40898800	1.30785600
C	4.75404100	2.68743600	-1.06945100	C	4.87536800	3.00602100	-1.06847600
H	3.46802000	4.29355900	-1.62735600	H	3.59066900	4.57857400	-1.71630300
C	5.92055700	2.93206700	1.01235300	C	5.70290300	3.16797400	1.18126000
H	5.65934400	4.79688600	2.02424400	H	5.20552300	4.95563100	2.23904700
C	5.58825000	2.13915100	-0.08875800	C	5.57888800	2.43131700	-0.00164300
H	4.48291700	2.08892300	-1.93271000	H	4.76590300	2.45367300	-1.99582400
H	6.59005800	2.53974400	1.77089600	H	6.27319400	2.76005100	2.00943000
C	5.95769500	0.70999000	-0.17421300	C	6.00689000	1.02615200	-0.09616200
C	5.76064000	-0.11881600	0.93421300	C	5.69598800	0.14389200	0.94942100
C	6.35821000	0.11794200	-1.37639800	C	6.58291000	0.49424100	-1.26027600
C	5.86617100	-1.49567800	0.82198100	C	5.82597500	-1.22023200	0.79014100
H	5.45316300	0.31698900	1.87831700	H	5.26800100	0.53327300	1.86577800
C	6.47384500	-1.25979600	-1.49258800	C	6.76436800	-0.86802600	-1.41416100
H	6.54825600	0.74263700	-2.24252300	H	6.86360600	1.16082800	-2.06783000
C	6.17698100	-2.07959600	-0.40418500	C	6.32708700	-1.73925400	-0.40902900
H	5.64842400	-2.13369100	1.67082700	H	5.51051300	-1.89867700	1.57384800
H	6.74209500	-1.70875300	-2.44228000	H	7.16848800	-1.26907800	-2.33621100
C	2.06842800	5.86564700	0.44841100	C	1.79481300	5.89357400	0.36586000
C	1.18544800	6.90392000	0.10685300	C	0.84118600	6.88951200	0.08715400
C	1.49045400	4.61297200	0.71033700	C	1.30525700	4.58233800	0.49435600
C	-0.18521100	6.69992200	0.01452800	C	-0.50879600	6.59670500	-0.04821400
H	1.58191100	7.89083000	-0.10896800	H	1.16589500	7.91758900	-0.03622000
C	0.12510000	4.40913800	0.64686000	C	-0.03971100	4.28704800	0.38530600
H	2.12957600	3.77650300	0.96864300	H	1.99880900	3.77403800	0.69567500
C	-0.74307200	5.44616300	0.28921800	C	-0.98028000	5.28812200	0.11564100
H	-0.83297300	7.52725300	-0.25569800	H	-1.21182100	7.39658800	-0.25575500
H	-0.27317500	3.41841100	0.83258700	H	-0.36680700	3.25785600	0.47769400
C	-2.18697600	5.15540300	0.17075000	C	-2.40348400	4.91579700	-0.00493400
C	-2.77460800	4.20518700	1.01515300	C	-2.90892500	3.86813700	0.77519400
C	-2.98355600	5.72377600	-0.82743700	C	-3.26668900	5.51586100	-0.92792600
C	-4.06258000	3.75765400	0.79042700	C	-4.18220500	3.37596700	0.56403200
H	-2.20156300	3.79147200	1.83729100	H	-2.28614300	3.42256300	1.54266500
C	-4.29576300	5.30358400	-1.01634600	C	-4.56172400	5.04150000	-1.10480100
H	-2.55644000	6.45985100	-1.50034000	H	-2.90487200	6.32650900	-1.55193300
C	-4.85373400	4.27122500	-0.24925200	C	-5.04190600	3.92706300	-0.40048100
H	-4.46080500	2.97173100	1.42358400	H	-4.51810700	2.52809700	1.15125700
H	-4.87532300	5.74282900	-1.82229400	H	-5.19318100	5.51176500	-1.85224600
B	-6.21022100	3.56840400	-0.61317300	B	-6.39845000	3.19870900	-0.71781300
C	4.75951200	-4.01333300	-0.42630900	C	4.97495000	-3.69559900	-0.49385100
C	4.50660800	-5.27307600	0.12863500	C	4.77490800	-5.00391300	0.00128300
C	3.66754500	-3.23375000	-0.82015800	C	3.84284100	-2.91690000	-0.81579600
C	3.20329900	-5.71702400	0.30668700	C	3.50083700	-5.48476300	0.19531400
H	5.33442900	-5.89483900	0.44869900	H	5.63012100	-5.60894900	0.27809300
C	2.37576300	-3.67657700	-0.61952500	C	2.58255200	-3.40653000	-0.62018600
H	3.83589100	-2.26845000	-1.28057600	H	3.97944800	-1.93955500	-1.26096600
C	2.10768600	-4.91974400	-0.03813700	C	2.35121000	-4.69787000	-0.07801100
H	3.03804100	-6.68221000	0.77389700	H	3.37856600	-6.47078300	0.62706000
H	1.55246200	-3.05119900	-0.94550900	H	1.74258900	-2.80324100	-0.93566100
C	0.70443100	-5.30391700	0.20870100	C	0.99841800	-5.13598200	0.17254200
C	-0.20005200	-4.31821200	0.61971900	C	-0.02988400	-4.17568500	0.33040200
C	0.21348100	-6.59924900	0.01135700	C	0.61054100	-6.49522500	0.24914700
C	-1.54575600	-4.59860500	0.75239300	C	-1.33764500	-4.54777900	0.49811800
H	0.16168800	-3.31833300	0.83154200	H	0.21774700	-3.12111600	0.36398100
C	-1.13691400	-6.88193800	0.18152000	C	-0.71126900	-6.85632400	0.41273100
H	0.88823800	-7.38229600	-0.31888000	H	1.35628700	-7.27297900	0.11899800
C	-2.06384900	-5.88504400	0.52730500	C	-1.75914700	-5.90316600	0.51931900
H	-2.21726500	-3.79888100	1.04365800	H	-2.07810800	-3.76932100	0.63539300
H	-1.48599400	-7.89260100	-0.00554800	H	-0.96069000	-7.91241200	0.41627400
C	-6.15339700	2.00185000	-0.63946700	C	-6.30960600	1.63973100	-0.78512500
C	-7.17080900	1.15247700	-0.17610000	C	-7.27358900	0.75595000	-0.26911600
C	-4.97428800	1.38413600	-1.08594100	C	-5.14146000	1.05195200	-1.30149900
C	-6.99783000	-0.22483900	-0.10456600	C	-7.05083200	-0.61306000	-0.19799500
H	-8.10596600	1.58042500	0.17110200	H	-8.20149100	1.15484000	0.12923300
C	-4.80943900	0.01317000	-1.05210000	C	-4.93091400	-0.31098900	-1.27124300
H	-4.15744200	1.99753800	-1.44935300	H	-4.36581800	1.68838300	-1.71331100
C	-5.80575800	-0.82002000	-0.53085700	C	-5.86148200	-1.17800100	-0.67966200
H	-7.79782800	-0.84557500	0.28532600	H	-7.80740900	-1.25399500	0.24262900
H	-3.86613100	-0.41545600	-1.37013100	H	-3.99156900	-0.70695600	-1.63862600
C	-5.50294600	-2.25721700	-0.36727000	C	-5.48638000	-2.58901700	-0.49086100
C	-4.65960200	-2.90281700	-1.28056800	C	-4.58785200	-3.20576000	-1.37513900
C	-5.91215400	-2.97247500	0.76138000	C	-5.86001400	-3.31343900	0.64928100
C	-4.13254000	-4.14868200	-0.99798500	C	-3.97780200	-4.40022900	-1.05079600
H	-4.37798200	-2.39861300	-2.19827700	H	-4.33834400	-2.71827300	-2.31155100
C	-5.41854600	-4.24849400	1.01305300	C	-5.26616500	-4.53262300	0.94966400
H	-6.56579300	-2.50029900	1.48719600	H	-6.55672000	-2.87513900	1.35661000
C	-4.46374200	-4.84219100	0.17666500	C	-4.23994200	-5.07706800	0.15706500
H	-3.41620500	-4.58225400	-1.68824500	H	-3.23723200	-4.80782400	-1.73163600
H	-5.72300100	-4.75805700	1.92201300	H	-5.53751700	-5.02930400	1.87638200
B	-3.61535500	-6.08702900	0.62298800	B	-3.27931600	-6.21532500	0.65899000
C	4.31573000	7.31953800	1.04389100	C	3.88599300	7.47203300	1.18256300
C	3.64951300	8.26266800	1.84378700	C	3.11020100	8.32758000	1.98292500
C	5.65016900	7.59256800	0.70018700	C	5.21869700	7.84812500	0.94344100
C	4.28175700	9.41879500	2.27949900	C	3.63457700	9.49780600	2.51366700
H	2.62396200	8.07460800	2.14294800	H	2.08419500	8.05767600	2.20839600
C	6.28262200	8.75639400	1.11401200	C	5.74433200	9.02669000	1.45343800
H	6.19377100	6.87973700	0.08883100	H	5.84749700	7.20423300	0.33703800
C	5.59835700	9.67015300	1.90842200	C	4.95172500	9.85312100	2.24296500
H	3.75019200	10.12567000	2.90732700	H	3.01801800	10.13438900	3.13903700
H	7.30976700	8.95023600	0.82461000	H	6.77264000	9.29999100	1.24268400
H	6.09257300	10.57652000	2.24171400	H	5.36159000	10.77085100	2.65106600

C	7.15593400	-4.24603400	-0.99173900	C	7.34130800	-3.84790200	-1.10643900
C	8.45354900	-3.85298100	-0.64485500	C	8.62920900	-3.46770600	-0.70354100
C	6.98877500	-5.38327600	-1.78960400	C	7.17452600	-4.93803300	-1.97259100
C	9.54900700	-4.58048200	-1.08490700	C	9.72747900	-4.17382600	-1.16001100
H	8.59752800	-2.97578400	-0.02534900	H	8.75284700	-2.63766300	-0.01902200
C	8.09232700	-6.11149300	-2.20980800	C	8.28368400	-5.63804600	-2.41364400
H	5.99385200	-5.69124100	-2.08667600	H	6.18302500	-5.20691300	-2.31477400
C	9.37990300	-5.71832400	-1.86497400	C	9.56245800	-5.26284600	-2.01180100
H	10.54512200	-4.25691100	-0.80298100	H	10.71984200	-3.88074900	-0.83817900
H	7.93907100	-6.98948000	-2.82802300	H	8.14977200	-6.47276800	-3.09149300
H	10.23827600	-6.28836700	-2.20081500	H	10.42650200	-5.81406500	-2.36307600
C	-4.27564000	-7.38353300	1.17517100	C	-3.83212400	-7.50642200	1.33562100
C	-5.60976600	-7.69688200	0.86678100	C	-5.14397500	-7.95245500	1.07826900
C	-3.57616800	-8.27757600	2.00254800	C	-3.07884300	-8.27511500	2.24533600
C	-6.21217500	-8.85212600	1.34431900	C	-5.66380400	-9.09315600	1.67213400
H	-6.17652300	-7.02308100	0.23256800	H	-5.76058200	-7.38925500	0.38486400
C	-4.17677600	-9.42613100	2.49838400	C	-3.59271500	-9.41397700	2.84760400
H	-2.54922700	-8.05591600	2.27275100	H	-2.07396600	-7.95463100	2.50008300
C	-5.49526100	-9.71713500	2.16430800	C	-4.88908300	-9.83268300	2.56111400
H	-7.24039000	-9.07816300	1.08349400	H	-6.67614600	-9.40981700	1.44208300
H	-3.61993700	-10.09591500	3.14468400	H	-2.98485700	-9.97602200	3.54961200
H	-5.96542600	-10.61727300	2.54583100	H	-5.29289700	-10.72410300	3.02932200
C	-7.49589000	4.37424900	-0.95846000	C	-7.72345900	3.98349900	-0.95498100
C	-7.65454900	5.70156100	-0.52663900	C	-7.89617900	5.28884000	-0.46501000
C	-8.53753400	3.81118800	-1.71428100	C	-8.79067700	3.41906300	-1.67355800
C	-8.80001300	6.42741600	-0.82103600	C	-9.07540600	5.99131200	-0.66989700
H	-6.86710400	6.16363500	0.05963800	H	-7.09187800	5.75227900	0.09693700
C	-9.67838800	4.53628000	-2.02791300	C	-9.96716800	4.12057600	-1.89647300
H	-8.44015600	2.79196700	-2.07295500	H	-8.68518800	2.41654800	-2.07469000
C	-9.81247000	5.84500400	-1.57621700	C	-10.11248600	5.40801600	-1.39029000
H	-8.90456000	7.44716600	-0.46676500	H	-9.18775200	6.99403400	-0.27164100
H	-10.46460600	4.08375700	-2.62239100	H	-10.77250000	3.66571400	-2.46315600
H	-10.70611500	6.41199600	-1.81450100	H	-11.03322300	5.95672400	-1.55797200
N	6.06062600	-3.48902900	-0.55200500	N	6.23555200	-3.12862600	-0.64657800

Structure S29. The coordinates of both the optimized structures of B2N2L$_2$ at S$_0$ and S$_1$ in toluene.

S_0-optimized structure				S_1-optimized structure			
B	4.93769200	5.04148400	0.55282800	B	5.60519700	4.43638300	0.32080300
C	5.46236000	3.59621600	0.23640700	C	6.01528400	2.92240600	0.08414900
C	5.00084600	2.88816800	-0.88352500	C	5.62182400	2.23784800	-1.07536500
C	6.25421100	2.88841700	1.15204500	C	6.61015700	2.15052700	1.09268400
C	5.26620900	1.54038400	-1.05247500	C	5.72022100	0.85992300	-1.18339000
H	4.38318300	3.39597000	-1.61727800	H	5.16464100	2.79419400	-1.88745000
C	6.47494700	1.52276100	1.01911900	C	6.67224600	0.76510000	1.01447800
H	6.65818700	3.40396700	2.01773600	H	6.97258100	2.64088100	1.99116000
C	5.95794800	0.81988200	-0.07203300	C	6.18936200	0.09259400	-0.11220300
H	4.85553700	1.01545500	-1.90835800	H	5.33825000	0.36485500	-2.07043900
H	7.04211800	0.99241900	1.77716100	H	7.07846900	0.19704800	1.84574900
C	5.97925600	-0.65639200	-0.14927100	C	5.98128500	-1.36904000	-0.13196900
C	5.62290300	-1.41305600	0.97117700	C	5.44266800	-2.00229800	0.99424600
C	6.18996400	-1.32961800	-1.35708200	C	6.15173100	-2.13073000	-1.29540600
C	5.38841400	-2.77469400	0.86678300	C	4.98197500	-3.30595900	0.92836800
H	5.45819300	-0.91577200	1.92062600	H	5.30920600	-1.43737200	1.90942200
C	5.96172700	-2.69331800	-1.46666100	C	5.70542100	-3.44145300	-1.36811100
H	6.49969500	-0.77047800	-2.23340000	H	6.60461700	-1.67541300	-2.16874200
C	5.50593400	-3.41630800	-0.36448300	C	5.06991200	-4.01201400	-0.26856100
H	5.04847900	-3.34019200	1.72681300	H	4.50019200	-3.76345000	1.78506600
H	6.08231400	-3.19306700	-2.42114500	H	5.79506700	-4.00341200	-2.29079200
C	3.38807400	5.24633300	0.44243600	C	4.10000900	4.74301300	0.24913700
C	2.75704200	6.44928900	0.08360100	C	3.51780700	6.02724100	0.07748100
C	2.54679700	4.15551700	0.71505300	C	3.16133200	3.68603600	0.36531100
C	1.37549200	6.55051300	-0.01752600	C	2.15443500	6.23065100	0.02594700
H	3.36196100	7.32203400	-0.14047500	H	4.16873400	6.88737200	-0.04452500
C	1.17056200	4.25593600	0.64252100	C	1.80385900	3.88200200	0.33809800
H	2.98450000	3.20218800	0.98779100	H	3.52718000	2.67352900	0.49198900
C	0.55362300	5.45349300	0.26506400	C	1.23369300	5.16497300	0.16722100
H	0.92741800	7.49660700	-0.30258800	H	1.78304800	7.24146900	-0.10718500
H	0.56397300	3.37902000	0.83677600	H	1.15820300	3.01525400	0.40955400
C	-0.91798900	5.48501000	0.13465000	C	-0.20623100	5.32440900	0.11315300
C	-1.70575800	4.68919900	0.97556500	C	-1.06074900	4.30481300	0.59481600
C	-1.56377800	6.21028100	-0.87098000	C	-0.85280000	6.44822400	-0.45356000
C	-3.05866100	4.53376300	0.74143000	C	-2.42120500	4.35998200	0.42978500
H	-1.24396000	4.16294000	1.80318900	H	-0.63883300	3.45963700	1.12545500
C	-2.93499200	6.08820000	-1.06865400	C	-2.22532000	6.50789200	-0.58925400
H	-0.98133900	6.83252700	-1.54211800	H	-0.25527700	7.26377400	-0.84738900
C	-3.71126900	5.20648000	-0.30357200	C	-3.08256300	5.44729200	-0.19734800
H	-3.62388700	3.85611900	1.37266700	H	-3.01665200	3.53948100	0.81534300
H	-3.39858100	6.64210200	-1.87901300	H	-2.65591800	7.37775400	-1.07618200
B	-5.19120200	4.82187000	-0.66197000	B	-4.60032400	5.37758100	-0.45497400
C	3.65033000	-4.93935300	-0.34019400	C	3.01906100	-5.24490000	-0.29356200
C	3.10930100	-6.13668300	0.14255700	C	2.26270000	-6.38162800	0.07496200
C	2.77333800	-3.88870700	-0.62666200	C	2.32321900	-4.04070800	-0.54344400
C	1.74130800	-6.26484100	0.34302000	C	0.89665600	-6.29954500	0.20926600
H	3.76628200	-6.96419300	0.38333100	H	2.76796900	-7.31146100	0.30131800
C	1.41726900	-4.01997500	-0.40147300	C	0.96456500	-3.97438200	-0.40164000
H	3.15878300	-2.96026200	-1.02806700	H	2.86683900	-3.16869400	-0.87889500
C	0.86503300	-5.20370500	0.09660500	C	0.19686100	-5.09122500	-0.00455700
H	1.35568800	-7.19578700	0.74562500	H	0.35978300	-7.17754500	0.54829400
H	0.76390600	-3.19046800	-0.64785200	H	0.47221500	-3.04392200	-0.65174300
C	-0.58869600	-5.25871800	0.34375900	C	-1.23153000	-4.95804900	0.17189100
C	-1.22774900	-4.13955700	0.88770800	C	-1.79414700	-3.68888800	0.43051200
C	-1.38806100	-6.35263000	-0.00179600	C	-2.12938200	-6.04521000	0.08197800
C	-2.60057200	-4.08917500	1.02642800	C	-3.14421300	-3.50414900	0.54322400
H	-0.63307000	-3.29548900	1.21889000	H	-1.14839100	-2.83607800	0.59284500
C	-2.77165500	-6.30377300	0.11500400	C	-3.48987600	-5.87583000	0.19207200
H	-0.92830100	-7.23611900	-0.43236800	H	-1.75089700	-7.03486300	-0.14411600
C	-3.39927200	-5.15471900	0.60335400	C	-4.03817800	-4.58993800	0.40407500
H	-3.06750000	-3.20615000	1.44548400	H	-3.53010900	-2.52008300	0.76933000
H	-3.37139900	-7.14500000	-0.21309700	H	-4.14842700	-6.72462600	0.06043500
C	-5.48179200	3.28140500	-0.67815800	C	-5.20601300	3.92206900	-0.58432900
C	-6.64475400	2.68038300	-0.17140400	C	-6.34234500	3.46297000	0.10152700
C	-4.48052100	2.41632700	-1.14781600	C	-4.51290600	2.96156600	-1.33976000
C	-6.77217300	1.29944900	-0.07659400	C	-6.69201700	2.11823500	0.13203100
H	-7.45309500	3.30635500	0.19338400	H	-6.93836300	4.16971700	0.67101900
C	-4.61679700	1.04279600	-1.09316800	C	-4.86824000	1.62629800	-1.34005900
H	-3.56087600	2.83304800	-1.54340100	H	-3.63700800	3.26765500	-1.90130500
C	-5.75148500	0.45356700	-0.52348100	C	-5.93834500	1.16542700	-0.56336300
H	-7.67410700	0.87252600	0.34960900	H	-7.55121300	1.80372000	0.71611800
H	-3.79996600	0.41408600	-1.42870700	H	-4.25321400	0.91882400	-1.88533700
C	-5.75876600	-1.01034800	-0.32990500	C	-6.10458100	-0.28971300	-0.39077200
C	-5.12156800	-1.84754800	-1.25329900	C	-5.71720600	-1.17916200	-1.40322100
C	-6.25445700	-1.59293300	0.84122000	C	-6.48620900	-0.83671000	0.84261400
C	-4.89038300	-3.18007300	-0.96909000	C	-5.55905300	-2.52834400	-1.14758200
H	-4.78462200	-1.44276600	-2.20095200	H	-5.49488600	-0.80014600	-2.39336500
C	-6.01961400	-2.92893400	1.13698100	C	-6.35165400	-2.19195800	1.10357100
H	-6.75322800	-0.97260600	1.57802500	H	-6.80281500	-0.17806400	1.64298800
C	-5.28049900	-3.72026500	0.25722800	C	-5.82080500	-3.02932600	0.12576500
H	-4.35636200	-3.80529300	-1.67517000	H	-5.19040000	-3.19531400	-1.91878800
H	-6.34620100	-3.34141500	2.08475800	H	-6.57182400	-2.58490100	2.08958500
C	5.90427700	6.16697900	1.02933300	C	6.71164200	5.48469000	0.67764500
C	5.46470100	7.24431800	1.81628400	C	6.45652100	6.62183900	1.46514300
C	7.26653400	6.13051300	0.68815700	C	8.03770900	5.30363800	0.24514700
C	6.33894700	8.23454200	2.24208100	C	7.45660000	7.52863300	1.78827800
H	4.42257500	7.29383600	2.11318300	H	5.45487700	6.78358500	1.84872900
C	8.14288700	7.12767800	1.09247200	C	9.04153200	6.21179900	0.55183200
H	7.63755700	5.30757500	0.08608500	H	8.27942400	4.43008100	-0.35252800
C	7.67909500	8.18052100	1.87392800	C	8.75403700	7.33107600	1.32628800
H	5.97804300	9.04973300	2.85982600	H	7.22615800	8.39082800	2.40577600
H	9.18779400	7.08346400	0.80515200	H	10.05172500	6.04643100	0.19117200
H	8.36292300	8.95717300	2.19950100	H	9.53660800	8.04051400	1.57402600
C	5.88236900	-5.73213300	-1.04651800	C	5.13887500	-6.42838000	-0.65656900

C	7.24706900	-5.71173900	-0.73800200	C	6.37993200	-6.60067900	-0.03976300
C	5.40209300	-6.72429700	-1.90757600	C	4.67439500	-7.38579200	-1.56257700
C	8.10371800	-6.65873500	-1.27832000	C	7.13611600	-7.72883200	-0.31549500
H	7.63035200	-4.94981300	-0.06952800	H	6.74105100	-5.85303900	0.65592100
C	6.26665200	-7.67618700	-2.42905000	C	5.43650300	-8.51436500	-1.82292800
H	4.35252600	-6.74435000	-2.17401400	H	3.73188000	-7.23262400	-2.07460700
C	7.62192000	-7.65211500	-2.12294800	C	6.66755200	-8.69248400	-1.20188800
H	9.15774000	-6.62346400	-1.02504800	H	8.09587900	-7.85732700	0.17116800
H	5.87324200	-8.43597200	-3.09576100	H	5.07228800	-9.24928000	-2.53148000
H	8.29311100	-8.39516300	-2.53766800	H	7.26237500	-9.57286800	-1.41448900
C	-5.66758400	-5.98751500	1.12041500	C	-6.34245300	-5.31693800	0.83982800
C	-6.99945300	-6.03353000	0.69203100	C	-7.59407900	-5.33765100	0.22008500
C	-5.22849900	-6.93313300	2.05367200	C	-6.06168700	-6.21978400	1.86931600
C	-7.86392300	-6.99628700	1.19047700	C	-8.54410400	-6.26520700	0.61680300
H	-7.35106300	-5.31353100	-0.03737900	H	-7.81115200	-4.63174900	-0.57237700
C	-6.09956800	-7.90124600	2.53181800	C	-7.01764000	-7.14855600	2.25038900
H	-4.20553100	-6.90332200	2.40840400	H	-5.10787000	-6.17545700	2.38132500
C	-7.42300700	-7.94130100	2.10964800	C	-8.26031300	-7.17754000	1.62736700
H	-8.89150900	-7.01257900	0.84370100	H	-9.51097600	-6.27789400	0.12748500
H	-5.73742200	-8.62381100	3.25521900	H	-6.79484000	-7.84109900	3.05383300
H	-8.10030700	-8.69647400	2.49112000	H	-9.00656900	-7.90072300	1.93410600
C	-6.27041300	5.89049200	-0.99972300	C	-5.52285500	6.63356000	-0.59143200
C	-6.12467300	7.22355800	-0.58128300	C	-5.20147500	7.86480100	0.00874300
C	-7.42278400	5.56540800	-1.73440000	C	-6.73512300	6.57884600	-1.30306100
C	-7.08557800	8.18241300	-0.86905700	C	-6.03052600	8.97255400	-0.10040400
H	-5.24572100	7.50462300	-0.01056100	H	-4.28789100	7.94355800	0.58864200
C	-8.37989000	6.52218400	-2.04109300	C	-7.56659800	7.68352800	-1.42482500
H	-7.55899700	4.54664900	-2.08156600	H	-7.02253700	5.64637600	-1.77893600
C	-8.21367400	7.83198900	-1.60337200	C	-7.21698500	8.88791600	-0.82240200
H	-6.95661300	9.20304100	-0.52553100	H	-5.75461300	9.90477300	0.38201200
H	-9.25631900	6.24987900	-2.61907300	H	-8.49010100	7.60772800	-1.98998400
H	-8.96346800	8.58067800	-1.83590100	H	-7.86597300	9.75280500	-0.91132600
N	5.03736100	-4.75104500	-0.50305600	N	4.39026900	-5.26255200	-0.38993700
N	-4.80480500	-5.00497900	0.61896600	N	-5.39264500	-4.35263200	0.44771100

Structure S30. The coordinates of both the optimized structures of B1N3L$_2$ at S$_0$ and S$_1$ in toluene.

S_0-optimized structure				S_1-optimized structure			
C	5.89498900	2.57755900	0.30001300	C	6.51906700	0.53365500	0.08632400
C	5.48025300	1.99297800	-0.89534300	C	5.83726200	0.11404400	-1.05645000
C	6.40391900	1.76326200	1.31156000	C	6.82598200	-0.40689000	1.07181200
C	5.48066200	0.61558700	-1.03793600	C	5.38036300	-1.18765700	-1.16220400
H	5.09798100	2.62530800	-1.68839100	H	5.61189900	0.83672100	-1.83193200
C	6.40438100	0.38410400	1.16115900	C	6.36267800	-1.70908900	0.96056100
H	6.75448400	2.21204700	2.23403200	H	7.38144200	-0.10011000	1.95057000
C	5.90066500	-0.21801800	0.00356700	C	5.59206200	-2.11354500	-0.13459600
H	5.08704800	0.17682700	-1.94802700	H	4.78984900	-1.47079300	-2.02644800
H	6.76934600	-0.23689300	1.97214100	H	6.57308600	-2.41396300	1.75769900
C	5.65612700	-1.67326500	-0.08368000	C	4.86846400	-3.40067600	-0.15792500
C	5.09180000	-2.34550500	1.00514800	C	4.22771500	-3.85780300	0.99938900
C	5.82658500	-2.38532700	-1.27551000	C	4.66347700	-4.11284300	-1.34561900
C	4.63011400	-3.64521700	0.88113600	C	3.33203800	-4.91296100	0.95046600
H	4.95105600	-1.82008400	1.94319900	H	4.37442000	-3.33097800	1.93544100
C	5.36425100	-3.68667500	-1.40690400	C	3.77246400	-5.17332400	-1.40169900
H	6.29390200	-1.90102400	-2.12617300	H	5.18005200	-3.80710800	-2.24856700
C	4.71423300	-4.30933500	-0.34139600	C	3.06017000	-5.54252000	-0.26215900
H	4.13916800	-4.13365000	1.71495900	H	2.78824000	-5.21265200	1.83909700
H	5.45950800	-4.20698200	-2.35318500	H	3.58339200	-5.68129500	-2.34056300
C	4.34547300	4.40532300	0.48746800	C	5.63548800	2.75610500	0.28752700
C	3.98057500	5.67737500	0.03165400	C	5.66214800	4.05664300	-0.22525600
C	3.32837000	3.53423700	0.88864000	C	4.42441200	2.27018500	0.78553100
C	2.64523900	6.04883300	-0.03227700	C	4.51046900	4.83102300	-0.24648900
H	4.74687500	6.37545500	-0.28407400	H	6.58963800	4.46185600	-0.61333800
C	2.00188800	3.90819300	0.80116800	C	3.27869400	3.03836500	0.73076400
H	3.57943700	2.54428200	1.24772200	H	4.37473200	1.26788000	1.19263000
C	1.62022800	5.17117900	0.33753600	C	3.28155400	4.34008700	0.21433600
H	2.39945500	7.04492900	-0.38446700	H	4.57240900	5.84001600	-0.63917200
H	1.24350000	3.18450100	1.07504400	H	2.35030900	2.60016300	1.07551600
C	0.18427400	5.48868700	0.20026600	C	2.01454700	5.09119400	0.12686300
C	-0.75130200	4.88475400	1.05068500	C	0.95581800	4.78758000	0.99422700
C	-0.30553300	6.30798000	-0.82221100	C	1.77107700	6.05460100	-0.86045600
C	-2.10734300	4.99299900	0.80799100	C	-0.30072200	5.32923400	0.80638400
H	-0.40882300	4.29521700	1.89356300	H	1.11549500	4.10406400	1.82097500
C	-1.67264200	6.46174700	-1.02332400	C	0.51400000	6.62849200	-1.01238500
H	0.38851200	6.78781200	-1.50405600	H	2.55836200	6.31791900	-1.55967200
C	-2.61220600	5.76526800	-0.25010800	C	-0.58370000	6.24253400	-0.22563200
H	-2.79703800	4.45070300	1.44655700	H	-1.10360200	5.02458900	1.47006800
H	-2.01363700	7.08432700	-1.84478300	H	0.36483500	7.34881100	-1.81147100
B	-4.13502400	5.67756300	-0.61740600	B	-2.08426000	6.66388400	-0.48423600
C	2.62820300	-5.49649600	-0.37416700	C	0.69719600	-5.95212600	-0.14399300
C	1.88730500	-6.57628400	0.11906800	C	-0.39660500	-6.76767700	0.20755900
C	1.94536100	-4.31941600	-0.69509800	C	0.47079300	-4.56869500	-0.26414600
C	0.51551200	-6.46582100	0.30593300	C	-1.64546000	-6.22004900	0.42278800
H	2.39221400	-7.49845800	0.38184700	H	-0.25278200	-7.83337700	0.33357200
C	0.58537600	-4.21277000	-0.48215000	C	-0.77020100	-4.03418800	-0.02234000
H	2.48813700	-3.47907100	-1.10888900	H	1.27550800	-3.91596700	-0.57303300
C	-0.16341200	-5.27453300	0.03460500	C	-1.87240800	-4.83432200	0.33089700
H	-0.02678900	-7.30919400	0.72069000	H	-2.45513800	-6.87822000	0.71842900
H	0.08388100	-3.28923600	-0.74924900	H	-0.90578200	-2.96989100	-0.16970900
C	-1.60344000	-5.06768600	0.28279500	C	-3.16432900	-4.20477800	0.54554100
C	-2.02530200	-3.85184300	0.83069700	C	-3.22548600	-2.86680100	0.98896200
C	-2.59000800	-5.99574500	-0.06331800	C	-4.39005000	-4.84304300	0.26150000
C	-3.36506800	-3.55092600	0.97316200	C	-4.40748500	-2.17817400	1.03477200
H	-1.28477000	-3.13229900	1.16177400	H	-2.32240600	-2.36981200	1.32022300
C	-3.94116000	-5.69350100	0.05553300	C	-5.58902500	-4.16475300	0.30654400
H	-2.30072900	-6.94775700	-0.49613700	H	-4.38771600	-5.87082800	-0.08268000
C	-4.34659400	-4.45000300	0.54790200	C	-5.61576300	-2.79472600	0.64403600
H	-3.66085500	-2.59823800	1.39520300	H	-4.42570800	-1.15235300	1.37857700
H	-4.68524000	-6.40910600	-0.27491300	H	-6.50192900	-4.66260800	0.00364100
C	-4.72566300	4.22483300	-0.63128800	C	-3.09270200	5.49336400	-0.54558800
C	-5.98959900	3.86381400	-0.13875000	C	-4.45419500	5.53549100	-0.14044900
C	-3.91158900	3.18125500	-1.09963800	C	-2.64703100	4.22128100	-0.99228000
C	-6.39095000	2.53527900	-0.05863900	C	-5.25724300	4.41399000	-0.10522900
H	-6.66081400	4.63563900	0.22479700	H	-4.86810300	6.47701600	0.20764600
C	-4.32027200	1.86221200	-1.06115000	C	-3.45473700	3.11340900	-1.02108000
H	-2.92332400	3.40974200	-1.48300800	H	-1.61966900	4.11842500	-1.32414500
C	-5.55602300	1.50692900	-0.50841000	C	-4.78596800	3.15306000	-0.54178300
H	-7.36395500	2.29271900	0.35599500	H	-6.27863000	4.50802400	0.25137100
H	-3.64262000	1.08578400	-1.39748000	H	-3.03253800	2.16983500	-1.34826700
C	-5.85796700	0.07173400	-0.33529000	C	-5.52884100	1.91662200	-0.42676700
C	-5.40292100	-0.86275100	-1.27287900	C	-5.21336800	0.81216200	-1.25852700
C	-6.45808100	-0.41603700	0.83017300	C	-6.50361300	1.69136000	0.57733400
C	-5.43966900	-2.21825600	-1.00617400	C	-5.68784500	-0.44725700	-0.99516100
H	-4.99444600	-0.52028900	-2.21691400	H	-4.57519500	0.96103100	-2.12061200
C	-6.49100900	-1.77595100	1.10878900	C	-6.98615000	0.42796700	0.85009000
H	-6.82294000	0.28184700	1.57604100	H	-6.80535900	2.51190000	1.21802900
C	-5.92356700	-2.68584600	0.21639100	C	-6.53172200	-0.67333200	0.10477800
H	-5.04083600	-2.92753700	-1.72207100	H	-5.40291900	-1.28295500	-1.62502400
H	-6.88932000	-2.12787600	2.05357700	H	-7.65556900	0.27064700	1.68855900
C	6.74826100	4.78612800	0.91342600	C	8.03732200	2.35414400	0.68788100
C	6.55775600	5.84156800	1.81136500	C	8.18513900	3.42485500	1.57572300
C	8.03737900	4.53741800	0.42989600	C	9.18503600	1.70947800	0.21241700
C	7.62986800	6.63275900	2.19821800	C	9.45037300	3.84104400	1.96423600
H	5.57003200	6.03631000	2.21096500	H	7.30733900	3.92616800	1.96447100
C	9.10373200	5.32489900	0.83703500	C	10.44323800	2.12418800	0.62148200
H	8.19696200	3.72517000	-0.26935000	H	9.08350300	0.88542200	-0.48403300
C	8.90991200	6.38190100	1.71868600	C	10.58833200	3.19541300	1.49564800
H	7.46008200	7.44561600	2.89599600	H	9.54223800	4.67330400	2.65367900
H	10.09444300	5.11425000	0.44915600	H	11.31902400	1.61065000	0.23975600
H	9.74449300	6.99988900	2.02878800	H	11.57377200	3.52238800	1.80652300
C	4.71146900	-6.65887300	-1.02103100	C	2.22236100	-7.77009900	-0.80711700
C	6.05029600	-6.86923600	-0.67265900	C	3.35592000	-8.44699800	-0.35095600

C	4.09117300	-7.56563900	-1.88688100	C	1.38060100	-8.38943500	-1.73484700
C	6.74550500	-7.95657200	-1.17961600	C	3.63470800	-9.72514700	-0.81058700
H	6.54072100	-6.17614300	0.00062700	H	4.01180600	-7.96667600	0.36543900
C	4.79277900	-8.65890700	-2.37444900	C	1.66192800	-9.67375500	-2.17705200
H	3.06123800	-7.40884500	-2.18296900	H	0.51701900	-7.85874700	-2.11741800
C	6.12312800	-8.86392500	-2.02910300	C	2.78790200	-10.34867300	-1.71988000
H	7.78244300	-8.09969000	-0.89563500	H	4.51694300	-10.23964100	-0.44686400
H	4.29267800	-9.34921500	-3.04519000	H	1.00257700	-10.14190800	-2.89928800
H	6.66726500	-9.71699000	-2.41740100	H	3.00647100	-11.34951800	-2.07335800
C	-6.73270400	-4.84909200	1.05535000	C	-8.04157400	-2.48342800	0.77819200
C	-8.04957300	-4.63516500	0.63152500	C	-9.12600200	-1.96131500	0.06376200
C	-6.48033400	-5.87370300	1.97432500	C	-8.26142200	-3.45345800	1.76408600
C	-9.08052100	-5.42359400	1.11958300	C	-10.40669000	-2.41720400	0.32674500
H	-8.25874100	-3.85091600	-0.08626900	H	-8.95066600	-1.21415900	-0.70026000
C	-7.51879300	-6.66573800	2.44212800	C	-9.54709900	-3.90515700	2.01012700
H	-5.46994000	-6.04334800	2.32578600	H	-7.42841600	-3.82623000	2.34767400
C	-8.82610500	-6.44821300	2.02387500	C	-10.62499100	-3.39205300	1.29526200
H	-10.09290500	-5.23960100	0.77648300	H	-11.24004800	-2.01343500	-0.23630800
H	-7.29983100	-7.45408000	3.15425700	H	-9.71010800	-4.65040500	2.78007400
H	-9.63398000	-7.06678600	2.39701400	H	-11.62958900	-3.74460600	1.49673100
C	-4.97817600	6.93523700	-0.97780400	C	-2.47925800	8.16305900	-0.66338900
C	-4.57314100	8.21758600	-0.57179400	C	-1.64404800	9.20196100	-0.20630500
C	-6.16663900	6.83737100	-1.72020200	C	-3.66580800	8.55874000	-1.31290600
C	-5.32134900	9.34516700	-0.87913200	C	-1.97367300	10.53956200	-0.37060800
H	-3.65999500	8.32461400	0.00435100	H	-0.71819200	8.94405000	0.29842800
C	-6.91183200	7.96179700	-2.04584700	C	-4.00173800	9.89351600	-1.48438500
H	-6.50047600	5.86209800	-2.05837800	H	-4.32682600	7.79448400	-1.70860800
C	-6.49130500	9.21757600	-1.62040700	C	-3.15753500	10.89449400	-1.01085400
H	-4.99380300	10.32378400	-0.54535300	H	-1.30723700	11.31093800	0.00259200
H	-7.82073700	7.86246800	-2.62926300	H	-4.92184900	10.15821300	-1.99607600
H	-7.07504000	10.09774000	-1.86833100	H	-3.41753500	11.93941400	-1.14351300
N	4.03045400	-5.54243400	-0.51118100	N	1.97288400	-6.45823400	-0.35232800
N	-5.69978100	-4.04170300	0.56365200	N	-6.74937600	-2.00712600	0.52241100
N	5.68366000	3.96677100	0.50511500	N	6.77114700	1.91410000	0.27269600

Structure S31. The coordinates of both the optimized structures of N4L$_2$ at S$_0$ and S$_1$ in toluene.

S_0-optimized structure				S_1-optimized structure			
C	5.42038000	3.33880100	0.31943200	C	5.20682400	3.74430600	0.24452400
C	5.07958100	2.68708200	-0.86470100	C	4.94132700	3.05332300	-0.93681600
C	6.05847600	2.61821500	1.32926800	C	5.90043200	3.09686200	1.26741400
C	5.28712800	1.32478300	-0.99913800	C	5.26967000	1.71355400	-1.05218400
H	4.59641400	3.24770600	-1.65662700	H	4.41613000	3.55913300	-1.73877400
C	6.26468900	1.25348900	1.18761500	C	6.22719100	1.75432400	1.14476600
H	6.35110900	3.12266500	2.24316700	H	6.13570400	3.63517800	2.17852000
C	5.84512000	0.57321200	0.03989200	C	5.87574500	1.02472300	0.00402200
H	4.95302100	0.82390300	-1.90095100	H	4.98817800	1.17509900	-1.95017500
H	6.72969500	0.70134300	1.99726200	H	6.72935100	1.25443600	1.96608600
C	5.82790500	-0.90280700	-0.04183400	C	5.96536700	-0.44942000	-0.04839300
C	5.36619200	-1.65038700	1.04599300	C	5.56788800	-1.20410800	1.06041100
C	6.11187100	-1.58301800	-1.23069300	C	6.27756000	-1.13379500	-1.22813500
C	5.10376200	-3.00455600	0.92157900	C	5.38766200	-2.57366000	0.96449100
H	5.14299500	-1.15043000	1.98200600	H	5.32449400	-0.70162600	1.98987700
C	5.85069600	-2.93920800	-1.36187600	C	6.09833900	-2.50537600	-1.33107900
H	6.50616600	-1.03478100	-2.07954200	H	6.62531000	-0.57893900	-2.09286500
C	5.29182300	-3.64950200	-0.29953100	C	5.59652900	-3.22765300	-0.24846800
H	4.68460900	-3.55975200	1.75281900	H	5.01352600	-3.13741800	1.81131400
H	6.02801000	-3.44074600	-2.30653300	H	6.29186700	-3.01275000	-2.26939500
C	3.62609400	4.92898900	0.48199200	C	3.25705600	5.13677400	0.39574700
C	3.08540700	6.09870700	-0.06212000	C	2.59080200	6.27096700	-0.08799900
C	2.74550200	3.95528700	0.96054500	C	2.47977800	4.04928900	0.81105700
C	1.71066000	6.27088200	-0.13936900	C	1.20806600	6.30138600	-0.16084700
H	3.74600500	6.87223200	-0.43597000	H	3.16313300	7.13090500	-0.41560900
C	1.37847800	4.12855100	0.86051600	C	1.10490400	4.08269300	0.71558900
H	3.13863900	3.04127100	1.38797100	H	2.96362100	3.15806600	1.19018800
C	0.82263300	5.28523600	0.30475800	C	0.41920200	5.20545000	0.22611700
H	1.32358000	7.19184800	-0.56221200	H	0.73292600	7.20384300	-0.52953200
H	0.72837000	3.32876600	1.19563900	H	0.55397100	3.19557400	1.00215600
C	-0.64266300	5.38157200	0.15041800	C	-1.04091700	5.17442100	0.09780400
C	-1.49262200	4.76320900	1.07520300	C	-1.80680600	4.28016100	0.87059800
C	-1.23319400	5.99269700	-0.96125300	C	-1.73780300	5.96246900	-0.83528500
C	-2.85493800	4.68028600	0.85526100	C	-3.15611100	4.11589400	0.66241000
H	-1.07924400	4.33390200	1.98078000	H	-1.33483700	3.70747600	1.65953700
C	-2.60009900	5.91218500	-1.19014200	C	-3.09832400	5.82153700	-1.04044700
H	-0.60652800	6.47664300	-1.70255600	H	-1.19230700	6.65828900	-1.46265000
C	-3.41971100	5.21333400	-0.30361600	C	-3.82641000	4.86404600	-0.31656500
H	-3.49261700	4.16917200	1.56726700	H	-3.71707200	3.40204900	1.25387600
H	-3.02713900	6.34238800	-2.08895200	H	-3.59492000	6.40814800	-1.80478700
C	3.39004500	-5.10823700	-0.35971000	C	3.78255600	-4.79634700	-0.26356900
C	2.79106600	-6.26934100	0.14113400	C	3.25569900	-6.00788900	0.20419900
C	2.56279600	-4.03713800	-0.71020500	C	2.88701000	-3.76290000	-0.56417100
C	1.41410800	-6.33716400	0.31090900	C	1.88907200	-6.16319400	0.38188800
H	3.40922100	-7.11289800	0.42538000	H	3.92396100	-6.82343000	0.45432900
C	1.19796300	-4.10689000	-0.51484800	C	1.53286900	-3.91907400	-0.35853800
H	2.99633000	-3.13893000	-1.13128100	H	3.25932500	-2.82796000	-0.96268900
C	0.58860700	-5.24896100	0.01366800	C	0.99235100	-5.11602800	0.13075800
H	0.98149500	-7.23782700	0.73417400	H	1.51755300	-7.10301700	0.77682700
H	0.58318500	-3.26135600	-0.80266900	H	0.86957200	-3.10266100	-0.62042000
C	-0.86738400	-5.22490900	0.25291000	C	-0.45440300	-5.19941300	0.36010000
C	-1.44373500	-4.06327900	0.77686300	C	-1.14143600	-4.06316600	0.81951400
C	-1.72599400	-6.27859200	-0.07392300	C	-1.21767800	-6.34498200	0.08657500
C	-2.81091600	-3.93553400	0.91788400	C	-2.51210800	-4.04358600	0.92971000
H	-0.80323700	-3.24711800	1.09209700	H	-0.57927500	-3.18391100	1.11242900
C	-3.10474200	-6.15203000	0.04395400	C	-2.59910300	-6.33588700	0.18293500
H	-1.31636200	-7.19346900	-0.48941300	H	-0.72430400	-7.24067100	-0.27574400
C	-3.66868600	-4.96312600	0.51600400	C	-3.27377400	-5.16884700	0.57657700
H	-3.22609800	-3.02093500	1.32290100	H	-3.01718000	-3.15401600	1.28495900
H	-3.74932600	-6.96489400	-0.27012000	H	-3.16596100	-7.21618000	-0.09687600
C	-5.13386000	3.57295900	-0.63303200	C	-5.45865400	3.18357500	-0.72829500
C	-6.29482400	3.07961800	-0.03097000	C	-6.51107100	2.56424600	-0.01625200
C	-4.25590600	2.66435200	-1.22748800	C	-4.59534800	2.36074600	-1.48128800
C	-6.53410500	1.71294300	0.01858300	C	-6.62688200	1.19572500	0.00783700
H	-6.99877500	3.76807100	0.42222800	H	-7.20545300	3.17618300	0.54922900
C	-4.49451600	1.30568000	-1.15875800	C	-4.73247300	0.99990600	-1.47775900
H	-3.35577200	3.02950600	-1.70700700	H	-3.80115600	2.82252200	-2.05717800
C	-5.62706800	0.79276600	-0.51750100	C	-5.71747400	0.33907500	-0.68289400
H	-7.43670600	1.35688000	0.50369700	H	-7.43479700	0.76208900	0.58585700
H	-3.75887900	0.62604800	-1.57265500	H	-4.02302100	0.41821200	-2.05156300
C	-5.74396400	-0.66865400	-0.33951300	C	-5.69506700	-1.08318500	-0.52448400
C	-5.22902400	-1.54501500	-1.30219400	C	-4.87784700	-1.90983900	-1.35585600
C	-6.22727200	-1.22476700	0.85008700	C	-6.37401900	-1.75896500	0.53425200
C	-5.09120800	-2.89557200	-1.03965900	C	-4.61986100	-3.21701500	-1.04852100
H	-4.90857100	-1.15894700	-2.26340100	H	-4.44834000	-1.50398200	-2.26296400
C	-6.09062400	-2.57940900	1.12133700	C	-6.12108000	-3.07311400	0.84416800
H	-6.63505500	-0.57576400	1.61757200	H	-7.04788400	-1.20600500	1.17799200
C	-5.46234500	-3.41655100	0.19977900	C	-5.17938600	-3.81846000	0.09864600
H	-4.64285400	-3.55328800	-1.77537900	H	-3.96692400	-3.80555000	-1.68387600
H	-6.40177700	-2.97615700	2.08108500	H	-6.59590100	-3.52475400	1.70828300
C	5.94751000	5.65953300	0.89632800	C	5.48772600	6.10530800	0.81884600
C	5.59381000	6.71559700	1.74316800	C	5.03498200	7.10236700	1.68977400
C	7.26791600	5.57648400	0.44009200	C	6.80265400	6.17033700	0.34441400
C	6.53636500	7.66614700	2.10756700	C	5.87606900	8.14173700	2.05937200
H	4.58119000	6.78643700	2.12099800	H	4.02661200	7.05621100	2.08243400
C	8.20462300	6.52374700	0.82515900	C	7.63842500	7.20572300	0.73419200
H	7.55410500	4.76678100	-0.22058600	H	7.16282000	5.40634300	-0.33433900
C	7.84795200	7.57919100	1.65635300	C	7.18255500	8.20276300	1.58897100
H	6.24024700	8.47610000	2.76543700	H	5.50582600	8.90370700	2.73668300
H	9.22203300	6.43909000	0.45893600	H	8.65373000	7.23688600	0.35412700
H	8.58114200	8.32163100	1.94915500	H	7.83646900	9.01456900	1.88551000
C	5.62201300	-5.98021600	-0.96900100	C	6.05599300	-5.54406000	-0.88125100
C	6.97555200	-5.99695300	-0.61374300	C	7.40250700	-5.49212300	-0.50468600
C	5.13982800	-6.97395300	-1.82752000	C	5.63758700	-6.55642300	-1.75116700

C	7.81905400	-6.98085400	-1.10675400	C	8.30283800	-6.42955600	-0.98747600
H	7.36050400	-5.23544300	0.05420400	H	7.73648600	-4.71382100	0.17127000
C	5.99064400	-7.96239800	-2.30109100	C	6.54490100	-7.49813400	-2.21470600
H	4.09968900	-6.96641000	-2.12931800	H	4.60337600	-6.59854800	-2.07016300
C	7.33493400	-7.97496500	-1.94916100	C	7.88231700	-7.44337000	-1.84061300
H	8.86432500	-6.97387500	-0.81738900	H	9.34187500	-6.37088100	-0.68216100
H	5.59569200	-8.72256400	-2.96646900	H	6.20046500	-8.27422200	-2.88959800
H	7.99565700	-8.74666000	-2.32668500	H	8.58724800	-8.17881600	-2.21045800
C	-5.98247000	-5.66794000	1.03150500	C	-5.52016900	-6.07222600	0.98295500
C	-7.31921200	-5.61605500	0.61883900	C	-6.79733600	-6.20624700	0.42426300
C	-5.59976800	-6.65573400	1.94593600	C	-5.11982500	-6.95590600	1.99337900
C	-8.24206700	-6.52593000	1.11178400	C	-7.64845900	-7.20536300	0.86780300
H	-7.62855600	-4.86095600	-0.09398900	H	-7.10444000	-5.53051500	-0.36464900
C	-6.52999400	-7.56975900	2.41909800	C	-5.97751000	-7.95714100	2.41916200
H	-4.57404000	-6.70095000	2.29079500	H	-4.14423600	-6.84194400	2.44986600
C	-7.85725800	-7.51366800	2.01103700	C	-7.24621400	-8.08933200	1.86362400
H	-9.27200500	-6.46618500	0.77687900	H	-8.63193200	-7.30039800	0.42115000
H	-6.21056100	-8.32627000	3.12778600	H	-5.65523700	-8.63137600	3.20486100
H	-8.58027700	-8.22734300	2.38846100	H	-7.91474500	-8.87099700	2.20513000
C	-5.67988000	5.96384600	-0.91260500	C	-6.14143900	5.53678800	-0.76494100
C	-5.49105200	7.25279000	-0.40005400	C	-6.01763600	6.77363900	-0.11800800
C	-6.77613800	5.72808400	-1.75031300	C	-7.26770500	5.29245100	-1.56165800
C	-6.37325100	8.27259200	-0.72370400	C	-6.99460400	7.74225700	-0.27928400
H	-4.65213500	7.44942700	0.25667600	H	-5.16222700	6.96104700	0.51937500
C	-7.66001700	6.75346800	-2.05262300	C	-8.24250500	6.26658300	-1.70313300
H	-6.92928900	4.74109100	-2.16945500	H	-7.35968300	4.34340000	-2.07527900
C	-7.46724300	8.03368100	-1.54721200	C	-8.11424800	7.49757600	-1.06820700
H	-6.20669400	9.26312400	-0.31425400	H	-6.88574600	8.69342900	0.22987100
H	-8.50217900	6.54707100	-2.70435700	H	-9.10570000	6.06409400	-2.32739700
H	-8.15773500	8.83240000	-1.79165100	H	-8.87847000	8.25702400	-1.18546600
N	4.78853400	-4.96651100	-0.47307000	N	5.16503800	-4.57313200	-0.39661300
N	-5.06284900	-4.73630700	0.53498300	N	-4.67166900	-5.04870200	0.54457600
N	-4.78053200	4.93999600	-0.59301700	N	-5.16449700	4.55005800	-0.60799100
N	5.01461500	4.68523700	0.51174400	N	4.66022100	5.04138900	0.42824400

Structure S32. The coordinates of both the optimized structures of $B5L_2$ at S_0 and S_1 in toluene.

S_0-optimized structure				S_1-optimized structure			
B	6.96234400	-4.93401500	1.33096000	B	-7.48399300	-4.21323000	-1.06243600
C	5.55296900	-5.54996500	1.04362200	C	-6.13345600	-4.95334500	-0.79450200
C	5.33625900	-6.90886700	0.76293500	C	-6.02045300	-6.34502700	-0.62495600
C	4.41871900	-4.72316600	1.07540700	C	-4.93617700	-4.21848100	-0.74966500
C	4.06644600	-7.40981700	0.51496900	C	-4.79583200	-6.96226700	-0.42820800
H	6.18316000	-7.58629900	0.72558400	H	-6.91648000	-6.95660000	-0.64255900
C	3.14530000	-5.22255500	0.86646000	C	-3.70835500	-4.82870100	-0.57907100
H	4.53964700	-3.66394900	1.27415300	H	-4.97167100	-3.14068100	-0.86153800
C	2.94498900	-6.57556200	0.57449600	C	-3.60727800	-6.21761300	-0.41756000
H	3.93704400	-8.46518400	0.29956200	H	-4.75370200	-8.04014300	-0.31653500
H	2.29522500	-4.54979000	0.88986600	H	-2.81441800	-4.21743700	-0.53703800
C	1.57831900	-7.07046500	0.31355400	C	-2.28838800	-6.84225300	-0.23919800
C	0.49451500	-6.56266300	1.03903800	C	-1.14874900	-6.25885100	-0.81480100
C	1.31663300	-8.00729400	-0.69052100	C	-2.10590500	-8.00584800	0.52271500
C	-0.80281500	-6.92425100	0.72110700	C	0.10878500	-6.77943500	-0.58935200
H	0.67482900	-5.87267300	1.85607500	H	-1.25736600	-5.39300200	-1.45786600
C	0.01404300	-8.38481700	-0.98537900	C	-0.84241300	-8.53597800	0.72384700
H	2.13739700	-8.40886200	-1.27515400	H	-2.96070400	-8.47424700	0.99853500
C	-1.08671700	-7.82911700	-0.31462600	C	0.31692600	-7.92500400	0.20483700
H	-1.62025600	-6.49051600	1.28723100	H	0.96183800	-6.29230100	-1.04947100
H	-0.15651500	-9.09476200	-1.78805300	H	-0.74035600	-9.42395100	1.33895700
B	-2.55797200	-8.11981400	-0.76832400	B	1.76077000	-8.44227700	0.50444200
C	7.21222000	-3.45164500	0.88814300	C	-7.59957900	-2.71300900	-0.62229100
C	6.77950100	-2.98326300	-0.36182100	C	-7.12128400	-2.26581800	0.61901200
C	7.79368700	-2.51304800	1.75339700	C	-8.08695800	-1.73704500	-1.50459200
C	6.91432500	-1.65376800	-0.72736100	C	-7.11147600	-0.92156100	0.95415800
H	6.31532100	-3.67493700	-1.05740800	H	-6.73153600	-2.98716400	1.33012000
C	7.87372200	-1.16844800	1.41900300	C	-8.02447600	-0.38476600	-1.19983700
H	8.15949600	-2.83718900	2.72246800	H	-8.48852600	-2.04191100	-2.46566900
C	7.43088100	-0.71658300	0.17296000	C	-7.52551300	0.04589200	0.03250000
H	6.55829100	-1.32057900	-1.69647400	H	-6.71710600	-0.60715700	1.91466700
H	8.29589000	-0.45976900	2.12383700	H	-8.37445400	0.34823500	-1.91913100
C	7.43051200	0.72020200	-0.17290700	C	-7.35434900	1.48139800	0.33630000
C	6.91349800	1.65712300	0.72742600	C	-6.76693600	2.32933800	-0.60848700
C	7.87310400	1.17229300	-1.41895800	C	-7.69707700	2.01450100	1.58197400
C	6.77799900	2.98655100	0.36189400	C	-6.46759700	3.64339200	-0.28853900
H	6.55764400	1.32375300	1.69654200	H	-6.48616600	1.93191200	-1.57806000
C	7.79238900	2.51685500	-1.75334200	C	-7.44867100	3.34837800	1.87331500
H	8.29561700	0.46382900	-2.12380000	H	-8.17330100	1.37913100	2.32139100
C	7.21046900	3.45515600	-0.88807000	C	-6.79493600	4.19080300	0.96147600
H	6.31347900	3.67799100	1.05748800	H	-5.95425100	4.25841900	-1.02059700
H	8.15802100	2.84118600	-2.72241800	H	-7.73777100	3.73760100	2.84431000
B	6.95986000	4.93739000	-1.33090100	B	-6.35717800	5.64308500	1.35403800
C	5.55019200	5.55266100	-1.04354600	C	-4.90411700	6.09280800	0.98671000
C	5.33279300	6.91147200	-0.76296500	C	-4.55081900	7.41796600	0.68320400
C	4.41637100	4.72526600	-1.07520200	C	-3.86821700	5.14502200	0.97208100
C	4.06272900	7.41178400	-0.51499700	C	-3.24603200	7.77406300	0.37481600
H	6.17934500	7.58934600	-0.72571400	H	-5.31802400	8.18534400	0.67915900
C	3.14270000	5.22401300	-0.86626200	C	-2.55842000	5.49883000	0.70050800
H	4.53784700	3.66608900	-1.27382500	H	-4.09606200	4.10671200	1.18654500
C	2.94170100	6.57694700	-0.57442000	C	-2.22177600	6.82089900	0.39160800
H	3.93278200	8.46709700	-0.29965600	H	-3.01027700	8.80840800	0.14798600
H	2.29296900	4.55080900	-0.88957800	H	-1.78827200	4.73553500	0.69139200
C	1.57478600	7.07119000	-0.31347400	C	-0.82029500	7.16543500	0.08022500
C	0.49122400	6.56284900	-1.03894300	C	0.22681500	6.53666700	0.76367500
C	1.31265200	8.00791900	0.69057400	C	-0.49269700	8.08320100	-0.92291100
C	-0.80627900	6.92381900	-0.72102500	C	1.54436800	6.77142300	0.41289500
H	0.67187500	5.87292800	-1.85596300	H	0.00163900	5.85717100	1.57830000
C	0.00988000	8.38482400	0.98542000	C	0.83244300	8.33261900	-1.25090900
H	2.13322400	8.40989600	1.27519500	H	-1.28465100	8.57463900	-1.47791700
C	-1.09061500	7.82857500	0.31468500	C	1.89073700	7.66308400	-0.61573300
H	-1.62351600	6.48967400	-1.28713000	H	2.32921000	6.25051400	0.95077900
H	-0.16102100	9.09470500	1.78807700	H	1.05102100	9.03404000	-2.04946700
C	-3.59287500	-6.94610400	-0.72782100	C	2.89711100	-7.38450400	0.63360600
C	-4.96973900	-7.14467300	-0.53159400	C	4.24644800	-7.62859500	0.25730300
C	-3.16873500	-5.61515100	-0.87223800	C	2.62076600	-6.07770200	1.11964200
C	-5.86155200	-6.08464000	-0.46303400	C	5.20544000	-6.64883500	0.28316300
H	-5.34969300	-8.15444400	-0.41688200	H	4.51661600	-8.61484000	-0.10352500
C	-4.05326700	-4.55342900	-0.81516000	C	3.56871100	-5.09428600	1.16812400
H	-2.11671900	-5.40697300	-1.03113400	H	1.61952400	-5.85264500	1.46786500
C	-5.42033600	-4.76335700	-0.60165200	C	4.90400500	-5.31969400	0.71200600
H	-6.91717800	-6.28689300	-0.31861500	H	6.20123000	-6.88714500	-0.06744800
H	-3.67096500	-3.54303900	-0.90201200	H	3.28893300	-4.12988100	1.56915200
C	-6.33944700	-3.61029800	-0.50520000	C	5.85569100	-4.25592500	0.65266400
C	-6.04925400	-2.41587800	-1.17512000	C	5.46293100	-2.90211700	0.88417600
C	-7.49389100	-3.65033300	0.28467100	C	7.23015400	-4.46313500	0.32210100
C	-6.84022800	-1.29412700	-1.00408900	C	6.34711100	-1.86769400	0.75512800
H	-5.18987500	-2.36264200	-1.83328300	H	4.43638000	-2.67131200	1.13194500
C	-8.31377800	-2.53697600	0.40755100	C	8.10998300	-3.41629400	0.22374600
H	-7.73731300	-4.55198700	0.83564700	H	7.59755300	-5.46750200	0.15510500
C	-7.99601600	-1.31766700	-0.20847200	C	7.71190000	-2.06315600	0.41046900
H	-6.55984900	-0.37460500	-1.50700300	H	5.99255000	-0.85830200	0.92645200
H	-9.19858100	-2.60411200	1.03255400	H	9.14295100	-3.62529800	-0.03128400
B	-2.56200100	8.11861600	0.76838700	B	3.38064800	7.83106500	-1.07082700
B	-8.82115700	-0.00214200	0.00003300	B	8.67082600	-0.84559800	0.26211900
C	-3.59635000	6.94440800	0.72795000	C	4.30738300	6.57146300	-1.03038800
C	-4.97329700	7.14231800	0.53164300	C	5.68508700	6.64160400	-0.75685500
C	-3.17158800	5.61366600	0.87247900	C	3.77562100	5.28576800	-1.23068500
C	-5.86460300	6.08185500	0.46309000	C	6.47015800	5.50546300	-0.64867600
H	-5.35372200	8.15190600	0.41687900	H	6.14654000	7.61163200	-0.60398800
C	-4.05561100	4.55152000	0.81541100	C	4.55577700	4.14885000	-1.14761700
H	-2.11948100	5.40599900	1.03144300	H	2.71905600	5.17715700	-1.44882800
C	-5.42276800	4.76079100	0.60180300	C	5.92136600	4.22762100	-0.83505500
H	-6.92031800	6.28359100	0.31859100	H	7.52771600	5.60999400	-0.43345300

H	-3.67283200	3.54131900	0.90235500	H	4.08864300	3.17965600	-1.27605500
C	-6.34132600	3.60728500	0.50535300	C	6.70884700	2.99860600	-0.66439700
C	-6.05062900	2.41305900	1.17539700	C	6.33391000	1.81142700	-1.31574700
C	-7.49570500	3.64669900	-0.28465000	C	7.81367700	2.93932400	0.19912100
C	-6.84101800	1.29089700	1.00434200	C	6.97419400	0.61915700	-1.04828600
H	-5.19130500	2.36030100	1.83366900	H	5.52820400	1.82832500	-2.04056900
C	-8.31501900	2.53292500	-0.40755200	C	8.47254400	1.74519800	0.43585300
H	-7.73951500	4.54820300	-0.83569900	H	8.12600000	3.82951700	0.73348200
C	-7.99671200	1.31380700	0.20857400	C	8.04785800	0.53073100	-0.13898100
H	-6.56024500	0.37154900	1.50735400	H	6.63613100	-0.27805000	-1.55626400
H	-9.19977900	2.59957700	-1.03266800	H	9.30486000	1.73640300	1.13186200
C	-10.37904300	-0.00255400	-0.00004800	C	10.20175300	-0.97250300	0.54947100
C	-11.10903700	-1.02325400	-0.63179700	C	10.71900700	-1.89464000	1.47794600
C	-11.10969600	1.01774400	0.63158900	C	11.13203500	-0.14792000	-0.11034800
C	-12.49666900	-1.02185800	-0.64267500	C	12.07968800	-1.99133400	1.73123800
H	-10.57461700	-1.82266900	-1.13406200	H	10.03572100	-2.53562800	2.02562700
C	-12.49732700	1.01556600	0.64226000	C	12.49557000	-0.24934100	0.12607300
H	-10.57579800	1.81746600	1.13392300	H	10.77352400	0.57591200	-0.83490400
C	-13.19262200	-0.00334000	-0.00025600	C	12.97452400	-1.17107300	1.05119900
H	-13.03765400	-1.81453400	-1.14797100	H	12.44576000	-2.70590000	2.46091800
H	-13.03882800	1.80794300	1.14747500	H	13.18762300	0.39160400	-0.40989400
H	-14.27747700	-0.00363500	-0.00033600	H	14.03932800	-1.24867700	1.24307300
C	-2.96603000	9.53698000	1.28450700	C	3.91479600	9.21307500	-1.56520100
C	-2.35079300	10.69304600	0.77674100	C	3.38636700	10.41665900	-1.06950200
C	-3.94838600	9.71198600	2.27293800	C	4.93802500	9.30650000	-2.52319000
C	-2.70960000	11.95970800	1.21688200	C	3.86371200	11.64902400	-1.49396800
H	-1.58605800	10.59326500	0.01343300	H	2.59595600	10.38120900	-0.32702700
C	-4.29335400	10.97371100	2.73793600	C	5.40374400	10.53505100	-2.97094400
H	-4.43712200	8.84025600	2.69517900	H	5.36347300	8.39710000	-2.93447200
C	-3.67889800	12.10110100	2.20389500	C	4.87094900	11.70965500	-2.45084700
H	-2.23001400	12.83714800	0.79666000	H	3.44841700	12.56317300	-1.08366100
H	-5.04412500	11.08023200	3.51352600	H	6.18486300	10.57889800	-3.72225400
H	-3.95385400	13.08892500	2.55802900	H	5.23953400	12.67120400	-2.79188000
C	8.06781700	5.74032400	-2.08128500	C	-7.32896500	6.57864100	2.13945900
C	9.42711600	5.43919800	-1.89355000	C	-8.72117900	6.44056600	2.01224100
C	7.75547300	6.78120000	-2.97143700	C	-6.85731100	7.58027400	3.00402100
C	10.42501800	6.15134700	-2.54404700	C	-9.59861200	7.27089100	2.69557400
H	9.70207700	4.63828500	-1.21527100	H	-9.11824400	5.67381400	1.35524400
C	8.74675600	7.48253200	-3.64375600	C	-7.72855600	8.39847500	3.70982200
H	6.71525100	7.03145700	-3.15050800	H	-5.78804000	7.70601100	3.13674400
C	10.08463300	7.17239100	-3.42474300	C	-9.10193400	8.24905200	3.55026200
H	11.46778900	5.90838900	-2.37082700	H	-10.66929100	7.15292800	2.56849100
H	8.47878900	8.27288900	-4.33651300	H	-7.33902000	9.15476600	4.38267900
H	10.86188300	7.72456700	-3.94224900	H	-9.78497000	8.89306900	4.09388500
C	8.07076200	-5.73646100	2.08118300	C	-8.66613400	-4.90325800	-1.81428200
C	9.42989800	-5.43461500	1.89341500	C	-9.99277500	-4.50475200	-1.57962800
C	7.75899800	-6.77759400	2.97123500	C	-8.45876700	-5.92791600	-2.75266400
C	10.42819500	-6.14631400	2.54379700	C	-11.05938100	-5.11206300	-2.22757300
H	9.70441600	-4.63348700	1.21521000	H	-10.18707500	-3.71038700	-0.86650700
C	8.75067200	-7.47848100	3.64344300	C	-9.51840200	-6.52325500	-3.42310200
H	6.71891200	-7.02840800	3.15032200	H	-7.44628000	-6.24896600	-2.97300700
C	10.08837900	-7.16762300	3.42440500	C	-10.82233200	-6.12098100	-3.15486700
H	11.47083400	-5.90279900	2.37055500	H	-12.07518500	-4.79562100	-2.01671400
H	8.48314400	-8.26904700	4.33613000	H	-9.32992600	-7.30255500	-4.15361900
H	10.86593500	-7.71945000	3.94182200	H	-11.65279100	-6.59092500	-3.67088200
C	-2.96133600	-9.53833900	-1.28453700	C	2.05523700	-9.96759500	0.67208900
C	-2.34558900	-10.69414900	-0.77680700	C	1.28837400	-10.93797100	0.00089800
C	-3.94357300	-9.71374600	-2.27301600	C	3.08986200	-10.43904100	1.50102500
C	-2.70380300	-11.96095300	-1.21702500	C	1.54643600	-12.29469800	0.13598000
H	-1.58092800	-10.59406100	-0.01346600	H	0.48491800	-10.61556600	-0.65315000
C	-4.28794500	-10.97560500	-2.73809300	C	3.34409300	-11.79442500	1.65339500
H	-4.43268800	-8.84221600	-2.69523100	H	3.69546500	-9.72506600	2.05005700
C	-3.67299400	-12.10274100	-2.20408400	C	2.57436700	-12.72823800	0.96683500
H	-2.22383500	-12.83819500	-0.79682600	H	0.94561800	-13.01716300	-0.40631800
H	-5.03863700	-11.08242900	-3.51371800	H	4.14295200	-12.12595700	2.30833800
H	-3.94748300	-13.09067400	-2.55827600	H	2.77455400	-13.78839800	1.07936400

Structure S33. The coordinates of both the optimized structures of B4N1L$_2$ at S$_0$ and S$_1$ in toluene.

S_0-optimized structure				S_1-optimized structure			
B	7.34721300	4.38225200	-1.24842300	B	-8.00429300	-3.08371000	1.28201300
C	5.99296800	5.11769400	-0.97786800	C	-7.75035900	-1.58380700	0.91850700
C	5.88978100	6.48950700	-0.69504600	C	-8.76773400	-0.67140800	0.59310300
C	4.79235200	4.39186200	-1.03079900	C	-6.43775600	-1.08496900	0.93313300
C	4.66405300	7.09771900	-0.46559300	C	-8.49164300	0.65442800	0.29190500
H	6.79129100	7.09109000	-0.64132600	H	-9.79796100	-1.01119700	0.56525000
C	3.56358000	4.99884800	-0.84059400	C	-6.15851300	0.24395800	0.66936200
H	4.82466500	3.32668200	-1.23175500	H	-5.61798000	-1.75601600	1.16475800
C	3.47606900	6.36341700	-0.54685500	C	-7.18023900	1.14037200	0.33873400
H	4.62270500	8.15983500	-0.24835500	H	-9.30379900	1.33056700	0.04649400
H	2.65896700	4.40222300	-0.88013500	H	-5.13049400	0.58841400	0.68277900
C	2.15215100	6.97170000	-0.30622200	C	-6.84510700	2.54603300	0.03663500
C	1.04298000	6.56104100	-1.05448400	C	-5.82847600	3.19198100	0.74909600
C	1.95171600	7.91962300	0.70137300	C	-7.48205400	3.25464800	-0.98699300
C	-0.22560700	7.02433300	-0.75331000	C	-5.42199800	4.46861400	0.40442600
H	1.18027700	5.86483500	-1.87464500	H	-5.35178800	2.68336300	1.57992200
C	0.67943300	8.39995600	0.97924400	C	-7.08494100	4.54506900	-1.30950300
H	2.79306100	8.24788000	1.30233400	H	-8.26529400	2.77382900	-1.56354700
C	-0.45173300	7.93992600	0.28716300	C	-6.02169600	5.17963800	-0.64792300
H	-1.06653700	6.66246900	-1.33536300	H	-4.61463700	4.92922700	0.96347500
H	0.55402400	9.11511900	1.78564800	H	-7.58400800	5.05792500	-2.12536700
B	-1.90213700	8.33688200	0.72799700	B	-5.46188900	6.57433000	-1.09731800
C	7.45817000	2.88054400	-0.81373000	C	-6.88826700	-4.11966100	0.91189900
C	6.97946600	2.44524500	0.43138300	C	-6.24153700	-4.07881700	-0.33294600
C	7.95261400	1.89870500	-1.68491200	C	-6.42166900	-5.06228100	1.84033900
C	6.98921100	1.10612800	0.78702600	C	-5.18503800	-4.92297800	-0.63237100
H	6.57895500	3.17182500	1.13100300	H	-6.56545600	-3.35812800	-1.07686500
C	7.90510600	0.54993400	-1.36117200	C	-5.32316600	-5.86646300	1.57057900
H	8.35060700	2.19489100	-2.65025700	H	-6.90723700	-5.14162800	2.80780400
C	7.41814500	0.13198200	-0.11986300	C	-4.68241700	-5.80538300	0.32980100
H	6.60152700	0.79986500	1.75289600	H	-4.69678500	-4.85593400	-1.59884100
H	8.26124500	-0.18987300	-2.07044600	H	-4.96247600	-6.55994800	2.32312200
C	7.28262700	-1.30180500	0.21205100	C	-3.45886200	-6.58318600	0.04915300
C	6.66992400	-2.17332200	-0.69361700	C	-2.41134600	-6.60394000	0.97618900
C	7.69309900	-1.80992500	1.44743600	C	-3.27822900	-7.24886500	-1.16679000
C	6.41612400	-3.48970500	-0.34420600	C	-1.20226300	-7.20309700	0.66259700
H	6.33623700	-1.79421400	-1.65377900	H	-2.53020000	-6.08945600	1.92400200
C	7.49361000	-3.14650900	1.76419700	C	-2.08495300	-7.89786400	-1.44851900
H	8.18603500	-1.15314600	2.15664500	H	-4.08635100	-7.26478200	-1.89077200
C	6.82044600	-4.01506600	0.89244400	C	-0.99774600	-7.86055600	-0.56118200
H	5.88188700	-4.12538900	-1.04291400	H	-0.38823900	-7.14874400	1.37857100
H	7.83963400	-3.51757000	2.72371500	H	-1.97895300	-8.41570500	-2.39644500
B	6.45351700	-5.48314700	1.30116900	B	0.40283300	-8.43440300	-0.96714300
C	4.99307400	-5.96809300	1.02285600	C	1.66512300	-7.55454700	-0.69875800
C	4.66050900	-7.28660100	0.67193000	C	2.95914700	-8.07528400	-0.50079200
C	3.93083600	-5.05591000	1.12780100	C	1.55073900	-6.15134300	-0.68665900
C	3.35006600	-7.66723600	0.42141800	C	4.05862600	-7.25780900	-0.31048200
H	5.44829100	-8.02726600	0.57916300	H	3.10140100	-9.15075000	-0.49128700
C	2.61738600	-5.43781100	0.91922000	C	2.64570100	-5.32614000	-0.52930800
H	4.14210700	-4.02279300	1.38162500	H	0.57367700	-5.70037300	-0.81843200
C	2.30135800	-6.74982600	0.55036700	C	3.93222500	-5.85815600	-0.33998900
H	3.13092400	-8.69430000	0.14879100	H	5.03709100	-7.70673200	-0.17814200
H	1.82535300	-4.70122700	0.99901000	H	2.49873000	-4.25234300	-0.51561200
C	0.89744600	-7.11275900	0.27914500	C	5.08057600	-4.96792700	-0.18369100
C	-0.13802100	-6.58226600	1.05658900	C	5.07896600	-3.69000700	-0.77645900
C	0.54563700	-7.93857700	-0.79226100	C	6.21781900	-5.33087100	0.56551000
C	-1.46433600	-6.83015600	0.75463800	C	6.11920300	-2.81040400	-0.59848200
H	0.10073200	-5.97957800	1.92589600	H	4.25883900	-3.39974600	-1.42113000
C	-0.78170900	-8.17100400	-1.11835600	C	7.27048700	-4.46156600	0.75131700
H	1.32256000	-8.35653500	-1.42323800	H	6.24761200	-6.29525200	1.05827600
C	-1.80633100	-7.59659600	-0.36281900	C	7.22977400	-3.17403600	0.18532100
H	-2.24813900	-6.41011700	1.37359500	H	6.10563500	-1.84448800	-1.08768800
H	-1.02771600	-8.77290200	-1.98518500	H	8.10769200	-4.74793000	1.37615300
C	-3.01621600	7.23849600	0.67860900	C	-3.91890100	6.79886600	-1.02968500
C	-4.37600700	7.53009700	0.47906700	C	-3.33281600	8.05016000	-0.76721500
C	-2.68387700	5.88154400	0.82170300	C	-3.03187400	5.71957500	-1.18913600
C	-5.33775300	6.53308700	0.40884300	C	-1.96227900	8.20593800	-0.63244000
H	-4.68631100	8.56333000	0.36365300	H	-3.97131000	8.91887200	-0.64284000
C	-3.63817000	4.88246400	0.76169700	C	-1.66319300	5.87090800	-1.07767700
H	-1.64885800	5.60212600	0.98251300	H	-3.43031900	4.73300100	-1.39879200
C	-4.98771400	5.18467500	0.54796800	C	-1.09205800	7.11576400	-0.77736300
H	-6.37708300	6.80693600	0.26377400	H	-1.55720500	9.19062800	-0.42592800
H	-3.32550000	3.84842900	0.84758900	H	-1.02625300	4.99950600	-1.17563400
C	-5.98282000	4.09617300	0.45498000	C	0.36155500	7.23096700	-0.57987000
C	-5.77082700	2.88512700	1.12427100	C	1.24495700	6.34888900	-1.21979800
C	-7.13629600	4.21397800	-0.32855700	C	0.91812200	8.17206300	0.29786400
C	-6.63856100	1.82045800	0.96033200	C	2.59658000	6.35977400	-0.93513600
H	-4.91342100	2.77333800	1.77767400	H	0.86504900	5.64837600	-1.95541200
C	-8.03200500	3.15973900	-0.44394400	C	2.27935600	8.19411600	0.55570400
H	-7.31993000	5.12942100	-0.87996700	H	0.26820400	8.86193600	0.82566000
C	-7.79582800	1.92274300	0.17335300	C	3.16671000	7.26222800	-0.01573900
H	-6.41916700	0.88434200	1.46288600	H	3.24008500	5.64774600	-1.44156700
H	-8.91455900	3.28722400	-1.06275600	H	2.66385900	8.92227200	1.26288300
B	-8.71476200	0.66672300	-0.01384600	B	4.67968600	7.17940500	0.37871200
C	-4.00553400	-6.62638500	-0.72238600	C	7.88880500	-0.90163800	0.57816800
C	-5.36849500	-6.73856200	-0.42803100	C	8.64882000	0.13341400	-0.00473200
C	-3.49506300	-5.35297300	-0.99314800	C	6.71552900	-0.56484600	1.28391900
C	-6.17883500	-5.61496600	-0.39335200	C	8.21002100	1.43355700	0.05914900
H	-5.79580300	-7.71281500	-0.22341000	H	9.54772300	-0.10862100	-0.55898600
C	-4.30728300	-4.23727600	-0.92579400	C	6.29410500	0.73689300	1.34848100
H	-2.44676400	-5.23434800	-1.23641400	H	6.16458600	-1.34226400	1.79955000
C	-5.67037300	-4.33188900	-0.62378900	C	6.99555900	1.78695800	0.70210800
H	-7.23346800	-5.74738700	-0.17970700	H	8.78129800	2.19379900	-0.45834300
H	-3.85746500	-3.26643900	-1.09560500	H	5.41449900	0.96330300	1.93621400

C	-6.50324000	-3.11624200	-0.52640300	C	6.46454100	3.13230000	0.67010900
C	-6.14534800	-1.94986800	-1.21466800	C	5.08871100	3.37060500	0.89642400
C	-7.64358200	-3.06231100	0.28463600	C	7.26086700	4.26352800	0.37363900
C	-6.84868700	-0.77219700	-1.03870200	C	4.55361700	4.63236200	0.79717800
H	-5.29887400	-1.96097000	-1.89133000	H	4.42326200	2.54183100	1.10746600
C	-8.38193000	-1.89363400	0.40714800	C	6.71724000	5.52794600	0.30843600
H	-7.94173800	-3.93573800	0.85387800	H	8.32708600	4.14478000	0.21544900
C	-7.99020900	-0.70418200	-0.22480800	C	5.33419500	5.77481400	0.49461600
H	-6.51085000	0.12017700	-1.55529800	H	3.48955400	4.75633900	0.96385200
H	-9.25848500	-1.89359500	1.04743100	H	7.37180100	6.36224400	0.07876200
C	-10.26896400	0.77895100	0.01490400	C	5.48931400	8.48698700	0.67581800
C	-10.91328900	1.85498400	0.64784900	C	5.17787200	9.70030100	0.03446700
C	-11.08148000	-0.19155700	-0.59469900	C	6.54958300	8.52012300	1.60048000
C	-12.29710800	1.95386600	0.68043600	C	5.88651000	10.86708300	0.28526300
H	-10.31460200	2.61850900	1.13321200	H	4.36670600	9.71994600	-0.68631800
C	-12.46545100	-0.08981000	-0.58315800	C	7.25512800	9.68513100	1.86756500
H	-10.61434200	-1.03171700	-1.09752600	H	6.81162300	7.61219500	2.13422000
C	-13.07507200	0.98233400	0.05964800	C	6.92936300	10.86502800	1.20603900
H	-12.77144200	2.78801300	1.18599500	H	5.62574000	11.78186400	-0.23714200
H	-13.07072100	-0.84577200	-1.07158600	H	8.06000000	9.67600600	2.59554500
H	-14.15690300	1.06094000	0.07674300	H	7.48170700	11.77643100	1.40913100
C	-3.62218600	-9.00237000	-1.18907000	C	9.58788500	-2.63790500	0.47742000
C	-3.24567700	-10.17196200	-0.52452200	C	10.06478000	-3.63022500	-0.38648400
C	-4.44854900	-9.09361000	-2.31164800	C	10.45334700	-2.02970700	1.39350300
C	-3.68427800	-11.40667000	-0.98033800	C	11.39482800	-4.00939000	-0.32473600
H	-2.60944600	-10.10628100	0.35029800	H	9.39688200	-4.07688500	-1.11263000
C	-4.89736900	-10.33174100	-2.74869900	C	11.78179000	-2.41707700	1.44083100
H	-4.73543500	-8.19172400	-2.83943200	H	10.07222600	-1.27801500	2.07375300
C	-4.51651400	-11.49522400	-2.09027600	C	12.25759000	-3.40634400	0.58574200
H	-3.38290700	-12.30491700	-0.45272200	H	11.76372500	-4.77014500	-1.00244500
H	-5.53883800	-10.38524400	-3.62146800	H	12.44688200	-1.95219400	2.15883200
H	-4.86397900	-12.46081500	-2.43912900	H	13.29799900	-3.70592500	0.62768000
C	7.50486000	-6.40134500	1.99885200	C	0.50525900	-9.81631600	-1.68833400
C	8.88133400	-6.20061500	1.80246500	C	-0.45114700	-10.82005800	-1.45663800
C	7.12182700	-7.45250300	2.84839400	C	1.53202700	-10.11022100	-2.60209500
C	9.82750300	-7.01671500	2.40653900	C	-0.37602100	-12.05632600	-2.08279100
H	9.21062200	-5.39559100	1.15391600	H	-1.26207500	-10.62559300	-0.76244800
C	8.06242600	-8.25883200	3.47412600	C	1.60184500	-11.33617500	-3.24899200
H	6.06721600	-7.62761000	3.03248600	H	2.27744300	-9.35365000	-2.82328400
C	9.41796100	-8.04552000	3.24790600	C	0.65058700	-12.31582100	-2.98451200
H	10.88405800	-6.84917900	2.22750000	H	-1.12064600	-12.81698000	-1.87405200
H	7.74108000	-9.05551500	4.13629500	H	2.39750300	-11.53014400	-3.96040300
H	10.15511400	-8.67956200	3.72899600	H	0.70674800	-13.27749800	-3.48329100
C	8.53349000	5.08736500	-1.97699900	C	-9.28896800	-3.53794400	2.04355200
C	9.85709100	4.66295300	-1.77321500	C	-9.77459900	-4.85015600	1.91787100
C	8.32941200	6.15838200	-2.86276900	C	-10.00059600	-2.66567100	2.88380300
C	10.92463700	5.28648000	-2.40387300	C	-10.92188800	-5.26626600	2.57879900
H	10.04842700	3.83549600	-1.09804100	H	-9.24563000	-5.55028900	1.27970400
C	9.38967500	6.77192500	-3.51538100	C	-11.13543300	-3.08092600	3.56664400
H	7.31876800	6.50277000	-3.05442000	H	-9.64546000	-1.64918500	3.01547000
C	10.69072400	6.34044200	-3.28051100	C	-11.60193200	-4.38147300	3.40875100
H	11.93853900	4.94872600	-2.21863300	H	-11.28407200	-6.28095500	2.45351600
H	9.20426400	7.58787100	-4.20551100	H	-11.65887500	-2.39162200	4.22036100
H	11.52195800	6.82378800	-3.78274200	H	-12.49352900	-4.70615200	3.93458500
C	-2.20573600	9.78000100	1.24379900	C	-6.42795300	7.69070000	-1.61134900
C	-1.49924100	10.88755000	0.74681500	C	-7.74739300	7.78629200	-1.13914100
C	-3.18343400	10.02620900	2.22162300	C	-6.01845300	8.63417700	-2.56800100
C	-1.76686200	12.17665800	1.18661900	C	-8.60867800	8.78004000	-1.58383400
H	-0.73501200	10.73206700	-0.00762800	H	-8.09692400	7.07353300	-0.39936200
C	-3.43816700	11.30935100	2.68622800	C	-6.88101000	9.61619600	-3.03582800
H	-3.74059800	9.19271500	2.63620700	H	-5.00856900	8.58437100	-2.96141500
C	-2.73446300	12.38843700	2.16267200	C	-8.17738200	9.69453900	-2.53863800
H	-1.21763300	13.01643400	0.77479300	H	-9.61817500	8.83998100	-1.19137300
H	-4.18771400	11.47031700	3.45354000	H	-6.54286300	10.32365200	-3.78536400
H	-2.93836500	13.39339900	2.51659200	H	-8.85097500	10.46636000	-2.89566800
N	-3.15993800	-7.74993500	-0.73056600	N	8.23988400	-2.24112000	0.41980500

Structure S34. The coordinates of both the optimized structures of B3N2L$_2$ at S$_0$ and S$_1$ in toluene.

S_0-optimized structure				S_1-optimized structure			
B	-5.36287500	6.51176400	1.23347200	B	-8.55246500	1.44837600	0.65673000
C	-3.83528900	6.76006400	1.00155600	C	-7.51948500	2.60693400	0.52797000
C	-3.28692400	8.01978600	0.71027100	C	-7.87201900	3.95347400	0.32285800
C	-2.93616400	5.68531000	1.08812300	C	-6.14441200	2.33402900	0.63742300
C	-1.92682500	8.19342900	0.49826000	C	-6.91954300	4.95619000	0.23257800
H	-3.94306400	8.88071300	0.63394500	H	-8.91941900	4.21854500	0.22165300
C	-1.57433300	5.85710600	0.91397900	C	-5.19062800	3.33125800	0.56911800
H	-3.31556600	4.69165800	1.29974500	H	-5.81731800	1.31114700	0.78769800
C	-1.04356500	7.11380900	0.60531700	C	-5.55385300	4.66918000	0.36553400
H	-1.54000200	9.18135400	0.27165600	H	-7.23784400	5.98256600	0.08482700
H	-0.91366100	4.99951500	0.97583700	H	-4.14136400	3.06914600	0.64136700
C	0.40767700	7.25243300	0.37020800	C	-4.51179300	5.70717500	0.28343800
C	1.31887300	6.49993500	1.12024000	C	-3.31679100	5.56344100	1.00015000
C	0.91095300	8.07919700	-0.63832700	C	-4.64547900	6.83890400	-0.53069600
C	2.66910600	6.51588000	0.81811500	C	-2.28520000	6.47240000	0.85633200
H	0.95937300	5.88979600	1.94159500	H	-3.19976600	4.73147700	1.68608200
C	2.27055300	8.11176700	-0.91588700	C	-3.61363100	7.75919400	-0.64829100
H	0.22598100	8.66543300	-1.24142600	H	-5.55040700	6.97637700	-1.11361800
C	3.18538400	7.30320700	-0.22382600	C	-2.38372400	7.59161600	0.01082300
H	3.34271800	5.89549500	1.39979000	H	-1.36915100	6.31595300	1.41601800
H	2.62539900	8.74415700	-1.72314800	H	-3.74851000	8.61315600	-1.30490700
B	4.68354500	7.19216800	-0.67013000	B	-1.15122100	8.54879200	-0.19188500
C	-5.95375500	5.13003000	0.79144900	C	-8.07915900	-0.00299400	0.28021300
C	-5.56694000	4.52709400	-0.41555300	C	-7.31931000	-0.25400700	-0.87195400
C	-6.82549800	4.40091200	1.61425500	C	-8.30678100	-1.08753000	1.13960900
C	-6.01776000	3.26880400	-0.77901700	C	-6.79453700	-1.50811800	-1.14000400
H	-4.88697400	5.05265700	-1.07811300	H	-7.11611500	0.55829500	-1.56198600
C	-7.23640400	3.11768000	1.28273400	C	-7.74011400	-2.33268200	0.90641300
H	-7.16423300	4.83659800	2.54877800	H	-8.91150200	-0.94315400	2.02909200
C	-6.83476300	2.52754400	0.08084100	C	-6.96416100	-2.55891000	-0.23326500
H	-5.68680500	2.82598100	-1.71249300	H	-6.19021300	-1.65989300	-2.02817300
H	-7.88781900	2.56874500	1.95479700	H	-7.90737300	-3.14194600	1.60978400
C	-7.19854300	1.13608200	-0.25667800	C	-6.26472300	-3.84215500	-0.44819000
C	-7.08362200	0.12337400	0.69999300	C	-5.58660200	-4.45745000	0.60888900
C	-7.60098200	0.77840000	-1.54705700	C	-6.19839800	-4.43724700	-1.71265700
C	-7.32510900	-1.20058200	0.37225600	C	-4.82896000	-5.59830500	0.40314800
H	-6.76661800	0.37334400	1.70650300	H	-5.62097300	-4.01272500	1.59678300
C	-7.84854800	-0.54414600	-1.88099600	C	-5.45154600	-5.58511300	-1.92605100
H	-7.71598600	1.54765500	-2.30298700	H	-6.73543000	-3.99026600	-2.54148900
C	-7.69225900	-1.55048800	-0.92709900	C	-4.74240400	-6.15441000	-0.87121800
H	-7.20660400	-1.97892000	1.11728300	H	-4.28423100	-6.05211500	1.22308200
H	-8.14692700	-0.80612100	-2.88962700	H	-5.39590100	-6.02963100	-2.91298200
C	-6.74941700	-3.77123800	-1.05971600	C	-2.55170100	-7.17043200	-0.83670500
C	-6.91321700	-5.07146200	-0.57305900	C	-1.75913000	-8.29307200	-0.51701600
C	-5.45421100	-3.31272200	-1.31126400	C	-1.92779500	-5.90663500	-0.87588900
C	-5.81109700	-5.88052000	-0.34410900	C	-0.41929700	-8.14743600	-0.24383000
H	-7.91013000	-5.44686800	-0.37365700	H	-2.21406300	-9.27370600	-0.46651500
C	-4.36025900	-4.12351900	-1.06725100	C	-0.58398400	-5.78162300	-0.62731500
H	-5.30705900	-2.30683100	-1.68584500	H	-2.51105200	-5.02892300	-1.11980200
C	-4.50956800	-5.42471100	-0.57808100	C	0.21881600	-6.89183300	-0.29773200
H	-5.96740900	-6.88950900	0.02221300	H	0.15690200	-9.03284300	-0.00324700
H	-3.36540700	-3.72847400	-1.24043800	H	-0.14663400	-4.79168700	-0.66017600
C	-3.31916000	-6.24826500	-0.29209100	C	1.63094300	-6.72657100	-0.01488900
C	-2.18881400	-6.18236100	-1.11446100	C	2.35435300	-5.63560400	-0.54299900
C	-3.25177800	-7.07355300	0.83444000	C	2.34108500	-7.62415400	0.81135000
C	-1.03122600	-6.87414500	-0.80564400	C	3.67579700	-5.43069800	-0.24377000
H	-2.22270000	-5.59093800	-2.02283200	H	1.87658700	-4.96196800	-1.24287900
C	-2.08954700	-7.75471000	1.16233700	C	3.66263400	-7.42581200	1.13018900
H	-4.10402000	-7.13743700	1.50240300	H	1.82200600	-8.45783500	1.26841700
C	-0.95458900	-7.64313500	0.35766100	C	4.36095800	-6.30734100	0.62570400
H	-0.16837400	-6.80695100	-1.45791500	H	4.20767500	-4.60519700	-0.69739700
H	-2.04790800	-8.35041800	2.06673100	H	4.15715400	-8.10141400	1.81585400
C	5.35909300	5.78063000	-0.63572900	C	0.25285800	7.91447500	-0.27827900
C	6.73738600	5.58874400	-0.44228700	C	1.45370900	8.58098300	0.06683900
C	4.58339700	4.62015000	-0.78978900	C	0.41830800	6.57350300	-0.70085500
C	7.30145700	4.32264900	-0.38840900	C	2.68482800	7.95812100	0.04490500
H	7.38194000	6.45265200	-0.31828100	H	1.40224900	9.60965800	0.41006800
C	5.13977300	3.35490100	-0.74677500	C	1.64358600	5.95229400	-0.74996700
H	3.51458300	4.71231600	-0.94562700	H	-0.45406200	6.01637500	-1.02535900
C	6.51206300	3.17631700	-0.53886100	C	2.82870600	6.60906300	-0.35012200
H	8.37203200	4.22348100	-0.24585300	H	3.55436100	8.51051400	0.38426300
H	4.49248500	2.49074600	-0.83996700	H	1.69515600	4.93942800	-1.13152000
C	7.07456700	1.81218000	-0.46025400	C	4.10170800	5.90963100	-0.33173600
C	6.46232900	0.75553100	-1.14452300	C	4.15467300	4.49779600	-0.33581600
C	8.19338000	1.51738400	0.32680800	C	5.34945200	6.57302300	-0.29780700
C	6.90581100	-0.54514900	-0.98772800	C	5.34684100	3.81542300	-0.31447800
H	5.62404600	0.95362800	-1.80225500	H	3.23333300	3.92754300	-0.32526400
C	8.66724900	0.21687500	0.43506400	C	6.54154500	5.88050400	-0.29329100
H	8.67936900	2.30791800	0.88791000	H	5.37775300	7.65739600	-0.29966800
C	8.01957300	-0.85675300	-0.19307900	C	6.60836800	4.46270000	-0.30157700
H	6.37911200	-1.34325600	-1.50018000	H	5.31500200	2.73151800	-0.32368100
H	9.53509100	0.02539500	1.05821400	H	7.46571000	6.44882800	-0.26307400
B	8.43674100	-2.35586500	-0.00290200	B	7.91276700	3.64819000	-0.32197200
C	1.44732400	-7.50707400	0.70249600	C	6.15577800	-4.72648500	0.96708800
C	2.68933900	-8.09973300	0.44760300	C	7.39385300	-4.42501400	0.39432000
C	1.41246800	-6.12553000	0.91886200	C	5.38654700	-3.69643400	1.51475900
C	3.84329700	-7.33396800	0.40594300	C	7.81958800	-3.11141600	0.32273700
H	2.75096400	-9.16835700	0.28121400	H	8.00734700	-5.21912500	-0.01458500
C	2.56674300	-5.36945400	0.84701200	C	5.81770900	-2.38876700	1.42657700
H	0.46888900	-5.63700900	1.12617900	H	4.44185800	-3.92534800	1.99347100
C	3.81504900	-5.94741800	0.59115600	C	7.03244200	-2.05448700	0.80798500
H	4.78790400	-7.83495400	0.22588200	H	8.78126800	-2.89981200	-0.12871700
H	2.48471300	-4.29687200	0.97724300	H	5.18042800	-1.60498900	1.81639100
C	5.02521400	-5.10696500	0.49791200	C	7.40891000	-0.64449200	0.62697800

C	5.09301000	-3.88056200	1.17122900
C	6.12286800	-5.47171300	-0.29152400
C	6.17020300	-3.03057600	0.99983100
H	4.28757100	-3.58407200	1.83298100
C	7.22834500	-4.64106200	-0.40985800
H	6.09995400	-6.40277300	-0.84713700
C	7.27344800	-3.38116200	0.20570100
H	6.16315900	-2.07007600	1.50443100
H	8.05714700	-4.95908300	-1.03456000
C	9.93110200	-2.79775400	-0.02541500
C	10.91301600	-2.02070000	-0.66226800
C	10.35015900	-3.98780600	0.59237700
C	12.24324800	-2.41504900	-0.69197200
H	10.62124200	-1.09823300	-1.15314200
C	11.68161800	-4.37909900	0.58416300
H	9.61727400	-4.60690000	1.09887400
C	12.62963400	-3.59405700	-0.06344400
H	12.98085700	-1.80398600	-1.20083300
H	11.98231800	-5.29610400	1.07921800
H	13.66997800	-3.90111300	-0.07806100
C	0.24681100	-9.58737300	1.20606400
C	-0.53878000	-10.55088600	0.56861400
C	0.99485800	-9.95401900	2.32768800
C	-0.57619700	-11.85188600	1.04865900
H	-1.11764300	-10.27288200	-0.30437300
C	0.96511700	-11.26228300	2.78919300
H	1.59656600	-9.21003400	2.83614100
C	0.17840200	-12.21825000	2.15717200
H	-1.19127800	-12.58726500	0.54172500
H	1.55188700	-11.53018800	3.66102200
H	0.15284900	-13.23742600	2.52516400
C	-9.02106400	-3.35378000	-1.90807000
C	-10.24689200	-2.74324700	-1.62014400
C	-8.99084600	-4.39239300	-2.84513200
C	-11.40563600	-3.16025700	-2.25789700
H	-10.28662500	-1.94218100	-0.89175500
C	-10.15976100	-4.81203100	-3.46316900
H	-8.04863400	-4.86660500	-3.09140000
C	-11.37499700	-4.20011900	-3.17964600
H	-12.34431600	-2.67217700	-2.01893400
H	-10.11280600	-5.61894300	-4.18651800
H	-12.28400200	-4.52794200	-3.67039800
C	-6.26744600	7.57958100	1.92506100
C	-7.64773200	7.63203400	1.66916000
C	-5.74363200	8.51930700	2.82823100
C	-8.46160200	8.58341200	2.26818800
H	-8.08441300	6.91913400	0.97760400
C	-6.55490800	9.45864700	3.44961100
H	-4.68363600	8.49986000	3.05811200
C	-7.91548000	9.49595500	3.16428900
H	-9.52215400	8.61153200	2.04267600
H	-6.12820000	10.16428700	4.15418900
H	-8.55038800	10.23472700	3.64171100
C	5.45441000	8.45182900	-1.17936200
C	5.15918600	9.73015100	-0.67774100
C	6.46042200	8.35979600	-2.15515500
C	5.84532600	10.85650600	-1.11061500
H	4.38508000	9.83694600	0.07509700
C	7.13277100	9.48488400	-2.61254100
H	6.70621200	7.38944700	-2.57352800
C	6.83085600	10.73526000	-2.08417800
H	5.60869600	11.83014700	-0.69527700
H	7.89509000	9.38830000	-3.37810100
H	7.36168800	11.61486400	-2.43249000
N	0.26264200	-8.26154800	0.72345700
N	-7.85038900	-2.91607000	-1.26896300
C	6.91517600	0.35000100	1.48291600
C	8.19270200	-0.23156400	-0.45918700
C	7.12013600	1.68988200	1.21001400
H	6.35950400	0.07102300	2.37160800
C	8.41448800	1.11400000	-0.70750300
H	8.58379800	-0.96676000	-1.15450600
C	7.84282100	2.12394500	0.08420300
H	6.69173600	2.43050100	1.87725600
H	9.00221000	1.39459100	-1.57588100
C	9.29020100	4.23840700	-0.78782500
C	9.38998900	5.25061000	-1.75942300
C	10.50170300	3.75099500	-0.26475800
C	10.61625200	5.75215500	-2.17451000
H	8.48281300	5.63711900	-2.21169100
C	11.73209700	4.25505800	-0.66373100
H	10.47164300	2.96288300	0.48135400
C	11.79499500	5.26078300	-1.62265000
H	10.65430800	6.52710800	-2.93342300
H	12.64558500	3.86288900	-0.22801100
H	12.75443500	5.65444600	-1.94144200
C	6.54285400	-7.09729000	1.36671900
C	6.59986500	-8.27939100	0.62535900
C	7.36862900	-6.92672700	2.47923300
C	7.46846400	-9.28907000	1.01039000
H	5.97991100	-8.39314100	-0.25600900
C	8.23669100	-7.94125200	2.85040100
H	7.31728600	-6.00735600	3.04978700
C	8.28815300	-9.12492600	2.12160300
H	7.51520100	-10.20231700	0.42881400
H	8.87108100	-7.80824500	3.71874500
H	8.96897200	-9.91490000	2.41567300
C	-4.48306900	-8.46413700	-1.60690800
C	-5.72241300	-8.88991700	-1.12603700
C	-3.84783500	-9.18511700	-2.62085700
C	-6.31061600	-10.03160000	-1.64856100
H	-6.21584700	-8.32727400	-0.34271100
C	-4.44077700	-10.33149800	-3.12802900
H	-2.90169100	-8.83675600	-3.01782300
C	-5.67254500	-10.75999700	-2.64621600
H	-7.27142200	-10.35702000	-1.26683100
H	-3.94340700	-10.88206500	-3.91827700
H	-6.13563300	-11.65225700	-3.05075700
C	-9.99817200	1.67567500	1.20378700
C	-11.05648400	0.84091400	0.80787500
C	-10.29574300	2.70124200	2.11643400
C	-12.34865200	1.03279200	1.27679900
H	-10.85989700	0.03374200	0.10967700
C	-11.58060700	2.88440000	2.60913800
H	-9.49980800	3.35410800	2.45822100
C	-12.61177200	2.05434900	2.18307900
H	-13.15061800	0.38337000	0.94247800
H	-11.78017400	3.67579000	3.32353900
H	-13.61846800	2.20098600	2.55976800
C	-1.38234200	10.09673400	-0.30183700
C	-2.45824000	10.72537600	0.34911800
C	-0.54048900	10.92188800	-1.06804400
C	-2.67338000	12.09387200	0.25755500
H	-3.13361600	10.12329100	0.94878800
C	-0.75571300	12.28918700	-1.17746800
H	0.29116700	10.47347100	-1.60159200
C	-1.82197700	12.88224200	-0.50959500
H	-3.50722200	12.54817800	0.78305100
H	-0.09172500	12.89490800	-1.78574800
H	-1.99029600	13.95114900	-0.58910600
N	5.66793800	-6.05436800	0.97922900
N	-3.90194300	-7.28280900	-1.09112000

Structure S35. The coordinates of both the optimized structures of B2N3L_2 at S_0 and S_1 in toluene.

S_0-optimized structure				S_1-optimized structure			
C	5.69387200	5.22874800	-1.00316600	C	-7.70601800	1.72574100	0.92433800
C	5.56413600	6.56831700	-0.62124000	C	-8.23180100	2.91792200	0.42212200
C	4.53004800	4.48215900	-1.20778600	C	-6.35758900	1.68826000	1.28203100
C	4.31052000	7.13241000	-0.44640900	C	-7.42233100	4.03418300	0.27387400
H	6.45189500	7.16778000	-0.45823400	H	-9.27918800	2.96739900	0.14717900
C	3.28468200	5.05201100	-1.01785900	C	-5.55426800	2.80095100	1.11172400
H	4.60543200	3.44292700	-1.50340800	H	-5.93412300	0.77223100	1.67674600
C	3.14074200	6.38851800	-0.63282200	C	-6.06234400	4.00093100	0.60237400
H	4.24242800	8.17652400	-0.16072800	H	-7.85832200	4.95125400	-0.10694300
H	2.40367500	4.43473400	-1.15193000	H	-4.50132000	2.72982200	1.35893500
C	1.79649200	6.95078900	-0.39893000	C	-5.16097900	5.14954700	0.38719700
C	0.70377000	6.52396900	-1.16385200	C	-4.07631500	5.37464700	1.24309700
C	1.55546400	7.87498100	0.62305500	C	-5.30915800	6.00615800	-0.70871400
C	-0.58106600	6.93936400	-0.86228800	C	-3.13963200	6.35506200	0.96435000
H	0.86553100	5.84984900	-1.99783400	H	-3.96319800	4.76745000	2.13522500
C	0.26840400	8.31664900	0.89475700	C	-4.37882500	7.00393900	-0.96464600
H	2.37873700	8.21649000	1.24133500	H	-6.13409300	5.85703800	-1.39804200
C	-0.84419000	7.83285600	0.18886900	C	-3.23744300	7.18220300	-0.16698700
H	-1.40622400	6.55870600	-1.45517000	H	-2.29638900	6.47846700	1.63624000
H	0.11638400	9.01645000	1.71015500	H	-4.51428300	7.63200900	-1.83980500
B	-2.30650500	8.17462300	0.63092500	B	-2.08364500	8.17839400	-0.56942400
C	7.08355800	3.25753800	-0.80162900	C	-7.91975200	-0.66975100	0.69484100
C	6.71869300	2.83118600	0.47562200	C	-7.26326100	-0.79656700	-0.53230500
C	7.52890700	2.31344400	-1.72717900	C	-7.93512500	-1.76387500	1.56422200
C	6.75695100	1.48607600	0.80351100	C	-6.60372300	-1.96789600	-0.85908200
H	6.37656600	3.56020600	1.20111200	H	-7.25143800	0.04368300	-1.21638300
C	7.56893300	0.96936900	-1.39001600	C	-7.26725500	-2.93116900	1.23179400
H	7.82882200	2.63631500	-2.71752900	H	-8.45273400	-1.68585200	2.51301200
C	7.16581300	0.52546900	-0.12672400	C	-6.57317900	-3.05188000	0.02391500
H	6.43262100	1.17137100	1.78937100	H	-6.07139200	-2.02936700	-1.80198100
H	7.91320300	0.24951700	-2.12461700	H	-7.27993900	-3.76258300	1.92857600
C	7.10780200	-0.91325500	0.20343300	C	-5.76722500	-4.24668300	-0.29064100
C	6.59297600	-1.83120600	-0.71707900	C	-4.95821500	-4.83250300	0.68909000
C	7.50498500	-1.39613400	1.45418400	C	-5.73643500	-4.78915600	-1.58069100
C	6.45267200	-3.17019900	-0.39339200	C	-4.11798600	-5.88969800	0.38475700
H	6.26840400	-1.48545300	-1.69239000	H	-4.96067400	-4.42893900	1.69519000
C	7.36364700	-2.73439200	1.78742600	C	-4.90595400	-5.85330300	-1.89282500
H	7.92681700	-0.71153600	2.18213500	H	-6.37190700	-4.36854300	-2.35183400
C	6.81864700	-3.63453500	0.87057600	C	-4.07365100	-6.39319500	-0.91437600
H	6.03288500	-3.86376300	-1.11275300	H	-3.47730200	-6.31863200	1.14674900
H	7.66371800	-3.08613900	2.76779100	H	-4.88342700	-6.25533800	-2.89909300
C	5.29557300	-5.50675000	1.03952200	C	-1.81070400	-7.23137100	-1.02803300
C	5.08331500	-6.79741200	0.54651300	C	-0.92711700	-8.30167700	-0.77989700
C	4.18280900	-4.71455000	1.33185200	C	-1.28296500	-5.92500200	-1.04513100
C	3.79596000	-7.27039600	0.34519200	C	0.40788500	-8.06606300	-0.54424700
H	5.93320700	-7.42997800	0.31852200	H	-1.30616100	-9.31513600	-0.75484800
C	2.90242900	-5.19093100	1.11424700	C	0.05610400	-5.70812200	-0.83319500
H	4.32712300	-3.71145600	1.71494100	H	-1.93759400	-5.08599600	-1.24012300
C	2.67589300	-6.47682200	0.61370400	C	0.94767600	-6.76529300	-0.56655800
H	3.66051400	-8.27984500	-0.02803300	H	1.05638300	-8.91385000	-0.35752500
H	2.05982700	-4.53935100	1.31711600	H	0.41961600	-4.68812500	-0.84532900
C	1.29941200	-6.93611500	0.34563100	C	2.35015500	-6.49890400	-0.30498600
C	0.24212000	-6.56585300	1.18446600	C	2.97503700	-5.34121100	-0.81378900
C	0.99125000	-7.70003300	-0.78426900	C	3.14003100	-7.35512900	0.49033000
C	-1.06611400	-6.90403400	0.88703600	C	4.27604900	-5.03085100	-0.51255500
H	0.44982800	-6.01584100	2.09575000	H	2.43584800	-4.69598800	-1.49577800
C	-0.31840500	-8.02800400	-1.09942700	C	4.44265300	-7.05197600	0.80929000
H	1.78355700	-7.99154000	-1.46519800	H	2.69576700	-8.24142700	0.92720200
C	-1.36739900	-7.61068500	-0.27908500	C	5.03531000	-5.86262000	0.33706100
H	-1.86916500	-6.60351300	1.54983100	H	4.73395600	-4.15143700	-0.94588600
H	-0.53546600	-8.58005600	-2.00647600	H	4.99829000	-7.69965800	1.47493400
C	-3.37845700	7.03458300	0.58138500	C	-0.62396700	7.69925900	-0.46726800
C	-4.75034200	7.26978400	0.39053800	C	0.50214800	8.56001300	-0.44524600
C	-2.98985900	5.69314900	0.72813500	C	-0.31075200	6.31966900	-0.40081500
C	-5.67165900	6.23424700	0.33506000	C	1.79733400	8.09540400	-0.36329900
H	-5.10317100	8.28896200	0.27193100	H	0.34275300	9.63333700	-0.47648800
C	-3.90365500	4.65609300	0.68477100	C	0.97724600	5.84662400	-0.32895200
H	-1.94279300	5.45730300	0.88037900	H	-1.11986000	5.59799000	-0.42285200
C	-5.26623000	4.90251300	0.48306600	C	2.09475400	6.71303400	-0.30884000
H	-6.72246000	6.46414300	0.19624900	H	2.60600100	8.81764700	-0.35943400
H	-3.54933800	3.63592500	0.77566700	H	1.12493400	4.77576100	-0.26421900
C	-6.21609700	3.77292400	0.41310100	C	3.44825600	6.19515900	-0.23480700
C	-5.94912400	2.58483600	1.10340100	C	3.72145400	4.82128200	-0.43315900
C	-7.37585800	3.82630600	-0.36795000	C	4.57351400	7.00632900	0.04764000
C	-6.76706500	1.47912000	0.95875000	C	4.98997200	4.30784100	-0.32489800
H	-5.08619600	2.52420300	1.75626000	H	2.91927200	4.14737600	-0.70813200
C	-8.22184400	2.72965200	-0.46521400	C	5.84713800	6.48703200	0.12869900
H	-7.60242700	4.72391500	-0.93274000	H	4.43452400	8.06420300	0.24140700
C	-7.92698900	1.51436100	0.16968700	C	6.12832800	5.10542400	-0.03872200
H	-6.50442900	0.56252700	1.47626500	H	5.12805500	3.24666200	-0.50231300
H	-9.10958500	2.80592200	-1.08507400	H	6.66182500	7.16116900	0.37443100
B	-8.77745500	0.20939400	-0.00748800	B	7.52478900	4.47096100	0.05531000
C	-3.62547300	-6.78685300	-0.62307000	C	6.64596900	-4.12229200	0.78468900
C	-4.98586600	-6.98469000	-0.36043800	C	7.86214900	-3.64863900	0.28467300
C	-3.18634400	-5.47973200	-0.85740800	C	5.74277800	-3.21593500	1.34763600
C	-5.86367200	-5.91296800	-0.33091900	C	8.13633500	-2.29371100	0.30249900
H	-5.35825600	-7.98564300	-0.17896100	H	8.57887100	-4.34284200	-0.13782800
C	-4.06717100	-4.41688000	-0.79792300	C	6.02160600	-1.86574200	1.34360000
H	-2.14139500	-5.29318400	-1.07008000	H	4.81316500	-3.57524600	1.77237800
C	-5.42964700	-4.59882100	-0.53693500	C	7.21467300	-1.35875300	0.80368900
H	-6.91320600	-6.11143800	-0.14430800	H	9.08520500	-1.95259200	-0.09290300
H	-3.67404400	-3.41761200	-0.94273500	H	5.27891900	-1.18648300	1.74161000
C	-6.33864500	-3.43788900	-0.46093300	C	7.43570900	0.09224300	0.71967900
C	-6.04286900	-2.25768700	-1.15501400	C	6.73707600	0.97756500	1.55360200

C	-7.49334700	-3.44897900	0.33110600	C	8.27780600	0.65398100	-0.25107400
C	-6.82007300	-1.12458700	-1.00043100	C	6.80758700	2.34404500	1.36095900
H	-5.18686000	-2.22323000	-1.81887600	H	6.12556200	0.59446400	2.36275500
C	-8.30337600	-2.32652300	0.43325800	C	8.36725500	2.02787200	-0.41242100
H	-7.74552300	-4.33586500	0.90197400	H	8.82805000	0.00996100	-0.92850000
C	-7.97504300	-1.11989000	-0.20253700	C	7.59692700	2.92125500	0.35013900
H	-6.53029200	-0.21743600	-1.52037200	H	6.22067100	2.99322100	2.00227300
H	-9.18732400	-2.37461900	1.06146100	H	9.01179400	2.42062100	-1.19273800
C	-10.33538800	0.23707800	0.01591600	C	8.85514600	5.26814900	-0.16729800
C	-11.03819600	1.28229000	0.63812600	C	8.92391900	6.40135000	-0.99873700
C	-11.09292500	-0.78147900	-0.58597400	C	10.05933500	4.86289100	0.43752500
C	-12.42538600	1.30613100	0.66850700	C	10.11086900	7.09059300	-1.20524300
H	-10.48241600	2.08149300	1.11707800	H	8.02601300	6.73358300	-1.50906300
C	-12.48038600	-0.75496700	-0.57668200	C	11.24892700	5.55163700	0.24568900
H	-10.58008200	-1.59941600	-1.08087100	H	10.05415400	3.98962200	1.08270100
C	-13.14841400	0.28810700	0.05590100	C	11.28052700	6.67188600	-0.57873300
H	-12.94499600	2.11791000	1.16590100	H	10.12635300	7.95579300	-1.86041400
H	-13.04296700	-1.54696000	-1.05901300	H	12.15559200	5.21491300	0.73840500
H	-14.23299000	0.30774600	0.07121300	H	12.20889900	7.21096500	-0.73543300
C	-3.08173500	-9.13057700	-1.11302000	C	7.28914400	-6.45794500	1.04692600
C	-2.58818600	-10.27789600	-0.48648000	C	7.45454600	-7.59622500	0.25438700
C	-3.92202400	-9.27032000	-2.22068300	C	8.10484300	-6.25732400	2.16228000
C	-2.92683600	-11.53588200	-0.96293600	C	8.42120900	-8.53163100	0.58964100
H	-1.93923300	-10.17615100	0.37542500	H	6.84025300	-7.73173900	-0.62786500
C	-4.26978900	-10.53331900	-2.67818000	C	9.07219000	-7.19653000	2.48273500
H	-4.29883200	-8.38675700	-2.72205100	H	7.96863000	-5.37424100	2.77459500
C	-3.77334300	-11.67338900	-2.05698400	C	9.23247600	-8.33651500	1.70209100
H	-2.53460600	-12.41534200	-0.46412800	H	8.55119900	-9.40971200	-0.03210300
H	-4.92343400	-10.62330500	-3.53888200	H	9.69858600	-7.03985000	3.35299300
H	-4.04231800	-12.65786800	-2.42203900	H	9.99053500	-9.06761600	1.95688200
C	7.61977000	-5.75054800	1.79992100	C	-3.66993800	-8.63249700	-1.78838000
C	8.95191600	-5.53931200	1.42848900	C	-4.84803000	-9.18121900	-1.27896800
C	7.33539000	-6.72369900	2.76376800	C	-3.02500100	-9.25025400	-2.86246700
C	9.96868500	-6.27982300	2.01281900	C	-5.36651600	-10.34083100	-1.83512100
H	9.18519200	-4.79394500	0.67759200	H	-5.34853300	-8.69879200	-0.44803400
C	8.35938700	-7.47023200	3.32815600	C	-3.54660400	-10.41605900	-3.40281900
H	6.31027900	-6.88985200	3.07211400	H	-2.12746400	-8.80774700	-3.27808800
C	9.68271200	-7.25381600	2.96234400	C	-4.71770300	-10.96616000	-2.89386600
H	10.99444700	-6.09962900	1.70995500	H	-6.28007800	-10.76192600	-1.43149100
H	8.11679100	-8.21943500	4.07397200	H	-3.04225600	-10.88691700	-4.23887800
H	10.47960100	-7.83517600	3.41135400	H	-5.12566200	-11.87322900	-3.32410300
C	8.03414100	5.29380800	-1.75677900	C	-9.76313200	0.60997200	1.66942000
C	9.34323000	5.01175500	-1.35344500	C	-10.76769100	-0.28381200	1.28294300
C	7.82821800	6.23464200	-2.77076900	C	-10.04059000	1.54586300	2.67056400
C	10.41571600	5.65369200	-1.95460100	C	-12.01249300	-0.24494800	1.89285600
H	9.51415700	4.28823200	-0.56517900	H	-10.56886200	-1.00418600	0.49820000
C	8.90776800	6.88333700	-3.35258000	C	-11.29545100	1.58669100	3.26095500
H	6.82087900	6.45227400	-3.10464300	H	-9.26963500	2.23867500	2.98518700
C	10.20816600	6.59752400	-2.95368300	C	-12.28898200	0.69164800	2.88234000
H	11.42296000	5.42037800	-1.62701000	H	-12.77799400	-0.94575200	1.57780900
H	8.72679600	7.60934600	-4.13777200	H	-11.49055900	2.32059600	4.03527800
H	11.04831600	7.10239000	-3.41619500	H	-13.26595100	0.72485200	3.35035700
C	-2.66246900	9.60224000	1.15746100	C	-2.47085600	9.60019000	-1.11422900
C	-1.99425700	10.73738900	0.66993600	C	-3.60714600	10.27427600	-0.63498900
C	-3.65045700	9.80729900	2.13438900	C	-1.72694400	10.25061800	-2.11357400
C	-2.30813800	12.01328900	1.11770800	C	-3.96704000	11.53115100	-1.10241700
H	-1.22298500	10.61367100	-0.08322600	H	-4.21541800	9.80069100	0.12941400
C	-3.95168000	11.07751500	2.60669700	C	-2.08877800	11.49984500	-2.60037100
H	-4.17873700	8.95187800	2.54206500	H	-0.85538900	9.75513700	-2.52844800
C	-3.28525400	12.18430600	2.09226100	C	-3.20796500	12.14918900	-2.09049900
H	-1.78767400	12.87468400	0.71316800	H	-4.84346400	12.02881100	-0.69990200
H	-4.70844500	11.20681200	3.37296100	H	-1.49710800	11.96879100	-3.37996600
H	-3.52554700	13.17905800	2.45220200	H	-3.48965800	13.12801000	-2.46429600
N	-2.71404600	-7.85595200	-0.63388000	N	6.31205300	-5.49276500	0.70978400
N	6.59440800	-4.98829700	1.21576700	N	-3.16031200	-7.43453100	-1.23626600
N	6.95444800	4.62325500	-1.15462400	N	-8.50107100	0.56288500	1.05253800

Structure S36. The coordinates of both the optimized structures of B1N4L$_2$ at S_0 and S_1 in toluene.

S_0-optimized structure				S_1-optimized structure			
C	5.45043400	5.38196600	-0.98698400	C	4.89359500	-5.83141200	-0.70990200
C	5.27820600	6.68002200	-0.49746900	C	6.27063200	-5.73838200	-0.42999100
C	4.31366200	4.62848900	-1.28917100	C	4.15051200	-4.63746400	-0.76053600
C	4.00618700	7.19821200	-0.31044500	C	6.86312700	-4.51362000	-0.21475500
H	6.14725100	7.28273800	-0.26094800	H	6.86880500	-6.63877300	-0.37303700
C	3.04866700	5.14997200	-1.08592300	C	4.75628000	-3.42210400	-0.55063100
H	4.42640600	3.62011900	-1.66893300	H	3.09055900	-4.67362100	-0.97399300
C	2.86191900	6.44415800	-0.59044600	C	6.13203400	-3.31108000	-0.27208500
H	3.90235100	8.21218900	0.06053000	H	7.92782800	-4.48831900	-0.01426300
H	2.18585900	4.52788000	-1.29624500	H	4.13757800	-2.53413500	-0.58236600
C	1.49862700	6.94986000	-0.33905200	C	6.74429100	-2.01234500	-0.05316900
C	0.44023600	6.61160300	-1.19001900	C	6.13663300	-0.83433600	-0.53789600
C	1.20124700	7.72395900	0.78679100	C	7.95300800	-1.85707300	0.66068400
C	-0.86075200	6.98666900	-0.90581600	C	6.66669400	0.40670400	-0.30070600
H	0.64198700	6.05594200	-2.09918500	H	5.24657600	-0.90034900	-1.15016100
C	-0.10187500	8.08898700	1.08857400	C	8.50291900	-0.62071900	0.90071900
H	1.99432200	7.99314100	1.47594700	H	8.43974300	-2.72597800	1.08643000
C	-1.15378100	7.69960200	0.25846800	C	7.86294800	0.54938400	0.43583600
H	-1.66558300	6.70852700	-1.57619200	H	6.18713100	1.28710300	-0.70722300
H	-0.31270300	8.64664200	1.99368700	H	9.40337800	-0.53768900	1.49610500
C	6.90026200	3.45384800	-0.81185200	C	2.87212300	-7.15810900	-0.67733300
C	6.52402200	2.99991800	0.45271300	C	2.35650200	-6.85597500	0.58184900
C	7.39759200	2.53579700	-1.73770600	C	2.00438600	-7.52386900	-1.70449000
C	6.60811200	1.65427500	0.76908500	C	0.98820100	-6.87003200	0.79321600
H	6.14058400	3.70794400	1.17831300	H	3.03229100	-6.58157000	1.38369800
C	7.48197800	1.19081600	-1.41234300	C	0.63620900	-7.53523900	-1.48408200
H	7.70505100	2.87954700	-2.71864300	H	2.40465400	-7.77687700	-2.67946500
C	7.07406800	0.71963200	-0.16053300	C	0.09953700	-7.18624400	-0.24006000
H	6.27738900	1.31754400	1.74551700	H	0.59917100	-6.59270200	1.76637600
H	7.86701000	0.49168200	-2.14684500	H	-0.02810700	-7.81189800	-2.29506700
C	7.07378000	-0.72244200	0.16039700	C	-1.35721100	-7.07709000	-0.02732400
C	6.60726000	-1.65688200	-0.76914300	C	-2.17423100	-6.48998800	-0.99884600
C	7.48169300	-1.19380400	1.41213900	C	-1.95297100	-7.47863200	1.17188800
C	6.52263700	-3.00248900	-0.45275600	C	-3.52150900	-6.27278600	-0.76629400
H	6.27652000	-1.32000800	-1.74552000	H	-1.74093000	-6.16377400	-1.93800900
C	7.39677200	-2.53874700	1.73751700	C	-3.29993300	-7.26147300	1.41347600
H	7.86715600	-0.49483800	2.14657600	H	-1.35102000	-7.96012300	1.93491700
C	6.89888600	-3.45658100	0.81174800	C	-4.09551100	-6.63099100	0.45494900
H	6.13876900	-3.71034900	-1.17829100	H	-4.13378300	-5.79101000	-1.51959300
H	7.70424600	-2.88263300	2.71840300	H	-3.73765100	-7.56041200	2.35869300
C	5.44830900	-5.38412700	0.98692600	C	-5.86045800	-4.97292800	0.57350900
C	5.27556500	-6.68210900	0.49739300	C	-7.16716300	-4.65329000	0.19189100
C	4.31183500	-4.63020400	1.28912200	C	-4.96235500	-3.92520900	0.79324900
C	4.00334100	-7.19979700	0.31037700	C	-7.55809600	-3.33082200	0.05286300
H	6.14437100	-7.28516300	0.26085500	H	-7.88350900	-5.44664400	0.01414200
C	3.04663300	-5.15118900	1.08588300	C	-5.35613900	-2.61049900	0.62304500
H	4.42497800	-3.62188100	1.66889000	H	-3.94340200	-4.14320800	1.08821500
C	2.85937200	-6.44529900	0.59040100	C	-6.66418500	-2.27425900	0.25944600
H	3.89910100	-8.21372900	-0.06060900	H	-8.58720300	-3.11858200	-0.21543400
H	2.18407100	-4.52876200	1.29621800	H	-4.62086100	-1.82731100	0.76739100
C	1.49588000	-6.95046600	0.33902300	C	-7.05698600	-0.86115400	0.09983000
C	0.43763100	-6.61179500	1.19000300	C	-6.43747000	0.14016100	0.85607800
C	1.19818300	-7.72445000	-0.78681500	C	-8.02968300	-0.45808100	-0.82198700
C	-0.86350700	-6.98634900	0.90581300	C	-6.74808700	1.47579400	0.68216300
H	0.63961200	-6.05621200	2.09916800	H	-5.71299000	-0.13045800	1.61578300
C	-0.10508500	-8.08896700	-1.08858400	C	-8.34657800	0.87896900	-1.00620700
H	1.99114600	-7.99394700	-1.47597900	H	-8.51670500	-1.19931000	-1.44641300
C	-1.15683000	-7.69916600	-0.25846800	C	-7.69770700	1.86950500	-0.26389600
H	-1.66822100	-6.70789100	1.57619900	H	-6.25209700	2.22948900	1.28211800
H	-0.31614200	-8.64654000	-1.99369400	H	-9.08055400	1.16296200	-1.75137800
C	-3.42709500	6.92062400	0.59525500	C	7.42190600	2.87638500	0.81415900
C	-4.78329800	7.13860800	0.32719200	C	7.57252400	4.04370300	0.05178000
C	-3.00954600	5.60770800	0.83641600	C	6.29069400	2.74184900	1.63242500
C	-5.67763300	6.08043600	0.29885200	C	6.58605500	5.00461000	0.05544900
H	-5.13950200	8.14446800	0.14034900	H	8.44478000	4.16245800	-0.58138600
C	-3.90638800	4.55846000	0.77709100	C	5.31682000	3.71150500	1.63832100
H	-1.96849200	5.40545700	1.05358000	H	6.19432300	1.86875600	2.26856300
C	-5.26496900	4.76032700	0.51077700	C	5.39509200	4.86211300	0.81315600
H	-6.72329300	6.29463400	0.10799500	H	6.70803200	5.86292100	-0.59279800
H	-3.52897700	3.55384600	0.92631300	H	4.47425400	3.58492800	2.30544200
C	-6.19118200	3.61284600	0.43612100	C	4.29490400	5.79743100	0.70814100
C	-5.91214500	2.42895500	1.13070200	C	2.99496900	5.45623000	1.15543000
C	-7.34662400	3.64018200	-0.35447600	C	4.42178800	7.06852000	0.09514400
C	-6.70658100	1.30746400	0.97839900	C	1.91593200	6.27789600	0.93983400
H	-5.05609100	2.38190800	1.79374200	H	2.82294900	4.50330400	1.64235500
C	-8.17389000	2.53000900	-0.45378200	C	3.33705000	7.89422800	-0.09408900
H	-7.58650200	4.53015000	-0.92587000	H	5.39606400	7.41312300	-0.23452000
C	-7.86296300	1.31938500	0.18291400	C	2.01430300	7.53002600	0.27576300
H	-6.42986400	0.39642900	1.49864900	H	0.94399200	5.95144200	1.29397300
H	-9.05869400	2.59113300	-1.07970100	H	3.49578300	8.84844200	-0.58618500
B	-8.68847000	0.00170200	0.00004400	B	0.77380300	8.38394600	-0.06172200
C	-3.42983900	-6.91929000	-0.59525100	C	-6.80770300	4.09252900	-0.52353300
C	-4.78613000	-7.13674100	-0.32720100	C	-6.67335200	5.18078400	0.33906200
C	-3.01177000	-5.60653700	-0.83640100	C	-5.77126900	3.80979800	-1.41314800
C	-5.68004800	-6.07821700	-0.29886100	C	-5.50920300	5.93370200	0.33949400
H	-5.14273200	-8.14246200	-0.14036700	H	-7.47158000	5.41226100	1.03537400
C	-3.90819700	-4.55693600	-0.77707400	C	-4.61374400	4.56633100	-1.40694000
H	-1.97063400	-5.40469600	-1.05355300	H	-5.87883800	2.98314800	-2.10627000
C	-5.26685900	-4.75827000	-0.51077300	C	-4.43882900	5.63255800	-0.51384300
H	-6.72579400	-6.29200000	-0.10801200	H	-5.40897400	6.73818900	1.05925800
H	-3.53039000	-3.55246900	-0.92628300	H	-3.83388600	4.33323300	-2.12234200
C	-6.19262000	-3.61042400	-0.43610900	C	-3.15959800	6.35985200	-0.44494700
C	-5.91310600	-2.42662900	-1.13066300	C	-1.96068300	5.71879100	-0.78647500
C	-7.34807900	-3.63732000	0.35447700	C	-3.06946700	7.68948900	-0.01028800

C	-6.70709900	-1.30482700	-0.97834100	C	-0.74374400	6.36406600	-0.67445200
H	-5.05702600	-2.37990700	-1.79369200	H	-1.97914800	4.68458400	-1.11301400
C	-8.17490500	-2.52682100	0.45380100	C	-1.84426500	8.32885400	0.09503300
H	-7.58831300	-4.52720600	0.92584900	H	-3.97410500	8.23661200	0.23535800
C	-7.86349100	-1.31630700	-0.18286400	C	-0.62624300	7.68918000	-0.21059800
H	-6.43001600	-0.39388900	-1.49856500	H	0.15498300	5.82442400	-0.95275100
H	-9.05973800	-2.58760800	1.07971200	H	-1.82431000	9.35814100	0.43933900
C	-10.24753500	0.00201700	0.00000000	C	0.89538800	9.93447000	-0.29641700
C	-10.97784700	1.03525900	0.61029600	C	1.80736900	10.72804900	0.42405200
C	-10.97822700	-1.03093500	-0.61033100	C	0.09118900	10.60849000	-1.23409300
C	-12.36560600	1.03432800	0.62075100	C	1.91434200	12.09763800	0.22278200
H	-10.44314500	1.84478900	1.09585200	H	2.43758400	10.25482400	1.17055900
C	-12.36598500	-1.02945600	-0.62085000	C	0.19777200	11.97569100	-1.45098300
H	-10.44382200	-1.84067900	-1.09585800	H	-0.62680600	10.03793900	-1.81490900
C	-13.06164200	0.00257300	-0.00006600	C	1.11079500	12.72989300	-0.72073400
H	-12.90662000	1.83751300	1.10926500	H	2.62410000	12.67625000	0.80582800
H	-12.90729400	-1.83242800	-1.10938800	H	-0.43291100	12.45647500	-2.19228700
H	-14.14653000	0.00278800	-0.00009100	H	1.19338300	13.79944900	-0.88341900
C	-2.84569100	-9.25642800	-1.07575300	C	-9.23545800	3.75863400	-0.60681800
C	-2.32040200	-10.39109600	-0.45175600	C	-10.32781400	3.13183300	0.00492600
C	-3.69381500	-9.41783300	-2.17454700	C	-9.45980200	4.92562300	-1.34682400
C	-2.63580500	-11.65747000	-0.92179900	C	-11.60478500	3.65471100	-0.13439400
H	-1.66514500	-10.27281100	0.40324200	H	-10.17099300	2.23745700	0.59557400
C	-4.01786700	-10.68943800	-2.62557800	C	-10.73960900	5.44666500	-1.46487500
H	-4.09524400	-8.54435800	-2.67440200	H	-8.62615400	5.41987500	-1.83072900
C	-3.49001600	-11.81666900	-2.00688300	C	-11.82393800	4.81607200	-0.86606700
H	-2.21894500	-12.52666800	-0.42496700	H	-12.43524300	3.15217300	0.34990300
H	-4.67795300	-10.79602200	-3.47945200	H	-10.88823200	6.35197300	-2.04358700
H	-3.74062900	-12.80783200	-2.36687700	H	-12.82304000	5.22401600	-0.96619700
C	7.78800100	-5.54392400	1.72437600	C	-6.28069700	-7.27679600	1.31899000
C	9.10850700	-5.27431500	1.34886900	C	-6.22065300	-8.60549600	0.88833000
C	7.54938900	-6.53410700	2.68335400	C	-7.16611300	-6.93750800	2.34590000
C	10.15820700	-5.97480300	1.92418400	C	-7.02461500	-9.57008700	1.47730100
H	9.30674200	-4.51481700	0.60204700	H	-5.54247000	-8.87533100	0.08728200
C	8.60658500	-7.23999000	3.23877100	C	-7.97910900	-7.90696900	2.91567400
H	6.53374100	-6.74531100	2.99530600	H	-7.21402900	-5.91367800	2.69666600
C	9.91796200	-6.96581100	2.86877100	C	-7.91310200	-9.22857100	2.49041200
H	11.17408000	-5.74939000	1.61828200	H	-6.96420400	-10.59531600	1.12880000
H	8.39913000	-8.00324900	3.98094900	H	-8.66017500	-7.62484700	3.71112100
H	10.74065700	-7.51570100	3.31081700	H	-8.54639200	-9.98294500	2.94273300
C	7.79020000	5.54089700	-1.72437100	C	4.95185200	-8.17348300	-1.43666600
C	9.11059000	5.27081700	-1.34879200	C	4.67938700	-9.44389900	-0.92587000
C	7.55198800	6.53122000	-2.68330400	C	5.86851800	-8.03313300	-2.48136700
C	10.16056600	5.97096700	-1.92401200	C	5.32188200	-10.55509000	-1.45113700
H	9.30851700	4.51122100	-0.60198800	H	3.96682200	-9.55338600	-0.11698400
C	8.60946100	7.23676200	-3.23862500	C	6.51549300	-9.14974000	-2.98987000
H	6.53643000	6.74280000	-2.99529600	H	6.06200600	-7.05198400	-2.89853600
C	9.92072200	6.96210400	-2.86856700	C	6.24597000	-10.41480600	-2.48019500
H	11.17634200	5.74519000	-1.61805700	H	5.10381500	-11.53633400	-1.04514900
H	8.40231500	8.00013400	-3.98077300	H	7.22286800	-9.02955600	-3.80266500
H	10.74363400	7.51172900	-3.31053700	H	6.74863600	-11.28521500	-2.88539400
C	-2.84201600	9.25752000	1.07579700	C	9.71919900	2.06508300	0.88520900
C	-2.31624400	10.39199400	0.45185300	C	10.66857800	1.41174600	0.09131900
C	-3.69010300	9.41923700	2.17457300	C	10.13319900	3.00993200	1.82956500
C	-2.63113900	11.65848100	0.92193000	C	12.01526100	1.68815600	0.26167200
H	-1.66101300	10.27346900	-0.40313200	H	10.34370600	0.71264300	-0.66977500
C	-4.01364600	10.69096000	2.62563800	C	11.48263500	3.27936400	1.98604800
H	-4.09190500	8.54591000	2.67438700	H	9.39394900	3.51409400	2.43951400
C	-3.48531500	11.81799500	2.00699600	C	12.42918300	2.61983400	1.20819400
H	-2.21390900	12.52752500	0.42513900	H	12.74481900	1.18588200	-0.36301000
H	-4.67371100	10.79778600	3.47949700	H	11.79722800	4.00592300	2.72597600
H	-3.73553000	12.80924800	2.36701700	H	13.48345900	2.83690600	1.33334600
N	-2.50077900	-7.97341300	-0.60311800	N	-7.94041500	3.24134800	-0.47590600
N	6.72987300	-4.82083800	1.14944300	N	-5.44527200	-6.31103100	0.72333500
N	6.73177900	4.81817900	-1.14951500	N	4.26928100	-7.05061600	-0.91581600
N	-2.49761900	7.97437900	0.60312900	N	8.34617600	1.81000200	0.71345500

Structure S37. The coordinates of both the optimized structures of N5L$_2$ at S$_0$ and S$_1$ in toluene.

S_0-optimized structure				S_1-optimized structure			
C	5.47489600	5.36518600	-0.93462900	C	-5.47340400	-5.35643100	-0.88380000
C	5.27767800	6.66399700	-0.45643500	C	-5.26772100	-6.66631300	-0.43687300
C	4.35197100	4.58583700	-1.22337100	C	-4.35274200	-4.55693200	-1.12828900
C	3.99605100	7.15798800	-0.26974500	C	-3.98497000	-7.15114000	-0.24283500
H	6.13476600	7.28709300	-0.22936400	H	-6.12049100	-7.30604000	-0.24220600
C	3.07757100	5.08293400	-1.01853100	C	-3.07762100	-5.04530800	-0.91587200
H	4.48279600	3.57629200	-1.59390400	H	-4.48700600	-3.53852700	-1.47209800
C	2.86568400	6.37839600	-0.53660800	C	-2.85520800	-6.35397700	-0.46920700
H	3.87373500	8.17385500	0.09013600	H	-3.86048300	-8.17665600	0.08739100
H	2.22770000	4.43991700	-1.21805500	H	-2.23474800	-4.38393600	-1.08066600
C	1.49381200	6.86249300	-0.29011200	C	-1.48643900	-6.83560100	-0.22845000
C	0.43790800	6.47979300	-1.12522100	C	-0.40950600	-6.34649300	-0.98252300
C	1.18619800	7.66559100	0.81266200	C	-1.19181600	-7.75736700	0.78557800
C	-0.86784600	6.84393700	-0.84975500	C	0.89185900	-6.71856800	-0.71202600
H	0.64505200	5.89946500	-2.01755500	H	-0.59822600	-5.67671600	-1.81377100
C	-0.12130000	8.02200600	1.10530200	C	0.10945000	-8.13323400	1.06991400
H	1.97613400	7.96849400	1.49139200	H	-1.99256600	-8.14533300	1.40550500
C	-1.16947000	7.59364800	0.28914300	C	1.17641900	-7.60097800	0.33665100
H	-1.66931300	6.53283700	-1.50951600	H	1.70513400	-6.32565700	-1.31052200
H	-0.33794900	8.60636800	1.99198200	H	0.30900300	-8.81270400	1.89020500
C	6.95748200	3.46196200	-0.77304800	C	-6.97444600	-3.46647300	-0.74355100
C	6.59331300	2.99248700	0.48926900	C	-6.62474200	-2.98605700	0.51901900
C	7.46036800	2.55836200	-1.70993100	C	-7.47585100	-2.57254200	-1.69086300
C	6.68940100	1.64439700	0.79105400	C	-6.73019100	-1.63658600	0.80974200
H	6.20674600	3.68990400	1.22342100	H	-6.23983800	-3.67589900	1.26113700
C	7.55722300	1.21078800	-1.39903600	C	-7.58116900	-1.22339600	-1.39117200
H	7.75991800	2.91481200	-2.68877600	H	-7.76489200	-2.93757100	-2.66969000
C	7.15644600	0.72306100	-0.15129000	C	-7.19155000	-0.72366100	-0.14426700
H	6.36486000	1.29465600	1.76496200	H	-6.41463800	-1.27829300	1.78345800
H	7.94421900	0.52251900	-2.14270300	H	-7.96502700	-0.54297900	-2.14361600
C	7.15646900	-0.72285600	0.15125200	C	-7.19148700	0.72423000	0.14435400
C	6.68942300	-1.64420400	-0.79108000	C	-6.73007800	1.63711600	-0.80966600
C	7.55729000	-1.21057300	1.39898700	C	-7.58103300	1.22399700	1.39126900
C	6.59337800	-2.99229700	-0.48929200	C	-6.62451100	2.98657900	-0.51894800
H	6.36484900	-1.29447200	-1.76498000	H	-6.41457900	1.27879700	-1.78339100
C	7.46047700	-2.55814900	1.70988500	C	-7.47559800	2.57313500	1.69095600
H	7.94428600	-0.52229400	2.14264500	H	-7.96492700	0.54361200	2.14372300
C	6.95759000	-3.46176300	0.77301500	C	-6.97414500	3.46702500	0.74363100
H	6.20681000	-3.68972400	-1.22343500	H	-6.23957000	3.67638900	-1.26107600
H	7.76006100	-2.91459100	2.68872200	H	-7.76458500	2.93818900	2.66979000
C	5.47506000	-5.36502900	0.93461200	C	-5.47294900	5.35686400	0.88383700
C	5.27787700	-6.66384500	0.45641800	C	-5.26717400	6.66672400	0.43688900
C	4.35211500	-4.58571500	1.22336900	C	-4.35234500	4.55728100	1.12831100
C	3.99626400	-7.15787500	0.26974000	C	-3.98439000	7.15145000	0.24281800
H	6.13498300	-7.28691400	0.22933700	H	-6.11989900	7.30651500	0.24223400
C	3.07772800	-5.08285100	1.01854000	C	-3.07719000	5.04555600	0.91586200
H	4.48291300	-3.57616700	1.59390300	H	-4.48668100	3.53889100	1.47213600
C	2.86587600	-6.37831800	0.53661500	C	-2.85468600	6.35420100	0.46917600
H	3.87397500	-8.17374500	-0.09014200	H	-3.85982800	8.17695100	-0.08742500
H	2.22783900	-4.43985900	1.21807500	H	-2.23436400	4.38412200	1.08064600
C	1.49401600	-6.86245500	0.29013000	C	-1.48588300	6.83571600	0.22838300
C	0.43810700	-6.47978700	1.12524800	C	-0.40897900	6.34656100	0.98246300
C	1.18641700	-7.66555900	-0.81264300	C	-1.19120600	7.75741600	-0.78568800
C	-0.86763900	-6.84396900	0.84979100	C	0.89241100	6.71853100	0.71193600
H	0.64524100	-5.89945500	2.01758200	H	-0.59773900	5.67683400	1.81374200
C	-0.12107300	-8.02201200	-1.10527400	C	0.11008600	8.13317300	-1.07005800
H	1.97635600	-7.96843700	-1.49138100	H	-1.99193600	8.14541700	-1.40561900
C	-1.16924900	-7.59368600	-0.28910500	C	1.17702300	7.60087000	-0.33678400
H	-1.66911000	-6.53289400	1.50955900	H	1.70566400	6.32558600	1.31044100
H	-0.33771200	-8.60637900	-1.99195300	H	0.30967900	8.81259300	-1.89038000
C	-3.44167500	6.80849700	0.61020000	C	3.43994000	-6.83809900	0.68571000
C	-4.77857800	7.00617900	0.25012000	C	4.75096700	-6.99313300	0.20239400
C	-3.03402800	5.51273600	0.94091600	C	3.06267700	-5.57109500	1.16185300
C	-5.66628800	5.94222400	0.21816400	C	5.62279800	-5.92704100	0.17337900
H	-5.12455300	8.00062500	-0.00528600	H	5.07456800	-7.96057400	-0.16393100
C	-3.92155500	4.45495400	0.87683600	C	3.93591100	-4.51085400	1.12316400
H	-2.00619500	5.33065200	1.22907800	H	2.06320000	-5.42705000	1.55463500
C	-5.26110600	4.63663000	0.51624800	C	5.25235700	-4.63280200	0.61464500
H	-6.69973800	6.13816100	-0.04567700	H	6.62240200	-6.10259100	-0.20545700
H	-3.55328800	3.45954500	1.09624400	H	3.57773700	-3.55336900	1.47886000
C	-6.17677500	3.48217600	0.43273900	C	6.13766700	-3.49152000	0.52838000
C	-5.97333200	2.34890100	1.22878700	C	5.82769400	-2.26052700	1.16309500
C	-7.24788500	3.44967400	-0.46807200	C	7.34297700	-3.51386500	-0.22052000
C	-6.76951000	1.22531500	1.10342000	C	6.60717400	-1.14275600	1.01341500
H	-5.18802500	2.34998700	1.97588300	H	4.96435300	-2.18879700	1.81174200
C	-8.05237200	2.32803100	-0.59962700	C	8.13819500	-2.40239900	-0.36911300
H	-7.42750900	4.29751400	-1.11979500	H	7.63599900	-4.41688400	-0.74187200
C	-7.80873800	1.19103200	0.17261300	C	7.77768200	-1.17660700	0.22880600
H	-6.58718600	0.35785000	1.72705600	H	6.33635200	-0.22183900	1.51638700
H	-8.85447500	2.31751400	-1.32880500	H	9.02741200	-2.45435900	-0.98683700
C	-3.44147800	-6.80860000	-0.61015800	C	3.44049000	6.83781600	-0.68583900
C	-4.77837500	-7.00631700	-0.25007400	C	4.75153300	6.99278100	-0.20255400
C	-3.03386900	-5.51283100	-0.94088500	C	3.06313100	5.57082000	-1.16192200
C	-5.66611500	-5.94238700	-0.21812600	C	5.62329200	5.92662800	-0.17350500
H	-5.12432100	-8.00077000	0.00534100	H	5.07520900	7.96021700	0.16371800
C	-3.92142600	-4.45507300	-0.87681400	C	3.93629200	4.51051700	-1.12319500
H	-2.00604200	-5.33071900	-1.22905200	H	2.06363600	5.42682600	-1.55467700
C	-5.26097100	-4.63678400	-0.51622300	C	5.25274900	4.63240000	-0.61470200
H	-6.69956000	-6.13835200	0.04571800	H	6.62291800	6.10212400	0.20529600
H	-3.55318800	-3.45965600	-1.09623300	H	3.57804500	3.55303600	-1.47882800
C	-6.17667400	-3.48235600	-0.43272600	C	6.13797800	3.49105100	-0.52838800
C	-5.97326400	-2.34908400	-1.22878900	C	5.82796400	2.26009300	-1.16314100
C	-7.24778300	-3.44987200	0.46808600	C	7.34322300	3.51330300	0.22060900
C	-6.76947400	-1.22552000	-1.10343600	C	6.60734900	1.14226100	-1.01340900

H	-5.18795700	-2.35015700	-1.97588500
C	-8.05230300	-2.32825000	0.59962800
H	-7.42738200	-4.29770900	1.11982100
C	-7.80870300	-1.19125500	-0.17263000
H	-6.58717500	-0.35805800	-1.72708400
H	-8.85440400	-2.31774700	1.32880700
C	-9.95059600	-0.00013500	-0.00004000
C	-10.66476300	0.97441900	0.70697700
C	-10.66472400	-0.97469700	-0.70708600
C	-12.05159000	0.97347400	0.69593100
H	-10.12835600	1.72930500	1.26901600
C	-12.05155100	-0.97377000	-0.69609100
H	-10.12828600	-1.72957400	-1.26910700
C	-12.75832600	-0.00015300	-0.00009200
H	-12.58306300	1.73862900	1.25153600
H	-12.58299400	-1.73893100	-1.25171700
H	-13.84212000	-0.00016000	-0.00011300
C	-2.88092100	-9.15805800	-1.05254400
C	-2.33340900	-10.28447600	-0.43174600
C	-3.77903800	-9.33651100	-2.10862300
C	-2.67474500	-11.55750900	-0.86404700
H	-1.64069800	-10.15504800	0.39145600
C	-4.12778500	-10.61448300	-2.52120500
H	-4.19999600	-8.47076700	-2.60575800
C	-3.57707500	-11.73309700	-1.90680500
H	-2.23956200	-12.41923300	-0.36982200
H	-4.82639700	-10.73313100	-3.34226600
H	-3.84734100	-12.72949500	-2.23687000
C	7.81107000	-5.56736800	1.67608900
C	9.13636800	-5.31935000	1.30268000
C	7.55478400	-6.55284600	2.63527300
C	10.17367100	-6.03654500	1.87983500
H	9.34788600	-4.56338200	0.55589600
C	8.59951600	-7.27566800	3.19258600
H	6.53539800	-6.74722200	2.94594800
C	9.91573100	-7.02314200	2.82439300
H	11.19357000	-5.82784100	1.57549400
H	8.37854300	-8.03501400	3.93486800
H	10.72863500	-7.58630700	3.26784100
C	7.81089900	5.56759500	-1.67611300
C	9.13620500	5.31960500	-1.30271200
C	7.55458600	6.55307700	-2.63528600
C	10.17348900	6.03683000	-1.87986500
H	9.34774400	4.56363400	-0.55593700
C	8.59929800	7.27592900	-3.19259700
H	6.53519500	6.74743100	-2.94595600
C	9.91552100	7.02343000	-2.82441200
H	11.19339400	5.82814600	-1.57553000
H	8.37830400	8.03527700	-3.93487100
H	10.72841000	7.58661900	-3.26785800
C	-2.88118200	9.15796700	1.05260200
C	-2.33370500	10.28440500	0.43180900
C	-3.77929900	9.33638700	2.10868600
C	-2.67507500	11.55742500	0.86412000
H	-1.64099400	10.15500200	-0.39139700
C	-4.12808100	10.61434600	2.52127800
H	-4.20023000	8.47062700	2.60581700
C	-3.57740600	11.73298000	1.90688400
H	-2.23991800	12.41916400	0.36990000
H	-4.82669300	10.73296900	3.34234300
H	-3.84769800	12.72936800	2.23695700
N	-2.51541700	-7.86809500	-0.62007400
N	6.76674300	-4.82626500	1.09849900
N	6.76659300	4.82646000	-1.09852800
N	-2.51564300	7.86801700	0.62012100
N	-8.54991700	-0.00012100	-0.00001600
H	4.96467200	2.18843900	-1.81186300
C	8.13834900	2.40177500	0.36925700
H	7.63626100	4.41629300	0.74200400
C	7.77779300	1.17601800	-0.22870300
H	6.33650200	0.22136800	-1.51641100
H	9.02751700	2.45366200	0.98705800
C	9.90143400	-0.00037700	0.00014600
C	10.60598700	-0.84767900	0.86171500
C	10.60613400	0.84686900	-0.86135900
C	11.99171700	-0.84662500	0.85296100
H	10.06025000	-1.49063400	1.54138000
C	11.99186200	0.84570400	-0.85247600
H	10.06051100	1.48986700	-1.54107500
C	12.69287500	-0.00048800	0.00027400
H	12.52725700	-1.50376500	1.52885500
H	12.52751700	1.50280300	-1.52832100
H	13.77672200	-0.00053100	0.00032400
C	2.89914900	9.20069300	-1.00249900
C	2.33304600	10.29526100	-0.34048600
C	3.85502200	9.42692600	-1.99826800
C	2.71193100	11.58557900	-0.67749500
H	1.60226200	10.12682100	0.44139100
C	4.23623000	10.72150400	-2.31648000
H	4.28828900	8.58411500	-2.52315900
C	3.66722900	11.80912300	-1.66307700
H	2.26461600	12.42298100	-0.15340800
H	4.97591400	10.87968300	-3.09358100
H	3.96498700	12.81943500	-1.91864200
C	-7.79666400	5.57896700	1.65558400
C	-9.12697600	5.35442500	1.28572100
C	-7.52083400	6.54590200	2.62795000
C	-10.15114200	6.07695300	1.87930800
H	-9.35215000	4.61265300	0.52872900
C	-8.55237000	7.27445600	3.20206900
H	-6.49696300	6.72059100	2.93560300
C	-9.87386600	7.04547200	2.83711500
H	-11.17556800	5.88720800	1.57786500
H	-8.31723700	8.01900800	3.95487600
H	-10.67645400	7.61305200	3.29356700
C	-7.79715600	-5.57834800	-1.65548900
C	-9.12744000	-5.35370200	-1.28558900
C	-7.52142700	-6.54529900	-2.62786800
C	-10.15167900	-6.07614600	-1.87915300
H	-9.35253500	-4.61191600	-0.52858800
C	-8.55303700	-7.27376900	-3.20196400
H	-6.49757900	-6.72006700	-2.93554800
C	-9.87450500	-7.04468100	-2.83697300
H	-11.17608200	-5.88632100	-1.57768200
H	-8.31798200	-8.01833500	-3.95478000
H	-10.67715000	-7.61219500	-3.29340700
C	2.89843700	-9.20094700	1.00228500
C	2.33224500	-10.29545100	0.34024200
C	3.85431400	-9.42728300	1.99802700
C	2.71104600	-11.58580800	0.67719600
H	1.60145900	-10.12693200	-0.44161600
C	4.23543500	-10.72189900	2.31618500
H	4.28764900	-8.58452100	2.52294100
C	3.66634600	-11.80945400	1.66275300
H	2.26366300	-12.42316000	0.15308600
H	4.97512200	-10.88015900	3.09326800
H	3.96403700	-12.81979700	1.91827600
N	2.51408000	7.89015500	-0.66662700
N	-6.76639800	4.83067600	1.06065300
N	-6.76681500	-4.83014000	-1.06058100
N	2.51345800	-7.89037100	0.66646600
N	8.49549400	-0.00032300	0.00008000

Structure S38. The coordinates of both the optimized structures of $B6L_2$ at S_0 and S_1 in toluene.

S_0-optimized structure				S_1-optimized structure			
B	9.64117300	-3.36684300	-0.68021900	B	-10.29791500	0.64310200	-0.02331500
C	8.49530100	-4.37871000	-0.30175000	C	-9.41393400	1.93594800	0.15709800
C	7.96441700	-4.49855200	0.98867400	C	-8.62607500	2.14668800	1.30105300
C	7.94076500	-5.19833500	-1.29471200	C	-9.36758000	2.93511100	-0.82930200
C	6.92765400	-5.37705900	1.27027800	C	-7.84792000	3.28206600	1.45380200
H	8.37949100	-3.91227000	1.80234200	H	-8.64585200	1.41935700	2.10689000
C	6.90864100	-6.08290600	-1.01750700	C	-8.59211600	4.07391900	-0.68235000
H	8.31315100	-5.13630200	-2.31289200	H	-9.93777000	2.80858800	-1.74449600
C	6.37935400	-6.18435700	0.27096500	C	-7.81139100	4.27041600	0.46250500
H	6.55341300	-5.45745100	2.28553400	H	-7.28213200	3.42287700	2.36852200
H	6.48886200	-6.68569700	-1.81601000	H	-8.56338100	4.80723400	-1.48119500
C	5.27039000	-7.11618000	0.56165400	C	-6.97288100	5.47154200	0.61829400
C	4.16863200	-6.70662200	1.31905600	C	-5.71279400	5.39434300	1.22412000
C	5.28912200	-8.42675900	0.07527000	C	-7.40831200	6.72216600	0.16358100
C	3.10819500	-7.56949100	1.54833800	C	-4.91438100	6.51912700	1.34640300
H	4.12870200	-5.68916000	1.69350500	H	-5.34359200	4.43384200	1.56685200
C	4.24997000	-9.30061800	0.35519800	C	-6.62184900	7.85025200	0.32930400
H	6.14274800	-8.76856900	-0.50051100	H	-8.38887900	6.81315800	-0.29096600
C	3.12160800	-8.89399300	1.08399800	C	-5.34658100	7.78374400	0.91421200
H	2.24911500	-7.21068500	2.10603800	H	-3.93171600	6.41903800	1.79546100
H	4.30604600	-10.31993100	-0.01274900	H	-6.99977300	8.80829200	-0.01198500
C	9.32174000	-1.83243200	-0.71857600	C	-9.55456200	-0.69914200	-0.29038200
C	8.54596900	-1.20830400	0.26865000	C	-8.21472600	-0.92228000	0.13117300
C	9.76931400	-1.01982000	-1.77196000	C	-10.13783900	-1.75157200	-1.05132300
C	8.28533400	0.15095900	0.24142400	C	-7.56328600	-2.10638200	-0.07967100
H	8.14981000	-1.79223100	1.09139500	H	-7.68752100	-0.12857700	0.64603500
C	9.46522300	0.33146900	-1.83387100	C	-9.48951900	-2.93305800	-1.29442400
H	10.34779900	-1.46025200	-2.57730400	H	-11.13227000	-1.61437900	-1.45902100
C	8.73425300	0.95160000	-0.81529700	C	-8.17762500	-3.18381000	-0.78760500
H	7.73469100	0.59991500	1.06009600	H	-6.54448300	-2.20527600	0.27063000
H	9.79003700	0.90888700	-2.69205400	H	-10.00727400	-3.70172500	-1.85304100
C	8.44678100	2.40034000	-0.84408700	C	-7.51707700	-4.43557900	-0.97139900
C	7.30237600	2.92099400	-0.22949400	C	-6.32451300	-4.76270900	-0.25571400
C	9.31690400	3.30094000	-1.46999800	C	-8.01003100	-5.43632700	-1.86434300
C	7.04937900	4.28229600	-0.23150100	C	-5.69997300	-5.96892900	-0.41804700
H	6.59074700	2.25048200	0.23854500	H	-5.92050800	-4.06257700	0.46359000
C	9.05948900	4.66194100	-1.46311200	C	-7.37416000	-6.63976800	-2.01249400
H	10.22284900	2.93488000	-1.93994500	H	-8.88779500	-5.23618900	-2.46507400
C	7.92098600	5.20018000	-0.84045400	C	-6.18842300	-6.97357800	-1.29911600
H	6.15586900	4.64633500	0.26346100	H	-4.81376800	-6.17806100	0.16979800
H	9.76153400	5.32815600	-1.95273400	H	-7.77842100	-7.36022100	-2.71449600
B	7.64735900	6.73893200	-0.79475900	B	-5.48689900	-8.35281100	-1.43442600
B	1.92364600	-9.86612000	1.34150700	B	-4.44629200	9.05215700	1.07202000
C	6.18437200	7.27055300	-0.62799900	C	-3.96838000	-8.48520500	-1.09922900
C	5.92141600	8.39960800	0.16344700	C	-3.44523000	-9.64653200	-0.49337300
C	5.07364700	6.62505900	-1.19497800	C	-3.04246900	-7.45305400	-1.35763500
C	4.62834800	8.82868000	0.41748600	C	-2.11916000	-9.74635700	-0.11744500
H	6.74914100	8.93171400	0.62016000	H	-4.11391800	-10.47240200	-0.27547500
C	3.77961700	7.06554900	-0.96962500	C	-1.71067300	-7.55607200	-1.01026000
H	5.22879800	5.76199200	-1.83401700	H	-3.38076800	-6.55289700	-1.86016700
C	3.53100000	8.16285300	-0.13727800	C	-1.21624700	-8.69787300	-0.35699000
H	4.46393100	9.66904700	1.08291300	H	-1.78506200	-10.63349800	0.40844500
H	2.94941200	6.55604400	-1.44661100	H	-1.03404000	-6.74732100	-1.26120800
C	0.45960000	-9.32704000	1.22610700	C	-2.89707300	8.92676100	0.89190500
C	0.14346800	-8.31920800	0.30171900	C	-2.34683400	8.01493700	-0.02273400
C	-0.60220000	-9.84091400	1.98797400	C	-1.99159100	9.72632200	1.60797700
C	-1.15678700	-7.87838700	0.11850800	C	-0.98057300	7.92531800	-0.23242400
H	0.93204200	-7.89385500	-0.30973100	H	-3.00837000	7.38050100	-0.60302400
C	-1.90061400	-9.38202100	1.83347700	C	-0.62196800	9.62069600	1.42643500
H	-0.40251300	-10.60830200	2.72852900	H	-2.37024100	10.43491100	2.33708900
C	-2.20574800	-8.40283400	0.88109500	C	-0.09000100	8.72600800	0.49103400
H	-1.36701300	-7.13861900	-0.64578900	H	-0.59921000	7.24373300	-0.98460800
H	-2.68730900	-9.77943200	2.46534700	H	0.04561200	10.22854500	2.02717400
C	2.15236500	8.58072500	0.18781000	C	0.18123300	-8.77035000	0.08853500
C	1.14181500	7.62809300	0.35229500	C	0.90032700	-7.60229200	0.38441500
C	1.82494400	9.92768900	0.38189600	C	0.83957900	-9.99861400	0.26016400
C	-0.14022500	8.00919800	0.71283600	C	2.20742600	-7.66325400	0.83065400
H	1.37312800	6.57562700	0.23100100	H	0.41748400	-6.63629700	0.29416000
C	0.54696400	10.29836200	0.76771800	C	2.13641200	-10.05161900	0.73941200
H	2.57950400	10.69034200	0.22245700	H	0.33276400	-10.92088000	0.00010400
C	-0.47355900	9.35147200	0.95464500	C	2.86370900	-8.88752600	1.04453200
H	-0.89643900	7.24300000	0.84537200	H	2.72776700	-6.73790700	1.05303900
H	0.32999400	11.35055300	0.91889800	H	2.60809800	-11.02051500	0.86563600
C	-3.59280500	-7.94972400	0.65589200	C	1.36437000	8.63652100	0.25354400
C	-3.86596200	-6.63181000	0.27261800	C	1.96451900	7.41905800	-0.08743900
C	-4.67215500	-8.82952900	0.79389500	C	2.18300400	9.76732400	0.34931900
C	-5.16157100	-6.22484100	0.00598400	C	3.32271400	7.34502100	-0.34297300
H	-3.05531500	-5.91477900	0.20582500	H	1.36309200	6.51761600	-0.12509000
C	-5.96368900	-8.41907800	0.50725400	C	3.53743400	9.68909300	0.06991400
H	-4.49179600	-9.85660100	1.09122500	H	1.74571400	10.72456900	0.61078600
C	-6.24880900	-7.11161100	0.07832600	C	4.14854000	8.48075000	-0.30527200
H	-5.33718600	-5.19191700	-0.27319600	H	3.75740400	6.38095500	-0.58303900
H	-6.77250600	-9.13225200	0.61968300	H	4.13765000	10.58852800	0.14933100
B	-1.89936000	9.73174600	1.47035900	B	4.30855900	-8.91167500	1.63841300
B	-7.68978100	-6.64449700	-0.30269500	B	5.66740500	8.37814900	-0.65404300
C	-8.01370700	-5.10257000	-0.33676100	C	6.38077500	6.97539700	-0.56787700
C	-8.72936800	-4.44755100	0.67252400	C	7.18639000	6.59888600	0.51346800
C	-7.58749700	-4.32630000	-1.42242900	C	6.23090300	6.04639700	-1.60545000
C	-9.00936300	-3.08919600	0.60058600	C	7.81598300	5.36182100	0.55463000
H	-9.06558000	-5.00228500	1.54337800	H	7.31921200	7.27862500	1.34987100
C	-7.85381400	-2.96729300	-1.49305800	C	6.84649700	4.80441000	-1.56268300
H	-7.04222900	-4.79316600	-2.23738200	H	5.62846100	6.29951800	-2.47289800
C	-8.57175200	-2.32315400	-0.48225700	C	7.65335400	4.43968100	-0.48182500
H	-9.54945300	-2.60970200	1.41038300	H	8.41925500	5.09597400	1.41644100
H	-7.52313300	-2.40025100	-2.35683200	H	6.72342900	4.11639400	-2.39241700

C	-8.84809400	-0.87480100	-0.56772300	C	8.30923800	3.11679400	-0.45102800
C	-7.85293400	0.01258700	-0.98874400	C	7.61590300	1.97117700	-0.85329700
C	-10.10515400	-0.35328100	-0.24523500	C	9.63788800	2.97372600	-0.03765900
C	-8.10630400	1.37165300	-1.07420500	C	8.23043800	0.72984900	-0.83825000
H	-6.86373700	-0.36761900	-1.22036600	H	6.57671500	2.05529400	-1.15286200
C	-10.36505800	1.00218500	-0.37894000	C	10.26006000	1.73510500	-0.06905900
H	-10.89459700	-1.02521300	0.07471900	H	10.19696200	3.85085700	0.27081200
C	-9.37388500	1.90583500	-0.79351900	C	9.57622900	0.57595700	-0.46904700
H	-7.30587100	2.03737700	-1.37953000	H	7.65894200	-0.14324700	-1.13514500
H	-11.35971300	1.37145400	-0.15101100	H	11.30158500	1.66275800	0.22688000
C	-3.12365100	8.84412100	1.06681300	C	5.28328200	-7.73444400	1.29961300
C	-4.09462600	8.47971900	2.01194300	C	6.08069500	-7.13429200	2.28606900
C	-3.28179100	8.34374500	-0.23464900	C	5.37123400	-7.20772200	0.00173400
C	-5.13910500	7.62722800	1.68947700	C	6.88569200	-6.04096200	2.00415300
H	-4.01749200	8.85761700	3.02620600	H	6.05313200	-7.52031900	3.29991200
C	-4.35677800	7.53887000	-0.57898200	C	6.22047500	-6.15492600	-0.30039600
H	-2.54699100	8.59330500	-0.99336100	H	4.76207400	-7.63579100	-0.78789600
C	-5.29116500	7.14837700	0.38513100	C	6.97512800	-5.53893200	0.70261900
H	-5.86440200	7.34921900	2.44682400	H	7.47599300	-5.58551100	2.79232900
H	-4.45220600	7.16809700	-1.59409200	H	6.26854900	-5.77148200	-1.31416200
C	-6.39833700	6.23312900	0.04160400	C	7.82504200	-4.37057000	0.39574500
C	-7.16303600	6.42118000	-1.11339000	C	8.67867100	-4.36868300	-0.71122900
C	-6.69593300	5.14332300	0.86529200	C	7.77705800	-3.22790800	1.19991900
C	-8.20749500	5.56055200	-1.41687100	C	9.47435200	-3.26693500	-0.98766200
H	-6.95467400	7.26842000	-1.75828200	H	8.73881300	-5.25141900	-1.33919400
C	-7.70237600	4.25438900	0.52353200	C	8.52912800	-2.10843900	0.88086000
H	-6.10229900	4.97169600	1.75692100	H	7.10918600	-3.20626300	2.05458900
C	-8.49678800	4.44420900	-0.61752000	C	9.40905500	-2.09946000	-0.21213500
H	-8.80412500	5.74909500	-2.30355900	H	10.15052800	-3.30537700	-1.83563900
H	-7.88295900	3.39366600	1.15917000	H	8.43728100	-1.21925500	1.49612900
B	-9.63961400	3.43556500	-0.97357700	B	10.25692300	-0.82836800	-0.55310800
C	2.19527700	-11.37012500	1.67019800	C	-5.09430400	10.43923100	1.39038700
C	1.29468800	-12.38205100	1.29868300	C	-4.52750700	11.63832700	0.92754800
C	3.36144700	-11.76373700	2.34733400	C	-6.27185200	10.53633400	2.15025100
C	1.54773800	-13.71790400	1.57887800	C	-5.11084500	12.86891100	1.19668400
H	0.38844700	-12.11557500	0.76520400	H	-3.62111200	11.60109400	0.33250200
C	3.60856300	-13.09445700	2.65519500	C	-6.84605500	11.76483500	2.44673100
H	4.07944000	-11.00776200	2.64739900	H	-6.73588800	9.63039300	2.52575500
C	2.70314500	-14.07521600	2.26516600	C	-6.26885600	12.93414800	1.96371100
H	0.84353900	-14.48135900	1.26628300	H	-4.66129500	13.77845100	0.81309600
H	4.50938000	-13.36951100	3.19310200	H	-7.74688500	11.81192200	3.04901400
H	2.89868600	-15.11737400	2.49427500	H	-6.72136700	13.89505600	2.18436100
C	-8.80857600	-7.65608400	-0.71886300	C	6.49560700	9.61366600	-1.13733900
C	-8.51057900	-8.86584100	-1.36818000	C	5.90579300	10.67283200	-1.84748800
C	-10.16520900	-7.35753000	-0.51166700	C	7.88250800	9.67799200	-0.92482700
C	-9.51133700	-9.73827900	-1.77469000	C	6.65664200	11.74635600	-2.30723800
H	-7.47732500	-9.11578000	-1.58217800	H	4.84402000	10.64284200	-2.06568800
C	-11.16906400	-8.23938200	-0.88422000	C	8.63404300	10.76271500	-1.35230900
H	-10.43677100	-6.41480900	-0.04911800	H	8.37905000	8.86045600	-0.41327200
C	-10.84308900	-9.43256300	-1.52024200	C	8.02120800	11.79953200	-2.04794600
H	-9.25306600	-10.65762000	-2.28899100	H	6.17798600	12.54284000	-2.86656000
H	-12.20771400	-7.99075700	-0.69516700	H	9.70073800	10.79426300	-1.15869100
H	-11.62670900	-10.11668000	-1.82772900	H	8.60866800	12.64184500	-2.39732300
C	-11.00881400	3.95432000	-1.51835300	C	11.74711200	-0.97378000	-0.99958300
C	-11.81189300	3.16653300	-2.35971600	C	12.36086300	-0.02838600	-1.83803100
C	-11.48465400	5.23414900	-1.18807900	C	12.52689500	-2.06481400	-0.58102500
C	-13.02172600	3.63373100	-2.85437400	C	13.68048100	-0.16766500	-2.24536500
H	-11.47127600	2.17595100	-2.64176300	H	11.78541200	0.82181700	-2.18836800
C	-12.70484500	5.69905700	-1.65864200	C	13.85430200	-2.19824100	-0.96390800
H	-10.88978900	5.86859800	-0.53954400	H	12.08388200	-2.81392300	0.06673700
C	-13.47314000	4.89990500	-2.49828300	C	14.43165500	-1.25086700	-1.80238200
H	-13.61592900	3.01080000	-3.51418400	H	14.12594000	0.56885100	-2.90534500
H	-13.05599700	6.68576000	-1.37650700	H	14.43845500	-3.04280600	-0.61473100
H	-14.42269500	5.26414800	-2.87580800	H	15.46592800	-1.35751000	-2.11183400
C	-2.10958000	10.95063800	2.42673300	C	4.77036800	-10.06013100	2.59174700
C	-3.31836600	11.66636700	2.42482500	C	6.12436800	-10.42523400	2.67896200
C	-1.11464500	11.37142100	3.32457600	C	3.86181400	-10.76285200	3.40082700
C	-3.51750600	12.75847200	3.25803100	C	6.54833200	-11.45006400	3.51334700
H	-4.11049600	11.36472300	1.74755600	H	6.85353700	-9.90112700	2.07010200
C	-1.31640400	12.44564800	4.18045100	C	4.28143600	-11.77201800	4.25647100
H	-0.17334500	10.83363100	3.36352900	H	2.81000900	-10.49914900	3.36958300
C	-2.51678200	13.14650500	4.14219500	C	5.62626400	-12.12228800	4.30827000
H	-4.45467700	13.30350300	3.22501500	H	7.59771500	-11.72190800	3.55011300
H	-0.53744200	12.73982500	4.87553800	H	3.56108900	-12.28849400	4.88165900
H	-2.67341900	13.99238600	4.80306000	H	5.95574800	-12.91679900	4.96932000
C	8.83243200	7.75939900	-0.86343600	C	-6.29875700	-9.62174100	-1.86698800
C	10.08267800	7.48491500	-0.28572900	C	-7.64340800	-9.81348000	-1.50141600
C	8.67897500	8.99957800	-1.50448900	C	-5.70175000	-10.63659100	-2.63709200
C	11.12352600	8.40241800	-0.33716800	C	-8.35041600	-10.94591600	-1.88059600
H	10.23454500	6.54129500	0.22773900	H	-8.13839100	-9.06477700	-0.89118000
C	9.72423400	9.90954600	-1.58654500	C	-6.40709800	-11.76359100	-3.03573800
H	7.72410500	9.24699900	-1.95669300	H	-4.66545400	-10.52785400	-2.94027900
C	10.94813300	9.61398900	-0.99645500	C	-7.73515800	-11.92404200	-2.65527800
H	12.07374400	8.17192300	0.13243400	H	-9.38338100	-11.06819000	-1.57202400
H	9.58358100	10.85300400	-2.10291200	H	-5.92085800	-12.52039200	-3.64232000
H	11.76323200	10.32795100	-1.04794800	H	-8.28731100	-12.80698000	-2.95895600
C	11.06097200	-3.90176700	-1.05168700	C	-11.85329000	0.74214100	0.03062500
C	12.17515400	-3.04647200	-1.11176200	C	-12.66305800	-0.37320200	0.32565900
C	11.29120900	-5.26969000	-1.27845100	C	-12.51759200	1.97404400	-0.13061800
C	13.44473500	-3.52474600	-1.40301000	C	-14.04283300	-0.27367300	0.42138200
H	12.04507400	-1.98955600	-0.90860000	H	-12.19718100	-1.33479400	0.51095100
C	12.55373900	-5.75373500	-1.58690800	C	-13.89768900	2.07797900	-0.05771100
H	10.46216900	-5.96501400	-1.20914100	H	-11.93426400	2.86950400	-0.31428200
C	13.63389000	-4.87974600	-1.65048800	C	-14.66836500	0.95193100	0.21646300
H	14.28787500	-2.84333300	-1.43498400	H	-14.63349500	-1.15176500	0.65995200
H	12.70069600	-6.81299500	-1.76738700	H	-14.37635500	3.04086100	-0.20112000
H	14.62448400	-5.25654600	-1.88193500	H	-15.74790000	1.03228600	0.28463600

Structure S39. The coordinates of both the optimized structures of B5N1L$_2$ at S$_0$ and S$_1$ in toluene.

S$_0$-optimized structure				S$_1$-optimized structure			
B	-9.29163800	4.07065100	-0.75810600	B	-8.70031300	5.19684200	-0.07294100
C	-8.08447200	4.99780200	-0.35201800	C	-7.32010900	5.93876700	0.12273500
C	-7.58275300	5.08654700	0.95251100	C	-6.58365400	5.82178000	1.30945800
C	-7.43931000	5.76316600	-1.33339800	C	-6.77024200	6.74483100	-0.88315000
C	-6.48584300	5.88029100	1.25794400	C	-5.36655200	6.46594600	1.48102100
H	-8.06579000	4.54066500	1.75685700	H	-6.97533100	5.22445600	2.12752100
C	-6.34884600	6.56617000	-1.03202500	C	-5.55852500	7.39972500	-0.71450700
H	-7.78607700	5.72143600	-2.36166900	H	-7.29223000	6.85362800	-1.82927700
C	-5.84753000	6.63315200	0.26976800	C	-4.83084800	7.26977300	0.47135400
H	-6.13243700	5.93674800	2.28226100	H	-4.83621200	6.36742300	2.42255800
H	-5.86071700	7.12827900	-1.82134800	H	-5.15508600	7.99698500	-1.52561800
C	-4.67191600	7.47129400	0.58378800	C	-3.53567900	7.95599200	0.64951000
C	-3.61719000	6.96852700	1.35190500	C	-2.45874700	7.30956000	1.26545300
C	-4.57755900	8.78177800	0.10635700	C	-3.34816900	9.26847500	0.20360800
C	-2.49175800	7.73963100	1.59807500	C	-1.23538700	7.94526600	1.40439600
H	-3.66618400	5.94915000	1.72007000	H	-2.57541100	6.28551400	1.60387600
C	-3.47397400	9.56607700	0.40479400	C	-2.13501700	9.91322100	0.38606100
H	-5.39296200	9.19506200	-0.47788100	H	-4.17442700	9.79605800	-0.26095000
C	-2.39103200	9.06329000	1.14233100	C	-1.03770600	9.26886200	0.97941600
H	-1.67108400	7.30766600	2.16179100	H	-0.41173200	7.40552300	1.86029200
H	-3.44192200	10.58890300	0.04371600	H	-2.02991900	10.93954000	0.04983900
C	-9.08186000	2.52015400	-0.78641800	C	-8.69740800	3.66580400	-0.34850400
C	-8.33055500	1.85479800	0.19354500	C	-7.60403100	2.85326200	0.02019700
C	-9.60455600	1.72342900	-1.81773300	C	-9.75358500	3.00850200	-1.01672200
C	-8.16946500	0.48048400	0.18487500	C	-7.59684700	1.48997700	-0.18368700
H	-7.87577600	2.42109200	0.99826700	H	-6.73859000	3.31253200	0.48487800
C	-9.39285600	0.35457700	-1.86603200	C	-9.74034800	1.65042500	-1.26066900
H	-10.16529600	2.19067100	-2.62060500	H	-10.60210300	3.58745400	-1.36361200
C	-8.69036400	-0.30585200	-0.85084500	C	-8.67232400	0.84270900	-0.82387000
H	-7.63690000	0.01036000	1.00330700	H	-6.72691200	0.91711400	0.11733900
H	-9.77106700	-0.20642600	-2.71310200	H	-10.58985600	1.19736100	-1.75954700
C	-8.50385700	-1.77004800	-0.86504800	C	-8.67904600	-0.60013800	-1.01527000
C	-7.43825700	-2.37837400	-0.19295300	C	-7.96214100	-1.45400900	-0.14944000
C	-9.39090000	-2.61889100	-1.53673300	C	-9.41289200	-1.21186300	-2.05591200
C	-7.27561200	-3.75172000	-0.16937200	C	-7.97252200	-2.81875700	-0.30442100
H	-6.70672900	-1.76904000	0.32472600	H	-7.43203400	-1.03433700	0.69634400
C	-9.22357500	-3.99238100	-1.54602700	C	-9.43121200	-2.57561000	-2.22638500
H	-10.24563500	-2.20347600	-2.05800400	H	-9.94040800	-0.59343000	-2.77143700
C	-8.16533900	-4.58953400	-0.85116000	C	-8.71120300	-3.41054400	-1.34869100
H	-6.44665300	-4.17778800	0.38130700	H	-7.45179800	-3.44958900	0.40512100
H	-9.92907300	-4.61156700	-2.08615400	H	-9.96921100	-3.01060000	-3.05976100
B	-1.11847800	9.93090200	1.41502200	B	0.34189800	9.98433300	1.14527700
C	-6.74957200	-6.57166300	-0.61803400	C	-7.57568600	-5.53341100	-1.19793700
C	-6.60835300	-7.63499800	0.27638200	C	-7.68113400	-6.77933600	-0.54578500
C	-5.61393200	-6.10300400	-1.28444700	C	-6.29112500	-5.03155600	-1.49197300
C	-5.36307100	-8.18999900	0.51649400	C	-6.55202300	-7.46230900	-0.17046800
H	-7.47751700	-8.00942700	0.80381200	H	-8.65925600	-7.16952200	-0.29183800
C	-4.36991200	-6.65017700	-1.02154000	C	-5.17093700	-5.73203100	-1.12393100
H	-5.71078000	-5.30045500	-2.00617500	H	-6.19599400	-4.10392600	-2.04364800
C	-4.21290000	-7.69862700	-0.10846700	C	-5.24851000	-6.96263100	-0.42258600
H	-5.27653400	-8.98588000	1.24790700	H	-6.67436600	-8.38304500	0.38447300
H	-3.50618500	-6.27258100	-1.55770400	H	-4.20533600	-5.33818800	-1.41274500
C	0.29661600	9.27513100	1.29209700	C	1.66284300	9.16770700	0.95086500
C	0.52879000	8.25972400	0.35125100	C	1.72701000	8.11307200	0.02673200
C	1.39760800	9.68910100	2.05913300	C	2.84055400	9.46049700	1.65730000
C	1.78928200	7.71970600	0.15602100	C	2.89982000	7.41214100	-0.20083400
H	-0.29272600	7.90862500	-0.26395100	H	0.84273600	7.85516600	-0.54620900
C	2.65475300	9.13042200	1.89253500	C	4.00995500	8.74432100	1.45691300
H	1.26155100	10.45744200	2.81301500	H	2.83418500	10.25758300	2.39333000
C	2.87889100	8.14679900	0.92229000	C	4.06487300	7.71293600	0.51274000
H	1.93783600	6.97797400	-0.62075400	H	2.92153600	6.63771200	-0.95945900
H	3.47202900	9.45259700	2.52837800	H	4.88811600	8.97674300	2.04947900
C	-2.88102300	-8.23565300	0.22759800	C	-4.06325600	-7.65362500	0.03908700
C	-1.77131600	-7.38764600	0.30869200	C	-2.81259400	-6.99760000	0.12215400
C	-2.69253900	-9.59240000	0.51767700	C	-4.09616100	-9.00340300	0.46382400
C	-0.53068300	-7.87547200	0.68449300	C	-1.69796100	-7.63490700	0.61612300
H	-1.89274100	-6.32792400	0.11252800	H	-2.72892900	-5.95486300	-0.16001300
C	-1.45423900	-10.06763300	0.91705200	C	-2.97714500	-9.62586500	0.96864300
H	-3.52543000	-10.28058500	0.42137000	H	-5.01138300	-9.57703000	0.37226300
C	-0.33553400	-9.22428200	1.02411600	C	-1.72727100	-8.96998300	1.08547800
H	0.30318100	-7.18535600	0.75423000	H	-0.77046900	-7.07693600	0.68512800
H	-1.34684100	-11.12344600	1.14232700	H	-3.05471300	-10.66447600	1.27220500
C	4.22563100	7.59091800	0.68225800	C	5.31459400	6.97060800	0.25411300
C	4.39445500	6.26268900	0.27502300	C	5.28683600	5.61401800	-0.08842100
C	5.37070000	8.38184700	0.82920400	C	6.56032400	7.60338700	0.32875400
C	5.65370400	5.76110600	-0.00440100	C	6.45692600	4.92909600	-0.36614500
H	3.53112400	5.61103600	0.19986800	H	4.34004400	5.08590900	-0.10959100
C	6.62550500	7.87745500	0.52941300	C	7.72493400	6.91624000	0.02771000
H	5.27244300	9.41474500	1.14448300	H	6.61235800	8.65456000	0.58995900
C	6.80689200	6.55916100	0.07788000	C	7.71040700	5.56287100	-0.34949500
H	5.74750700	4.72246100	-0.30123700	H	6.39980500	3.87351400	-0.60792700
H	7.48757600	8.52378000	0.64983400	H	8.67081800	7.44244300	0.09138600
B	1.04676700	-9.71756600	1.55865900	B	-0.47748400	-9.62202100	1.72952200
B	8.20640600	5.98703300	-0.31487900	B	9.00624600	4.77672200	-0.72575600
C	8.40918500	4.42512800	-0.36946700	C	8.99414100	3.20304200	-0.64038300
C	9.07194000	3.70484000	0.63146300	C	9.51921300	2.49688700	0.44822100
C	7.92216300	3.69661700	-1.46259100	C	8.43895100	2.44924700	-1.68220900
C	9.24277700	2.32937500	0.54517800	C	9.49322300	1.10906200	0.49464300
H	9.45212000	4.22202800	1.50733800	H	9.94686700	3.03775100	1.28717400
C	8.07937600	2.32147600	-1.54758900	C	8.39687000	1.06403900	-1.63347300
H	7.41493500	4.21365200	-2.27180100	H	8.03429300	2.95247500	-2.55544500
C	8.74389900	1.61177400	-0.54423400	C	8.92415800	0.36733300	-0.54306800
H	9.74341400	1.79986500	1.34913200	H	9.89136600	0.59442500	1.36297200
H	7.70382100	1.79170100	-2.41666500	H	7.97222500	0.51121500	-2.46477000
C	8.90001900	0.14633900	-0.64063000	C	8.86816600	-1.10786400	-0.50034700

C	7.83395200	-0.65247800	-1.06535400	C	7.71280200	-1.78365400	-0.90444000
C	10.10889900	-0.48004500	-0.32087300	C	9.96113100	-1.86732400	-0.07013800
C	7.97113600	-2.02792500	-1.15312600	C	7.65347400	-3.16708800	-0.87171800
H	6.88070700	-0.18898500	-1.29611800	H	6.84452300	-1.21360600	-1.21734400
C	10.25327200	-1.85239500	-0.45798000	C	9.91004500	-3.25298900	-0.08394300
H	10.95201500	0.12221000	0.00107800	H	10.87087300	-1.36369500	0.23960100
C	9.18851000	-2.66819300	-0.87216500	C	8.75523300	-3.94505100	-0.48201800
H	7.11694500	-2.62292800	-1.45882600	H	6.73451800	-3.66127900	-1.16882600
H	11.21305200	-2.30517500	-0.23104400	H	10.78584700	-3.81397200	0.22589700
C	2.35382600	-8.97460800	1.12246100	C	0.94937000	-9.07496700	1.37403600
C	3.35445200	-8.67295800	2.05880200	C	1.92199300	-8.86397800	2.36634600
C	2.56207600	-8.53747300	-0.19466600	C	1.31784600	-8.74779900	0.05781000
C	4.47487600	-7.93356900	1.71334800	C	3.16033700	-8.31461300	2.07448900
H	3.24068500	-9.00775600	3.08466100	H	1.69056200	-9.11965000	3.39548000
C	3.70750600	-7.84710000	-0.56000300	C	2.57316100	-8.24823400	-0.25074400
H	1.80912200	-8.74351700	-0.94862600	H	0.59994300	-8.89375000	-0.74330600
C	4.67191300	-7.51177600	0.39536900	C	3.51134200	-8.00107900	0.75711100
H	5.22190000	-7.70030000	2.46466700	H	3.87933800	-8.14933700	2.87044700
H	3.83773500	-7.52013100	-1.58624300	H	2.82035100	-8.00470200	-1.27908100
C	5.85510800	-6.70662800	0.03065600	C	4.82198400	-7.40245300	0.44111900
C	6.59719000	-6.98283900	-1.12147000	C	5.58424800	-7.85204800	-0.64235000
C	6.24885700	-5.63064200	0.83197100	C	5.32612700	-6.34891000	1.21157300
C	7.71043000	-6.22098500	-1.44423700	C	6.81529100	-7.28158300	-0.92762300
H	6.31510600	-7.82166700	-1.74916500	H	5.21829800	-8.67778500	-1.24354100
C	7.32738300	-4.83932100	0.47151100	C	6.52827400	-5.74412900	0.88307700
H	5.67464200	-5.39043500	1.72046100	H	4.74285200	-5.97517500	2.04646300
C	8.09811500	-5.11847800	-0.66752200	C	7.31537700	-6.19928000	-0.18664100
H	8.28472400	-6.47685700	-2.32880700	H	7.39682000	-7.67204500	-1.75668400
H	7.58397100	-3.98555700	1.09008500	H	6.87078900	-4.89950100	1.47221600
B	9.32100100	-4.21651100	-1.04278500	B	8.67115400	-5.50563100	-0.53964500
C	-1.26695700	11.44785700	1.76224900	C	0.39889700	11.51129000	1.47937600
C	-0.28347300	12.38698400	1.41014500	C	1.44916300	12.32576900	1.02489900
C	-2.40139500	11.92729500	2.43795200	C	-0.60423600	12.12506200	2.24760100
C	-0.42715700	13.73519200	1.70797400	C	1.49153000	13.68354600	1.31090000
H	0.60129400	12.05396700	0.87802200	H	2.23780400	11.88685000	0.42289600
C	-2.53989400	13.26955200	2.76324000	C	-0.55478600	13.47652000	2.56086500
H	-3.18165200	11.22933700	2.72269400	H	-1.43023900	11.52556200	2.61552700
C	-1.55374800	14.17731900	2.39275300	C	0.49172600	14.25985200	2.08668200
H	0.33992800	14.44194500	1.41040100	H	2.30578000	14.29290600	0.93377800
H	-3.41882300	13.61073100	3.29946300	H	-1.33448900	13.92189200	3.16930300
H	-1.66412700	15.22881100	2.63569900	H	0.52747500	15.31855500	2.32054800
C	9.39966300	6.91467800	-0.71948500	C	10.29916700	5.49365700	-1.23442400
C	9.19531600	8.15440200	-1.34815200	C	10.25062800	6.71035800	-1.93526500
C	10.72939100	6.50800200	-0.52198500	C	11.56270200	4.90788500	-1.05227900
C	10.26005800	8.95280700	-1.74413700	C	11.40283700	7.31795600	-2.41567700
H	8.18417500	8.48747700	-1.55428000	H	9.29073100	7.17607800	-2.12936300
C	11.79805400	7.31484700	-0.88420700	C	12.72136100	5.52476500	-1.50082500
H	10.92773800	5.53967300	-0.07536700	H	11.63519900	3.95048700	-0.54739200
C	11.56453200	8.54004900	-1.49966800	C	12.64246900	6.73204000	-2.18691700
H	10.07303800	9.89790200	-2.24236700	H	11.33531400	8.24929600	-2.96740800
H	12.81461900	6.98294300	-0.70315800	H	13.68568600	5.05828500	-1.33108600
H	12.39836200	9.16607000	-1.79882300	H	13.54516500	7.20947300	-2.55283100
C	10.63714800	-4.85422000	-1.59173600	C	9.90737600	-6.35903800	-0.97182700
C	11.49960200	-4.14179800	-2.44149600	C	10.89964700	-5.84200100	-1.82110800
C	11.00431300	-6.16819200	-1.25687000	C	10.06793800	-7.68145900	-0.52584000
C	12.66254900	-4.71263000	-2.93984000	C	11.99035100	-6.60595200	-2.21310100
H	11.24295400	-3.12706800	-2.72659800	H	10.80344600	-4.82692400	-2.19154800
C	12.17763600	-6.73770900	-1.73161600	C	11.16802000	-8.44381400	-0.89376800
H	10.36183500	-6.74627400	-0.60109800	H	9.32018000	-8.11199400	0.13182300
C	13.00637700	-6.01101000	-2.57956900	C	12.12927100	-7.90756200	-1.74364100
H	13.30390300	-4.14567200	-3.60594800	H	12.73485600	-6.18718000	-2.88162800
H	12.44514000	-7.74935900	-1.44615700	H	11.27489500	-9.45765100	-0.52331200
H	13.91921500	-6.45672700	-2.96024500	H	12.98544500	-8.50390100	-2.04074200
C	1.13866000	-10.90419200	2.57399900	C	-0.60601400	-10.78701200	2.77019200
C	2.25972800	-11.75034800	2.59171300	C	0.40144500	-11.76136400	2.89219800
C	0.12213500	-11.16462000	3.50755400	C	-1.70605800	-10.90597900	3.63880000
C	2.35231700	-12.81571300	3.47674100	C	0.30825800	-12.80275000	3.80489700
H	3.06712800	-11.57303800	1.88890600	H	1.27123400	-11.70175000	2.24599000
C	0.22012600	-12.21086600	4.41485800	C	-1.79904000	-11.93386400	4.56710300
H	-0.75204200	-10.52276200	3.53290400	H	-2.49512400	-10.16236700	3.59658800
C	1.33297100	-13.04427100	4.39458600	C	-0.79358100	-12.89165200	4.64934100
H	3.22196000	-13.46370300	3.45787400	H	1.09772500	-13.54496200	3.86258800
H	-0.57204100	-12.37990300	5.13628500	H	-2.65565700	-11.98817200	5.23108100
H	1.40746800	-13.86878200	5.09563400	H	-0.86608300	-13.69943600	5.36990300
C	-9.14735100	-6.82892800	-0.90025700	C	-9.87364400	-5.45367000	-1.96144300
C	-10.27425200	-6.57821900	-0.11535300	C	-11.13198100	-5.06061000	-1.49484100
C	-9.13607700	-7.93911800	-1.74600000	C	-9.75912300	-6.51127500	-2.86892100
C	-11.37475200	-7.42066300	-0.18836300	C	-12.26370700	-5.72239000	-1.94112900
H	-10.28254300	-5.72244800	0.54955600	H	-11.21052600	-4.25820800	-0.77167300
C	-10.23370100	-8.78632100	-1.79987900	C	-10.89949200	-7.16478800	-3.30531800
H	-8.26246100	-8.13190600	-2.35786500	H	-8.78178700	-6.79641900	-3.23856900
C	-11.36005000	-8.52963600	-1.02659100	C	-12.15325900	-6.77416500	-2.84554900
H	-12.24397700	-7.21424600	0.42627400	H	-13.23673800	-5.42350700	-1.56962900
H	-10.21155800	-9.64531300	-2.46133700	H	-10.80906200	-7.97662600	-4.01727500
H	-12.21902500	-9.18895600	-1.07581200	H	-13.04219900	-7.28925500	-3.18997700
C	-10.66097000	4.70899400	-1.15928800	C	-10.02537500	6.02258600	0.03316700
C	-11.84003700	3.94494600	-1.20625900	C	-11.23580500	5.43419100	0.44364700
C	-10.77919100	6.08413900	-1.42415600	C	-10.04606600	7.40838300	-0.20605500
C	-13.06533300	4.51561300	-1.52083900	C	-12.39991900	6.17668700	0.58382300
H	-11.79671900	2.88711900	-0.97303000	H	-11.25768800	4.37689700	0.68367600
C	-11.99640000	6.65926900	-1.75775500	C	-11.21047600	8.15326100	-0.09241300
H	-9.89773500	6.71281400	-1.36510000	H	-9.12682700	7.91095600	-0.48655400
C	-13.14324000	5.87384200	-1.80716600	C	-12.39402800	7.53864700	0.30349600
H	-13.96050900	3.90360400	-1.54089200	H	-13.31337900	5.69408300	0.91498600
H	-12.05594600	7.72155700	-1.96819300	H	-11.19465500	9.21810200	-0.29913800
H	-14.09867600	6.32254900	-2.05747600	H	-13.30364100	8.12078300	0.40564600
N	-8.01276100	-5.98397400	-0.83513200	N	-8.71255300	-4.79241900	-1.50986100

Structure S40. The coordinates of both the optimized structures of B4N2L$_2$ at S$_0$ and S$_1$ in toluene.

S_0-optimized structure				S_1-optimized structure			
C	-8.89390100	3.24370100	0.03576100	C	-6.80931500	6.18446100	0.64243900
C	-8.70714100	3.87852200	1.26311800	C	-6.21727000	6.61258000	1.82856300
C	-8.12011400	3.63067800	-1.05629500	C	-6.14900700	6.37949600	-0.56898300
C	-7.75420600	4.87778700	1.39448300	C	-4.97114800	7.21848200	1.79998400
H	-9.31602600	3.58491300	2.11084700	H	-6.73618200	6.46565000	2.76877500
C	-7.18300200	4.64427800	-0.92539300	C	-4.91063400	7.00134700	-0.59070900
H	-8.26341600	3.13353200	-2.00893600	H	-6.61286300	6.04420000	-1.48961000
C	-6.98303800	5.28170000	0.30133400	C	-4.29881100	7.42649700	0.59192000
H	-7.61791400	5.36712200	2.35282700	H	-4.51777000	7.55214400	2.72667400
H	-6.58607200	4.93798600	-1.78196600	H	-4.40059400	7.14452800	-1.53676300
C	-5.98226100	6.36368500	0.43210200	C	-2.97226700	8.07691400	0.56429200
C	-4.97919000	6.30284900	1.40306100	C	-1.95540100	7.66166200	1.42854600
C	-6.01692800	7.46931800	-0.42145600	C	-2.70344900	9.11596200	-0.33081300
C	-4.01959200	7.30080400	1.48867000	C	-0.69998900	8.24722900	1.37198100
H	-4.93194200	5.44558600	2.06650000	H	-2.14228200	6.84674300	2.12005300
C	-5.09583500	8.49694000	-0.28333600	C	-1.46420000	9.73827800	-0.33714300
H	-6.79534000	7.53580300	-1.17439700	H	-3.48530800	9.45963200	-1.00000300
C	-4.06146700	8.43313200	0.66182800	C	-0.42333600	9.31185300	0.50115700
H	-3.22386200	7.20526200	2.22069900	H	0.08380300	7.87635100	2.02444200
H	-5.17035600	9.36248100	-0.93382400	H	-1.29421800	10.56615600	-1.01778300
C	-9.38450200	0.89381900	-0.31343500	C	-8.20855200	4.24443800	0.23695900
C	-8.44695100	0.34266400	0.55889700	C	-7.11623700	3.36238500	0.32349500
C	-9.83301700	0.11920800	-1.38464400	C	-9.41627800	3.75540100	-0.29566300
C	-7.99205100	-0.95239600	0.37837500	C	-7.24731500	2.04569600	-0.05409900
H	-8.09165500	0.92910700	1.39842400	H	-6.17052900	3.71737400	0.71223700
C	-9.36587800	-1.17242000	-1.56302700	C	-9.52634700	2.44192800	-0.69323700
H	-10.54774400	0.53986100	-2.08292900	H	-10.26516900	4.41979900	-0.39470900
C	-8.44120400	-1.74831400	-0.68188000	C	-8.45566300	1.53729900	-0.56515600
H	-7.30107400	-1.36065500	1.10632700	H	-6.38427000	1.39602900	0.02998000
H	-9.71191000	-1.73230700	-2.42420300	H	-10.47757700	2.09729400	-1.08194200
C	-7.97381600	-3.13994800	-0.84349200	C	-8.59295700	0.13524100	-0.91731800
C	-6.77374000	-3.58112200	-0.27577700	C	-7.82875900	-0.85471700	-0.26556800
C	-8.71962600	-4.09336900	-1.54604100	C	-9.50002000	-0.30256700	-1.90616700
C	-6.35681400	-4.89722100	-0.36711100	C	-7.96164900	-2.18689200	-0.56354900
H	-6.13808000	-2.88363800	0.25694500	H	-7.15855100	-0.57040200	0.53630900
C	-8.29613600	-5.40425800	-1.67790400	C	-9.63345700	-1.62999800	-2.22971300
H	-9.66763400	-3.81736100	-1.99300600	H	-10.06979400	0.42740100	-2.46854000
C	-7.11133200	-5.84043400	-1.07339500	C	-8.87220300	-2.61365200	-1.55717100
H	-5.43416600	-5.19298100	0.11544800	H	-7.39737700	-2.92328600	-0.00762900
H	-8.90315100	-6.10462200	-2.23809300	H	-10.29827800	-1.92371500	-3.03150200
B	-2.98921000	9.56867500	0.77333800	B	0.99254500	9.98096100	0.46287300
C	-5.36689700	-7.55824900	-0.96941100	C	-7.92400300	-4.83555600	-1.57512100
C	-5.05032900	-8.63399100	-0.13675700	C	-8.15816700	-6.01244300	-0.84902600
C	-4.32431600	-6.86602100	-1.59190400	C	-6.61386300	-4.53379400	-1.97489700
C	-3.73013100	-8.98205200	0.09016400	C	-7.10835000	-6.82447200	-0.48715700
H	-5.84540100	-9.18118500	0.35535300	H	-9.16678000	-6.25606900	-0.53455900
C	-3.00619400	-7.20821400	-1.34191500	C	-5.57087200	-5.35520500	-1.61409400
H	-4.55319200	-6.05045800	-2.26776100	H	-6.42879300	-3.65477100	-2.58262000
C	-2.67713700	-8.26584800	-0.48694700	C	-5.76515700	-6.51745900	-0.82521700
H	-3.51277100	-9.79318800	0.77603700	H	-7.32128000	-7.69310400	0.12275400
H	-2.21708900	-6.65781600	-1.84231100	H	-4.57811900	-5.10796400	-1.96762800
C	-1.47588300	9.18253200	0.84883300	C	2.26232000	9.06970400	0.49051000
C	-1.00328200	8.07149900	0.13382600	C	2.25297300	7.82771300	-0.16281100
C	-0.52026400	9.93783600	1.54715900	C	3.46452600	9.44998800	1.10805800
C	0.34629300	7.76669000	0.06789600	C	3.38620900	7.03540000	-0.23959600
H	-1.70747700	7.45517400	-0.41485500	H	1.34450700	7.49162300	-0.65135200
C	0.82678400	9.61448700	1.51938600	C	4.58991200	8.64227100	1.07215700
H	-0.84185900	10.79225900	2.13355200	H	3.51331300	10.39327700	1.64203000
C	1.29067100	8.53666100	0.75528300	C	4.57943300	7.42675800	0.37754100
H	0.67465000	6.93991800	-0.55162100	H	3.35349900	6.11355200	-0.80906600
H	1.52832100	10.20338000	2.09978200	H	5.48645900	8.95433100	1.59605900
C	-1.27693600	-8.58555700	-0.15102400	C	-4.65565800	-7.31854200	-0.35625800
C	-0.32958000	-7.56736100	0.00074200	C	-3.32770900	-6.82341900	-0.35390800
C	-0.86486300	-9.90416200	0.07845000	C	-4.83156900	-8.61900700	0.17906300
C	0.96683500	-7.85635200	0.39371800	C	-2.28711600	-7.54459700	0.17918700
H	-0.62546400	-6.53481500	-0.14961200	H	-3.12442600	-5.82564100	-0.72667600
C	0.42666500	-10.18239200	0.49405900	C	-3.78390400	-9.33091800	0.71233900
H	-1.56597100	-10.71764300	-0.07449900	H	-5.81044000	-9.08518600	0.14774100
C	1.38093700	-9.16774600	0.67933000	C	-2.45469200	-8.82716800	0.76922700
H	1.66842400	-7.03934700	0.52297100	H	-1.30117300	-7.09212200	0.19141600
H	0.70777000	-11.21483700	0.67303400	H	-3.97975900	-10.32865100	1.09131700
C	2.73203300	8.23865100	0.63235300	C	5.79099000	6.59009600	0.26251600
C	3.18596000	6.93176800	0.41917600	C	5.69748800	5.19905700	0.13803700
C	3.68669300	9.26088400	0.68748600	C	7.06771900	7.16362700	0.24520500
C	4.53043300	6.67110000	0.22017000	C	6.83020600	4.42496100	-0.04250900
H	2.47937500	6.10955000	0.42472200	H	4.72844600	4.71723100	0.20185000
C	5.02959000	8.99202100	0.48267300	C	8.19594400	6.38194100	0.06046300
H	3.36898000	10.28410600	0.85287800	H	7.17527700	8.23827000	0.33997300
C	5.49351500	7.69422700	0.20822500	C	8.11372000	4.99100400	-0.12161200
H	4.84496400	5.64581200	0.06043200	H	6.71937600	3.35007400	-0.13169400
H	5.73627500	9.81246500	0.53229900	H	9.16641500	6.86477600	0.05403700
B	2.80756700	-9.44239900	1.25203700	B	-1.29521600	-9.55893300	1.46499500
B	6.98994000	7.37924300	-0.10676800	B	9.36194100	4.09548500	-0.40333400
C	7.48049800	5.88154000	-0.09408000	C	9.22312100	2.53028100	-0.28442800
C	8.23583700	5.31417200	0.93863300	C	9.67927300	1.78251600	0.80704400
C	7.16863200	5.05652000	-1.18295200	C	8.61888000	1.82419200	-1.33307900
C	8.66347900	3.99369000	0.88512700	C	9.54370800	0.40050400	0.84838300
H	8.48620400	5.90631400	1.81372200	H	10.13763500	2.28543200	1.65333900
C	7.58532000	3.73545800	-1.23696300	C	8.47267300	0.44640500	-1.29208700
H	6.59413800	5.45545800	-2.01375900	H	8.26012400	2.36021900	-2.20692600
C	8.34282800	3.17892500	-0.20312000	C	8.93311000	-0.29285300	-0.19922800
H	9.22973500	3.58006500	1.71310500	H	9.88945300	-0.14549000	1.71999600
H	7.34170000	3.13226900	-2.10512700	H	8.01654700	-0.06926300	-2.13044400
C	8.77967100	1.76992500	-0.27368800	C	8.76623800	-1.75997600	-0.16981400
C	7.90619600	0.78073100	-0.73603200	C	7.57597600	-2.34453100	-0.61293500

C	10.07205700	1.38728000	0.10027500	C	9.78815800	-2.60317500	0.27865800
C	8.31154400	-0.54114800	-0.81563700	C	7.41636500	-3.72034400	-0.60549600
H	6.89116800	1.05064700	-1.00717600	H	6.75971700	-1.70931400	-0.94011600
C	10.48468600	0.06935600	-0.02476000	C	9.63617800	-3.98120000	0.24343800
H	10.76933900	2.13960000	0.45368800	H	10.72408100	-2.17214200	0.61855500
C	9.61771300	-0.93492400	-0.48411100	C	8.44688200	-4.58244100	-0.19841900
H	7.60298600	-1.28825000	-1.15730000	H	6.47359900	-4.14220300	-0.93722400
H	11.50349900	-0.18982400	0.24448900	H	10.45992600	-4.60852400	0.56850400
C	3.97842800	-8.45691900	0.92117200	C	0.19230800	-9.13319700	1.18042900
C	4.85533300	-8.02543400	1.92795200	C	1.11855000	-8.97226100	2.22674200
C	4.17421300	-7.92829400	-0.36374700	C	0.67336900	-8.86799000	-0.11439600
C	5.83950900	-7.08141600	1.67969400	C	2.41048300	-8.52308900	2.00722900
H	4.74913200	-8.42378400	2.93169600	H	0.80478200	-9.18777500	3.24344100
C	5.19431900	-7.02847200	-0.63248000	C	1.97837500	-8.46461900	-0.34987300
H	3.51262000	-8.22995900	-1.16952800	H	0.00090000	-8.98467800	-0.95879100
C	6.02835800	-6.57103800	0.39238600	C	2.86842100	-8.26129700	0.71071500
H	6.48955400	-6.75299900	2.48376300	H	3.08754900	-8.39600700	2.84625000
H	5.31989200	-6.63631200	-1.63625200	H	2.30584900	-8.26317300	-1.36514800
C	7.06405700	-5.55145200	0.12993400	C	4.23348600	-7.76058800	0.47199100
C	7.91070500	-5.64311100	-0.97844100	C	5.02151100	-8.25662700	-0.57334400
C	7.20559900	-4.45435200	0.98508300	C	4.77284800	-6.75094000	1.27858200
C	8.88074700	-4.67853500	-1.20790900	C	6.30295000	-7.77405100	-0.78776900
H	7.82515000	-6.49439200	-1.64563300	H	4.63100600	-9.05065500	-1.20120200
C	8.13765000	-3.46522900	0.71606200	C	6.03043800	-6.23218900	1.02069600
H	6.54646300	-4.35856200	1.84133500	H	4.17403000	-6.34092700	2.08497300
C	9.01033800	-3.55353800	-0.37930200	C	6.84012800	-6.73372700	-0.01189100
H	9.54306600	-4.79054900	-2.06026000	H	6.89766300	-8.19952000	-1.58995700
H	8.19476900	-2.60319600	1.37268000	H	6.40041600	-5.41876400	1.63681700
B	10.05665400	-2.42441900	-0.66342200	B	8.25238000	-6.13266600	-0.29438000
C	-3.43715300	11.06490200	0.75126400	C	1.12097300	11.53397900	0.36248400
C	-2.59722600	12.08476400	0.27365000	C	2.23408100	12.14483500	-0.23887100
C	-4.71204300	11.44139700	1.20616400	C	0.11874200	12.37845200	0.86885200
C	-3.00876700	13.41006700	0.24538800	C	2.33888900	13.52531100	-0.33768400
H	-1.61096800	11.82909000	-0.09850700	H	3.02205100	11.52318100	-0.65031300
C	-5.12140600	12.76747700	1.20460800	C	0.22836200	13.76005300	0.79673100
H	-5.38731300	10.67813400	1.57814400	H	-0.75493300	11.93979100	1.33924300
C	-4.27027300	13.75408300	0.71869600	C	1.33808000	14.33554300	0.18738100
H	-2.34652200	14.17641700	-0.14266600	H	3.20159400	13.97164300	-0.82028300
H	-6.10539100	13.03314300	1.57550200	H	-0.55222300	14.38973100	1.20974600
H	-4.59115600	14.79024100	0.70621000	H	1.42218400	15.41496000	0.12023900
C	8.00807300	8.49245000	-0.52516000	C	10.72782200	4.68828100	-0.88568700
C	7.61916600	9.60716900	-1.28625800	C	10.79901400	5.81475100	-1.72177000
C	9.37054800	8.37962800	-0.20458400	C	11.93942500	4.06595000	-0.54437900
C	8.53782100	10.56612700	-1.69239400	C	12.01530500	6.30318400	-2.18063900
H	6.58326300	9.71154100	-1.58962200	H	9.88417300	6.30108200	-2.04207700
C	10.28793300	9.35187500	-0.57550500	C	13.16038500	4.56942500	-0.96890700
H	9.71711300	7.51202700	0.34641800	H	11.92284000	3.16942600	0.06606600
C	9.87271800	10.44778400	-1.32443800	C	13.20028000	5.68941400	-1.79251500
H	8.21226200	11.40750900	-2.29449300	H	12.03937500	7.16412000	-2.83995200
H	11.33103100	9.24785100	-0.29712500	H	14.08156200	4.07866900	-0.67404800
H	10.59059900	11.20087200	-1.63112100	H	14.15242600	6.07500800	-2.14080000
C	11.49860400	-2.78566300	-1.14404400	C	9.44288300	-7.06087400	-0.70426500
C	12.25134400	-1.90500700	-1.93856500	C	10.49384000	-6.60049200	-1.51458000
C	12.09289300	-4.01189500	-0.80242800	C	9.50176300	-8.39680900	-0.27387000
C	13.52645000	-2.23332200	-2.37782400	C	11.54328600	-7.43029900	-1.88499000
H	11.81984400	-0.95298300	-2.22877400	H	10.47714100	-5.57633500	-1.87207100
C	13.37687900	-4.33717600	-1.21715800	C	10.55981400	-9.22615700	-0.61991800
H	11.53938200	-4.71547000	-0.18953300	H	8.70676200	-8.78500500	0.35402900
C	14.09389600	-3.44881100	-2.01130100	C	11.58084400	-8.74453300	-1.43188200
H	14.08075100	-1.54149300	-3.00280400	H	12.33455800	-7.05315100	-2.52403800
H	13.81810700	-5.28464700	-0.92718800	H	10.58701900	-10.24977200	-0.26207100
H	15.09390500	-3.70422200	-2.34537500	H	12.40430900	-9.39287500	-1.71212400
C	3.07239000	-10.65102700	2.20957800	C	-1.54460100	-10.69853800	2.52070700
C	4.32675100	-11.28272700	2.23610200	C	-0.63953400	-11.76751300	2.65939500
C	2.08694000	-11.14249100	3.08137700	C	-2.64969900	-10.70351100	3.39191600
C	4.57870900	-12.36403100	3.06918600	C	-0.83411700	-12.78543300	3.58292500
H	5.11373800	-10.92300300	1.58147500	H	0.23444000	-11.80009800	2.01631100
C	2.34000700	-12.20532800	3.93806700	C	-2.84596300	-11.70928600	4.32904100
H	1.11105600	-10.66915000	3.10011300	H	-3.36079300	-9.88524200	3.34320400
C	3.58519700	-12.82402500	3.92652200	C	-1.94108200	-12.76155400	4.42537100
H	5.55142100	-12.84378300	3.05754800	H	-0.11914200	-13.59949300	3.64953900
H	1.56619500	-12.55457300	4.61320400	H	-3.70562300	-11.67109900	4.99088200
H	3.78253500	-13.66107800	4.58773800	H	-2.09380100	-13.55160700	5.15320100
C	-7.68141700	-8.21009000	-1.27538900	C	-10.16668300	-4.48169400	-2.42995300
C	-8.80129500	-8.23335700	-0.44254500	C	-11.42347800	-4.09199700	-1.95627600
C	-7.50503200	-9.22750800	-2.21389700	C	-10.06889300	-5.44210300	-3.44063900
C	-9.73432000	-9.25422400	-0.55955500	C	-12.56784300	-4.64442400	-2.50918500
H	-8.93627100	-7.44929700	0.29333000	H	-11.49462600	-3.37924500	-1.14339900
C	-8.43363100	-10.25400600	-2.31301000	C	-11.22068800	-5.98679600	-3.98391000
H	-6.63638300	-9.20672000	-2.86191600	H	-9.09195000	-5.74393600	-3.79761600
C	-9.55454200	-10.27054800	-1.49098900	C	-12.47287200	-5.59017600	-3.52461400
H	-10.60040800	-9.26100200	0.09299900	H	-13.53896800	-4.34637600	-2.13134100
H	-8.28465800	-11.03849100	-3.04666400	H	-11.13903600	-6.72336100	-4.77466300
H	-10.28228900	-11.06951400	-1.57522900	H	-13.37042300	-6.02311100	-3.95029100
C	-11.22121600	2.49416900	0.00598200	C	-9.17973900	6.28920500	1.17568400
C	-12.14311000	1.49272400	0.33887500	C	-10.11260600	5.68327200	2.02147800
C	-11.69618800	3.79638900	-0.19864900	C	-9.31197600	7.64438800	0.86607800
C	-13.49321000	1.78956400	0.44931800	C	-11.17069900	6.42205900	2.52934300
H	-11.79802900	0.48195300	0.51742800	H	-9.99900500	4.63890000	2.28698600
C	-13.04784100	4.08018100	-0.07165000	C	-10.36647800	8.37679900	1.39078500
H	-11.00255300	4.58536900	-0.46193900	H	-8.58817400	8.11733600	0.21315800
C	-13.96093000	3.08289800	0.24859000	C	-11.30374800	7.77086400	2.21928000
H	-14.18511100	0.99549100	0.70933300	H	-11.88601600	5.94162900	3.18732300
H	-13.38839800	5.09695100	-0.23619300	H	-10.45877100	9.42785900	1.14166600
H	-15.01670600	3.30922700	0.34130400	H	-12.12798100	8.34589700	2.62468200
N	-6.71149800	-7.18233400	-1.16650800	N	-8.98809700	-3.94881200	-1.86389500
N	-9.85738500	2.21049800	-0.11167000	N	-8.08560000	5.55661600	0.66263100

Structure S41. The coordinates of both the optimized structures of B3N3L$_2$ at S$_0$ and S$_1$ in toluene.

S_0-optimized structure				S_1-optimized structure			
C	-9.20293000	1.51420300	-0.03918400	C	-6.49808100	6.37388000	0.62326000
C	-9.36000800	2.28815700	1.11142100	C	-5.92604000	6.86678800	1.79440300
C	-8.28268000	1.91707700	-1.00640400	C	-5.79138700	6.45585800	-0.57534900
C	-8.61436200	3.44420100	1.28361900	C	-4.65887000	7.42646100	1.76397300
H	-10.07527600	1.98076000	1.86575500	H	-6.47843900	6.80923600	2.72514900
C	-7.54782200	3.07933200	-0.83302200	C	-4.52862300	7.02575500	-0.59849000
H	-8.15227100	1.31437400	-1.89792500	H	-6.23746500	6.07226100	-1.48582700
C	-7.70191100	3.86453000	0.31206500	C	-3.93792700	7.51860700	0.56909100
H	-8.75398300	4.04141500	2.17829000	H	-4.22642000	7.81451600	2.67949200
H	-6.83576600	3.37984900	-1.59399900	H	-3.98398200	7.07607500	-1.53469900
C	-6.92897500	5.11444800	0.48027300	C	-2.58932700	8.12072400	0.54012300
C	-6.19546700	5.36057600	1.64369100	C	-1.62769600	7.77634100	1.49423500
C	-6.91889200	6.08278200	-0.52771600	C	-2.23780400	9.05033400	-0.44276100
C	-5.46856600	6.53261900	1.79372700	C	-0.36066900	8.33586100	1.46765700
H	-6.18233400	4.61714100	2.43331600	H	-1.87124400	7.04920900	2.26125100
C	-6.20657100	7.26230600	-0.37686300	C	-0.97458900	9.61844700	-0.47234000
H	-7.49101500	5.91428800	-1.43358600	H	-2.96931200	9.34340600	-1.18802000
C	-5.47270600	7.49369300	0.78519400	C	-0.02063400	9.26516800	0.48342900
H	-4.89350200	6.71264200	2.69493300	H	0.37605500	8.05478500	2.21120100
H	-6.21494500	8.01348100	-1.15858600	H	-0.71997100	10.34214800	-1.23782800
C	-9.25421000	-0.88283000	-0.40393400	C	-7.97608800	4.48557300	0.24449000
C	-8.27104000	-1.26036600	0.50997700	C	-6.92713000	3.55553700	0.35976700
C	-9.52136500	-1.72667300	-1.48313700	C	-9.20002800	4.04265200	-0.29110100
C	-7.59120500	-2.45670700	0.35840500	C	-7.11339900	2.24096900	-0.00152200
H	-8.05854600	-0.61926400	1.35788600	H	-5.97107100	3.87279800	0.75573400
C	-8.82968300	-2.91734600	-1.63195700	C	-9.36582700	2.72938600	-0.66946300
H	-10.27109300	-1.43870800	-2.21127900	H	-10.01735200	4.74265600	-0.40853600
C	-7.85259900	-3.32018900	-0.71218400	C	-8.33749100	1.77992100	-0.52030300
H	-6.86989600	-2.73836500	1.11630800	H	-6.28156500	1.55461400	0.10169500
H	-9.03984000	-3.53087700	-2.50040300	H	-10.32870900	2.42226000	-1.06085000
C	-7.13973200	-4.60635100	-0.84764800	C	-8.53144900	0.38205700	-0.86256300
C	-5.89902300	-4.82397800	-0.23890900	C	-7.80933000	-0.63468200	-0.20392700
C	-7.67861700	-5.67766400	-1.56913100	C	-9.45272200	-0.02413100	-1.85188500
C	-5.24826200	-6.04271600	-0.31203900	C	-7.99342600	-1.96171900	-0.49765900
H	-5.41810500	-4.02320000	0.31049700	H	-7.13082500	-0.37429100	0.59909300
C	-7.02102300	-6.89012100	-1.68223000	C	-9.63933200	-1.34675700	-2.16897100
H	-8.64562800	-5.57557000	-2.04776400	H	-9.99002300	0.72518600	-2.42075100
C	-5.79616500	-7.10464300	-1.03990400	C	-8.91826300	-2.35643000	-1.49113800
H	-4.30261300	-6.16836000	0.19956200	H	-7.45918000	-2.71786600	0.06150100
H	-7.47239800	-7.68791400	-2.25894800	H	-10.31382900	-1.61745500	-2.97088500
C	-3.76359800	-8.46484400	-0.91024900	C	-8.05054500	-4.61072400	-1.51065700
C	-3.25456200	-9.46409700	-0.07756700	C	-8.32100200	-5.77970700	-0.78439700
C	-2.86718300	-7.58376600	-1.52167100	C	-6.73353800	-4.35458100	-1.91958300
C	-1.89391800	-9.55556700	0.15950200	C	-7.29824000	-6.63048400	-0.43400200
H	-3.93465100	-10.15494400	0.40612100	H	-9.33502700	-5.98651400	-0.46113200
C	-1.51028000	-7.66973400	-1.26131100	C	-5.71802600	-5.21435400	-1.57018900
H	-3.24176900	-6.82383600	-2.19730700	H	-6.52201600	-3.48110100	-2.52660200
C	-0.99242400	-8.64924100	-0.40677400	C	-5.94830500	-6.37249300	-0.78519200
H	-1.53151100	-10.31359200	0.84489900	H	-7.53573500	-7.49152000	0.17765300
H	-0.83607400	-6.97668700	-1.75251300	H	-4.71979000	-5.00271000	-1.93137900
C	-3.33290800	8.63928100	0.90411800	C	2.39335500	8.98463600	0.43383500
C	-2.68028700	7.65529700	0.15479500	C	2.35947600	7.78644500	-0.28450900
C	-2.54894400	9.55439600	1.61811800	C	3.56525400	9.31550500	1.12186800
C	-1.29860200	7.61727000	0.09287200	C	3.46773500	6.95951400	-0.32501400
H	-3.26198300	6.93486600	-0.40708300	H	1.46580300	7.51717300	-0.83464300
C	-1.16883900	9.51097300	1.54143700	C	4.67083700	8.48502100	1.06684100
H	-3.02989900	10.30213600	2.23718000	H	3.60316600	10.22769600	1.70539000
C	-0.50536600	8.55073800	0.76745800	C	4.65430200	7.28938100	0.33880500
H	-0.83012800	6.87148500	-0.53905800	H	3.42221100	6.05987300	-0.92785200
H	-0.59468500	10.22449600	2.12199900	H	5.55530700	8.75957000	1.63045800
C	0.44128500	-8.69572900	-0.06433300	C	-4.86528300	-7.21804000	-0.33318800
C	1.17501500	-7.51421900	0.08579500	C	-3.52143100	-6.76819500	-0.32996100
C	1.09889800	-9.91003000	0.16779600	C	-5.08324600	-8.52032500	0.18185900
C	2.50383200	-7.54772400	0.47467600	C	-2.50377000	-7.53261200	0.18657600
H	0.68521300	-6.55842000	-0.06469700	H	-3.28659000	-5.77174300	-0.68757900
C	2.42096200	-9.93399300	0.58039300	C	-4.05785200	-9.27624600	0.69776900
H	0.56709000	-10.84332700	0.01721000	H	-6.07764500	-8.95250600	0.14588600
C	3.16356600	-8.75461200	0.75925400	C	-2.71203300	-8.81847500	0.75636200
H	3.03554800	-6.61074400	0.60003100	H	-1.50299400	-7.11415100	0.20159900
H	2.89563500	-10.89285300	0.76021200	H	-4.28532700	-10.27362500	1.05962300
C	0.96124200	8.54161100	0.62426900	C	5.84183400	6.42144100	0.23792800
C	1.65978400	7.35364600	0.37204400	C	5.71316900	5.03899200	0.05353100
C	1.70421200	9.72670000	0.70293200	C	7.13696800	6.95115200	0.29850100
C	3.02710500	7.36250900	0.16158100	C	6.82794400	4.23543300	-0.10640200
H	1.12602100	6.40997600	0.35875000	H	4.72815600	4.58587400	0.05703500
C	3.07141700	9.72756800	0.48666000	C	8.24718600	6.14054300	0.13274300
H	1.19735500	10.66545500	0.89635600	H	7.27516100	8.01715900	0.44067700
C	3.77658900	8.55166400	0.17618000	C	8.13161900	4.76018600	-0.10608000
H	3.53182400	6.42127400	-0.02649900	H	6.68706100	3.16859500	-0.24004300
H	3.60669000	10.66780400	0.55568400	H	9.23139400	6.59174500	0.18795700
B	4.62189900	-8.74730100	1.31831800	B	-1.57308800	-9.59869300	1.43301500
B	5.30172300	8.54091300	-0.14525700	B	9.36293400	3.83633500	-0.35981000
C	6.08091100	7.17018800	-0.14747400	C	9.18189000	2.27342300	-0.26491000
C	6.93604300	6.76149000	0.88270500	C	9.59840400	1.50888400	0.83111500
C	5.94107800	6.30412500	-1.24002700	C	8.58498000	1.58687400	-1.33045100
C	7.62262400	5.55566600	0.82253200	C	9.43323800	0.12995700	0.86040100
H	7.06176200	7.38781800	1.76083800	H	10.04920200	1.99681400	1.69019100
C	6.61598100	5.09449100	-1.30030400	C	8.40840200	0.21207600	-1.30150800
H	5.29734400	6.58313300	-2.06899300	H	8.25695700	2.13582400	-2.20831500
C	7.47242600	4.69835300	-0.26987500	C	8.83080900	-0.54365700	-0.20466800
H	8.26244600	5.26144300	1.64803800	H	9.74935800	-0.42897700	1.73507500
H	6.49885200	4.45840300	-2.17127000	H	7.95886100	-0.28869700	-2.15240000
C	8.18774000	3.40864900	-0.34651800	C	8.63540100	-2.00754100	-0.18799100
C	7.53377000	2.26167300	-0.80688300	C	7.43986600	-2.56701800	-0.64882500
C	9.53258200	3.29903300	0.02213100	C	9.63599400	-2.87225500	0.26750200

C	8.20065500	1.05047100	-0.88700400
H	6.48403400	2.31806400	-1.07439100
C	10.20599600	2.09355500	-0.10475000
H	10.06178200	4.17872100	0.37309300
C	9.56086800	0.93258400	-0.55973300
H	7.65863800	0.17348800	-1.22485000
H	11.25728900	2.04855500	0.16062300
C	5.57626000	-7.55727000	0.96561500
C	6.36510900	-6.95347100	1.95646500
C	5.64654300	-7.01132300	-0.32507900
C	7.13928800	-5.83521900	1.68826900
H	6.35358100	-7.35649400	2.96389900
C	6.46408400	-5.92953200	-0.61397100
H	5.04637300	-7.44418500	-1.11902800
C	7.20422300	-5.30699700	0.39588400
H	7.72224400	-5.37768400	2.48060500
H	6.49508300	-5.52839600	-1.62155200
C	8.00885600	-4.10113400	0.11325800
C	8.84313500	-4.02767900	-1.00590300
C	7.93398300	-2.99105800	0.95997100
C	9.59360200	-2.88775100	-1.25382400
H	8.92447900	-4.88377800	-1.66748000
C	8.64145100	-1.83503900	0.67265400
H	7.27925900	-3.02432000	1.82432900
C	9.50115100	-1.75323900	-0.43338300
H	10.25480500	-2.86982000	-2.11421700
H	8.52937300	-0.97372800	1.32308700
B	10.29342800	-0.43704700	-0.73427700
C	-5.43362000	9.91832900	0.94400400
C	-4.92006100	11.05770900	0.31581100
C	-6.68594400	9.99631900	1.56147000
C	-5.63732500	12.24510500	0.32528700
H	-3.96076500	11.00960800	-0.18479000
C	-7.40127300	11.18471200	1.55051700
H	-7.09487400	9.12033200	2.05069900
C	-6.88218700	12.31957400	0.93910100
H	-5.22135800	13.11663300	-0.16851200
H	-8.37039900	11.22277000	2.03588600
H	-7.44099300	13.24821700	0.93815400
C	6.07982900	9.83885200	-0.55044100
C	5.47257100	10.86818900	-1.28862900
C	7.44131700	9.99040400	-0.24223000
C	6.18109200	11.99545900	-1.68347700
H	4.43315100	10.77240500	-1.58253300
C	8.14693500	11.12917300	-0.60259300
H	7.95694300	9.19902200	0.29098400
C	7.51730700	12.13495600	-1.32796300
H	5.69030700	12.76676300	-2.26718300
H	9.19326500	11.22662500	-0.33400600
H	8.07053800	13.01923200	-1.62570000
C	11.77643800	-0.50103900	-1.22106000
C	12.33259600	0.51003100	-2.02216700
C	12.60787000	-1.57988000	-0.87701400
C	13.64645000	0.44481500	-2.46516200
H	11.71630500	1.35360500	-2.31428500
C	13.92979100	-1.64037700	-1.29574900
H	12.21025800	-2.37793100	-0.25891600
C	14.44949300	-0.62890700	-2.09620700
H	14.04713700	1.23167800	-3.09505300
H	14.55463400	-2.47759700	-1.00400400
H	15.47933800	-0.67808000	-2.43359500
C	5.12296500	-9.87427100	2.28048100
C	6.47620100	-10.25042300	2.30146400
C	4.25698400	-10.54039700	3.16311600
C	6.93852900	-11.25484600	3.14054500
H	7.17418000	-9.75052600	1.63798300
C	4.71690000	-11.52652500	4.02539900
H	3.20784400	-10.26551800	3.18561400
C	6.05852200	-11.89147800	4.00890200
H	7.98578500	-11.53674800	3.12492900
H	4.02978700	-12.01356800	4.70892500
H	6.41866000	-12.66847700	4.67477400
C	-5.91199300	-9.53909800	-1.24713300
C	-7.03469700	-9.76593400	-0.44918300
C	-5.52211000	-10.50954200	-2.17111900
C	-7.75992300	-10.94133100	-0.58640800
H	-7.33509400	-9.01829600	0.27554700
C	-6.24235500	-11.68967200	-2.28983900
H	-4.65216000	-10.33115800	-2.79253600
C	-7.36693200	-11.91000800	-1.50293200
H	-8.63041500	-11.10539300	0.03910900
H	-5.92801500	-12.43524100	-3.01172600
H	-7.93226800	-12.82949100	-1.60250400
C	-11.35636400	0.36061300	-0.18845700
C	-12.08836300	-0.77321900	0.18503600
C	-12.05412400	1.52940500	-0.51767600
C	-13.47438500	-0.73403000	0.21827700
H	-11.56742100	-1.68360700	0.45483500
C	-13.43963800	1.55864600	-0.46501700
H	-11.50630700	2.41398700	-0.81877100
C	-14.16391000	0.42949900	-0.10150000
H	-14.01857600	-1.62526000	0.51205200
H	-13.95679200	2.47611200	-0.72488100
H	-15.24683900	0.45590700	-0.06792800
N	-5.15320700	-8.34972500	-1.11847800
N	-9.95654700	0.33127400	-0.23136800
N	-4.73234100	8.69734400	0.94122900
C	7.25382800	-3.93964200	-0.64947700
H	6.63992500	-1.91462500	-0.98251600
C	9.45877400	-4.24697700	0.22261100
H	10.57559600	-2.46057600	0.62097200
C	8.26317300	-4.82304000	-0.23516000
H	6.30670200	-4.34197800	-0.99299200
H	10.26686100	-4.89153000	0.55328400
C	-0.07510100	-9.21230100	1.14486700
C	0.85975300	-9.08318000	2.18778800
C	0.40591100	-8.94757800	-0.15000800
C	2.16059300	-8.66149600	1.96572800
H	0.54595600	-9.30039500	3.20413700
C	1.71881400	-8.57256300	-0.38862500
H	-0.27342900	-9.04054200	-0.99186700
C	2.61791800	-8.39816300	0.66935700
H	2.84435500	-8.55679700	2.80241900
H	2.04600200	-8.36962900	-1.40370600
C	3.99180400	-7.92305000	0.42839600
C	4.76646700	-8.42779300	-0.62269900
C	4.55287500	-6.92840000	1.23869600
C	6.05629300	-7.96869200	-0.83870400
H	4.35853900	-9.21031900	-1.25391900
C	5.81912900	-6.43200900	0.97919900
H	3.96455400	-6.51167300	2.04936200
C	6.61577800	-6.94346200	-0.05858800
H	6.64010700	-8.40093300	-1.64528800
H	6.20647800	-5.62898800	1.59826900
B	8.03952400	-6.36888300	-0.33845900
C	1.41453800	11.23070400	0.34929800
C	2.41986500	11.79375800	-0.44232300
C	0.53453000	12.07747500	1.02969400
C	2.54504000	13.17222800	-0.53632100
H	3.09980500	11.14821800	-0.98514800
C	0.65774900	13.45425100	0.91396800
H	-0.24376500	11.65045300	1.65103300
C	1.66518900	14.01249800	0.13594800
H	3.33115400	13.59073400	-1.15525400
H	-0.03407500	14.09477000	1.44998400
H	1.76331200	15.08874800	0.05409000
C	10.76120300	4.40047800	-0.78227300
C	10.89139700	5.55198500	-1.57620000
C	11.94325500	3.72989500	-0.42867900
C	12.13484300	6.01868600	-1.98171600
H	10.00130300	6.07665700	-1.90544800
C	13.19086900	4.20915000	-0.80061900
H	11.88200800	2.81425500	0.14957700
C	13.28896100	5.35557000	-1.58182000
H	12.20415000	6.90096400	-2.60883000
H	14.08772200	3.68000000	-0.49721300
H	14.26216100	5.72355500	-1.88860900
C	9.21298900	-7.31773400	-0.74983400
C	10.27831800	-6.87139100	-1.54921500
C	9.24210900	-8.65845300	-0.33135900
C	11.31328800	-7.71879800	-1.92043400
H	10.28461600	-5.84388400	-1.89727400
C	10.28527000	-9.50594600	-0.67859900
H	8.43558900	-9.03615100	0.28817900
C	11.32126700	-9.03762900	-1.47931500
H	12.11633400	-7.35189700	-2.55069800
H	10.28942600	-10.53313800	-0.33012000
H	12.13333900	-9.69990700	-1.76020800
C	-1.85044400	-10.74625200	2.47257800
C	-0.97212400	-11.83939800	2.59500200
C	-2.95419600	-10.73591500	3.34563900
C	-1.19054700	-12.86472800	3.50485600
H	-0.10006300	-11.88478200	1.95010700
C	-3.17435300	-11.74966100	4.26872800
H	-3.64432600	-9.89921800	3.31012600
C	-2.29559200	-12.82520000	4.34926100
H	-0.49580300	-13.69702400	3.55916400
H	-4.03197400	-11.69936700	4.93241700
H	-2.46692700	-13.62121400	5.06637200
C	-10.28515900	-4.17828400	-2.35089900
C	-11.52369600	-3.73854300	-1.87291500
C	-10.22964900	-5.14735300	-3.35659400
C	-12.69128800	-4.25020400	-2.41630400
H	-11.56313300	-3.01900000	-1.06393400
C	-11.40430800	-5.65125000	-3.89019100
H	-9.26692900	-5.48801200	-3.71715300
C	-12.63796300	-5.20486400	-3.42643600
H	-13.64829500	-3.91331100	-2.03518100
H	-11.35503700	-6.39495200	-4.67692900
H	-13.55366000	-5.60594200	-3.84459200
C	-8.86752800	6.58774600	1.12822300
C	-9.82409400	6.04319300	1.98880500
C	-8.95234100	7.93463900	0.77021600
C	-10.85878700	6.83447500	2.46497500
H	-9.74777400	5.00506000	2.28985300
C	-9.98372700	8.72012900	1.26324300
H	-8.21044800	8.35899900	0.10437100
C	-10.94432000	8.17533700	2.10742700
H	-11.59304900	6.40174000	3.13506000
H	-10.03992400	9.76409900	0.97646400
H	-11.75049700	8.79165700	2.48775100
N	-9.08461100	-3.68750400	-1.79353300
N	-7.79765400	5.79928000	0.64612700
N	1.27327400	9.83309800	0.45730000

Structure S42. The coordinates of both the optimized structures of B2N4L$_2$ at S$_0$ and S$_1$ in toluene.

S_0-optimized structure				S_1-optimized structure			
C	-9.32962500	-0.36631200	-0.02069700	C	-6.01076300	6.84493400	1.34368400
C	-9.63365700	0.34923100	1.13797900	C	-5.18801700	7.51460600	2.26046200
C	-8.52321400	0.22672200	-0.99149900	C	-5.58336600	6.73674200	0.01301500
C	-9.14104500	1.63297200	1.31654600	C	-3.96606900	8.01931400	1.86295000
H	-10.26365200	-0.10545200	1.89402800	H	-5.50572500	7.61494400	3.29110400
C	-8.04303800	1.51443500	-0.81224800	C	-4.37298300	7.26770000	-0.38015900
H	-8.27970300	-0.32885400	-1.88992300	H	-6.21078800	6.22543500	-0.70686400
C	-8.34386300	2.24043100	0.34267800	C	-3.52219500	7.90697100	0.53581700
H	-9.39222200	2.18101800	2.21834000	H	-3.34595700	8.52421100	2.59504600
H	-7.41604700	1.96231700	-1.57563600	H	-4.06173700	7.14988200	-1.41175000
C	-7.83888900	3.61985300	0.51912400	C	-2.20938100	8.39515500	0.12050600
C	-7.14121700	3.99240400	1.67081100	C	-1.10057700	8.32869800	0.98320300
C	-8.05002300	4.58459000	-0.47005300	C	-1.99748900	8.90644100	-1.17323700
C	-6.66092000	5.28454200	1.82673300	C	0.15871100	8.69448200	0.56465500
H	-6.95921700	3.25607200	2.44605400	H	-1.22525500	7.93889700	1.98662600
C	-7.58615800	5.88132300	-0.31250700	C	-0.75228800	9.31312700	-1.59378000
H	-8.59931300	4.31723800	-1.36621100	H	-2.83423000	8.99085800	-1.85664400
C	-6.88361900	6.23912100	0.83678900	C	0.35776300	9.19084600	-0.73687000
H	-6.11056000	5.56351700	2.71809600	H	1.00445300	8.60841200	1.23530200
H	-7.76506900	6.62617400	-1.07961700	H	-0.61453900	9.68848700	-2.60004500
C	-8.88200900	-2.72163300	-0.38314900	C	-7.54165700	4.97304100	1.26000400
C	-7.86086900	-2.89841900	0.54959700	C	-6.57129600	3.96920300	1.25920000
C	-8.94539100	-3.58832200	-1.47529600	C	-8.82220900	4.67643100	0.78959000
C	-6.94371100	-3.92516500	0.40362700	C	-6.87676700	2.70234900	0.79624400
H	-7.80341200	-2.23728900	1.40675300	H	-5.57439900	4.18833300	1.62320000
C	-8.01794000	-4.60691300	-1.61848900	C	-9.12060400	3.40165800	0.33592700
H	-9.72304000	-3.45238400	-2.21836100	H	-9.58425400	5.44718900	0.78883200
C	-6.99768200	-4.81013800	-0.67982200	C	-8.15760800	2.38580800	0.32952400
H	-6.19353900	-4.05858400	1.17413400	H	-6.10141800	1.94469700	0.78652400
H	-8.07616400	-5.23851400	-2.49748300	H	-10.12685000	3.18559800	-0.00576400
C	-6.02935700	-5.91671600	-0.81413800	C	-8.46553500	1.02407600	-0.14152500
C	-4.77866600	-5.87834200	-0.18835700	C	-7.91576900	-0.09332200	0.49522700
C	-6.32113400	-7.06621200	-1.55717300	C	-9.29729700	0.79142500	-1.24149500
C	-3.88258100	-6.92917300	-0.27145100	C	-8.17650200	-1.37841300	0.05764000
H	-4.48491500	-5.00441800	0.38114800	H	-7.29870800	0.04270300	1.37668700
C	-5.41974200	-8.10900100	-1.67952500	C	-9.54639000	-0.48997300	-1.70373300
H	-7.28310500	-7.16379500	-2.04696000	H	-9.72290500	1.63234400	-1.77872900
C	-4.18248500	-8.06930100	-1.02562100	C	-8.98622000	-1.60087000	-1.06209800
H	-2.93708500	-6.86008800	0.25102900	H	-7.75394100	-2.22161300	0.58970900
H	-5.68536500	-8.97342200	-2.27526200	H	-10.16643500	-0.63329900	-2.58032800
C	-1.90233400	-8.95689800	-0.90104700	C	-8.21370600	-3.88825600	-1.35444000
C	-1.19290400	-9.84318600	-0.08796000	C	-8.52504700	-5.13717700	-0.81529400
C	-1.21493500	-7.88728600	-1.48025100	C	-6.88792100	-3.61916400	-1.70026100
C	0.15470600	-9.64493800	0.16049000	C	-7.52873500	-6.07521100	-0.59909800
H	-1.70952500	-10.67737000	0.37164400	H	-9.54910700	-5.36008800	-0.53916400
C	0.12828600	-7.68593000	-1.20954400	C	-5.89744700	-4.55819500	-1.46978000
H	-1.74159300	-7.20806400	-2.14042000	H	-6.63701800	-2.66423500	-2.14728700
C	0.84324400	-8.55182700	-0.37470600	C	-6.18982100	-5.80300500	-0.90049200
H	0.66965200	-10.32502600	0.83017700	H	-7.79075800	-7.01969700	-0.13527800
H	0.63948100	-6.85166000	-1.67757200	H	-4.87822700	-4.32870400	-1.76034400
C	-5.01354100	7.78775200	0.92741600	C	2.71247800	8.66746000	-0.81060100
C	-4.19750500	6.96505300	0.14443100	C	2.55213100	7.27044900	-0.77677400
C	-4.40972900	8.83127700	1.64030400	C	3.97240200	9.20714300	-0.49686300
C	-2.83797300	7.20465200	0.05063200	C	3.60955000	6.45392400	-0.46224000
H	-4.63835500	6.15104900	-0.41782200	H	1.59076300	6.83563200	-1.02455400
C	-3.05115900	9.06551300	1.53156700	C	5.02670800	8.38086600	-0.18865800
H	-5.01372700	9.45898000	2.28431700	H	4.11093800	10.28222900	-0.50101000
C	-2.22981600	8.26807100	0.72511800	C	4.90195400	6.96541700	-0.17186200
H	-2.24655300	6.57604700	-0.60534300	H	3.43673900	5.38623300	-0.44301800
H	-2.61550500	9.87101000	2.11206900	H	5.98612800	8.83580900	0.02066300
C	2.25323200	-8.29996100	-0.02575700	C	-5.12355900	-6.77093000	-0.58539700
C	2.71808500	-6.99982600	0.19041400	C	-3.86212000	-6.33740100	-0.16660700
C	3.16723900	-9.34418700	0.15226600	C	-5.33525600	-8.15273400	-0.65037700
C	4.01646400	-6.75038500	0.60110400	C	-2.86985000	-7.23022800	0.19922300
H	2.03452400	-6.16409500	0.08709100	H	-3.66387700	-5.27513600	-0.07480800
C	4.47361100	-9.10640900	0.54263700	C	-4.34658000	-9.05578800	-0.30061700
H	2.85809300	-10.36507100	-0.04516200	H	-6.28501400	-8.53300500	-1.01072000
C	4.91612400	-7.80301100	0.79733900	C	-3.09929400	-8.60881200	0.15153400
H	4.32856600	-5.73208600	0.79744200	H	-1.91626300	-6.85643100	0.55143000
H	5.16040300	-9.93668000	0.65497900	H	-4.53799600	-10.11933300	-0.37816100
C	-0.79485300	8.55427100	0.55055400	C	6.02462000	6.09527600	0.08821000
C	0.12027800	7.53283500	0.26446800	C	5.97240900	4.70389900	-0.18555600
C	-0.30220500	9.86318700	0.63269000	C	7.25345700	6.57055200	0.61584200
C	1.45333300	7.81704200	0.02743100	C	7.05932400	3.88706700	-0.00264900
H	-0.21545500	6.50206700	0.24605700	H	5.06686000	4.26373300	-0.58635900
C	1.03180600	10.13999800	0.38781300	C	8.33766000	5.74453900	0.78751700
H	-0.98162500	10.67931500	0.85169300	H	7.34255400	7.60578700	0.92646200
C	1.95004700	9.13174800	0.04688200	C	8.32384200	4.36121700	0.44625400
H	2.13188000	6.99843400	-0.18648900	H	6.94986800	2.83365800	-0.23414000
H	1.37034800	11.16748600	0.45947500	H	9.23389900	6.16684900	1.22805800
B	3.43935200	9.42595400	-0.30423900	B	9.53529700	3.42387900	0.59688500
C	4.48005000	8.24124400	-0.29684000	C	9.29564900	1.85893800	0.49706900
C	5.34914600	7.99013700	0.77117800	C	9.44266500	0.98724800	1.58691700
C	4.56609600	7.38709300	-1.40371900	C	8.89509200	1.26949400	-0.71286400
C	6.26201700	6.94410300	0.73428900	C	9.20583900	-0.37680900	1.48117700
H	5.30829400	8.61408200	1.65903300	H	9.72192500	1.38816700	2.55675900
C	5.46490700	6.33173500	-1.43885700	C	8.63759800	-0.08836300	-0.82505300
H	3.92109900	7.55116400	-2.26191700	H	8.78487700	1.89373100	-1.59462900
C	6.33227500	6.09163900	-0.36999500	C	8.78689400	-0.94028900	0.27319200
H	6.90806000	6.76659300	1.58775500	H	9.30607700	-1.00898300	2.35798600
H	5.51460000	5.69929900	-2.31897900	H	8.34058400	-0.50213500	-1.78366400
C	7.28417000	4.96342600	-0.41514900	C	8.48831000	-2.38066300	0.15671000
C	6.88653800	3.71982000	-0.91598500	C	7.34979900	-2.81621800	-0.53041700
C	8.60242200	5.10441700	0.03060300	C	9.32326700	-3.34881600	0.72568700
C	7.77220900	2.65564500	-0.95479000	C	7.05071000	-4.16511700	-0.62573900

H	5.86267700	3.58136800	-1.24641700
C	9.49591900	4.04740500	-0.05379800
H	8.93616100	6.06377500	0.41178000
C	9.10744700	2.78985900	-0.54225000
H	7.42488500	1.69642300	-1.32407500
H	10.51939900	4.19716500	0.27440400
C	6.83008600	-6.30839900	0.96827000
C	7.27616100	-5.49543800	2.01063200
C	6.97239900	-5.86335000	-0.34632700
C	7.83379100	-4.25666300	1.74121500
H	7.17753300	-5.83821600	3.03406800
C	7.53325700	-4.62334100	-0.60843800
H	6.62791800	-6.49121500	-1.16005700
C	7.96598400	-3.79232300	0.42927600
H	8.17852900	-3.63922800	2.56344100
H	7.61656500	-4.28374000	-1.63506800
C	8.52138800	-2.45181000	0.15572800
C	9.43725700	-2.23887200	-0.87916200
C	8.12814200	-1.35374900	0.92733900
C	9.95644000	-0.97484900	-1.11582300
H	9.76697800	-3.07929900	-1.48094000
C	8.61316900	-0.08575400	0.65233800
H	7.40434000	-1.49336900	1.72325200
C	9.55040300	0.14092500	-0.36755000
H	10.68545900	-0.84609700	-1.90933100
H	8.26127100	0.75184900	1.24553600
B	10.09393900	1.58142200	-0.64590700
C	-7.32510300	8.61992600	1.04338800
C	-7.06889400	9.84663000	0.42204000
C	-8.54740900	8.43866800	1.69810900
C	-8.00725000	10.86702800	0.47484600
H	-6.13567000	9.99714700	-0.10678900
C	-9.48465900	9.46073500	1.73067400
H	-8.75781900	7.49261900	2.18249900
C	-9.22154800	10.68394600	1.12593100
H	-7.78908500	11.81018200	-0.01436000
H	-10.42590500	9.29870900	2.24456800
H	-9.95346000	11.48262400	1.15895400
C	3.93523700	10.84722400	-0.73392000
C	3.11666300	11.74035300	-1.44484300
C	5.25134100	11.25797800	-0.46710700
C	3.58127500	12.98336500	-1.85379600
H	2.10588600	11.44583700	-1.70483500
C	5.71240200	12.51134300	-0.84272500
H	5.92485300	10.57996400	0.04625000
C	4.87696700	13.37696300	-1.54066400
H	2.93246200	13.64642300	-2.41582500
H	6.72844400	12.80895900	-0.60714000
H	5.23948300	14.35137100	-1.85021300
C	11.59251000	1.80260200	-1.02982400
C	11.99350500	2.88780800	-1.82656000
C	12.59475700	0.92101900	-0.59209300
C	13.32276000	3.07838100	-2.17812500
H	11.24475600	3.58482600	-2.18769200
C	13.92932700	1.11872500	-0.91844400
H	12.32045400	0.07351800	0.02719600
C	14.29440300	2.19623000	-1.71812000
H	13.60365200	3.91646700	-2.80677500
H	14.68530700	0.43123000	-0.55463500
H	15.33535800	2.34814900	-1.98307400
C	6.87434500	-8.43884200	2.13333200
C	8.25213100	-8.64338700	2.01994100
C	6.17257200	-9.09758900	3.14659800
C	8.91037600	-9.48641800	2.90318300
H	8.80159100	-8.13768900	1.23454500
C	6.83745400	-9.95224700	4.01441600
H	5.10669900	-8.93555100	3.25320100
C	8.20852600	-10.15108700	3.90211000
H	9.97987200	-9.63311700	2.79931700
H	6.27714800	-10.45461600	4.79541400
H	8.72423000	-10.81510400	4.58618900
C	-3.76242500	-10.46290600	-1.30036500
C	-4.81933800	-10.95420700	-0.53177200
C	-3.15742000	-11.29662200	-2.24211400
C	-5.26897700	-12.25429300	-0.71580000
H	-5.28490200	-10.31308900	0.20762200
C	-3.60161200	-12.60073800	-2.40787700
H	-2.33822800	-10.91455900	-2.84017800
C	-4.66196700	-13.08519100	-1.65058700
H	-6.09108500	-12.62308900	-0.11236600
H	-3.12239200	-13.23718100	-3.14354400
H	-5.01189000	-14.10204800	-1.78683300
C	-11.19863600	-1.93581300	-0.20596300
C	-11.69177000	-3.20085000	0.13742100
C	-12.11384700	-0.92766500	-0.53431900
C	-13.05709100	-3.44469800	0.14061700
H	-11.00189500	-3.99088500	0.40736100
C	-13.47706600	-1.18206600	-0.51284100
H	-11.75272800	0.05537400	-0.81072000
C	-13.96317100	-2.44085200	-0.18040900
H	-13.41361600	-4.43279400	0.41136300
H	-14.16488300	-0.38424800	-0.77186900
H	-15.02917400	-2.63602000	-0.17114800
N	-3.28414400	-9.14192200	-1.12247800
N	-9.82241600	-1.67934200	-0.21817200
N	-6.39473300	7.56411700	0.99812500
N	6.22527800	-7.55886000	1.24130300
H	6.67704400	-2.08183900	-0.96035000
C	9.04609000	-4.69940800	0.58041800
H	10.21724400	-3.03429700	1.25386000
C	7.89786200	-5.14949800	-0.09143000
H	6.14300500	-4.46787700	-1.13776500
H	9.73101400	-5.42531700	1.00698600
C	-0.74106600	-9.14422700	0.41322500
C	0.07815900	-9.05020700	1.53795200
C	-0.20688900	-8.85689000	-0.84249200
C	1.40233100	-8.66450200	1.40688800
H	-0.33059800	-9.27942800	2.51536100
C	1.12077900	-8.47743400	-0.96718200
H	-0.84176300	-8.92693600	-1.71838600
C	1.94798800	-8.36833900	0.15433500
H	2.02915700	-8.60170200	2.28963700
H	1.51880700	-8.24357900	-1.94857900
C	3.35682100	-7.94136100	0.02938200
C	4.19500800	-8.48346600	-0.94956000
C	3.88501700	-6.97916400	0.89512000
C	5.52509400	-8.09916300	-1.03319400
H	3.80690600	-9.23916600	-1.62465200
C	5.19999600	-6.56030500	0.76895200
H	3.24543300	-6.53190900	1.64876900
C	6.06404800	-7.12016400	-0.18456800
H	6.16040200	-8.56272500	-1.78107300
H	5.57214800	-5.78637600	1.43220600
B	7.56014000	-6.66331200	-0.26531800
C	1.85153400	10.56134500	-2.07128400
C	2.78015900	10.43791200	-3.11160300
C	1.14931500	11.76124900	-1.90314100
C	2.99099700	11.50152900	-3.97286800
H	3.31297600	9.50491500	-3.24587200
C	1.36823700	12.81461400	-2.77446500
H	0.45535000	11.86445200	-1.07772400
C	2.28841200	12.69182400	-3.81147100
H	3.70363100	11.39578200	-4.78249500
H	0.82894700	13.74397400	-2.63263200
H	2.46015900	13.52081800	-4.48780700
C	11.00277600	3.94963000	0.79865300
C	11.43434400	5.17620100	0.25511900
C	11.98931300	3.18977800	1.45693700
C	12.74004000	5.62794700	0.38842100
H	10.73309200	5.77675700	-0.31361800
C	13.29232000	3.64010400	1.61046800
H	11.72801100	2.21615600	1.85637200
C	13.67703500	4.86755000	1.07994000
H	13.03056200	6.57495000	-0.05561500
H	14.01605200	3.02658800	2.13780800
H	14.69738500	5.21905400	1.19069600
C	8.69107500	-7.71301000	-0.51693600
C	9.89566500	-7.35439900	-1.14436900
C	8.54357600	-9.05451500	-0.12720800
C	10.89689200	-8.28640700	-1.38082900
H	10.04081000	-6.32785400	-1.46363700
C	9.54988500	-9.98713200	-0.33784400
H	7.62684400	-9.36548600	0.36264500
C	10.72728600	-9.60444400	-0.97123700
H	11.81178500	-7.98581300	-1.87995300
H	9.41642300	-11.01349900	-0.01321700
H	11.51202400	-10.33291000	-1.14572400
C	-2.41387700	-10.70034400	1.24181200
C	-1.63458100	-11.84369600	1.04239800
C	-3.47497300	-10.75561300	2.15047800
C	-1.91189800	-13.01148400	1.73758200
H	-0.81121000	-11.81035600	0.33866600
C	-3.75422400	-11.93390600	2.82786300
H	-4.07694800	-9.87246400	2.32714700
C	-2.97585700	-13.06840100	2.63018500
H	-1.29552000	-13.88762900	1.56844900
H	-4.58108800	-11.95702100	3.52940900
H	-3.19449700	-13.98451400	3.16657700
C	-10.45214200	-3.27220800	-2.09586700
C	-11.65516500	-2.80274900	-1.56199800
C	-10.48738300	-4.13091400	-3.19752200
C	-12.86431800	-3.17631600	-2.13081300
H	-11.63836100	-2.14761100	-0.69908800
C	-11.70217200	-4.51302100	-3.74769700
H	-9.55786300	-4.49543900	-3.61869100
C	-12.89760100	-4.03537500	-3.22313300
H	-13.78868400	-2.80320200	-1.70364300
H	-11.71081700	-5.18022900	-4.60258200
H	-13.84472100	-4.33046300	-3.65977300
C	-8.05663000	6.89824200	2.67824600
C	-8.72221500	6.15198300	3.65444400
C	-8.23692000	8.28400600	2.63636500
C	-9.55345100	6.78493300	4.56668900
H	-8.58160500	5.07875400	3.69766400
C	-9.05767100	8.90764000	3.56388200
H	-7.73819800	8.86560700	1.87004500
C	-9.72351400	8.16415100	4.53185000
H	-10.06162800	6.19296300	5.31967200
H	-9.18916600	9.98293900	3.51760200
H	-10.37007200	8.65438400	5.25026200
N	-9.21393100	-2.90439300	-1.52823900
N	-7.22005700	6.26411400	1.73811100
N	1.63807900	9.49290000	-1.18287100
N	-2.10323900	-9.51495700	0.54529500

Structure S43. The coordinates of both the optimized structures of B1N5L$_2$ at S$_0$ and S$_1$ in toluene.

S_0-optimized structure				S_1-optimized structure			
C	-9.30006400	-1.37746500	0.57290400	C	8.99731000	-1.13812400	-1.65373900
C	-9.58610400	-0.67565000	1.74522600	C	8.96660400	-0.17309500	-2.66843100
C	-8.66090600	-0.71329200	-0.47379600	C	8.81075900	-0.72707200	-0.32836500
C	-9.24247800	0.66263300	1.86122000	C	8.72163600	1.15235400	-2.36434100
H	-10.08416900	-1.18494000	2.56237300	H	9.11619400	-0.47460300	-3.69819800
C	-8.32799700	0.62628400	-0.35460400	C	8.59354300	0.60318000	-0.03115400
H	-8.42850100	-1.25562700	-1.38312500	H	8.83244800	-1.46521200	0.46442400
C	-8.61363000	1.33908500	0.81240800	C	8.52680600	1.57430200	-1.04103700
H	-9.47918200	1.19701200	2.77509400	H	8.69339100	1.87932000	-3.16837900
H	-7.82759200	1.12725500	-1.17622600	H	8.43456700	0.89208200	1.00158700
C	-8.25940200	2.77086100	0.92072500	C	8.22651900	2.96950900	-0.71566200
C	-7.53470300	3.25801400	2.01152900	C	7.40254500	3.74768400	-1.54639300
C	-8.63293400	3.67051000	-0.08186500	C	8.71871500	3.56371400	0.45956700
C	-7.17779700	4.59646800	2.09022700	C	7.04198600	5.03157400	-1.19988400
H	-7.22849300	2.57502800	2.79648200	H	7.00528000	3.31790500	-2.45856800
C	-8.29046100	5.01100600	-0.00203400	C	8.40146000	4.86038200	0.79815700
H	-9.20833000	3.31458900	-0.92951300	H	9.37061600	2.99442800	1.11208400
C	-7.55174100	5.48195400	1.08188500	C	7.53663000	5.60907500	-0.01844800
H	-6.60049800	4.96395600	2.93103600	H	6.38227200	5.60728500	-1.83730300
H	-8.58883800	5.70163700	-0.78258000	H	8.78334600	5.29439800	1.71395600
C	-8.59285300	-3.64822800	0.11482500	C	8.30298800	-3.43316100	-1.34170500
C	-7.48001600	-3.75609400	0.94677900	C	6.93097100	-3.17812800	-1.30559800
C	-8.64775900	-4.43703600	-1.03418200	C	8.78545900	-4.61655600	-0.77921500
C	-6.45766900	-4.63907400	0.64379000	C	6.06571000	-4.08726400	-0.72424600
H	-7.43129300	-3.15558600	1.84805800	H	6.54628600	-2.26142800	-1.73682800
C	-7.61403700	-5.30812100	-1.33745400	C	7.90960900	-5.52476100	-0.20578800
H	-9.50253300	-4.35482100	-1.69608200	H	9.84879900	-4.82527800	-0.80216100
C	-6.49427300	-5.43883600	-0.50522500	C	6.53152800	-5.28369100	-0.16816300
H	-5.63196900	-4.72807600	1.33954100	H	5.00593400	-3.86068200	-0.69196600
H	-7.67266400	-5.87690800	-2.25797000	H	8.30163800	-6.44806500	0.20657900
C	-5.40308600	-6.38561600	-0.81343100	C	5.58665200	-6.24885900	0.42135800
C	-4.12946700	-6.24508200	-0.25174700	C	4.34256100	-6.48056000	-0.17361600
C	-5.58940700	-7.47875700	-1.66735600	C	5.88039600	-6.95609400	1.59129200
C	-3.11255500	-7.15061600	-0.49576300	C	3.43390500	-7.37052600	0.36826300
H	-3.91282200	-5.40444000	0.39669600	H	4.09353300	-5.98047800	-1.10334200
C	-4.57075600	-8.37115800	-1.95112100	C	4.96786200	-7.83038200	2.15795600
H	-6.55829600	-7.65419600	-2.12005800	H	6.82522000	-6.78629700	2.09680800
C	-3.30972900	-8.23610500	-1.35718200	C	3.72390300	-8.05291800	1.55533200
H	-2.15357200	-7.00888200	-0.01449600	H	2.49067000	-7.54492000	-0.13413400
H	-4.76087300	-9.19431900	-2.62845500	H	5.21283300	-8.33514700	3.08460100
C	-0.93380300	-8.83698600	-1.39432700	C	1.40386800	-8.68297200	1.92218900
C	-0.10613500	-9.71418500	-0.69114500	C	0.56001000	-9.69748900	1.46890200
C	-0.39062800	-7.63840400	-1.86328100	C	0.86584200	-7.41451200	2.14814300
C	1.21449600	-9.38470500	-0.43832900	C	-0.77631100	-9.43580200	1.21392900
H	-0.51060400	-10.64784800	-0.31862900	H	0.96313100	-10.68694300	1.28692200
C	0.92676100	-7.30903100	-1.58930500	C	-0.46808400	-7.15946200	1.88029200
H	-1.00984400	-6.96025800	-2.43893900	H	1.50405700	-6.62531900	2.52845200
C	1.75800900	-8.16739100	-0.86091900	C	-1.31848500	-8.15813500	1.39127800
H	1.82118000	-10.06552700	0.14859200	H	-1.39758600	-10.22982500	0.81470600
H	1.32531900	-6.37510400	-1.97064500	H	-0.86273400	-6.16851000	2.07554600
C	-5.81721500	7.17583100	0.95419800	C	5.77814300	7.24040600	0.22463500
C	-4.98032700	6.33660100	0.21138800	C	4.76834300	6.29940600	0.50361300
C	-5.26513000	8.34112500	1.50196200	C	5.39921100	8.54011300	-0.16206900
C	-3.65531900	6.67231300	-0.00298200	C	3.44530700	6.65312500	0.42673500
H	-5.37906400	5.42957300	-0.22553100	H	5.04260900	5.30385100	0.83222300
C	-3.94258200	8.67296400	1.26774000	C	4.07149100	8.88485400	-0.23533400
H	-5.88089300	8.98863200	2.11447500	H	6.16261300	9.26403800	-0.42294100
C	-3.10489000	7.85421000	0.50115900	C	3.03240200	7.96549600	0.07421300
H	-3.04993300	6.02116400	-0.62353200	H	2.70379900	5.91832200	0.71105200
H	-3.54527400	9.57666400	1.71710200	H	3.81949700	9.88403900	-0.56702300
C	3.13670600	-7.79149600	-0.49865200	C	-2.71577300	-7.86495900	1.02353200
C	3.45901300	-6.47558300	-0.15670500	C	-3.07024400	-6.62637700	0.48131600
C	4.16286900	-8.74013700	-0.42543600	C	-3.73201400	-8.81864100	1.15334900
C	4.72543600	-6.12621900	0.28150600	C	-4.35951500	-6.36000200	0.05334600
H	2.68772300	-5.71335400	-0.17968900	H	-2.31005200	-5.86576400	0.34089200
C	5.43707800	-8.40051100	-0.00692400	C	-5.02741200	-8.56031100	0.74134900
H	3.96684200	-9.76394000	-0.72570900	H	-3.51244900	-9.77681200	1.61193100
C	5.73471800	-7.08906900	0.38373600	C	-5.36008100	-7.33132300	0.15787500
H	4.92536000	-5.10363200	0.57677300	H	-4.58724200	-5.40017800	-0.39366400
H	6.21141200	-9.15746500	0.02481700	H	-5.79276700	-9.31611600	0.87058700
C	-1.71513700	8.23062100	0.18616100	C	1.63924000	8.34103300	0.08149600
C	-0.72318500	7.25790100	0.01429800	C	0.60328500	7.37096600	0.08988300
C	-1.35397400	9.57077000	-0.00272500	C	1.22200400	9.69567000	0.14106700
C	0.56022600	7.61005900	-0.36798000	C	-0.71780800	7.72587300	0.20231200
H	-0.96016300	6.21466000	0.19213400	H	0.84941900	6.31966600	-0.00843700
C	-0.07299900	9.91360800	-0.40174400	C	-0.10186100	10.03759100	0.27646400
H	-2.10156900	10.34749500	0.11606300	H	1.96153700	10.48866400	0.12793000
C	0.92202300	8.94478200	-0.61586300	C	-1.14684800	9.07416700	0.34623800
H	1.29949300	6.82760600	-0.50164300	H	-1.46393300	6.93859600	0.21881400
H	0.16112300	10.96035400	-0.56442700	H	-0.35834800	11.09005400	0.33408400
B	2.33194300	9.29210000	-1.18635800	B	-2.61754000	9.42876900	0.63279600
C	3.54517100	8.34417000	-0.90010800	C	-3.75389400	8.37773300	0.34759000
C	3.78772100	7.79019400	0.36591600	C	-3.82700100	7.63125600	-0.84203700
C	4.42087300	7.97785000	-1.93327400	C	-4.75375200	8.11701800	1.30159400
C	4.85187100	6.93003100	0.59237600	C	-4.82050900	6.68950300	-1.06402200
H	3.12845100	8.04043700	1.19108100	H	-3.08240800	7.79840900	-1.61450000
C	5.45074400	7.07335500	-1.72759800	C	-5.72218800	7.14521500	1.10932000
H	4.27947000	8.39816200	-2.92368300	H	-4.75620700	8.68262400	2.22814400
C	5.68746400	6.53852800	-0.45809200	C	-5.77653300	6.41216500	-0.08122700
H	5.01335200	6.51858800	1.58331100	H	-4.83694000	6.13127600	-1.99511200
H	6.10058600	6.79683800	-2.55126800	H	-6.46608400	6.96474200	1.87913600
C	6.78016100	5.56743900	-0.24583300	C	-6.80101000	5.36868400	-0.27827400
C	6.95125800	4.49426400	-1.12446600	C	-7.07558700	4.44290500	0.73384300
C	7.66588500	5.68550100	0.82910400	C	-7.52488200	5.26818700	-1.47154000
C	7.96700700	3.57154900	-0.93810200	C	-8.03434300	3.45737400	0.56424500

H	6.26768600	4.37339900	-1.95766600
C	8.68925700	4.76946400	1.01812700
H	7.55890200	6.51464900	1.52021100
C	8.84927800	3.70055600	0.13469500
H	8.08271100	2.74041100	-1.62420100
H	9.37279800	4.87824800	1.85237500
C	7.45454400	-5.40860100	0.76774800
C	7.69248300	-4.66183400	1.92093100
C	7.65033100	-4.81519600	-0.47857600
C	8.10681400	-3.34350400	1.82612400
H	7.54930200	-5.12205300	2.89186100
C	8.06873900	-3.49581600	-0.56649500
H	7.46382500	-5.39292800	-1.37675700
C	8.29835000	-2.73393800	0.58276000
H	8.29470200	-2.77759700	2.73203800
H	8.20146600	-3.04250700	-1.54292200
C	8.71487600	-1.31877400	0.49954000
C	9.74303100	-0.90602400	-0.35281300
C	8.08484800	-0.34930700	1.28433000
C	10.13354500	0.42270800	-0.41524500
H	10.25403100	-1.63946800	-0.96737400
C	8.46767400	0.98018200	1.22615300
H	7.27345400	-0.64065800	1.94244200
C	9.50006300	1.38176800	0.37766200
H	10.93711000	0.72438000	-1.07725800
H	7.96564400	1.71952100	1.83941900
C	-8.18240200	7.82481600	1.16316700
C	-8.08148900	9.00266100	0.41617000
C	-9.33816900	7.60203700	1.91793100
C	-9.10642300	9.93753800	0.44311200
H	-7.20181000	9.18195300	-0.18980500
C	-10.36375700	8.53574500	1.92416900
H	-9.42683800	6.69206300	2.49945200
C	-10.25464000	9.71281500	1.19330300
H	-9.00924800	10.84438800	-0.14388800
H	-11.25154200	8.34263700	2.51642000
H	-11.05473200	10.44385700	1.20575100
C	2.54367800	10.54080200	-2.10639400
C	1.53763200	11.01741800	-2.96266900
C	3.77041900	11.22492200	-2.11342100
C	1.74488900	12.11626000	-3.78569400
H	0.58231700	10.50454600	-2.99624600
C	3.97577200	12.34162000	-2.91198600
H	4.57285800	10.87778400	-1.47084000
C	2.96288200	12.78624700	-3.75461900
H	0.95611300	12.45347400	-4.44956000
H	4.92749700	12.86126400	-2.88510100
H	3.12396400	13.65131200	-4.38907800
C	11.21152000	3.13738000	0.46154600
C	11.67091600	4.32147800	-0.12723500
C	12.11503500	2.35776800	1.19344100
C	12.99330400	4.71211800	0.02184600
H	10.98876000	4.93236000	-0.70587200
C	13.43878200	2.75180300	1.32062900
H	11.77505700	1.44444200	1.66623300
C	13.89043000	3.93170500	0.74143300
H	13.32581200	5.63347700	-0.44411000
H	14.11990300	2.13132600	1.89307300
H	14.92435600	4.23814000	0.84933400
C	7.75862500	-7.64224100	1.66154400
C	9.15278600	-7.64453800	1.56244000
C	7.14379700	-8.50714900	2.57139800
C	9.91112800	-8.49237600	2.35621000
H	9.63643400	-6.97738800	0.85855100
C	7.91062800	-9.36349000	3.34877000
H	6.06497300	-8.50433100	2.66984100
C	9.29705900	-9.36174600	3.25016200
H	10.99164600	-8.47951300	2.26428600
H	7.41612100	-10.02712000	4.04977200
H	9.89175200	-10.02835300	3.86398600
C	-2.60486700	-10.50364000	-1.94746700
C	-3.54852700	-11.22483300	-1.21375100
C	-1.94360800	-11.12477400	-3.00785500
C	-3.83276400	-12.54145500	-1.54855600
H	-4.05634400	-10.74928000	-0.38281700
C	-2.22089600	-12.44680400	-3.32539400
H	-1.21170400	-10.56397000	-3.57761500
C	-3.16962600	-13.16038200	-2.60205400
H	-4.56832400	-13.09029900	-0.97084300
H	-1.69952100	-12.91704600	-4.15179200
H	-3.38966900	-14.19089800	-2.85630700
C	-10.94714000	-3.19465200	0.62119000
C	-11.20424000	-4.52631400	0.97136500
C	-12.03314400	-2.32547900	0.45556900
C	-12.50759300	-4.96842500	1.14256000
H	-10.37845700	-5.21246000	1.11365300
C	-13.33028000	-2.77833300	0.64464400
H	-11.85787900	-1.29478300	0.17281900
C	-13.58268900	-4.10177500	0.98579500
H	-12.67941700	-6.00445100	1.41443200
H	-14.15359400	-2.08498100	0.50966300
H	-14.59877900	-4.45149000	1.12642100
N	-2.29048600	-9.16247500	-1.61628400
N	-9.63487700	-2.74536400	0.43811900
N	-7.16595200	6.84893100	1.14856600
N	7.00828000	-6.75171100	0.86454700
N	9.87366300	2.74325200	0.32103400

H	-6.51600400	4.48949400	1.66160100
C	-8.49050300	4.28842900	-1.64524200
H	-7.33898400	5.98104500	-2.26758100
C	-8.75423700	3.37256900	-0.62649400
H	-8.23199600	2.74316900	1.35567800
H	-9.05274500	4.23101000	-2.57060000
C	-7.11783900	-5.73108700	-0.35349100
C	-7.33893200	-5.10771100	-1.58049900
C	-7.34121200	-5.01851400	0.82299800
C	-7.76749200	-3.79076400	-1.62646100
H	-7.17257900	-5.66272000	-2.49679800
C	-7.77832200	-3.70296200	0.77061000
H	-7.16517300	-5.50204900	1.77723000
C	-7.99431500	-3.06424500	-0.45368700
H	-7.94244700	-3.31883000	-2.58716200
H	-7.93777500	-3.15565500	1.69332900
C	-8.44382600	-1.65757200	-0.51951500
C	-9.52872700	-1.20330500	0.23566700
C	-7.79616200	-0.74255700	-1.35340900
C	-9.96094800	0.11099500	0.15171000
H	-10.05186600	-1.89453600	0.88788200
C	-8.21844700	0.57388200	-1.43820600
H	-6.94107900	-1.06553400	-1.93744700
C	-9.31272100	1.01533600	-0.69281000
H	-10.81007100	0.44374000	0.73754800
H	-7.70198100	1.27175900	-2.08702700
C	8.03413200	7.77203100	0.96394800
C	7.62018200	8.60797900	2.00858700
C	9.35667900	7.83216700	0.50605900
C	8.52345500	9.48552200	2.58406400
H	6.60194900	8.54753500	2.37186300
C	10.24893300	8.71160300	1.09458000
H	9.66616600	7.20526000	-0.32133700
C	9.83884100	9.54254700	2.13340000
H	8.19950100	10.12200800	3.39917400
H	11.26801200	8.76007600	0.72897200
H	10.54013700	10.23277800	2.58713900
C	-3.03017500	10.79764200	1.28629900
C	-2.21952100	11.47541700	2.21654400
C	-4.26863100	11.39777100	0.98863600
C	-2.60924400	12.67336400	2.79984300
H	-1.26875900	11.03792800	2.50325600
C	-4.66136600	12.60207900	1.55591700
H	-4.93306500	10.90558000	0.28543000
C	-3.83174900	13.24821000	2.46703900
H	-1.96021400	13.15928800	3.52169100
H	-5.61893600	13.03858500	1.28984300
H	-4.13751100	14.18661800	2.91766400
C	-11.07157100	2.69377700	-1.02922600
C	-11.57817500	3.93569400	-0.62624900
C	-11.92979600	1.80306800	-1.68699800
C	-12.89993000	4.27084700	-0.87967300
H	-10.93238400	4.63622300	-0.11126600
C	-13.25312300	2.14575700	-1.92012700
H	-11.55424500	0.84364400	-2.02105200
C	-13.75163400	3.38112100	-1.52335900
H	-13.26755500	5.23908300	-0.55707300
H	-13.89687800	1.43863600	-2.43239600
H	-14.78512500	3.64611300	-1.71377900
C	-7.43874700	-8.06665600	-0.93131700
C	-8.83143900	-8.02487700	-0.81364900
C	-6.85689900	-9.08214300	-1.69661900
C	-9.61835300	-8.97662700	-1.44503300
H	-9.29222400	-7.24156400	-0.22351000
C	-7.65283400	-10.03868500	-2.31042400
H	-5.78059500	-9.11752500	-1.81336700
C	-9.03697000	-9.99400200	-2.19252800
H	-10.69662200	-8.92589300	-1.34022900
H	-7.18207100	-10.81771300	-2.90030300
H	-9.65426700	-10.74047400	-2.67880100
C	3.18324200	-10.09216300	2.80668800
C	4.24589600	-10.87052600	2.34026000
C	2.50099800	-10.49443600	3.95775800
C	4.62328800	-12.01788300	3.02322000
H	4.77184100	-10.57435100	1.44041300
C	2.87447300	-11.65284400	4.62329000
H	1.67812200	-9.89311700	4.32605000
C	3.94009100	-12.41911800	4.16537600
H	5.45005400	-12.61067000	2.64741000
H	2.33389500	-11.94986700	5.51526000
H	4.23380300	-13.31984300	4.69191200
C	10.11874500	-2.91548800	-2.90190800
C	9.84804700	-3.99565800	-3.74716600
C	11.34658900	-2.25506600	-3.01447400
C	10.79077200	-4.40472200	-4.67873300
H	8.89702200	-4.50893800	-3.67414100
C	12.27404900	-2.66268000	-3.96116200
H	11.57226900	-1.42776200	-2.35213400
C	12.00637400	-3.74104800	-4.79690200
H	10.56361100	-5.24381800	-5.32695100
H	13.22145400	-2.14008000	-4.03353500
H	12.73724100	-4.06162200	-5.53003600
N	2.78261700	-8.92400000	2.12430500
N	9.17645200	-2.49845100	-1.94374400
N	7.12533100	6.88516000	0.36383500
N	-6.65906200	-7.07502400	-0.30339900
N	-9.73380100	2.35920000	-0.78954300

Structure S44. The coordinates of both the optimized structures of N6L$_2$ at S$_0$ and S$_1$ in toluene.

S_0-optimized structure				S_1-optimized structure			
C	-9.36619300	-0.31747100	0.48408600	C	-9.09664600	-1.14169800	-1.04672400
C	-9.53217000	0.39274100	1.67376800	C	-8.92092400	-1.87407700	-2.22206600
C	-8.67591500	0.27893300	-0.57027700	C	-8.57962600	-1.64382000	0.14809600
C	-9.01839100	1.67451900	1.80059900	C	-8.23548300	-3.07839900	-2.19848600
H	-10.07095300	-0.06526100	2.49551500	H	-9.32315700	-1.49223700	-3.15336700
C	-8.17340000	1.56384300	-0.44096900	C	-7.90144300	-2.85121700	0.16446800
H	-8.53788200	-0.27269600	-1.49318000	H	-8.70990600	-1.07671600	1.06261200
C	-8.33620300	2.28484400	0.74446200	C	-7.71205200	-3.59027800	-1.00728500
H	-9.16142500	2.21825600	2.72827300	H	-8.11194100	-3.63804500	-3.11942000
H	-7.63515200	2.01315700	-1.26850300	H	-7.49514700	-3.22070700	1.09970800
C	-7.80208400	3.65886400	0.86532000	C	-6.97093100	-4.86785700	-0.98152400
C	-6.98597000	4.02731800	1.93779700	C	-5.97376600	-5.14189700	-1.92231600
C	-8.09535400	4.62130700	-0.10501500	C	-7.23590800	-5.83221100	-0.00447000
C	-6.46622900	5.31016500	2.03085900	C	-5.25924200	-6.32892900	-1.88310500
H	-6.73971800	3.29349700	2.69754200	H	-5.74187600	-4.40556100	-2.68415800
C	-7.58950300	5.90811900	-0.01037900	C	-6.52882400	-7.02312000	0.03753900
H	-8.73738900	4.35856300	-0.93876800	H	-8.01450800	-5.64957100	0.72823400
C	-6.76351800	6.26051100	1.05611800	C	-5.52807300	-7.28069700	-0.89974400
H	-5.82161600	5.58397000	2.85832500	H	-4.48038500	-6.52402200	-2.61136700
H	-7.82799700	6.64854200	-0.76542700	H	-6.74694800	-7.76184300	0.80019600
C	-8.95252800	-2.66182400	0.03901700	C	-9.09286300	1.22756000	-0.54673400
C	-7.87947600	-2.90745500	0.89369900	C	-7.73586500	1.42052600	-0.82842900
C	-9.08390000	-3.44178900	-1.11001300	C	-9.74602900	2.16806200	0.25993100
C	-6.97138400	-3.91379900	0.61235900	C	-7.06375500	2.52076300	-0.33434000
H	-7.77274700	-2.31474400	1.79511800	H	-7.21056200	0.69644400	-1.44014300
C	-8.16356500	-4.43856900	-1.39105300	C	-9.06536100	3.26610500	0.75181300
H	-9.90726800	-3.25360300	-1.78983400	H	-10.79637400	2.03435900	0.49158500
C	-7.08600100	-4.70645700	-0.53636700	C	-7.70692100	3.48074400	0.46438000
H	-6.17743600	-4.10407200	1.32459500	H	-6.00829300	2.63031700	-0.55638200
H	-8.27493400	-4.99796600	-2.31251900	H	-9.60563000	3.98828300	1.35403300
C	-6.11540300	-5.78277000	-0.82136900	C	-6.98377000	4.65020100	0.95998500
C	-4.83790600	-5.78626200	-0.25104700	C	-5.93577100	5.22092200	0.21441200
C	-6.42612300	-6.85952300	-1.65974700	C	-7.29038500	5.25296500	2.19493600
C	-3.93528500	-6.81068400	-0.47286600	C	-5.22797600	6.30970700	0.66997500
H	-4.52647800	-4.96657300	0.38545700	H	-5.70345600	4.82511100	-0.76768000
C	-5.51971900	-7.87208000	-1.92115300	C	-6.57868200	6.33247200	2.67318900
H	-7.40676100	-6.92562700	-2.11652500	H	-8.07059500	4.82798900	2.81655700
C	-4.25531200	-7.87846500	-1.31929700	C	-5.52682600	6.88578500	1.91913300
H	-2.96902300	-6.77660200	0.01344400	H	-4.44943000	6.74570200	0.05665100
H	-5.80251400	-8.67816700	-2.58661400	H	-6.80980000	6.73962500	3.65014900
C	-1.96672300	-8.75122100	-1.32909900	C	-3.40510200	8.02823000	2.07566400
C	-1.25046000	-9.69896400	-0.59593000	C	-2.80643100	9.25981600	1.71934200
C	-1.28699300	-7.63420800	-1.82060300	C	-2.60240400	6.86433500	2.02712100
C	0.09650600	-9.51394200	-0.33403700	C	-1.50849000	9.30986000	1.28450800
H	-1.76167700	-10.57154300	-0.20677300	H	-3.39854200	10.16731100	1.75010000
C	0.05599500	-7.44808600	-1.53717700	C	-1.30798300	6.92066500	1.58742700
H	-1.82029900	-6.90537100	-2.41987600	H	-3.01939400	5.92078600	2.36025700
C	0.77717100	-8.37532200	-0.77665500	C	-0.70083900	8.13838400	1.15178200
H	0.61673800	-10.24385600	0.27648900	H	-1.11617300	10.26970700	0.97242800
H	0.56126300	-6.57469400	-1.93506500	H	-0.72518300	6.00836500	1.60852700
C	-4.83187600	7.72785300	0.94749200	C	-3.38845600	-8.40502700	-0.65740000
C	-4.12360100	6.85642200	0.11598400	C	-2.83282700	-7.38319600	0.11670500
C	-4.12523200	8.75522300	1.58269500	C	-2.52538500	-9.34175600	-1.23542100
C	-2.76713500	7.03203200	-0.09792500	C	-1.46570700	-7.31993600	0.32393400
H	-4.64598200	6.05301500	-0.38898800	H	-3.48104400	-6.64938400	0.58043500
C	-2.77083000	8.92644300	1.35333100	C	-1.16053100	-9.27412700	-1.01274100
H	-4.64518800	9.42233300	2.26009100	H	-2.93100200	-10.12598600	-1.86372900
C	-2.05713900	8.07843100	0.49813100	C	-0.59409900	-8.26799400	-0.22133700
H	-2.26058700	6.36539900	-0.78704400	H	-1.07423900	-6.53990900	0.96731700
H	-2.25160800	9.72460000	1.87285600	H	-0.51823500	-10.00694700	-1.48896200
C	2.18402700	-8.14321200	-0.40224900	C	0.60716600	8.16993600	0.57463200
C	2.64060700	-6.86210800	-0.08103400	C	1.26977600	6.98084500	0.14835100
C	3.10207200	-9.19391300	-0.29725800	C	1.31780200	9.38451000	0.33795600
C	3.93249300	-6.64025700	0.36517200	C	2.47967000	7.00444800	-0.49455400
H	1.95461800	-6.02321800	-0.12748700	H	0.78221200	6.02187700	0.26924300
C	4.40155900	-8.98259300	0.12869800	C	2.53519900	9.41205200	-0.29329300
H	2.80118800	-10.19738500	-0.57887400	H	0.90969300	10.32287800	0.69290600
C	4.83349600	-7.70207900	0.49530900	C	3.14208800	8.22331400	-0.75453100
H	4.23679000	-5.63895400	0.64387100	H	2.91879400	6.07676900	-0.84221900
H	5.09069000	-9.81663400	0.18416300	H	3.04500400	10.35812800	-0.43553000
C	-0.63053200	8.29509700	0.19271100	C	0.85145900	-8.22199400	0.06797300
C	0.22483400	7.22903100	-0.10129400	C	1.51554300	-7.01092100	0.28431200
C	-0.08475800	9.58235600	0.13261800	C	1.61063100	-9.39194900	0.18481000
C	1.53975800	7.43289000	-0.48476300	C	2.85139800	-6.96566500	0.64637500
H	-0.14367900	6.21137800	-0.03387800	H	0.97797900	-6.07603400	0.17073500
C	1.23449800	9.79808700	-0.22586600	C	2.95207700	-9.35920700	0.52356700
H	-0.71169200	10.44092500	0.34686500	H	1.13724800	-10.35547700	0.03129200
C	2.06492800	8.72596600	-0.57136400	C	3.59170700	-8.14317000	0.79035500
H	2.16353500	6.58125300	-0.72703400	H	3.32362000	-6.00782000	0.82675700
H	1.62063600	10.80949500	-0.26565000	H	3.50616600	-10.28623400	0.60866300
C	4.35167700	7.92155600	-0.81505800	C	5.70430800	-6.94098300	0.94456000
C	4.67748600	7.47094900	0.46389300	C	5.95169200	-6.51914200	-0.36134300
C	4.98268600	7.34617400	-1.91774100	C	6.21273100	-6.19375300	2.00663000
C	5.61109400	6.45967900	0.63256900	C	6.68664800	-5.36691000	-0.59705700
H	4.18732600	7.91335400	1.32365500	H	5.55582500	-7.09606900	-1.18941500
C	5.91375900	6.33591200	-1.74201000	C	6.94405800	-5.04278500	1.76418600
H	4.73845300	7.69698700	-2.91382100	H	6.03006800	-6.52113300	3.02369300
C	6.24333800	5.86975700	-0.46591200	C	7.19129200	-4.60384100	0.45999800
H	5.84083400	6.10895900	1.63293700	H	6.85589200	-5.04263600	-1.61819300
H	6.40203200	5.90561500	-2.60961700	H	7.33903700	-4.47674600	2.60073400
C	7.22153600	4.77598400	-0.29564200	C	7.95002300	-3.35912500	0.22076200
C	7.16189100	3.63798400	-1.10478300	C	7.66377900	-2.20443000	0.95429500
C	8.23220800	4.84180600	0.66792600	C	8.97129200	-3.29289200	-0.73165500
C	8.07546700	2.60722900	-0.96225500	C	8.37054100	-1.03139000	0.75139900
H	6.37885900	3.55408000	-1.85055600	H	6.86519900	-2.22282900	1.68800000

C	9.15058000	3.81424900	0.81712900	C	9.68472300	-2.12273300	-0.94011400
H	8.30793400	5.71768700	1.30334000	H	9.22027100	-4.17619100	-1.31004900
C	9.08305700	2.68388600	0.00025500	C	9.39390900	-0.97691700	-0.19621800
H	8.01195500	1.73097800	-1.59697100	H	8.13140400	-0.14508900	1.32751900
H	9.93015000	3.88489300	1.56693800	H	10.47686700	-2.09202500	-1.67943000
C	6.72467800	-6.20925000	0.84841000	C	5.25259400	7.17487700	-1.30133600
C	7.07705000	-5.47499900	1.98041200	C	5.77767800	6.52132000	-2.42334700
C	6.95124900	-5.66075200	-0.41349500	C	5.59699700	6.71237800	-0.02574400
C	7.63340300	-4.21322100	1.84972300	C	6.60132400	5.42317900	-2.26663500
H	6.91098500	-5.89988600	2.96367200	H	5.51783900	6.87143700	-3.41544000
C	7.50932100	-4.39747200	-0.53697000	C	6.43738400	5.62401300	0.11862100
H	6.67721900	-6.22765300	-1.29596800	H	5.19290800	7.21190300	0.84698600
C	7.85510900	-3.64719600	0.59084800	C	6.94578600	4.94243900	-0.99581600
H	7.90893700	-3.65868300	2.74010700	H	6.99447700	4.92803000	-3.14781100
H	7.66195500	-3.97732300	-1.52520700	H	6.68040800	5.27343600	1.11576800
C	8.42193800	-2.28868200	0.46649800	C	7.78381300	3.74212500	-0.84414600
C	9.44331300	-2.00237400	-0.44400500	C	8.78687400	3.66215600	0.12986000
C	7.94493800	-1.24509700	1.26451600	C	7.58683000	2.62912000	-1.67045700
C	9.97117500	-0.72532000	-0.55327500	C	9.55942700	2.52144100	0.27447100
H	9.83905900	-2.79557500	-1.06920800	H	8.97293200	4.51344200	0.77580000
C	8.46653500	0.03341700	1.16126500	C	8.35083800	1.48429700	-1.52739800
H	7.14267800	-1.43581200	1.96900200	H	6.80477800	2.65414000	-2.42134000
C	9.48797800	0.30873100	0.25105600	C	9.34649000	1.41563300	-0.55181500
H	10.76566800	-0.52307400	-1.26227400	H	10.33484800	2.47978600	1.03096600
H	8.08126400	0.83127800	1.78552600	H	8.17562200	0.62734800	-2.16770500
C	-7.09477900	8.66470800	1.17935200	C	-5.46281200	-9.71201000	-0.80222700
C	-6.81731400	9.85339800	0.49593700	C	-5.01290400	-10.76645700	-0.00102400
C	-8.29099500	8.56603800	1.89816100	C	-6.62645100	-9.88918200	-1.55802500
C	-7.70846600	10.91550600	0.54859100	C	-5.70535000	-11.96836800	0.02761700
H	-5.90470100	9.94199400	-0.08047900	H	-4.12175600	-10.64016300	0.60162400
C	-9.18161500	9.62863600	1.93014600	C	-7.31985200	-11.08927100	-1.50831700
H	-8.51694100	7.65129400	2.43317800	H	-6.98225200	-9.08087300	-2.18580100
C	-8.89701100	10.81356100	1.26171800	C	-6.86380400	-12.14007600	-0.72093400
H	-7.47367200	11.82753100	0.01038000	H	-5.33977800	-12.77313100	0.65627000
H	-10.10265400	9.52951500	2.49427000	H	-8.21962800	-11.20509600	-2.10274000
H	-9.59194900	11.64464000	1.29448100	H	-7.40425600	-13.07904700	-0.69022500
C	3.73072800	10.05139600	-1.79638100	C	5.47743400	-9.08895200	2.04825000
C	2.85222600	10.56152500	-2.75672200	C	4.71087500	-9.68801400	3.05209800
C	4.98850600	10.64328100	-1.64882200	C	6.81898400	-9.45729600	1.91179000
C	3.22329900	11.64775500	-3.53642300	C	5.27465000	-10.64359900	3.88548600
H	1.87989800	10.10339400	-2.89181100	H	3.67402800	-9.40103900	3.17915700
C	5.35631100	11.71765800	-2.44525600	C	7.37747600	-10.40105600	2.76105500
H	5.67461800	10.25499200	-0.90528300	H	7.41955600	-8.99843400	1.13515800
C	4.47602500	12.23193800	-3.39013100	C	6.60974100	-11.00551100	3.74969800
H	2.52815700	12.02938400	-4.27636400	H	4.66417300	-11.09693500	4.65890400
H	6.33663700	12.16292700	-2.31514100	H	8.42015400	-10.67321300	2.63868100
H	4.76300800	13.07660100	-4.00576200	H	7.04676000	-11.74811000	4.40724500
C	11.38415200	1.85922900	0.17540000	C	11.50177100	0.26340200	-0.39732100
C	11.93649300	2.94356400	-0.51570900	C	12.24341700	-0.68402400	0.31829700
C	12.23760800	1.01768100	0.89799000	C	12.18805300	1.25350700	-1.11060200
C	13.30279600	3.17874500	-0.47451800	C	13.62970400	-0.64164100	0.30893800
H	11.29139700	3.59866700	-1.08854800	H	11.72981100	-1.45020600	0.88610500
C	13.60417400	1.25402900	0.91719500	C	13.57416400	1.29417900	-1.09791000
H	11.82509700	0.18022700	1.44755800	H	11.62993100	1.98864200	-1.67762900
C	14.14927200	2.33634900	0.23658200	C	14.30834600	0.34703600	-0.39372500
H	13.70856300	4.02503100	-1.01826900	H	14.18283800	-1.38610100	0.87147500
H	14.24621600	0.58877900	1.48442700	H	14.08349600	2.07085000	-1.65821100
H	15.21700900	2.52066700	0.26016400	H	15.39165700	0.37910300	-0.39220500
C	6.77716800	-8.43896400	1.80313600	C	4.62739500	9.25411700	-2.39251500
C	8.16184700	-8.60327600	1.70815300	C	5.93804400	9.71966400	-2.55211200
C	6.06706100	-9.20429100	2.73250700	C	3.60529000	9.80938900	-3.17137400
C	8.81802900	-9.51159300	2.52567200	C	6.21568800	10.71361800	-3.47665300
H	8.71869100	-8.01429200	0.98879600	H	6.72982500	9.30264500	-1.94138300
C	6.73060800	-10.12262000	3.53373500	C	3.89495500	10.81070100	-4.08492600
H	4.99558200	-9.07544000	2.82716200	H	2.59209600	9.44241400	-3.06281500
C	8.10817200	-10.28179700	3.43949400	C	5.19830800	11.26921700	-4.24615600
H	9.89292000	-9.62503800	2.43676500	H	7.23525200	11.06617400	-3.58650300
H	6.16326300	-10.70730600	4.24969400	H	3.09398400	11.22660800	-4.68610900
H	8.62239100	-10.99630900	4.07185400	H	5.41895300	12.05132700	-4.96329700
C	-3.81718900	-10.22388200	-1.86446800	C	-5.34795100	8.95368900	3.19551100
C	-4.84844600	-10.81252200	-1.13021900	C	-6.62028400	9.44681200	2.88955600
C	-3.22155300	-10.94187200	-2.90252500	C	-4.64544200	9.48204200	4.28266800
C	-5.28277700	-12.09314300	-1.44278000	C	-7.18087100	10.44460200	3.67123200
H	-5.30644900	-10.26243700	-0.31651800	H	-7.15445100	9.05359100	2.03307700
C	-3.64981600	-12.22836500	-3.19744900	C	-5.21399800	10.48434100	5.05260000
H	-2.42183600	-10.48404600	-3.47315800	H	-3.66310300	9.09192700	4.51992800
C	-4.68514800	-12.80891500	-2.47390400	C	-6.48300300	10.96964000	4.75413200
H	-6.08496000	-12.53864000	-0.86481900	H	-8.16544400	10.82443600	3.42269300
H	-3.17783900	-12.77445900	-4.00671500	H	-4.66439500	10.88139300	5.89855700
H	-5.02276500	-13.81138300	-2.71073400	H	-6.92416300	11.75317300	5.35924200
C	-11.23973800	-1.89906900	0.48810000	C	-11.04377200	0.21176000	-1.63254100
C	-11.68638300	-3.18876200	0.80445200	C	-11.44423300	1.38594400	-2.28027000
C	-12.19167500	-0.88419500	0.32467900	C	-11.93783800	-0.86326400	-1.57203200
C	-13.04154400	-3.44831100	0.94391900	C	-12.70948700	1.47916600	-2.84047500
H	-10.96737700	-3.98615800	0.94613400	H	-10.75810100	2.22140600	-2.34794800
C	-13.54257700	-1.15611400	0.48141300	C	-13.19421500	-0.76300700	-2.15041200
H	-11.87000700	0.11794300	0.06938400	H	-11.64389000	-1.77514100	-1.06630400
C	-13.98312000	-2.43793500	0.78830000	C	-13.59356600	0.40780600	-2.78455000
H	-13.36038500	-4.45558300	1.19009600	H	-12.99894300	2.39763600	-3.33955800
H	-14.25878300	-0.35216800	0.34882600	H	-13.87155900	-1.60807600	-2.09147900
H	-15.04045800	-2.64564300	0.90409600	H	-14.57903500	0.48414200	-3.22911000
N	6.13239400	-7.49035700	0.98112600	N	4.34976100	8.24370900	-1.45439100
N	10.00174400	1.62031200	0.14480200	N	10.10043600	0.22886700	-0.39962400
N	3.38598800	8.94310500	-0.99484600	N	4.93656100	-8.10730600	1.19174300
N	-3.35058300	-8.92254100	-1.55642400	N	-4.75954100	7.94702600	2.40270400
N	-9.87556500	-1.63109500	0.33908400	N	-9.76745600	0.10397300	-1.05739500
N	-6.21469500	7.56779000	1.14134200	N	-4.77988100	-8.48258100	-0.84739700

Structure S45. The coordinates of both the optimized structures of B7L$_2$ at S$_0$ and S$_1$ in toluene.

S_0-optimized structure				S_1-optimized structure			
B	11.16996600	4.36833000	0.53616200	B	-11.18555700	-4.29914700	0.48226000
C	9.99620800	5.27670000	0.04312500	C	-10.03169100	-5.18718500	-0.08883700
C	9.16641000	4.88120900	-1.01815800	C	-9.20671400	-4.73160600	-1.12965000
C	9.70336300	6.51076000	0.64599700	C	-9.75151500	-6.46399800	0.42441900
C	8.11047900	5.66490800	-1.45094700	C	-8.16574300	-5.49795100	-1.62545200
H	9.35358000	3.93545900	-1.51519600	H	-9.38505700	-3.75159000	-1.55892200
C	8.63197400	7.28867200	0.23577100	C	-8.69546400	-7.22662900	-0.04905700
H	10.32201100	6.86132500	1.46539400	H	-10.36753200	-6.86133700	1.22416900
C	7.81376900	6.87943800	-0.82267700	C	-7.88027400	-6.75629600	-1.08417900
H	7.48096800	5.31273800	-2.26052100	H	-7.53787900	-5.09945000	-2.41463400
H	8.43946100	8.23637200	0.72681500	H	-8.51217800	-8.20841700	0.37381400
C	6.64956300	7.67753200	-1.25492400	C	-6.72772700	-7.53601400	-1.57665000
C	6.26035900	7.71035800	-2.59779600	C	-6.34186600	-7.47994500	-2.91975200
C	5.87951500	8.39233700	-0.33038700	C	-5.96284900	-8.32038100	-0.70588800
C	5.13050500	8.40786800	-2.99218400	C	-5.21803700	-8.15728700	-3.36414500
H	6.85883600	7.19771300	-3.34271000	H	-6.93745400	-6.91124100	-3.62530000
C	4.73584300	9.06554400	-0.72896600	C	-4.82568500	-8.97349300	-1.15276800
H	6.15403300	8.38003200	0.71873300	H	-6.23499200	-8.37810300	0.34232800
C	4.31623500	9.08264700	-2.06878900	C	-4.40721600	-8.89987900	-2.49110800
H	4.85825000	8.41322700	-4.04215000	H	-4.94693700	-8.09102500	-4.41239400
H	4.14791100	9.58567200	0.02004900	H	-4.24028600	-9.54844700	-0.44296800
C	11.02763100	2.81313200	0.43481000	C	-11.02395700	-2.74253300	0.48729400
C	9.78577500	2.17620100	0.59005900	C	-9.77143300	-2.13181000	0.66156700
C	12.12946500	1.98776000	0.15958800	C	-12.12027100	-1.88727000	0.29279600
C	9.65032100	0.80190000	0.48194900	C	-9.62035700	-0.75494700	0.64456000
H	8.90546800	2.77339300	0.80261900	H	-8.89484900	-2.75208500	0.81521300
C	11.99667800	0.61545600	0.01919000	C	-11.97272800	-0.51026200	0.24499400
H	13.11093600	2.43436700	0.04004600	H	-13.10961600	-2.31309100	0.16290200
C	10.75295900	-0.00441100	0.18057100	C	-10.71793300	0.08302800	0.42098100
H	8.67100200	0.34988700	0.59532400	H	-8.63320000	-0.32319900	0.76792300
H	12.87242200	0.01130700	-0.19140500	H	-12.84486100	0.11670700	0.09501000
C	10.60495500	-1.46569200	0.02931000	C	-10.55447400	1.54926300	0.35908700
C	9.71089900	-2.18715600	0.82771600	C	-9.62744200	2.21088100	1.17199200
C	11.35170000	-2.16824900	-0.92270800	C	-11.32023000	2.31740000	-0.52463200
C	9.56438000	-3.55592700	0.66961900	C	-9.46693500	3.58503000	1.09179600
H	9.14168800	-1.67219600	1.59390500	H	-9.04187500	1.64433700	1.88779600
C	11.21917100	-3.54062900	-1.05594200	C	-11.17369500	3.69361100	-0.57858300
H	12.02753200	-1.62874700	-1.57723200	H	-12.02228100	1.82677100	-1.18977800
C	10.31939000	-4.27754000	-0.26889100	C	-10.23928000	4.37087900	0.22144300
H	8.86404100	-4.08489100	1.30739800	H	-8.74000700	4.06610500	1.73781400
H	11.81405600	-4.05413900	-1.80376700	H	-11.78441800	4.25834300	-1.27485900
B	10.17880000	-5.82764900	-0.41665600	B	-10.07812600	5.92454200	0.14609600
B	2.97481800	9.73675500	-2.54030400	B	-3.06607100	-9.52536300	-3.00134800
C	1.70565600	9.69136600	-1.62766700	C	-1.80552000	-9.54345800	-2.07560000
C	0.64153900	10.59385500	-1.79650800	C	-0.74893700	-10.44835400	-2.27619800
C	1.55550900	8.72365000	-0.61982800	C	-1.66132000	-8.63388100	-1.01421800
C	-0.50715300	10.52387100	-1.02494200	C	0.38472800	-10.43891500	-1.47914600
H	0.72083200	11.37311200	-2.54659700	H	-0.82375800	-11.18341400	-3.07018800
C	0.41582900	8.65406700	0.16533900	C	-0.53640100	-8.62377200	-0.20592500
H	2.33919900	7.99033600	-0.46610700	H	-2.43864200	-7.89989300	-0.83392700
C	-0.64019600	9.54738300	-0.03334600	C	0.51014800	-9.52234700	-0.43088300
H	-1.30689600	11.24109300	-1.17651100	H	1.17653800	-11.15960700	-1.65410800
H	0.32102100	7.87637600	0.91577100	H	-0.44591800	-7.88974000	0.58769800
C	-1.88040000	9.43629400	0.76208300	C	1.72714100	-9.47571900	0.40477200
C	-3.12351400	9.40282300	0.12425200	C	2.99357000	-9.45366600	-0.18662200
C	-1.84284600	9.33339000	2.15518300	C	1.64178300	-9.41742600	1.79845300
C	-4.28578600	9.22401700	0.85688100	C	4.13214200	-9.32283100	0.59250400
H	-3.16863200	9.46761800	-0.95773200	H	3.07706500	-9.48749900	-1.26777000
C	-3.01696700	9.22506900	2.88677800	C	2.78998200	-9.35428600	2.57405000
H	-0.88655700	9.36836600	2.66647600	H	0.66721500	-9.44876600	2.27421000
C	-4.26789400	9.14546400	2.25755000	C	4.06467800	-9.27865000	1.99335500
H	-5.23242500	9.14655700	0.33205600	H	5.09884600	-9.25277100	0.10433600
H	-2.96080300	9.17924800	3.96969900	H	2.69550200	-9.33869100	3.65507200
C	8.80688300	-6.52624900	-0.13922900	C	-8.68455600	6.59169600	0.38826600
C	7.58130300	-5.91518600	-0.44951100	C	-7.48362400	5.96375600	0.01974000
C	8.74759600	-7.80576900	0.43540100	C	-8.57727000	7.86489900	0.97076000
C	6.36999800	-6.54972200	-0.22060500	C	-6.25354100	6.57786400	0.19618900
H	7.57884600	-4.92352600	-0.88985500	H	-7.51661800	4.97640600	-0.42876700
C	7.54054500	-8.43032200	0.70205200	C	-7.35016500	8.47165700	1.17927500
H	9.67016500	-8.31920600	0.68456800	H	-9.47861200	8.39008500	1.26816600
C	6.32799700	-7.81700500	0.36971000	C	-6.16337200	7.84441400	0.78377400
H	5.44298000	-6.04567800	-0.47203500	H	-5.34803400	6.06011900	-0.10109200
H	7.53490500	-9.42200900	1.14095900	H	-7.31086100	9.46242500	1.61838300
C	5.04725500	-8.50261000	0.63478300	C	-4.86321000	8.51950000	0.96687600
C	3.98142500	-8.42874900	-0.26826000	C	-3.84037900	8.39035000	0.02078800
C	4.87649100	-9.26484600	1.79524200	C	-4.62904400	9.33400000	2.08070600
C	2.79526900	-9.10328800	-0.02155800	C	-2.63945400	9.06472100	0.17706600
H	4.09758600	-7.86337700	-1.18659900	H	-4.00292700	7.78313400	-0.86301400
C	3.69587600	-9.95047200	2.02477400	C	-3.43591600	10.02267600	2.21737600
H	5.67484400	-9.30842000	2.52801400	H	-5.38859600	9.41982500	2.84999600
C	2.61936300	-9.89639100	1.12396900	C	-2.40539500	9.91761500	1.26812000
H	1.99492900	-9.03921400	-0.75133800	H	-1.87489700	8.95574100	-0.58504000
H	3.59886300	-10.53617900	2.93243200	H	-3.29230400	10.65323300	3.08818700
B	-5.60198900	8.94402600	3.05254300	B	5.35663300	-9.09897200	2.86041100
B	1.31294000	-10.71542100	1.38354300	B	-1.09064900	10.75415200	1.39438000
C	-6.61559500	7.88063000	2.51406300	C	6.40585800	-8.03197800	2.41140900
C	-8.00533000	8.07390800	2.50574900	C	7.78964100	-8.17614100	2.60979000
C	-6.13816500	6.66010400	2.01221100	C	5.97452100	-6.84092700	1.80322500
C	-8.86931500	7.11301200	2.00063500	C	8.68748700	-7.19616700	2.22053600
H	-8.41665500	9.00234600	2.88867900	H	8.16861900	-9.08074300	3.07393000
C	-6.99845900	5.67169500	1.56407700	C	6.85989400	-5.83669600	1.45614400
H	-5.06867000	6.48021300	1.98191100	H	4.91652300	-6.69697600	1.61219000
C	-8.37899900	5.88871700	1.53687700	C	8.23788400	-5.99508900	1.65437500
H	-9.93872700	7.29592600	1.99473700	H	9.74829500	-7.33952800	2.39531700

H	-6.59907800	4.73519600	1.18974600
C	-9.27983100	4.84319700	1.01099400
C	-9.11976600	3.50842300	1.39600500
C	-10.29025700	5.15290400	0.09663300
C	-9.92607800	2.51707600	0.86185500
H	-8.36427500	3.25361800	2.13156500
C	-11.09201400	4.15390300	-0.43361700
H	-10.42087300	6.18039300	-0.22606700
C	-10.92697200	2.80378700	-0.08148900
H	-9.78327700	1.49395300	1.19194200
H	-11.84951700	4.42267900	-1.16187200
C	-0.07717300	-10.22632400	0.86022100
C	-1.07875900	-11.14771500	0.51394100
C	-0.40525500	-8.86603500	0.74062700
C	-2.32877600	-10.74064900	0.08052100
H	-0.87227100	-12.20962300	0.59150400
C	-1.66313800	-8.45037000	0.33116400
H	0.33734100	-8.11616900	0.99252200
C	-2.65170800	-9.38191700	-0.00484800
H	-3.08127000	-11.48528100	-0.15356800
H	-1.87720800	-7.38999600	0.25334300
C	-4.01262600	-8.96552400	-0.40032800
C	-4.61991900	-7.84186700	0.17009400
C	-4.75233600	-9.71451500	-1.32339200
C	-5.92481800	-7.50065400	-0.15098100
H	-4.07799700	-7.25320200	0.90214000
C	-6.06529300	-9.38565800	-1.61467700
H	-4.29103800	-10.56290600	-1.81680600
C	-6.69807400	-8.27742600	-1.02826800
H	-6.37121800	-6.63221600	0.32200000
H	-6.61525300	-9.99995700	-2.31972000
B	-11.79353300	1.68165200	-0.74375500
B	-8.21004500	-7.96118500	-1.26582800
C	-11.22847400	0.23010400	-0.89596300
C	-12.08375200	-0.88380700	-0.88742300
C	-9.85513700	-0.03977400	-1.01633500
C	-11.60172700	-2.17977500	-0.96525900
H	-13.15434900	-0.72961100	-0.80752700
C	-9.36564300	-1.33291200	-1.09900100
H	-9.15149700	0.78495300	-1.04601600
C	-10.22879600	-2.43434900	-1.06273600
H	-12.30447000	-3.00503000	-0.97095300
H	-8.29476200	-1.48972800	-1.16103900
C	-9.71203200	-3.81798000	-1.11546600
C	-8.49872800	-4.12151000	-1.74460600
C	-10.42202200	-4.87158900	-0.52790700
C	-8.01044300	-5.41871600	-1.76494600
H	-7.93663900	-3.33606300	-2.23695400
C	-9.95449600	-6.17323000	-0.59891100
H	-11.34468000	-4.66948700	0.00380000
C	-8.73055700	-6.48676300	-1.20917300
H	-7.05866300	-5.61350100	-2.24877500
H	-10.54228600	-6.96620200	-0.14847400
C	12.47416700	5.00419000	1.11917900
C	13.21352500	4.36048900	2.12506000
C	12.95537400	6.24080200	0.65873700
C	14.36442300	4.92694600	2.65575900
H	12.86936700	3.40455700	2.50581100
C	14.12090500	6.80028700	1.16438400
H	12.41185200	6.76379500	-0.12116500
C	14.82389200	6.14625900	2.17020800
H	14.90753000	4.41634100	3.44367400
H	14.47946400	7.74885800	0.77949000
H	15.72898200	6.58629600	2.57512200
C	11.41728200	-6.68991800	-0.83156900
C	12.71982600	-6.33868400	-0.44072700
C	11.27055800	-7.85028900	-1.60931100
C	13.81769000	-7.11118000	-0.79484400
H	12.87041600	-5.45126100	0.16499300
C	12.36643400	-8.61162900	-1.99231900
H	10.27869100	-8.15138900	-1.92986800
C	13.64272600	-8.24619900	-1.57872700
H	14.81106300	-6.82638600	-0.46521000
H	12.22627800	-9.49387000	-2.60752500
H	14.49995100	-8.84550400	-1.86646700
C	1.39795000	-12.06067000	2.18326900
C	2.49295600	-12.92844000	2.04097600
C	0.38169400	-12.44964400	3.07115800
C	2.56396400	-14.12902500	2.73480400
H	3.29602500	-12.66181600	1.36183900
C	0.46084800	-13.63318500	3.79279100
H	-0.47945500	-11.80374900	3.20728500
C	1.55015200	-14.47970900	3.61930600
H	3.41276100	-14.78908000	2.59186700
H	-0.33010300	-13.90014000	4.48535400
H	1.60858300	-15.41113400	4.17226700
C	-9.22755400	-9.12171100	-1.52249400
C	-9.05021800	-10.39886700	-0.96519700
C	-10.36717300	-8.92073700	-2.31898200
C	-9.96581400	-11.41955000	-1.18234500
H	-8.18695100	-10.58803400	-0.33609600
C	-11.27202600	-9.94436000	-2.56437700
H	-10.53710000	-7.94559700	-2.76313900
C	-11.07520300	-11.19538200	-1.99009200
H	-9.81367500	-12.39168800	-0.72593800
H	-12.13486300	-9.76640400	-3.19706000
H	-11.78674300	-11.99416900	-2.17012500
H	6.48764000	-4.93096600	0.99039200
C	9.17226700	-4.92479400	1.28076600
C	8.81419300	-3.57752400	1.43929400
C	10.44036700	-5.20624400	0.75219300
C	9.67879400	-2.56491100	1.07339900
H	7.85719600	-3.33015900	1.88572300
C	11.29911300	-4.18611900	0.38709400
H	10.73427400	-6.23815500	0.59250200
C	10.95019400	-2.82510800	0.52044200
H	9.37350700	-1.53774900	1.24087300
H	12.26169200	-4.44245200	-0.04180500
C	0.26103200	10.22352200	0.81749800
C	1.21480400	11.10572800	0.28015900
C	0.59899200	8.85897600	0.82477800
C	2.42146500	10.65837800	-0.22533400
H	1.00006000	12.16874400	0.25907200
C	1.82200900	8.40690400	0.35920600
H	-0.10993800	8.13934900	1.22101900
C	2.76094500	9.29809200	-0.18134900
H	3.13467300	11.37524700	-0.61637000
H	2.04484700	7.34590300	0.38411600
C	4.06902400	8.84002300	-0.66547200
C	4.73719700	7.76525000	-0.05723900
C	4.71186900	9.48370100	-1.73527600
C	5.99287300	7.37593400	-0.48236600
H	4.28103600	7.26373900	0.78936900
C	5.96764700	9.09011800	-2.15184800
H	4.20395500	10.28681700	-2.25777200
C	6.66514300	8.02541200	-1.54028800
H	6.48978800	6.56787900	0.04374500
H	6.42669700	9.60805500	-2.98665100
B	11.92276300	-1.68326700	0.09523600
B	8.11266300	7.65028600	-1.97699200
C	11.36501800	-0.28941200	-0.31747100
C	12.11907100	0.90041600	-0.12465500
C	10.07459000	-0.11429100	-0.88971900
C	11.62722800	2.14172400	-0.42820700
H	13.10990300	0.82308400	0.30804600
C	9.56689700	1.11938000	-1.19741400
H	9.47498100	-0.99160800	-1.10335900
C	10.31595300	2.31286500	-0.96681300
H	12.23655700	3.00891500	-0.21036900
H	8.58680700	1.18089700	-1.65225200
C	9.77872900	3.60671300	-1.24569900
C	8.38847500	3.80519700	-1.50725000
C	10.59867100	4.77516900	-1.26236700
C	7.87278500	5.05520900	-1.72465200
H	7.71432900	2.95863300	-1.49617400
C	10.07237400	6.01285400	-1.51619500
H	11.66529100	4.68647400	-1.10378500
C	8.68337200	6.22315700	-1.73729500
H	6.80849100	5.15616500	-1.90519300
H	10.73606900	6.86966700	-1.53290800
C	-12.48993500	-4.95757300	1.03974000
C	-13.20369000	-4.37833900	2.10175200
C	-12.99649700	-6.15123500	0.49993200
C	-14.35411900	-4.96612300	2.60983700
H	-12.83948700	-3.45726200	2.54468100
C	-14.16180900	-6.73008400	0.98382900
H	-12.47304800	-6.62427200	-0.32435400
C	-14.83901500	-6.14103500	2.04591400
H	-14.87704300	-4.50621800	3.44141800
H	-14.54021900	-7.64353300	0.53783500
H	-15.74379300	-6.59699500	2.43352800
C	-11.32108900	6.81921200	-0.17903800
C	-12.60859900	6.46917200	0.25976800
C	-11.19603700	8.00394800	-0.92304000
C	-13.71171600	7.26603800	-0.01516600
H	-12.74245500	5.56175300	0.83927000
C	-12.29875700	8.79054100	-1.22755600
H	-10.21685400	8.30444000	-1.28094700
C	-13.55894500	8.42581000	-0.76679700
H	-14.69219600	6.98073000	0.35062600
H	-12.17608000	9.69168100	-1.81864500
H	-14.42081100	9.04431000	-0.99355300
C	-1.12770000	12.15585200	2.09292000
C	-2.24609800	12.99956300	1.99066500
C	-0.03929800	12.62237700	2.84855000
C	-2.27299600	14.24785400	2.59783000
H	-3.10362800	12.67529300	1.41048800
C	-0.07042800	13.85652300	3.48351500
H	0.84194300	11.99751500	2.94974400
C	-1.18658000	14.67547500	3.35293300
H	-3.14300200	14.88625100	2.48768200
H	0.77782400	14.18403100	4.07493500
H	-1.20929400	15.64543400	3.83810400
C	9.03394200	8.73591500	-2.63919200
C	9.01160700	10.07802600	-2.21999700
C	9.92459500	8.41225200	-3.67802000
C	9.83605000	11.03703400	-2.79299500
H	8.34442700	10.36944400	-1.41535700
C	10.73726900	9.36902000	-4.27034900
H	9.96933800	7.38869200	-4.03698200
C	10.69883100	10.68649200	-3.82562000
H	9.80507500	12.06081000	-2.43518100
H	11.40403000	9.08811300	-5.07881500
H	11.33836700	11.43512600	-4.28079700

C	-13.23608900	2.01127000	-1.25725400	C	13.47226400	-1.92511700	0.10116800
C	-14.07489600	2.89778600	-0.56271700	C	14.08804400	-2.72190300	1.08302800
C	-13.74437500	1.43264800	-2.43146300	C	14.31335400	-1.35765600	-0.87255200
C	-15.35979800	3.18061500	-1.00644400	C	15.46055100	-2.92948000	1.10119500
H	-13.71573300	3.36198200	0.34998100	H	13.47620600	-3.17300000	1.85745000
C	-15.01656800	1.73449400	-2.89850100	C	15.68393100	-1.57651600	-0.87322500
H	-13.12338500	0.74379900	-2.99481500	H	13.87815900	-0.74323400	-1.65450200
C	-15.83019100	2.60441700	-2.18115000	C	16.26373600	-2.36098500	0.11848400
H	-15.99307900	3.85553800	-0.44073600	H	15.90589100	-3.53709300	1.88194600
H	-15.37761800	1.28646800	-3.81799100	H	16.30292600	-1.13390600	-1.64655800
H	-16.82924600	2.83273300	-2.53681700	H	17.33550800	-2.52798000	0.12533300
C	2.90612600	10.38344500	-3.96548000	C	-2.98804400	-10.07636400	-4.46508400
C	1.77098800	10.26479400	-4.78339500	C	-1.83883300	-9.92310600	-5.25734000
C	3.99733400	11.10131700	-4.48126200	C	-4.08313900	-10.73887900	-5.04313200
C	1.72849000	10.82632400	-6.05270500	C	-1.78646100	-10.39900100	-6.56075400
H	0.91297500	9.70663900	-4.42353500	H	-0.97733000	-9.40544100	-4.84868600
C	3.95055600	11.69281200	-5.73643100	C	-4.02761000	-11.24474800	-6.33479200
H	4.89466000	11.20494600	-3.87984100	H	-4.99057100	-10.86783800	-4.46215200
C	2.81611600	11.55048600	-6.52776000	C	-2.87906300	-11.06970000	-7.09878500
H	0.84535200	10.70423400	-6.67054600	H	-0.89207400	-10.25161400	-7.15649900
H	4.80152000	12.25695300	-6.10268900	H	-4.88224000	-11.76769800	-6.75030800
H	2.78112800	12.00008600	-7.51434100	H	-2.83674400	-11.45244200	-8.11289900
C	-5.89600200	9.76368100	4.34635300	C	5.55664500	-9.93832000	4.16143400
C	-5.27366300	11.00147500	4.57985600	C	4.95541400	-11.19990400	4.30678500
C	-6.78846900	9.29584200	5.32540700	C	6.33146200	-9.46724200	5.23462600
C	-5.53739900	11.74185400	5.72359600	C	5.13232200	-11.96092400	5.45379300
H	-4.58076600	11.39258000	3.84241700	H	4.34845300	-11.59256900	3.49778500
C	-7.04131200	10.02035500	6.48174800	C	6.49387800	-10.21211200	6.39441600
H	-7.27988400	8.33967000	5.18053700	H	6.79981600	-8.49146900	5.16217200
C	-6.41919700	11.24845800	6.67921200	C	5.89913200	-11.46459800	6.50248200
H	-5.05386400	12.70095200	5.87420800	H	4.66879000	-12.93815100	5.53486800
H	-7.72484500	9.63131800	7.22860800	H	7.08565000	-9.81925000	7.21411000
H	-6.62117000	11.82067700	7.57844000	H	6.03135700	-12.05248300	7.40454100

Structure S46. The coordinates of both the optimized structures of B6N1L$_2$ at S$_0$ and S$_1$ in toluene.

S_0-optimized structure				S_1-optimized structure			
B	-10.94516900	-4.78352600	0.42216000	B	-10.80710300	-4.97364000	0.60465100
C	-9.73822100	-5.64315000	-0.07852800	C	-9.61201100	-5.82037100	0.05646500
C	-8.91184000	-5.20233000	-1.12447000	C	-8.82231600	-5.36501500	-1.01148300
C	-9.41024700	-6.87684300	0.50671800	C	-9.25835800	-7.05800200	0.61816800
C	-7.82460300	-5.94188500	-1.55761800	C	-7.74526200	-6.09369100	-1.48630800
H	-9.12650200	-4.25565300	-1.60846600	H	-9.05806700	-4.41533000	-1.47949000
C	-8.30843900	-7.61074500	0.09614200	C	-8.16706600	-7.78192600	0.16442200
H	-10.02562200	-7.26202000	1.31291000	H	-9.84472400	-7.45458900	1.44025300
C	-7.49256500	-7.15533900	-0.94504300	C	-7.38701700	-7.31155600	-0.89747300
H	-7.19867600	-5.55481100	-2.35401000	H	-7.14733000	-5.69448900	-2.29802500
H	-8.08918800	-8.55983400	0.57314000	H	-7.92808100	-8.73442800	0.62472800
C	-6.29492800	-7.90427200	-1.37380300	C	-6.19848000	-8.04786100	-1.37104500
C	-5.88760300	-7.90318900	-2.71165400	C	-5.82531300	-8.02009600	-2.71858700
C	-5.51110700	-8.60390000	-0.44921400	C	-5.38765300	-8.76037800	-0.48013600
C	-4.72770600	-8.55282200	-3.10070000	C	-4.67075600	-8.65330800	-3.14854800
H	-6.49479600	-7.40142000	-3.45692900	H	-6.45443700	-7.50924100	-3.43905800
C	-4.33876200	-9.22934600	-0.84205100	C	-4.22166100	-9.37025400	-0.91393500
H	-5.79904600	-8.61670200	0.59627500	H	-5.64860200	-8.79512400	0.57190500
C	-3.90125900	-9.21107200	-2.17614300	C	-3.81647400	-9.32200200	-2.25745300
H	-4.44157500	-8.53219100	-4.14677300	H	-4.41072200	-8.61011000	-4.20073300
H	-3.74188700	-9.73878200	-0.09279500	H	-3.60322300	-9.88992300	-0.18965800
C	-10.85463600	-3.22277200	0.35641600	C	-10.73537400	-3.41190300	0.54192500
C	-9.63373600	-2.54835200	0.52028400	C	-9.51767200	-2.72225100	0.66211300
C	-11.98525700	-2.42744700	0.11047800	C	-11.88402300	-2.62978000	0.34038500
C	-9.54516300	-1.16799300	0.44771800	C	-9.44874200	-1.34087400	0.58842400
H	-8.73235900	-3.12058700	0.71200300	H	-8.60283800	-3.28351500	0.81953900
C	-11.90012700	-1.04805000	0.00892400	C	-11.81971300	-1.24962700	0.23788100
H	-12.95200400	-2.90345100	-0.01523300	H	-12.84894900	-3.11740800	0.25045000
C	-10.67668400	-0.39075600	0.17832200	C	-10.59880400	-0.57678500	0.36156900
H	-8.58046600	-0.68691000	0.56613400	H	-8.48605900	-0.84846400	0.67150400
H	-12.79803300	-0.46878300	-0.17668500	H	-12.73141300	-0.68175900	0.08809600
C	-10.58026700	1.07875900	0.07142900	C	-10.52640200	0.89285400	0.25045600
C	-9.69126300	1.80390000	0.87216100	C	-9.61224700	1.63326300	1.00898100
C	-11.37421500	1.78700400	-0.83730500	C	-11.37190900	1.58971700	-0.62059400
C	-9.59644100	3.18169700	0.75898400	C	-9.54658700	3.01230700	0.89452600
H	-9.08515100	1.28416800	1.60606300	H	-8.96526200	1.12377000	1.71471900
C	-11.29510800	3.16734200	-0.92296400	C	-11.31872500	2.97033800	-0.70903400
H	-12.04657300	1.24694400	-1.49491100	H	-12.06446500	1.03963200	-1.24827000
C	-10.40199500	3.90814900	-0.13226700	C	-10.40376200	3.72800200	0.04179000
H	-8.89751800	3.71319500	1.39626100	H	-8.83101800	3.55417800	1.50425200
H	-11.92831200	3.68503500	-1.63569700	H	-11.99110100	3.47696200	-1.39312900
B	-10.32240800	5.46785900	-0.21982000	B	-10.36566200	5.28699800	-0.04799400
B	-2.52979800	-9.80788500	-2.63707200	B	-2.44905800	-9.89289600	-2.76204000
C	-1.27778000	-9.73186600	-1.70284300	C	-1.17784300	-9.81983700	-1.85417500
C	-0.18055000	-10.59495800	-1.86570700	C	-0.07212500	-10.66268200	-2.06027600
C	-1.17751700	-8.77351100	-0.68000100	C	-1.06992000	-8.88524300	-0.81025600
C	0.95244200	-10.49605600	-1.07427000	C	1.07473600	-10.56799600	-1.28836800
H	-0.22069800	-11.36610100	-2.62723000	H	-0.11686200	-11.41509200	-2.84006600
C	-0.05357000	-8.67517200	0.12449400	C	0.06750300	-8.79237500	-0.02455400
H	-1.98850700	-8.06976100	-0.52970400	H	-1.88653700	-8.19597500	-0.62721400
C	1.03584300	-9.52878300	-0.06838500	C	1.16382300	-9.62654000	-0.25882600
H	1.77893200	-11.18318100	-1.22184600	H	1.90750000	-11.23939100	-1.46927800
H	0.00246500	-7.90436600	0.88584300	H	0.12806300	-8.04085200	0.75548400
C	2.25946500	-9.38303000	0.74678000	C	2.39776100	-9.48711100	0.54178000
C	3.50943700	-9.29748900	0.12745900	C	3.63703800	-9.36000400	-0.09152700
C	2.19833300	-9.29536100	2.14006700	C	2.35469800	-9.44619000	1.93783300
C	4.65344100	-9.08100600	0.87840500	C	4.78778300	-9.14792200	0.65068400
H	3.57223500	-9.35046300	-0.95429100	H	3.68544600	-9.37702700	-1.17520600
C	3.35696700	-9.14899500	2.88957200	C	3.52112800	-9.30448500	2.67588300
H	1.23689400	-9.37130200	2.63706500	H	1.40160700	-9.55486000	2.44480300
C	4.61257400	-9.01484300	2.27927000	C	4.76526900	-9.12792800	2.05326800
H	5.60367200	-8.96230600	0.36799600	H	5.72867000	-8.99592400	0.13182600
H	3.28374000	-9.11494100	3.97190600	H	3.46293000	-9.30772000	3.75967400
C	-8.96583800	6.20255500	0.02811300	C	-9.02413400	6.06355100	0.15596400
C	-7.73578600	5.64895600	-0.36304500	C	-7.78384500	5.54090900	-0.25336800
C	-8.92098200	7.45906100	0.65336600	C	-9.00301100	7.33959900	0.74690300
C	-6.53718800	6.31638900	-0.16423600	C	-6.60587800	6.25348400	-0.11288500
H	-7.72070000	4.67596400	-0.84325600	H	-7.74840000	4.55602500	-0.70688500
C	-7.72445100	8.11537300	0.88816800	C	-7.82719200	8.04279800	0.92779400
H	-9.84715800	7.92906000	0.96704700	H	-9.93552300	7.78648500	1.07415100
C	-6.50751200	7.56136100	0.47415800	C	-6.60007900	7.52207800	0.48765700
H	-5.60696300	5.85456400	-0.47790000	H	-5.67263100	5.80905800	-0.44125100
H	-7.73202500	9.08922600	1.36565500	H	-7.86324900	9.03030400	1.37402100
C	-5.24141200	8.28174500	0.70290200	C	-5.36751500	8.29881800	0.63660200
C	-4.21329800	8.27914000	-0.24492600	C	-4.31559200	8.19887600	-0.29169800
C	-5.03430000	9.01670600	1.87443300	C	-5.20601400	9.20349800	1.70232100
C	-3.04653500	9.00063500	-0.05190000	C	-3.18231600	8.97650800	-0.18874800
H	-4.34809000	7.74240000	-1.17774700	H	-4.41758000	7.53970600	-1.14521900
C	-3.86349000	9.72035400	2.08916900	C	-4.07967200	9.98342000	1.82273800
H	-5.79234700	9.01186700	2.64991900	H	-5.96732800	9.26669700	2.47011500
C	-2.85421000	9.73915800	1.12037500	C	-3.05200700	9.89751100	0.86595600
H	-2.28770700	9.01110800	-0.82505600	H	-2.41424000	8.91993700	-0.95020800
H	-3.72544100	10.26373500	3.01585200	H	-3.96969300	10.64823400	2.67009700
B	5.92514300	-8.76297200	3.09556300	B	6.08272500	-8.87837400	2.86304200
C	6.89927200	-7.65320200	2.57821100	C	7.02603500	-7.72754100	2.38339500
C	8.29624700	-7.78427100	2.59085600	C	8.42646200	-7.81741700	2.40304200
C	6.37576700	-6.45338700	2.07231400	C	6.47142400	-6.52682000	1.91324600
C	9.12415500	-6.78448100	2.10097700	C	9.22769900	-6.77589400	1.95891300
H	8.74273700	-8.69467800	2.97769000	H	8.89709600	-8.72750100	2.76095800
C	7.19781600	-5.42680100	1.63867600	C	7.26578000	-5.46142300	1.52400000
H	5.29995000	-6.32097300	2.02628000	H	5.39250700	-6.42475700	1.86211900
C	8.58689300	-5.58235200	1.63123900	C	8.65931400	-5.57297100	1.52822400
H	10.20057800	-6.91972600	2.11092400	H	10.30758000	-6.87852500	1.97674100
H	6.76260900	-4.50855300	1.25922600	H	6.80632700	-4.54392100	1.17236000

C	9.44708300	-4.49790300	1.11646800	C	9.49377700	-4.44320000	1.07342000
C	9.21894400	-3.17058100	1.49293000	C	9.20883800	-3.13754900	1.48551500
C	10.48445000	-4.76339900	0.21855000	C	10.57009100	-4.64254200	0.20452500
C	9.98424100	-2.14437600	0.96433700	C	9.96009100	-2.06960700	1.02302000
H	8.44186100	-2.94869200	2.21653400	H	8.40094100	-2.96563300	2.18887600
C	11.24519200	-3.72977800	-0.30580800	C	11.31568200	-3.56735500	-0.25369100
H	10.66816300	-5.78498800	-0.09697200	H	10.79903700	-5.64569600	-0.13962900
C	11.01010300	-2.38738700	0.03569400	C	11.02724800	-2.24582000	0.12637800
H	9.78801500	-1.12762000	1.28659300	H	9.72141200	-1.07196600	1.37508200
H	12.02506700	-3.96549200	-1.02179400	H	12.13007700	-3.75289700	-0.94578400
C	-0.43867900	10.11781500	0.84005200	C	-0.67331200	10.31704600	0.54623100
C	0.47063000	11.08181500	0.39348700	C	0.21122800	11.25067400	-0.03297300
C	-0.04446000	8.77592700	0.81839000	C	-0.23214700	8.98759200	0.71317000
C	1.73506600	10.71635400	-0.03033900	C	1.47630400	10.87632600	-0.40182200
H	0.18756600	12.12724400	0.39784600	H	-0.11883400	12.26933100	-0.19518700
C	1.21935200	8.42058100	0.37601900	C	1.04056600	8.62723200	0.34530400
H	-0.72988900	8.00836300	1.15713700	H	-0.88954300	8.25684000	1.16854000
C	2.14567700	9.37933800	-0.04762000	C	1.95825300	9.55452700	-0.21438500
H	2.42651300	11.49435200	-0.33303800	H	2.12700700	11.63098800	-0.82220900
H	1.48847200	7.37023600	0.36177200	H	1.34226300	7.59916300	0.49765000
C	3.51726400	9.02015700	-0.45715800	C	3.32178800	9.19785200	-0.53980600
C	4.19295500	7.94350000	0.12875400	C	3.92866100	8.01685000	-0.04719800
C	4.20405900	9.77715200	-1.41517000	C	4.14126900	10.04152000	-1.32944800
C	5.50600500	7.65708900	-0.21050200	C	5.25446000	7.73511300	-0.28627600
H	3.69715200	7.34792100	0.88748000	H	3.36207700	7.33831500	0.57971800
C	5.52678900	9.50797000	-1.72138300	C	5.46624400	9.75363500	-1.55289600
H	3.69293900	10.58639100	-1.92495700	H	3.72030900	10.92482800	-1.79496200
C	6.22600700	8.44801900	-1.12049300	C	6.10022300	8.60064400	-1.02458700
H	6.00257000	6.82358100	0.27534000	H	5.67935900	6.83720200	0.14960900
H	6.03156900	10.13007500	-2.45301200	H	6.04710600	10.43289300	-2.16738100
B	11.82764900	-1.22863500	-0.62611200	B	11.84623800	-1.03878000	-0.44743100
B	7.74594300	8.20132400	-1.37586100	B	7.62327000	8.34903400	-1.18179300
C	11.18994100	0.18921100	-0.80484800	C	11.18782400	0.36282900	-0.61682800
C	11.98546000	1.34669500	-0.80818500	C	11.95717900	1.54049500	-0.58225400
C	9.80554300	0.38495600	-0.94121500	C	9.80193300	0.54015700	-0.78682300
C	11.43735500	2.61400900	-0.91500900	C	11.38847400	2.79649600	-0.68367000
H	13.06176300	1.25047800	-0.71506300	H	13.03254900	1.46589600	-0.46085200
C	9.25008200	1.64896600	-1.05106400	C	9.22578000	1.79284400	-0.89289300
H	9.14599600	-0.47567600	-0.96233800	H	9.15868600	-0.33152200	-0.84162200
C	10.05395900	2.79480700	-1.02989700	C	10.00204100	2.96146600	-0.83328100
H	12.09660600	3.47434400	-0.92942900	H	12.03541500	3.66557200	-0.66785500
H	8.17302300	1.74782100	-1.12407000	H	8.14930600	1.86639800	-0.99513600
C	9.46518000	4.14769300	-1.11699700	C	9.39501900	4.29787700	-0.91700800
C	8.23735800	4.37054200	-1.75166200	C	8.12425900	4.50217100	-1.47951200
C	10.11866400	5.25261000	-0.55885300	C	10.06385600	5.43395700	-0.43266500
C	7.68061400	5.63907700	-1.80376400	C	7.55785000	5.76234100	-1.54138000
H	7.71744300	3.54470200	-2.22381800	H	7.58237100	3.66447300	-1.90408300
C	9.58340600	6.52562200	-0.66361100	C	9.50583200	6.69366600	-0.52977500
H	11.05155000	5.11385600	-0.02487500	H	11.02806100	5.32633800	0.05073800
C	8.34372100	6.75779800	-1.27803800	C	8.22588500	6.91439600	-1.07857900
H	6.71909800	5.77053700	-2.28968700	H	6.57848900	5.86919600	-1.99722300
H	10.12994300	7.36038400	-0.23723200	H	10.06456600	7.54033400	-0.14462900
C	-12.23055700	-5.47636200	0.98224700	C	-12.06502900	-5.68030100	1.20878300
C	-12.99484500	-4.88521200	2.00164100	C	-12.79268500	-5.10208400	2.26184400
C	-12.66825400	-6.71561400	0.48681900	C	-12.51329900	-6.91920800	0.72204100
C	-14.12813900	-5.50400000	2.51151400	C	-13.90019500	-5.73295100	2.81198700
H	-12.68419600	-3.92922600	2.41007500	H	-12.47308500	-4.14656200	2.66444400
C	-13.81634300	-7.32715700	0.97166200	C	-13.63668300	-7.54280600	1.24777900
H	-12.10413500	-7.19943600	-0.30368400	H	-11.97766000	-7.39332700	-0.09375000
C	-14.54474700	-6.72439900	1.99131300	C	-14.32828900	-6.95286100	2.30002200
H	-14.69091700	-5.03316500	3.31031700	H	-14.43414800	-5.27186200	3.63588800
H	-14.14127800	-8.27654900	0.55986000	H	-13.97081800	-8.49171100	0.84225600
H	-15.43609500	-7.20518400	2.38005900	H	-15.19984700	-7.44314900	2.72047900
C	-11.60934700	6.29790300	-0.54462300	C	-11.68226700	6.08377900	-0.33720000
C	-12.88082000	5.87457200	-0.12406900	C	-12.92794800	5.63576000	0.13385100
C	-11.53942600	7.49912800	-1.26917800	C	-11.67064100	7.27645200	-1.08002300
C	-14.02163200	6.61642100	-0.39916700	C	-14.09637600	6.34456100	-0.11027200
H	-12.97218800	4.95345500	0.44185400	H	-12.97604400	4.72088000	0.71501500
C	-12.67874800	8.23187200	-1.57305500	C	-12.83808800	7.97671800	-1.35145200
H	-10.57353500	7.85574200	-1.61126400	H	-10.72791000	7.65162600	-1.46458500
C	-13.92256200	7.79421700	-1.13154000	C	-14.05435700	7.51464000	-0.86039800
H	-14.98929300	6.27467300	-0.04789700	H	-15.04178600	5.98289600	0.27959300
H	-12.59792300	9.14769500	-2.14860600	H	-12.80116200	8.88566000	-1.94235700
H	-14.81346800	8.37028300	-1.35794600	H	-14.96724900	8.06468700	-1.06248600
C	-1.80650600	11.73446500	2.03270800	C	-2.08710700	12.02431700	1.50236900
C	-2.77506000	12.67276100	1.67385600	C	-3.14958900	12.83217400	1.08786300
C	-0.94419600	12.01114500	3.09434100	C	-1.16351100	12.49931700	2.43738300
C	-2.88256400	13.86556500	2.37511600	C	-3.28347900	14.10700200	1.61283000
H	-3.44107300	12.46031300	0.84566300	H	-3.84732300	12.46309500	0.34602700
C	-1.04689000	13.21364400	3.77961500	C	-1.31019600	13.77584800	2.95508200
H	-0.19517600	11.28033900	3.37641800	H	-0.35221200	11.85943900	2.76226800
C	-2.01784200	14.14419800	3.42743500	C	-2.36727300	14.58264500	2.54601700
H	-3.63944700	14.58654900	2.08681400	H	-4.10077900	14.73699200	1.28222200
H	-0.37084300	13.41720800	4.60263200	H	-0.59981200	14.13869500	3.68835800
H	-2.09997500	15.07959900	3.96897700	H	-2.47630800	15.58113800	2.95256400
C	8.70028700	9.40015500	-1.69476800	C	8.59706300	9.56231700	-1.41468100
C	8.47020500	10.68754900	-1.18222700	C	8.38716800	10.82074600	-0.82187300
C	9.83387500	9.22473500	-2.50567100	C	9.74111500	9.43557800	-2.22375300
C	9.32911300	11.74304900	-1.45720300	C	9.25955900	11.88260900	-1.01994700
H	7.61078600	10.85782900	-0.54257200	H	7.52614400	10.96165700	-0.17616600
C	10.68152300	10.28141100	-2.80864500	C	10.61025100	10.49607000	-2.44153100
H	10.04444000	8.24243700	-2.91536000	H	9.94367900	8.48198700	-2.70077700
C	10.43279900	11.54280600	-2.27891400	C	10.37456700	11.72637400	-1.83682700
H	9.13713200	12.72349600	-1.03480600	H	9.07180200	12.83515900	-0.53470500
H	11.54035100	10.12164200	-3.45158600	H	11.47548600	10.36345900	-3.08312100
H	11.09970400	12.36819900	-2.50423300	H	11.05541200	12.55543500	-1.99861100
C	13.29273300	-1.48982900	-1.11481700	C	13.34640000	-1.25534500	-0.85282200

C	14.16436900	-2.32456600	-0.39692800	C	14.19482500	-2.08527200	-0.10222400
C	13.78937800	-0.89986500	-2.28835600	C	13.89723300	-0.63329400	-1.98486800
C	15.46891600	-2.54649700	-0.81739900	C	15.52604700	-2.27164900	-0.45049900
H	13.81462800	-2.79638200	0.51553000	H	13.80383100	-2.58340900	0.77910100
C	15.08267100	-1.14146000	-2.73196500	C	15.21955000	-0.83736000	-2.35666300
H	13.14310000	-0.25055800	-2.86991000	H	13.27042100	0.01220000	-2.59147500
C	15.92808700	-1.96027700	-1.99158400	C	16.03979700	-1.65230700	-1.58443000
H	16.12648300	-3.18191800	-0.23392900	H	16.16261500	-2.90500400	0.15823700
H	15.43497000	-0.68624500	-3.65132400	H	15.61334700	-0.35611700	-3.24556300
H	16.94304400	-2.14118200	-2.32905100	H	17.07635600	-1.80482500	-1.86581400
C	-2.41426300	-10.42671600	-4.07154100	C	-2.35919200	-10.48048900	-4.21136600
C	-1.26996700	-10.25411100	-4.86681400	C	-1.23630000	-10.27588200	-5.02922900
C	-3.47064600	-11.17250200	-4.61925100	C	-3.41851800	-11.22862900	-4.75009700
C	-1.18597400	-10.79074700	-6.14478400	C	-1.17559000	-10.78432700	-6.32000100
H	-0.43835700	-9.67322500	-4.48186700	H	-0.40334500	-9.69216400	-4.65154800
C	-3.38173600	-11.73887100	-5.88367600	C	-3.35220100	-11.76698400	-6.02807700
H	-4.37395000	-11.31835400	-4.03581600	H	-4.30585700	-11.39877800	-4.14897400
C	-2.23947100	-11.54302200	-6.65199700	C	-2.23102000	-11.53969300	-6.81850100
H	-0.29706200	-10.62691400	-6.74442200	H	-0.30323800	-10.59600100	-6.93652300
H	-4.20602900	-12.32533000	-6.27500900	H	-4.17787100	-12.35607200	-6.41251900
H	-2.17173300	-11.97293700	-7.64562900	H	-2.18125800	-11.94762900	-7.82240600
C	6.23513300	-9.57845800	4.38821600	C	6.42211000	-9.73852300	4.11962000
C	5.66244000	-10.84302700	4.60466200	C	5.88563900	-11.02654300	4.28405600
C	7.09323600	-9.08039900	5.38294300	C	7.26985200	-9.25921900	5.13222400
C	5.94144300	-11.57992000	5.74703700	C	6.19030300	-11.80346500	5.39291100
H	4.99679900	-11.25746100	3.85495300	H	5.22790800	-11.42687100	3.51979600
C	7.36058500	-9.80201300	6.53782300	C	7.56225300	-10.02161000	6.25446400
H	7.54549400	-8.10319700	5.25154300	H	7.69341600	-8.26470100	5.04169100
C	6.78847800	-11.05677600	6.71816300	C	7.02672600	-11.29867900	6.38275700
H	5.49691800	-12.55965400	5.88441900	H	5.77391800	-12.80027700	5.48982900
H	8.01660200	-9.38992300	7.29689200	H	8.20947700	-9.62363900	7.02848500
H	7.00214200	-11.62658000	7.61622500	H	7.26028000	-11.90000600	7.25495800
N	-1.69816400	10.51033800	1.32474400	N	-1.93589800	10.72386900	0.96489300

Structure S47. The coordinates of both the optimized structures of B5N2L$_2$ at S$_0$ and S$_1$ in toluene.

S_0-optimized structure				S_1-optimized structure			
B	-11.30559200	-3.95240000	0.50277000	B	-11.47591200	-3.40472300	0.72697600
C	-10.18282600	-4.91105400	-0.01365400	C	-10.43716500	-4.40181100	0.11785800
C	-9.33331400	-4.53833500	-1.06757300	C	-9.60721100	-4.02837100	-0.95148600
C	-9.95487200	-6.17041600	0.56440700	C	-10.26599600	-5.70082700	0.62367800
C	-8.31813500	-5.36636200	-1.51516200	C	-8.66192100	-4.89130600	-1.47864200
H	-9.47132100	-3.57473500	-1.54605500	H	-9.70437500	-3.03584200	-1.37800200
C	-8.92409900	-6.99396200	0.13947800	C	-9.30552200	-6.56214300	0.11715200
H	-10.59173400	-6.50472200	1.37660800	H	-10.89055000	-6.03740900	1.44442600
C	-8.08398700	-6.60583900	-0.90962800	C	-8.48120200	-6.17174100	-0.94384700
H	-7.66990200	-5.03122700	-2.31719900	H	-8.02462600	-4.55468400	-2.28870600
H	-8.78065100	-7.95969300	0.61185900	H	-9.20572300	-7.55777400	0.53552700
C	-6.95800800	-7.45093400	-1.35331800	C	-7.42253800	-7.05579000	-1.46947600
C	-6.56623700	-7.47930500	-2.69547800	C	-7.06510800	-7.02504300	-2.82122400
C	-6.22460200	-8.21511500	-0.43857300	C	-6.71525600	-7.91713700	-0.62307200
C	-5.46693800	-8.21984100	-3.09809100	C	-6.02035500	-7.80049100	-3.29689200
H	-7.13838300	-6.92760500	-3.43319900	H	-7.62068800	-6.39684400	-3.50874100
C	-5.11047100	-8.93168700	-0.84485400	C	-5.65570600	-8.67071300	-1.10180900
H	-6.50231600	-8.20738100	0.60976000	H	-6.96630800	-7.95824000	0.43116000
C	-4.68641600	-8.94441100	-2.18333100	C	-5.26212500	-8.62307300	-2.44875300
H	-5.19088200	-8.21968200	-4.14709400	H	-5.76871100	-7.75087000	-4.35090100
H	-4.54867000	-9.48931100	-0.10279200	H	-5.11149000	-9.30530700	-0.41031000
C	-11.07971100	-2.40527800	0.43957300	C	-11.16337700	-1.87081500	0.71417300
C	-9.80338100	-1.84090800	0.59682900	C	-9.85148300	-1.38350100	0.83132200
C	-12.13750300	-1.51358600	0.20044400	C	-12.17858600	-0.91426300	0.55511700
C	-9.59402100	-0.47362900	0.52390100	C	-9.56825300	-0.02820100	0.79098900
H	-8.95477700	-2.49048800	0.78286600	H	-9.03368100	-2.08496200	0.95701200
C	-11.93158400	-0.14708700	0.09757000	C	-11.90035300	0.44144200	0.48537900
H	-13.14314500	-1.90244800	0.08014800	H	-13.20905600	-1.24274900	0.47035800
C	-10.65410600	0.40007400	0.25970800	C	-10.58753700	0.91110300	0.60162900
H	-8.58999700	-0.07950500	0.63638300	H	-8.53942200	0.30592800	0.86875500
H	-12.77575200	0.50904500	-0.08417700	H	-12.71365700	1.14855200	0.36432000
C	-10.42935700	1.85519900	0.14721200	C	-10.28456300	2.35352500	0.51410500
C	-9.47883600	2.50420700	0.94274800	C	-9.25077900	2.92436100	1.26500000
C	-11.15991100	2.62525800	-0.76435600	C	-11.02076200	3.18991300	-0.33222800
C	-9.26367800	3.86781200	0.82118900	C	-8.96088600	4.27548000	1.16208500
H	-8.91884600	1.93736800	1.67855100	H	-8.68308200	2.30554800	1.95115200
C	-10.95895200	3.99245300	-0.85947100	C	-10.74434100	4.54480300	-0.40705500
H	-11.87866900	2.14265600	-1.41745100	H	-11.80441000	2.76857500	-0.95200000
C	-10.00279200	4.65673700	-0.07458800	C	-9.70353500	5.13051400	0.33185900
H	-8.52051400	4.33974800	1.45529500	H	-8.15367900	4.68511600	1.76035900
H	-11.54497600	4.55946400	-1.57496500	H	-11.33692500	5.16408300	-1.07190200
B	-9.78766900	6.20279300	-0.17728900	B	-9.40250400	6.66091600	0.23939600
B	-3.36951000	-9.64382500	-2.65928000	B	-3.99462200	-9.36089200	-2.99851200
C	-2.10417600	-9.65633600	-1.74017500	C	-2.71450000	-9.49544200	-2.11307900
C	-1.07192100	-10.59433600	-1.91328500	C	-1.73231000	-10.46800200	-2.37044300
C	-1.92581400	-8.70623500	-0.72023200	C	-2.47100500	-8.62846100	-1.03365700
C	0.07351100	-10.57463300	-1.13379900	C	-0.57605000	-10.56027900	-1.61261600
H	-1.17427100	-11.36161400	-2.67290300	H	-1.88492100	-11.17502500	-3.17861600
C	-0.78981800	-8.68695500	0.07288400	C	-1.32429100	-8.72194100	-0.26249700
H	-2.68355500	-7.94697000	-0.56269900	H	-3.18550300	-7.84419500	-0.81033800
C	0.23465700	-9.61539700	-0.12961200	C	-0.35126300	-9.68477700	-0.54560700
H	0.84814100	-11.31815000	-1.28894800	H	0.15679100	-11.32938000	-1.83245400
H	-0.67221700	-7.92204300	0.83312100	H	-1.15610400	-8.01897300	0.54643400
C	1.47163400	-9.55688200	0.67634400	C	0.89320400	-9.74744700	0.24704600
C	2.72062800	-9.55746900	0.04919000	C	2.13719800	-9.77640500	-0.38913400
C	1.42537200	-9.46775900	2.07010700	C	0.86049400	-9.74613200	1.64427200
C	3.88166400	-9.42266900	0.79324200	C	3.30818500	-9.75303100	0.35236500
H	2.77288900	-9.61284300	-1.03299100	H	2.18156300	-9.76647700	-1.47324800
C	2.59597400	-9.40403700	2.81243300	C	2.03707500	-9.79063300	2.37801400
H	0.46408200	-9.47751800	2.57305600	H	-0.09712400	-9.73803600	2.15466500
C	3.85401400	-9.35647800	2.19435900	C	3.29367600	-9.76891100	1.75524700
H	4.83475500	-9.36967500	0.27710100	H	4.26031800	-9.72160200	-0.16745400
H	2.53209500	-9.36744600	3.89527700	H	1.98113400	-9.82079600	3.46162000
C	-8.37478900	6.82399600	0.06411600	C	-7.94135600	7.19773900	0.40043100
C	-7.19311100	6.15922300	-0.30284600	C	-6.82522400	6.47180100	-0.04609700
C	-8.22718500	8.08954000	0.65472300	C	-7.68741000	8.45101500	0.98017500
C	-5.94393100	6.72980200	-0.11455900	C	-5.53762600	6.97676600	0.05105700
H	-7.25684700	5.17562000	-0.75670200	H	-6.97217500	5.49438100	-0.49352600
C	-6.98143500	8.65161700	0.87620800	C	-6.40218300	8.94913400	1.10897700
H	-9.11198900	8.64356000	0.94996300	H	-8.51912300	9.04917500	1.33655100
C	-5.81258600	7.98735600	0.48574500	C	-5.30174500	8.22696500	0.63312500
H	-5.05434100	6.18297300	-0.40858700	H	-4.70152700	6.38489200	-0.30591000
H	-6.91095800	9.63699300	1.32385500	H	-6.25070800	9.93030100	1.54550300
C	-4.49292900	8.61019000	0.69599500	C	-3.94433900	8.79651800	0.72488600
C	-3.45844100	8.47355000	-0.23513400	C	-3.00075900	8.60194300	-0.29008500
C	-4.23671700	9.38784200	1.82995700	C	-3.57207600	9.59311800	1.81392700
C	-2.23895000	9.10816300	-0.06473200	C	-1.75622800	9.20799600	-0.24503900
H	-3.62597200	7.89977000	-1.14018800	H	-3.26473800	8.01224100	-1.16066300
C	-3.01590700	10.00748100	2.02233100	C	-2.32804800	10.19231700	1.87627600
H	-4.99876400	9.48526400	2.59531000	H	-4.26258100	9.73026100	2.63802200
C	-1.99907300	9.89482000	1.06712200	C	-1.41058400	10.02130900	0.83688600
H	-1.47400600	9.01384400	-0.82604600	H	-1.06335400	9.08301900	-1.06887300
H	-2.84379200	10.58704900	2.92096000	H	-2.06152700	10.80080700	2.73194300
B	5.18436000	-9.19755800	3.00526800	B	4.63325800	-9.72967400	2.57377400
C	6.22901100	-8.15225300	2.49202800	C	5.72063800	-8.69881100	2.16507000
C	7.61511400	-8.36973600	2.51543400	C	7.10161000	-8.91177900	2.33867000
C	5.78536900	-6.92124900	1.98445800	C	5.34640300	-7.45819400	1.61453600
C	8.50757800	-7.42107700	2.03786700	C	8.04030300	-7.95932800	1.98377500
H	8.00106400	-9.30727000	2.90254100	H	7.44412300	-9.85470700	2.75308100
C	6.67313200	-5.94620100	1.56148200	C	6.27487700	-6.48344400	1.30317000
H	4.72025400	-6.72228400	1.92992900	H	4.29475100	-7.25343700	1.44268900
C	8.05021500	-6.18599700	1.56854500	C	7.64920600	-6.70971600	1.47620800
H	9.57352400	-7.62222000	2.05846300	H	9.09453200	-8.16266600	2.13904400
H	6.29880700	-5.00170800	1.18140900	H	5.94134200	-5.54117400	0.88225900

C	8.98273200	-5.15283500	1.07490900	C	8.62738100	-5.67224700	1.13898700
C	8.82298100	-3.81488100	1.44902300	C	8.33394400	-4.31262100	1.33279400
C	10.02683700	-5.47729600	0.20443200	C	9.89000900	-5.98518700	0.61130700
C	9.66363600	-2.83509900	0.94736000	C	9.24655100	-3.32879500	1.00801900
H	8.04049000	-3.54831100	2.15141700	H	7.38905300	-4.03341900	1.78800900
C	10.86411600	-4.49010300	-0.29187800	C	10.79076400	-4.99228000	0.27555300
H	10.15810900	-6.50705600	-0.11069200	H	10.14554400	-7.02309600	0.42234000
C	10.70175000	-3.13724100	0.05042900	C	10.51146800	-3.61688300	0.44698200
H	9.51932500	-1.80904700	1.26766000	H	8.98889300	-2.29730900	1.22218400
H	11.64994900	-4.77058100	-0.98487900	H	11.74149500	-5.28190000	-0.15970300
C	0.43772000	10.06804900	0.77523400	C	1.00847500	10.14846900	0.45396100
C	1.39528300	10.93162000	0.23650700	C	2.03653400	10.95664500	-0.06649600
C	0.74718300	8.70786300	0.86402100	C	1.24225100	8.76414100	0.55730100
C	2.62959600	10.45400700	-0.16571500	C	3.23723300	10.40710500	-0.44658300
H	1.17583600	11.98939700	0.15562200	H	1.87878300	12.02229300	-0.16921400
C	1.97902100	8.23704500	0.43739600	C	2.45614400	8.22919900	0.18770100
H	0.02005900	8.01691300	1.27408900	H	0.47016500	8.11718000	0.95392700
C	2.95711800	9.09702000	-0.07353400	C	3.49811000	9.02868000	-0.32205300
H	3.36360000	11.15913700	-0.53925200	H	4.00760000	11.07018900	-0.81999300
H	2.18282200	7.17407600	0.50643900	H	2.59564300	7.15923700	0.28496200
C	4.29794100	8.61983200	-0.46286700	C	4.79756700	8.48356100	-0.67199100
C	4.93122500	7.57771700	0.22196900	C	5.29112500	7.29711400	-0.08847900
C	5.01131900	9.22962600	-1.50141900	C	5.64691500	9.14441900	-1.58690100
C	6.22729900	7.19165400	-0.07973700	C	6.55405500	6.82689100	-0.35324500
H	4.42320200	7.09217300	1.04805100	H	4.69227800	6.76873200	0.64296600
C	6.30154000	8.84488300	-1.81898000	C	6.90796700	8.68585800	-1.86734600
H	4.53948200	10.00851000	-2.09012600	H	5.28798000	10.01612000	-2.11975700
C	6.94111800	7.83475500	-1.09430300	C	7.40973600	7.52738700	-1.23240400
H	6.70426100	6.40852300	0.49769600	H	6.91978500	5.94557600	0.15698900
H	6.82624100	9.33312700	-2.63163800	H	7.51644900	9.19854000	-2.60075700
B	11.61575900	-2.02733700	-0.56812900	B	11.53982700	-2.51128000	0.06381200
C	11.07920100	-0.57020100	-0.75173800	C	11.09454400	-1.05662100	-0.24316600
C	11.94705300	0.53314400	-0.70200300	C	11.97288400	0.04258000	-0.07452900
C	9.71769700	-0.28174100	-0.94477900	C	9.79729100	-0.71080300	-0.69830300
C	11.48755900	1.83468700	-0.81172300	C	11.58845000	1.34754600	-0.28742600
H	13.00990700	0.36561900	-0.56497500	H	12.98857100	-0.14839800	0.25535200
C	9.25078900	1.01715900	-1.05911200	C	9.40551000	0.59045000	-0.92560500
H	9.00513800	-1.09707600	-1.00716700	H	9.08133000	-1.50266700	-0.88953300
C	10.12498900	2.10864300	-0.98357700	C	10.28197300	1.67557100	-0.71080100
H	12.20179200	2.64976000	-0.78580600	H	12.32320700	2.13197900	-0.14410700
H	8.18662600	1.18693100	-1.17836800	H	8.38707900	0.77369800	-1.25018200
C	9.63354800	3.49726100	-1.07512800	C	9.86476500	3.05641200	-0.90417600
C	8.45844500	3.81851500	-1.76514200	C	8.71862100	3.39810900	-1.65678900
C	10.32929100	4.54988500	-0.46874200	C	10.59025500	4.13131400	-0.34110000
C	8.00543600	5.12378500	-1.85791900	C	8.32336800	4.70721900	-1.83348600
H	7.90433400	3.03986000	-2.27662100	H	8.14749600	2.62016100	-2.14742600
C	9.88529800	5.85694700	-0.55538100	C	10.21243800	5.44225700	-0.52222300
H	11.21848200	4.34240400	0.11501200	H	11.45174300	3.92817600	0.28162300
C	8.71821300	6.16457800	-1.25839600	C	9.06736000	5.74990300	-1.26708300
H	7.10425900	5.34759500	-2.41694200	H	7.45518100	4.93613600	-2.44157000
H	10.43807000	6.65038400	-0.06643000	H	10.78413300	6.24113100	-0.06404100
C	-12.64046100	-4.53259300	1.07480100	C	-12.81644500	-3.93038000	1.33724400
C	-13.34175000	-3.87801700	2.10071000	C	-13.42811300	-3.28496900	2.42446200
C	-13.18747000	-5.72961800	0.58422600	C	-13.45920800	-5.06691200	0.81965800
C	-14.51928000	-4.39713400	2.62137800	C	-14.61072500	-3.75690000	2.97749800
H	-12.94640000	-2.95222200	2.50543900	H	-12.95783700	-2.40517600	2.85119100
C	-14.37912700	-6.24053100	1.08029300	C	-14.65676000	-5.52836200	1.34894500
H	-12.67428300	-6.25983500	-0.21120800	H	-13.01694900	-5.58801000	-0.02298000
C	-15.04369500	-5.57740000	2.10607600	C	-15.23102200	-4.87695400	2.43492800
H	-15.03233700	-3.87981900	3.42490300	H	-15.05340800	-3.24918500	3.82766700
H	-14.78811300	-7.15852800	0.67223200	H	-15.14044600	-6.39913800	0.91966000
H	-15.96940300	-5.97991500	2.50334500	H	-16.16096600	-5.24172900	2.85791000
C	-10.99926800	7.13505100	-0.51689800	C	-10.57100400	7.67014900	-0.01949800
C	-12.30087100	6.83340000	-0.08398800	C	-11.85990400	7.44320000	0.49063000
C	-10.82862000	8.30851400	-1.26958600	C	-10.37482900	8.84051100	-0.77103000
C	-13.37414400	7.66516700	-0.37350300	C	-12.89505200	8.34328800	0.27661000
H	-12.46974100	5.93655000	0.50279200	H	-12.04783400	6.55082300	1.07836900
C	-11.90149400	9.12999800	-1.58836700	C	-11.41215600	9.73032600	-1.01467400
H	-9.83692100	8.57206400	-1.62207700	H	-9.39277600	9.04713700	-1.18347200
C	-13.17661100	8.81252300	-1.13373000	C	-12.67397300	9.48610700	-0.48392900
H	-14.36632500	7.41696500	-0.01217200	H	-13.87645800	8.15184700	0.69691400
H	-11.74375400	10.02156200	-2.18567400	H	-11.23684700	10.61779100	-1.61318000
H	-14.01518100	9.45831500	-1.37156400	H	-13.48376200	10.18535300	-0.66282600
C	-0.79209100	11.83875300	1.89532800	C	-0.20043500	12.06659000	1.31896500
C	-1.69585400	12.83074200	1.51113200	C	-1.11033000	12.97228800	0.77486300
C	0.11345300	12.09996700	2.92475900	C	0.68133200	12.47976800	2.31760400
C	-1.69770200	14.05949400	2.15615800	C	-1.13381200	14.28289900	1.22932500
H	-2.39477700	12.63199600	0.70704100	H	-1.79169800	12.64525900	-0.00164400
C	0.11659500	13.33732300	3.55314400	C	0.65717800	13.79518000	2.75836500
H	0.81296500	11.32906000	3.22639700	H	1.37589200	11.76687300	2.74664800
C	-0.79039300	14.32128500	3.17646900	C	-0.25008600	14.69984500	2.21857000
H	-2.40497400	14.82147800	1.84783500	H	-1.84140500	14.98351200	0.80104300
H	0.82537900	13.52713300	4.35158600	H	1.34204600	14.10964200	3.53747500
H	-0.78991000	15.28441700	3.67380600	H	-0.27046000	15.72497600	2.56956400
C	9.22424300	8.51285300	-1.63703400	C	9.70445100	8.00630600	-1.86549000
C	9.19477700	9.72416700	-0.94107500	C	9.84042600	9.24383400	-1.23061200
C	10.21143500	8.30965200	-2.60493000	C	10.59298900	7.63816700	-2.87829100
C	10.12889800	10.71180800	-1.21900900	C	10.84837600	10.11015000	-1.62396500
H	8.43890100	9.88682400	-0.18205600	H	9.17182400	9.50953100	-0.42052300
C	11.15177900	9.29641300	-2.86279800	C	11.59690300	8.51276900	-3.26145900
H	10.23538300	7.37534300	-3.15343600	H	10.48331600	6.67536000	-3.36198800
C	11.11494100	10.50489700	-2.17671100	C	11.72782400	9.75041400	-2.63953400
H	10.09213600	11.64580400	-0.66901300	H	10.95719100	11.06446300	-1.12197000
H	11.91062400	9.12190900	-3.61766200	H	12.27809800	8.22678300	-4.05416300
H	11.84649600	11.27682800	-2.38586600	H	12.51751400	10.42877300	-2.94021800
C	13.07933200	-2.37785300	-1.00385200	C	13.07423500	-2.87326000	0.00614100
C	13.86826400	-3.27415800	-0.26489900	C	13.67428500	-3.72789500	0.94820600

C	13.65694400	-1.80845300	-2.15015000	C	13.91944400	-2.36038300	-0.99432800
C	15.17110300	-3.57471100	-0.63933500	C	15.02612100	-4.04358600	0.90334800
H	13.45518400	-3.73195600	0.62801100	H	13.06277000	-4.14519500	1.74221600
C	14.94807300	-2.12785300	-2.54862100	C	15.26940100	-2.68039800	-1.05698100
H	13.07580500	-1.11240500	-2.74619000	H	13.49925300	-1.70144100	-1.74805700
C	15.71092300	-3.00700000	-1.78804900	C	15.83194300	-3.52310700	-0.10388700
H	15.76444100	-4.25687400	-0.03998500	H	15.45367600	-4.69810700	1.65647200
H	15.36327900	-1.68638000	-3.44823100	H	15.88569200	-2.27303800	-1.85242500
H	16.72436700	-3.24915300	-2.08981700	H	16.88707600	-3.77242000	-0.14617000
C	-3.31939600	-10.27389600	-4.09247600	C	-4.01129100	-9.90349700	-4.46821500
C	-2.17530800	-10.19313700	-4.90255200	C	-2.88393600	-9.83023300	-5.30209000
C	-4.43754000	-10.93655900	-4.62431300	C	-5.17396200	-10.47595700	-5.00914200
C	-2.14942500	-10.73847100	-6.17934400	C	-2.91574900	-10.29754600	-6.60939000
H	-1.29635000	-9.67720700	-4.53049600	H	-1.97124400	-9.38260300	-4.92296600
C	-4.40894500	-11.51176300	-5.88755400	C	-5.20442400	-10.97373800	-6.30482500
H	-5.34205300	-11.00964200	-4.02921300	H	-6.06687000	-10.54028000	-4.39569400
C	-3.26460700	-11.40785000	-6.67056200	C	-4.07481300	-10.87957800	-7.11019600
H	-1.25809600	-10.64633300	-6.79057800	H	-2.03507900	-10.21320600	-7.23700300
H	-5.28137700	-12.03311100	-6.26649700	H	-6.11094000	-11.42729900	-6.69099300
H	-3.24324000	-11.84463700	-7.66327700	H	-4.09877300	-11.25612400	-8.12724600
C	5.44148400	-10.03882500	4.29325000	C	4.82591300	-10.69651000	3.78887200
C	4.78922800	-11.26572900	4.50112900	C	4.15909300	-11.93201400	3.84542500
C	6.32763600	-9.60235500	5.29231600	C	5.65364400	-10.36609400	4.87509900
C	5.01904200	-12.02597400	5.63915500	C	4.32373100	-12.80068900	4.91547700
H	4.09966900	-11.63242700	3.74816800	H	3.50759300	-12.21792900	3.02613100
C	6.54658400	-10.34660000	6.44306400	C	5.80765900	-11.21985000	5.95889100
H	6.84091800	-8.65483700	5.16788300	H	6.17209900	-9.41324200	4.87381600
C	5.89575200	-11.56362500	6.61476300	C	5.14715200	-12.44358100	5.97787500
H	4.51290200	-12.97627300	5.76991400	H	3.80677200	-13.75440000	4.92554200
H	7.22584500	-9.98141600	7.20569900	H	6.44383500	-10.93301400	6.78947600
H	6.07114000	-12.15122400	7.50963400	H	5.27135300	-13.11609600	6.82009200
N	-0.78766800	10.58019100	1.24389400	N	-0.18340000	10.71829100	0.86799800
N	8.27943200	7.49867900	-1.36805300	N	8.69510000	7.09972600	-1.46097400

Structure S48. The coordinates of both the optimized structures of B4N3L$_2$ at S$_0$ and S$_1$ in toluene.

S_0-optimized structure				S_1-optimized structure			
B	-11.28807200	-4.02438100	0.58803000	B	10.99790000	-4.71307600	-0.54096000
C	-10.16164900	-4.97342700	0.06188500	C	9.81350300	-5.57464600	0.02620800
C	-9.32749800	-4.59406000	-1.00187800	C	9.01575700	-5.11801700	1.08968800
C	-9.91476100	-6.22949200	0.63936800	C	9.48751300	-6.84052000	-0.49146900
C	-8.30905000	-5.41258000	-1.45923800	C	7.95898300	-5.85925400	1.58899200
H	-9.48025200	-3.63268800	-1.48032700	H	9.23250300	-4.15254700	1.53424400
C	-8.88057800	-7.04348500	0.20430600	C	8.41726300	-7.57999100	-0.01386200
H	-10.53920900	-6.56873400	1.45913900	H	10.08429300	-7.24875000	-1.30085700
C	-8.05596700	-6.64876400	-0.85461200	C	7.62555600	-7.10241800	1.03762600
H	-7.67293800	-5.07202800	-2.26864200	H	7.35637500	-5.45128400	2.39338700
H	-8.72233400	-8.00691900	0.67666200	H	8.20448700	-8.55217600	-0.44630900
C	-6.92644900	-7.48288300	-1.30990800	C	6.45724200	-7.84954100	1.53488700
C	-6.54830700	-7.50684200	-2.65605600	C	6.07137300	-7.77611700	2.87890200
C	-6.17590200	-8.23998400	-0.40320300	C	5.66707300	-8.62168200	0.67281000
C	-5.44527100	-8.23525900	-3.07018600	C	4.92826000	-8.41460900	3.32817900
H	-7.13389000	-6.96116400	-3.38767500	H	6.68368900	-7.22098000	3.58085500
C	-5.05839100	-8.94452100	-0.82119500	C	4.51096000	-9.23557200	1.12392900
H	-6.44298000	-8.23581900	0.64791800	H	5.93774000	-8.69732800	-0.37466800
C	-4.64750900	-8.95155100	-2.16381900	C	4.09079500	-9.13781700	2.46179900
H	-5.18000800	-8.23170400	-4.12195500	H	4.65987800	-8.33065100	4.37598700
H	-4.48340000	-9.49681000	-0.08527600	H	3.91059900	-9.80024700	0.41800000
C	-11.07729700	-2.47542600	0.51695100	C	10.88199500	-3.17406300	-0.58658200
C	-9.80486300	-1.89869700	0.66029500	C	9.63263000	-2.51186600	-0.62792000
C	-12.14513100	-1.59475500	0.28177100	C	12.01130200	-2.32272900	-0.57439900
C	-9.60840900	-0.53011700	0.57704100	C	9.51766600	-1.13900100	-0.64976000
H	-8.94888700	-2.53949500	0.84299200	H	8.72513500	-3.10623400	-0.65315900
C	-11.95230000	-0.22713800	0.16825400	C	11.90551800	-0.94818500	-0.57036100
H	-13.14823300	-1.99334300	0.17250400	H	13.00173100	-2.76556100	-0.54574400
C	-10.67839900	0.33228500	0.31565600	C	10.65185100	-0.30077700	-0.60633400
H	-8.60705300	-0.12633300	0.67859900	H	8.52737500	-0.69768300	-0.66261900
H	-12.80373200	0.42033300	-0.01065100	H	12.81257200	-0.35408300	-0.56599200
C	-10.46678000	1.78827200	0.18998000	C	10.53322800	1.14855300	-0.58925600
C	-9.51667500	2.45146400	0.97417700	C	9.37582600	1.81079600	-1.05410800
C	-11.20864100	2.54459700	-0.72395200	C	11.56687900	1.97510500	-0.09772100
C	-9.31227100	3.81540000	0.83891800	C	9.26366900	3.18290800	-1.02187500
H	-8.94821000	1.89540900	1.71171800	H	8.56842000	1.23121700	-1.48827600
C	-11.01803800	3.91227800	-0.83296200	C	11.44930800	3.34655100	-0.07625800
H	-11.92772300	2.05074200	-1.36827800	H	12.46174600	1.52185900	0.31445200
C	-10.06196000	4.59061000	-0.06006700	C	10.29146000	4.02748900	-0.53110600
H	-8.56892500	4.29849300	1.46433700	H	8.36297300	3.63459600	-1.42517200
H	-11.61218300	4.46836600	-1.55034200	H	12.26679200	3.92715100	0.33843200
B	-9.85703400	6.13691700	-0.17890500	B	10.17894100	5.56223000	-0.50627700
B	-3.32746700	-9.63429300	-2.65502400	B	2.72989300	-9.69868900	2.97915100
C	-2.05148300	-9.63421300	-1.75091200	C	1.46561600	-9.70199500	2.05464600
C	-1.00787400	-10.55567200	-1.94386000	C	0.36778700	-10.54416700	2.30120500
C	-1.87435600	-8.68950800	-0.72567700	C	1.35330800	-8.82939600	0.95893100
C	0.14677500	-10.52492900	-1.17853900	C	-0.77638200	-10.50495900	1.52016300
H	-1.10837700	-11.31860300	-2.70806500	H	0.41573300	-11.25031300	3.12304700
C	-0.72930700	-8.66010300	0.05409700	C	0.21996900	-8.79430000	0.16231700
H	-2.64075800	-7.94228600	-0.55324300	H	2.16330000	-8.14216500	0.74244700
C	0.30611600	-9.57160300	-0.16846200	C	-0.86961100	-9.62402200	0.43881700
H	0.93049700	-11.25539200	-1.34925500	H	-1.60358100	-11.17332700	1.73537100
H	-0.61374300	-7.90000000	0.81945800	H	0.15714200	-8.08938100	-0.65999600
C	1.55240200	-9.50267400	0.62246200	C	-2.10230900	-9.53833000	-0.37130400
C	2.79233200	-9.46979100	-0.02135000	C	-3.34308000	-9.37476800	0.25102900
C	1.52378500	-9.43928400	2.01807600	C	-2.05770200	-9.58297200	-1.76729700
C	3.96111500	-9.32919800	0.70947600	C	-4.49293000	-9.20943600	-0.50404900
H	2.83057500	-9.50450100	-1.10500600	H	-3.39264400	-9.32652600	1.33370900
C	2.70347700	-9.37088200	2.74572000	C	-3.22323600	-9.48809900	-2.51422100
H	0.56973000	-9.47422400	2.53360000	H	-1.10361200	-9.72041500	-2.26525300
C	3.95181300	-9.29150000	2.11177300	C	-4.46869100	-9.27486100	-1.90538500
H	4.90610500	-9.24999300	0.18189800	H	-5.43485900	-9.02796900	0.00351000
H	2.65389600	-9.35695700	3.82989000	H	-3.16318400	-9.55712700	-3.59573300
C	-8.44653900	6.76832600	0.04976000	C	8.78665200	6.28773700	-0.54572400
C	-7.26294500	6.10710100	-0.31746000	C	7.62672800	5.75894500	0.05487200
C	-8.30312100	8.03906700	0.63013500	C	8.63390600	7.54399500	-1.16450400
C	-6.01596700	6.68530200	-0.13817200	C	6.41557600	6.42953500	0.04701500
H	-7.32342200	5.11987000	-0.76382500	H	7.68632100	4.79912600	0.55703300
C	-7.05934800	8.60891200	0.84288300	C	7.42443300	8.21260100	-1.19751600
H	-9.18959600	8.59075000	0.92478500	H	9.49708700	8.00643400	-1.63162400
C	-5.88863400	7.94747900	0.45320900	C	6.28330300	7.67413500	-0.58493300
H	-5.12473800	6.14085000	-0.43162700	H	5.55192900	5.97070400	0.51792800
H	-6.99202100	9.59775600	1.28332200	H	7.37142100	9.18735800	-1.67103300
C	-4.57106200	8.57696100	0.65657800	C	5.01304000	8.41117500	-0.58924200
C	-3.53807500	8.43986100	-0.27617400	C	4.10505200	8.32003300	0.47726100
C	-4.31513900	9.36045900	1.78654000	C	4.67671500	9.26639500	-1.65129200
C	-2.32022100	9.07898000	-0.11087900	C	2.93934800	9.06426400	0.50230300
H	-3.70551300	7.86161300	-1.17841100	H	4.34530800	7.69625700	1.33001600
C	-3.09584000	9.98454400	1.97394100	C	3.51731200	10.01515300	-1.63747500
H	-5.07596000	9.45871800	2.55301600	H	5.32957900	9.32801800	-2.51355200
C	-2.08028400	9.87073700	1.01746200	C	2.63931100	9.92711700	-0.55469100
H	-1.55651600	8.98380400	-0.87332400	H	2.27960600	9.01292100	1.36099500
H	-2.92378700	10.56823800	2.86989200	H	3.27886200	10.66657300	-2.46990500
B	5.29502300	-9.13654100	2.90327100	B	-5.78380800	-9.07468300	-2.73017900
C	6.31148200	-8.05841900	2.40563200	C	-6.73273900	-7.90203400	-2.31549400
C	7.70051400	-8.25725000	2.38131400	C	-8.13255700	-7.99922700	-2.32620400
C	5.83936500	-6.81417200	1.95914400	C	-6.18352100	-6.67352700	-1.91667000
C	8.56706500	-7.27876100	1.91668400	C	-8.93809000	-6.93723800	-1.94229100
H	8.10916200	-9.20368300	2.72090900	H	-8.59954800	-8.93042000	-2.63066900
C	6.70272700	-5.81233400	1.54898900	C	-6.98232200	-5.59200400	-1.58487300
H	4.77066100	-6.62740900	1.94118300	H	-5.10502700	-6.56362200	-1.87405200
C	8.08331300	-6.03174700	1.50721500	C	-8.37559100	-5.70881600	-1.58047100
H	9.63568800	-7.46626100	1.90155700	H	-10.01758700	-7.04586100	-1.95505700
H	6.30539700	-4.86002300	1.21411900	H	-6.52500200	-4.65535100	-1.28417500

C	8.98782900	-4.97039100	1.02684500
C	8.82712800	-3.64597100	1.44379800
C	10.01398800	-5.24101700	0.11846200
C	9.64829300	-2.63521300	0.97596600
H	8.06452300	-3.40692100	2.17723100
C	10.81630400	-4.23142600	-0.38622800
H	10.15574100	-6.25488400	-0.24040200
C	10.64307800	-2.90661800	0.02991200
H	9.51656000	-1.62547000	1.34443500
H	11.57548500	-4.46769700	-1.12155500
C	0.35654900	10.04908400	0.72376000
C	1.31573900	10.91459100	0.19102500
C	0.66688600	8.68889200	0.80954900
C	2.55234100	10.43916600	-0.20667600
H	1.09605600	11.97245500	0.11201500
C	1.90070000	8.22026400	0.38617200
H	-0.06098900	7.99588900	1.21477000
C	2.88106800	9.08219800	-0.11729300
H	3.28721400	11.14643700	-0.57430300
H	2.10430800	7.15711600	0.45253000
C	4.22463000	8.60748800	-0.50008100
C	4.85028700	7.55599900	0.17715100
C	4.94960700	9.22865900	-1.52402900
C	6.14893500	7.17207100	-0.11625200
H	4.33378200	7.05968600	0.99149500
C	6.24284500	8.84740400	-1.83260000
H	4.48462900	10.01442100	-2.10908500
C	6.87562600	7.82836500	-1.11336600
H	6.61815100	6.37978400	0.45488400
H	6.77526200	9.34500000	-2.63447600
C	10.97837800	-0.56863300	-0.64124400
C	11.83673600	0.51974400	-0.45971100
C	9.64863300	-0.30518000	-0.98550100
C	11.37665300	1.81616600	-0.60942700
H	12.87549700	0.34719000	-0.20599000
C	9.19205300	0.99693700	-1.09839000
H	8.96173000	-1.12642900	-1.14891300
C	10.03950800	2.09490500	-0.91329200
H	12.08380000	2.62885500	-0.49003300
H	8.14407600	1.15567600	-1.32571400
C	9.55120200	3.48357300	-1.02549200
C	8.44302500	3.81419900	-1.81489800
C	10.18350000	4.52823700	-0.34040800
C	7.99419900	5.12030100	-1.92552700
H	7.94070900	3.04336400	-2.38813000
C	9.74548300	5.83640000	-0.44855200
H	11.01708600	4.31359700	0.31818000
C	8.64641700	6.15158400	-1.24870700
H	7.14549500	5.35232900	-2.55877900
H	10.24971200	6.62486000	0.09805800
C	-12.61166600	-4.61508600	1.17509400
C	-13.30951800	-3.96292400	2.20487600
C	-13.15211300	-5.81896500	0.69412400
C	-14.47744600	-4.49072100	2.73827600
H	-12.91907600	-3.03186900	2.60224400
C	-14.33447900	-6.33878000	1.20301200
H	-12.64145000	-6.34745800	-0.10407300
C	-14.99571000	-5.67771900	2.23228200
H	-14.98793300	-3.97494600	3.54442300
H	-14.73875000	-7.26205300	0.80222600
H	-15.91403500	-6.08711100	2.63953900
C	-11.07577300	7.05885300	-0.52088900
C	-12.37367600	6.75293600	-0.07993400
C	-10.91543300	8.22710200	-1.28389200
C	-13.45323200	7.57570100	-0.37181200
H	-12.53456200	5.86003100	0.51504200
C	-11.99461300	9.03944300	-1.60480300
H	-9.92684600	8.49373600	-1.64274700
C	-13.26587100	8.71794100	-1.14225000
H	-14.44235500	7.32446200	-0.00424800
H	-11.84484000	9.92701900	-2.21004500
H	-14.10936500	9.35663200	-1.38185300
C	-0.87776300	11.81909200	1.84008100
C	-1.78120000	12.80970300	1.45169200
C	0.02393000	12.08226800	2.87235400
C	-1.78656900	14.03917600	2.09534000
H	-2.47710200	12.60920100	0.64540800
C	0.02361600	13.32030500	3.49943000
H	0.72325300	11.31241900	3.17711900
C	-0.88305300	14.30293500	3.11850800
H	-2.49354600	14.80010800	1.78375200
H	0.72946000	13.51174000	4.30008600
H	-0.88522200	15.26662200	3.61477400
C	9.17079800	8.49251300	-1.63909000
C	9.12208200	9.72570100	-0.98246300
C	10.19823100	8.25472100	-2.55670600
C	10.07420700	10.69855800	-1.25081500
H	8.33753700	9.91721800	-0.26030300
C	11.15519500	9.22778900	-2.80475200
H	10.24049300	7.30345300	-3.07391900
C	11.09852400	10.45750100	-2.15904700
H	10.02061100	11.64888100	-0.73086600
H	11.94433200	9.02462600	-3.52044600
H	11.84374200	11.21833700	-2.36044400
C	12.79105500	-2.17759600	-0.85880200
C	13.64159600	-2.83298600	0.03237600
C	13.26833000	-1.81065700	-2.11759400

C	-9.21587000	-4.56035400	-1.19181900
C	-8.93332100	-3.27363300	-1.65953100
C	-10.30522200	-4.71206900	-0.33034600
C	-9.70112100	-2.18439200	-1.28563300
H	-8.11844100	-3.12845600	-2.36037600
C	-11.05760700	-3.62376900	0.07860200
H	-10.54059900	-5.69418800	0.06513600
C	-10.76314500	-2.33991600	-0.38974100
H	-9.47823800	-1.20573500	-1.69291800
H	-11.87354600	-3.76604000	0.77666300
C	0.25506200	10.29003100	-0.15910100
C	-0.72380900	11.16905700	0.35424400
C	-0.08740900	8.92725200	-0.30348800
C	-1.97694400	10.71242700	0.67023600
H	-0.47973800	12.21191400	0.50574100
C	-1.35230400	8.48962000	0.00507000
H	0.64370200	8.22937000	-0.68970300
C	-2.34914500	9.36259700	0.49180600
H	-2.70081900	11.42792500	1.03838000
H	-1.57437600	7.43765100	-0.12516400
C	-3.70414800	8.92183100	0.75998700
C	-4.24456800	7.76385200	0.16189200
C	-4.56672900	9.66264700	1.59638200
C	-5.55775500	7.39753000	0.33813100
H	-3.63849500	7.17301800	-0.51362700
C	-5.87851900	9.30814400	1.78435600
H	-4.18728400	10.51690100	2.14260200
C	-6.41940100	8.17957000	1.13407500
H	-5.94784100	6.53293700	-0.18318400
H	-6.50153800	9.88966300	2.45108200
C	-10.97961600	0.03034800	0.21249600
C	-11.74886400	1.18264500	0.00710400
C	-9.64881800	0.19552200	0.61897900
C	-11.20372900	2.43899600	0.19120800
H	-12.78384400	1.09032800	-0.29698100
C	-9.10927100	1.45877000	0.77752300
H	-9.03020600	-0.67358300	0.80343300
C	-9.86662200	2.61712800	0.56625400
H	-11.84512200	3.30072300	0.04734700
H	-8.06614200	1.53765800	1.06185500
C	-9.29211700	3.96337300	0.72805600
C	-8.19472900	4.20181300	1.56706800
C	-9.83374400	5.06426700	0.04886300
C	-7.66971900	5.47200600	1.73060400
H	-7.76554700	3.38740300	2.13817400
C	-9.32790100	6.33889200	0.21572700
H	-10.65263800	4.91863700	-0.64493400
C	-8.23884500	6.55544500	1.06035500
H	-6.83893800	5.63480100	2.40747000
H	-9.76458200	7.17295300	-0.32066300
C	12.29370300	-5.44663000	-1.04717300
C	13.03378700	-4.97322800	-2.14398900
C	12.76168000	-6.61858600	-0.42885700
C	14.17056100	-5.62866800	-2.59890500
H	12.69938700	-4.07723700	-2.65690100
C	13.90780900	-7.27048000	-0.86438400
H	12.21754000	-7.01941800	0.42047500
C	14.61575000	-6.77854300	-1.95568800
H	14.71192900	-5.24171400	-3.45615100
H	14.24892100	-8.16578400	-0.35467000
H	15.50739100	-7.28920700	-2.30411800
C	11.47387500	6.45954000	-0.44251800
C	12.64510700	6.12663400	-1.14579800
C	11.51565500	7.64498700	0.31276800
C	13.78314500	6.92169100	-1.10024500
H	12.65494800	5.22705000	-1.75286200
C	12.65322400	8.43868100	0.37788900
H	10.63368300	7.94361700	0.87109700
C	13.79450100	8.08128200	-0.33261200
H	14.66453900	6.63644200	-1.66583800
H	12.65107700	9.33941400	0.98371600
H	14.68374700	8.70147700	-0.28959100
C	1.67730400	12.12635400	-0.86153100
C	2.71961800	12.85110500	-0.28363900
C	0.83814900	12.73018100	-1.79890800
C	2.91006200	14.17816700	-0.63672300
H	3.36814700	12.37286300	0.44044400
C	1.03586100	14.05941100	-2.14177900
H	0.04707000	12.15258500	-2.26252000
C	2.06992300	14.78686100	-1.56318600
H	3.71678500	14.74019500	-0.18105900
H	0.38735900	14.52321200	-2.87590100
H	2.22473700	15.82371900	-1.83754500
C	-8.71087800	8.87966900	1.55236300
C	-8.71311600	10.07668800	0.83480900
C	-9.67007200	8.65574900	2.53980800
C	-9.66247900	11.04729300	1.11877000
H	-7.98013400	10.23462300	0.05231800
C	-10.61937300	9.62983100	2.81027200
H	-9.66286400	7.72365900	3.09217500
C	-10.61774600	10.82827700	2.10502200
H	-9.66365900	11.97307600	0.55510200
H	-11.35923800	9.45347600	3.58235800
H	-11.36083100	11.58709000	2.32014500
C	-12.92817000	-1.42831300	0.24024400
C	-13.71729200	-2.01553800	-0.74854200
C	-13.51181800	-1.03694800	1.44521000

C	14.94742000	-3.12359400	-0.33718600	C	-15.07337200	-2.21295200	-0.52885000
H	13.27247200	-3.11252100	1.01234700	H	-13.26129200	-2.31634200	-1.68465800
C	14.58052000	-2.08930100	-2.47337600	C	-14.87175200	-1.22289200	1.65108000
H	12.60586600	-1.30619700	-2.81141400	H	-12.89437700	-0.58956900	2.21561200
C	15.42472500	-2.75024200	-1.58838600	C	-15.65721100	-1.81440100	0.66825000
H	15.59885800	-3.63324600	0.36407300	H	-15.67796000	-2.67114900	-1.30353100
H	14.93986100	-1.79861200	-3.45432100	H	-15.31577400	-0.91642100	2.59160800
H	16.44721400	-2.97244800	-1.87152100	H	-16.71750500	-1.96564000	0.83509100
C	-3.28629700	-10.26007400	-4.09058200	C	2.62234200	-10.19594100	4.46275000
C	-2.15530500	-10.15772100	-4.91638500	C	1.48815800	-9.95029700	5.25313000
C	-4.40021700	-10.94069000	-4.60831400	C	3.67824700	-10.90379300	5.05934100
C	-2.13799300	-10.69972700	-6.19476400	C	1.41322600	-10.38079400	6.57133800
H	-1.28017100	-9.62761800	-4.55536000	H	0.65665800	-9.39602900	4.83022700
C	-4.37933900	-11.51286700	-5.87309900	C	3.59883200	-11.36420200	6.36688000
H	-5.29505200	-11.03028200	-4.00094400	H	4.57423000	-11.10323000	4.48049100
C	-3.24809500	-11.38752000	-6.67183500	C	2.46630700	-11.09772000	7.12827300
H	-1.25712500	-10.59068700	-6.81827000	H	0.53163900	-10.16145000	7.16427300
H	-5.24787200	-12.04848200	-6.24095800	H	4.42358100	-11.92269500	6.79646300
H	-3.23308500	-11.82184100	-7.66574700	H	2.40611300	-11.44495600	8.15426000
C	5.59153000	-10.02015800	4.15435600	C	-6.11926500	-10.00086200	-3.93998000
C	4.95528800	-11.25950300	4.33612100	C	-5.57890700	-11.29423100	-4.03518300
C	6.49884800	-9.60994800	5.14546600	C	-6.96710400	-9.57859900	-4.97758600
C	5.22008900	-12.05604000	5.44127600	C	-5.87952200	-12.12958900	-5.10186900
H	4.24996400	-11.60658200	3.58848800	H	-4.92102800	-11.65148300	-3.25005500
C	6.75366400	-10.39131300	6.26378500	C	-7.25557400	-10.40009000	-6.05830600
H	7.00037100	-8.65380300	5.04083000	H	-7.39351700	-8.58180300	-4.94046100
C	6.11762600	-11.61947000	6.40980000	C	-6.71588200	-11.68049400	-6.11818300
H	4.72518700	-13.01477500	5.55206300	H	-5.45963200	-13.12863700	-5.14548700
H	7.44904700	-10.04616300	7.02121300	H	-7.90240500	-10.04567600	-6.85357900
H	6.32071000	-12.23579400	7.27915100	H	-6.94587600	-12.32776900	-6.95782100
N	-0.87042400	10.55978400	1.18990800	N	1.49526000	10.76038600	-0.51566700
N	8.21367800	7.49010800	-1.37862500	N	-7.75546800	7.87067400	1.26050700
N	11.45444800	-1.88396700	-0.48919300	N	-11.53821200	-1.23875800	0.02401600

Structure S49. The coordinates of both the optimized structures of B3N4L$_2$ at S$_0$ and S$_1$ in toluene.

S_0-optimized structure				S_1-optimized structure			
B	-10.90655800	-4.68851700	0.54770100	B	-10.46260600	-5.59261600	0.46790400
C	-9.70921000	-5.56453200	0.05240900	C	-9.19839400	-6.34131500	-0.08671600
C	-8.90273900	-5.15675200	-1.02210700	C	-8.44299500	-5.82525400	-1.15393400
C	-9.37090200	-6.77983500	0.66924400	C	-8.75356900	-7.56104000	0.45258400
C	-7.82582800	-5.91180800	-1.45459500	C	-7.31626300	-6.46782300	-1.63736700
H	-9.12538900	-4.22434100	-1.52970400	H	-8.75015000	-4.89217700	-1.61425100
C	-8.27774800	-7.52742600	0.26019300	C	-7.61293000	-8.19906500	-0.00771300
H	-9.97080000	-7.13906000	1.49876600	H	-9.31270800	-8.01292700	1.26562600
C	-7.48327300	-7.10629300	-0.81149000	C	-6.86568200	-7.66277200	-1.06334400
H	-7.21556800	-5.55167700	-2.27543400	H	-6.75213000	-6.01697900	-2.44686200
H	-8.04901000	-8.46122100	0.76234700	H	-7.30806000	-9.13868700	0.44133300
C	-6.29715000	-7.87240500	-1.24186300	C	-5.62791800	-8.30259000	-1.54191200
C	-5.92510500	-7.92488400	-2.58898000	C	-5.24904900	-8.22956300	-2.88798700
C	-5.49071100	-8.53674400	-0.31093400	C	-4.76821100	-8.96946800	-0.65902400
C	-4.77877700	-8.59571800	-2.98265000	C	-4.05219000	-8.77345600	-3.32200900
H	-6.55033700	-7.44969600	-3.33683400	H	-5.91005900	-7.75291500	-3.60341700
C	-4.33084900	-9.18158600	-0.70940600	C	-3.56009900	-9.48607900	-1.09528500
H	-5.75117600	-8.50651200	0.74142000	H	-5.03061400	-9.03797700	0.39105400
C	-3.93077400	-9.22080200	-2.05468300	C	-3.15310100	-9.39329100	-2.43749100
H	-4.52112600	-8.61921900	-4.03610800	H	-3.79270400	-8.69591800	-4.37259400
H	-3.71451300	-9.66176800	0.04343000	H	-2.90840500	-9.96958400	-0.37477500
C	-10.81074700	-3.13043400	0.44082800	C	-10.49727900	-4.04993300	0.50373900
C	-9.58522100	-2.45891600	0.57959800	C	-9.31827200	-3.26880400	0.53800800
C	-11.93946300	-2.33563100	0.18481100	C	-11.70435900	-3.31257600	0.49838500
C	-9.49089900	-1.08082700	0.47696100	C	-9.33811500	-1.89150600	0.56468200
H	-8.68516700	-3.03158900	0.77624800	H	-8.35675500	-3.77148000	0.55648800
C	-11.84840900	-0.95931000	0.05103000	C	-11.73366200	-1.93437800	0.49958400
H	-12.90932900	-2.80973600	0.07706300	H	-12.64698000	-3.84994600	0.47308500
C	-10.62088100	-0.30455900	0.19881100	C	-10.54900200	-1.16760000	0.53372500
H	-8.52345500	-0.60130600	0.57837100	H	-8.39565900	-1.35552600	0.57315400
H	-12.74437300	-0.37948100	-0.14223100	H	-12.69467100	-1.43219400	0.50295500
C	-10.51798500	1.16190500	0.06124300	C	-10.57162800	0.28630800	0.52863200
C	-9.62971600	1.89925100	0.85147200	C	-9.48047700	1.05337000	0.99298200
C	-11.30395900	1.85434100	-0.86625900	C	-11.68384400	1.01356000	0.05169500
C	-9.52717100	3.27378100	0.70954500	C	-9.50094200	2.42996300	0.97185000
H	-9.03050000	1.39169100	1.59947200	H	-8.61829400	0.55089000	1.41777100
C	-11.21565600	3.23197900	-0.98223400	C	-11.69876200	2.39006800	0.04119700
H	-11.97652100	1.30352300	-1.51475300	H	-12.53433600	0.47946800	-0.35771900
C	-10.32249300	3.98495200	-0.20306500	C	-10.60827900	3.17565900	0.49396200
H	-8.82982100	3.81523400	1.34007100	H	-8.64542000	2.96278200	1.37439900
H	-11.84141300	3.73753000	-1.71009400	H	-12.57120500	2.89279600	-0.36310600
B	-10.23116500	5.54165900	-0.32755000	B	-10.64259600	4.71415500	0.47846500
B	-2.57780500	-9.85254600	-2.52395300	B	-1.75142900	-9.86315500	-2.93614500
C	-1.30253100	-9.74381900	-1.62626700	C	-0.49790100	-9.73792800	-2.00605800
C	-0.22447700	-10.63588400	-1.75818100	C	0.64750700	-10.53148300	-2.18801300
C	-1.16240600	-8.72686500	-0.66662600	C	-0.44804500	-8.79422100	-0.96629000
C	0.92483000	-10.51355300	-0.99416300	C	1.77424900	-10.38535800	-1.39454800
H	-0.29467500	-11.45106600	-2.47016600	H	0.64990500	-11.28816500	-2.96522900
C	-0.01876500	-8.59983600	0.10496400	C	0.67235500	-8.64388700	-0.16531000
H	-1.95779500	-8.00046800	-0.54239600	H	-1.29862700	-8.14279600	-0.80001800
C	1.04964500	-9.48737200	-0.05235700	C	1.80777300	-9.43233800	-0.37180500
H	1.73210300	-11.22870800	-1.11223700	H	2.63508100	-11.02635000	-1.55404200
H	0.06775500	-7.78273500	0.81334700	H	0.68571100	-7.88181000	0.60679400
C	2.28822200	-9.32213500	0.73492200	C	3.01914200	-9.23387400	0.44920700
C	3.53651500	-9.35488800	0.10802500	C	4.27763100	-9.14383700	-0.15155500
C	2.24861500	-9.10134200	2.11473100	C	2.94032900	-9.09803100	1.83847300
C	4.70258900	-9.15639700	0.82846800	C	5.41403300	-8.90600600	0.60393000
H	3.59187100	-9.51495200	-0.96322600	H	4.36240200	-9.23701500	-1.22871700
C	3.41226200	-8.91216000	2.84363900	C	4.07441300	-8.86765200	2.60144500
H	1.29168900	-9.08307300	2.62502200	H	1.97608300	-9.17861400	2.32833500
C	4.65281600	-8.92805400	2.20389300	C	5.32259500	-8.75810500	1.98774900
H	5.66355300	-9.16731700	0.32729300	H	6.38247700	-8.82125700	0.12446400
H	3.36570900	-8.74233500	3.91320500	H	3.99785100	-8.76303000	3.67768300
C	-8.87350500	6.27371500	-0.07915100	C	-9.32498500	5.56750400	0.51669500
C	-7.64045100	5.69943000	-0.42927400	C	-8.12210900	5.14899400	-0.08614400
C	-8.82992900	7.54828300	0.50875300	C	-9.28739300	6.83097300	1.13852600
C	-6.44036900	6.36214000	-0.22473800	C	-6.97682700	5.92642300	-0.07484100
H	-7.62393100	4.71282800	-0.88079300	H	-8.09435500	4.18963600	-0.59180200
C	-7.63279900	8.20190200	0.74766300	C	-8.14369400	7.60690200	1.17386100
H	-9.75800000	8.03493700	0.78974000	H	-10.18900800	7.21129000	1.60712400
C	-6.41215700	7.62546500	0.37705000	C	-6.95786300	7.17563000	0.56141500
H	-5.50824800	5.88274800	-0.50441300	H	-6.07492600	5.54879900	-0.54587800
H	-7.64206500	9.19003200	1.19481100	H	-8.17904300	8.58103300	1.65039100
C	-5.14427500	8.33992000	0.61286100	C	-5.75734500	8.02158000	0.57176400
C	-4.08945600	8.28767000	-0.30378300	C	-4.83722400	8.00743900	-0.48813800
C	-4.95942200	9.11721400	1.76075700	C	-5.50316400	8.90391900	1.63431600
C	-2.91827900	8.99988600	-0.10450700	C	-3.73808300	8.84691800	-0.50477200
H	-4.20452400	7.71715700	-1.21902700	H	-5.01675200	7.36525900	-1.34207500
C	-3.78568700	9.81364600	1.98216600	C	-4.41030500	9.74722900	1.62894400
H	-5.73825400	9.15152400	2.51462900	H	-6.16584100	8.91189300	2.49117600
C	-2.74827200	9.78186200	1.04321100	C	-3.51875200	9.73178900	0.55383500
H	-2.13681200	8.96860400	-0.85413000	H	-3.06935400	8.85022600	-1.35801900
H	-3.66663600	10.38961900	2.89160800	H	-4.23428100	10.41718600	2.46222800
C	6.67633000	-7.63214900	2.46158100	C	7.22362500	-7.31963000	2.38973400
C	8.04468100	-7.82111000	2.25874600	C	8.61682700	-7.35222300	2.28929300
C	6.12060400	-6.38291500	2.18299700	C	6.55899700	-6.12398000	2.10911900
C	8.83190900	-6.78265200	1.78608800	C	9.31939800	-6.21697300	1.91838900
H	8.48802900	-8.78626000	2.47572200	H	9.14665800	-8.27276500	2.50510800
C	6.91320900	-5.35190500	1.70768500	C	7.26887700	-4.99561500	1.73618200
H	5.05831300	-6.22808100	2.33248400	H	5.47813500	-6.08701000	2.17728400
C	8.28382100	-5.52852700	1.49799200	C	8.66240300	-5.01590200	1.63217000
H	9.89507200	-6.94529000	1.64505000	H	10.40157100	-6.26165600	1.85866300
H	6.45747400	-4.39523800	1.47677100	H	6.72914800	-4.08404600	1.50366600
C	9.10946300	-4.42416300	0.97326900	C	9.40196400	-3.80768700	1.22039600

C	8.95570400	-3.11851700	1.44840000
C	10.05355400	-4.63600100	-0.03468900
C	9.70467600	-2.07047800	0.94174300
H	8.25843500	-2.92354000	2.25622800
C	10.78518100	-3.58982800	-0.57136900
H	10.18509500	-5.63278300	-0.44212700
C	10.61900400	-2.28540500	-0.09475700
H	9.58093100	-1.07570200	1.35197400
H	11.48194400	-3.78072900	-1.37843400
C	-0.32312600	10.10639800	0.79732400
C	0.58959300	11.03190500	0.28445000
C	0.06495100	8.76588200	0.87453300
C	1.85544300	10.63267400	-0.10527500
H	0.30934700	12.07596600	0.21328900
C	1.32765800	8.37347800	0.45927700
H	-0.62606600	8.02822700	1.26519600
C	2.26062200	9.29591700	-0.02669300
H	2.55224000	11.38430300	-0.45875200
H	1.59163500	7.32318300	0.51732000
C	3.63082300	8.90186800	-0.40618800
C	4.31172400	7.88142900	0.26539100
C	4.32270100	9.56998900	-1.42325500
C	5.62909900	7.56965200	-0.02931800
H	3.82172100	7.35283700	1.07579900
C	5.63467400	9.26030700	-1.73335300
H	3.81724200	10.33501800	-2.00231500
C	6.31949900	8.26954100	-1.02257600
H	6.13974900	6.79943600	0.53649400
H	6.14050200	9.79162100	-2.53078700
C	10.82044700	0.07016600	-0.75076100
C	11.63954600	1.19371800	-0.60473300
C	9.46207700	0.27717300	-1.01184800
C	11.11580800	2.46980500	-0.71459600
H	12.69753100	1.06464200	-0.41172000
C	8.94410500	1.55884800	-1.08741800
H	8.80349300	-0.57290400	-1.14118800
C	9.75317100	2.69123100	-0.94285300
H	11.79169000	3.31260800	-0.62603900
H	7.87904300	1.67471700	-1.25365700
C	9.20034200	4.05740800	-1.02307800
C	8.04880700	4.34295900	-1.76640500
C	9.81246100	5.12518100	-0.35585700
C	7.53922100	5.62840300	-1.85070700
H	7.55926300	3.55319200	-2.32487100
C	9.31224300	6.41293000	-0.43558900
H	10.68071200	4.94358900	0.26703000
C	8.16999200	6.68405700	-1.19064000
H	6.65768400	5.82557700	-2.44991800
H	9.80185500	7.21914300	0.09817000
C	-12.18563700	-5.36184900	1.14447600
C	-12.93705500	-4.73766700	2.15372300
C	-12.62968400	-6.61619200	0.69473100
C	-14.06395000	-5.33903500	2.69744800
H	-12.62106500	-3.76925800	2.52726100
C	-13.77152700	-7.21127300	1.21357500
H	-12.07561400	-7.12537800	-0.08689000
C	-14.48696500	-6.57550700	2.22232800
H	-14.61663000	-4.84247700	3.48768300
H	-14.10172000	-8.17331700	0.83680400
H	-15.37331000	-7.04314000	2.63766100
C	-11.50827500	6.37179000	-0.68970900
C	-12.78734400	5.97159600	-0.26966800
C	-11.42104800	7.55030800	-1.44878800
C	-13.91903500	6.71448100	-0.57787100
H	-12.89219900	5.06842400	0.32227200
C	-12.55105200	8.28311600	-1.78563700
H	-10.44883300	7.88872800	-1.79148500
C	-13.80279900	7.86916500	-1.34374200
H	-14.89297300	6.39134000	-0.22628500
H	-12.45672100	9.18075000	-2.38720700
H	-14.68648000	8.44571000	-1.59581100
C	-1.67552300	11.78151900	1.92426500
C	-2.63745200	12.72082900	1.54859700
C	-0.79785700	12.08161800	2.96728600
C	-2.72382200	13.93497000	2.21511900
H	-3.31500700	12.49276100	0.73407700
C	-0.87954300	13.30509200	3.61712800
H	-0.05294400	11.35195700	3.26269200
C	-1.84429600	14.23583200	3.24890000
H	-3.47583900	14.65546900	1.91300800
H	-0.19138900	13.52524900	4.42576500
H	-1.90997400	15.18785200	3.76301300
C	8.57438800	9.05497900	-1.55038900
C	8.47225700	10.27294900	-0.87209200
C	9.59743000	8.88580400	-2.48775000
C	9.36811700	11.29843100	-1.13818700
H	7.69020000	10.41127200	-0.13516500
C	10.49873600	9.91124900	-2.73345100
H	9.68028100	7.94662900	-3.02187100
C	10.38879700	11.12559200	-2.06586900
H	9.27361600	12.23630500	-0.60174400
H	11.28557200	9.76133300	-3.46466100
H	11.09033300	11.92729600	-2.26568000
C	12.68032900	-1.46293300	-1.09907400
C	13.59723600	-2.12765900	-0.28330300
C	13.07724500	-1.03503200	-2.36693300
C	14.88733200	-2.36658500	-0.73552600

C	9.06397400	-2.54821200	1.72501100
C	10.45054500	-3.87438900	0.29885500
C	9.73874600	-1.40568800	1.32986000
H	8.28039500	-2.46541100	2.47036600
C	11.11193700	-2.73440100	-0.12594500
H	10.72550600	-4.83238600	-0.12896700
C	10.76190900	-1.47998600	0.38067100
H	9.47505800	-0.44855000	1.76361700
H	11.89864200	-2.81300500	-0.86655600
C	-1.16880600	10.28289000	0.17482000
C	-0.26175200	11.23410800	-0.34070300
C	-0.71898700	8.95317200	0.32873100
C	1.02374700	10.87615500	-0.65514800
H	-0.58704400	12.25395600	-0.49607800
C	0.57709500	8.61530400	0.02340900
H	-1.39265300	8.20197800	0.71914000
C	1.50126800	9.56106300	-0.46996300
H	1.68909200	11.64449800	-1.02753200
H	0.88196800	7.58550100	0.16299100
C	2.88657800	9.22638000	-0.73882900
C	3.51492000	8.11243100	-0.14346800
C	3.68857500	10.03198900	-1.57518000
C	4.85165200	7.84685100	-0.32359900
H	2.95692700	7.47589000	0.53168800
C	5.02290600	9.77767000	-1.76799300
H	3.24365700	10.85685300	-2.11706800
C	5.64899400	8.69187500	-1.12177800
H	5.30749100	7.01345500	0.19506600
H	5.59854000	10.40608200	-2.43483300
C	10.79484400	0.89895700	-0.22413600
C	11.48802800	2.10465800	-0.05411400
C	9.44114700	0.96724300	-0.58127600
C	10.85088300	3.31767500	-0.23012500
H	12.53658000	2.08796600	0.21470200
C	8.81075700	2.18828400	-0.73411300
H	8.87794600	0.05534300	-0.73356800
C	9.49326500	3.39889200	-0.56349100
H	11.43495400	4.22315800	-0.11454700
H	7.75495000	2.19202200	-0.97998300
C	8.82435000	4.70001100	-0.72582900
C	7.69051300	4.85158700	-1.53674900
C	9.30886100	5.84562600	-0.07771500
C	7.07541200	6.08036600	-1.70097300
H	7.30327800	4.00206800	-2.08648800
C	8.71130800	7.07964100	-0.24406700
H	10.15568500	5.76736400	0.59294900
C	7.58568200	7.20966400	-1.05871800
H	6.21783000	6.17656800	-2.35688800
H	9.10362300	7.94837500	0.27112100
C	-11.67990200	-6.44597300	0.98184300
C	-12.45745500	-6.04225200	2.08051200
C	-12.03378000	-7.66085300	0.37068600
C	-13.52309800	-6.80326800	2.54328800
H	-12.20936200	-5.11602800	2.58864900
C	-13.10940600	-8.41915800	0.81380300
H	-11.45569900	-8.01086800	-0.47871400
C	-13.85740000	-7.99346200	1.90627700
H	-14.09569200	-6.46737300	3.40177900
H	-13.36382600	-9.34569300	0.30925600
H	-14.69379900	-8.58682200	2.26066500
C	-12.01766200	5.48389200	0.42683300
C	-13.14450500	5.03963700	1.14077900
C	-12.17995800	6.65988300	-0.32678800
C	-14.35400200	5.72183800	1.10719000
H	-13.06197800	4.14254100	1.74602800
C	-13.38926900	7.34048200	-0.38040100
H	-11.33599300	7.04154800	-0.89328900
C	-14.48394800	6.87503300	0.34079100
H	-15.19858200	5.35308400	1.68062900
H	-13.47960500	8.23717800	-0.98545800
H	-15.42901500	7.40694300	0.30680200
C	-2.73611900	12.00064500	0.86926900
C	-3.83051500	12.64122700	0.28815800
C	-1.95100300	12.66832700	1.81022400
C	-4.12709600	13.94857300	0.64171900
H	-4.43662700	12.11385500	-0.43860400
C	-2.25487700	13.97729800	2.15345500
H	-1.11879400	12.15457300	2.27701200
C	-3.34129000	14.62113200	1.57165300
H	-4.97424100	14.44535200	0.18351200
H	-1.64817500	14.49002400	2.89084100
H	-3.57888900	15.64211500	1.84636900
C	7.87765400	9.56302100	-1.55373300
C	7.79504800	10.75650700	-0.83527200
C	8.84102800	9.41376500	-2.55112900
C	8.66359100	11.79753200	-1.12837600
H	7.05959300	10.85835900	-0.04586400
C	9.70961400	10.45806300	-2.83061200
H	8.89946900	8.48401900	-3.10435500
C	9.62275300	11.65283400	-2.12451400
H	8.59919300	12.72088200	-0.56441400
H	10.45281200	10.33902500	-3.61048800
H	10.30270800	12.46669300	-2.34720300
C	12.83993000	-0.42012000	-0.30386300
C	13.69156800	-0.96403900	0.65754600
C	13.36495600	0.01862200	-1.51967100
C	15.05124700	-1.07071900	0.40062900

H	13.29185000	-2.45498200	0.70375800	H	13.28113300	-1.30250900	1.60180900
C	14.37435800	-1.26214800	-2.80483400	C	14.72804700	-0.07680900	-1.76270700
H	12.36432800	-0.52383300	-3.00344800	H	12.69986200	0.43136200	-2.26946800
C	15.28435900	-1.93206400	-1.99497200	C	15.57618800	-0.62431900	-0.80682700
H	15.58992200	-2.88399800	-0.09163100	H	15.70477800	-1.49545300	1.15436100
H	14.66996700	-0.92413500	-3.79190800	H	15.12589100	0.26590900	-2.71135700
H	16.29489300	-2.11387100	-2.34250300	H	16.63930700	-0.70435900	-1.00254800
C	-2.50609900	-10.54437500	-3.92727700	C	-1.59585700	-10.40520100	-4.39906900
C	-1.37506100	-10.43676700	-4.75245600	C	-0.46367300	-10.12473800	-5.18090500
C	-3.58837100	-11.29445200	-4.41541900	C	-2.60090400	-11.19143200	-4.98550500
C	-1.32813500	-11.03921400	-6.00267100	C	-0.34328400	-10.59535800	-6.48182300
H	-0.52416400	-9.85505100	-4.41369200	H	0.32910200	-9.51144500	-4.76524400
C	-3.53705800	-11.92570400	-5.65087400	C	-2.47513000	-11.69031700	-6.27513200
H	-4.48187800	-11.39182100	-3.80735100	H	-3.49302500	-11.42228000	-4.41249300
C	-2.40704400	-11.79337000	-6.45024500	C	-1.34662500	-11.38737700	-7.02886100
H	-0.44854000	-10.92539900	-6.62712400	H	0.53487700	-10.34856300	-7.06902300
H	-4.38093000	-12.51357000	-5.99560900	H	-3.26053900	-12.30839700	-6.69676100
H	-2.36857100	-12.27466600	-7.42162900	H	-1.25070600	-11.76538200	-8.04117600
C	6.16227500	-9.41263200	4.07271300	C	6.84170600	-9.26702200	3.83851700
C	5.69869900	-10.72411100	4.23213000	C	6.50926600	-10.62644800	3.86047500
C	6.94219300	-8.84871700	5.08918700	C	7.52562500	-8.72513100	4.93285300
C	6.00283100	-11.44148500	5.37934800	C	6.84876900	-11.41424700	4.95027300
H	5.10284000	-11.18021900	3.45083700	H	5.98633300	-11.06346800	3.01841500
C	7.25244300	-9.58330600	6.22418100	C	7.87408600	-9.52789900	6.00898700
H	7.30035200	-7.83141200	4.98922100	H	7.77785800	-7.67174800	4.94004500
C	6.78454200	-10.88222200	6.38338500	C	7.53754100	-10.87622100	6.03092100
H	5.63230800	-12.45600100	5.47967100	H	6.58021600	-12.46508900	4.94481500
H	7.85780300	-9.12455800	6.99859300	H	8.40293500	-9.08558000	6.84640300
H	7.02506500	-11.44929700	7.27508500	H	7.80619800	-11.49733100	6.87744300
N	-1.58483500	10.53805300	1.25129100	N	-2.44506900	10.65398600	0.52385700
N	7.67276600	8.00149400	-1.29215800	N	7.00436900	8.48388300	-1.25476800
N	11.36006100	-1.22447600	-0.64495000	N	11.44717000	-0.32514700	-0.04928500
N	5.84928000	-8.68415000	2.91737000	N	6.48590800	-8.46867600	2.74165000

Structure S50. The coordinates of both the optimized structures of B2N5L$_2$ at S_0 and S_1 in toluene.

S_0-optimized structure				S_1-optimized structure			
B	-10.84918000	-4.64155000	0.42773200	B	-10.65926900	-5.00576300	0.35196700
C	-9.63343800	-5.50465800	-0.04034500	C	-9.41278000	-5.81838300	-0.15306100
C	-8.77394100	-5.06588400	-1.06057100	C	-8.55055400	-5.31128300	-1.13972500
C	-9.32792600	-6.74264400	0.54858100	C	-9.09538500	-7.09212600	0.34852900
C	-7.67851400	-5.80945400	-1.46452900	C	-7.44174800	-6.01343200	-1.58123100
H	-8.96916600	-4.11657400	-1.54775700	H	-8.75715200	-4.33752200	-1.57072800
C	-8.22059300	-7.48231300	0.16492200	C	-7.97964200	-7.79627500	-0.07665000
H	-9.96734000	-7.12760600	1.33602600	H	-9.73911100	-7.53847700	1.09969900
C	-7.37049900	-7.02892400	-0.85015300	C	-7.12387800	-7.26884000	-1.05043700
H	-7.02815100	-5.42101200	-2.24052400	H	-6.79214600	-5.56602700	-2.32601300
H	-8.02297200	-8.43546100	0.64339900	H	-7.78051400	-8.77973300	0.33644500
C	-6.16964400	-7.78706300	-1.24964000	C	-5.91351000	-7.98728000	-1.49272800
C	-5.70943600	-7.77039800	-2.56998100	C	-5.43226500	-7.85991500	-2.80023100
C	-5.42620000	-8.51886400	-0.31739900	C	-5.18039000	-8.79322900	-0.61423700
C	-4.54225700	-8.41648700	-2.93756100	C	-4.25487900	-8.46752600	-3.20136700
H	-6.27390500	-7.24117500	-3.32930000	H	-5.98847800	-7.27105500	-3.52061900
C	-4.26650100	-9.18346500	-0.67886100	C	-4.01384200	-9.42458600	-1.01328000
H	-5.74632000	-8.55211700	0.71797800	H	-5.51564600	-8.91188300	0.40996000
C	-3.79294600	-9.12127300	-1.99230800	C	-3.52098900	-9.24895800	-2.30777100
H	-4.20180200	-8.37107700	-3.96496600	H	-3.89659400	-8.33489400	-4.21544700
H	-3.70780400	-9.73771700	0.06597400	H	-3.46250500	-10.03785000	-0.30992300
C	-10.75776100	-3.08071800	0.35438900	C	-10.60737900	-3.46521600	0.43142600
C	-9.54208300	-2.40420300	0.54558100	C	-9.39350900	-2.75027500	0.55800400
C	-11.88209200	-2.28775700	0.07453000	C	-11.77022700	-2.66333500	0.36460800
C	-9.45233300	-1.02433500	0.46529900	C	-9.34133600	-1.37416300	0.60656100
H	-8.64577500	-2.97453500	0.76481500	H	-8.46352800	-3.30481300	0.63071300
C	-11.79518700	-0.90898400	-0.03447100	C	-11.72600700	-1.28595500	0.38896500
H	-12.84515900	-2.76526500	-0.07217800	H	-12.73673700	-3.14794400	0.27029800
C	-10.57678700	-0.24966200	0.16110100	C	-10.50683000	-0.58402000	0.50795700
H	-8.49110500	-0.54188900	0.60470600	H	-8.37364300	-0.89175000	0.68548700
H	-12.68840700	-0.33172400	-0.24707500	H	-12.65763800	-0.73373300	0.34042700
C	-10.47675200	1.21881100	0.04384500	C	-10.45286200	0.86908600	0.51672200
C	-9.60499500	1.94994900	0.85802200	C	-9.34176000	1.57652000	1.02708100
C	-11.24757700	1.91991800	-0.88994700	C	-11.50683200	1.65752500	0.00474900
C	-9.50168000	3.32589300	0.73134600	C	-9.28894800	2.95238400	1.01405200
H	-9.01842800	1.43622700	1.61175000	H	-8.52214300	1.02861600	1.47913300
C	-11.16108500	3.29898800	-0.98909200	C	-11.45028300	3.03267400	0.00517500
H	-11.90666600	1.37507100	-1.55695800	H	-12.36850700	1.17291900	-0.44089700
C	-10.28221900	4.04510700	-0.18760600	C	-10.33796700	3.75881400	0.50289100
H	-8.81543900	3.86184900	1.37859400	H	-8.42135900	3.43743400	1.45004700
H	-11.77573600	3.81108800	-1.72186200	H	-12.28085800	3.58229600	-0.42549100
B	-10.18814300	5.60273100	-0.29792700	B	-10.29426400	5.29717300	0.49775500
C	-1.43575100	-9.63909400	-1.56651800	C	-1.15245600	-9.72179400	-1.91517400
C	-0.42868400	-10.60885100	-1.63773300	C	-0.10720800	-10.64782900	-2.02489800
C	-1.25117800	-8.55980400	-0.69505200	C	-1.01206700	-8.67875400	-0.99165700
C	0.72862400	-10.48198000	-0.88859300	C	1.03968100	-10.51383100	-1.26128500
H	-0.55420900	-11.46609100	-2.28761300	H	-0.19658100	-11.47909000	-2.71326700
C	-0.10570800	-8.46418200	0.07697200	C	0.12416000	-8.57753900	-0.20753000
H	-2.00146500	-7.78180600	-0.63205900	H	-1.79311300	-7.93552700	-0.89465300
C	0.91699000	-9.41137100	-0.01076400	C	1.18191700	-9.48131000	-0.33065500
H	1.49034500	-11.25088000	-0.96355400	H	1.82892000	-11.25103800	-1.36551200
H	0.01740100	-7.60554100	0.72853800	H	0.20932900	-7.74789600	0.48641000
C	2.16079100	-9.26425800	0.77061500	C	2.40891400	-9.33115800	0.47568200
C	3.40808400	-9.41277600	0.15741100	C	3.67072000	-9.41536600	-0.12029200
C	2.13163000	-8.94790000	2.13263100	C	2.34660600	-9.07802500	1.84995400
C	4.58201200	-9.23577900	0.87167500	C	4.82649600	-9.23765700	0.62334400
H	3.45708100	-9.64597400	-0.90067800	H	3.74464300	-9.59944000	-1.18660400
C	3.30270300	-8.77348500	2.85415000	C	3.49924100	-8.90331100	2.60038900
H	1.17583400	-8.84268300	2.63458200	H	1.37941600	-9.02509400	2.33790000
C	4.54101200	-8.90715300	2.22591400	C	4.75084000	-8.97299900	1.98968100
H	5.54299400	-9.33790100	0.38049300	H	5.79901700	-9.29058800	0.14717800
H	3.26464300	-8.52785100	3.90943200	H	3.43632400	-8.70832500	3.66505200
C	-8.82940500	6.32869200	-0.03835800	C	-8.93763400	6.08272600	0.56426500
C	-7.59805500	5.75090100	-0.38869700	C	-7.74492800	5.60203300	-0.01394600
C	-8.78177600	7.60003600	0.55618900	C	-8.84794800	7.34845000	1.17803700
C	-6.39577100	6.40807000	-0.17967900	C	-6.56534500	6.32509800	0.00691700
H	-7.58482200	4.76619100	-0.84440400	H	-7.75493200	4.63766100	-0.51084200
C	-7.58241300	8.24790800	0.79976700	C	-7.66992300	8.06967600	1.22457700
H	-9.70835500	8.08888500	0.83833600	H	-9.73738800	7.77523000	1.62969200
C	-6.36358000	7.66878500	0.42729700	C	-6.49665500	7.57956100	0.63103100
H	-5.46508300	5.92643200	-0.46024200	H	-5.67562900	5.90137800	-0.44805400
H	-7.58836000	9.23387300	1.25174200	H	-7.66690100	9.04841100	1.69290800
C	-5.09296000	8.37748900	0.66553900	C	-5.26134500	8.37134600	0.64388000
C	-4.03950700	8.32502000	-0.25266500	C	-4.33181200	8.30157600	-0.40647400
C	-4.90343000	9.14874500	1.81670300	C	-4.97831600	9.25849300	1.69605100
C	-2.86488000	9.03080700	-0.05136300	C	-3.19725100	9.09146700	-0.42431700
H	-4.15830800	7.75946800	-1.17050100	H	-4.53215000	7.65604800	-1.25320000
C	-3.72608200	9.83850000	2.04022100	C	-3.84914900	10.05200600	1.68995700
H	-5.68137500	9.18331300	2.57149100	H	-5.64796000	9.30815400	2.54609000
C	-2.68965500	9.80604800	1.10014900	C	-2.94817700	9.98127900	0.62429400
H	-2.08447600	8.99948000	-0.80207000	H	-2.52179800	9.05350300	-1.27141800
H	-3.60337600	10.40943500	2.95234400	H	-3.65083800	10.72438000	2.51625400
C	6.58428400	-7.63987200	2.46467100	C	6.73993800	-7.65036900	2.36659300
C	7.95028000	-7.84178400	2.25570100	C	8.12924200	-7.76196700	2.26274500
C	6.03852300	-6.38818600	2.17680400	C	6.14363300	-6.42224000	2.07222000
C	8.74433700	-6.81392000	1.77158400	C	8.89394000	-6.67154400	1.88091700
H	8.38667800	-8.80904400	2.47732100	H	8.60730300	-8.70881800	2.48543300
C	6.83803700	-5.36765800	1.69053300	C	6.91560100	-5.33866800	1.68953000
H	4.97807400	-6.22292000	2.32786500	H	5.06655200	-6.32398100	2.13896100
C	8.20625900	-5.55692800	1.47732000	C	8.30574600	-5.43726400	1.58681000
H	9.80533400	-6.98719000	1.62657200	H	9.97176900	-6.77760200	1.81949600
H	6.38932900	-4.40903600	1.45385200	H	6.42725700	-4.40045000	1.44929800
C	9.03939200	-4.46231500	0.94400400	C	9.11364200	-4.27489600	1.17164800
C	8.90053700	-3.15395800	1.41626100	C	8.85026600	-2.99732900	1.67513700

C	9.97618200	-4.68576700	-0.06822800
C	9.65689100	-2.11443000	0.90303100
H	8.20923000	-2.95031500	2.22707900
C	10.71491800	-3.64795300	-0.61155700
H	10.09629500	-5.68493100	-0.47336300
C	10.56379600	-2.34081500	-0.13746400
H	9.54467800	-1.11747800	1.31128800
H	11.40573600	-3.84762700	-1.42163100
C	-0.26379200	10.11869100	0.84953600
C	0.65143300	11.04196600	0.33720100
C	0.11750900	8.77579000	0.91825400
C	1.91308100	10.63788100	-0.06122800
H	0.37650500	12.08789400	0.27292300
C	1.37582000	8.37868300	0.49434000
H	-0.57555500	8.03990000	1.30862600
C	2.31115800	9.29847600	0.00789400
H	2.61208700	11.38755200	-0.41446400
H	1.63437600	7.32669900	0.54551000
C	3.67644400	8.89803400	-0.38245100
C	4.35479200	7.86904000	0.27857300
C	4.36602000	9.56709000	-1.40045900
C	5.66767500	7.54938100	-0.02739800
H	3.86677300	7.33928800	1.08942400
C	5.67337700	9.24954900	-1.72191500
H	3.86206100	10.33906800	-1.97156100
C	6.35593800	8.24944300	-1.02206400
H	6.17665400	6.77219200	0.53030800
H	6.17715300	9.78167400	-2.52010100
C	10.78535100	0.01148000	-0.79829100
C	11.61575700	1.12718000	-0.65585400
C	9.42786500	0.23153100	-1.05332200
C	11.10350900	2.40824400	-0.76211900
H	12.67336500	0.98803500	-0.46786300
C	8.92162600	1.51806100	-1.12548500
H	8.76071200	-0.61225300	-1.17995500
C	9.74188400	2.64270000	-0.98331500
H	11.78778800	3.24449300	-0.67582600
H	7.85691300	1.64399100	-1.28652100
C	9.20105700	4.01403800	-1.05758500
C	8.04793100	4.31179600	-1.79365500
C	9.82612700	5.07448500	-0.39067800
C	7.54874800	5.60177300	-1.87082300
H	7.54855500	3.52799200	-2.35180200
C	9.33637700	6.36668200	-0.46337400
H	10.69625500	4.88356500	0.22675400
C	8.19197200	6.64978500	-1.21064100
H	6.66541100	5.80841800	-2.46418400
H	9.83573800	7.16699500	0.07024700
C	-12.14849300	-5.32853300	0.96402200
C	-12.93090700	-4.73164000	1.96608600
C	-12.58158100	-6.56699600	0.46276000
C	-14.07684300	-5.34443300	2.45462500
H	-12.62495300	-3.77552700	2.37782600
C	-13.74169400	-7.17269200	0.92600500
H	-12.00380900	-7.05487600	-0.31530300
C	-14.48795000	-6.56452200	1.92937900
H	-14.65371700	-4.86908500	3.24062200
H	-14.06227900	-8.12171300	0.50990000
H	-15.38893100	-7.04065700	2.30130100
C	-11.46207400	6.43730000	-0.66042000
C	-12.74405000	6.03150700	-0.25479200
C	-11.36983300	7.62448800	-1.40527600
C	-13.87381200	6.77740100	-0.56270300
H	-12.85273900	5.12121300	0.32543200
C	-12.49769300	8.36072800	-1.74181600
H	-10.39534700	7.96705000	-1.73722000
C	-13.75245000	7.94111000	-1.31399600
H	-14.85020300	6.44964700	-0.22236500
H	-12.39940700	9.26532100	-2.33222600
H	-14.63454200	8.52017400	-1.56586500
C	-1.60319400	11.79407800	1.99153500
C	-2.56110700	12.74102100	1.62485400
C	-0.72015500	12.08302300	3.03316800
C	-2.63828000	13.95162300	2.29889100
H	-3.24278100	12.52167900	0.81137300
C	-0.79264600	13.30304300	3.69055000
H	0.02165600	11.34740300	3.32153500
C	-1.75344000	14.24136800	3.33130800
H	-3.38727700	14.67813800	2.00375700
H	-0.10037400	13.51451200	4.49799200
H	-1.81189500	15.19065900	3.85131000
C	8.61283600	9.01831900	-1.56657600
C	8.52702400	10.23484700	-0.88343700
C	9.62635500	8.84347900	-2.51319500
C	9.42938100	11.25347300	-1.15388100
H	7.75258400	10.37740500	-0.13930800
C	10.53430000	9.86196700	-2.76333300
H	9.69653900	7.90539100	-3.05103400
C	10.44053600	11.07498000	-2.09088100
H	9.34765300	12.19028600	-0.61349800
H	11.31355900	9.70773600	-3.50172200
H	11.14719600	11.87131500	-2.29406900
C	12.62912100	-1.53985100	-1.15136600
C	13.54181900	-2.21285600	-0.33764100
C	13.02684700	-1.11645500	-2.42051100
C	14.82838900	-2.46428700	-0.79310000
H	13.23591600	-2.53683600	0.65035500

C	10.15753800	-4.40392800	0.25148000
C	9.59193900	-1.89686600	1.28046300
H	8.07230200	-2.86829700	2.41983000
C	10.88548200	-3.30511200	-0.17327900
H	10.37657500	-5.37703100	-0.17461100
C	10.60986500	-2.03183500	0.33231600
H	9.38465700	-0.92588700	1.71405000
H	11.66723200	-3.43056400	-0.91269100
C	-0.57495300	10.41541200	0.24759100
C	0.37484900	11.31489400	-0.28344200
C	-0.18980800	9.06778300	0.41876900
C	1.63943000	10.88975600	-0.60000000
H	0.09879500	12.34738100	-0.45078600
C	1.08583200	8.66210700	0.11025100
H	-0.89754800	8.35637400	0.82320900
C	2.05144100	9.55452200	-0.40237700
H	2.34035100	11.61882400	-0.98604500
H	1.34007900	7.61993800	0.25965000
C	3.41376200	9.14378800	-0.68459900
C	3.99429200	8.01434500	-0.07077300
C	4.23917400	9.88316000	-1.55804800
C	5.30944600	7.66968700	-0.27443700
H	3.41887300	7.42970400	0.63614900
C	5.55244400	9.54892300	-1.77497300
H	3.82784500	10.71727100	-2.11231200
C	6.13152000	8.44454500	-1.11714200
H	5.73171400	6.82653900	0.25684800
H	6.14531500	10.12640200	-2.47217200
C	10.78808100	0.34221200	-0.26837400
C	11.55590000	1.50269700	-0.10441500
C	9.43878100	0.49470000	-0.61577900
C	10.99378700	2.75311700	-0.27577900
H	12.60353500	1.42126300	0.15611700
C	8.88383700	1.75248800	-0.76238700
H	8.81883100	-0.38021800	-0.76438200
C	9.64086500	2.91854900	-0.59636600
H	11.63425800	3.62065300	-0.16699400
H	7.82850300	1.82117300	-1.00068100
C	9.05107300	4.25834000	-0.75236400
C	7.91684800	4.47650500	-1.54699600
C	9.61391700	5.37388100	-0.11533700
C	7.37712300	5.74045000	-1.70882100
H	7.46861300	3.65016400	-2.08545500
C	9.09261800	6.64247300	-0.28100600
H	10.46272500	5.24501600	0.54500700
C	7.96706100	6.83885700	-1.08183300
H	6.51682300	5.88672500	-2.35165500
H	9.54604500	7.48786100	0.22283600
C	-11.95643200	-5.79347700	0.76629400
C	-12.76212200	-5.37562800	1.83889600
C	-12.35672600	-6.95915800	0.09182300
C	-13.89768700	-6.07888700	2.21945800
H	-12.48112900	-4.48545000	2.39234200
C	-13.50204500	-7.65738500	0.45139800
H	-11.75950700	-7.31765500	-0.74065000
C	-14.27552600	-7.22086200	1.52161400
H	-14.49099000	-5.73505700	3.06052000
H	-13.79083500	-8.54602900	-0.10053800
H	-15.16623700	-7.76855300	1.81125400
C	-11.62942400	6.13310500	0.41941600
C	-12.79591900	5.73507200	1.09667300
C	-11.71722500	7.32356400	-0.32429900
C	-13.97132600	6.47275200	1.03778000
H	-12.77212700	4.82869600	1.69328400
C	-12.89145700	8.06085300	-0.40205600
H	-10.84176800	7.67129700	-0.86421300
C	-14.02658300	7.63953800	0.28288600
H	-14.84771000	6.13711700	1.58326200
H	-12.92295300	8.96734000	-0.99850700
H	-14.94465800	8.21535600	0.22959000
C	-2.05651500	12.21199900	0.92910300
C	-3.12068800	12.90115800	0.34704000
C	-1.23529100	12.84783000	1.86147400
C	-3.35074100	14.22430100	0.69095500
H	-3.75484800	12.39872400	-0.37337400
C	-1.47280000	14.17280600	2.19508400
H	-0.42734400	12.29746600	2.32908600
C	-2.52870900	14.86475100	1.61228700
H	-4.17426900	14.75851100	0.23185600
H	-0.83821900	14.66096500	2.92561600
H	-2.71448600	15.89842300	1.87938500
C	8.39977100	9.15569700	-1.64051100
C	8.41765900	10.37404700	-0.96003400
C	9.32224600	8.91183900	-2.65801200
C	9.34486400	11.34459700	-1.30997100
H	7.71420800	10.55005900	-0.15481600
C	10.25018900	9.88597000	-2.99429500
H	9.30318900	7.96385300	-3.18228500
C	10.26355300	11.10514500	-2.32571100
H	9.35771500	12.28688600	-0.77447300
H	10.96118900	9.69298300	-3.78922300
H	10.98951000	11.86417000	-2.59248000
C	12.74848600	-1.09996800	-0.35020800
C	13.56526200	-1.68799700	0.61541200
C	13.29957400	-0.70134400	-1.56822000
C	14.91622200	-1.87798500	0.36059300
H	13.13442700	-1.99499200	1.56139600

C	14.32059300	-1.35611200	-2.86165400	C	14.65465000	-0.88015000	-1.80932500
H	12.31714800	-0.59887900	-3.05547300	H	12.66080400	-0.25395900	-2.32102100
C	15.22630100	-2.03422900	-2.05380100	C	15.46768300	-1.47176400	-0.84922700
H	15.52765800	-2.98809000	-0.15075500	H	15.54245200	-2.33654300	1.11768000
H	14.61680400	-1.02147800	-3.84970500	H	15.07304500	-0.56796300	-2.75968600
H	16.23414300	-2.22588000	-2.40385100	H	16.52411800	-1.61721000	-1.04349700
C	-2.51278000	-10.36415800	-3.63720000	C	-2.23088500	-10.41738600	-3.99966300
C	-1.49083300	-10.04133400	-4.53153200	C	-1.20313900	-10.08402300	-4.88444800
C	-3.48289000	-11.29394800	-4.01373500	C	-3.21639900	-11.31786700	-4.40839500
C	-1.43728800	-10.65125700	-5.77708700	C	-1.15831600	-10.65527200	-6.14848000
H	-0.74106000	-9.31315500	-4.24520800	H	-0.44245000	-9.37613800	-4.57691500
C	-3.43232200	-11.88624500	-5.26767600	C	-3.17400100	-11.87075400	-5.68025400
H	-4.27466600	-11.54642500	-3.31791900	H	-4.01504800	-11.57749300	-3.72329000
C	-2.40779400	-11.57280200	-6.15342200	C	-2.14321700	-11.54798200	-6.55537300
H	-0.63790300	-10.39210300	-6.46246700	H	-0.35425700	-10.38660500	-6.82484700
H	-4.19278200	-12.60676400	-5.54759400	H	-3.94752200	-12.56768900	-5.98331200
H	-2.36628000	-12.04233600	-7.12952000	H	-2.10893400	-11.98630700	-7.54617500
C	6.05944100	-9.41795200	4.07917300	C	6.25472400	-9.57175500	3.82331500
C	5.53442400	-10.70386300	4.25897900	C	5.80257600	-10.89614500	3.86371400
C	6.89208800	-8.89030100	5.07399000	C	7.01059100	-9.08879600	4.89849500
C	5.83286300	-11.43134600	5.40135900	C	6.09852700	-11.70666300	4.94956400
H	4.89439700	-11.13216400	3.49725600	H	5.21992800	-11.28796300	3.03888800
C	7.19429000	-9.63534400	6.20413800	C	7.31226700	-9.91493900	5.97091000
H	7.29722700	-7.89216900	4.96173500	H	7.35525600	-8.06200100	4.89460200
C	6.66763300	-10.90914800	6.38221100	C	6.85877500	-11.22823500	6.00998500
H	5.41387900	-12.42530800	5.51631600	H	5.73661200	-12.72915000	4.95728000
H	7.84118700	-9.20377800	6.96047600	H	7.89829600	-9.51739100	6.79264300
H	6.90287700	-11.48423300	7.27017200	H	7.09232000	-11.86721500	6.85361100
N	-1.52160500	10.55426200	1.31082100	N	-1.83232800	10.85076200	0.59296500
N	7.70460800	7.97162400	-1.30404400	N	7.46680000	8.14712900	-1.28363400
N	11.31246800	-1.28834300	-0.69409200	N	11.36367800	-0.92022000	-0.09718600
N	5.74977300	-8.68035600	2.93083700	N	5.93976400	-8.75112700	2.73211900
N	-2.58696500	-9.74880700	-2.36307100	N	-2.30095100	-9.83969500	-2.71004800

Structure S51. The coordinates of both the optimized structures of B1N6L$_2$ at S_0 and S_1 in toluene.

S_0-optimized structure				S_1-optimized structure			
C	-10.04609800	-4.55938500	0.01790000	C	-9.43407200	-5.79407500	0.10551200
C	-9.24727600	-4.26751600	-1.09092900	C	-8.73658400	-5.26926500	-1.00071000
C	-9.80622800	-5.74757200	0.71337400	C	-8.98691500	-7.00772100	0.66613300
C	-8.22268900	-5.11905000	-1.46427800	C	-7.63720700	-5.92144700	-1.50376200
H	-9.41927700	-3.35607600	-1.65068700	H	-9.07639600	-4.34839100	-1.45641600
C	-8.79449100	-6.60537700	0.31489200	C	-7.87436000	-7.63862300	0.16052800
H	-10.41934700	-5.99994600	1.57026200	H	-9.50318900	-7.42638200	1.52072500
C	-7.96700500	-6.30820100	-0.77294100	C	-7.15445800	-7.11436700	-0.93192900
H	-7.59690100	-4.84079500	-2.30474300	H	-7.11424000	-5.47629800	-2.34105400
H	-8.64863000	-7.52847100	0.86494300	H	-7.55523600	-8.56701800	0.61857500
C	-6.84891000	-7.18752200	-1.16537900	C	-5.93678500	-7.74257000	-1.43062200
C	-6.43603600	-7.28753400	-2.49791600	C	-5.49752100	-7.54042900	-2.75040000
C	-6.13438200	-7.92753000	-0.21686400	C	-5.12182200	-8.53409400	-0.60241400
C	-5.33890600	-8.04872600	-2.86237100	C	-4.30177700	-8.05240200	-3.20460400
H	-6.98083100	-6.75761300	-3.27108400	H	-6.10867700	-6.97168100	-3.44077900
C	-5.05187400	-8.71347300	-0.57497400	C	-3.92039900	-9.04646100	-1.04434500
H	-6.41506300	-7.87372400	0.82910100	H	-5.41445400	-8.72179900	0.42388100
C	-4.61996600	-8.76488900	-1.90272200	C	-3.47017700	-8.79149700	-2.34934800
H	-5.03238600	-8.08854500	-3.90073000	H	-3.98941700	-7.86176100	-4.22348100
H	-4.51815700	-9.27295900	0.18416100	H	-3.31000300	-9.63843000	-0.37373500
C	-10.86237600	-2.28357700	0.34874300	C	-10.50787900	-3.69166000	0.59917900
C	-9.61706100	-1.72076200	0.64458500	C	-9.34199900	-2.96510700	0.89835600
C	-11.90234100	-1.43013300	-0.03079900	C	-11.66743100	-2.98643800	0.23537000
C	-9.41732400	-0.35553300	0.53832000	C	-9.33331600	-1.59424800	0.81300100
H	-8.79732100	-2.36162900	0.94586000	H	-8.44636600	-3.49441000	1.20298700
C	-11.69613200	-0.06394300	-0.11597900	C	-11.64871300	-1.61454700	0.15251800
H	-12.87813000	-1.84242600	-0.25780900	H	-12.57605100	-3.53151000	0.00626500
C	-10.44875000	0.50811900	0.15516600	C	-10.48017800	-0.85495100	0.42546000
H	-8.43028100	0.04305000	0.74416800	H	-8.40926200	-1.07462700	1.03082800
H	-12.52934000	0.57176600	-0.39382500	H	-12.56551000	-1.10615300	-0.11700100
C	-10.22833000	1.96210200	0.03330400	C	-10.45555100	0.58502000	0.31075100
C	-9.32300200	2.63259500	0.86429900	C	-9.41849100	1.36826900	0.87855600
C	-10.91693700	2.71694700	-0.92379700	C	-11.46083400	1.30087900	-0.38732200
C	-9.10750500	3.99465800	0.73049500	C	-9.39036100	2.73568300	0.74955600
H	-8.79692600	2.08366000	1.63768200	H	-8.64506300	0.89114400	1.47014300
C	-10.72382200	4.08445400	-1.02640200	C	-11.42584100	2.66932900	-0.50383800
H	-11.59823200	2.22209200	-1.60714300	H	-12.25895200	0.76109900	-0.88480100
C	-9.80872400	4.76745000	-0.20905300	C	-10.38915300	3.46829000	0.05167400
H	-8.39617000	4.47960100	1.39093600	H	-8.58649100	3.28153800	1.23230300
H	-11.28033100	4.63679500	-1.77633300	H	-12.20897600	3.15767900	-1.07419300
B	-9.59500900	6.31112200	-0.32485500	B	-10.36184400	5.00265600	-0.08008500
C	-2.30679000	-9.47724700	-1.51370300	C	-1.10189800	-9.24740300	-1.94136000
C	-1.39809200	-10.54160100	-1.55470600	C	-0.07382200	-10.17511000	-2.13744200
C	-1.99791900	-8.37843300	-0.70346500	C	-0.96597900	-8.30556500	-0.91623000
C	-0.21810400	-10.49190700	-0.83214600	C	1.06405000	-10.13832900	-1.35001500
H	-1.61969200	-11.41362100	-2.15751700	H	-0.16736200	-10.92409400	-2.91432000
C	-0.83298100	-8.35806700	0.04458800	C	0.16012400	-8.30230700	-0.11216200
H	-2.66809400	-7.52892300	-0.66745700	H	-1.73289700	-7.55642800	-0.76418000
C	0.08963300	-9.40512200	-0.00997200	C	1.20722400	-9.20460200	-0.31943100
H	0.46423100	-11.33407400	-0.88047100	H	1.84767600	-10.86830800	-1.52127700
H	-0.61365600	-7.48557300	0.65099300	H	0.25398900	-7.54521500	0.65876000
C	1.35155500	-9.35025500	0.75427400	C	2.43621200	-9.13306400	0.49359400
C	2.57243600	-9.63564800	0.13662700	C	3.69356200	-9.16045000	-0.11640700
C	1.36550800	-8.99390200	2.10655400	C	2.38244800	-8.99368800	1.88404700
C	3.76479900	-9.55431900	0.83808600	C	4.85257100	-9.02880000	0.62990700
H	2.58774500	-9.90248800	-0.91451300	H	3.76216400	-9.25676000	-1.19452000
C	2.55457800	-8.91642300	2.81545000	C	3.53875800	-8.86862600	2.63794000
H	0.42882200	-8.78256400	2.61106500	H	1.41912700	-8.98478200	2.38253900
C	3.76767400	-9.18870800	2.18324100	C	4.78764100	-8.87113800	2.01457300
H	4.70710100	-9.76300200	0.34433700	H	5.82047600	-9.03024000	0.14213300
H	2.55084000	-8.64097400	3.86405500	H	3.48014700	-8.75646900	3.71452200
C	-8.19173500	6.93740000	-0.04095800	C	-9.02975800	5.81231800	0.09579400
C	-7.00081200	6.26906000	-0.36883200	C	-7.77465800	5.32286800	-0.31669000
C	-8.06041800	8.20761800	0.54321000	C	-9.02284200	7.10398500	0.65762700
C	-5.75720700	6.84149900	-0.15199300	C	-6.60867600	6.05823600	-0.18041800
H	-7.05279900	5.28121300	-0.81487400	H	-7.71836100	4.33876700	-0.77110000
C	-6.82085100	8.77131200	0.79482900	C	-7.86079800	7.83775300	0.81782300
H	-8.95307100	8.76425600	0.80870300	H	-9.96289400	7.54059400	0.97943300
C	-5.64227900	8.10405500	0.44073900	C	-6.62179400	7.33404500	0.39860600
H	-4.85977200	6.29266900	-0.41711300	H	-5.66594000	5.62930200	-0.50551900
H	-6.76170400	9.75996500	1.23696200	H	-7.91454800	8.83402600	1.24485200
C	-4.32710700	8.72618400	0.67928800	C	-5.39261500	8.12835900	0.55468600
C	-3.27513500	8.58807700	-0.23174000	C	-4.35772200	8.08200200	-0.38793300
C	-4.09071300	9.49962900	1.82020900	C	-5.21847600	8.98314600	1.65047000
C	-2.05566700	9.21400800	-0.03344300	C	-3.22363300	8.86766800	-0.26423400
H	-3.42796800	8.01892400	-1.14230200	H	-4.46484300	7.45456000	-1.26599100
C	-2.86940700	10.10939100	2.04124700	C	-4.08179200	9.75701800	1.79438800
H	-4.86848000	9.59986900	2.56928800	H	-5.97646200	9.01821400	2.42500100
C	-1.83272700	9.99125000	1.10836000	C	-3.07010900	9.72499500	0.82944900
H	-1.27569600	9.11791900	-0.77906100	H	-2.46029600	8.83624800	-1.03291800
H	-2.71223900	10.68376300	2.94594200	H	-3.97139100	10.39554500	2.66274400
C	5.91768600	-8.10640300	2.40418200	C	6.80058700	-7.58869200	2.37230200
C	7.26053600	-8.42624900	2.19175500	C	8.17314900	-7.75099100	2.17508100
C	5.48149200	-6.81305300	2.11378900	C	6.23414900	-6.33141100	2.15906200
C	8.13971400	-7.47148600	1.70518300	C	8.95279300	-6.67955100	1.76773600
H	7.61197300	-9.42738400	2.41384000	H	8.62557100	-8.72173500	2.34285300
C	6.36555500	-5.86602800	1.62505600	C	7.01895000	-5.26783500	1.74746300
H	4.43950900	-6.55655600	2.26590200	H	5.16892600	-6.19612100	2.30644400
C	7.71223700	-6.17266100	1.41115900	C	8.39313800	-5.41792700	1.53984900
H	9.18154200	-7.73594400	1.55899200	H	10.01900400	-6.82325400	1.62955100
H	6.00059100	-4.87262700	1.38787600	H	6.55373300	-4.30559100	1.56357800
C	8.63739700	-5.15327800	0.88017100	C	9.20659400	-4.27917000	1.07315800
C	8.61248200	-3.83936200	1.35676100	C	9.01644200	-2.99456700	1.59122600
C	9.55254700	-5.45399700	-0.13186200	C	10.17034800	-4.43408500	0.07331900

C	9.45706700	-2.86787900	0.84804000
H	7.94059100	-3.57934900	2.16777500
C	10.37956800	-4.48252200	-0.67080900
H	9.58619800	-6.45863000	-0.53982000
C	10.34215900	-3.16876700	-0.19235500
H	9.43133600	-1.86637800	1.25969200
H	11.05159200	-4.73892900	-1.48072900
C	0.61005600	10.12975700	0.86785300
C	1.59300500	10.97953300	0.35363900
C	0.89250800	8.76332100	0.94968300
C	2.82331500	10.48118400	-0.03615200
H	1.39576200	12.04221700	0.28011400
C	2.11980900	8.27202900	0.53408200
H	0.14625800	8.08293300	1.34260100
C	3.12157900	9.11662500	0.04347900
H	3.57613200	11.17546500	-0.39212500
H	2.30051700	7.20441000	0.59392400
C	4.45270600	8.61134400	-0.34324500
C	5.04680300	7.53179900	0.31834900
C	5.19346200	9.22263900	-1.36165900
C	6.32916100	7.10704500	0.01097800
H	4.51833500	7.04296000	1.12948800
C	6.47087200	8.80091600	-1.68392900
H	4.75212500	10.03121800	-1.93373600
C	7.07052200	7.74869100	-0.98483500
H	6.77459400	6.29139600	0.56812200
H	7.01542500	9.29047600	-2.48259900
C	10.76993000	-0.84201400	-0.84208100
C	11.69507200	0.19617000	-0.69707300
C	9.43627300	-0.50263600	-1.09203600
C	11.29589900	1.51767700	-0.79344700
H	12.73725300	-0.03541500	-0.51425500
C	9.04365600	0.82347000	-1.15387600
H	8.69776900	-1.28420200	-1.22183000
C	9.95905400	1.87147300	-1.00659100
H	12.05080500	2.29060200	-0.70555200
H	7.99326900	1.04221500	-1.30924700
C	9.53924500	3.28527800	-1.06616400
C	8.41828300	3.69027300	-1.80074300
C	10.25171400	4.27980200	-0.38529000
C	8.03276300	5.01942900	-1.86339700
H	7.85482700	2.95974700	-2.37004100
C	9.87586400	5.61036100	-0.44387700
H	11.09992700	4.00757900	0.23227400
C	8.76245300	6.00009000	-1.18992400
H	7.17234500	5.30841400	-2.45604200
H	10.44085700	6.35824800	0.10029900
C	-12.28877000	-4.18390800	0.90785800
C	-12.88892200	-3.62762200	2.03987900
C	-12.91554500	-5.25085300	0.26002600
C	-14.09743100	-4.12540700	2.50540400
H	-12.40299800	-2.80494300	2.55122400
C	-14.11448600	-5.75429100	0.74408000
H	-12.45811200	-5.68196300	-0.62273300
C	-14.71548000	-5.19331500	1.86503100
H	-14.55060500	-3.68206500	3.38526000
H	-14.58835400	-6.58326000	0.22994900
H	-15.65587800	-5.58445500	2.23589400
C	-10.79380600	7.23661200	-0.72381300
C	-12.11151700	6.93246800	-0.34473400
C	-10.59583600	8.40610100	-1.47595300
C	-13.17469800	7.75806400	-0.68481800
H	-12.30144800	6.03846000	0.23991700
C	-11.65716100	9.22168200	-1.84466400
H	-9.59104700	8.67124300	-1.78782700
C	-12.94929200	8.90185200	-1.44267700
H	-14.18047600	7.50795800	-0.36468300
H	-11.47744700	10.11044400	-2.43997800
H	-13.77939800	9.54306900	-1.71946600
C	-0.61475900	11.91181900	1.97718000
C	-1.49922500	12.91866500	1.58655200
C	0.27742400	12.15336600	3.02305200
C	-1.49549000	14.14227400	2.24126000
H	-2.18765800	12.73550100	0.76980400
C	0.28660500	13.38570000	3.66110700
H	0.96192000	11.37108500	3.32981200
C	-0.60135000	14.38439400	3.27791700
H	-2.18781800	14.91579200	1.92773900
H	0.98506200	13.56000200	4.47210200
H	-0.59634500	15.34356300	3.78283200
C	9.38404100	8.32746600	-1.52780500
C	9.39728200	9.54656200	-0.84378200
C	10.38249100	8.07032000	-2.47176900
C	10.38151500	10.48727900	-1.11078200
H	8.63524100	9.75205200	-0.10160100
C	11.37206100	9.01059700	-2.71851600
H	10.37670000	7.13025500	-3.01066000
C	11.37712500	10.22672300	-2.04520600
H	10.37594700	11.42704100	-0.56936300
H	12.13796000	8.79299300	-3.45490300
H	12.14787200	10.96199400	-2.24548700
C	12.47149300	-2.54758900	-1.19964200
C	13.32087900	-3.29805000	-0.38517000
C	12.90658200	-2.15892300	-2.46760500
C	14.58139800	-3.66013700	-0.83895800
H	12.98625200	-3.59526800	0.60193800
C	14.17517300	-2.51006600	-2.90717100

C	9.74630400	-1.91244200	1.13079100
H	8.30406200	-2.84352300	2.39522900
C	10.88434100	-3.35340800	-0.41648000
H	10.33115800	-5.41240800	-0.36696600
C	10.67843300	-2.06993300	0.09979400
H	9.59320000	-0.93504700	1.57186900
H	11.59672300	-3.50015700	-1.21915000
C	-0.65989400	10.14556400	0.58337600
C	0.25000500	11.06099100	0.04377800
C	-0.23880400	8.82274500	0.75720900
C	1.53895100	10.67340000	-0.27266700
H	-0.05261200	12.08986400	-0.10793300
C	1.04866500	8.44161000	0.41539800
H	-0.92447300	8.09050700	1.16637900
C	1.97701900	9.35646200	-0.09380000
H	2.22761100	11.42158700	-0.64895400
H	1.33516800	7.40418400	0.54964300
C	3.37052200	8.97960500	-0.39697900
C	4.04618600	8.00568400	0.34617600
C	4.09020000	9.62081000	-1.41261800
C	5.38221600	7.71550100	0.12083100
H	3.53466300	7.49953400	1.15749700
C	5.42192200	9.33345700	-1.65173500
H	3.59212100	10.34571800	-2.04672500
C	6.09796900	8.39204500	-0.87009000
H	5.88690000	6.98322800	0.74013700
H	5.95044500	9.84454300	-2.44779800
C	10.81596100	0.30613000	-0.49610400
C	11.59765100	1.45208100	-0.32194900
C	9.45509000	0.47529400	-0.77096400
C	11.03685300	2.71291700	-0.42392400
H	12.65642300	1.35289000	-0.11591800
C	8.89924500	1.74163500	-0.83744900
H	8.82440600	-0.39242200	-0.92059300
C	9.67165000	2.89629600	-0.67040100
H	11.68548800	3.57461500	-0.31645700
H	7.83340600	1.82732500	-1.01664600
C	9.08172900	4.24659700	-0.75313600
C	7.93345500	4.50246600	-1.51207200
C	9.65657000	5.33055900	-0.07872400
C	7.39440300	5.77487400	-1.60843000
H	7.47106600	3.69998700	-2.07560300
C	9.12714900	6.60558500	-0.17019700
H	10.51956800	5.17217100	0.55775800
C	7.99152300	6.84864900	-0.94556200
H	6.51759800	5.94828600	-2.22165300
H	9.58981400	7.42433400	0.36839400
C	-11.57394200	-5.77198000	1.26292700
C	-12.15322800	-5.24399100	2.42130200
C	-12.08291200	-6.94904800	0.70333700
C	-13.21997800	-5.89786000	3.01531500
H	-11.75272600	-4.33480400	2.85223700
C	-13.14764900	-7.59456600	1.31027400
H	-11.65533900	-7.33656800	-0.21355200
C	-13.72058300	-7.07439600	2.46660200
H	-13.65823100	-5.48857200	3.91803000
H	-13.54334900	-8.50092400	0.86682900
H	-14.55673700	-7.58037100	2.93469200
C	-11.68238800	5.80307400	-0.39431700
C	-12.92955400	5.43546700	0.14273800
C	-11.67316900	6.93964700	-1.22350400
C	-14.08979900	6.14847000	-0.12989300
H	-12.98371100	4.57558100	0.80299000
C	-12.82963400	7.65094000	-1.51430100
H	-10.73238700	7.26485100	-1.65672900
C	-14.04685400	7.25898100	-0.96643400
H	-15.03129300	5.83911600	0.31321600
H	-12.78271600	8.51525400	-2.16938100
H	-14.95185300	7.81584000	-1.18585800
C	-2.12797600	11.87160600	1.46999900
C	-3.15382400	12.68411700	0.98308400
C	-1.28702600	12.36071500	2.47177100
C	-3.33642100	13.96012800	1.49667800
H	-3.80566600	12.30809300	0.20327900
C	-1.46603900	13.64417600	2.96776300
H	-0.49489800	11.72934200	2.85704900
C	-2.49253600	14.44941800	2.48725700
H	-4.13757100	14.57966700	1.10897900
H	-0.80571100	14.01047100	3.74615200
H	-2.63410500	15.44896100	2.88211300
C	8.35352600	9.22976500	-1.29314300
C	8.18248900	10.44988400	-0.63270100
C	9.42274800	9.08523000	-2.18201700
C	9.05740200	11.50069600	-0.86771000
H	7.36301600	10.56964600	0.06581800
C	10.30196200	10.13650700	-2.39654800
H	9.55866000	8.14512500	-2.70345500
C	10.12434200	11.35226900	-1.74627700
H	8.90907200	12.43974900	-0.34569600
H	11.12506600	10.00558400	-3.09055800
H	10.80910800	12.17388400	-1.92197800
C	12.72650900	-1.16191300	-0.85465500
C	13.65357500	-1.82314000	-0.04754400
C	13.12331400	-0.69044000	-2.10702300
C	14.95370500	-2.01568800	-0.49314400
H	13.34813900	-2.18404200	0.92773400
C	14.43000200	-0.87136800	-2.53790600

H	12.24564000	-1.58077300	-3.10291500	H	12.40297700	-0.18155400	-2.73695600
C	15.01707300	-3.26529100	-2.09870000	C	15.35033700	-1.53781800	-1.73691100
H	15.23129300	-4.24378600	-0.19635300	H	15.66413300	-2.53069400	0.14404800
H	14.50095300	-2.20154600	-3.89441700	H	14.72545700	-0.49950400	-3.51276900
H	16.00480300	-3.54399100	-2.44763900	H	16.36856800	-1.68312300	-2.07911700
C	-3.51452500	-10.21210800	-3.50774100	C	-2.06011000	-9.57862900	-4.15873100
C	-2.49629000	-10.04653300	-4.44823600	C	-1.03992100	-8.99674400	-4.91161100
C	-4.58242800	-11.06215500	-3.79908900	C	-2.92862800	-10.49055000	-4.75857500
C	-2.54353800	-10.73230600	-5.65394000	C	-0.89039800	-9.33236200	-6.24960400
H	-1.67038200	-9.38005900	-4.22872500	H	-0.37107000	-8.28282500	-4.44557800
C	-4.63121500	-11.73057300	-5.01416500	C	-2.78010100	-10.81000900	-6.10071400
H	-5.37168400	-11.19234800	-3.06771200	H	-3.71402100	-10.94672300	-4.16711100
C	-3.61102300	-11.57389900	-5.94542600	C	-1.75964300	-10.23622400	-6.85037100
H	-1.74592900	-10.59503100	-6.37574200	H	-0.09637800	-8.87401200	-6.82817900
H	-5.46699200	-12.38770600	-5.22762300	H	-3.45864000	-11.52111500	-6.55809500
H	-3.64764900	-12.10288900	-6.89084200	H	-1.64191800	-10.49192400	-7.89702700
C	5.24842400	-9.83057800	4.02453600	C	6.27605500	-9.46657900	3.86847000
C	4.60370000	-11.05960600	4.21324600	C	5.80243600	-10.78172300	3.95007400
C	6.13709900	-9.38357300	5.01051800	C	7.04958600	-8.96565100	4.92220300
C	4.84169900	-11.81025700	5.35483300	C	6.09106000	-11.56421800	5.05801500
H	3.91797200	-11.42594600	3.45905000	H	5.21151200	-11.18929500	3.13874000
C	6.37677700	-10.15204300	6.13995800	C	7.34395400	-9.76470900	6.01713400
H	6.63534700	-8.42928900	4.89201200	H	7.41511000	-7.94685900	4.88294600
C	5.73180200	-11.36894800	6.32666600	C	6.86645100	-11.06731600	6.09907900
H	4.32988800	-12.75892500	5.47654300	H	5.71351900	-12.58032000	5.09801300
H	7.06910100	-9.78292400	6.88911100	H	7.94468700	-9.35411000	6.82156300
H	5.91899900	-11.96217500	7.21411300	H	7.09508400	-11.68495100	6.95975700
N	-0.61602100	10.65864600	1.31621600	N	-1.94705700	10.56488500	0.95679800
N	8.39130000	7.36015400	-1.26896600	N	7.47087000	8.15368300	-1.06620100
N	11.18062400	-2.18355800	-0.74440400	N	11.39623400	-0.97239400	-0.40681200
N	4.99863500	-9.06910300	2.87739300	N	5.97817200	-8.67411700	2.75250300
N	-3.48583400	-9.51647100	-2.27436100	N	-2.22654100	-9.24604200	-2.78880500
N	-11.06266300	-3.67423000	0.42584900	N	-10.50105600	-5.09809500	0.64733300

Structure S52. The coordinates of both the optimized structures of N7L$_2$ at S$_0$ and S$_1$ in toluene.

S$_0$-optimized structure				S$_1$-optimized structure			
C	-9.83445700	-4.81985500	0.01879800	C	-9.81156400	-4.84909500	0.28566000
C	-9.01445600	-4.49103300	-1.06441900	C	-9.03828800	-4.51328200	-0.82935900
C	-9.57811400	-6.01040900	0.70540700	C	-9.53301600	-6.04929600	0.94664900
C	-7.96044100	-5.31142900	-1.42498700	C	-8.00738300	-5.33635800	-1.24599300
H	-9.19540100	-3.57593600	-1.61519400	H	-9.23694200	-3.59101100	-1.36165900
C	-8.53474200	-6.83580700	0.32034000	C	-8.51440200	-6.87729700	0.50534500
H	-10.20358300	-6.29047500	1.54453200	H	-10.12213100	-6.33489900	1.80990200
C	-7.69149200	-6.50407600	-0.74511500	C	-7.71704700	-6.53898900	-0.59305600
H	-7.32219800	-5.00572300	-2.24641300	H	-7.40444400	-5.02470300	-2.09146400
H	-8.37673600	-7.76169000	0.86242200	H	-8.33969200	-7.81066800	1.02916900
C	-6.54698800	-7.35283800	-1.12831800	C	-6.59694300	-7.39075600	-1.03651000
C	-6.11528100	-7.43156100	-2.45633100	C	-6.21885700	-7.45007100	-2.38187200
C	-5.82834300	-8.08486400	-0.17670700	C	-5.84758400	-8.14541800	-0.12712500
C	-4.99748600	-8.16576400	-2.81322400	C	-5.12175100	-8.18575000	-2.79538000
H	-6.66227700	-6.90665200	-3.23137200	H	-6.79175400	-6.90819800	-3.12586900
C	-4.72407600	-8.84359400	-0.52777500	C	-4.76458400	-8.90630600	-0.53481400
H	-6.12425000	-8.04643100	0.86574700	H	-6.10109000	-8.12305500	0.92690000
C	-4.27583400	-8.87465100	-1.85054800	C	-4.36812100	-8.91636800	-1.87438900
H	-4.67700400	-8.19064400	-3.84787100	H	-4.84235700	-8.19425100	-3.84215400
H	-4.18681900	-9.39771100	0.23292000	H	-4.20257600	-9.47795600	0.19435800
C	-10.71965800	-2.56829300	0.32024200	C	-10.67721100	-2.59660400	0.63037000
C	-9.52061500	-1.96212800	0.70357600	C	-9.46406000	-1.98860300	0.96379800
C	-11.75074500	-1.75806700	-0.16059500	C	-11.72580200	-1.78894300	0.18422600
C	-9.35349900	-0.59312000	0.58302200	C	-9.29994500	-0.62100900	0.82523500
H	-8.70958400	-2.57324400	1.08157400	H	-8.63994600	-2.59805200	1.31536300
C	-11.58148100	-0.38689400	-0.25584300	C	-11.55837000	-0.41932900	0.06952100
H	-12.69135300	-2.20784500	-0.45586000	H	-12.67766800	-2.23984700	-0.07043500
C	-10.37742900	0.22947500	0.10128100	C	-10.34007300	0.19867500	0.37309200
H	-8.39911400	-0.15722400	0.85694600	H	-8.33512700	-0.18444400	1.05860500
H	-12.40829600	0.21830700	-0.61084500	H	-12.39769000	0.18355100	-0.25908600
C	-10.19291200	1.68668700	-0.03235500	C	-10.15931300	1.65271100	0.21083700
C	-9.40201700	2.40926100	0.86712200	C	-9.32948500	2.38994200	1.06315400
C	-10.80032700	2.40229600	-1.06980400	C	-10.81155700	2.35212500	-0.81092300
C	-9.23306800	3.77833000	0.74752300	C	-9.16679700	3.75681100	0.91487600
H	-8.93948200	1.89808100	1.70434200	H	-8.83069500	1.89256800	1.88772400
C	-10.62816800	3.76860300	-1.20645800	C	-10.64709300	3.71547600	-0.97624700
H	-11.39044500	1.87427000	-1.81077300	H	-11.43259900	1.81271600	-1.51747600
C	-9.84553800	4.48154500	-0.29356100	C	-9.82623200	4.44329100	-0.10920600
H	-8.63484400	4.31353000	1.47537400	H	-8.53887500	4.30387100	1.60812300
H	-11.09520300	4.29124500	-2.03268600	H	-11.14974200	4.22471600	-1.78976600
C	-1.94547800	-9.52260900	-1.45363800	C	-2.02957400	-9.58526700	-1.57657200
C	-1.00206600	-10.55651200	-1.49434100	C	-1.09445000	-10.62317100	-1.67097400
C	-1.67356400	-8.41491400	-0.64222600	C	-1.72274000	-8.49543000	-0.75341400
C	0.17639600	-10.46705600	-0.77325400	C	0.10773700	-10.55591400	-0.98744200
H	-1.19499600	-11.43570700	-2.09657300	H	-1.31306100	-11.48854900	-2.28433400
C	-0.50883900	-8.35524100	0.10408100	C	-0.53416000	-8.45866900	-0.04443900
H	-2.37200100	-7.58855400	-0.60493500	H	-2.41377800	-7.66565800	-0.67631100
C	0.44920700	-9.36985300	0.04752600	C	0.41390200	-9.47840600	-0.15261000
H	0.88652600	-11.28588300	-0.82246500	H	0.81042500	-11.37773600	-1.07694300
H	-0.31850300	-7.47568800	0.71004600	H	-0.31801900	-7.59341500	0.57335900
C	1.71186200	-9.26940300	0.80582200	C	1.69801000	-9.40375700	0.57167000
C	2.93865300	-9.51656100	0.18329400	C	2.90632900	-9.63935200	-0.09018200
C	1.72104600	-8.90453700	2.15592300	C	1.74430900	-9.07598400	1.93043700
C	4.13128800	-9.38893700	0.87723700	C	4.11760000	-9.53575800	0.57515600
H	2.95769900	-9.78949100	-0.86621200	H	2.89584200	-9.88361600	-1.14686500
C	2.91058800	-8.78051000	2.85736600	C	2.95245100	-8.97758100	2.60338800
H	0.78043400	-8.72268600	2.66460400	H	0.81808000	-8.90362800	2.46797300
C	4.12903300	-9.01372500	2.21977000	C	4.15208100	-9.19856400	1.92719700
H	5.07752400	-9.56765400	0.37928200	H	5.04972600	-9.70481200	0.04805900
H	2.90291300	-8.49796800	3.90405600	H	2.97428800	-8.72478800	3.65746800
C	-8.44253000	6.47581100	-0.13460500	C	-8.44242500	6.46154500	-0.05476600
C	-7.24806900	5.90443100	-0.58035100	C	-7.24501900	5.86349600	-0.46512100
C	-8.38158600	7.66737600	0.59156600	C	-8.37796600	7.71902700	0.55646400
C	-6.03271800	6.51030800	-0.30523300	C	-6.03489600	6.50663000	-0.27742400
H	-7.27557700	4.97818400	-1.14230100	H	-7.26766200	4.88747400	-0.93531100
C	-7.16358000	8.27316100	0.84737600	C	-7.16582600	8.35866600	0.72706700
H	-9.29806200	8.12556100	0.94410300	H	-9.29127200	8.20116600	0.88400900
C	-5.96002400	7.71115600	0.40816300	C	-5.95689000	7.77681400	0.31440800
H	-5.11983700	6.03478400	-0.64714300	H	-5.12721000	6.00379700	-0.59194800
H	-7.14897700	9.21272800	1.38862200	H	-7.16141300	9.34746800	1.17114800
C	-4.67167200	8.37510800	0.68201700	C	-4.68474400	8.48738100	0.47453700
C	-3.63300600	8.37635900	-0.25442800	C	-3.61008300	8.29392100	-0.40982500
C	-4.44959100	9.05126900	1.88597700	C	-4.49789500	9.42564300	1.50458200
C	-2.44190000	9.04333900	-0.01793100	C	-2.43601800	9.01239800	-0.30085600
H	-3.77546600	7.88533800	-1.21107900	H	-3.71681800	7.60290400	-1.23839000
C	-3.25742400	9.70484400	2.14101700	C	-3.32987200	10.14633300	1.62912900
H	-5.21491200	9.04029800	2.65437300	H	-5.27587000	9.56986200	2.24549600
C	-2.23683600	9.72628000	1.18485100	C	-2.28064900	9.97278900	0.71139700
H	-1.67160900	9.05498300	-0.77983400	H	-1.64333700	8.86813000	-1.02511200
H	-3.11066400	10.20472700	3.09079000	H	-3.21344300	10.84620300	2.44767000
C	6.24080600	-7.85408600	2.41534800	C	6.26585100	-8.04051400	2.12315300
C	7.59358400	-8.12795300	2.20163700	C	7.62208700	-8.28949300	1.89829900
C	5.75725900	-6.58099400	2.11060800	C	5.76587600	-6.76432400	1.85842700
C	8.43622900	-7.14832200	1.70001900	C	8.45174100	-7.28281200	1.43117300
H	7.98165700	-9.11292800	2.43474200	H	8.02361700	-9.27639600	2.09794800
C	6.60505300	-5.60899300	1.60693300	C	6.60109400	-5.76509200	1.38876700
H	4.70730700	-6.36000200	2.26355500	H	4.71331200	-6.56124600	2.01786500
C	7.96124800	-5.86940200	1.39206900	C	7.96164700	-5.99892000	1.16814000
H	9.48637200	-7.37702100	1.55305000	H	9.50501800	-7.49311500	1.27928300
H	6.20400700	-4.63225700	1.35905400	H	6.18651600	-4.78622200	1.17380000
C	8.84694300	-4.82280100	0.84703600	C	8.83906600	-4.92283100	0.67327900
C	8.77489000	-3.50596700	1.31053100	C	8.73179900	-3.61987300	1.17029700
C	9.76932200	-5.09974200	-0.16521700	C	9.79916400	-5.15664900	-0.31598200
C	9.58117900	-2.50913400	0.78901700	C	9.54292400	-2.59846100	0.70933900

H	8.09644900	-3.26285300	2.12134600
C	10.55771000	-4.10371000	-0.71721800
H	9.83889200	-6.10654500	-0.56321700
C	10.47314300	-2.78744700	-0.25181700
H	9.51957700	-1.50514800	1.19066400
H	11.23591200	-4.34315600	-1.52719300
C	0.19527400	9.98570200	0.95690600
C	1.14290500	10.89924800	0.48709200
C	0.53096200	8.62878900	0.96574600
C	2.39123000	10.47050700	0.07235500
H	0.90366100	11.95570400	0.46826000
C	1.77637800	8.20857000	0.52685200
H	-0.18832200	7.90025100	1.32081200
C	2.74348500	9.11667200	0.08261600
H	3.11607800	11.21063300	-0.24745000
H	1.99942300	7.14725400	0.53107100
C	4.09424300	8.68568400	-0.32497400
C	4.73744700	7.61115000	0.29744900
C	4.80445400	9.36527300	-1.32152200
C	6.03685400	7.25543400	-0.02533200
H	4.23285200	7.07138400	1.09141400
C	6.09809200	9.01144400	-1.66076200
H	4.32656000	10.17444400	-1.86290700
C	6.74610800	7.96298400	-0.99983800
H	6.51923200	6.44172100	0.50307000
H	6.61753300	9.55209500	-2.44301300
C	10.80949900	-0.45196600	-0.91993500
C	11.69264000	0.62169300	-0.77267100
C	9.46453400	-0.16595500	-1.17543400
C	11.24174300	1.92645000	-0.86994500
H	12.74242900	0.43107500	-0.58597500
C	9.01986900	1.14352500	-1.23869600
H	8.75822000	-0.97647400	-1.30711300
C	9.89252300	2.22682200	-1.08703800
H	11.96481100	2.72874000	-0.77793400
H	7.96223400	1.32076400	-1.39782400
C	9.41527700	3.62250200	-1.14171600
C	8.27751800	3.98376400	-1.87329500
C	10.08502600	4.64216100	-0.45486300
C	7.83324300	5.29491200	-1.92404700
H	7.74614300	3.23331600	-2.44750900
C	9.64994600	5.95507300	-0.50095800
H	10.94561600	4.40249600	0.15901500
C	8.51795500	6.30083800	-1.24058700
H	6.95962900	5.55058700	-2.51268100
H	10.18157900	6.72238900	0.04971300
C	-12.10264200	-4.49964200	0.87603000
C	-12.75278400	-3.92116900	1.96917000
C	-12.68096900	-5.60714500	0.25042300
C	-13.95936300	-4.43768400	2.41867800
H	-12.30645200	-3.06616100	2.46319100
C	-13.87863500	-6.12780100	0.71895500
H	-12.18772200	-6.05596900	-0.60368900
C	-14.52818300	-5.54575400	1.80144700
H	-14.45031900	-3.97595400	3.26833900
H	-14.31356300	-6.98786400	0.22164000
H	-15.46743500	-5.95102000	2.15981500
C	-10.77638900	6.67870900	-0.80932900
C	-12.05563300	6.43909200	-0.30048700
C	-10.59041900	7.73134300	-1.70931300
C	-13.12427000	7.23129900	-0.69458800
H	-12.20666200	5.63067400	0.40507100
C	-11.66202700	8.52920000	-2.08333900
H	-9.60252700	7.91910500	-2.11311200
C	-12.93572100	8.28309600	-1.58377600
H	-14.11021600	7.03142600	-0.28938700
H	-11.49940100	9.34164100	-2.78311000
H	-13.77191100	8.90417700	-1.88363600
C	-1.10894700	11.65266200	2.15637500
C	-2.06642700	12.61920300	1.84212500
C	-0.20675300	11.89824900	3.19333500
C	-2.12362600	13.80455000	2.56132600
H	-2.76360900	12.43489900	1.03314500
C	-0.25955400	13.09381300	3.89579500
H	0.53447200	11.14803700	3.44285700
C	-1.21953100	14.05118400	3.58820800
H	-2.87274200	14.54591200	2.30591300
H	0.44780200	13.27048700	4.69857900
H	-1.26235900	14.98109000	4.14356000
C	9.02839000	8.65952800	-1.54025600
C	8.99306900	9.86072800	-0.82616300
C	10.03104700	8.46916800	-2.49544000
C	9.93386900	10.84966400	-1.07495300
H	8.22763900	10.01466300	-0.07503100
C	10.97745000	9.45724400	-2.72399500
H	10.06228800	7.54300800	-3.05710300
C	10.93384300	10.65558000	-2.02082000
H	9.89119700	11.77482600	-0.51052700
H	11.74744500	9.29130500	-3.46952400
H	11.67051800	11.42852700	-2.20701300
C	12.57332400	-2.09366100	-1.27479700
C	13.45191600	-2.81174900	-0.46197800
C	12.99117900	-1.68959300	-2.54382200
C	14.72398200	-3.12692200	-0.91847200
H	13.13091100	-3.12086600	0.52598500
C	14.27102800	-1.99338500	-2.98595200
H	12.30787300	-1.13680100	-3.17805200

H	8.02332400	-3.40889300	1.96416000
C	10.59543800	-4.13666200	-0.80719400
H	9.89509700	-6.14946400	-0.74255300
C	10.48203100	-2.83628300	-0.30103800
H	9.45538200	-1.60838500	1.13940500
H	11.30412600	-4.34373100	-1.59970700
C	0.13516500	10.21712300	0.47174400
C	1.08458300	11.01659300	-0.19938400
C	0.50546700	8.89707700	0.80658400
C	2.33529000	10.53669600	-0.48782000
H	0.81768500	12.02678600	-0.48758300
C	1.76352900	8.42597400	0.52380800
H	-0.20685900	8.25682000	1.31435100
C	2.75206200	9.22406100	-0.12157500
H	3.02848100	11.20334200	-0.98492700
H	1.99701800	7.40739000	0.80848300
C	4.09203700	8.76585400	-0.35084200
C	4.61355300	7.58534700	0.25398000
C	5.01037400	9.49318400	-1.16429500
C	5.92449200	7.20527600	0.11735600
H	3.98108800	6.98743400	0.89839000
C	6.31941800	9.11768100	-1.30987000
H	4.66948300	10.35870900	-1.71792000
C	6.82692400	7.97738500	-0.64846800
H	6.28605300	6.32603600	0.63775900
H	6.97558300	9.69522400	-1.95073500
C	10.83979000	-0.47207900	-0.82497900
C	11.71147200	0.58355900	-0.52794400
C	9.51396700	-0.15625800	-1.15177000
C	11.27020300	1.89072200	-0.54417800
H	12.74550300	0.37074700	-0.28445600
C	9.07663200	1.15363100	-1.14547100
H	8.81956600	-0.95056300	-1.39831400
C	9.93481100	2.22532100	-0.83640400
H	11.98934000	2.67304700	-0.33305200
H	8.03368300	1.34418000	-1.36998300
C	9.47200900	3.61057100	-0.81226700
C	8.30399500	4.02548100	-1.48384500
C	10.17749600	4.60685000	-0.10653300
C	7.87094700	5.33388800	-1.46114000
H	7.74250000	3.31280100	-2.07626900
C	9.76183400	5.91807100	-0.08131500
H	11.05237600	4.33826300	0.47293100
C	8.60049000	6.31543200	-0.76785100
H	6.98214400	5.62108100	-2.01044900
H	10.31695000	6.64858000	0.49484200
C	-12.03962300	-4.52510400	1.24184100
C	-12.64511700	-3.94790700	2.36100500
C	-12.64687400	-5.62697100	0.63398200
C	-13.83649800	-4.46023200	2.85365200
H	-12.17604500	-3.09716600	2.84114900
C	-13.82874700	-6.14353300	1.14526300
H	-12.18878800	-6.07459200	-0.24009000
C	-14.43393800	-5.56283300	2.25382300
H	-14.29273900	-3.99968500	3.72307400
H	-14.28664700	-6.99916900	0.66110400
H	-15.36102700	-5.96498600	2.64578900
C	-10.80397600	6.61153500	-0.63016300
C	-12.04155700	6.40063000	-0.01767400
C	-10.68756500	7.60117200	-1.60905000
C	-13.14139300	7.16141600	-0.38707400
H	-12.13450100	5.63945800	0.74806700
C	-11.78900400	8.36838900	-1.95979400
H	-9.73094200	7.76323800	-2.09173600
C	-13.02224100	8.15171000	-1.35554900
H	-14.09543000	6.98654500	0.09803300
H	-11.68307600	9.13230200	-2.72223100
H	-13.88214000	8.74876100	-1.63670000
C	-1.19943100	12.07682100	1.25758800
C	-2.19995800	12.92423400	0.77328400
C	-0.26571500	12.56694300	2.17448700
C	-2.26749000	14.23777600	1.21184100
H	-2.91332800	12.54868400	0.04949000
C	-0.33798600	13.88441500	2.60003000
H	0.50592300	11.90639400	2.55124400
C	-1.33820300	14.72596000	2.12445300
H	-3.04482400	14.88799300	0.82628000
H	0.38744000	14.25223400	3.31701600
H	-1.39185400	15.75490100	2.46095700
C	9.14453700	8.67666300	-0.75491000
C	9.01795000	9.77485300	0.09990600
C	10.22679000	8.60811900	-1.63650800
C	9.96397800	10.78773900	0.06891700
H	8.18282600	9.81976700	0.78839200
C	11.17020400	9.62371000	-1.65400400
H	10.31373100	7.76287500	-2.30866800
C	11.04399700	10.71823800	-0.80490600
H	9.86139000	11.63194700	0.74141600
H	12.00310000	9.56459200	-2.34559400
H	11.78156800	11.51225000	-0.82498200
C	12.60887400	-2.08767200	-1.23001800
C	13.46020600	-2.87201200	-0.44905600
C	13.07133000	-1.57591900	-2.44394200
C	14.74880100	-3.14603500	-0.88451600
H	13.10639100	-3.26236000	0.49796100
C	14.36646700	-1.84199800	-2.86412500
H	12.40921700	-0.97096900	-3.05237300

C	15.14218200	-2.71655100	-2.17917900	C	15.21061600	-2.63078900	-2.09047900
H	15.39646200	-3.68565700	-0.27695100	H	15.40007300	-3.75578100	-0.26806600
H	14.58296600	-1.67324100	-3.97397000	H	14.71244300	-1.43955600	-3.80975900
H	16.13889900	-2.95833500	-2.53005500	H	16.22014700	-2.84166200	-2.42416900
C	-3.12968000	-10.29812300	-3.44716400	C	-3.29205000	-10.31703700	-3.53879000
C	-2.11277100	-10.11144800	-4.38526600	C	-2.30915900	-10.12079100	-4.51057600
C	-4.17768300	-11.17202600	-3.74047200	C	-4.35664500	-11.17852500	-3.80814900
C	-2.14131400	-10.80014300	-5.58989700	C	-2.38742100	-10.78790000	-5.72511400
H	-1.30250600	-9.42633500	-4.16491900	H	-1.48633000	-9.44504700	-4.30860600
C	-4.20805600	-11.84343300	-4.95447000	C	-4.43697100	-11.82810500	-5.03168800
H	-4.96615700	-11.31831900	-3.01131100	H	-5.11860800	-11.33236500	-3.05282000
C	-3.18884300	-11.66587600	-5.88304400	C	-3.45152600	-11.64113400	-5.99424400
H	-1.34489400	-10.64625200	-6.30966700	H	-1.61699700	-10.62675400	-6.47110300
H	-5.02852400	-12.51921000	-5.16911500	H	-5.26982000	-12.49440200	-5.22747200
H	-3.21090100	-12.19722100	-6.82759000	H	-3.51258800	-12.15535800	-6.94651900
C	5.64065000	-9.57891700	4.06134500	C	5.70574600	-9.83706800	3.70597000
C	5.04513700	-10.82968900	4.26786500	C	5.12307200	-11.10056300	3.86585000
C	6.51305700	-9.08415700	5.03894100	C	6.58720400	-9.37737800	4.69244400
C	5.31473800	-11.55512600	5.41867500	C	5.41411900	-11.87295500	4.98030200
H	4.37282900	-11.23247700	3.52015900	H	4.44366100	-11.47598200	3.11033600
C	6.78493700	-9.82740300	6.17793400	C	6.88067500	-10.16693600	5.79431000
H	6.97324400	-8.11274300	4.90637900	H	7.03717100	-8.39695800	4.59632500
C	6.18867900	-11.06612000	6.38234700	C	6.29737600	-11.41856300	5.95239100
H	4.84090600	-12.52149300	5.55420100	H	4.94991000	-12.84826000	5.08030100
H	7.46372900	-9.42152100	6.92043200	H	7.56590700	-9.78762300	6.54488300
H	6.40081600	-11.63964800	7.27711100	H	6.52641400	-12.02846000	6.81848500
N	-1.05058100	10.43867400	1.43106200	N	-1.11606900	10.73819300	0.81049900
N	8.08124400	7.64243300	-1.30050500	N	8.17786000	7.64555600	-0.73688300
N	11.27123200	-1.77672900	-0.81723900	N	11.29271500	-1.80180200	-0.79544700
N	5.35910500	-8.84337400	2.90457500	N	5.40027800	-9.05517100	2.58565700
N	-3.12163700	-9.59989000	-2.21514100	N	-3.23312500	-9.64094200	-2.29595600
N	-10.88211800	-3.96639400	0.41017000	N	-10.83535100	-3.99431900	0.73273700
N	-9.68538600	5.87335400	-0.41825900	N	-9.68242800	5.83366100	-0.26263400

Structure S53. The coordinates of both the optimized structures of B4L$_3$ at S_0 and S_1 in toluene.

S_0-optimized structure				S_1-optimized structure			
B	-3.77352200	-5.96896800	-0.00028200	B	-3.38089100	-6.19067400	-0.00103200
H	0.92272600	-4.09153100	-2.18798600	H	-3.89514300	-1.14388600	2.17133100
C	4.35545700	-5.64676300	0.81318200	C	-7.10765900	1.03940800	-0.68268500
C	4.57124500	-4.47714100	0.05956800	C	-6.13237900	1.82065900	-0.01530000
C	3.46556800	-3.91292900	-0.60254200	C	-5.06613300	1.13249000	0.60648200
C	2.21358100	-4.48593100	-0.49710000	C	-4.96743100	-0.23680700	0.52618000
C	2.01783200	-5.62738200	0.29751900	C	-5.92557200	-0.98683500	-0.19356600
C	3.09682400	-6.21686500	0.95099000	C	-7.01202800	-0.33531100	-0.79113100
H	5.19501200	-6.09964300	1.33000300	H	-7.94074300	1.54586000	-1.15795100
H	3.60035600	-3.01428700	-1.19587800	H	-4.31353400	1.69699000	1.14687300
H	2.96025200	-7.09870600	1.56795300	H	-7.75811000	-0.89552500	-1.34432200
C	0.90968700	-4.03491200	-1.09328100	C	-3.90475800	-1.14649500	1.07519300
H	0.67540100	-2.99566000	-0.83866400	H	-2.90036500	-0.85152600	0.75380500
C	-0.07493300	-5.00198100	-0.49786300	C	-4.30714700	-2.48599600	0.52568200
C	-1.45156200	-5.02178100	-0.60437300	C	-3.65013800	-3.69145100	0.60553100
C	-2.20874900	-6.00518000	0.05815800	C	-4.17535000	-4.84656300	-0.01659700
C	-1.51261500	-6.96861800	0.81304900	C	-5.41827700	-4.71608900	-0.68368500
C	-0.13108700	-6.94373500	0.95145900	C	-6.08078700	-3.50772700	-0.79161700
C	0.59107600	-5.94885700	0.29751600	C	-5.51876500	-2.37254300	-0.19382200
H	-1.95847900	-4.26763700	-1.19778600	H	-2.71176000	-3.75978600	1.14577400
H	-2.07709600	-7.73756600	1.32992200	H	-5.84556700	-5.59220200	-1.15933200
H	0.37023300	-7.68161800	1.56880400	H	-7.01126400	-3.43958600	-1.34481900
B	5.96933700	-3.77377500	0.00068900	B	-6.19075200	3.38094600	0.00105400
H	-4.08787900	-0.92141500	2.18669000	H	1.14405700	-3.89566700	-2.17163400
C	-6.96972300	1.51255500	-0.81052400	C	4.71614400	-5.41812500	0.68390200
C	-6.00486300	2.20904500	-0.05778000	C	4.84667800	-4.17539500	0.01644700
C	-5.02040400	1.45213300	0.60345800	C	3.69158500	-3.65032000	-0.60583500
C	-5.00080800	0.07546900	0.49750600	C	2.48611500	-4.30727600	-0.52584000
C	-5.94907100	-0.59092700	-0.29585600	C	2.37261100	-5.51869800	0.19398900
C	-6.94509500	0.13097300	-0.94838600	C	3.50776900	-6.08058100	0.79197500
H	-7.73961400	2.07681600	-1.32626600	H	5.59221900	-5.84527400	1.15974100
H	-4.26530100	1.95920000	1.19547400	H	3.75995200	-2.71208500	-1.14632000
H	-7.68411100	-0.37058100	-1.56418400	H	3.43958600	-7.01089100	1.34545300
C	-4.03281300	-0.90885500	1.09189900	C	1.14663700	-3.90500400	-1.07549300
H	-2.99393100	-0.67471300	0.83569000	H	0.85168600	-2.90052100	-0.75436700
C	-4.48482800	-2.21302800	0.49712800	C	0.23691000	-4.96751500	-0.52623100
C	-3.91172500	-3.46495900	0.60235500	C	-1.13238300	-5.06622700	-0.60657400
C	-4.47671500	-4.57084700	-0.05869600	C	-1.82059700	-6.13231200	0.01544500
C	-5.64773000	-4.35554500	-0.81029000	C	-1.03937500	-7.10739300	0.68317100
C	-6.21810200	-3.09698800	-0.94776100	C	0.33533500	-7.01173800	0.79166300
C	-5.62750200	-2.01765900	-0.29582700	C	0.98690100	-5.92546900	0.19380500
H	-3.01219700	-3.59946000	1.19441600	H	-1.69684800	-4.31377000	-1.14719600
H	-6.10163300	-5.19548400	-1.32560800	H	-1.54584400	-7.94030000	1.15872100
H	-7.10110300	-2.96074900	-1.56313900	H	0.89551600	-7.75765200	1.34511400
H	2.99349400	0.67461200	0.83408000	H	-0.85171700	2.90065000	-0.75446200
C	5.64772000	4.35535300	-0.81101900	C	1.03943700	7.10745600	0.68315700
C	4.47643500	4.57071500	-0.05984500	C	1.82060500	6.13238200	0.01536100
C	3.91142300	3.46486200	0.60124100	C	1.13233300	5.06634700	-0.60668100
C	4.48457400	2.21291500	0.49616600	C	-0.23695000	4.96763200	-0.52623500
C	5.62753900	2.01750100	-0.29635300	C	-0.98688400	5.92554300	0.19392100
C	6.21824000	3.09683100	-0.94818000	C	-0.33526600	7.01180200	0.79174800
H	6.10170100	5.19519700	-1.32641400	H	1.54595600	7.94036200	1.15866000
H	3.01176800	3.59926000	1.19306500	H	1.69674900	4.31391500	-1.14738900
H	7.10143000	2.96066200	-1.56330200	H	-0.89540100	7.75770200	1.34526400
C	4.03226500	0.90873400	1.09074400	C	-1.14671200	3.90515100	-1.07549000
H	4.08685300	0.92127900	2.18556300	H	-1.14423400	3.89589400	-2.17163200
C	5.00051800	-0.07562100	0.49680100	C	-2.48613800	4.30736800	-0.52568000
C	5.02008300	-1.45230500	0.60266000	C	-3.69160500	3.65040000	-0.60561700
C	6.00527200	-2.20906300	-0.05768200	C	-4.84664900	4.17541000	0.01680600
C	6.97055800	-1.51255500	-0.80981100	C	-4.71607800	5.41808600	0.68434900
C	6.94580400	-0.13099400	-0.94782300	C	-3.50770000	6.08055000	0.79237000
C	5.94926800	0.59080600	-0.29599600	C	-2.37258700	5.51874200	0.19422400
H	4.26454100	-1.95955100	1.19402100	H	-3.76000800	2.71221500	-1.14618400
H	7.74092600	-2.07676500	-1.32489400	H	-5.59211600	5.84518900	1.16030100
H	7.68513700	0.37064500	-1.56317400	H	-3.43948200	7.01081800	1.34591400
B	-5.96919400	3.77377500	0.00035000	B	6.19078500	-3.38092600	0.00076300
B	3.77343800	5.96898500	-0.00202800	B	3.38089600	6.19069500	-0.00117500
H	-0.67529900	2.99508600	-0.83798100	H	2.90019800	0.85147400	0.75302600
C	1.51232100	6.97075000	0.80797000	C	5.41836900	4.71616500	-0.68369000
C	2.20867900	6.00538500	0.05572700	C	4.17531200	4.84657200	-0.01681800
C	1.45153600	5.02130800	-0.60581700	C	3.65000500	3.69140500	0.60513300
C	0.07490400	5.00191800	-0.49980000	C	4.30701600	2.48596100	0.52527200
C	-0.59134000	5.95024300	0.29364200	C	5.51875400	2.37256200	-0.19405300
C	0.13072600	6.94630400	0.94592100	C	6.08088900	3.50781400	-0.79163400
H	2.07663300	7.74080000	1.32335400	H	5.84575100	5.59233600	-1.15914700
H	1.95843700	4.26605200	-1.19775000	H	2.71155700	3.75969800	1.14525500
H	-0.37068800	7.68659700	1.56163100	H	7.01146900	3.43972500	-1.34467000
C	-0.90953000	4.03398700	-1.09404000	C	3.90453500	1.14642000	1.07460900
H	-0.92232600	4.08916800	-2.18882300	H	3.89473800	1.14372600	2.17074600
C	-2.21355000	4.48589900	-0.49888200	C	4.96730000	0.23677800	0.52570400
C	-3.46538500	3.91255300	-0.60357000	C	5.06600700	-1.13251400	0.60595700
C	-4.57123900	4.47733800	0.05782000	C	6.13237600	-1.82064700	-0.01566400
C	-4.35591200	5.64858800	0.80899500	C	7.10773700	-1.03934300	-0.68288900
C	-3.09739000	6.21925500	0.94590800	C	7.01209900	0.33537400	-0.79129500
C	-2.01809200	5.62871600	0.29384600	C	5.92555200	0.98686700	-0.19384700
H	-3.59984900	3.01294700	-1.19543900	H	4.31333100	-1.69704400	1.14621100
H	-5.19567600	6.10238500	1.32467000	H	7.94088400	-1.54575700	-1.15807900
H	-2.96118600	7.10232500	1.56119200	H	7.75825700	0.89562100	-1.34434800
C	7.29017400	-4.60880900	-0.00045600	C	-7.56480500	4.13066600	0.00195900
C	7.33864900	-5.89968400	-0.55199800	C	-8.69869600	3.58546500	0.62859000
C	8.47788200	-4.09908900	0.54956400	C	-7.72146300	5.37945900	-0.62377000
C	8.51322900	-6.63934600	-0.56652700	C	-9.91689000	4.25095800	0.63986000
H	6.44011900	-6.32249800	-0.98909000	H	-8.61620900	2.62620700	1.12927900
C	9.65019500	-4.84235300	0.56180400	C	-8.94095600	6.04262000	-0.63352800

H	8.47464400	-3.10643100	0.98749500
C	9.67043100	-6.11294400	-0.00296000
H	8.52750800	-7.62818300	-1.01208200
H	10.54991600	-4.43105200	1.00664500
H	10.58712500	-6.69297400	-0.00374300
C	-4.60894600	-7.28959700	0.00030300
C	-5.89985000	-7.33779100	0.55174900
C	-4.10008900	-8.47704900	-0.55111300
C	-6.64002300	-8.51206000	0.56570400
H	-6.32230000	-6.43926700	0.98923600
C	-4.84386600	-9.64903600	-0.56391100
H	-3.10773600	-8.47390000	-0.98970100
C	-6.11425500	-9.66913900	0.00130500
H	-7.62883000	-8.52613600	1.01133100
H	-4.43317900	-10.54857800	-1.00968200
H	-6.69473200	-10.58554900	0.00153100
C	4.60942000	7.28921500	0.00096200
C	4.10114600	8.47762800	-0.54892400
C	5.89968500	7.33645200	0.55395600
C	4.84534900	9.64936500	-0.55978600
H	3.10890900	8.47545300	-0.98777700
C	6.64032400	8.51040100	0.56970100
H	6.32141700	6.43731500	0.99089500
C	6.11541300	9.66830500	0.00621400
H	4.43514500	10.54968900	-1.00441500
H	7.62878100	8.52361100	1.01613400
H	6.69615500	10.58454800	0.00810500
C	-7.29041800	4.60825000	0.00059300
C	-7.33952400	5.89943600	-0.55013600
C	-8.47761900	4.09800400	0.55117900
C	-8.51435200	6.63874100	-0.56373500
H	-6.44127400	6.32283300	-0.98723200
C	-9.65018000	4.84086800	0.56429500
H	-8.47390100	3.10511400	0.98861500
C	-9.67113600	6.11169400	0.00010300
H	-8.52908200	7.62785100	-1.00866400
H	-10.54955300	4.42903700	1.00935500
H	-10.58801900	6.69142500	0.00007800
H	-6.87108400	5.83015300	-1.12516400
C	-10.04232800	5.48117400	0.00355800
H	-10.77094000	3.81031400	1.14317700
H	-9.03390000	6.99939400	-1.13638800
H	-10.99476300	6.00040200	0.00405200
C	-4.13070300	-7.56468800	-0.00207400
C	-3.58579100	-8.69847800	-0.62913100
C	-5.37943500	-7.72133100	0.62378200
C	-4.25144500	-9.91658200	-0.64059700
H	-2.62665100	-8.61597400	-1.13003900
C	-6.04275700	-8.94074000	0.63334500
H	-5.82995800	-6.87100600	1.12542100
C	-5.48155700	-10.04202900	-0.00409800
H	-3.81101300	-10.77054900	-1.14423900
H	-6.99947600	-9.03367600	1.13631200
H	-6.00091800	-10.99439100	-0.00475700
C	4.13074900	7.56466700	-0.00210400
C	5.37982500	7.72099900	0.62313500
C	3.58551500	8.69873600	-0.62838300
C	6.04317900	8.94038600	0.63287300
H	5.83060800	6.87042900	1.12413000
C	4.25119900	9.91682700	-0.63968100
H	2.62607400	8.61647900	-1.12876000
C	5.48166000	10.04196500	-0.00379200
H	7.00016500	9.03308100	1.13537500
H	3.81051000	10.77103100	-1.14269900
H	6.00103900	10.99431700	-0.00431200
C	7.56483000	-4.13068600	0.00209700
C	8.69853400	-3.58557600	0.62913700
C	7.72162800	-5.37949400	-0.62357200
C	9.91667500	-4.25115700	0.64085400
H	8.61592700	-2.62633600	1.12983900
C	8.94108300	-6.04273600	-0.63290400
H	6.87139900	-5.83014700	-1.12525400
C	10.04226400	-5.48137300	0.00458200
H	10.77056800	-3.81057900	1.14449500
H	9.03413800	-6.99951500	-1.13573400
H	10.99466200	-6.00066800	0.00541900

Structure S54. The coordinates of both the optimized structures of B3N1L$_3$ at S$_0$ and S$_1$ in toluene.

S_0-optimized structure				S_1-optimized structure			
B	7.02819500	0.01915300	-0.02640900	B	-0.04270500	6.99453000	-0.02764300
H	2.94570500	-2.94519600	2.15577100	H	2.93796900	2.91439100	2.15053600
C	2.43642700	-6.70445800	-0.81500500	C	6.73245300	2.45751000	-0.78481300
C	1.33244300	-6.26092700	-0.06190100	C	6.28890600	1.34188900	-0.04852800
C	1.44474000	-5.02077300	0.59283400	C	5.04149400	1.44283200	0.59651100
C	2.59378700	-4.26417300	0.47730200	C	4.27928600	2.58734800	0.48472600
C	3.66335300	-4.70945400	-0.31685200	C	4.72496500	3.67032200	-0.29340500
C	3.58820200	-5.94213800	-0.96026300	C	5.96623000	3.60746200	-0.92390300
H	2.37299700	-7.65951500	-1.32584200	H	7.69203200	2.40462700	-1.28861000
H	0.61397400	-4.65355000	1.18668000	H	4.67335200	0.60423200	1.17876200
H	4.40630900	-6.29995200	-1.57647100	H	6.32646800	4.43417100	-1.52716700
C	2.90250200	-2.91424800	1.06081700	C	2.91967200	2.87982100	1.05488500
H	2.14444700	-2.16762400	0.80074800	H	2.18374000	2.11624000	0.78050200
C	4.24280100	-2.59678100	0.46008700	C	2.59696200	4.22267800	0.46119200
C	4.98929500	-1.43934100	0.55828700	C	1.43309500	4.95772100	0.55404500
C	6.22663200	-1.32501900	-0.10085500	C	1.30739000	6.20217300	-0.09514100
C	6.67459400	-2.43128300	-0.84769300	C	2.42680500	6.66765200	-0.81681800
C	5.91961800	-3.58940500	-0.98022700	C	3.59439800	5.92771700	-0.93997300
C	4.69246600	-3.66977000	-0.32660700	C	3.67950700	4.69000000	-0.30407500
H	4.61853000	-0.60516800	1.14512300	H	0.59843000	4.57406800	1.13235400
H	7.62775500	-2.36575300	-1.36192700	H	2.36022300	7.62618900	-1.32111700
H	6.28003300	-4.40994200	-1.59169900	H	4.42014800	6.30596200	-1.53387500
B	-0.00948800	-7.06638000	0.00057400	B	7.09541400	0.00080100	-0.00006400
H	2.98504300	3.02871100	-2.26882300	H	-2.94117600	2.85157400	-2.23944400
C	2.25714300	6.58765100	0.91491100	C	-6.66885700	2.29579300	0.78570700
C	1.22225900	6.15942300	0.07172300	C	-6.18810400	1.20609600	0.02625500
C	1.38563900	4.98898100	-0.67929800	C	-4.95751300	1.31851800	-0.66370700
C	2.54122200	4.24805400	-0.53619400	C	-4.23258300	2.47399300	-0.54815000
C	3.57137300	4.66935400	0.31975600	C	-4.69784600	3.55982500	0.23662200
C	3.42985000	5.85661000	1.03239300	C	-5.93945800	3.45930500	0.88890600
H	2.12557300	7.49291100	1.49533100	H	-7.60227600	2.19199000	1.32529600
H	0.59379500	4.66132800	-1.34290200	H	-4.60889800	0.49649600	-1.27719100
H	4.20923200	6.20092100	1.70363000	H	-6.31251500	4.27468100	1.49770600
C	2.90233900	2.93825300	-1.17951800	C	-2.89513500	2.81197000	-1.14501200
H	2.14988000	2.16591400	-0.98787900	H	-2.13248100	2.06858500	-0.89147000
C	4.22744800	2.61491900	-0.54547800	C	-2.60385200	4.16237900	-0.54989300
C	4.99707900	1.47597700	-0.66603600	C	-1.46493300	4.92583200	-0.64563000
C	6.21330600	1.35327400	0.03257600	C	-1.37148400	6.17981100	0.00435300
C	6.61713100	2.43678700	0.83482300	C	-2.51217500	6.61817200	0.72311200
C	5.83441500	3.57336200	0.99387200	C	-3.65373600	5.85159700	0.85406900
C	4.62544000	3.65797400	0.30839500	C	-3.70380300	4.59781200	0.22582200
H	4.66035100	0.65980200	-1.29751100	H	-0.62003500	4.56566200	-1.22341900
H	7.55446300	2.36698600	1.37696700	H	-2.46876900	7.57885500	1.22525500
H	6.15869000	4.37224300	1.65251000	H	-4.48783900	6.20817000	1.44944500
H	-2.15011600	-2.16210300	-0.80075900	H	2.18419700	-2.11577300	-0.78045500
C	-6.68109500	-2.41322600	0.84736600	C	2.42831400	-6.66706500	0.81703600
C	-6.23013600	-1.30831200	0.10035300	C	1.30879000	-6.20186600	0.09535500
C	-4.99304000	-1.42604200	-0.55864700	C	1.43419300	-4.95741500	-0.55388200
C	-4.24966800	-2.58548000	-0.46021500	C	2.59789900	-4.22210600	-0.46108200
C	-4.70228100	-3.65713000	0.32660400	C	3.68056000	-4.68915600	0.30418200
C	-5.92920200	-3.57330800	0.98018000	C	3.59574300	-5.92686100	0.94013900
H	-7.63411700	-2.34506200	1.36151900	H	2.36195500	-7.62559700	1.32137600
H	-4.61998800	-0.59295300	-1.14557400	H	0.59942800	-4.57397200	-1.13218400
H	-6.29184800	-4.39275800	1.59178500	H	4.42159000	-6.30489300	1.53404300
C	-2.91019600	-2.90667900	-1.06078800	C	2.92029400	-2.87919800	-1.05482900
H	-2.95338400	-2.93767000	-2.15574100	H	2.93857200	-2.91380200	-2.15048000
C	-2.60520900	-4.25736200	-0.47704900	C	4.27985500	-2.58640100	-0.48471300
C	-1.45819800	-5.01706100	-0.59232900	C	5.04180500	-1.44171500	-0.59655400
C	-1.34930900	-6.25739200	0.06263100	C	6.28920700	-1.34047400	0.04845200
C	-2.45459200	-6.69787700	0.81564300	C	6.73302500	-2.45597100	0.78475900
C	-3.60437700	-5.93248800	0.96060200	C	5.96706000	-3.60608800	0.92390800
C	-3.67605700	-4.69965300	0.31705400	C	4.72579600	-3.66924300	0.29344600
H	-0.62637500	-4.65216100	-1.18613000	H	4.67346000	-0.60321200	-1.17881700
H	-2.39376900	-7.65301100	1.32664800	H	7.69260900	-2.40285800	1.28852400
H	-4.42355000	-6.28799600	1.57673100	H	6.32749900	-4.43269700	1.52719000
B	-7.02810400	0.03796600	0.02560100	B	-0.04112500	-6.99453600	0.02790700
H	-2.14423400	2.17160000	0.98798200	H	-2.13206800	-2.06905200	0.89154200
C	-6.61047000	2.45458100	-0.83528900	C	-2.51063000	-6.61875100	-0.72297600
C	-6.20965600	1.36990400	-0.03309600	C	-1.37009400	-6.18011700	-0.00415600
C	-4.99321000	1.48927100	0.66571000	C	-1.46385300	-4.92615600	0.64580000
C	-4.22055500	2.62618700	0.54545200	C	-2.60294400	-4.16296000	0.54998600
C	-4.61569600	3.67044900	-0.30827400	C	-3.70274000	-4.59865500	-0.22578800
C	-5.82474300	3.58912400	-0.99401400	C	-3.65235900	-5.85243100	-0.85401300
H	-7.54788600	2.38733400	-1.37760800	H	-2.46697800	-7.57943300	-1.22509900
H	-4.65874000	0.67210500	1.29710500	H	-0.61906400	-4.56578200	1.22362300
H	-6.14677100	4.38898600	-1.65256000	H	-4.48634600	-6.20920000	-1.44943300
C	-2.89466100	2.94589600	1.17969700	C	-2.89456600	-2.81260500	1.14505800
H	-2.97724100	3.03635800	2.26901000	H	-2.94065700	-2.85219600	2.23948900
C	-2.53002800	4.25486500	0.53666300	C	-4.23205700	-2.47494500	0.54811500
C	-1.37246200	4.99265000	0.67992700	C	-4.95725800	-1.31962900	0.66361300
C	-1.20594200	6.16282600	-0.07083100	C	-6.18782800	-1.20749700	-0.02642500
C	-2.23973400	6.59407000	-0.91383400	C	-6.66828500	-2.29731000	-0.78589400
C	-3.41437600	5.86617000	-1.03150200	C	-5.93861300	-3.46065900	-0.88903200
C	-3.55902500	4.67908700	-0.31922600	C	-4.69702600	-3.56088500	-0.23666700
H	-0.58149200	4.66268900	1.34343100	H	-4.60886700	-0.49751900	1.27710600
H	-2.10576700	7.49912400	-1.49402600	H	-7.60169300	-2.19372700	-1.32554300
H	-4.19283000	6.21269900	-1.70267500	H	-6.31144700	-4.27612700	-1.49784500
C	-0.01156300	-8.62887900	0.00050400	C	8.65908100	0.00096400	-0.00017700
C	1.05305800	-9.36147600	0.55069800	C	9.39046100	1.05769000	0.56641200
C	-1.07813300	-9.35860300	-0.54973400	C	9.39059200	-1.05561200	-0.56687500
C	1.04898600	-10.74953000	0.56422400	C	10.77862100	1.05571300	0.57952900
H	1.89145900	-8.82923900	0.98775100	H	8.85680500	1.88839800	1.01628900
C	-1.07786300	-10.74666100	-0.56318100	C	10.77875000	-1.05334500	-0.58021100
H	-1.91506300	-8.82410400	-0.98684200	H	8.85703900	-1.88643400	-1.01666500

C	-0.01539200	-11.44466000	0.00055000	C	11.47549400	0.00125700	-0.00039700
H	1.87677200	-11.29105300	1.00922700	H	11.31882000	1.87760100	1.03703900
H	-1.90710600	-11.28594800	-1.00818600	H	11.31904900	-1.87512200	-1.03780400
H	-0.01686500	-12.52950500	0.00057700	H	12.56037300	0.00137100	-0.00048200
C	8.59044200	0.02610100	0.00507900	C	-0.05095100	8.55820400	0.02155700
C	9.32530600	1.10718900	-0.50881300	C	-1.11603500	9.31005000	-0.50665300
C	9.31765400	-1.04540600	0.54898800	C	1.01143600	9.28164800	0.59301200
C	10.71331000	1.11472200	-0.49342900	C	-1.12176100	10.69743100	-0.47026200
H	8.79466400	1.94998100	-0.93930200	H	-1.94940300	8.79082300	-0.96889600
C	10.70513300	-1.03465100	0.59048100	C	1.00652900	10.66844900	0.64793900
H	8.78137100	-1.89471500	0.95916300	H	1.85098800	8.73912800	1.01530400
C	11.40562500	0.04451800	0.06268100	C	-0.06041700	11.38253400	0.11236600
H	11.25693300	1.95583100	-0.91002000	H	-1.95383800	11.24765800	-0.89721100
H	11.24217700	-1.86841100	1.02975600	H	1.83607800	11.19551400	1.10768000
H	12.49022800	0.05159300	0.08491000	H	-0.06409500	12.46681900	0.14749500
C	-8.59032200	0.04907100	-0.00631600	C	-0.04901800	-8.55821000	-0.02124400
C	-9.32243400	1.13208900	0.50743800	C	-1.11395100	-9.31027000	0.50696000
C	-9.32024800	-1.02046300	-0.55046900	C	1.01354900	-9.28142700	-0.59264700
C	-10.71040800	1.14337700	0.49161600	C	-1.11936100	-10.69765400	0.47062100
H	-8.78965300	1.97341400	0.93815200	H	-1.94745200	-8.79121200	0.96915500
C	-10.70768300	-1.00596700	-0.59237900	C	1.00895300	-10.66823100	-0.64753100
H	-8.78613200	-1.87120400	-0.96050700	H	1.85299100	-8.73873100	-1.01493000
C	-11.40543100	0.07506600	-0.06475900	C	-0.05784700	-11.38253400	-0.11195800
H	-11.25189000	1.98592400	0.90809000	H	-1.95132500	-11.24805500	0.89756500
H	-11.24683500	-1.83826600	-1.03184100	H	1.83863000	-11.19512800	-1.10723200
H	-12.49000400	0.08505700	-0.08732100	H	-0.06128000	-12.46682100	-0.14704900
C	0.01118500	8.28475600	0.00039100	C	-8.29666900	-0.00093600	-0.00017700
C	-0.94758500	8.99470000	0.72803000	C	-8.99320900	-0.91255300	0.79752200
C	0.97204800	8.99179700	-0.72731800	C	-8.99333900	0.91053200	-0.79793400
C	-0.94694500	10.38195700	0.71711000	C	-10.37867600	-0.90801900	0.79180900
H	-1.69088000	8.45375100	1.30170100	H	-8.44446800	-1.60201500	1.42748900
C	0.97562800	10.37904800	-0.71642400	C	-10.37880600	0.90570100	-0.79233400
H	1.71369800	8.44858000	-1.30097400	H	-8.44469300	1.60011300	-1.42785200
C	0.01540600	11.08373700	0.00034300	C	-11.07561900	-0.00123300	-0.00029100
H	-1.69869200	10.91681200	1.28716000	H	-10.91645600	-1.60906000	1.41922300
H	1.72898000	10.91161700	-1.28649400	H	-10.91668400	1.60662800	-1.41979200
H	0.01703500	12.16764300	0.00034500	H	-12.15931600	-0.00134800	-0.00033400
N	0.00914000	6.87467600	0.00047200	N	-6.88470400	-0.00077900	-0.00011500

Structure S55. The coordinates of both the optimized structures of B2N2L$_3$ at S_0 and S_1 in toluene.

S_0-optimized structure				S_1-optimized structure			
B	4.85390300	5.09019000	-0.02099500	B	4.99749000	4.94952000	0.03547200
H	4.14760400	0.08216100	2.13121600	H	0.00021200	3.73168700	-1.85686600
C	6.49362700	-2.88230100	-0.85294500	C	-3.01409500	6.54194700	0.62440600
C	5.40669400	-3.36930200	-0.10216800	C	-3.49801800	5.32154100	0.08461400
C	4.59203600	-2.42816500	0.55284000	C	-2.51317300	4.42070700	-0.40024200
C	4.84932400	-1.07618700	0.44449300	C	-1.17477100	4.71202700	-0.32510900
C	5.91423300	-0.61427900	-0.34614800	C	-0.72033400	5.92024000	0.25366000
C	6.74682100	-1.52380400	-0.99365600	C	-1.66840200	6.84239300	0.72342400
H	7.13713800	-3.59064200	-1.36428700	H	-3.73319900	7.25605000	1.01287000
H	3.74893400	-2.77174300	1.14335200	H	-2.83077300	3.48556300	-0.85023000
H	7.57348400	-1.18266300	-1.60788300	H	-1.35215400	7.77694300	1.17622200
C	4.09602600	0.08121000	1.03617800	C	0.00024700	3.88444600	-0.77102000
H	3.03241500	0.06007300	0.77525000	H	0.00019200	2.88714500	-0.31694800
C	4.80272000	1.26771900	0.44464700	C	1.17539700	4.71187100	-0.32516400
C	4.49187000	2.60838300	0.55315100	C	2.51375700	4.42037100	-0.40035600
C	5.26846200	3.58126500	-0.10170700	C	3.49874400	5.32107600	0.08445100
C	6.37391200	3.13798200	-0.85253000	C	3.01500800	6.54155200	0.62425300
C	6.68090700	1.79063500	-0.99342100	C	1.66936000	6.84217700	0.72333400
C	5.88514900	0.84862600	-0.34604500	C	0.72114700	5.92014500	0.25362400
H	3.63575700	2.91807200	1.14369300	H	2.83121200	3.48518200	-0.85035000
H	6.98875700	3.87141700	-1.36377800	H	3.73422700	7.25556400	1.01267400
H	7.52049600	1.48270900	-1.60766500	H	1.35325800	7.77677200	1.17614100
B	5.05257800	-4.89354200	-0.02160600	B	-4.99681500	4.95019000	0.03569000
H	-0.12014900	4.22125100	-2.23952700	H	4.39576700	-0.05056800	2.35048100
C	-3.19406200	6.16344700	0.93660900	C	6.19371500	-3.07690200	-0.95871000
C	-3.59805000	5.11389600	0.09853600	C	5.17353700	-3.48837200	-0.09212000
C	-2.63618000	4.41907900	-0.64513400	C	4.50498500	-2.54874300	0.70616700
C	-1.30318300	4.74770000	-0.50518300	C	4.82529900	-1.21615700	0.58144400
C	-0.89759200	5.78909100	0.34443900	C	5.82944700	-0.79131200	-0.31227900
C	-1.85590700	6.50849200	1.05324000	C	6.52772500	-1.73655100	-1.06622000
H	-3.94039100	6.69635500	1.51344700	H	6.70185200	-3.81476700	-1.56827500
H	-2.94371900	3.61537800	-1.30398900	H	3.73657600	-2.87751100	1.39684000
H	-1.56812600	7.31380900	1.72047400	H	7.30423000	-1.43139300	-1.75836300
C	-0.10671900	4.10543500	-1.14959800	C	4.21087000	-0.03032500	1.27007200
H	-0.06261200	3.02915800	-0.95153800	H	3.12407000	0.00318500	1.14050100
C	1.04160600	4.84834600	-0.52406900	C	4.90394500	1.12786100	0.60629600
C	2.39686400	4.62393000	-0.65122500	C	4.65450200	2.47791400	0.73560800
C	3.32641900	5.42199000	0.04276400	C	5.38397800	3.42462400	-0.01129900
C	2.82125300	6.46122800	0.84619400	C	6.41164100	2.93127200	-0.84396200
C	1.45846200	6.67638700	1.00980500	C	6.65955900	1.57624200	-1.00288100
C	0.56167800	5.85689400	0.32928700	C	5.88464800	0.66153300	-0.28799300
H	2.75413000	3.81761300	-1.28399800	H	3.87093500	2.82193400	1.40323300
H	3.51911100	7.09317600	1.38575000	H	7.00268500	3.64075300	-1.41413300
H	1.10500200	7.46251000	1.66875300	H	7.42848000	1.23803200	-1.69010500
H	0.05807800	-3.02896100	-0.95040500	H	-3.12407400	0.00354800	1.14052300
C	-2.94635000	-6.28520200	0.93768600	C	-6.19414100	-3.07602500	-0.95882000
C	-3.39181200	-5.25246500	0.09973600	C	-5.17402000	-3.48767400	-0.09224600
C	-2.45835400	-4.51993900	-0.64397300	C	-4.50534200	-2.54817000	0.70608300
C	-1.11336100	-4.79544900	-0.50428500	C	-4.82547200	-1.21553500	0.58141600
C	-0.66660900	-5.82012400	0.34503400	C	-5.82956100	-0.79051300	-0.31228800
C	-1.59553800	-6.57698100	1.05397200	C	-6.52796800	-1.73562400	-1.06627000
H	-3.67085800	-6.84736100	1.51459800	H	-6.70237500	-3.81379100	-1.56842300
H	-2.79762500	-3.72905100	-1.30276500	H	-3.73698100	-2.87707100	1.39674500
H	-1.27596000	-7.37037500	1.72102600	H	-7.30443100	-1.43033000	-1.75840200
C	0.05654900	-4.10608300	-1.14877800	C	-4.21087900	-0.02981600	1.27009400
H	0.04739200	-4.22201100	-2.23873700	H	-4.39577800	-0.05007900	2.35050200
C	1.23356500	-4.80309200	-0.52375500	C	-4.90379400	1.12849300	0.60636700
C	2.57881800	-4.52500700	-0.65106300	C	-4.65416300	2.47850600	0.73573300
C	3.53948300	-5.28589500	0.04229600	C	-5.38351300	3.42534700	-0.01113000
C	3.07614900	-6.34479000	0.84526200	C	-6.41124700	2.93217300	-0.84380900
C	1.72299400	-6.61388100	1.00912300	C	-6.65935100	1.57718300	-1.00278700
C	0.79421400	-5.83022700	0.32928700	C	-5.88456300	0.66233800	-0.28794000
H	2.90365000	-3.70482900	-1.28351100	H	-3.87054500	2.82239000	1.40336900
H	3.79866700	-6.94884700	1.38430900	H	-7.00219900	3.64176100	-1.41394500
H	1.40119600	-7.41377600	1.66770300	H	-7.42832100	1.23910800	-1.69002200
H	-3.08803700	-0.06105300	1.21040100	H	-0.00017500	-2.83265500	-0.80878200
C	-6.05811700	-3.13181800	-1.02187100	C	-2.99582700	-6.33266900	0.67041000
C	-5.11653300	-3.55377500	-0.07604100	C	-3.44693200	-5.18208800	-0.01917900
C	-4.49018400	-2.60973300	0.74589300	C	-2.50636100	-4.34648900	-0.66039900
C	-4.77059700	-1.26936800	0.57178300	C	-1.17168900	-4.63843000	-0.55987800
C	-5.69712600	-0.84383800	-0.39396000	C	-0.71814800	-5.76557500	0.16557600
C	-6.35816200	-1.78554200	-1.17658000	C	-1.65334800	-6.62179200	0.76414300
H	-6.53969000	-3.86972400	-1.65263700	H	-3.71877100	-6.96527000	1.16908800
H	-3.77051300	-2.93905800	1.48698000	H	-2.84725000	-3.48408400	-1.21906900
H	-7.07788800	-1.48015300	-1.92836100	H	-1.33101800	-7.48897100	1.32891800
C	-4.18051700	-0.08275500	1.28372900	C	-0.00024400	-3.88185400	-1.11912800
H	-4.42074800	-0.08736100	2.35312300	H	-0.00022700	-3.88588500	-2.21467000
C	-4.81729000	1.07924100	0.57140700	C	1.17107500	-4.63859500	-0.55984000
C	-4.59038000	2.42973100	0.74509800	C	2.50579100	-4.34683800	-0.66031600
C	-5.25379800	3.34789900	-0.07707100	C	3.44622600	-5.18256000	-0.01905300
C	-6.17789100	2.88856000	-1.02273300	C	2.99493500	-6.33307700	0.67052300
C	-6.42419200	1.53136500	-1.17703300	C	1.65241300	-6.62201800	0.76420400
C	-5.72618500	0.61692400	-0.39418300	C	0.71735200	-5.76567500	0.16560100
H	-3.88430300	2.78761400	1.48602500	H	2.84681600	-3.48448200	-1.21897700
H	-6.68844100	3.60655500	-1.65368900	H	3.71777100	-6.96577800	1.16923100
H	-7.13130400	1.19737200	-1.92863400	H	1.32994400	-7.48915000	1.32897100
C	6.17258000	-5.98269500	0.01057000	C	-6.13092000	6.03426800	0.00437000
C	7.44266400	-5.71264900	0.54603000	C	-5.94694700	7.28994900	-0.60180100
C	5.94904200	-7.27382200	-0.49528400	C	-7.39653600	5.78747400	0.56591000
C	8.43500700	-6.68250800	0.58655900	C	-6.95815500	8.24047000	-0.64308700
H	7.64777300	-4.72681400	0.95003600	H	-4.99188600	7.51522600	-1.06493000
C	6.94359700	-8.24212300	-0.48024400	C	-8.40967500	6.73633900	0.54131900
H	4.97992800	-7.51480200	-0.91946000	H	-7.58259500	4.82948500	1.04143800
C	8.18785800	-7.94855000	0.06702100	C	-8.19457600	7.96900800	-0.06642900

H	9.40283200	-6.45215800	1.01879100	H	-6.78362800	9.19536600	-1.12839900
H	6.74935400	-9.22731800	-0.89040100	H	-9.37045900	6.51546100	0.99526300
H	8.96431800	-8.70587900	0.08877600	H	-8.98549600	8.71121200	-0.09331300
C	5.92952700	6.22317000	0.01124000	C	6.13173300	6.03345100	0.00405700
C	5.65413700	7.50464300	-0.49355400	C	7.39734400	5.78652200	0.56555000
C	7.20970500	6.00385800	0.54585800	C	5.94789200	7.28912900	-0.60216100
C	6.60900900	8.51209400	-0.47813700	C	8.41060300	6.73525700	0.54086700
H	4.67583500	7.70689300	-0.91690600	H	7.58330300	4.82853300	1.04111600
C	8.16231700	7.01275500	0.58676000	C	6.95921800	8.23951900	-0.64353800
H	7.45444200	5.02674500	0.94897900	H	4.99283800	7.51450500	-1.06525700
C	7.86431900	8.26825900	0.06831800	C	8.19563200	7.96792600	-0.06692700
H	6.37514000	9.48903200	-0.88736100	H	9.37138100	6.51427800	0.99477500
H	9.13882800	6.82109200	1.01834300	H	6.78478900	9.19441600	-1.12888400
H	8.60976700	9.05612700	0.09028200	H	8.98664600	8.71002800	-0.09388400
C	-5.75232500	-5.91405200	0.01792600	C	-5.79276600	-5.84327600	0.02842700
C	-6.96889700	-5.70330400	0.67377900	C	-5.69233600	-6.99294500	-0.75784800
C	-5.54514600	-7.12398100	-0.65123300	C	-6.89769600	-5.66511600	0.86283200
C	-7.95389500	-6.67972900	0.65241300	C	-6.68353400	-7.96070200	-0.69358000
H	-7.13637100	-4.77139400	1.20063800	H	-4.84727100	-7.11469400	-1.42512300
C	-6.53010500	-8.10092900	-0.65098600	C	-7.88696400	-6.63503800	0.91183500
H	-4.61057400	-7.29345000	-1.17261200	H	-6.96974900	-4.77220500	1.47186500
C	-7.74175500	-7.88640100	-0.00435700	C	-7.78355000	-7.78656400	0.13874600
H	-8.89092400	-6.49773100	1.16731800	H	-6.60262300	-8.84815900	-1.31060100
H	-6.35083100	-9.03304400	-1.17570000	H	-8.73945500	-6.49290500	1.56582700
H	-8.51125400	-8.64966600	-0.01279000	H	-8.55918400	-8.54221000	0.18122800
C	-5.98319200	5.68094200	0.01732800	C	5.79198100	-5.84403800	0.02875800
C	-7.18955200	5.42215000	0.67486200	C	5.69148500	-6.99374900	-0.75744600
C	-5.82551600	6.89768100	-0.65302000	C	6.89686800	-5.66594400	0.86323500
C	-8.21303800	6.35816800	0.65369000	C	6.68257100	-7.96161200	-0.69303600
H	-7.31886900	4.48474000	1.20270200	H	4.84646000	-7.11545300	-1.42477800
C	-6.84899800	7.83418600	-0.65264700	C	7.88602500	-6.63597300	0.91238000
H	-4.89918000	7.10406700	-1.17578600	H	6.96897500	-4.77299900	1.47221100
C	-8.05031600	7.57176800	-0.00440700	C	7.78254200	-7.78753900	0.13936200
H	-9.14138200	6.13917500	1.16993100	H	6.60160900	-8.84910300	-1.31000200
H	-6.70792200	8.77224400	-1.17841500	H	8.73848100	-6.49388700	1.56642800
H	-8.84985600	8.30350700	-0.01255000	H	8.55809000	-8.54326900	0.18195600
N	-4.95007800	4.72484700	0.02645400	N	4.78910400	-4.84850100	-0.03409800
N	-4.75820100	-4.91749400	0.02777400	N	-4.78976900	-4.84785300	-0.03428400

Structure S56. The coordinates of both the optimized structures of B1N3L$_3$ at S$_0$ and S$_1$ in toluene.

S_0-optimized structure				S_1-optimized structure			
B	7.00240000	-0.00171200	0.00029300	B	0.01270200	-6.96117000	0.00253400
H	2.94787900	-2.99306000	2.21332000	H	2.97717700	-2.89177300	-2.20586400
C	2.22952400	-6.57733500	-0.94179300	C	6.62295800	-2.22667200	0.90068000
C	1.19304500	-6.14234500	-0.10303900	C	6.16625200	-1.17418400	0.09119200
C	1.35645000	-4.96396900	0.63563400	C	4.96371700	-1.31702200	-0.63546400
C	2.51244700	-4.22535800	0.48807500	C	4.23198300	-2.46632000	-0.49771500
C	3.54461300	-4.65325400	-0.36202300	C	4.67567500	-3.53107300	0.33084400
C	3.40230500	-5.84684800	-1.06448500	C	5.89892800	-3.39840200	1.01457400
H	2.09938800	-7.48735400	-1.51509500	H	7.54420200	-2.10319200	1.45863900
H	0.56435700	-4.62892900	1.29503600	H	4.63012700	-0.51702800	-1.28690200
H	4.18190000	-6.19752400	-1.73227000	H	6.26366100	-4.19372000	1.65456300
C	2.87098500	-2.91132800	1.12290500	C	2.90611500	-2.81872300	-1.11406700
H	2.12012100	-2.14000400	0.92098500	H	2.14049300	-2.06468800	-0.90229500
C	4.19893400	-2.59421600	0.49294900	C	2.59587800	-4.15169100	-0.48631100
C	4.96911400	-1.45610000	0.61329800	C	1.45800700	-4.90916400	-0.58990600
C	6.18973600	-1.33698700	-0.07787100	C	1.33861900	-6.16531900	0.06786300
C	6.59590000	-2.42353100	-0.87458600	C	2.47782100	-6.59066200	0.80870000
C	5.81228100	-3.55997300	-1.03371400	C	3.61436200	-5.82260900	0.95546000
C	4.59955300	-3.64197700	-0.35384700	C	3.68637200	-4.57093600	0.31624200
H	4.62978600	-0.63754100	1.24010700	H	0.62736500	-4.55292400	-1.19114200
H	7.53643300	-2.35730900	-1.41184000	H	2.42777400	-7.54455800	1.32414100
H	6.13956200	-4.36149800	-1.68771100	H	4.43411000	-6.17616900	1.57349500
H	2.94943000	2.99143100	-2.21316900	H	-3.05034200	-2.92951000	2.12439700
C	2.23270800	6.57632500	0.94161200	C	-6.56320100	-2.16431300	-1.10527900
C	1.19604700	6.14177200	0.10285700	C	-6.16037000	-1.15280300	-0.22717000
C	1.35890400	4.96325200	-0.63571000	C	-5.00356900	-1.32791200	0.54088500
C	2.51453500	4.22409000	-0.48804600	C	-4.24987400	-2.47191400	0.38189400
C	3.54687900	4.65155400	0.36205300	C	-4.64849000	-3.48974300	-0.50252900
C	3.40512800	5.84527600	1.06440900	C	-5.82434900	-3.33426500	-1.23316400
H	2.10299500	7.48645400	1.51483500	H	-7.45893400	-2.02328800	-1.69911400
H	0.56667200	4.62854200	-1.29511100	H	-4.69445400	-0.55132900	1.23145500
H	4.18486900	6.19562700	1.73219400	H	-6.15431000	-4.10087500	-1.92606500
C	2.87245600	2.90983000	-1.12274900	C	-2.94656000	-2.83359100	1.03718600
H	2.12120800	2.13889000	-0.92079200	H	-2.17793200	-2.07210600	0.86483800
C	4.20022600	2.59212300	-0.49271700	C	-2.60634100	-4.14871100	0.39052600
C	4.96985600	1.45362100	-0.61293600	C	-1.47311900	-4.92017800	0.54057400
C	6.19039400	1.33397200	0.07829000	C	-1.31966800	-6.13832800	-0.15323500
C	6.59705900	2.42038800	0.87492400	C	-2.39354600	-6.53500400	-0.97820100
C	5.81398800	3.55722600	1.03392200	C	-3.52779900	-5.75433300	-1.16312900
C	4.60132600	3.63976300	0.35400300	C	-3.63457500	-4.54168800	-0.48342300
H	4.63015000	0.63517100	-1.23968300	H	-0.67777500	-4.58752300	1.20080200
H	7.53753700	2.35375300	1.41222200	H	-2.31492600	-7.47460300	-1.51650500
H	6.14163500	4.35864800	1.68786300	H	-4.31135700	-6.08431200	-1.83842400
H	-2.16384700	-2.17900400	-1.18448400	H	2.10304300	1.99016500	0.94358300
C	-6.43645600	-2.17719300	1.05027000	C	2.34681800	6.53933200	-0.68226800
C	-6.08439100	-1.20473900	0.10627000	C	1.24132400	6.05013500	0.04712900
C	-4.96741900	-1.40926500	-0.71213500	C	1.35070100	4.81764200	0.72136200
C	-4.20154200	-2.54465800	-0.54149900	C	2.51271900	4.09462800	0.61848000
C	-4.54080700	-3.50923300	0.42094100	C	3.60584800	4.56692100	-0.14297600
C	-5.67742400	-3.32903900	1.20375400	C	3.51400800	5.81191500	-0.77779100
H	-7.30277700	-2.01238100	1.68004700	H	2.25672200	7.48272000	-1.20556000
H	-4.70175800	-0.66199200	-1.45137700	H	0.51574900	4.44891100	1.30427700
H	-5.96013500	-4.05957000	1.95410700	H	4.33566200	6.19497800	-1.37248600
C	-2.94198700	-2.94606100	-1.25874800	C	2.84056200	2.75296400	1.21106200
H	-3.11663200	-3.11020600	-2.32830400	H	2.87160100	2.78976200	2.30571600
C	-2.55195400	-4.21593000	-0.55349000	C	4.19399500	2.46095600	0.62589600
C	-1.42673300	-4.99393700	-0.73567500	C	4.94193600	1.31879300	0.72595400
C	-1.22550300	-6.11152100	0.08253900	C	6.17783900	1.23907700	0.04102800
C	-2.19478700	-6.45742400	1.03110500	C	6.64310900	2.36392200	-0.68390800
C	-3.33996700	-5.68950200	1.19241100	C	5.88718000	3.50983000	-0.77433300
C	-3.51341300	-4.54849900	0.41485300	C	4.63651600	3.56580100	-0.14007000
H	-0.68186800	-4.73373300	-1.47933300	H	4.60044200	0.47412400	1.31058500
H	-2.03269600	-7.32545200	1.65923200	H	7.58172900	2.29485600	-1.21836400
H	-4.06865700	-5.96772200	1.94618100	H	6.24608600	4.34266100	-1.36816700
H	-2.16276500	2.18014700	1.18457600	H	-2.19278100	2.14456400	-1.21240100
C	-6.43539700	2.18025500	-1.05013300	C	-2.16797600	6.40192400	1.06178600
C	-6.08380200	1.20770100	-0.10606100	C	-1.17944400	6.02943400	0.14566600
C	-4.96671800	1.41174200	0.71231400	C	-1.37806600	4.92520900	-0.69136500
C	-4.20028800	2.54674800	0.54158500	C	-2.53398600	4.18426000	-0.56193700
C	-4.53909000	3.51141600	-0.42092600	C	-3.51889200	4.53990600	0.37652100
C	-5.67580100	3.33171500	-1.20371600	C	-3.33929900	5.66667600	1.17501200
H	-7.30180500	2.01581900	-1.67989000	H	-2.00103100	7.26089600	1.70095500
H	-4.70141500	0.66439600	1.45161100	H	-0.61624900	4.65387500	-1.41367000
H	-5.95816200	4.06232600	-1.95412200	H	-4.08725400	5.95886500	1.90366700
C	-2.94053000	2.94759000	1.25879000	C	-2.94276900	2.93787000	-1.29704400
H	-3.11508400	3.11190000	2.32833500	H	-3.08008000	3.12442300	-2.36834500
C	-2.54988400	4.21721600	0.55343400	C	-4.23524300	2.56852400	-0.62269800
C	-1.42428200	4.99468900	0.73554900	C	-5.01920400	1.45036200	-0.81775300
C	-1.22251600	6.11211200	-0.08275200	C	-6.15923300	1.26265500	-0.02519300
C	-2.19164000	6.45841600	-1.03133400	C	-6.52037900	2.24833000	0.90516800
C	-3.33719900	5.69104300	-1.19256800	C	-5.74248900	3.38262500	1.08170600
C	-3.51119100	4.55018200	-0.41492400	C	-4.57781700	3.53661400	0.33509900
H	-0.67953600	4.73417800	1.47921800	H	-4.74539700	0.69672000	-1.54694900
H	-2.02913300	7.32631800	-1.65952900	H	-7.40374900	2.10110500	1.51467500
H	-4.06576200	5.96956200	-1.94635100	H	-6.02973800	4.11639400	1.82715400
C	-0.05120400	-8.25568200	-0.01605100	C	8.27942500	0.02342300	-0.10985300
C	0.93961600	-8.98575500	0.64731900	C	8.88672300	-0.91740900	-0.94578100
C	-1.07956000	-8.94580900	-0.66491100	C	9.06691300	0.92295400	0.61463700
C	0.90366300	-10.37254800	0.64887400	C	10.26713900	-0.94433700	-1.06115900
H	1.73507300	-8.46154500	1.16344700	H	8.27141600	-1.61137600	-1.50501800
C	-1.11583100	-10.33220300	-0.64150900	C	10.44663800	0.88871900	0.48652100
H	-1.84849200	-8.38926700	-1.18748300	H	8.59345600	1.62902100	1.28645600
C	-0.12403500	-11.05640800	0.00984300	C	11.05259800	-0.04265800	-0.34979600
H	1.68130700	-10.92115100	1.16907600	H	10.73154900	-1.67069400	-1.71795300

H	-1.92188300	-10.84926200	-1.15061200	H	11.05200400	1.58357900	1.05709700
H	-0.15193800	-12.13987600	0.01981400	H	12.13194100	-0.06997200	-0.44247100
C	8.56502300	-0.00209000	0.00019300	C	-0.03159000	-8.52233200	0.10645400
C	9.29647200	1.08616800	-0.50316600	C	-1.17234800	-9.20109400	0.57974600
C	9.29601200	-1.09069800	0.50346300	C	1.05468500	-9.33264800	-0.28065100
C	10.68455700	1.08482200	-0.51590300	C	-1.22270200	-10.58440900	0.67500500
H	8.76274200	1.94182300	-0.90340400	H	-2.03451400	-8.62010700	0.89169500
C	10.68409900	-1.09001400	0.51603600	C	1.01161800	-10.71676300	-0.19420700
H	8.76192100	-1.94609600	0.90376900	H	1.94752000	-8.85795100	-0.67498700
C	11.38091100	-0.00276300	0.00002400	C	-0.12866600	-11.35310800	0.28802200
H	11.22526200	1.93228500	-0.92339000	H	-2.11747200	-11.06782000	1.05486900
H	11.22444700	-1.93773500	0.92345800	H	1.86779900	-11.30492700	-0.50987100
H	12.46579000	-0.00302200	-0.00004100	H	-0.16547200	-12.43517500	0.35815800
C	-8.21656600	0.00200500	0.00018500	C	0.00619400	8.15927700	0.00786800
C	-8.92833400	1.05244300	0.58904700	C	-0.95306600	8.80047500	-0.77911800
C	-8.92886800	-1.04809200	-0.58863700	C	0.91642900	8.92345200	0.74238400
C	-10.31532200	1.05171200	0.57816500	C	-0.99530800	10.18566400	-0.82922700
H	-8.38902700	1.86704000	1.05743100	H	-1.65826700	8.20925300	-1.35084400
C	-10.31585500	-1.04669800	-0.57768100	C	0.87380500	10.30803600	0.67385600
H	-8.38997600	-1.86294900	-1.05704600	H	1.64715200	8.42863100	1.37128400
C	-11.02083800	0.00267600	0.00026100	C	-0.08147100	10.94667400	-0.10880900
H	-10.84758200	1.87576800	1.04081000	H	-1.74282300	10.67213000	-1.44558200
H	-10.84853400	-1.87050000	-1.04029600	H	1.58342700	10.89058300	1.25047800
H	-12.10469100	0.00293600	0.00029000	H	-0.11586300	12.02892100	-0.15391700
C	-0.04717000	8.25570800	0.01563800	C	-8.28753500	0.05276100	-0.02319800
C	-1.07513000	8.94639700	0.66452900	C	-9.04719200	1.07172000	-0.60741400
C	0.94394100	8.98523800	-0.64789500	C	-8.94678000	-0.97331000	0.66064100
C	-1.11072900	10.33280600	0.64100000	C	-10.42967200	1.06679200	-0.49661300
H	-1.84428300	8.39027900	1.18722700	H	-8.54744600	1.86446200	-1.15162500
C	0.90866200	10.37204800	-0.64957800	C	-10.33112800	-0.97740400	0.75035000
H	1.73909300	8.46059400	-1.16405100	H	-8.36906900	-1.76567000	1.12106700
C	-0.11864300	11.05646800	-0.01051300	C	-11.08320500	0.04216200	0.17847500
H	-1.91648100	10.85030500	1.15013100	H	-11.00042200	1.86569600	-0.95751200
H	1.68652300	10.92022300	-1.16990500	H	-10.82348600	-1.78229400	1.28517400
H	-0.14602000	12.13994800	-0.02058300	H	-12.16428900	0.03716400	0.25588400
N	-0.02033300	6.84848800	0.02636400	N	-6.88517800	0.06135200	-0.13093100
N	-6.81147400	0.00166300	0.00015200	N	0.03963700	6.74890500	0.07373300
N	-0.02368100	-6.84847400	-0.02664700	N	6.87450900	0.04555400	0.02235300

Structure S57. The coordinates of both the optimized structures of N4L$_3$ at S$_0$ and S$_1$ in toluene.

S$_0$-optimized structure				S$_1$-optimized structure			
H	4.16369900	1.31830000	2.30586200	H	2.29422400	3.53629800	2.28284300
C	6.72730400	-1.03690300	-1.03961700	C	5.87782500	3.54478600	-0.95065500
C	5.96033500	-1.73907500	-0.10192700	C	5.70427600	2.45366100	-0.07967600
C	5.05374300	-1.04404400	0.70708700	C	4.52219900	2.35999500	0.68191200
C	4.89201700	0.31492000	0.53070800	C	3.54478000	3.31220600	0.52972800
C	5.64441600	1.01634700	-0.42541300	C	3.70324900	4.39397000	-0.36622300
C	6.58109200	0.33460700	-1.19666700	C	4.89759000	4.50949800	-1.09099500
H	7.42766400	-1.58069500	-1.66265000	H	6.78583500	3.60943500	-1.53878800
H	4.46810500	-1.58540900	1.44157500	H	4.39275400	1.53000800	1.36690600
H	7.17440800	0.85361900	-1.94184700	H	5.04963000	5.32960500	-1.78429900
C	3.94496000	1.24778200	1.23405800	C	2.20023000	3.39473500	1.19993300
H	2.90393400	0.91863100	1.14966000	H	1.61020400	2.48384300	1.05480100
C	4.18451700	2.55377000	0.52779500	C	1.57283500	4.58912000	0.53382000
C	3.53595300	3.75934400	0.70100200	C	0.30448600	5.09098000	0.69050400
C	3.87932000	4.84742700	-0.11001500	C	-0.09597700	6.20929200	-0.06793500
C	4.91125900	4.71122600	-1.04660500	C	0.82640500	6.81632800	-0.93977400
C	5.57963200	3.50429500	-1.20050500	C	2.10744500	6.31714600	-1.08452600
C	5.20408500	2.40981900	-0.42716600	C	2.49250700	5.17822600	-0.36352900
H	2.74506100	3.86742500	1.43469700	H	-0.39796300	4.63189000	1.37651800
H	5.17272300	5.55729900	-1.67124600	H	0.51243800	7.67260200	-1.52523800
H	6.36404400	3.41878400	-1.94488900	H	2.79198800	6.79319800	-1.77814900
H	-1.31814300	4.16321600	-2.30558000	H	-3.53864200	2.29654300	-2.28387900
C	-4.71130900	4.91146200	1.04646400	C	-6.81561500	0.82559200	0.94041400
C	-4.84743800	3.87931500	0.11009000	C	-6.20939600	-0.09595400	0.06712500
C	-3.75928700	3.53577600	-0.70076600	C	-5.09180100	0.30523700	-0.69198500
C	-2.55372800	4.18437700	-0.52760000	C	-4.58980100	1.57343800	-0.53456100
C	-2.40984400	5.20414400	0.42716000	C	-5.17804200	2.49223900	0.36424800
C	-3.50438500	5.57986000	1.20032500	C	-6.31629100	2.10648700	1.08593600
H	-5.55743400	5.17307200	1.67097200	H	-7.67132000	0.51105400	1.52640200
H	-3.86730600	2.74473400	-1.43430900	H	-4.63334600	-0.39655400	-1.37909600
H	-3.41893800	6.36442800	1.94455100	H	-6.79167800	2.79035400	1.78068100
C	-1.24769400	3.94467800	-1.23373000	C	-3.39604800	2.20147600	-1.20119800
H	-0.91855000	2.90366800	-1.14911200	H	-2.48502800	1.61129200	-1.05751800
C	-0.31487900	4.89187700	-0.53050700	C	-3.31285100	3.54535500	-0.52973200
C	1.04409000	5.05357400	-0.70685200	C	-2.36075700	4.52289600	-0.68184200
C	1.73908900	5.96032300	0.10202000	C	-2.45363200	5.70419400	0.08105300
C	1.03685500	6.72746900	1.03952300	C	-3.54390300	5.87689100	0.95327100
C	-0.33466500	6.58129100	1.19652200	C	-4.50852100	4.89655300	1.09353500
C	-1.01636900	5.64446300	0.42542200	C	-4.39374600	3.70295100	0.36742100
H	1.58548400	4.46779300	-1.44120400	H	-1.53144300	4.39411800	-1.36777600
H	1.58060100	7.42795500	1.66245400	H	-3.60793800	6.78428800	1.54241600
H	-0.85371100	7.17476000	1.94155800	H	-5.32794700	5.04791200	1.78779200
H	0.91856100	-2.90358300	-1.14908600	H	2.48183500	-1.60889100	-1.04918100
C	-1.03685100	-6.72755500	1.03924700	C	3.54611900	-5.87863900	0.95004800
C	-1.73907600	-5.96032100	0.10181100	C	2.45356200	-5.70418000	0.08105900
C	-1.04408400	-5.05350500	-0.70698400	C	2.35860600	-4.52130200	-0.67911600
C	0.31489000	-4.89183700	-0.53063300	C	3.31103900	-3.54401800	-0.52746400
C	1.01637300	-5.64450300	0.42523600	C	4.39427800	-3.70341100	0.36654000
C	0.33466700	-6.58139300	1.19626400	C	4.51102300	-4.89851400	1.08986200
H	-1.58061200	-7.42808600	1.66211500	H	3.61179400	-6.78731400	1.53704100
H	-1.58547900	-4.46765100	-1.44127800	H	1.52750800	-4.39118200	-1.36263700
H	0.85371500	-7.17492000	1.94125300	H	5.33229500	-5.05127800	1.78162800
C	1.24770900	-3.94458500	-1.23377700	C	3.39247100	-2.19878600	-1.19643200
H	1.31816900	-4.16304400	-2.30564200	H	3.53222400	-2.29168400	-2.27967100
C	2.55373900	-4.18433800	-0.52765700	C	4.58798700	-1.57209500	-0.53168900
C	3.75930000	-3.53573400	-0.70078000	C	5.08966800	-0.30362800	-0.68799000
C	4.84745400	-3.87932300	0.11005600	C	6.20930000	0.09600200	0.06891700
C	4.71131000	-4.91153300	1.04636100	C	6.81771100	-0.82722300	0.93889400
C	3.50438700	-5.57994600	1.20016400	C	6.31867900	-2.10836500	1.08326100
C	2.40984500	-5.20417800	0.42702700	C	5.17854500	-2.49267100	0.36377700
H	3.86732100	-2.74464800	-1.43427500	H	4.62948800	0.39952400	-1.37255800
H	5.55742600	-5.17319600	1.67085900	H	7.67500800	-0.51387400	1.52319300
H	3.41894500	-6.36457200	1.94433100	H	6.79585400	-2.79361800	1.77541100
H	-2.90349900	-0.91851200	1.14887000	H	-1.61029100	-2.48318100	1.05279500
C	-4.91167000	-4.71134700	-1.04627600	C	-0.82633800	-6.81676300	-0.93943600
C	-3.87934200	-4.84744200	-0.11009800	C	0.09594900	-6.20927600	-0.06781900
C	-3.53566900	-3.75925800	0.70066500	C	-0.30452500	-5.09050000	0.68990800
C	-4.18431100	-2.55371400	0.52758800	C	-1.57285400	-4.58870800	0.53283100
C	-5.20426500	-2.40986400	-0.42698200	C	-2.49243600	-5.17826300	-0.36431600
C	-5.58011500	-3.50443400	-1.20003800	C	-2.10732800	-6.31758900	-1.08464900
H	-5.17340100	-5.55748800	-1.67071100	H	-0.51232600	-7.67338300	-1.52437100
H	-2.74449200	-3.86725200	1.43406700	H	0.39787600	-4.63105100	1.37573600
H	-6.36483500	-3.41902700	-1.94410900	H	-2.79180600	-6.79398800	-1.77809800
C	-3.94449400	-1.24765900	1.23364000	C	-2.20032500	-3.39400800	1.19830200
H	-4.16288100	-1.31809200	2.30552300	H	-2.29443100	-3.53505200	2.28127000
C	-4.89181500	-0.31486700	0.53055300	C	-3.54480700	-3.31181300	0.52792300
C	-5.05346000	1.04411200	0.70686200	C	-4.52227200	-2.35957300	0.67960500
C	-5.96036300	1.73907900	-0.10186400	C	-5.70423600	-2.45355500	-0.08212000
C	-6.72770900	1.03681200	-1.03917500	C	-5.87764300	-3.54502500	-0.95269400
C	-6.58157200	-0.33471600	-1.19614400	C	-4.89738600	-4.50979600	-1.09248400
C	-5.64459500	-1.01639300	-0.42519900	C	-3.70315200	-4.39396600	-0.36758100
H	-4.46752800	1.58553800	1.44107100	H	-4.39294300	-1.52931900	1.36429700
H	-7.42831900	1.58053800	-1.66198500	H	-6.78557500	-3.60993300	-1.54091700
H	-7.17519500	-0.85379200	-1.94103600	H	-5.04932700	-5.33020400	-1.78545400
C	7.29647500	-3.79268800	0.00527300	C	8.03326900	1.71907600	-0.01336900
C	8.41075800	-3.19175600	0.60006300	C	8.51855800	2.84539500	0.65795800
C	7.44569400	-5.04955000	-0.59017700	C	8.93079600	0.88815200	-0.69064300
C	9.64134200	-3.83167400	0.58865900	C	9.87481000	3.13378100	0.64289200
H	8.30604600	-2.22263500	1.07307500	H	7.82789800	3.48474500	1.19443800
C	8.67720200	-5.68769000	-0.58010100	C	10.28658700	1.17902300	-0.68728700
H	6.59256000	-5.52141500	-1.06279600	H	8.55731100	0.02126000	-1.22228800
C	9.78510800	-5.08452200	0.00388100	C	10.76762000	2.30316600	-0.02521000
H	10.49260000	-3.34824100	1.05564100	H	10.23674200	4.00913900	1.17077000
H	8.77136900	-6.66178100	-1.04775800	H	10.97030300	0.52707100	-1.21961100

H	10.74704500	-5.58388800	0.00330500	H	11.82760600	2.52953000	-0.02974500
C	3.79273300	7.29643200	-0.00517700	C	-1.71918700	8.03320900	0.01351800
C	3.19187900	8.41068100	-0.60011100	C	-2.84473500	8.51800800	-0.65946000
C	5.04953900	7.44567800	0.59038700	C	-0.88910400	8.93122600	0.69117300
C	3.83182000	9.64125400	-0.58874800	C	-3.13321200	9.87425300	-0.64561200
H	2.22280100	8.30595400	-1.07320500	H	-3.48339200	7.82697200	-1.19627600
C	5.68770200	8.67717200	0.58027100	C	-1.18004500	10.28700000	0.68659500
H	5.52133800	6.59257700	1.06313100	H	-0.02280900	8.55814500	1.22407400
C	5.08461600	9.78504300	-0.00386400	C	-2.30343100	10.76754300	0.02288400
H	3.34844800	10.49248200	-1.05584600	H	-4.00797200	10.23579000	-1.17475300
H	6.66174800	8.77135900	1.04801500	H	-0.52875100	10.97109700	1.21923600
H	5.58400300	10.74696900	-0.00332500	H	-2.52985700	11.82752000	0.02647000
C	-3.79269000	-7.29645700	-0.00542500	C	1.71914700	-8.03316400	0.01417100
C	-5.04967200	-7.44563900	0.58978400	C	0.88752500	-8.93153100	0.68946600
C	-3.19164500	-8.41076300	-0.60004800	C	2.84641300	-8.51754800	-0.65621600
C	-5.68782000	-8.67713800	0.57963200	C	1.17862100	-10.28727300	0.68503600
H	-5.52161600	-6.59247500	1.06227200	H	0.01990000	-8.55874800	1.22041000
C	-3.83157600	-9.64134400	-0.58871900	C	3.13500300	-9.87376800	-0.64222400
H	-2.22242700	-8.30607700	-1.07286600	H	3.48634000	-7.82620000	-1.19112200
C	-5.08454100	-9.78507400	-0.00418700	C	2.30367800	-10.76741900	0.02386800
H	-6.66200400	-8.77128400	1.04709900	H	0.52610900	-10.97165800	1.21581200
H	-3.34805700	-10.49262500	-1.05557000	H	4.01109800	-10.23499800	-1.16936100
H	-5.58391400	-10.74700800	-0.00366900	H	2.53020600	-11.82737400	0.02756600
C	-7.29644800	3.79273600	0.00529000	C	-8.03325100	-1.71921500	-0.01443000
C	-8.41080500	3.19167500	0.59981100	C	-8.51766800	-2.84465800	0.65899500
C	-7.44557500	5.04975000	-0.58985700	C	-8.93161300	-0.88941400	-0.69197800
C	-9.64137400	3.83162200	0.58844400	C	-9.87388700	-3.13328000	0.64574800
H	-8.30616000	2.22242700	1.07257700	H	-7.82635400	-3.48311700	1.19569000
C	-8.67706800	5.68791800	-0.57974100	C	-10.28735300	-1.18049900	-0.68680200
H	-6.59237900	5.52171300	-1.06226900	H	-8.55882800	-0.02323000	-1.22526400
C	-9.78504800	5.08462600	0.00397400	C	-10.76752300	-2.30376300	-0.02261200
H	-10.49269200	3.34809000	1.05521400	H	-10.23512900	-4.00794600	1.17524300
H	-8.77116500	6.66213000	-1.04715800	H	-10.97172300	-0.52942300	-1.21935700
H	-10.74697200	5.58401600	0.00343300	H	-11.82747700	-2.53030200	-0.02573200
N	-3.14482300	-6.04954000	-0.00561100	N	1.42455400	-6.65660800	0.00909200
N	-6.04955300	3.14482900	0.00552700	N	-6.65670100	-1.42456500	-0.01007700
N	3.14483300	6.04954000	-0.00540900	N	-1.42463600	6.65662200	0.00855300
N	6.04956300	-3.14482100	0.00553500	N	6.65667500	1.42464600	-0.00721400

Structure S58. The coordinates of both the optimized structures of B5L$_3$ at S$_0$ and S$_1$ in toluene.

S$_0$-optimized structure				S$_1$-optimized structure			
H	2.79274500	3.51771600	-0.13756300	H	-2.77852400	3.54363000	0.13735700
C	6.92007500	4.53069100	2.17256100	C	-6.97611100	4.59769600	-2.02675800
C	6.87650100	3.47302700	1.24306700	C	-6.90301400	3.51674900	-1.12354100
C	5.73017100	3.35422300	0.43522400	C	-5.73152600	3.38510500	-0.35187400
C	4.70993200	4.28331200	0.52253400	C	-4.71635400	4.31712200	-0.45019500
C	4.79087900	5.33766700	1.44561800	C	-4.82595700	5.39182600	-1.34903200
C	5.88983600	5.45044500	2.29331600	C	-5.95245800	5.52125400	-2.15923700
H	7.78847700	4.62825900	2.81512300	H	-7.86425300	4.70748900	-2.63951100
H	5.66834600	2.54460600	-0.28505000	H	-5.64751200	2.56229600	0.35111500
H	5.95288700	6.25274700	3.02070900	H	-6.03884100	6.33901200	-2.86669900
C	3.43975300	4.39130100	-0.27728300	C	-3.42284600	4.41257300	0.31355800
H	3.63769200	4.46239200	-1.35238000	H	-3.58901100	4.46216600	1.39519000
C	2.81323700	5.64818300	0.26328900	C	-2.81462900	5.68129200	-0.22094600
C	1.64548500	6.28506900	-0.11592200	C	-1.63837400	6.31407100	0.13519100
C	1.26953100	7.50110400	0.48319700	C	-1.28004400	7.54098700	-0.45514600
C	2.09506700	8.01771200	1.50078100	C	-2.13697800	8.07269000	-1.44024200
C	3.24707300	7.36926700	1.91859200	C	-3.30026600	7.43139500	-1.83349500
C	3.61516800	6.18751800	1.28193800	C	-3.64897000	6.23770600	-1.20576600
H	1.02654700	5.86440800	-0.90251000	H	-0.99726700	5.88258500	0.89774500
H	1.81715900	8.95225500	1.97607400	H	-1.87294100	9.01435900	-1.90909600
H	3.85678700	7.78922200	2.71141300	H	-3.93386600	7.86360700	-2.60054800
H	-3.63711300	4.46290800	1.35242000	H	3.58905700	4.46211800	-1.39518900
C	-2.09400500	8.01796500	-1.50080800	C	2.13705600	8.07267800	1.44021300
C	-1.26854000	7.50126600	-0.48321200	C	1.28011900	7.54097700	0.45511900
C	-1.64465800	6.28529300	0.11593100	C	1.63843700	6.31405300	-0.13520800
C	-2.81249500	5.64855600	-0.26327000	C	2.81468500	5.68126400	0.22093600
C	-3.61435200	6.18797900	-1.28193100	C	3.64902900	6.23767600	1.20575300
C	-3.24609700	7.36966600	-1.91860800	C	3.30033700	7.43137400	1.83347300
H	-1.81597000	8.95246000	-1.97611900	H	1.87302800	9.01435300	1.90906000
H	-1.02577800	5.86456500	0.90252700	H	0.99732700	5.88256800	-0.89776100
H	-3.85575300	7.78968700	-2.71143900	H	3.93393900	7.86358400	2.60052400
C	-3.43918000	4.39176900	0.27732500	C	3.42289000	4.41253400	-0.31355700
H	-2.79228900	3.51809500	0.13762400	H	2.77855800	3.54359900	-0.13735200
C	-4.70937100	4.28393400	-0.52249400	C	4.71639400	4.31707500	0.45020000
C	-5.72973300	3.35498200	-0.43517000	C	5.73155700	3.38504700	0.35188800
C	-6.87604500	3.47392100	-1.24302000	C	6.90304500	3.51668500	1.12355700
C	-6.91947500	4.53157300	-2.17253400	C	6.97615100	4.59763700	2.02676600
C	-5.88911400	5.45118900	-2.29330300	C	5.95250700	5.52120600	2.15923600
C	-4.79017500	5.33828100	-1.44559900	C	4.82600600	5.39178500	1.34902900
H	-5.66801800	2.54537000	0.28512000	H	5.64753600	2.56223400	-0.35109500
H	-7.78786100	4.62924200	-2.81510300	H	7.86429200	4.70742500	2.63952100
H	-5.95205500	6.25348400	-3.02071200	H	6.03889700	6.33896900	2.86669200
B	-8.08231200	2.50513100	-1.02734900	B	8.10308200	2.54235100	0.90605900
B	8.08264000	2.50407600	1.02740700	B	-8.10306100	2.54243000	-0.90603300
H	4.57065700	-1.71950000	0.93688900	H	-4.52449200	-1.62632300	-0.83576400
C	8.60397500	0.53572600	-0.54887100	C	-8.66473800	0.49286500	0.55648900
C	7.82525100	1.05076700	0.50366500	C	-7.84266000	1.07559000	-0.43295800
C	6.80280700	0.24249800	1.03074700	C	-6.78180800	0.30494900	-0.95129000
C	6.58907000	-1.02891400	0.52832800	C	-6.57122900	-0.98329300	-0.50420700
C	7.34136800	-1.49136900	-0.56330300	C	-7.37386500	-1.51795800	0.52600200
C	8.35470600	-0.70911700	-1.10716400	C	-8.42841100	-0.77275400	1.05974600
H	9.40723000	1.14297400	-0.95280000	H	-9.49276300	1.07125600	0.95184500
H	6.18894100	0.61900400	1.84374800	H	-6.14027000	0.72971700	-1.71739500
H	8.94572100	-1.06485100	-1.94440100	H	-9.05427800	-1.17985900	1.84641200
C	5.60380000	-2.08325500	0.95921900	C	-5.54895500	-2.00069500	-0.93660500
H	5.79401800	-2.41590800	1.98605400	H	-5.67716200	-2.27831700	-1.98891500
C	5.83444600	-3.18439000	-0.04248800	C	-5.81910800	-3.15767800	-0.01072800
C	5.19265600	-4.40106700	-0.18231900	C	-5.17826800	-4.37250400	0.09184800
C	5.53037600	-5.27419500	-1.23522300	C	-5.56032600	-5.31453500	1.07792800
C	6.55563600	-4.86903900	-2.11381600	C	-6.64974700	-4.96255400	1.91729100
C	7.21818800	-3.65943200	-1.97770000	C	-7.31297900	-3.75732400	1.81677700
C	6.85307300	-2.81441800	-0.93553600	C	-6.89598200	-2.84179900	0.84612600
H	4.42579400	-4.69083100	0.52853600	H	-4.37779900	-4.61688500	-0.59812500
H	6.82750900	-5.52250600	-2.93483400	H	-6.96053800	-5.66708300	2.67982300
H	7.99794200	-3.37809800	-2.67733100	H	-8.13336400	-3.52225500	2.48620700
H	-5.79426400	-2.41512500	-1.98597200	H	5.67710000	-2.27836100	1.98891100
C	-6.55628500	-4.86819200	2.11386100	C	6.64971600	-4.96262100	-1.91727000
C	-5.53105100	-5.27346600	1.23529300	C	5.56027600	-5.31458500	-1.07792500
C	-5.19319700	-4.40037100	0.18240400	C	5.17821400	-4.37254600	-0.09185400
C	-5.83483600	-3.18361500	0.04256400	C	5.81906800	-3.15772900	0.01073000
C	-6.85344200	-2.81352500	0.93558900	C	6.89596000	-2.84186600	-0.84610800
C	-7.21868700	-3.65850300	1.97773600	C	7.31296100	-3.75739900	-1.81674800
H	-6.82826000	-5.52163100	2.93486700	H	6.96051100	-5.66715700	-2.67979400
H	-4.42635100	-4.69022300	-0.52843300	H	4.37772900	-4.61691500	0.59810600
H	-7.99842500	-3.37708000	2.67734900	H	8.13336200	-3.52234300	-2.48616600
C	-5.60403100	-2.08250100	-0.95912900	C	5.54891500	-2.00074000	0.93659900
H	-4.57084500	-1.71887200	-0.93677000	H	4.52445900	-1.62635400	0.83574000
C	-6.58918500	-1.02804400	-0.52825600	C	6.57121000	-0.98335200	0.50421600
C	-6.80275500	0.24339700	-1.03067300	C	6.78179800	0.30488800	0.95130000
C	-7.82511300	1.05178700	-0.50361000	C	7.84266800	1.07551400	0.43298300
C	-8.60392500	0.53683500	0.54890400	C	8.66475500	0.49277700	-0.55644900
C	-8.35482100	-0.70804200	1.10719400	C	8.42842000	-0.77284100	-1.05970700
C	-7.34156600	-1.49041500	0.56335300	C	7.37385600	-1.51803000	-0.52597900
H	-6.18882300	0.61983400	-1.84365700	H	6.14025300	0.72966600	1.71739300
H	-9.40711500	1.14417900	0.95281800	H	9.49279500	1.07115500	-0.95179300
H	-8.94590000	-1.06371000	1.94441500	H	9.05429500	-1.17995600	-1.84636100
B	4.79782000	-6.63370600	-1.48913400	B	-4.84870600	-6.68666300	1.28903600
B	-4.79866400	-6.63306600	1.48921400	B	4.84864300	-6.68670600	-1.28904000
H	-0.27564300	-4.53392200	-0.83373700	H	0.23821200	-4.56475000	0.84551100
C	2.87392200	-8.21457100	-0.83600500	C	-2.88971300	-8.26787400	0.72246400
C	3.32380300	-6.89216700	-1.02855700	C	-3.36564200	-6.93400800	0.88914100
C	2.40597000	-5.85116600	-0.78609400	C	-2.42748400	-5.88676100	0.68185200
C	1.11923200	-6.12949200	-0.36285200	C	-1.13400800	-6.15990800	0.31322300
C	0.69828900	-7.45980100	-0.20785100	C	-0.68993400	-7.50336200	0.17619000

C	1.58019700	-8.51042000	-0.43788600	C	-1.59203500	-8.56349500	0.38271600
H	3.56746500	-9.03084600	-1.00173800	H	-3.59051900	-9.08211900	0.86278900
H	2.70911000	-4.82190300	-0.94549600	H	-2.73712400	-4.85889200	0.83275900
H	1.26754400	-9.54067300	-0.30552900	H	-1.27149700	-9.59277500	0.26535700
C	-0.00032000	-5.18943500	0.00006000	C	-0.00002000	-5.21928300	0.00001700
H	0.27509800	-4.53398900	0.83387800	H	-0.23824100	-4.56472600	-0.84546100
C	-1.12000600	-6.12934300	0.36294100	C	1.13395500	-6.15991800	-0.31321300
C	-2.40670400	-5.85084500	0.78619300	C	2.42743400	-5.88678100	-0.68183500
C	-3.32468600	-6.89172300	1.02862200	C	3.36557600	-6.93403800	-0.88915000
C	-2.87499500	-8.21418500	0.83602800	C	2.88962800	-8.26790100	-0.72250400
C	-1.58131400	-8.51020700	0.43789800	C	1.59194500	-8.56351000	-0.38276200
C	-0.69925500	-7.45970700	0.20789700	C	0.68986000	-7.50336800	-0.17621200
H	-2.70969600	-4.82154400	0.94562900	H	2.73709000	-4.85891300	-0.83271700
H	-3.56865600	-9.03036600	1.00173700	H	3.59042100	-9.08215300	-0.86285000
H	-1.26880800	-9.54050100	0.30550900	H	1.27139200	-9.59278700	-0.26542800
C	0.00065400	9.84297500	-0.00003100	C	0.00005100	9.88088900	-0.00002600
C	-1.17115200	10.57485200	0.25304200	C	1.16205300	10.61328500	-0.29413500
C	1.17256000	10.57468700	-0.25312000	C	-1.16194100	10.61330200	0.29407400
C	-1.17191100	11.96306300	0.26886000	C	1.16254700	12.00153700	-0.30940800
H	-2.09480700	10.04270200	0.45499000	H	2.07811000	10.08142500	-0.52900300
C	1.17350900	11.96289800	-0.26896800	C	-1.16241900	12.00155400	0.30933200
H	2.09614300	10.04240800	-0.45505700	H	-2.07800500	10.08145600	0.52894900
C	0.00084600	12.66002100	-0.00006100	C	0.00006800	12.69891100	-0.00004200
H	-2.08763400	12.50302200	0.48448000	H	2.07040700	12.54124600	-0.55671400
H	2.08930500	12.50272700	-0.48459900	H	-2.07027200	12.54127700	0.55663300
H	0.00092000	13.74486200	-0.00007300	H	0.00007500	13.78374200	-0.00004800
C	9.55343400	2.98443300	1.25903000	C	-9.57713100	3.03440100	-1.09816300
C	10.54989900	2.07594200	1.65282000	C	-10.58264600	2.15150400	-1.52683700
C	9.94016200	4.32203600	1.07409600	C	-9.95735600	4.36278400	-0.84356700
C	11.85800000	2.48315800	1.87556200	C	-11.89191900	2.57369100	-1.71211700
H	10.28722600	1.03306400	1.79639600	H	-10.32655000	1.11672600	-1.72957800
C	11.25238500	4.73182300	1.26770400	C	-11.26984600	4.78666200	-1.00179000
H	9.20082100	5.04893500	0.75523800	H	-9.21153600	5.07090100	-0.49833800
C	12.21235900	3.81320300	1.67768900	C	-12.23919800	3.89337200	-1.44449100
H	12.60405500	1.76381200	2.19556600	H	-12.64412300	1.87349200	-2.05928900
H	11.52757200	5.76790300	1.10230600	H	-11.53797500	5.81468000	-0.78280000
H	13.23636200	4.13275600	1.83938400	H	-13.26359200	4.22434700	-1.57812500
C	5.56743300	-7.77201200	-2.25097200	C	-5.66998200	-7.85776900	1.95013300
C	6.91024800	-8.06186600	-1.96193900	C	-6.98604100	-8.14067800	1.54795100
C	4.94699600	-8.53791700	-3.25048800	C	-5.13291300	-8.66167400	2.96920000
C	7.59366300	-9.07573900	-2.62049900	C	-7.71864800	-9.17533400	2.11532900
H	7.42405200	-7.48707000	-1.19810400	H	-7.43994800	-7.54096300	0.76515600
C	5.63355700	-9.53082400	-3.93729600	C	-5.86609600	-9.68394400	3.55804800
H	3.90964200	-8.34195000	-3.50200400	H	-4.12155200	-8.47235600	3.31554700
C	6.95767500	-9.80781500	-3.61675300	C	-7.16161400	-9.94848800	3.12775200
H	8.62557200	-9.28981400	-2.36347900	H	-8.72768800	-9.37642700	1.77089200
H	5.13399900	-10.09442300	-4.71801700	H	-5.42570300	-10.27748700	4.35240900
H	7.49262100	-10.59164000	-4.14228700	H	-7.73381900	-10.75165100	3.57962800
C	-5.56847200	-7.77132500	2.25092500	C	5.66990700	-7.85781300	-1.95015000
C	-6.91131900	-8.06095800	1.96181900	C	6.98597300	-8.14072200	-1.54799000
C	-4.94818200	-8.53740800	3.25039600	C	5.13282000	-8.66171600	-2.96920800
C	-7.59490700	-9.07478600	2.62026800	C	7.71856900	-9.17537900	-2.11538000
H	-7.42501200	-7.48602000	1.19801600	H	7.43989400	-7.54100600	-0.76520400
C	-5.63491400	-9.53027300	3.93709400	C	5.86599200	-9.68398800	-3.55806700
H	-3.91080800	-8.34161600	3.50196500	H	4.12145300	-8.47239800	-3.31553800
C	-6.95906200	-9.80704200	3.61648100	C	7.16151700	-9.94853300	-3.12779300
H	-8.62683800	-9.28868700	2.36319300	H	8.72761500	-9.37647200	-1.77096000
H	-5.13546600	-10.09401400	4.71778400	H	5.42558600	-10.27753100	-4.35242100
H	-7.49414200	-10.59083300	4.14192900	H	7.73371300	-10.75169700	-3.57967800
C	-9.55304100	2.98566700	-1.25901600	C	9.57715600	3.03430500	1.09820400
C	-9.93961000	4.32331800	-1.07410100	C	9.95739600	4.36268500	0.84362200
C	-10.54960600	2.07729500	-1.65282500	C	10.58265800	2.15139300	1.52687800
C	-11.25177800	4.73326400	-1.26774700	C	11.26989000	4.78654800	1.00185800
H	-9.20018900	5.05013000	-0.75522800	H	9.21158700	5.07081400	0.49839500
C	-11.85765200	2.48467000	-1.87560400	C	11.89193400	2.57356300	1.71217100
H	-10.28705700	1.03438300	-1.79638600	H	10.32654900	1.11661600	1.72960700
C	-12.21185300	3.81475900	-1.67775100	C	12.23922900	3.89324300	1.44455800
H	-11.52684200	5.76938000	-1.10236300	H	11.53803300	5.81456400	0.78288000
H	-12.60378600	1.76541300	-2.19562300	H	12.64412800	1.87335300	2.05934100
H	-13.23581300	4.13443600	-1.83947500	H	13.26362600	4.22420500	1.57820200
B	0.00054800	8.27684100	-0.00001500	B	0.00004200	8.31390100	-0.00001800

Structure S59. The coordinates of both the optimized structures of B4N1L$_3$ at S$_0$ and S$_1$ in toluene.

S_0-optimized structure				S_1-optimized structure			
H	-2.74137600	3.52331300	0.28357400	H	2.65780200	3.45674400	-0.11863300
C	-6.77582900	4.50303700	-2.20410500	C	6.80246100	4.58381300	2.12532200
C	-6.77636900	3.47363300	-1.24309100	C	6.77103300	3.49226300	1.22022100
C	-5.65832500	3.36976000	-0.39268700	C	5.60555400	3.34030600	0.43092100
C	-4.62601600	4.28414200	-0.47424700	C	4.57739700	4.25204900	0.50840400
C	-4.65777500	5.30635400	-1.43710800	C	4.64744000	5.33970700	1.40873500
C	-5.72821500	5.40388300	-2.32268400	C	5.76757000	5.48842900	2.23939900
H	-7.62188300	4.59178400	-2.87732400	H	7.67535600	4.70624200	2.75726200
H	-5.62985500	2.58242900	0.35412700	H	5.54927700	2.51599700	-0.27273500
H	-5.75717900	6.18152900	-3.07860800	H	5.82877500	6.30677700	2.94912200
C	-3.38422800	4.40684300	0.36850400	C	3.30062500	4.32803900	-0.28727500
H	-3.61964000	4.51876900	1.43242500	H	3.48804100	4.37333600	-1.36521700
C	-2.72020300	5.63444600	-0.19459800	C	2.66505500	5.59216200	0.22570200
C	-1.56959400	6.27153600	0.22985600	C	1.51223900	6.21068000	-0.18443500
C	-1.14735000	7.43190700	-0.43124600	C	1.14113500	7.43527800	0.41975600
C	-1.88810000	7.91823500	-1.51862900	C	1.93825100	7.98354500	1.44972100
C	-3.04956900	7.28644800	-1.93046800	C	3.09173000	7.35624100	1.85902200
C	-3.47439200	6.14081700	-1.26494100	C	3.47944200	6.15676300	1.24022300
H	-1.00344800	5.89758000	1.07546200	H	0.91149000	5.80483500	-0.98981800
H	-1.54469900	8.80706300	-2.03380500	H	1.61994400	8.90020500	1.93028800
H	-3.61073900	7.68718800	-2.76759000	H	3.68872900	7.78968400	2.65270400
H	3.61960200	4.51881800	-1.43243400	H	-3.48905300	4.37250700	1.36515400
C	1.88801000	7.91826200	1.51861500	C	-1.94010200	7.98315500	-1.44968400
C	1.14726800	7.43192500	0.43123100	C	-1.14285200	7.43503800	-0.41974200
C	1.56952900	6.27156000	-0.22987100	C	-1.51367600	6.21033900	0.18441700
C	2.72014600	5.63448400	0.19458600	C	-2.66635300	5.59157000	-0.22573100
C	3.47432600	6.14086500	1.26493000	C	-3.48087500	6.15601600	-1.24022900
C	3.04948600	7.28649000	1.93045600	C	-3.09344200	7.35560100	-1.85899300
H	1.54459600	8.80708500	2.03379200	H	-1.62200600	8.89990000	-1.93022900
H	1.00339000	5.89759700	-1.07547800	H	-0.91282900	5.80460600	0.98978300
H	3.61064900	7.68723700	2.76758000	H	-3.69054600	7.78893400	-2.65265800
C	3.38418600	4.40688900	-0.36851400	C	-3.30163000	4.32728500	0.28721000
H	2.74134300	3.52335200	-0.28358800	H	-2.65860600	3.45614400	0.11854100
C	4.62597200	4.28420100	0.47424200	C	-4.57838700	4.25102500	-0.50846700
C	5.65829000	3.36982900	0.39268800	C	-5.60632900	3.33903800	-0.43101200
C	6.77633000	3.47371500	1.24309600	C	-6.77184500	3.49074900	-1.22030400
C	6.77577600	4.50312100	2.20410800	C	-6.80353200	4.58232200	-2.12536800
C	5.72815400	5.40395700	2.32268100	C	-5.76885200	5.48718300	-2.23941800
C	4.65771700	5.30641600	1.43710100	C	-4.64868600	5.33869500	-1.40876200
H	5.62983000	2.58249600	-0.35412300	H	-5.54985800	2.51471700	0.27261400
H	7.62182700	4.59187800	2.87733000	H	-7.67645800	4.70456900	-2.75730000
H	5.75710700	6.18160500	3.07860400	H	-5.83025100	6.30554200	-2.94911300
B	7.99318500	2.51799000	1.04418700	B	-7.98662700	2.53850800	-1.02472500
B	-7.99321600	2.51790000	-1.04417200	B	7.98604100	2.54031700	1.02461200
H	-4.52434600	-1.73367300	-0.91982400	H	4.49345700	-1.70070500	0.86204900
C	-8.54060400	0.56259700	0.54969200	C	8.57811000	0.54261500	-0.50577100
C	-7.75314500	1.06792600	-0.50075600	C	7.75335000	1.08326200	0.50227900
C	-6.73595100	0.24942800	-1.02135500	C	6.71320000	0.27356700	0.99978900
C	-6.53672100	-1.02323800	-0.51638900	C	6.52295800	-1.00593000	0.51273100
C	-7.29677100	-1.47681700	0.57346100	C	7.32124400	-1.49049500	-0.53773300
C	-8.30451200	-0.68332100	1.11196000	C	8.35798300	-0.71283500	-1.04886400
H	-9.34063800	1.17760100	0.94853300	H	9.39549600	1.14493500	-0.88849700
H	-6.11487000	0.61886400	-1.83210500	H	6.06959400	0.65865900	1.78554100
H	-8.90196300	-1.03153900	1.94783500	H	8.98651400	-1.08188700	-1.85271000
C	-5.56097000	-2.08726300	-0.94405600	C	5.52734800	-2.05706400	0.92863300
H	-5.75226500	-2.41903400	-1.97099000	H	5.68225100	-2.36823800	1.96814900
C	-5.80458900	-3.18460900	0.05828700	C	5.79937400	-3.17684700	-0.04168100
C	-5.17347400	-4.40624000	0.20075300	C	5.16709800	-4.39668800	-0.18080200
C	-5.51786900	-5.27417500	1.25575400	C	5.53823000	-5.28890600	-1.20867500
C	-6.54127600	-4.85917000	2.13189800	C	6.59489300	-4.89496200	-2.05646200
C	-7.19470600	-3.64480100	1.99193900	C	7.25356600	-3.68432400	-1.91616000
C	-6.82143700	-2.80405200	0.94903500	C	6.85137200	-2.81821100	-0.90312600
H	-4.40880100	-4.70344200	-0.50929700	H	4.37961800	-4.67566300	0.51141500
H	-6.81865500	-5.50823800	2.95461700	H	6.89383800	-5.55956200	-2.85901200
H	-7.97312400	-3.35614500	2.69009500	H	8.05777600	-3.41514100	-2.59264300
H	5.75227300	-2.41896000	1.97099200	H	-5.68174400	-2.36954800	-1.96817600
C	6.54134400	-4.85910000	-2.13188200	C	-6.59372500	-4.89636900	2.05652400
C	5.51793600	-5.27411400	-1.25574400	C	-5.53699000	-5.29009600	1.20872600
C	5.17352200	-4.40618000	-0.20074900	C	-5.16608500	-4.39782300	0.18081900
C	5.80462200	-3.18454100	-0.05828300	C	-5.79863900	-3.17812900	0.04167800
C	6.82147200	-2.80397400	-0.94902400	C	-6.85069900	-2.81970800	0.90313700
C	7.19475900	-3.64472300	-1.99192300	C	-7.25267500	-3.68588400	1.91620400
H	6.81873700	-5.50816700	-2.95459700	H	-6.89250200	-5.56101400	2.85909900
H	4.40884600	-4.70338800	0.50929500	H	-4.37856000	-4.67664000	-0.51141100
H	7.97317700	-3.35605900	-2.69007400	H	-8.05693200	-3.41686500	2.59269600
C	5.56098300	-2.08719400	0.94405500	C	-5.52688800	-2.05831100	-0.92867300
H	4.52435500	-1.73361600	0.91981400	H	-4.49307700	-1.70171600	-0.86212100
C	6.53672600	-1.02316000	0.51639400	C	-6.52272700	-1.00739100	-0.51277700
C	6.73594000	0.24950900	1.02135800	C	-6.71327100	0.27205000	-0.99986600
C	7.75312900	1.06801600	0.50076500	C	-7.75359400	1.08152200	-0.50235600
C	8.54060000	0.56269400	-0.54967800	C	-8.57820900	0.54071400	0.50572700
C	8.30452400	-0.68322800	-1.11194500	C	-8.35778600	-0.71467000	1.04885000
C	7.29678800	-1.47673400	-0.57345000	C	-7.32088100	-1.49210900	0.53771800
H	6.11485000	0.61893900	1.83210400	H	-6.06976800	0.65726700	-1.78564200
H	9.34063000	1.17770500	-0.94851400	H	-9.39572300	1.14286000	0.88845500
H	8.90198400	-1.03144100	-1.94781600	H	-8.98621500	-1.08384300	1.85272000
B	-4.79016700	-6.63528900	1.51448600	B	4.80770200	-6.64469100	-1.47082900
B	4.79025200	-6.63523700	-1.51447600	B	-4.80615100	-6.64571000	1.47090300
H	0.28209700	-4.54196400	0.83160600	H	-0.27580300	-4.55204200	-0.83344400
C	-2.86978900	-8.22054300	0.85625000	C	2.88006300	-8.22839600	-0.82783200
C	-3.31753500	-6.89774600	1.05098700	C	3.32890600	-6.90569700	-1.02144800
C	-2.39965900	-5.85798100	0.80389100	C	2.40618300	-5.86729500	-0.78648200
C	-1.11636500	-6.13741700	0.37163800	C	1.12000400	-6.14735000	-0.36330500
C	-0.69725700	-7.46791900	0.21325000	C	0.70009900	-7.47783600	-0.20685600

C	-1.57866400	-8.51759800	0.45001400	C	1.58511800	-8.52652000	-0.43426700
H	-3.56306100	-9.03625500	1.02611100	H	3.57571000	-9.04382000	-0.98935800
H	-2.70021000	-4.82840600	0.96575900	H	2.70545000	-4.83772400	-0.95025700
H	-1.26794100	-9.54822000	0.31591800	H	1.27446300	-9.55746500	-0.30211600
C	0.00004100	-5.19743100	0.00002300	C	0.00063700	-5.20749400	0.00007800
H	-0.28201900	-4.54195000	-0.83154700	H	0.27694900	-4.55203400	0.83363600
C	1.11645100	-6.13740500	-0.37160900	C	-1.11854800	-6.14758800	0.36341000
C	2.39974300	-5.85795500	-0.80385900	C	-2.40478200	-5.86780600	0.78660100
C	3.31762300	-6.89771100	-1.05097700	C	-3.32730400	-6.90640000	1.02150900
C	2.86988400	-8.22051400	-0.85626100	C	-2.87820100	-8.22900100	0.82782500
C	1.57876100	-8.51758200	-0.45002800	C	-1.58319700	-8.52685200	0.43424500
C	0.69734900	-7.46791200	-0.21324500	C	-0.69838300	-7.47798300	0.20689000
H	2.70028900	-4.82837600	-0.96570800	H	-2.70424800	-4.83830200	0.95043500
H	3.56316000	-9.03621900	-1.02613700	H	-3.57369000	-9.04456900	0.98930600
H	1.26804300	-9.54820800	-0.31595000	H	-1.27234200	-9.55772900	0.30204100
C	-0.00006400	9.53814000	0.00000200	C	-0.00107700	9.52693700	0.00001300
C	1.09957600	10.24486900	-0.49191000	C	-1.08939900	10.22157100	0.53224000
C	-1.09972300	10.24483200	0.49192400	C	1.08710600	10.22178900	-0.53221300
C	1.09879700	11.63231800	-0.48158800	C	-1.08304000	11.60734500	0.52955200
H	1.95231700	9.70085300	-0.88082400	H	-1.91975600	9.67149100	0.95797400
C	-1.09898100	11.63228100	0.48162300	C	1.08047100	11.60756100	-0.52952800
H	-1.95244900	9.70078700	0.88083000	H	1.91757400	9.67187200	-0.95794400
C	-0.00010200	12.33450300	0.00002300	C	-0.00135500	12.30367400	0.00001200
H	1.96011800	12.16684500	-0.86687500	H	-1.92273600	12.14553400	0.95318400
H	-1.96031700	12.16678000	0.86691700	H	1.92005900	12.14591800	-0.95315900
H	-0.00011600	13.41845600	0.00003000	H	-0.00146400	13.38740000	0.00001200
C	-9.45557800	3.00054200	-1.32045000	C	9.45067900	3.03381500	1.28177100
C	-10.45204400	2.08829200	-1.70543900	C	10.45273100	2.13544100	1.69138200
C	-9.83452400	4.34623100	-1.18444900	C	9.83920100	4.37349700	1.10073000
C	-11.75243100	2.49848100	-1.96487700	C	11.75605300	2.54962300	1.92790900
H	-10.19534300	1.03954400	-1.81232700	H	10.19546800	1.09131900	1.83677200
C	-11.13913700	4.76033800	-1.41647400	C	11.14458100	4.79281000	1.31701700
H	-9.09494900	5.07643000	-0.87383900	H	9.10333500	5.09646900	0.76433600
C	-12.09907600	3.83690100	-1.81562800	C	12.10779200	3.88199800	1.73848900
H	-12.49852300	1.77539600	-2.27630200	H	12.50121000	1.83287300	2.25693600
H	-11.40852500	5.80331800	-1.28913500	H	11.41389600	5.83144700	1.15534400
H	-13.11716500	4.15925000	-2.00658600	H	13.12745600	4.20790200	1.91450200
C	-5.56109000	-7.76914100	2.28122700	C	5.57877700	-7.78581000	-2.22934100
C	-6.90611900	-8.05448800	1.99803200	C	6.91490800	-8.09013600	-1.92484600
C	-4.93939800	-8.53586700	3.27934300	C	4.96448200	-8.54175300	-3.24015400
C	-7.59060900	-9.06478300	2.66096600	C	7.59798700	-9.10604200	-2.58088700
H	-7.42077500	-7.47907500	1.23523800	H	7.42371100	-7.52471000	-1.15070700
C	-5.62678300	-9.52523500	3.97039700	C	5.65054300	-9.53747700	-3.92347900
H	-3.90031200	-8.34330600	3.52630200	H	3.93169900	-8.33604300	-3.50273500
C	-6.95322900	-9.79776800	3.65566400	C	6.96844200	-9.82743100	-3.58891400
H	-8.62434600	-9.27543200	2.40849700	H	8.62473400	-9.33036400	-2.31196500
H	-5.12612300	-10.08961200	4.74985300	H	5.15527900	-10.09312800	-4.71267400
H	-7.48890900	-10.57886100	4.18451500	H	7.50306300	-10.61316300	-4.11199300
C	5.56118400	-7.76907500	-2.28122900	C	-5.57699700	-7.78702300	2.22935800
C	6.90621600	-8.05441400	-1.99803600	C	-6.91304600	-8.09164700	1.92480100
C	4.93949900	-8.53579500	-3.27935400	C	-4.96257100	-8.54284800	3.24017900
C	7.59071300	-9.06469600	-2.66098200	C	-7.59592000	-9.10772300	2.58079200
H	7.42086700	-7.47900500	-1.23523700	H	-7.42194600	-7.52631900	1.15065500
C	5.62689200	-9.52515000	-3.97041900	C	-5.64843400	-9.53874400	3.92345400
H	3.90041100	-8.34324000	-3.52631100	H	-3.92984500	-8.33690900	3.50280700
C	6.95334000	-9.79767600	-3.65568800	C	-6.96625200	-9.82899100	3.58882800
H	8.62445200	-9.27534000	-2.40851500	H	-8.62260500	-9.33227300	2.31182300
H	5.12623700	-10.08952200	-4.74988100	H	-5.15307500	-10.09430000	4.71265700
H	7.48902600	-10.57875900	-4.18454800	H	-7.50071500	-10.61485700	4.11186700
C	9.45554400	3.00065300	1.32044900	C	-9.45138700	3.03169000	-1.28180100
C	9.83446900	4.34634700	1.18443900	C	-9.84019200	4.37128400	-1.10071800
C	10.45202600	2.08841900	1.70543200	C	-10.45326300	2.13310400	-1.69137800
C	11.13907900	4.76047300	1.41645000	C	-11.14567400	4.79031600	-1.31693300
H	9.09488000	5.07653500	0.87383400	H	-9.10446600	5.09441000	-0.76435000
C	11.75241000	2.49862700	1.96485500	C	-11.75668700	2.54700600	-1.92783200
H	10.19534100	1.03966800	1.81232700	H	-10.19578000	1.08904100	-1.83679900
C	12.09903500	3.83705100	1.81559700	C	-12.10870700	3.87930100	-1.73837100
H	11.40845000	5.80345700	1.28910400	H	-11.41520700	5.82889200	-1.15522800
H	12.49851500	1.77555400	2.27627600	H	-12.50170400	1.83009900	-2.25683300
H	13.11712100	4.15941500	2.00654300	H	-13.12845100	4.20498600	-1.91432700
N	-0.00004600	8.12411200	-0.00000800	N	-0.00093200	8.11046400	0.00001100

Structure S60. The coordinates of both the optimized structures of B3N2L$_3$ at S$_0$ and S$_1$ in toluene.

S_0-optimized structure				S_1-optimized structure			
H	-3.35462400	3.05672600	0.55462300	H	2.74612200	-3.38839000	0.17615100
C	-7.19728900	3.22339400	-2.35667100	C	7.01530600	-4.42008500	-1.85773700
C	-7.14948100	2.31982400	-1.28558300	C	6.88079000	-3.33084400	-0.95746100
C	-6.14989700	2.45528000	-0.31378200	C	5.69030300	-3.19519100	-0.20399900
C	-5.23995200	3.48979900	-0.41846500	C	4.70937000	-4.14260800	-0.33090900
C	-5.31155500	4.41289900	-1.47229900	C	4.86105800	-5.24444500	-1.20760400
C	-6.29443800	4.27091600	-2.44738600	C	6.02503000	-5.36212600	-1.98186900
H	-7.96105200	3.10086500	-3.11534600	H	7.90684800	-4.49146700	-2.46623500
H	-6.10998700	1.76111300	0.51790000	H	5.58572300	-2.37122400	0.48988900
H	-6.35980600	4.96434100	-3.27902800	H	6.14572600	-6.17950700	-2.68301500
C	-4.10928900	3.85005600	0.50824400	C	3.39374500	-4.24474700	0.39087400
H	-4.45362600	4.01234900	1.53530700	H	3.52346800	-4.27677800	1.47716200
C	-3.56013900	5.10832000	-0.11000200	C	2.82160700	-5.52740000	-0.14716900
C	-2.54552100	5.92917400	0.34416200	C	1.64902900	-6.15571800	0.18464000
C	-2.22123200	7.07973400	-0.38401000	C	1.33652600	-7.39283300	-0.42307500
C	-2.91805600	7.37623800	-1.56127500	C	2.22689800	-7.94548500	-1.37767700
C	-3.94530900	6.55871800	-2.00741100	C	3.39553400	-7.30646500	-1.71130000
C	-4.27264700	5.41942600	-1.27969400	C	3.71366000	-6.08925700	-1.09265500
H	-2.00979000	5.70575900	1.26034500	H	0.98117900	-5.73763000	0.92736300
H	-2.65043200	8.26483500	-2.12069500	H	1.96383600	-8.87505800	-1.86457800
H	-4.47874000	6.81270700	-2.91704200	H	4.05403800	-7.74098800	-2.45419100
H	2.86561100	4.89221900	-1.37026200	H	-3.53392600	-4.55386900	-1.49256700
C	0.71320700	8.05910300	1.56165900	C	-1.69301100	-7.90671000	1.44628700
C	0.03223500	7.45052900	0.49553700	C	-0.97568500	-7.38774600	0.36231200
C	0.60768500	6.34866800	-0.15080500	C	-1.42374500	-6.23878000	-0.30067000
C	1.84727800	5.89647000	0.25974200	C	-2.58763600	-5.63353500	0.12894300
C	2.53943500	6.52863800	1.30446300	C	-3.31941200	-6.16402300	1.20647500
C	1.96135400	7.61166000	1.96005900	C	-2.86347300	-7.30047400	1.86990200
H	0.25291800	8.89745300	2.07017100	H	-1.32512500	-8.79298600	1.94906800
H	0.08770200	5.87774300	-0.97725100	H	-0.87480400	-5.85835700	-1.15502700
H	2.47216400	8.10556500	2.77965600	H	-3.41233100	-7.71633300	2.70697600
C	2.67404800	4.76967500	-0.29878300	C	-3.29391500	-4.42981000	-0.43111900
H	2.17224400	3.80247000	-0.18100500	H	-2.67908900	-3.52593200	-0.35466100
C	3.93894700	4.85073300	0.51402600	C	-4.53077100	-4.34570900	0.42228100
C	5.09593100	4.10253700	0.41788600	C	-5.59220100	-3.46354300	0.34448000
C	6.20514600	4.38821500	1.23815000	C	-6.69825500	-3.59866200	1.20342700
C	6.06967100	5.42124000	2.18592800	C	-6.66032600	-4.62731000	2.16586800
C	4.90093200	6.15581400	2.31843400	C	-5.58679700	-5.49692600	2.27913900
C	3.83739400	5.88331800	1.46124900	C	-4.52496700	-5.36554500	1.38685000
H	5.17023100	3.30867000	-0.31876300	H	-5.59459000	-2.67850600	-0.40484500
H	6.90723100	5.64805900	2.83705300	H	-7.50135500	-4.74200700	2.84133600
H	4.82885000	6.94230800	3.06228700	H	-5.58845800	-6.27499600	3.03528100
B	7.54842900	3.62604100	1.02125700	B	-7.95290400	-2.67833100	1.00600700
H	-4.22904200	-2.37353100	-1.11543600	H	4.49514600	1.69837300	-1.03288800
C	-8.35028700	-0.42907100	0.53041800	C	8.33367700	-0.64410500	0.78867500
C	-7.70967400	0.04882300	-0.61870700	C	7.63525600	-1.10844100	-0.32901000
C	-6.67090500	-0.68406600	-1.20048100	C	6.69579400	-0.29679500	-0.97727800
C	-6.27953400	-1.87480600	-0.61617600	C	6.46155700	0.97002800	-0.48340100
C	-6.90772700	-2.33887800	0.55030900	C	7.14420700	1.43292100	0.65860000
C	-7.95462000	-1.61973100	1.11843100	C	8.08808300	0.62300500	1.28966100
H	-9.15427900	0.15425600	0.96374600	H	9.05781900	-1.29282500	1.26758300
H	-6.19196200	-0.30828700	-2.09810300	H	6.18113100	-0.66710100	-1.85759500
H	-8.44894300	-1.97000400	2.01796300	H	8.62213100	0.97074700	2.16653200
C	-5.21499900	-2.84647900	-1.05063300	C	5.53516000	2.04044300	-0.99474600
H	-5.42700300	-3.26092200	-2.04275100	H	5.79873500	2.34738300	-2.01321300
C	-5.26673400	-3.89999400	0.02484900	C	5.73064700	3.15392000	0.00173400
C	-4.48900900	-5.03011900	0.19086800	C	5.11877900	4.39090400	0.06781200
C	-4.67304700	-5.87099700	1.30656900	C	5.42200100	5.29347900	1.10888400
C	-5.68978600	-5.52734700	2.21978600	C	6.39516400	4.88357900	2.04879700
C	-6.48216200	-4.40060300	2.06306300	C	7.01998300	3.65018400	1.99488400
C	-6.26575500	-3.58224200	0.95982000	C	6.68199200	2.77488500	0.96407400
H	-3.73147600	-5.27674500	-0.54605000	H	4.40293900	4.67974200	-0.69479100
H	-5.84765100	-6.15919500	3.08654900	H	6.65187000	5.56219300	2.85474800
H	-7.24809000	-4.16229500	2.79342000	H	7.75229000	3.37238200	2.74616900
H	6.05415400	-1.58776200	1.95748700	H	-5.58797300	2.18971300	1.95841600
C	7.17874800	-3.88766000	-2.14653700	C	-6.77510000	4.86024800	-1.88976800
C	6.22394800	-4.44397400	-1.27142200	C	-5.65945900	5.23016900	-1.10380900
C	5.75885200	-3.63424800	-0.21650600	C	-5.22246600	4.29274700	-0.14205600
C	6.21026600	-2.33565000	-0.07262100	C	-5.84134400	3.06517400	-0.01161900
C	7.16151200	-1.81311900	-0.96380100	C	-6.94914200	2.73333300	-0.81127700
C	7.65130300	-2.59202100	-2.00666300	C	-7.41995200	3.64273900	-1.75703000
H	7.54664300	-4.49075800	-2.96885000	H	-7.13427600	5.55850200	-2.63763300
H	5.04275100	-4.03684200	0.49229200	H	-4.39509900	4.54707600	0.51181000
H	8.38005600	-2.19497900	-2.70528000	H	-8.26951100	3.40068400	-2.38764400
C	5.81399600	-1.28569300	0.93162400	C	-5.50539500	1.91365900	0.90080500
H	4.73721600	-1.08489300	0.91102600	H	-4.47923300	1.55937000	0.75231300
C	6.62530300	-0.09207700	0.50271400	C	-6.52855400	0.87546100	0.52015400
C	6.63893200	1.19605400	1.00748100	C	-6.68852900	-0.41707100	0.97664900
C	7.52325700	2.15449300	0.48294300	C	-7.76333800	-1.21155600	0.52432200
C	8.37282500	1.76853200	-0.56990900	C	-8.64782400	-0.64198700	-0.41417800
C	8.32032200	0.50074400	-1.13048900	C	-8.46000000	0.62973100	-0.93116500
C	7.44057600	-0.43091900	-0.58899000	C	-7.39269000	1.39474300	-0.46278900
H	5.97347900	1.47121300	1.82047000	H	-5.99534500	-0.82612300	1.70620400
H	9.07237600	2.49387000	-0.97217800	H	-9.48639000	-1.23346500	-0.76695700
H	8.95952300	0.24273800	-1.96819800	H	-9.13702000	1.02251600	-1.68254900
B	-3.77381000	-7.11887400	1.58983400	B	4.72770700	6.69430800	1.26553600
B	5.69473300	-5.89430200	-1.52739700	B	-4.96984500	6.61910500	-1.33046000
H	0.93834700	-4.38115700	0.75337200	H	-0.30125100	4.54873300	0.67828800
C	-1.67069000	-8.45674000	0.92416100	C	2.78199200	8.27489700	0.67113800
C	-2.29246700	-7.20224500	1.08984800	C	3.27132500	6.95038700	0.82555200
C	-1.53113000	-6.05472300	0.79391300	C	2.34213700	5.90635200	0.56549500
C	-0.22613800	-6.16842800	0.35163500	C	1.04951700	6.17704800	0.18377500
C	0.37017300	-7.43302100	0.22489400	C	0.59260700	7.50933400	0.06655500
C	-0.35667200	-8.58612200	0.50320600	C	1.48321500	8.56367500	0.30698600

H	-2.24262300	-9.35408100	1.13103800	H	3.46709500	9.09906700	0.83627800
H	-1.97035500	-5.07197600	0.92858200	H	2.65428000	4.87527000	0.69645500
H	0.09098200	-9.56799100	0.39254000	H	1.16079200	9.59482900	0.20276000
C	0.74816900	-5.09511900	-0.05575500	C	-0.07444000	5.22818500	-0.15117500
H	0.37618000	-4.51126500	-0.90521900	H	0.17146400	4.59644900	-1.01267600
C	1.98261300	-5.88277700	-0.40658600	C	-1.22570400	6.15828100	-0.44290500
C	3.21707200	-5.44055100	-0.84463100	C	-2.52252200	5.86513500	-0.79459400
C	4.26906900	-6.35042800	-1.06749000	C	-3.48569700	6.89118000	-0.98617800
C	4.00534500	-7.71748700	-0.84505400	C	-3.02170600	8.22249300	-0.82419400
C	2.76623800	-8.17852400	-0.42926700	C	-1.71905900	8.53526000	-0.49218500
C	1.74961900	-7.25378100	-0.21382800	C	-0.79981300	7.49741600	-0.29516800
H	3.37632500	-4.38319200	-1.02740500	H	-2.81286600	4.83054400	-0.94452700
H	4.80381800	-8.43456800	-0.99774400	H	-3.73102200	9.03301000	-0.94990400
H	2.59915800	-9.23838100	-0.26955200	H	-1.41883700	9.57129300	-0.37156200
C	-1.45177600	9.34448700	0.06782300	C	0.11092000	-9.48043200	-0.21340500
C	-0.46713400	10.23818900	-0.36219600	C	-0.97082800	-10.06943200	-0.86761100
C	-2.68880100	9.84560200	0.48149100	C	1.11036100	-10.27872500	0.34382500
C	-0.71526200	11.60320700	-0.36441700	C	-1.04366700	-11.45067500	-0.96766000
H	0.49118100	9.85734600	-0.69492600	H	-1.74368400	-9.44291600	-1.29659900
C	-2.93333500	11.21099800	0.45895200	C	1.03150600	-11.65912000	0.23147500
H	-3.45545700	9.15865500	0.81989400	H	1.93595900	-9.81417200	0.87021600
C	-1.94890600	12.09922900	0.04141100	C	-0.04392500	-12.24925300	-0.42265400
H	0.06009900	12.28237000	-0.70172300	H	-1.88325300	-11.90449200	-1.48122900
H	-3.89905500	11.58233200	0.78404100	H	1.80751600	-12.27538700	0.67055200
H	-2.14121100	13.16590300	0.03148800	H	-0.10537900	-13.32811400	-0.50388900
C	-9.46444200	1.51753900	-1.44567200	C	9.24441700	-2.75422200	-1.09927000
C	-10.25680500	0.48638900	-1.96063800	C	10.04127200	-1.86128600	-1.81671300
C	-10.05616700	2.75793800	-1.18692000	C	9.78729900	-3.95096600	-0.62742200
C	-11.60613900	0.69345000	-2.20511300	C	11.36741500	-2.17491800	-2.07085600
H	-9.80702300	-0.47713400	-2.16927300	H	9.61538800	-0.93162400	-2.17423500
C	-11.40326700	2.95884700	-1.45143300	C	11.11363900	-4.25696400	-0.89319500
H	-9.45847300	3.56232100	-0.77572300	H	9.17310600	-4.62568900	-0.04269800
C	-12.18946400	1.93086700	-1.95841300	C	11.90753200	-3.37277200	-1.61485000
H	-12.20234600	-0.11952600	-2.60499200	H	11.98043000	-1.48067400	-2.63361500
H	-11.84340700	3.92807800	-1.24330800	H	11.53195500	-5.18428400	-0.51917300
H	-13.24262000	2.09169400	-2.15774700	H	12.94521300	-3.61249500	-1.81504400
C	-4.37027700	-8.30789800	2.42440000	C	5.57404000	7.84939100	1.93018000
C	-5.68444300	-8.75507000	2.21448200	C	6.91915200	8.06019800	1.58287700
C	-3.61969800	-8.96479100	3.41236200	C	5.03560400	8.70658900	2.90445700
C	-6.21632400	-9.81518800	2.93683100	C	7.67587700	9.07569300	2.15304400
H	-6.29600500	-8.26753000	1.46210700	H	7.37825400	7.41501400	0.83995300
C	-4.15526100	-10.00420500	4.16172500	C	5.79005700	9.71212700	3.49623300
H	-2.60075000	-8.64486800	3.60484500	H	4.00320800	8.57214900	3.21128100
C	-5.45371200	-10.43761100	3.91870700	C	7.11359300	9.90588300	3.11681800
H	-7.22889600	-10.15122700	2.74064700	H	8.70771100	9.21883800	1.84833000
H	-3.55804100	-10.48162200	4.93121600	H	5.34368500	10.34802500	4.25403500
H	-5.87067100	-11.25752600	4.49370200	H	7.70312700	10.69558400	3.57066000
C	6.61892200	-6.91650900	-2.28053300	C	-5.84704200	7.78164900	-1.94419600
C	7.99199800	-6.99946600	-1.99991000	C	-7.14900600	8.04010500	-1.48513900
C	6.11158700	-7.78258300	-3.26204100	C	-5.37121900	8.60113600	-2.98072100
C	8.81346500	-7.91154600	-2.64941400	C	-7.92488100	9.06403600	-2.01370900
H	8.42005600	-6.34266500	-1.24949500	H	-7.55802100	7.42657900	-0.68825500
C	6.93262400	-8.67461400	-3.93951200	C	-6.14731300	9.61344400	-3.53182300
H	5.05489300	-7.74624000	-3.50659100	H	-4.37121200	8.43247500	-3.36805000
C	8.28556500	-8.74673700	-3.62755500	C	-7.42744200	9.85303200	-3.04510300
H	9.86758500	-7.96602000	-2.39927900	H	-8.92153400	9.24475400	-1.62388800
H	6.51649300	-9.31961300	-4.70601500	H	-5.75154200	10.21923300	-4.34072100
H	8.92701600	-9.45147700	-4.14585600	H	-8.03341200	10.64805900	-3.46697300
C	8.92577700	4.32544500	1.27230000	C	-9.39096200	-3.25679900	1.23173300
C	9.09853200	5.70985400	1.10990400	C	-9.68989500	-4.61365100	1.02277900
C	10.05127300	3.57859900	1.65785200	C	-10.44440700	-2.42830000	1.65420200
C	10.32965900	6.31735900	1.31681900	C	-10.96986500	-5.11578800	1.21437800
H	8.25478800	6.31637900	0.79856000	H	-8.90465300	-5.28334100	0.68782100
C	11.27887400	4.18219400	1.89316000	C	-11.72177800	-2.92695500	1.87083100
H	9.95510000	2.50531400	1.78451500	H	-10.25037300	-1.37476600	1.82573900
C	11.42089100	5.55431400	1.71734800	C	-11.98854300	-4.27298800	1.64579400
H	10.43933900	7.38631900	1.16876400	H	-11.17483400	-6.16507400	1.03000800
H	12.12786500	3.58364000	2.20545900	H	-12.51192300	-2.26577900	2.21033100
H	12.38173800	6.02792200	1.88872100	H	-12.98794300	-4.66395700	1.80476600
N	-1.21050500	7.95372900	0.07548600	N	0.18013300	-8.06688600	-0.09351200
N	-8.10045600	1.28632700	-1.18464300	N	7.89668000	-2.41716000	-0.81846200

Structure S61. The coordinates of both the optimized structures of B2N3L$_3$ at S$_0$ and S$_1$ in toluene.

S_0-optimized structure				S_1-optimized structure			
H	2.99595000	3.35772000	-0.51393200	H	-2.41799700	3.55308400	0.16118300
C	6.88916700	3.84661500	2.29199000	C	-6.53953800	4.93899200	-1.96526500
C	6.89147700	2.94781500	1.21528800	C	-6.52386200	3.87075700	-1.03510700
C	5.85508600	2.99972500	0.27441700	C	-5.36422900	3.64126300	-0.26193000
C	4.86154500	3.94994400	0.41239800	C	-4.28971300	4.48206100	-0.40172600
C	4.88002800	4.86928200	1.47158400	C	-4.31940200	5.56418400	-1.31189300
C	5.89946700	4.80867200	2.41731500	C	-5.45719900	5.77513100	-2.10240400
H	7.68125200	3.78773400	3.02894000	H	-7.41487600	5.08462000	-2.58455000
H	5.85087900	2.30782000	-0.56002700	H	-5.34954200	2.83275100	0.45790900
H	5.92687500	5.49917900	3.25351200	H	-5.48962200	6.58150000	-2.82592100
C	3.67894600	4.21416000	-0.48105100	C	-2.98118300	4.47502000	0.33992400
H	3.97977400	4.41213600	-1.51550600	H	-3.12267400	4.55237300	1.42255000
C	3.03784900	5.41505500	0.16187600	C	-2.27608200	5.67775200	-0.22453300
C	1.94357000	6.14544500	-0.26005800	C	-1.05290200	6.19712900	0.11202200
C	1.53483400	7.25644300	0.48712100	C	-0.60712500	7.37679700	-0.52756900
C	2.23131800	7.60394000	1.65081100	C	-1.41980000	7.97974200	-1.52091400
C	3.33944100	6.87917800	2.06287900	C	-2.64122200	7.44908000	-1.85667600
C	3.75018600	5.77990600	1.31605500	C	-3.09180500	6.29371800	-1.20389000
H	1.40830900	5.88154300	-1.16560900	H	-0.44306400	5.74076800	0.88145700
H	1.89910000	8.45966600	2.22656100	H	-1.05509800	8.85865400	-2.03557200
H	3.87028400	7.17337400	2.96185300	H	-3.23768900	7.92082700	-2.62870400
H	-3.34781500	4.58502600	1.41403900	H	3.96040100	4.02138200	-1.38898200
C	-1.47299100	7.98720900	-1.44304400	C	2.41505000	7.64314900	1.40117700
C	-0.74763300	7.42807100	-0.37950800	C	1.67255000	7.16162400	0.31702600
C	-1.22254500	6.26703900	0.24443500	C	2.01755900	5.94897600	-0.29699500
C	-2.40908200	5.70551600	-0.18744800	C	3.10532200	5.24816700	0.17787300
C	-3.14662500	6.28513700	-1.23177700	C	3.87155800	5.74213200	1.25346100
C	-2.66842500	7.42923300	-1.86334600	C	3.51154200	6.94061400	1.87112000
H	-1.08941200	8.87393100	-1.93285400	H	2.12661500	8.57805900	1.86696800
H	-0.66785500	5.83461100	1.06942500	H	1.45142800	5.59579400	-1.15210500
H	-3.21514900	7.88578700	-2.68135400	H	4.08297600	7.32992000	2.70594400
C	-3.13101200	4.49659100	0.34399000	C	3.69970100	3.96180500	-0.32680800
H	-2.53726000	3.58356700	0.22141000	H	2.99885400	3.12512000	-0.22550800
C	-4.38689400	4.46747400	-0.48611900	C	4.91465200	3.79260900	0.54688000
C	-5.46651600	3.60864400	-0.41834200	C	5.89506600	2.82204700	0.50088000
C	-6.58632200	3.79684000	-1.25225700	C	7.01223000	2.87783600	1.36043800
C	-6.53865900	4.85200500	-2.18403200	C	7.05293600	3.94184000	2.29040900
C	-5.44614200	5.69989700	-2.28728400	C	6.06340800	4.90720700	2.36834600
C	-4.37363900	5.51963100	-1.41689500	C	4.99135100	4.84459200	1.47678900
H	-5.47346100	2.80019500	0.30600700	H	5.83506200	2.02378900	-0.23209400
H	-7.38445500	5.00499300	-2.84594300	H	7.89739900	4.00461100	2.96886000
H	-5.44075500	6.50067800	-3.01926500	H	6.13466800	5.70654100	3.09929300
B	-7.85218400	2.90543900	-1.06232600	B	8.18970900	1.85321900	1.18552400
H	4.47425300	-2.06655700	1.25107100	H	-4.68323200	-1.37665800	-1.11972300
C	8.16479600	0.37216300	-0.72294900	C	-8.17293400	1.39405000	0.80458800
C	7.60761600	0.73764300	0.50583600	C	-7.49092000	1.74874900	-0.36200200
C	6.72344600	-0.12399700	1.16089600	C	-6.67326700	0.82498700	-1.02209700
C	6.39708900	-1.32969500	0.56787600	C	-6.53638100	-0.44060700	-0.48812000
C	6.93635800	-1.68287900	-0.67962800	C	-7.19046200	-0.78817000	0.70820600
C	7.83395500	-0.83502300	-1.32048700	C	-8.02128800	0.12884000	1.34773400
H	8.85220700	1.05418000	-1.21009000	H	-8.80482900	2.12802700	1.29082300
H	6.30909900	0.16803100	2.11988300	H	-6.16940800	1.11262800	-1.93864700
H	8.26278900	-1.09883400	-2.28112600	H	-8.53685900	-0.13108800	2.26523700
C	5.49287100	-2.42542200	1.06765300	C	-5.74563400	-1.61454300	-1.00104400
H	5.84986400	-2.84595000	2.01447800	H	-6.10299800	-1.94336500	-1.98325900
C	5.53769900	-3.43136400	-0.05247500	C	-5.97128500	-2.65675100	0.06263900
C	4.87738400	-4.64069600	-0.16117500	C	-5.45280800	-3.93336800	0.14663000
C	5.01464200	-5.39357100	-1.33549200	C	-5.73877600	-4.71981400	1.27509400
C	5.85334400	-4.92502600	-2.35724000	C	-6.58883200	-4.20111500	2.27023100
C	6.54913600	-3.73459200	-2.22512800	C	-7.13796400	-2.93762300	2.15995900
C	6.38558100	-2.97492900	-1.07270400	C	-6.82179600	-2.14995200	1.05735200
H	4.24819800	-5.00249300	0.64366600	H	-4.81930600	-4.32489600	-0.63965700
H	5.95347900	-5.50578700	-3.26566500	H	-6.80427300	-4.80012400	3.14536300
H	7.19245300	-3.39775300	-3.03083200	H	-7.78449300	-2.56527000	2.94718800
H	-5.86610800	-2.14540000	-2.00656600	H	5.24920500	-2.73632800	1.98283500
C	-6.74424400	-4.53249400	2.10912100	C	6.30012700	-5.47245600	-1.86605700
C	-5.73672900	-4.99171500	1.23696800	C	5.08035100	-5.70275800	-1.17553600
C	-5.36138900	-4.14540200	0.17522200	C	4.72970500	-4.73176300	-0.19682300
C	-5.94365900	-2.90046600	0.02575900	C	5.50060100	-3.61384600	0.01271700
C	-6.94253800	-2.47352000	0.91561900	C	6.70673300	-3.41688800	-0.69879300
C	-7.34927200	-3.29380000	1.96232100	C	7.10608700	-4.37513300	-1.64037600
H	-7.04732200	-5.16581700	2.93517300	H	6.60157100	-6.18314600	-2.62761500
H	-4.60968400	-4.47679700	-0.53351500	H	3.83808200	-4.87929100	0.40281800
H	-8.11511500	-2.97048300	2.65926500	H	8.02249500	-4.24510700	-2.20751200
C	-5.65494000	-1.81889900	-0.98177400	C	5.25877900	-2.43879200	0.92744800
H	-4.60323500	-1.51273400	-0.96381300	H	4.29326000	-1.95922800	0.73173700
C	-6.57950300	-0.71012700	-0.55398200	C	6.42360800	-1.53017100	0.62315800
C	-6.71665000	0.57131600	-1.05728500	C	6.69694700	-0.26642700	1.08970800
C	-7.69019200	1.43931400	-0.53315900	C	7.88950700	0.41220000	0.72137500
C	-8.50139600	0.97095200	0.51633900	C	8.77421600	-0.28675700	-0.13923900
C	-8.32677400	-0.28620400	1.07635500	C	8.48316200	-1.52890100	-0.66707600
C	-7.35874900	-1.12715200	0.53707400	C	7.28946100	-2.16507500	-0.29772200
H	-6.07835100	0.91088200	-1.86776000	H	6.00415100	0.22116600	1.77014700
H	-9.26901300	1.62439900	0.91779200	H	9.69890900	0.20010100	-0.43259000
H	-8.93945500	-0.60532800	1.91282600	H	9.16847700	-2.00167800	-1.36371500
B	-5.05450600	-6.37524800	1.51055100	B	4.19409300	-6.91143700	-1.54929900
H	-0.49598700	-4.41851300	-0.83340300	H	-0.10503100	-4.45923300	0.81786200
C	2.59207100	-8.15156000	-0.80792200	C	-3.69264500	-7.71083100	0.59356300
C	3.03052400	-6.83644600	-1.04357000	C	-3.93245900	-6.36778100	0.91698300
C	2.14178200	-5.77388100	-0.81833600	C	-2.90284500	-5.43038200	0.75188200
C	0.86663000	-6.04112800	-0.35830800	C	-1.68052300	-5.84546400	0.25985100
C	0.42220800	-7.35800400	-0.17027300	C	-1.42748200	-7.20023200	-0.00862800
C	1.29743900	-8.41506900	-0.39651200	C	-2.44659900	-8.13236400	0.15716100
H	3.27968900	-8.97368400	-0.95867200	H	-4.49220900	-8.43281800	0.70349700

H	2.45413500	-4.75373000	-1.00364600	H	-3.06435400	-4.39125100	1.01224900
H	0.98604400	-9.44203200	-0.23859700	H	-2.28380200	-9.18184700	-0.06285400
C	-0.22783200	-5.07227200	0.00389000	C	-0.45798200	-5.02337400	-0.05257800
H	0.07453600	-4.41571800	0.82748600	H	-0.66150900	-4.28755100	-0.83910200
C	-1.36441600	-5.98269800	0.39053000	C	0.54191100	-6.06407900	-0.48769700
C	-2.64203700	-5.66745800	0.80818200	C	1.85773000	-5.90562900	-0.87718000
C	-3.58606800	-6.68139400	1.07329300	C	2.67169600	-7.02139900	-1.16712400
C	-3.16156400	-8.01608600	0.91169600	C	2.05509000	-8.28956700	-1.08523400
C	-1.87470000	-8.34906600	0.51813000	C	0.72422900	-8.46283000	-0.73803200
C	-0.96849500	-7.32501800	0.26041100	C	-0.04052000	-7.34053200	-0.43268200
H	-2.92065600	-4.62812800	0.94541100	H	2.26927700	-4.90670900	-0.96727500
H	-3.87186000	-8.81456000	1.09374900	H	2.65245800	-9.16926700	-1.29744900
H	-1.58744500	-9.38925500	0.40649900	H	0.29647400	-9.45930500	-0.69092000
C	0.54231000	9.44654200	0.09525400	C	0.83057400	9.32854600	-0.37709700
C	-0.52400100	10.22739200	0.54949600	C	1.99406300	9.76490000	-1.01170400
C	1.71821000	10.07838500	-0.31774900	C	-0.09417300	10.25730000	0.10333300
C	-0.41601000	11.61026100	0.57640700	C	2.22098500	11.12317100	-1.17148200
H	-1.43573500	9.74475600	0.88112000	H	2.70826100	9.03827000	-1.37987800
C	1.82282800	11.46098800	-0.27045700	C	0.14076300	11.61367900	-0.06672800
H	2.54760600	9.47960400	-0.67515300	H	-0.98306000	9.91219600	0.61846700
C	0.75709700	12.23657900	0.17126300	C	1.29671900	12.05130400	-0.70326100
H	-1.25337400	12.20063300	0.93207000	H	3.12290200	11.45722400	-1.67099500
H	2.74270300	11.93472500	-0.59541800	H	-0.57684500	12.33123200	0.31409400
H	0.84015700	13.31689900	0.20043000	H	1.47985200	13.11194300	-0.82961400
C	9.26892100	2.32568700	1.30482800	C	-8.94240600	3.53248500	-1.13981200
C	10.16289600	1.33697700	1.73058300	C	-9.84990300	2.70338800	-1.80085600
C	9.75167600	3.62164800	1.09277000	C	-9.34578300	4.79751800	-0.70702500
C	11.50033700	1.64015300	1.93654500	C	-11.14370100	3.14371800	-2.03447000
H	9.80128900	0.32978000	1.90016300	H	-9.53421300	1.72128100	-2.13193800
C	11.08854300	3.91661800	1.31768300	C	-10.63955300	5.23146300	-0.95480600
H	9.07788200	4.39615600	0.74741800	H	-8.64918500	5.42905100	-0.16848200
C	11.97446200	2.93164900	1.73805500	C	-11.54317700	4.40872100	-1.61774100
H	12.17462600	0.85763200	2.26738100	H	-11.84112800	2.49544200	-2.55223800
H	11.44124300	4.92795800	1.14622800	H	-10.94666100	6.21285900	-0.61187000
H	13.01897300	3.16659500	1.90687100	H	-12.55503000	4.74924100	-1.80355100
C	4.98966000	-7.63800700	-2.24869500	C	-5.90058600	-6.94598000	2.22175500
C	6.24109000	-8.09944600	-1.84240200	C	-7.20418200	-7.30424500	1.88054300
C	4.39608100	-8.17393600	-3.39144600	C	-5.29623800	-7.52272400	3.33827600
C	6.89180700	-9.08252100	-2.57518000	C	-7.89777100	-8.22364000	2.65567600
H	6.69753200	-7.68079700	-0.95286100	H	-7.66591400	-6.85812900	1.00713400
C	5.04547200	-9.16772900	-4.11085000	C	-5.99053000	-8.45184600	4.10047800
H	3.42566100	-7.80929900	-3.70797500	H	-4.28248200	-7.24198400	3.59918800
C	6.29600200	-9.62343500	-3.70879400	C	-7.29356600	-8.80318100	3.76546600
H	7.86463300	-9.43470300	-2.25061700	H	-8.91064400	-8.49735500	2.38195600
H	4.57558200	-9.57849100	-4.99756700	H	-5.51240600	-8.89602000	4.96633100
H	6.80322100	-10.39562500	-4.27586200	H	-7.83413600	-9.52670100	4.36497000
C	-5.87651300	-7.47918800	2.26956100	C	4.82399200	-8.10813000	-2.36846600
C	-7.22416900	-7.72290600	1.96153400	C	6.05925900	-8.67598100	-2.01629800
C	-5.30097500	-8.25710100	3.28636300	C	4.17175700	-8.66355000	-3.48143800
C	-7.95446300	-8.70549100	2.61727500	C	6.60959000	-9.73644300	-2.72593200
H	-7.70474000	-7.13596800	1.18533700	H	6.59544100	-8.27906500	-1.15987400
C	-6.03379800	-9.21891600	3.96987100	C	4.72313200	-9.70941800	-4.21080900
H	-4.26173600	-8.09515600	3.55382600	H	3.21107700	-8.25873000	-3.78504900
C	-7.36152200	-9.45149500	3.62967100	C	5.94519500	-10.25435700	-3.83195900
H	-8.98938700	-8.88410600	2.34553500	H	7.56088300	-10.15797700	-2.41718900
H	-5.56748500	-9.79287100	4.76356400	H	4.19720900	-10.10366800	-5.07455400
H	-7.93289500	-10.21085300	4.15284600	H	6.37547500	-11.07714800	-4.39331900
C	-9.28808200	3.46863300	-1.32698400	C	9.66931200	2.33354100	1.40944900
C	-9.59823500	4.82868300	-1.16298700	C	10.07435800	3.66112700	1.17880100
C	-10.32999800	2.61613100	-1.72814400	C	10.66486600	1.44665500	1.85986200
C	-10.88037500	5.31308500	-1.38431300	C	11.38412600	4.07682900	1.37607200
H	-8.82251200	5.51402900	-0.83863700	H	9.34315500	4.37969000	0.82234400
C	-11.60808400	3.09694700	-1.97715400	C	11.97468600	1.85527500	2.07138900
H	-10.12704900	1.55793400	-1.85578900	H	10.39512900	0.41434700	2.05812000
C	-11.88624600	4.44792300	-1.80046400	C	12.34225400	3.17458200	1.82731700
H	-11.09640500	6.36555800	-1.23512100	H	11.66121700	5.10703500	1.17559400
H	-12.39027300	2.41894000	-2.30125100	H	12.71221900	1.14365100	2.42869500
H	-12.88683200	4.82536700	-1.98280500	H	13.36577600	3.49684700	1.98780800
N	0.43928500	8.03908100	0.06056500	N	0.60473400	7.93957300	-0.19554100
N	7.92027000	1.99652700	1.08188800	N	-7.63164000	3.06371900	-0.87988200
N	4.33663800	-6.61746800	-1.50639400	N	-5.19474700	-5.99819100	1.43172500

Structure S62. The coordinates of both the optimized structures of B1N4L$_3$ at S$_0$ and S$_1$ in toluene.

S_0-optimized structure				S_1-optimized structure			
H	-3.22781000	3.13942500	0.54382800	H	-3.66457900	2.70744700	0.56716100
C	-7.06263800	3.45747800	-2.36182600	C	-7.70170400	2.42600200	-2.06729700
C	-7.04169800	2.53856500	-1.30197700	C	-7.46604900	1.53143100	-1.01351700
C	-6.03648000	2.63396800	-0.33091700	C	-6.42037200	1.78176700	-0.11463800
C	-5.09845500	3.64428100	-0.42277500	C	-5.65024000	2.91635600	-0.27255300
C	-5.14273800	4.58236200	-1.46428800	C	-5.90595500	3.82653000	-1.31147100
C	-6.13008700	4.47967600	-2.44008900	C	-6.93649500	3.57159900	-2.21371300
H	-7.82724700	3.36555000	-3.12394800	H	-8.50070300	2.21563600	-2.76837300
H	-6.01205100	1.92657100	0.48990800	H	-6.23954800	1.09465700	0.70424500
H	-6.17539900	5.18332700	-3.26449400	H	-7.14350400	4.25392300	-3.03108800
C	-3.95652000	3.95713000	0.50709200	C	-4.50702000	3.40816700	0.57459400
H	-4.29512500	4.11858900	1.53623100	H	-4.79698000	3.53518500	1.62320400
C	-3.36745200	5.20396700	-0.09693400	C	-4.14955600	4.71784000	-0.07643400
C	-2.32392900	5.98343600	0.36421800	C	-3.19610700	5.64094600	0.30187500
C	-1.95956500	7.12939500	-0.35217800	C	-3.04743300	6.81867600	-0.44608900
C	-2.64849000	7.46359900	-1.52390500	C	-3.86406200	7.02991600	-1.56870900
C	-3.70560800	6.68828500	-1.97636600	C	-4.82776700	6.10687400	-1.93642200
C	-4.07150800	5.55267400	-1.26135000	C	-4.97821100	4.94154500	-1.18951300
H	-1.79436800	5.73030900	1.27626100	H	-2.57182500	5.47644400	1.17275200
H	-2.35061900	8.34804800	-2.07457900	H	-3.73134700	7.93201500	-2.15330300
H	-4.23142800	6.97153000	-2.88179100	H	-5.44714500	6.29690700	-2.80641100
H	3.01776900	4.72537600	-1.35053300	H	2.21092600	4.93255300	-1.27440600
C	1.02151000	7.98925300	1.58625200	C	-0.20741400	8.12767600	1.42113200
C	0.30845800	7.40736900	0.52613000	C	-0.82173400	7.39489100	0.39571200
C	0.83403000	6.28100700	-0.12067600	C	-0.14853200	6.30062200	-0.17032200
C	2.05645300	5.77890100	0.28312500	C	1.11561500	5.98087500	0.27713800
C	2.78259700	6.38509500	1.32023500	C	1.75279800	6.74684900	1.27477400
C	2.25356000	7.49230900	1.97664300	C	1.07163100	7.81919900	1.85320500
H	0.59993700	8.84668800	2.09645400	H	-0.74202600	8.95833100	1.86676800
H	0.28977100	5.82917300	-0.94209700	H	-0.61760700	5.73478300	-0.96771200
H	2.78969500	7.96755500	2.79107000	H	1.53237500	8.41422700	2.63428600
C	2.83058400	4.61616100	-0.27684300	C	2.03833200	4.88772900	-0.19363200
H	2.28990500	3.67127700	-0.14986600	H	1.62205500	3.89469500	0.01345300
C	4.10481500	4.64833200	0.52451000	C	3.29719800	5.14987900	0.59372800
C	5.22939600	3.85329300	0.41966300	C	4.52206100	4.52520700	0.51374000
C	6.35921000	4.09854000	1.22441300	C	5.63223500	4.98299300	1.26655200
C	6.27612900	5.14008200	2.16862400	C	5.39041000	6.07122200	2.14328700
C	5.13851300	5.92064700	2.31187500	C	4.15774400	6.68766500	2.25840400
C	4.05494800	5.68789400	1.46790100	C	3.09874900	6.24306300	1.46122800
H	5.26355600	3.05369200	-0.31365600	H	4.66354700	3.70154700	-0.17966900
H	7.13043500	5.33761100	2.80761200	H	6.21262400	6.43620600	2.75031200
H	5.10681700	6.71256000	3.05284900	H	4.02462700	7.51831900	2.94491400
B	7.66907800	3.28624800	0.97758700	B	7.05122700	4.38910100	1.02570400
H	-4.36139100	-2.35191800	-1.32774400	H	-3.99789500	-2.76257700	-1.28028100
C	-8.18230000	-0.08798300	0.60841900	C	-8.13918500	-1.37174400	0.82608000
C	-7.64124200	0.28594000	-0.62460000	C	-7.68337000	-0.79811200	-0.36709400
C	-6.71230800	-0.53843800	-1.26487000	C	-6.62493800	-1.38505300	-1.06987400
C	-6.32171800	-1.71400200	-0.65026100	C	-6.03168800	-2.52430900	-0.56106700
C	-6.84035100	-2.07105100	0.60494200	C	-6.47004400	-3.07868500	0.65304200
C	-7.78599400	-1.26363700	1.22893400	C	-7.53673700	-2.50728200	1.34149000
H	-8.90655500	0.56486100	1.08212800	H	-8.96153600	-0.90199500	1.35287100
H	-6.31157400	-0.23957600	-2.22755800	H	-6.29181200	-0.93844000	-2.00035100
H	-8.19998200	-1.53296800	2.19454100	H	-7.88715500	-2.92862900	2.27733700
C	-5.35527400	-2.76627700	-1.12690900	C	-4.90852300	-3.35211500	-1.12888500
H	-5.69224500	-3.23299100	-2.05944000	H	-5.17111100	-3.77649200	-2.10435300
C	-5.33309900	-3.74261500	0.02007100	C	-4.71705100	-4.41726200	-0.08090200
C	-4.58816700	-4.89808700	0.16321300	C	-3.80459300	-5.45389300	-0.04806100
C	-4.66466800	-5.62033000	1.36279200	C	-3.76690200	-6.29457100	1.07457900
C	-5.53697500	-5.18182000	2.37083500	C	-4.68466400	-6.09723500	2.11781300
C	-6.31775300	-4.05035700	2.20337900	C	-5.62553700	-5.08530700	2.06035000
C	-6.20917800	-3.31545900	1.02883500	C	-5.63994200	-4.23208500	0.96162700
H	-3.93595300	-5.23813100	-0.63255800	H	-3.12385000	-5.61953500	-0.87480100
H	-5.59272600	-5.73677600	3.29881800	H	-4.64841400	-6.74603700	2.98364800
H	-6.98207100	-3.73568500	3.00100400	H	-6.32421300	-4.95417000	2.87910200
H	6.02679500	-1.88288000	1.96723500	H	6.03100200	-0.93082900	2.04067200
C	6.72980000	-4.15412300	-2.25404100	C	7.44572100	-3.18119600	-2.01822400
C	5.82339900	-4.66165000	-1.30820400	C	6.53908500	-3.79023200	-1.13050100
C	5.49119400	-3.87512400	-0.19491600	C	5.98870600	-3.03175400	-0.07422500
C	6.02041600	-2.60344500	-0.08008200	C	6.30865500	-1.69988900	0.03208600
C	6.93867400	-2.11136900	-1.01907400	C	7.24155300	-1.09556000	-0.84471700
C	7.29949200	-2.90124800	-2.10472800	C	7.81242900	-1.85966600	-1.87167700
H	6.98260300	-4.75376200	-3.11906600	H	7.85458600	-3.76159600	-2.83586700
H	4.81296700	-4.25774700	0.55793500	H	5.32468100	-3.49862900	0.64330900
H	7.99753600	-2.53901400	-2.85178000	H	8.51536200	-1.41662600	-2.56787200
C	5.73901600	-1.55324400	0.96230800	C	5.79121000	-0.67048800	1.00327700
H	4.67309300	-1.30519200	1.01017700	H	4.70103100	-0.57669800	0.95034000
C	6.57823200	-0.39064100	0.49953400	C	6.50194000	0.58680400	0.56812200
C	6.67451900	0.88897000	1.01294300	C	6.37861800	1.87345100	1.03967400
C	7.57052300	1.81621000	0.44997800	C	7.19903100	2.91809200	0.53997700
C	8.34786500	1.40158600	-0.64586400	C	8.14721000	2.56075800	-0.45512200
C	8.20652500	0.14414900	-1.21669000	C	8.23491700	1.29165300	-0.98805900
C	7.31247400	-0.75352100	-0.64235800	C	7.39701200	0.28603100	-0.48405700
H	6.06367200	1.18399800	1.86131600	H	5.66074600	2.10003600	1.82276600
H	9.05827900	2.09854500	-1.07866300	H	8.79996400	3.33654200	-0.84205100
H	8.78846900	-0.13048300	-2.09025000	H	8.93960200	1.07839300	-1.78581200
H	0.74485400	-4.22550300	0.71026200	H	1.35864700	-4.11418100	0.53439800
C	-2.02672100	-8.19478500	0.93876900	C	-0.78398800	-8.46830200	0.56995300
C	-2.57041600	-6.91086400	1.10511600	C	-1.52093700	-7.27545400	0.74688600
C	-1.77233800	-5.79497700	0.80947400	C	-0.88605900	-6.04113600	0.49498100
C	-0.48205800	-5.98373600	0.35310600	C	0.42942700	-6.02856900	0.09213300
C	0.07239300	-7.26721100	0.24241900	C	1.17652100	-7.21987100	-0.01450100
C	-0.71355000	-8.37564000	0.53524400	C	0.54742300	-8.44591100	0.21668700
H	-2.64311200	-9.06115600	1.14201100	H	-1.27483300	-9.41981900	0.72349900
H	-2.16457900	-4.79392300	0.93854200	H	-1.42324900	-5.11228700	0.63414500

H	-0.32046400	-9.38183100	0.43621400	H	1.08754900	-9.37972200	0.10825300
C	0.52485200	-4.95024200	-0.08136900	C	1.29615800	-4.85183200	-0.27233500
H	0.16351300	-4.37346300	-0.94014000	H	0.90716200	-4.32441100	-1.15006200
C	1.73340900	-5.78368200	-0.42127100	C	2.63211400	-5.49357100	-0.54139000
C	2.96934400	-5.36863400	-0.87847000	C	3.81836700	-4.90131100	-0.89906200
C	3.99308700	-6.31229400	-1.05344900	C	4.98252500	-5.69765500	-1.01300200
C	3.72050500	-7.66378100	-0.78914900	C	4.88026700	-7.09313700	-0.79423200
C	2.46127700	-8.08135600	-0.38891400	C	3.67575800	-7.68534000	-0.49313500
C	1.45604400	-7.14053700	-0.19874100	C	2.53247100	-6.89009400	-0.35907300
H	3.14949500	-4.32263700	-1.09332000	H	3.86594600	-3.83821800	-1.09401500
H	4.50964600	-8.39549900	-0.90621800	H	5.77219900	-7.70243200	-0.84847100
H	2.28242000	-9.13553900	-0.20608200	H	3.63137200	-8.75630300	-0.33115800
C	-1.10421100	9.35929800	0.11658700	C	-2.38291200	9.15157700	-0.24502300
C	-0.09060900	10.21864100	-0.31613800	C	-1.43322500	10.01640600	-0.79536300
C	-2.31811900	9.90337400	0.54412500	C	-3.62368700	9.66231600	0.14606000
C	-0.28754900	11.59197200	-0.30718900	C	-1.72175900	11.36493400	-0.94447200
H	0.85004600	9.80466200	-0.65953200	H	-0.47135600	9.62462400	-1.10386400
C	-2.51173700	11.27705900	0.53277700	C	-3.90811600	11.00961000	-0.02100600
H	-3.10707200	9.24311000	0.88438900	H	-4.35916300	8.99746100	0.58347100
C	-1.49832400	12.13082800	0.11269900	C	-2.95986800	11.86992200	-0.56308100
H	0.50983500	12.24406400	-0.64661200	H	-0.97463400	12.02290300	-1.37445100
H	-3.46023700	11.68180600	0.86850000	H	-4.87463900	11.39098100	0.28971800
H	-1.65085000	13.20395600	0.11147100	H	-3.18274000	12.92363700	-0.68543400
C	-9.37061200	1.76359200	-1.48513700	C	-9.67542100	0.46224900	-0.97199300
C	-10.18402200	0.71570200	-1.93085400	C	-10.39320800	-0.65464900	-1.41299200
C	-9.94491200	3.02552100	-1.29663300	C	-10.37475000	1.62878300	-0.64576000
C	-11.53161300	0.92810500	-2.17949900	C	-11.77491300	-0.60317800	-1.51855400
H	-9.75162800	-0.26624300	-2.08182800	H	-9.86021000	-1.56088900	-1.67521100
C	-11.29094200	3.22956000	-1.56387000	C	-11.75558600	1.67357600	-0.77127500
H	-9.33564700	3.84503200	-0.93584100	H	-9.83376300	2.49719800	-0.29023900
C	-12.09636500	2.18606800	-2.00433900	C	-12.46706200	0.56070700	-1.20421400
H	-12.14193800	0.10061600	-2.52481500	H	-12.31240100	-1.47988600	-1.86317600
H	-11.71500400	4.21600200	-1.40978700	H	-12.27982300	2.58703500	-0.51224700
H	-13.14848400	2.35035300	-2.20624000	H	-13.54635200	0.59982200	-1.29500100
C	-4.43193300	-7.79628700	2.40132200	C	-3.28029800	-8.52901800	1.85359100
C	-5.64152000	-8.39780000	2.05612400	C	-4.35072900	-9.25740700	1.34060000
C	-3.77272500	-8.19524600	3.56410700	C	-2.65973600	-8.93663300	3.03311400
C	-6.18660600	-9.38248700	2.86879900	C	-4.79619900	-10.39111600	2.00645200
H	-6.14893900	-8.08592800	1.15044300	H	-4.82635400	-8.92959600	0.42354200
C	-4.31556000	-9.19104800	4.36436100	C	-3.10565200	-10.07580700	3.68894300
H	-2.83469100	-7.72285500	3.83205500	H	-1.83355200	-8.35729400	3.42901800
C	-5.52501400	-9.78605300	4.02291200	C	-4.17447800	-10.80468500	3.17914000
H	-7.12775000	-9.84396100	2.59114300	H	-5.62798500	-10.95671500	1.60232400
H	-3.79506600	-9.49419300	5.26605800	H	-2.62183000	-10.38814400	4.60739700
H	-5.94932000	-10.55957800	4.65280600	H	-4.52297000	-11.69160500	3.69568200
C	6.04254000	-6.89814800	-2.21994700	C	7.23401100	-5.98814600	-1.84995500
C	7.30406900	-7.27261200	-1.75958700	C	8.45876100	-6.09320600	-1.19357300
C	5.54144300	-7.46309800	-3.39217500	C	7.01114600	-6.68391300	-3.03711200
C	8.05742600	-8.19756000	-2.46972600	C	9.45401900	-6.90144200	-1.72303800
H	7.68761800	-6.83216700	-0.84645800	H	8.61966600	-5.54399300	-0.27345600
C	6.29360400	-8.39819900	-4.08992800	C	8.01247800	-7.49121000	-3.55833500
H	4.56138600	-7.16650100	-3.74770100	H	6.05982700	-6.58095000	-3.54604800
C	7.55459400	-8.76640600	-3.63433100	C	9.23429500	-7.60248000	-2.90422700
H	9.03752800	-8.48258200	-2.10372000	H	10.40344800	-6.98713700	-1.20731200
H	5.89560500	-8.83219600	-5.00044900	H	7.83976300	-8.02596100	-4.48527300
H	8.14209500	-9.49299800	-4.18398500	H	10.01534900	-8.23120800	-3.31582900
C	9.07700800	3.94111400	1.17561600	C	8.33186300	5.28564600	1.17815800
C	9.28888100	5.32000100	1.01298600	C	8.31260300	6.67945700	0.97172700
C	10.19162400	3.15566900	1.51300500	C	9.58068800	4.73133900	1.52371900
C	10.54636100	5.88582400	1.17483800	C	9.45085900	7.46278300	1.10176400
H	8.45410100	5.95553400	0.73744000	H	7.37983100	7.15421900	0.68492600
C	11.44694900	3.71714700	1.70204200	C	10.72327100	5.50692100	1.66225100
H	10.06438800	2.08542800	1.63803700	H	9.64702000	3.66195300	1.69846700
C	11.62690300	5.08503200	1.52801200	C	10.66604600	6.88143500	1.45192700
H	10.68527800	6.95159500	1.02813800	H	9.39312300	8.53221300	0.92363400
H	12.28750600	3.08918700	1.97731400	H	11.66325500	5.03951200	1.93926200
H	12.60880800	5.52589000	1.66387000	H	11.55697100	7.49149400	1.55791000
N	-0.91536300	7.96028100	0.11313700	N	-2.09271300	7.77966900	-0.07949400
N	-8.01164600	1.52260400	-1.21778300	N	-8.27544800	0.39037400	-0.85294800
N	-3.88881000	-6.77536900	1.57592000	N	-2.84019000	-7.35239300	1.17856600
N	5.27618400	-5.94252000	-1.49847800	N	6.21629100	-5.14681200	-1.31416500

Structure S63. The coordinates of both the optimized structures of $N5L_3$ at S_0 and S_1 in toluene.

S_0-optimized structure				S_1-optimized structure			
H	-2.80664700	3.41810200	0.46959600	H	-2.79753300	3.38647500	0.45208800
C	-6.65713300	4.16390300	-2.33514100	C	-6.66497100	4.15642100	-2.32707500
C	-6.71325600	3.24857600	-1.27369000	C	-6.71927600	3.23914300	-1.26602300
C	-5.67874200	3.22942700	-0.32915600	C	-5.67589200	3.20904800	-0.32932000
C	-4.63531900	4.12736300	-0.44767100	C	-4.63038000	4.10168700	-0.45166600
C	-4.60051600	5.06588000	-1.48950200	C	-4.59763200	5.04493000	-1.49167900
C	-5.61770500	5.07482500	-2.43955500	C	-5.62132200	5.06077400	-2.43665100
H	-7.44679400	4.15978800	-3.07697700	H	-7.46069500	4.15901500	-3.06248200
H	-5.71363000	2.52430100	0.49331000	H	-5.71205400	2.50203400	0.49145700
H	-5.60564400	5.77980200	-3.26398900	H	-5.61085800	5.76834500	-3.25884700
C	-3.44275200	4.31038400	0.45285300	C	-3.43317700	4.27921000	0.44374100
H	-3.73517900	4.50338600	1.49065700	H	-3.72006300	4.46647000	1.48408700
C	-2.73672900	5.48798700	-0.16457700	C	-2.72979000	5.45966200	-0.17105400
C	-1.60665000	6.15041900	0.27480100	C	-1.60011100	6.12090600	0.26831900
C	-1.13816700	7.25587900	-0.44538600	C	-1.13595300	7.23270900	-0.44675700
C	-1.81199800	7.66300500	-1.60381800	C	-1.81413200	7.64482600	-1.60176800
C	-2.95433400	7.00516800	-2.03432900	C	-2.95512500	6.98680800	-2.03348800
C	-3.42511600	5.91301800	-1.31249500	C	-3.42305400	5.88907200	-1.31591000
H	-1.08819400	5.83868200	1.17486500	H	-1.07895200	5.80547300	1.16549300
H	-1.43376800	8.51197500	-2.16090300	H	-1.43897300	8.49749300	-2.15529400
H	-3.46470400	7.34552400	-2.92892200	H	-3.46803300	7.33080100	-2.92520900
H	3.73435400	4.50484700	-1.49091800	H	3.72008600	4.46631300	-1.48401400
C	1.80996200	7.66312100	1.60417700	C	1.81433500	7.64481900	1.60179800
C	1.13635800	7.25609800	0.44557600	C	1.13611300	7.23268700	0.44681800
C	1.60522900	6.15095700	-0.27484800	C	1.60021900	6.12084800	-0.26823700
C	2.73545500	5.48873600	0.16446900	C	2.72989000	5.45958400	0.17112500
C	3.42360600	5.91366900	1.31256500	C	3.42320000	5.88901000	1.31594800
C	2.95244200	7.00549900	2.03463300	C	2.95532100	6.98678100	2.03350600
H	1.43143300	8.51183800	2.16144500	H	1.43921700	8.49751400	2.15530800
H	1.08694800	5.83930100	-1.17504200	H	1.07902600	5.80540400	-1.16538700
H	3.46262500	7.34576900	2.92936400	H	3.46826600	7.33078600	2.92520200
C	3.44187600	4.31149500	-0.45319300	C	3.43322600	4.27909300	-0.44365400
H	2.80602700	3.41903600	-0.47023200	H	2.79755700	3.38637500	-0.45195400
C	4.63440300	4.12857900	0.44740500	C	4.63045100	4.10156600	0.45172200
C	5.67809100	3.23096600	0.32876800	C	5.67593600	3.20889600	0.32937200
C	6.71250200	3.25016300	1.27341400	C	6.71935000	3.23899300	1.26604000
C	6.65600400	4.16519400	2.33510100	C	6.66510300	4.15630600	2.32706500
C	5.61631000	5.07579800	2.43964000	C	5.62148000	5.06068800	2.43664700
C	4.59922600	5.06681700	1.48947500	C	4.59776000	5.04484200	1.49170800
H	5.71326000	2.52606000	-0.49387400	H	5.71205400	2.50185600	-0.49138600
H	7.44557800	4.16110000	3.07702900	H	7.46085200	4.15890300	3.06244600
H	5.60396100	5.78055200	3.26426000	H	5.61106100	5.76828500	3.25882100
H	-4.57228900	-1.88649300	-1.27279500	H	-4.51825000	-1.83704700	-1.22132300
C	-8.13777200	0.76238700	0.65933400	C	-8.20959100	0.71736600	0.62501400
C	-7.55612800	1.08145000	-0.57087000	C	-7.57696800	1.07719100	-0.57238500
C	-6.71807200	0.16445000	-1.21103600	C	-6.68864100	0.18415000	-1.19231000
C	-6.45700700	-1.04790500	-0.59953500	C	-6.43704300	-1.03321500	-0.59735300
C	-7.01616100	-1.35352900	0.65197200	C	-7.04185400	-1.37871000	0.63033200
C	-7.87245200	-0.45172100	1.27562800	C	-7.94807500	-0.49870900	1.23107000
H	-8.78919900	1.48697500	1.13436900	H	-8.89538200	1.41996300	1.08491900
H	-6.28405500	0.42155400	-2.17135300	H	-6.21730800	0.46715200	-2.12755400
H	-8.31695000	-0.67847400	2.23859800	H	-8.43002500	-0.75039300	2.16947700
C	-5.60519900	-2.19427200	-1.07737000	C	-5.55256300	-2.16076400	-1.06326900
H	-5.98569700	-2.61919100	-2.01314400	H	-5.89748900	-2.57738700	-2.01646800
C	-5.69110400	-3.17205200	0.06520700	C	-5.66182000	-3.15849200	0.06147200
C	-5.07127100	-4.39912300	0.20607400	C	-5.03016100	-4.37216500	0.20553000
C	-5.22871900	-5.11533800	1.40132500	C	-5.23125200	-5.11623500	1.39012200
C	-6.05579300	-4.59361700	2.40755000	C	-6.10931500	-4.61838600	2.37432900
C	-6.71330500	-3.38571200	2.24243000	C	-6.77351000	-3.42206200	2.20379200
C	-6.52255900	-2.66001200	1.07238100	C	-6.54593500	-2.66814600	1.04576400
H	-4.45188400	-4.80048000	-0.58739400	H	-4.38164900	-4.75812900	-0.57170500
H	-6.17365800	-5.14414300	3.33245000	H	-6.24977500	-5.18197500	3.28830300
H	-7.34490700	-3.00695000	3.03878300	H	-7.44088900	-3.06349300	2.97968200
H	5.98655300	-2.61773300	2.01311100	H	5.89745000	-2.57753000	2.01646300
C	6.05687200	-4.59234000	-2.40749700	C	6.10918300	-4.61850600	-2.37434800
C	5.22999500	-5.11422700	-1.40119700	C	5.23112500	-5.11634700	-1.39013100
C	5.07243500	-4.39799700	-0.20596900	C	5.03006200	-4.37228000	-0.20553300
C	5.69196300	-3.17076200	-0.06519700	C	5.66174400	-3.15861900	-0.06147700
C	6.52322000	-2.65855200	-1.07244700	C	6.54585600	-2.66828200	-1.04577700
C	6.71408100	-3.38425600	-2.24247500	C	6.77340300	-3.42219500	-2.20381200
H	6.17482000	-5.14287700	-3.33238000	H	6.24962100	-5.18209300	-3.28832800
H	4.45319900	-4.79947700	0.58755400	H	4.38155500	-4.75823700	0.57170800
H	7.34553300	-3.00536700	-3.03888500	H	7.44077800	-3.06363400	-2.97970900
C	5.60588100	-2.19295400	1.07734400	C	5.55251900	-2.16089500	1.06327100
H	4.57290500	-1.88543400	1.27282500	H	4.51821400	-1.83716000	1.22134100
C	6.45735900	-1.04638600	0.59940100	C	6.43701400	-1.03336000	0.59735100
C	6.71815100	0.16606300	1.21083200	C	6.68864300	0.18399700	1.19231200
C	7.55591700	1.08325600	0.57056400	C	7.57698000	1.07702400	0.57238300
C	8.13755400	0.76429400	-0.65967000	C	8.20958400	0.71719400	-0.62502500
C	7.87251200	-0.44991200	-1.27589000	C	7.94803700	-0.49887200	-1.23108600
C	7.01650600	-1.35192000	-0.65213100	C	7.04180400	-1.37885800	-0.63034300
H	6.28413500	0.42309500	2.17116800	H	6.21732700	0.46700200	2.12756400
H	8.78875200	1.48903500	-1.13478600	H	8.89538500	1.41978000	-1.08493200
H	8.31700100	-0.67659000	-2.23888200	H	8.42997100	-0.75056000	-2.16949900
H	0.30878000	-4.27535500	0.82238000	H	0.30550000	-4.21790200	0.82309900
C	-2.87039600	-7.92985000	0.91737500	C	-2.85221900	-7.90508500	0.92025500
C	-3.27587500	-6.60358900	1.13432100	C	-3.26786500	-6.57218400	1.14702400
C	-2.36575300	-5.56677700	0.87892300	C	-2.35741400	-5.51579900	0.87938000
C	-1.10513600	-5.87088900	0.40354800	C	-1.10821900	-5.81284300	0.40140900
C	-0.69021400	-7.19999300	0.23263800	C	-0.67667500	-7.15303900	0.22418500
C	-1.58579500	-8.23095000	0.49304400	C	-1.58027400	-8.20103100	0.48798300
H	-3.57398000	-8.73372600	1.09311200	H	-3.55664800	-8.70975400	1.09002500
H	-2.65203000	-4.53625700	1.04927600	H	-2.65016500	-4.48937900	1.06078300
H	-1.30149200	-9.26802100	0.35007600	H	-1.29246200	-9.23488600	0.33346200

C	0.00067900	-4.93083400	0.00021400	C	-0.00006500	-4.87297400	-0.00001800
H	-0.30753200	-4.27531900	-0.82188400	H	-0.30562000	-4.21791200	-0.82314700
C	1.10665000	-5.87066000	-0.40322200	C	1.10807500	-5.81286700	-0.40142900
C	2.36721200	-5.56628600	-0.87857600	C	2.35727500	-5.51585100	-0.87940400
C	3.27750600	-6.60291900	-1.13409200	C	3.26771000	-6.57225500	-1.14703000
C	2.87225300	-7.92927000	-0.91728100	C	2.85204200	-7.90514500	-0.92023900
C	1.58770700	-8.23063100	-0.49297100	C	1.58009200	-8.20106400	-0.48796300
C	0.69195400	-7.19985200	-0.23245000	C	0.67651000	-7.15305300	-0.22418300
H	2.65331500	-4.53570000	-1.04882700	H	2.65004200	-4.48943800	-1.06082400
H	3.57597000	-8.73301000	-1.09311600	H	3.55645900	-8.70982900	-1.08999400
H	1.30357700	-9.26776600	-0.35011300	H	1.29226400	-9.23491200	-0.33342400
C	-0.00118100	9.37070500	0.00027500	C	0.00010700	9.34688100	0.00001700
C	1.15440600	10.08228600	-0.33735700	C	1.15431700	10.05770400	-0.34366100
C	-1.15696700	10.08190000	0.33804200	C	-1.15408000	10.05774800	0.34367800
C	1.15119900	11.46939000	-0.32694100	C	1.15127900	11.44475000	-0.33365500
H	2.05356900	9.54198400	-0.60845800	H	2.05160100	9.51669400	-0.61952100
C	-1.15414600	11.46900600	0.32789400	C	-1.15099900	11.44479300	0.33364000
H	-2.05598000	9.54129400	0.60903600	H	-2.05138100	9.51677300	0.61955200
C	-0.00157200	12.17387800	0.00054500	C	0.00015100	12.14916100	-0.00001600
H	2.05771200	12.00255800	-0.59230600	H	2.05626000	11.97796500	-0.60400700
H	-2.06080800	12.00187100	0.59336100	H	-2.05596300	11.97804300	0.60398000
H	-0.00172200	13.25779700	0.00064900	H	0.00016800	13.23308200	-0.00002800
C	-9.11893600	2.74962800	-1.40067500	C	-9.12719600	2.77133700	-1.36979300
C	-10.05404400	1.80823900	-1.84480200	C	-10.07261200	1.84991400	-1.83374300
C	-9.54085100	4.06633900	-1.18685100	C	-9.53251800	4.08819500	-1.12574100
C	-11.37198800	2.17751400	-2.06781100	C	-11.38603900	2.23935100	-2.04726300
H	-9.73933700	0.78552100	-2.01525900	H	-9.76751700	0.82853700	-2.02768400
C	-10.85846200	4.42756500	-1.42841000	C	-10.84602400	4.46962900	-1.35697400
H	-8.83509200	4.80491700	-0.82698700	H	-8.81693900	4.80896500	-0.74945400
C	-11.78522200	3.48950600	-1.86759700	C	-11.78335100	3.55155000	-1.81623200
H	-12.07890600	1.43089200	-2.41317700	H	-12.10202600	1.50967500	-2.40960100
H	-11.16389500	5.45385000	-1.25500400	H	-11.14038300	5.49480800	-1.15980700
H	-12.81449000	3.77626700	-2.04955800	H	-12.80955000	3.85401400	-1.99005400
C	-5.21946300	-7.31737800	2.41725500	C	-5.20171900	-7.31287800	2.40201600
C	-6.49414200	-7.76914300	2.07634100	C	-6.47291700	-7.76057800	2.04443600
C	-4.59497900	-7.82088500	3.55863100	C	-4.57153400	-7.83463100	3.53128800
C	-7.13655500	-8.70792500	2.87184600	C	-7.10731000	-8.72417300	2.81556300
H	-6.97515500	-7.37749200	1.18742700	H	-6.95212900	-7.35179800	1.16266900
C	-5.23664500	-8.77098500	4.34103500	C	-5.21124400	-8.80134300	4.29371100
H	-3.60616300	-7.46534400	3.82432700	H	-3.58684700	-7.47438200	3.80491400
C	-6.51012700	-9.21595000	4.00415400	C	-6.47974400	-9.24876900	3.94000400
H	-8.12752500	-9.05198700	2.59713700	H	-8.09329600	-9.07277100	2.52974500
H	-4.74199800	-9.15583400	5.22597100	H	-4.71938600	-9.19876600	5.17432900
H	-7.01099200	-9.95396100	4.62028800	H	-6.97724700	-10.00315100	4.53869500
C	5.22121200	-7.31629700	-2.41706600	C	5.20155800	-7.31300400	-2.40199600
C	6.49605200	-7.76770000	-2.07627600	C	6.47272800	-7.76074800	-2.04437400
C	4.59673400	-7.82002700	-3.55834600	C	4.57139000	-7.83474200	-3.53128400
C	7.13863000	-8.70634300	-2.87181200	C	7.10711000	-8.72437200	-2.81547400
H	6.97705900	-7.37587800	-1.18743400	H	6.95192700	-7.35198100	-1.16259400
C	5.23856900	-8.76998600	-4.34078100	C	5.21108800	-8.80148300	-4.29368100
H	3.60779000	-7.46476800	-3.82394300	H	3.58672300	-7.47446000	-3.80494400
C	6.51221100	-9.21458900	-4.00402500	C	6.47956000	-9.24895200	-3.93993200
H	8.12972500	-9.05012300	-2.59720000	H	8.09307500	-9.07300400	-2.52962300
H	4.74392600	-9.15501100	-5.22564200	H	4.71924300	-9.19889400	-5.17431100
H	7.01320700	-9.95249100	-4.62018400	H	6.97705500	-10.00335600	-4.53860300
C	9.11834300	2.75186700	1.40022200	C	9.12726100	2.77114100	1.36974400
C	9.54004300	4.06856400	1.18588400	C	9.53256900	4.08801500	1.12575700
C	10.05359300	1.81082500	1.84478500	C	10.07270600	1.84968300	1.83356300
C	10.85757500	4.43012100	1.42737700	C	10.84609100	4.46943000	1.35692700
H	8.83418100	4.80687100	0.82566700	H	8.81696500	4.80881300	0.74956900
C	11.37145800	2.18042400	2.06772900	C	11.38614900	2.23910200	2.04702100
H	9.73905900	0.78811900	2.01563700	H	9.76762200	0.82829200	2.02745200
C	11.78447300	3.49240600	1.86700700	C	11.78344800	3.55131700	1.81605600
H	11.16283800	5.45638800	1.25356700	H	11.14043800	5.49462200	1.15981200
H	12.07848500	1.43406700	2.41344100	H	12.10216000	1.50939900	2.40925800
H	12.81367900	3.77942300	2.04891500	H	12.80965900	3.85376700	1.98982900
N	4.57545800	-6.34296500	-1.61020700	N	4.55391300	-6.32068500	-1.61715400
N	7.79125700	2.35333500	1.16022500	N	7.80441000	2.35373100	1.13908300
N	-0.00098800	7.96245600	0.00013500	N	0.00008600	7.93842000	0.00003400
N	-7.79176200	2.35144000	-1.16060400	N	-7.80436000	2.35390800	-1.13907700
N	-4.57388500	-6.34390400	1.61042800	N	-4.55406400	-6.32058600	1.61714700

Structure S64. The coordinates of both the optimized structures of B6L$_3$ at S$_0$ and S$_1$ in toluene.

S_0-optimized structure				S_1-optimized structure			
B	8.78747800	4.81754000	-0.05193700	B	-9.72876500	-2.41405000	-0.04557800
B	8.56627100	-5.20022900	-0.05579900	B	-6.95413400	7.20958100	-0.02934900
B	0.21987100	10.01696700	-0.03954000	B	-2.76781500	-9.62129600	-0.05684300
B	-0.22118600	-10.01797200	-0.03643300	B	2.76970000	9.62152700	-0.05792800
B	-8.78666200	-4.81829100	-0.05114800	B	9.72688700	2.41671800	-0.05228900
C	10.23550700	5.31703500	0.27586400	C	-11.26194200	-2.52789400	0.26394500
C	10.45579400	6.42095100	1.11566800	C	-11.76568000	-3.52117000	1.12038400
C	11.36796700	4.67964300	-0.25688300	C	-12.19325800	-1.64221200	-0.30378200
C	11.73789500	6.85723100	1.42150700	C	-13.12090400	-3.61850000	1.40611400
H	9.60409600	6.93687200	1.54650900	H	-11.07697000	-4.22194300	1.58095900
C	12.65241600	5.12803800	0.02034500	C	-13.55285300	-1.74865900	-0.04336200
H	11.23537600	3.82533900	-0.91250300	H	-11.84293400	-0.86442100	-0.97454600
C	12.83951300	6.21465700	0.86750100	C	-14.01961700	-2.73481700	0.81881500
H	11.87955600	7.70221000	2.08674400	H	-13.47837200	-4.38654700	2.08374300
H	13.50883700	4.62759500	-0.41866600	H	-14.24986900	-1.06037400	-0.50952600
H	13.84217400	6.56043400	1.09536900	H	-15.08029200	-2.81465700	1.03184400
C	9.99216500	-5.76144600	0.26936200	C	-8.19206300	8.11974900	0.28704200
C	11.14957900	-5.17124900	-0.26404000	C	-9.45275200	7.86673800	-0.27966900
C	10.16680100	-6.87545200	1.10653200	C	-8.09063900	9.22518400	1.14834600
C	12.41427100	-5.67460600	0.00947100	C	-10.54826100	8.67712800	-0.01317800
H	11.05201500	-4.31032900	-0.91713300	H	-9.57023000	7.02509900	-0.95451200
C	11.42978100	-7.36649000	1.40887800	C	-9.18699900	10.02596000	1.43952100
H	9.29463300	-7.35559100	1.53792200	H	-7.13440400	9.45120700	1.60894500
C	12.55659200	-6.77018000	0.85384500	C	-10.41907200	9.75667800	0.85374700
H	13.29036600	-5.21006300	-0.43018700	H	-11.50527300	8.46546600	-0.47817300
H	11.53665500	-8.21822700	2.07200100	H	-9.08086200	10.86343700	2.12087800
H	13.54409200	-7.15886500	1.07873500	H	-11.27528200	10.38604000	1.07174100
C	-0.00486200	11.53117100	0.29249600	C	-2.93479600	-11.15065300	0.24723700
C	-1.09628500	12.24181800	-0.23315500	C	-2.07860900	-12.10827300	-0.32158200
C	0.87659500	12.23520800	1.12910700	C	-3.94713000	-11.62425800	1.09848500
C	-1.29114600	13.58739600	0.04806100	C	-2.23153200	-13.46467300	-0.06800700
H	-1.79582400	11.73071600	-0.88635500	H	-1.28689700	-11.78097600	-0.98763100
C	0.67233400	13.57311200	1.43887500	C	-4.09038800	-12.97669400	1.37803500
H	1.73021900	11.71770100	1.55420300	H	-4.62604200	-10.91446000	1.55975000
C	-0.40975700	14.25390900	0.89207400	C	-3.23544400	-13.90176300	0.78917500
H	-2.13319400	14.11629600	-0.38533500	H	-1.56580500	-14.18238100	-0.53546300
H	1.35973900	14.08800100	2.10140200	H	-4.87215600	-13.31137200	2.05157400
H	-0.56570300	15.30232300	1.12288900	H	-3.35154500	-14.96013900	0.99697100
C	-9.72296200	6.20664900	0.27366100	C	7.81550300	-8.48411600	0.28804300
C	-9.73134900	7.50734600	-0.25607300	C	7.50924800	-9.73482100	-0.27393000
C	-10.79450300	5.84579300	1.10674300	C	8.92646800	-8.42413900	1.14577900
C	-10.76169600	8.39693500	0.01713300	C	8.27581300	-10.86120400	-0.00651800
H	-8.92059100	7.81952100	-0.90605400	H	6.66080600	-9.81996900	-0.94506300
C	-11.81323200	6.73942400	1.40882400	C	9.68248700	-9.55125200	1.43902200
H	-10.81990900	4.84935100	1.53528500	H	9.19261400	-7.47608000	1.60171200
C	-11.80189700	8.01574800	0.85748000	C	9.36172400	-10.77293100	0.85751900
H	-10.75199400	9.38972700	-0.41977100	H	8.02468100	-11.81027700	-0.46804200
H	-12.62014000	6.43980600	2.06891100	H	10.52554500	-9.47737500	2.11771300
H	-12.60262600	8.71245100	1.08206100	H	9.95638100	-11.65332700	1.07670300
C	-0.51433900	-11.52093200	0.29341500	C	3.43951600	11.00667500	0.24844000
C	0.32988700	-12.26331000	1.13517900	C	2.83287700	11.94379700	1.10181900
C	-1.63263700	-12.18257900	-0.23976100	C	4.67324600	11.36698600	-0.31945600
C	0.06500800	-13.59109500	1.44258000	C	3.42734600	13.16670600	1.38257900
H	1.20230500	-11.78367400	1.56657400	H	1.88168200	11.70074200	1.56409600
C	-1.88790500	-13.51861200	0.03868900	C	5.26283900	12.59749100	-0.06287300
H	-2.30491000	-11.64069300	-0.89674300	H	5.17104700	10.67189900	-0.98775300
C	-1.04214700	-14.22353900	0.88792300	C	4.64272000	13.49956600	0.79473800
H	0.72458800	-14.13591300	2.10945200	H	2.94143900	13.86360900	2.05720100
H	-2.74935400	-14.00990400	-0.40080800	H	6.20838800	12.85338500	-0.52911100
H	-1.24526100	-15.26430900	1.11675800	H	5.10508600	14.45822700	1.00436100
C	-9.98597000	-5.77296600	0.27169200	C	11.13420300	3.03579800	0.25790300
C	-11.03880500	-5.36924300	1.10904500	C	12.04441300	2.39565400	1.11564800
C	-10.05387800	-7.06892900	-0.26537100	C	11.54174800	4.25462500	-0.31001800
C	-12.09606200	-6.21799700	1.40825100	C	13.28663900	2.94568100	1.40213700
H	-11.01861800	-4.37526400	1.54348300	H	11.76461400	1.45403800	1.57670300
C	-11.12252900	-7.91298800	0.00505800	C	12.79204700	4.79879400	-0.04867900
H	-9.25948100	-7.41328300	-0.91926500	H	10.86833300	4.77672400	-0.98193900
C	-12.14301900	-7.49048400	0.84970500	C	13.66622300	4.14737400	0.81458900
H	-12.88762800	-5.88631800	2.07156900	H	13.96221600	2.43542200	2.08041800
H	-11.15826600	-8.90268400	-0.43746600	H	13.08506500	5.73345300	-0.51498300
H	-12.97373600	-8.15177600	1.07199900	H	14.64005300	4.57459400	1.02874100
B	-8.56680200	5.20107400	-0.05128700	B	6.95486100	-7.21206000	-0.02870300
H	3.69580000	6.08228800	1.53804300	H	-5.10248200	-4.91330400	1.52313200
C	7.84556600	7.14761600	-0.66654900	C	-9.42029100	-4.91975000	-0.61962000
C	7.61716300	5.84754400	-0.17498700	C	-8.86108900	-3.70689100	-0.15573800
C	6.30491400	5.50940200	0.20471100	C	-7.49799200	-3.71343900	0.20969200
C	5.28482000	6.43779600	0.10898900	C	-6.75082100	-4.86912900	0.11830900
C	5.54161400	7.72158300	-0.39843700	C	-7.33364000	-6.05823000	-0.36447600
C	6.82639100	8.07868100	-0.79675500	C	-8.67798600	-6.08015900	-0.74158400
H	8.85048000	7.42459400	-0.96631300	H	-10.46621500	-4.93371800	-0.90577100
H	6.10036700	4.51530400	0.58980900	H	-7.04275900	-2.80123500	0.58196300
H	7.03215100	9.06652100	-1.19506100	H	-9.13461400	-6.98858900	-1.11942900
C	3.83064500	6.30692900	0.47403200	C	-5.30584100	-5.10804400	0.46418000
H	3.33929000	5.49849100	-0.07878400	H	-4.63362100	-4.45798000	-0.10662300
C	3.27455700	7.65768800	0.11182900	C	-5.11757300	-6.55976800	0.11535500
C	1.98023100	8.13376500	0.21088700	C	-3.98814000	-7.34671100	0.20362000
C	1.67358000	9.45444700	-0.16632200	C	-4.02963800	-8.70829900	-0.16424600
C	2.72041200	10.25796400	-0.65870500	C	-5.26082600	-9.22382600	-0.62934700
C	4.01661100	9.78402100	-0.79221700	C	-6.39522300	-8.44165000	-0.74786200
C	4.29377200	8.47887300	-0.39654300	C	-6.32671100	-7.10015300	-0.36674500
H	1.19367300	7.49277100	0.59665300	H	-3.06016300	-6.92376000	0.57506900
H	2.50155400	11.27770400	-0.95644800	H	-5.31082400	-10.26766100	-0.91884400
H	4.79698300	10.42331600	-1.19111200	H	-7.31902400	-8.86583800	-1.12629600
H	10.73747300	-0.23744300	0.76324600	H	-10.38322100	2.98501100	0.66434600
C	7.40318100	-3.16264500	-1.15485900	C	-6.31970200	4.92981100	-1.09493000
C	8.36406600	-3.66173300	-0.25402500	C	-7.15359500	5.67433300	-0.22783400
C	9.14517100	-2.73091700	0.45561000	C	-8.18559800	4.98006300	0.44099500
C	8.95574100	-1.37327600	0.27651900	C	-8.35060100	3.62302800	0.26297300

C	8.00095200	-0.90628600	-0.64110800
C	7.22335600	-1.80391000	-1.36683400
H	6.79600700	-3.86573100	-1.71479800
H	9.89333100	-3.08693700	1.15683300
H	6.48811400	-1.45227000	-2.08286800
C	9.65515500	-0.21344300	0.93194300
H	9.50998300	-0.21063900	2.01814300
C	9.00792500	0.97681200	0.27735000
C	9.25716100	2.32454100	0.45772600
C	8.51858600	3.28971200	-0.25168400
C	7.53695400	2.83434200	-1.15332300
C	7.29658900	1.48515800	-1.36590900
C	8.03342900	0.55336400	-0.64067400
H	10.01965500	2.64644400	1.16001700
H	6.96202600	3.56408600	-1.71321600
H	6.54651300	1.16702100	-2.08219300
H	-4.57470500	8.33621400	2.02800500
C	-6.22192200	5.10887600	-1.14782200
C	-7.10850500	5.73132300	-0.24764600
C	-6.64318600	6.85318100	0.46302800
C	-5.35102500	7.31109900	0.28499700
C	-4.49599100	6.67986100	-0.63272900
C	-4.93328000	5.57601100	-1.35894500
H	-6.56532100	4.24564900	-1.70777100
H	-7.30455700	7.35251800	1.16411800
H	-4.28173700	5.08581900	-2.07455900
C	-4.64434800	8.46561000	0.94204900
H	-5.16467600	9.41527900	0.77513900
C	-3.28974100	8.44104600	0.28746800
C	-2.20875400	9.28336200	0.46899400
C	-1.01171300	9.07394600	-0.24057000
C	-0.96309800	7.99405400	-1.14360100
C	-2.04953200	7.15903000	-1.35743600
C	-3.21601100	7.38211300	-0.63171700
H	-2.27477900	10.10755400	1.17225200
H	-0.05028600	7.82095400	-1.70339200
H	-1.98584700	6.34765400	-2.07468200
H	-5.57503000	-9.18004300	0.76883000
C	-6.43728500	-4.82561600	-1.14214300
C	-7.35250600	-5.41130300	-0.24599900
C	-6.93835600	-6.55466700	0.46213100
C	-5.66709900	-7.06818100	0.28563800
C	-4.78300000	-6.47233700	-0.62816800
C	-5.16982300	-5.34810600	-1.35181100
H	-6.74111100	-3.94648800	-1.70013100
H	-7.62248400	-7.02668300	1.16024300
H	-4.49574600	-4.88465300	-2.06428500
C	-5.01371100	-8.25487900	0.94040100
H	-4.94115900	-8.13245100	2.02694600
C	-3.65783700	-8.28803200	0.28891600
C	-2.61596400	-9.17849500	0.46953900
C	-1.40934800	-9.02041200	-0.23715500
C	-1.31061600	-7.94059300	-1.13603200
C	-2.35819900	-7.05701500	-1.34890400
C	-3.53512500	-7.23035400	-0.62650100
H	-2.72032600	-10.00145700	1.16953600
H	-0.38991300	-7.80633600	-1.69350900
H	-2.25682600	-6.24689800	-2.06324300
H	3.42499700	-6.23889500	1.53961200
C	2.26726300	-10.37092000	-0.65071300
C	1.25643900	-9.52091400	-0.16111000
C	1.62066800	-8.21429800	0.21397400
C	2.93478900	-7.79613500	0.11568300
C	3.91719700	-8.66253500	-0.39006200
C	3.58331200	-9.95504300	-0.78347200
H	2.00359100	-11.38042600	-0.94716100
H	0.86271400	-7.53846800	0.59740300
H	4.33519700	-10.62879500	-1.18035500
C	3.54951500	-6.47048800	0.47582900
H	3.09378700	-5.64197800	-0.07773900
C	4.99644700	-6.66545800	0.11073000
C	6.05599900	-5.78221000	0.20370800
C	7.35210000	-6.17822200	-0.17564700
C	7.52339300	-7.48823100	-0.66408100
C	6.46469500	-8.37451300	-0.79100100
C	5.19693200	-7.96058900	-0.39332100
H	5.89522000	-4.77936700	0.58673800
H	8.51516300	-7.80913700	-0.96392800
H	6.62696500	-9.37147300	-1.18667500
H	-7.12383900	0.15777000	1.53921500
C	-10.11309900	3.22132100	-0.67135200
C	-8.87403000	3.67287000	-0.17655400
C	-7.92603300	2.70496200	0.20418100
C	-8.22033000	1.35753800	0.10720300
C	-9.45939100	0.93871600	-0.40358200
C	-10.40989700	1.87334500	-0.80343900
H	-10.85456000	3.95353700	-0.97216000
H	-6.96356800	3.02439900	0.59144300
H	-11.36745300	1.55833500	-1.20423900
C	-7.38143700	0.16316800	0.47412700
H	-6.43330100	0.14216300	-0.07465900
C	-8.27204000	-0.99330500	0.10765600
C	-8.03735400	-2.35237200	0.20527300
C	-9.02671100	-3.27784400	-0.17578500
C	-10.24435400	-2.77242800	-0.67170000
C	-10.48159200	-1.41271900	-0.80403600
C	-9.49135400	-0.52062000	-0.40352500
H	-7.08991700	-2.71357100	0.59288600
H	-11.01701400	-3.47141900	-0.97294400
H	-11.42407700	-1.05604700	-1.20562500
C	-7.50818300	2.91112000	-0.61611900
C	-6.48916000	3.57451900	-1.30536600
H	-5.53012100	5.44785000	-1.62818000
H	-8.84117400	5.52353300	1.11360500
H	-5.84134800	3.03795300	-1.99009900
C	-9.35203200	2.68753500	0.88459000
H	-9.26250600	2.66017400	1.97612900
C	-9.00159800	1.36434900	0.25955400
C	-9.58464700	0.12704200	0.43315900
C	-9.08097000	-1.00706900	-0.24012200
C	-7.97922400	-0.81856300	-1.10674800
C	-7.40015000	0.41962200	-1.31204900
C	-7.90915400	1.52111300	-0.61857300
H	-10.42910700	0.01380600	1.10520400
H	-7.58739900	-1.67566100	-1.64341300
H	-6.56627600	0.53106900	-1.99675300
H	2.31441100	-9.32983500	1.97835600
C	4.70450000	-6.49448500	-1.10196800
C	5.41251700	-7.35231100	-0.23042800
C	4.67828800	-8.35146000	0.44262400
C	3.31500400	-8.46346200	0.26318200
C	2.63946000	-7.59884100	-0.62037900
C	3.34253900	-6.61168000	-1.31351000
H	5.25380000	-5.72827500	-1.63806700
H	5.19446600	-9.02513700	1.11893700
H	2.83327700	-5.94660200	-2.00261700
C	2.34064100	-9.42535500	0.88723800
H	2.59898500	-10.46821200	0.67247000
C	1.03310600	-9.02783500	0.25760800
C	-0.22578500	-9.56558000	0.42926400
C	-1.33789400	-9.02352100	-0.24882000
C	-1.10765600	-7.93276900	-1.11701200
C	0.15270800	-7.40027700	-1.32094200
C	1.23177400	-7.94688800	-0.62367900
H	-0.37135600	-10.40373500	1.10303300
H	-1.94829900	-7.51106100	-1.65713200
H	0.29659900	-6.57370500	-2.00849700
H	7.77770500	7.49667200	0.63513600
C	7.43153100	3.00208700	-1.11182600
C	8.49494200	3.35537700	-0.24873700
C	8.40992000	4.59632600	0.41914200
C	7.31504000	5.41565800	0.24383100
C	6.27451500	5.03844000	-0.63038200
C	6.33949200	3.82380800	-1.31863900
H	7.48604600	2.05919500	-1.64487200
H	9.21061300	4.89484200	1.08798400
H	5.54944200	3.52898600	-2.00094500
C	7.00671800	6.75222400	0.86249900
H	6.94400800	6.69260300	1.95466900
C	5.68204900	7.10645500	0.24266400
C	4.90165900	8.22952100	0.41631300
C	3.66357500	8.35676000	-0.25107000
C	3.27353300	7.30422300	-1.11230500
C	4.05692400	6.18445300	-1.31822200
C	5.26929400	6.07866300	-0.63072200
H	5.22793500	9.02006800	1.08425000
H	2.33261800	7.39006900	-1.64477900
H	3.73428500	5.40440800	-1.99934100
H	-1.69889500	6.84970700	1.50662900
C	0.44265300	10.61328300	-0.61454600
C	1.21644000	9.51721600	-0.16340000
C	0.52911800	8.33668400	0.19542000
C	-0.84484900	8.26950100	0.11127700
C	-1.58684800	9.37542600	-0.35629700
C	-0.93304800	10.55409700	-0.72783900
H	0.95319800	11.52734600	-0.89655700
H	1.09272300	7.48268800	0.55711700
H	-1.49254600	11.40772900	-1.09480500
C	-1.77320300	7.13516100	0.45143400
H	-1.55157900	6.23430500	-0.13118600
C	-3.12608400	7.70385900	0.11883800
C	-4.37168200	7.12124200	0.21095000
C	-5.53313200	7.84398200	-0.14069100
C	-5.36425500	9.17491200	-0.59168500
C	-4.12092200	9.76489100	-0.71448600
C	-2.98973800	9.02789600	-0.35086400
H	-4.46874000	6.10269800	0.57286900
H	-6.24482000	9.74458400	-0.86723800
H	-4.02735300	10.78091700	-1.08222300
H	6.80511500	-1.95163500	1.52782800
C	8.97552600	-5.69630500	-0.59516400
C	7.64364000	-5.81535300	-0.13710300
C	6.96718900	-4.63024800	0.22208500
C	7.59670000	-3.40630000	0.13126300
C	8.92033100	-3.31931000	-0.34526500
C	9.61161300	-4.47432500	-0.71676500
H	9.51046100	-6.59645900	-0.87712000
H	5.94724200	-4.68997600	0.58825300
H	10.62854400	-4.41753500	-1.08971500
C	7.08093500	-2.03396800	0.47065100
H	6.18496100	-1.77805000	-0.10554100
C	8.24665900	-1.14726000	0.12510900
C	8.36411600	0.22436100	0.20934200
C	9.56627400	0.86748500	-0.15518200
C	10.63048200	0.05595400	-0.61126300
C	10.51979100	-1.31763900	-0.72561600
C	9.32095100	-1.92690800	-0.34925100
H	7.53284100	0.81850000	0.57529900
H	11.56161700	0.53263400	-0.89701400
H	11.35015800	-1.90815300	-1.09762000

Structure S65. The coordinates of both the optimized structures of B5N1L_3 at S_0 and S_1 in toluene.

S_0-optimized structure				S_1-optimized structure			
B	-9.99475600	-1.23739100	-0.06370900	B	-10.06346100	-0.76677600	-0.05146600
B	-6.03931800	7.96760400	-0.17156500	B	-5.66052700	8.23638400	-0.16934800
C	6.75913700	-9.23737200	0.16954800	C	6.32083000	-9.50899100	0.20013100
C	7.91991700	-9.30401100	0.95731200	C	7.46672800	-9.64005200	1.00467800
C	6.27190800	-10.44413700	-0.35844900	C	5.79346600	-10.69376200	-0.34383000
C	8.55329300	-10.51166000	1.21835500	C	8.04606000	-10.87553300	1.26030000
H	8.32366200	-8.39120500	1.38269300	H	7.90247000	-8.75153300	1.45049000
C	6.91571300	-11.65209200	-0.12593800	C	6.37775700	-11.93114900	-0.10998700
H	5.37895400	-10.43005800	-0.97467200	H	4.91306900	-10.63705300	-0.97573600
C	8.05503600	-11.68846000	0.67036700	C	7.50518900	-12.02742800	0.69891100
H	9.43924400	-10.53594800	1.84378600	H	8.92184800	-10.94187600	1.89756700
H	6.52709500	-12.56689900	-0.56040300	H	5.95290800	-12.82377500	-0.55737000
H	8.55411100	-12.63221400	0.86307500	H	7.95935100	-12.99385400	0.89041100
C	-4.22622000	-10.68092300	0.41690100	C	-4.73202600	-10.46523000	0.37868400
C	-3.47306500	-11.73781800	-0.11987600	C	-4.04081200	-11.55537800	-0.17477600
C	-5.29043900	-11.01877700	1.26875500	C	-5.80885200	-10.75657300	1.23196400
C	-3.77365400	-13.06293600	0.16548000	C	-4.40997300	-12.86588600	0.09801200
H	-2.64575800	-11.51398900	-0.78523900	H	-3.20687000	-11.36848800	-0.84333400
C	-5.57876600	-12.34011300	1.58282600	C	-6.16670900	-12.06409800	1.53238000
H	-5.89354100	-10.22774800	1.70213500	H	-6.36723100	-9.93946900	1.67696400
C	-4.82393400	-13.36594300	1.02480600	C	-5.47065000	-13.12285600	0.95958200
H	-3.18632700	-13.86034800	-0.27701500	H	-3.86813300	-13.68817200	-0.35703200
H	-6.39620600	-12.57195100	2.25709200	H	-6.99265300	-12.25917800	2.20809500
H	-5.05419800	-14.39991000	1.25868600	H	-5.75486400	-14.14566700	1.18311200
C	11.14152200	1.61250100	0.19433900	C	11.23242300	1.09249300	0.15159100
C	11.75431100	2.65512100	-0.50401100	C	11.86649600	2.10446200	-0.57254500
C	11.84874300	0.96220100	1.20780700	C	11.91788700	0.41287400	1.16093300
C	13.04793600	3.04269400	-0.18542300	C	13.18187600	2.43041800	-0.28367700
H	11.21115900	3.15720500	-1.29609900	H	11.33053900	2.61336600	-1.36454100
C	13.14842500	1.34470900	1.50765200	C	13.23343000	0.74812400	1.43929300
H	11.37436200	0.15684800	1.75628100	H	11.41096900	-0.35800500	1.72858400
C	13.75415700	2.38849000	0.81763000	C	13.86870700	1.75540200	0.72046000
H	13.51107000	3.85349200	-0.73692500	H	13.67604000	3.20867500	-0.85319000
H	13.68504800	0.83019000	2.29716300	H	13.76073800	0.22708700	2.22964500
H	14.76725800	2.68897700	1.05899900	H	14.89736300	2.01400800	0.94230400
C	4.72848300	10.36156900	0.52846700	C	5.22417900	10.09111200	0.51041100
C	6.04009800	10.57670800	0.07473800	C	6.53597200	10.25279800	0.03086000
C	4.17781800	11.33107500	1.38239000	C	4.73912000	11.08949800	1.37341200
C	6.76042400	11.70567000	0.44124800	C	7.31448800	11.34737700	0.38167200
H	6.49577400	9.85022200	-0.58993200	H	6.94580600	9.50920100	-0.64522500
C	4.90176700	12.44828300	1.77667000	C	5.51813200	12.17646300	1.74655400
H	3.16698600	11.19727500	1.75322700	H	3.73236500	11.00116600	1.76856400
C	6.19376400	12.64090400	1.30011100	C	6.80894700	12.31199900	1.24690800
H	7.76572200	11.85498200	0.06209600	H	8.31768900	11.44999700	-0.01899000
H	4.45758700	13.17306800	2.45048000	H	5.11781100	12.92187600	2.42580500
H	6.75777400	13.51875700	1.59695400	H	7.41692000	13.16502500	1.52906700
C	-11.52667900	-1.16146800	0.25307300	C	-11.59073500	-0.61991900	0.26367100
C	-12.14580000	-2.09310600	1.10231900	C	-12.25446900	-1.52831100	1.10435900
C	-12.33917600	-0.15874600	-0.30094500	C	-12.35435200	0.42416600	-0.28338100
C	-13.50074300	-2.02039400	1.39635500	C	-13.60512000	-1.39486400	1.39675300
H	-11.54843700	-2.88056100	1.54985100	H	-11.69531300	-2.34649600	1.54606800
C	-13.70046200	-0.09664700	-0.03517000	C	-13.71174300	0.54784700	-0.01904900
H	-11.89501000	0.57628600	-0.96404500	H	-11.87503000	1.14267300	-0.94008700
C	-14.28255500	-1.02471900	0.82114800	C	-14.33832400	-0.35893200	0.82868300
H	-13.94978000	-2.74327500	2.06895100	H	-14.08883200	-2.10159800	2.06245300
H	-14.30776900	0.67820800	-0.49056500	H	-14.28125000	1.35398200	-0.46897900
H	-15.34383900	-0.97234200	1.03965500	H	-15.39643600	-0.25866100	1.04594700
C	-7.15097100	9.02886200	0.13231100	C	-6.72635500	9.34744500	0.12758000
C	-6.90715000	10.12792200	0.97197200	C	-6.44615100	10.42658600	0.98164700
C	-8.43550000	8.91935300	-0.42487100	C	-8.00678300	9.30185200	-0.44776100
C	-7.89580800	11.06136200	1.25309300	C	-7.39503200	11.40231500	1.25683100
H	-5.92687900	10.24316100	1.42252800	H	-5.46885100	10.49225900	1.44838100
C	-9.42058900	9.86464600	-0.17253800	C	-8.95162500	10.28876400	-0.20053100
H	-8.65902700	8.08346700	-1.07957400	H	-8.25903500	8.48206800	-1.11242800
C	-9.15363700	10.93487400	0.67407600	C	-8.64884900	11.33894100	0.65909700
H	-7.68489100	11.89159000	1.91855300	H	-7.15602300	12.21612200	1.93310600
H	-10.39891700	9.76475500	-0.63026800	H	-9.92690000	10.23701400	-0.67269400
H	-9.92466100	11.66898100	0.88233300	H	-9.38851700	12.10581000	0.86317500
B	3.91376800	9.09425200	0.09825600	B	4.34076800	8.86026700	0.10087900
H	6.59673100	-2.68179400	1.56477600	H	6.42144700	-2.91968200	1.52916100
C	8.17069900	-6.57882300	-0.78820900	C	7.88510100	-6.92784400	-0.70410700
C	6.87517100	-6.56125600	-0.23673400	C	6.56205300	-6.84371200	-0.20083700
C	6.36098200	-5.32106600	0.18912700	C	6.09880300	-5.56507400	0.18870800
C	7.11834000	-4.17111300	0.07819000	C	6.91018700	-4.45804500	0.08103800
C	8.40000000	-4.21716900	-0.49595600	C	8.21633800	-4.57249600	-0.44665700
C	8.92806800	-5.42659800	-0.93824600	C	8.70290800	-5.82558100	-0.84356100
H	8.58377100	-7.52446600	-1.12266500	H	8.25947300	-7.89867100	-1.00985900
H	5.36615000	-5.27682600	0.62164300	H	5.09735500	-5.46645100	0.59502800
H	9.91542800	-5.47332600	-1.38539600	H	9.70206600	-5.92965300	-1.25344500
C	6.78743000	-2.76052800	0.48867300	C	6.63310900	-3.02726200	0.45966900
H	5.88951300	-2.38489400	-0.01444000	H	5.76778500	-2.61741900	-0.07228500
C	8.01793800	-1.99719900	0.07800400	C	7.90848600	-2.33002600	0.07142000
C	8.29159100	-0.65011100	0.21798600	C	8.24146900	-1.00572100	0.18681600
C	9.51365100	-0.14783100	-0.24763900	C	9.52256800	-0.58158500	-0.23708200
C	10.44014400	-1.01961500	-0.83853600	C	10.43452100	-1.51946200	-0.77169200
C	10.17122800	-2.37299700	-0.95663100	C	10.09470200	-2.84775800	-0.88269400
C	8.95393100	-2.86856200	-0.50036700	C	8.82524700	-3.27213500	-0.45946800
H	7.57623200	0.01860100	0.68286000	H	7.55463700	-0.29007600	0.62264900
H	11.37754000	-0.62126200	-1.20715500	H	11.39979300	-1.17548000	-1.12153900
H	10.90347500	-3.02785600	-1.41641300	H	10.80098600	-3.55140500	-1.30736800
H	1.35604300	-10.57188100	0.80369100	H	0.86056400	-10.65709500	0.67313200
C	-2.07941600	-7.71928000	-1.04151600	C	-2.44405200	-7.55913700	-1.00785000
C	-2.40956500	-8.75001200	-0.14000500	C	-2.82449500	-8.61571500	-0.15450600
C	-1.35745400	-9.38075900	0.54992300	C	-1.79707400	-9.32875800	0.49754800
C	-0.04853300	-8.98285600	0.35130000	C	-0.47297900	-8.98559900	0.31152600
C	0.24976600	-7.96089100	-0.56432900	C	-0.12529900	-7.93149000	-0.55323500
C	-0.76888800	-7.32814100	-1.27067300	C	-1.11864800	-7.21849700	-1.22313000
H	-2.87656900	-7.22702800	-1.58800500	H	-3.21755600	-7.00546800	-1.52912600

H	-1.58200400	-10.17840000	1.25105900
H	-0.54690600	-6.54303800	-1.98565700
C	1.21690000	-9.49963200	0.98076600
H	1.21731900	-9.36158500	2.06791100
C	2.28179100	-8.67321200	0.31169600
C	3.65542800	-8.71680200	0.46243600
C	4.48184300	-7.83347700	-0.25634000
C	3.86377300	-6.92182900	-1.13408000
C	2.48971500	-6.88827200	-1.31882300
C	1.69648200	-7.76699800	-0.58720500
H	4.10441100	-9.42947600	1.14719800
H	4.48618300	-6.23597600	-1.69865600
H	2.04647000	-6.18810500	-2.01896000
H	7.49127200	5.45918000	2.12050800
C	4.19965000	6.82895500	-1.11751500
C	4.65094100	7.73880700	-0.14216500
C	5.79836100	7.39088100	0.59665700
C	6.44285900	6.19054900	0.37015900
C	5.98366200	5.31366000	-0.62737100
C	4.85740000	5.63588200	-1.37933300
H	3.31793500	7.07938300	-1.69785000
H	6.16804900	8.07024000	1.35845100
H	4.49467500	4.96789600	-2.15347400
C	7.65618700	5.61193200	1.04805600
H	8.53038000	6.26614500	0.95787400
C	7.85474800	4.31071800	0.31918200
C	8.82358500	3.34582000	0.51556000
C	8.82759200	2.20321600	-0.29447600
C	7.84490400	2.05388300	-1.28437400
C	6.86169200	3.01323100	-1.46305400
C	6.86388000	4.15115300	-0.66179900
H	9.57823100	3.46262700	1.28488600
H	7.86371900	1.17555700	-1.91812000
H	6.11346200	2.87687000	-2.23645000
H	-9.92984400	4.19712200	0.65711600
C	-5.72990600	5.63312900	-1.25572400
C	-6.43555700	6.46689000	-0.36658200
C	-7.51457100	5.90735600	0.34238300
C	-7.84963000	4.57644700	0.17662000
C	-7.13577700	3.77376600	-0.72758700
C	-6.07454900	4.30460600	-1.45500200
H	-4.89914200	6.04924700	-1.81548700
H	-8.07695400	6.52673200	1.03401800
H	-5.52218100	3.69410300	-2.16140200
C	-8.93613100	3.77223800	0.83752800
H	-8.80867000	3.73109700	1.92513900
C	-8.77762100	2.41688900	0.20356900
C	-9.51092200	1.26161200	0.40026200
C	-9.18041400	0.07918500	-0.28727500
C	-8.09679900	0.12205000	-1.18603600
C	-7.37204900	1.28185900	-1.41619100
C	-7.71190700	2.43240300	-0.71062600
H	-10.34113000	1.25780900	1.09958100
H	-7.83124500	-0.77885800	-1.72867300
H	-6.55442700	1.28629600	-2.12919500
H	-5.72762400	-4.24596400	1.61121300
C	-9.96781400	-3.76405500	-0.62463700
C	-9.28331900	-2.62708800	-0.15268200
C	-7.94028300	-2.78321200	0.23758600
C	-7.32847400	-4.02110800	0.16944600
C	-8.03266400	-5.13262700	-0.32068400
C	-9.35755100	-5.00494400	-0.72731600
H	-11.00291900	-3.66162300	-0.93211300
H	-7.38916300	-1.92451000	0.60804300
H	-9.90710000	-5.85749500	-1.11199600
C	-5.92805300	-4.42123800	0.54819500
H	-5.17335000	-3.85386600	-0.00772800
C	-5.89793800	-5.88663000	0.20698000
C	-4.86246100	-6.79606200	0.31708900
C	-5.05172100	-8.14245300	-0.04611600
C	-6.31849500	-8.52071800	-0.53200400
C	-7.35693200	-7.61307100	-0.67570800
C	-7.14446100	-6.29078300	-0.29752900
H	-3.89761000	-6.47664600	0.69841600
H	-6.48183800	-9.55414100	-0.81797600
H	-8.31535900	-7.93276700	-1.07071300
H	-0.91943000	6.93140400	1.47575900
C	1.76065000	10.37301600	-0.56263300
C	2.36304300	9.19295100	-0.08512000
C	1.52456600	8.10981800	0.23630500
C	0.15250600	8.22097000	0.10455400
C	-0.41743800	9.40564600	-0.38835800
C	0.38927600	10.48585000	-0.73409100
H	2.39246900	11.21746900	-0.81629700
H	1.96247400	7.18858400	0.60787900
H	-0.04254300	11.40296500	-1.12044000
C	-0.92585900	7.21926700	0.41850500
H	-0.80742800	6.29374000	-0.15600400
C	-2.18335200	7.95737700	0.04592800
C	-3.50081100	7.54336600	0.11158000
C	-4.54277600	8.40943000	-0.26966900
C	-4.19379800	9.69306200	-0.73324500
C	-2.87562800	10.11217900	-0.82917500
C	-1.86732300	9.24087900	-0.42776600
H	-3.73989700	6.54914100	0.47573600
H	-4.98313900	10.37218500	-1.03667000
H	-2.64020100	11.10277700	-1.20350700
N	9.82017200	1.22024000	-0.12174200
B	-3.89507700	-9.18792200	0.07769300
B	6.03897200	-7.87359700	-0.10622800

H	-2.05688300	-10.14598000	1.16318800
H	-0.86255300	-6.41065500	-1.90057700
C	0.76934900	-9.59096200	0.90908700
H	0.77561100	-9.51363100	2.00223000
C	1.87119500	-8.77741600	0.28313400
C	3.23866700	-8.88281600	0.43802100
C	4.11385600	-7.99755100	-0.22880300
C	3.52758500	-7.02000100	-1.06469100
C	2.15967400	-6.91830600	-1.24948700
C	1.32205800	-7.80071200	-0.56750600
H	3.65234700	-9.64419800	1.09198200
H	4.17829400	-6.33799100	-1.60172700
H	1.75009600	-6.16646500	-1.91633000
H	7.86284400	5.14320700	2.08383200
C	4.48874000	6.54113000	-1.05693700
C	5.01289000	7.47053500	-0.12466800
C	6.18316600	7.09562000	0.57464500
C	6.77296600	5.87064400	0.35639300
C	6.23740300	4.97452400	-0.59528700
C	5.08238200	5.32048500	-1.30843200
H	3.59688100	6.81254800	-1.61110600
H	6.60686900	7.78029200	1.30229200
H	4.66140300	4.64523900	-2.04591100
C	7.99136600	5.26685900	1.00288400
H	8.88462400	5.88523300	0.86321800
C	8.11441700	3.94159800	0.30187200
C	9.05009000	2.95476300	0.47355000
C	8.95418200	1.77808400	-0.30287500
C	7.91255500	1.63741100	-1.24594700
C	6.97527600	2.63195800	-1.41264900
C	7.06065200	3.79600200	-0.63511400
H	9.84025400	3.05222100	1.20866500
H	7.87808700	0.74802800	-1.86281500
H	6.19441700	2.51723900	-2.15539700
H	-9.73173700	4.66121700	0.66627700
C	-5.47251700	5.88832800	-1.25773500
C	-6.13138200	6.75590500	-0.36482400
C	-7.23486800	6.24978800	0.34671800
C	-7.63620000	4.93712200	0.18193600
C	-6.96528400	4.09949300	-0.72338800
C	-5.88225100	4.57812000	-1.45506700
H	-4.62533400	6.26358200	-1.82181900
H	-7.76422300	6.89639600	1.03959600
H	-5.36327000	3.94146700	-2.16382600
C	-8.75991600	4.18791900	0.84563800
H	-8.63303600	4.14165400	1.93313100
C	-8.66964600	2.82548100	0.21313500
C	-9.45824900	1.70773300	0.41254100
C	-9.18672700	0.50923500	-0.27333100
C	-8.10299300	0.49781600	-1.17286000
C	-7.32335400	1.62072300	-1.40644000
C	-7.60593100	2.78797500	-0.70306800
H	-10.28673600	1.74556300	1.11296100
H	-7.88162500	-0.41618700	-1.71343400
H	-6.50747600	1.58378800	-2.12051400
H	-5.95983400	-3.98765400	1.63862400
C	-10.14975000	-3.28795900	-0.63169700
C	-9.41811900	-2.18785600	-0.14365400
C	-8.08725700	-2.40957900	0.25689800
C	-7.53269800	-3.67362400	0.18308800
C	-8.28158200	-4.74723000	-0.32578300
C	-9.59558900	-4.55433600	-0.74242000
H	-11.17645300	-3.13537300	-0.94668200
H	-7.50033100	-1.58032100	0.63937900
H	-10.18005400	-5.37660300	-1.14140800
C	-6.15598400	-4.14112600	0.57140100
H	-5.37037300	-3.60169300	0.03070500
C	-6.18796300	-5.60224200	0.21217200
C	-5.19454200	-6.55764100	0.32084000
C	-5.43595200	-7.88918100	-0.06575200
C	-6.71389400	-8.20444100	-0.56780600
C	-7.71154300	-7.25130300	-0.70654400
C	-7.44616500	-5.94406200	-0.30906200
H	-4.22163500	-6.28600200	0.71832800
H	-6.91898200	-9.22572800	-0.87029900
H	-8.67921800	-7.52448700	-1.11401300
H	-0.59537100	6.89012400	1.42177300
C	2.24861200	10.25975700	-0.50920900
C	2.80012200	9.03457100	-0.07337300
C	1.90000900	7.98733100	0.21699000
C	0.53727900	8.16985900	0.08907300
C	0.02241800	9.39856700	-0.36095800
C	0.88633300	10.44832500	-0.66954100
H	2.92077000	11.07880700	-0.74161600
H	2.28750300	7.03586100	0.56835900
H	0.50201600	11.39944100	-1.02321200
C	-0.59002000	7.21338400	0.37474700
H	-0.52088000	6.30274900	-0.23092200
C	-1.81069700	8.02543700	0.03244900
C	-3.14547100	7.67672700	0.09159700
C	-4.14745000	8.60486300	-0.25779200
C	-3.73287600	9.88423800	-0.68184300
C	-2.39681800	10.24072500	-0.76879500
C	-1.42900200	9.30801600	-0.40024500
H	-3.43234400	6.68524400	0.42779400
H	-4.48759300	10.61074500	-0.96302500
H	-2.11367000	11.23010200	-1.11226800
N	9.88769300	0.75719600	-0.13774500
B	-4.32485600	-8.98636800	0.05253700
B	5.66157300	-8.11189900	-0.07648100

Structure S66. The coordinates of both the optimized structures of B4N2L$_3$ at S$_0$ and S$_1$ in toluene.

S$_0$-optimized structure				S$_1$-optimized structure			
B	-8.75503300	-4.93563300	-0.18776800	B	-8.91958100	-4.63577300	-0.17311800
B	-8.66686800	5.08232600	-0.20072600	B	-8.48844100	5.37902400	-0.31590500
C	9.55708700	-5.77214600	0.10303600	C	9.32141100	-6.11780300	-0.08973900
C	10.52421900	-5.40039400	1.04019100	C	10.27704000	-5.95992300	0.91460100
C	9.68029800	-7.00170700	-0.54923200	C	9.36018300	-7.23860900	-0.91908100
C	11.59414500	-6.24101900	1.31153300	C	11.27182200	-6.91355800	1.07529500
H	10.43163000	-4.45009000	1.55266400	H	10.22998100	-5.09754200	1.56939000
C	10.74427700	-7.84330800	-0.25834800	C	10.35329600	-8.19064800	-0.74390000
H	8.93941600	-7.29192900	-1.28486100	H	8.61586300	-7.35484900	-1.69786200
C	11.70953300	-7.46849700	0.66932600	C	11.31327400	-8.03120000	0.24958200
H	12.33591000	-5.93690600	2.04185600	H	12.00895700	-6.78810900	1.86006600
H	10.82549400	-8.79376400	-0.77429400	H	10.38123600	-9.05746900	-1.39402400
H	12.54318200	-8.12557500	0.88876200	H	12.08842800	-8.77691600	0.38195200
C	0.22610000	-11.32818900	0.68111600	C	-0.11611100	-11.29753400	0.77148300
C	1.33949200	-12.05305100	0.22589000	C	0.98565900	-12.04095400	0.31649600
C	-0.64960500	-11.98358600	1.56196200	C	-0.99657800	-11.93765000	1.65952300
C	1.56180500	-13.36644000	0.61758400	C	1.19114800	-13.35556400	0.71272100
H	2.03452600	-11.58001600	-0.45998600	H	1.68516100	-11.58120100	-0.37406400
C	-0.41881400	-13.28687200	1.98131800	C	-0.78351000	-13.24298800	2.08189600
H	-1.52078800	-11.45422000	1.93359200	H	-1.85724400	-11.39377300	2.03441600
C	0.68551100	-13.98361500	1.50339400	C	0.30887100	-13.95780500	1.60315800
H	2.42090500	-13.90846200	0.23701000	H	2.04116900	-13.91122500	0.33112500
H	-1.10297000	-13.76291200	2.67553700	H	-1.47227200	-13.70622500	2.78025400
H	0.86233600	-15.00609800	1.81979900	H	0.47184100	-14.98165000	1.92255000
C	9.65773200	5.61099200	0.10968500	C	9.82810600	5.31415400	0.01957200
C	9.80157500	6.83979100	-0.53983000	C	9.96526900	6.45635800	-0.77016200
C	10.61863000	5.22095600	1.04583200	C	10.76106000	5.03864300	1.02057100
C	10.87952000	7.66275200	-0.24717700	C	11.03671200	7.31068600	-0.56036800
H	9.06564100	7.14418600	-1.27470000	H	9.23744200	6.66222100	-1.54570100
C	11.70260400	6.04285700	1.31883000	C	11.83189500	5.89754600	1.21762400
H	10.51022000	4.27119600	1.55620200	H	10.63408300	4.16319800	1.64638700
C	11.83851000	7.26970500	0.67946500	C	11.97337400	7.03476300	0.43031000
H	10.97659800	8.61290900	-0.76093300	H	11.14318500	8.19359500	-1.17969300
H	12.43926200	5.72455600	2.04828200	H	12.54988400	5.68403300	2.00089200
H	12.68310600	7.91217300	0.90028600	H	12.80791600	7.70705700	0.59116200
C	0.41884100	11.32125000	0.68729100	C	0.85475800	11.26443600	0.80410200
C	1.54463000	12.02826900	0.23449000	C	2.01395700	11.91201900	0.34098300
C	-0.44741200	11.98993900	1.56749600	C	0.05465200	11.98199100	1.71098700
C	1.78767000	13.33751600	0.62779400	C	2.34912300	13.19738400	0.74475000
H	2.23288700	11.54461500	-0.45080200	H	2.65889400	11.39614600	-0.36350200
C	-0.19610300	13.28890700	1.98842800	C	0.39063900	13.26112100	2.13429700
H	-1.32763800	11.47445800	1.93729200	H	-0.84472800	11.51627400	2.10058000
C	0.92014800	13.96801900	1.51284200	C	1.53894900	13.87722400	1.64842100
H	2.65605300	13.86588800	0.24907000	H	3.24391000	13.67198700	0.35474000
H	-0.87348200	13.77541300	2.68204900	H	-0.24490300	13.78026600	2.84444600
H	1.11306300	14.98721100	1.83044600	H	1.80074700	14.87928200	1.97161500
C	-10.19717300	-5.46537200	0.11762900	C	-10.37513400	-5.12160900	0.15792800
C	-10.41217600	-6.55158800	0.98144900	C	-10.61512400	-6.16646000	1.06549900
C	-11.33057600	-4.87076900	-0.46059000	C	-11.49778000	-4.52909500	-0.44354500
C	-11.69075300	-7.01233300	1.26556700	C	-11.90289900	-6.58907100	1.36765300
H	-9.55977100	-7.03346400	1.44877700	H	-9.77304300	-6.64720300	1.55273200
C	-12.61038200	-5.34444400	-0.20491300	C	-12.78762200	-4.96270000	-0.16692000
H	-11.20168600	-4.02989400	-1.13412700	H	-11.35181600	-3.72003700	-1.15178200
C	-12.79284400	-6.41313500	0.66575700	C	-12.99363400	-5.99129500	0.74585700
H	-11.82897100	-7.84250400	1.94990300	H	-12.05726800	-7.38819600	2.08506200
H	-13.46697900	-4.87736500	-0.67894300	H	-13.63406000	-4.49530600	-0.65895300
H	-13.79235900	-6.77828900	0.87657500	H	-14.00067200	-6.32552400	0.97201900
C	-10.10001500	5.63733400	0.10219300	C	-9.92775100	5.96745500	-0.06981000
C	-10.29729500	6.72880000	0.96362400	C	-10.15982100	7.01421900	0.83828400
C	-11.24288000	5.06067800	-0.47558400	C	-11.04963800	5.45745700	-0.74515200
C	-11.56813200	7.21167600	1.24578000	C	-11.43358000	7.52042900	1.06358100
H	-9.43724500	7.19713500	1.43070400	H	-9.32194900	7.42977300	1.38868600
C	-12.51468300	5.55645100	-0.22191800	C	-12.32382800	5.97108900	-0.54264700
H	-11.12770800	4.21623400	-1.14713800	H	-10.91375000	4.64534500	-1.45234800
C	-12.67973000	6.62992200	0.64635300	C	-12.52100100	7.00394000	0.36766300
H	-11.69278600	8.04548200	1.92829400	H	-11.57890900	8.32067200	1.78199900
H	-13.37872000	5.10287200	-0.69556100	H	-13.16617900	5.56334100	-1.09209000
H	-13.67308600	7.01236000	0.85562400	H	-13.51629000	7.40203600	0.53531600
B	0.13757400	9.84933000	0.22922100	B	0.47708600	9.80943800	0.34296100
H	7.23609300	-0.06038000	1.65482800	H	6.90763800	-0.29200000	1.40775100
C	9.82920400	-3.08962700	-1.06656100	C	9.86731800	-3.39517200	-0.80920700
C	8.71447300	-3.52888300	-0.34170000	C	8.60098900	-3.80651000	-0.32728100
C	7.84436700	-2.59117300	0.22777300	C	7.65939000	-2.83206300	0.07038000
C	8.09711100	-1.24398300	0.05432400	C	7.99161300	-1.50429400	-0.02233400
C	9.22120200	-0.80648100	-0.66401600	C	9.25704800	-1.09803700	-0.50664200
C	10.09162400	-1.73725500	-1.22131800	C	10.19370200	-2.06314500	-0.89878800
H	10.49145700	-3.82369400	-1.50985700	H	10.57411900	-4.14572700	-1.13743500
H	6.98588500	-2.93113200	0.79628900	H	6.69766800	-3.14131600	0.46011800
H	10.96211000	-1.42034200	-1.78549700	H	11.16157100	-1.77149900	-1.28974500
C	7.31690600	-0.06054800	0.56223300	C	7.17522800	-0.29573100	0.34601700
H	6.29225700	-0.05109100	0.17445900	H	6.23620200	-0.24960000	-0.21474100
C	8.11834600	1.10917900	0.05557000	C	8.09533600	0.84194100	-0.00309300
C	7.88978600	2.46046200	0.23038800	C	7.88203000	2.19081500	0.10955700
C	8.77655900	3.38300000	-0.33808400	C	8.91115600	3.08389700	-0.26848300
C	9.88341900	2.92467100	-1.06324900	C	10.13919500	2.56922600	-0.75378900
C	10.12160500	1.56794400	-1.21948100	C	10.34370700	1.21572300	-0.86747600
C	9.23444100	0.65235800	-0.66323500	C	9.32142000	0.33217000	-0.49415500
H	7.03741600	2.81518900	0.79907500	H	6.95159600	2.58011100	0.50311600
H	10.55871000	3.64727600	-1.50570900	H	10.91005300	3.25959500	-1.06963700
H	10.98632100	1.23610500	-1.78395600	H	11.28236300	0.84468400	-1.26198300
H	5.34290800	-9.05816500	0.95830600	H	5.03003900	-9.11929400	1.08410700
C	1.09216800	-7.87958400	-1.01571000	C	0.82903400	-7.91021200	-0.97924700
C	1.17233600	-8.89384900	-0.04194400	C	0.87703600	-8.88963400	0.03288700
C	2.38063700	-9.02183700	0.67082100	C	2.07373500	-9.01991600	0.76234400
C	3.43799400	-8.16919600	0.42136400	C	3.15413500	-8.20218900	0.49092000
C	3.33486800	-7.17906700	-0.57068400	C	3.08258100	-7.24953500	-0.53898300
C	2.15639500	-7.03575200	-1.29763300	C	1.91431100	-7.10284200	-1.28229200
H	0.17168300	-7.76707000	-1.57855500	H	-0.08413600	-7.79819100	-1.55350300

H	2.47196200	-9.79163800	1.43081600	H	2.13990600	-9.76481200	1.54909600
H	2.06615100	-6.27814400	-2.06899900	H	1.84890600	-6.37285100	-2.08220400
C	4.79744000	-8.11414000	1.06615700	C	4.50896500	-8.15766400	1.14531900
H	4.73264600	-7.91344100	2.14142600	H	4.44210100	-7.90665600	2.20977900
C	5.47157300	-6.99141300	0.32485700	C	5.21936400	-7.08816400	0.36236000
C	6.74587400	-6.48488400	0.49066300	C	6.50949400	-6.61882400	0.50200600
C	7.17626300	-5.42960400	-0.32451900	C	6.96071900	-5.61257000	-0.36008200
C	6.30215800	-4.89806700	-1.28491500	C	6.11322800	-5.09386100	-1.34747200
C	5.01821800	-5.39582900	-1.43404700	C	4.81487400	-5.55811200	-1.47571200
C	4.59706000	-6.45146400	-0.63104600	C	4.36378300	-6.56126300	-0.62129500
H	7.41444100	-6.89296000	1.23998900	H	7.17283000	-7.01516400	1.26230300
H	6.64503900	-4.09080200	-1.92059400	H	6.48888600	-4.33667000	-2.02542200
H	4.36225700	-4.97074200	-2.18607700	H	4.17243800	-5.15510000	-2.25039400
H	4.86755300	7.83065300	2.14697500	H	5.10444000	7.51932500	2.26104000
C	1.22974800	7.86108600	-1.01334900	C	1.51609800	7.80367100	-0.94151700
C	1.32579100	8.87259200	-0.03814900	C	1.63040200	8.78069700	0.07375000
C	2.53536900	8.97972800	0.67588500	C	2.83693700	8.81243800	0.80404400
C	3.57875800	8.11007500	0.42624400	C	3.85035200	7.91437200	0.53530800
C	3.46015200	7.12310800	-0.56722700	C	3.70976600	6.96518700	-0.49363100
C	2.28011000	7.00011800	-1.29534200	C	2.53117000	6.91182500	-1.23910400
H	0.30801600	7.76453000	-1.57709400	H	0.60175800	7.76856300	-1.52369900
H	2.63864900	9.74693200	1.43696800	H	2.95967400	9.54932000	1.59175500
H	2.17815000	6.24499500	-2.06770400	H	2.41032300	6.19007300	-2.04059700
C	4.93659400	8.03171100	1.07203800	C	5.19512100	7.76303700	1.19660500
H	5.49768000	8.96674700	0.96599200	H	5.79202400	8.67971000	1.13707900
C	5.59270700	6.89894000	0.32981200	C	5.82267000	6.63866000	0.41877200
C	6.85832300	6.37110600	0.49593500	C	7.06688200	6.06683800	0.56466500
C	7.27168300	5.30982900	-0.32021800	C	7.43800800	5.02225500	-0.29540600
C	6.38974100	4.79422500	-1.28214300	C	6.55650300	4.57531700	-1.28959400
C	5.11441700	5.31345400	-1.43169600	C	5.30301000	5.14434900	-1.42566800
C	4.71016300	6.37479100	-0.62749200	C	4.92747400	6.18200700	-0.57101200
H	7.53298300	6.76702100	1.24631300	H	7.75555300	6.40552700	1.33037600
H	6.71975600	3.98219800	-1.91856100	H	6.87449600	3.79221100	-1.96772700
H	4.45200000	4.90037900	-2.18476700	H	4.63513800	4.79732700	-2.20577000
H	-10.82479300	0.09303800	0.53063400	H	-10.88278200	0.47208700	0.32128500
C	-7.48460400	3.06083700	-1.31376900	C	-7.35401600	3.29530400	-1.38725200
C	-8.46240200	3.54761700	-0.42460400	C	-8.34020200	3.83622100	-0.53404400
C	-9.25088200	2.60727400	0.26373600	C	-9.19720000	2.92532400	0.11789000
C	-9.04830900	1.25205400	0.08067300	C	-9.04600900	1.56334400	-0.05206400
C	-8.07354200	0.79706600	-0.82168000	C	-8.05127900	1.05680500	-0.90720000
C	-7.29211800	1.70467900	-1.53105800	C	-7.20715000	1.93273400	-1.58905500
H	-6.87165900	3.77149600	-1.85780400	H	-6.69648700	3.97612100	-1.91745100
H	-10.01266600	2.95391500	0.95497000	H	-9.97165000	3.30611700	0.77677400
H	-6.54186900	1.36256300	-2.23598700	H	-6.44467100	1.55759900	-2.26423700
C	-9.74606500	0.08378500	0.72267000	C	-9.81624600	0.42862000	0.56976100
H	-9.62428200	0.08419800	1.81166300	H	-9.75212700	0.44125400	1.66361700
C	-9.06910700	-1.09836000	0.08382800	C	-9.15070000	-0.78769700	-0.01724400
C	-9.29550900	-2.44932200	0.27056100	C	-9.42073700	-2.12489600	0.19128200
C	-8.52399800	-3.40529600	-0.41551200	C	-8.64686000	-3.12471500	-0.43545100
C	-7.53824500	-2.93814400	-1.30638400	C	-7.61466900	-2.69930700	-1.29835600
C	-7.32183600	-1.58620200	-1.52719300	C	-7.35091900	-1.36104700	-1.54023300
C	-8.08664000	-0.66303100	-0.81988400	C	-8.11561800	-0.39554600	-0.88608100
H	-10.06299600	-2.78064500	0.96298800	H	-10.22237500	-2.41701900	0.86265300
H	-6.93820700	-3.66092900	-1.84881100	H	-7.01596400	-3.44965500	-1.80397800
H	-6.56582300	-1.25935300	-2.23318400	H	-6.55778900	-1.07280200	-2.22238600
H	-3.65391400	-5.95367000	1.52959000	H	-3.83052800	-5.76247200	1.51855900
C	-7.73005200	-7.26045400	-0.68535400	C	-7.97397100	-7.01305900	-0.60088800
C	-7.55216900	-5.93231500	-0.25089800	C	-7.75021300	-5.67459900	-0.21735200
C	-6.25773600	-5.53498300	0.13326400	C	-6.43723200	-5.31173200	0.14189400
C	-5.20726100	-6.43296900	0.09733800	C	-5.41764600	-6.24400800	0.12373700
C	-5.41487800	-7.74812300	-0.34865900	C	-5.67190600	-7.56841900	-0.27011000
C	-6.68048300	-8.16415500	-0.75157400	C	-6.95775600	-7.95443300	-0.64249400
H	-8.71963900	-7.58306400	-0.99045400	H	-8.97689400	-7.31298700	-0.88517800
H	-6.09148200	-4.51832800	0.47530000	H	-6.23370100	-4.29167700	0.45185600
H	-6.84842000	-9.17600500	-1.10476000	H	-7.16508200	-8.97247500	-0.95535100
C	-3.76427100	-6.23678900	0.47676500	C	-3.96395800	-6.08041300	0.47836300
H	-3.28972400	-5.44223300	-0.10985700	H	-3.47277200	-5.32322600	-0.14306900
C	-3.16032800	-7.58579100	0.19374300	C	-3.40626200	-7.45771000	0.23857500
C	-1.85347900	-8.01410200	0.33784200	C	-2.11337400	-7.92153900	0.38183100
C	-1.50044700	-9.34336100	0.04140700	C	-1.80336800	-9.27412500	0.13477200
C	-2.51361900	-10.20626300	-0.41936800	C	-2.85311200	-10.12031300	-0.27777100
C	-3.82079100	-9.78091400	-0.60194100	C	-4.14752000	-9.66043300	-0.46070600
C	-4.14466700	-8.46487200	-0.28547700	C	-4.42750200	-8.32205300	-0.19354100
H	-1.09320100	-7.32801600	0.69806500	H	-1.32810000	-7.24597100	0.70705100
H	-2.25908400	-11.23477500	-0.65184100	H	-2.63519600	-11.16431400	-0.47603200
H	-4.57420100	-10.46598600	-0.97602300	H	-4.92698100	-10.33497100	-0.79901200
H	-3.55177400	6.01135800	1.52272900	H	-3.29141900	5.95901900	1.31130800
C	-2.33691400	10.24625200	-0.41949200	C	-2.00103700	10.35250600	-0.16687700
C	-1.33923000	9.36608700	0.04240100	C	-1.00292600	9.39962300	0.15606700
C	-1.71489700	8.04256900	0.33675300	C	-1.43195700	8.05650200	0.30499600
C	-3.02838700	7.63623500	0.18938800	C	-2.75513300	7.71033600	0.15168300
C	-3.99682200	8.53204100	-0.29126900	C	-3.72035200	8.68240400	-0.18880700
C	-3.65055800	9.84285800	-0.60543500	C	-3.32796300	10.01559500	-0.35317300
H	-2.06482300	11.27062800	-0.65028400	H	-1.70392600	11.38789400	-0.29702300
H	-0.96687900	7.34364900	0.69793900	H	-0.70394300	7.29630800	0.57217400
H	-4.39167800	10.54071300	-0.98043500	H	-4.05338500	10.77703300	-0.62160600
C	-3.65550600	6.29733600	0.47001000	C	-3.41916600	6.36590800	0.30167900
H	-3.19335300	5.49549200	-0.11660600	H	-3.00284100	5.62210200	-0.38724800
C	-5.09428300	6.51816700	0.08812000	C	-4.86344500	6.66922000	-0.00291800
C	-6.16006400	5.63833600	0.12223400	C	-5.95118400	5.82568700	-0.01951700
C	-7.44685600	6.05785900	-0.26406600	C	-7.24862600	6.30955500	-0.31889700
C	-7.60102500	7.38868500	-0.69938800	C	-7.36108700	7.69213300	-0.60349900
C	-6.53604500	8.27428500	-0.76367500	C	-6.27734200	8.55001100	-0.60176300
C	-5.27865000	7.83675500	-0.35789900	C	-5.01099700	8.04274000	-0.29125200
H	-6.01190100	4.61908100	0.46484300	H	-5.82215400	4.77579400	0.22435600
H	-8.58431800	7.72814500	-1.00654200	H	-8.33927800	8.09115100	-0.85021600
H	-6.68590500	9.28883200	-1.11730800	H	-6.41043700	9.60078800	-0.83870800
N	8.56199200	4.76906800	-0.17992200	N	8.72349600	4.44705000	-0.18041000
N	8.47572800	-4.91109600	-0.18475600	N	8.29449700	-5.15154400	-0.25605100
B	-0.03158900	-9.85123200	0.22530200	B	-0.35359800	-9.81785400	0.31347000

Structure S67. The coordinates of both the optimized structures of B3N3L$_3$ at S$_0$ and S$_1$ in toluene.

S_0-optimized structure				S_1-optimized structure			
B	-6.27826700	-7.67606900	-0.17832300	B	9.46062600	-3.05409400	-0.43811800
B	-9.88425400	1.67360600	-0.08405900	B	2.02550800	-9.75355300	0.05463800
C	10.85129600	-2.03562600	0.33614400	C	-2.03667600	10.74830400	0.55913400
C	11.59254800	-1.30180800	1.26683500	C	-2.89422500	11.05719100	1.61510600
C	11.41508800	-3.18705500	-0.22124500	C	-1.30096600	11.75879400	-0.06138400
C	12.86973700	-1.70978900	1.62292800	C	-3.00931800	12.37020500	2.04656600
H	11.16116400	-0.41145200	1.70877400	H	-3.45990500	10.26684400	2.09390900
C	12.68618500	-3.59628900	0.15477200	C	-1.41746900	13.06737600	0.38332700
H	10.85227900	-3.75710300	-0.95088700	H	-0.65218300	11.51508900	-0.89452400
C	13.42435800	-2.86064300	1.07486000	C	-2.27112200	13.37814700	1.43606400
H	13.42924700	-1.12766300	2.34707300	H	-3.67345000	12.60453200	2.87039400
H	13.10739800	-4.49172100	-0.28922000	H	-0.84887200	13.84980100	-0.10589100
H	14.41998200	-3.17991000	1.36064200	H	-2.36297300	14.40247300	1.77767200
C	4.15756700	-10.29111700	0.49577300	C	8.09803800	7.53986800	0.66449700
C	5.35610700	-10.63806500	-0.13258200	C	8.08979100	8.81841300	0.10337700
C	3.66307000	-11.10864600	1.51510900	C	8.97365400	7.24422400	1.71025800
C	6.04654600	-11.77594700	0.25930500	C	8.94205300	9.79401300	0.59867800
H	5.74092600	-10.01138400	-0.92850400	H	7.42692600	9.03702600	-0.72568200
C	4.35140900	-12.25410200	1.88808100	C	9.82655700	8.22527900	2.19247900
H	2.73785900	-10.84072400	2.01164800	H	8.97756800	6.24993600	2.14025900
C	5.54822500	-12.59329300	1.26738500	C	9.81300300	9.50261600	1.64267200
H	6.97501900	-12.03148600	-0.23952300	H	8.93609400	10.78261200	0.15413900
H	3.95376400	-12.87779800	2.68118300	H	10.50104500	7.99022000	3.00762700
H	6.08690400	-13.48520000	1.56609700	H	10.48248200	10.26559000	2.02231000
C	6.98051000	8.64734100	0.04391000	C	-10.63140500	3.35254000	-0.37748300
C	6.70401000	9.84228600	-0.62526200	C	-11.52852600	2.64850900	-1.18292000
C	7.99792300	8.62420900	1.00090600	C	-11.12199300	4.27234400	0.55179100
C	7.42549200	10.99049300	-0.33197500	C	-12.89349100	2.86172900	-1.05538300
H	5.92242200	9.86433000	-1.37557800	H	-11.14991300	1.93606000	-1.90629800
C	8.72615300	9.77310300	1.27454600	C	-12.48780100	4.49084000	0.66073400
H	8.21379700	7.70168300	1.52689000	H	-10.42864000	4.81001500	1.18797700
C	8.44305400	10.96356400	0.61490400	C	-13.38081800	3.78576800	-0.13811900
H	7.19850700	11.90960500	-0.86104500	H	-13.57911300	2.30862200	-1.68758100
H	9.51279700	9.73785900	2.02027900	H	-12.85572500	5.20683300	1.38724400
H	9.00964700	11.86074800	0.83612900	H	-14.44758700	3.95348100	-0.04505200
C	-3.67066800	10.81063300	0.57132900	C	-8.80023800	-7.28673200	0.68478700
C	-2.86568100	11.85665200	0.09144100	C	-10.04196600	-6.91440300	0.14393600
C	-4.71371500	11.15352200	1.44700500	C	-8.79521300	-8.35584400	1.59580600
C	-3.09636900	13.17689700	0.45404900	C	-11.21270700	-7.58145500	0.47909500
H	-2.05311400	11.62873800	-0.59048300	H	-10.08503700	-6.09391500	-0.56498200
C	-4.93312000	12.46779900	1.83722200	C	-9.96491200	-9.01157000	1.95643600
H	-5.35546800	10.37091000	1.83807500	H	-7.85480300	-8.66904900	2.03706800
C	-4.12755800	13.48378400	1.33488400	C	-11.17710000	-8.62935700	1.39258200
H	-2.46959400	13.96713600	0.05483900	H	-12.15490200	-7.28273300	0.03174800
H	-5.73606300	12.70230800	2.52781500	H	-9.93157600	-9.82513100	2.67332400
H	-4.30366200	14.51311100	1.62869900	H	-12.09123600	-9.14671800	1.66466600
C	-7.43376500	-8.69942600	0.09234200	C	10.86789100	-3.72153600	-0.21930700
C	-7.24184500	-9.81960900	0.91734000	C	11.86649000	-3.10741000	0.55737800
C	-8.70584200	-8.53396800	-0.47928700	C	11.19859800	-4.95855300	-0.80072700
C	-8.26773400	-10.72029000	1.17077900	C	13.10924300	-3.69407000	0.75599900
H	-6.27233400	-9.97759600	1.37810600	H	11.65487600	-2.15102000	1.02529700
C	-9.72844200	-9.44585800	-0.25448800	C	12.44399400	-5.54582100	-0.62148200
H	-8.88963800	-7.68045000	-1.12353100	H	10.46169700	-5.46169000	-1.41833900
C	-9.51233800	-10.53832700	0.57813700	C	13.40503200	-4.91734200	0.16361600
H	-8.09633900	-11.56809500	1.82538100	H	13.85066000	-3.19687900	1.37341800
H	-10.69628600	-9.30287500	-0.72302500	H	12.66696300	-6.49654400	-1.09509500
H	-10.31253500	-11.24657700	0.76495400	H	14.37698600	-5.37637900	0.31111600
C	-11.41896600	1.66058300	0.22875800	C	2.58756400	-11.18527300	0.39844800
C	-12.00056800	2.60730600	1.08773100	C	1.97396800	-12.00850400	1.35804100
C	-12.27148800	0.69957200	-0.33883800	C	3.72216500	-11.70867600	-0.24477200
C	-13.35830000	2.58862300	1.37743100	C	2.46806900	-13.26859100	1.66970900
H	-11.37165600	3.36310000	1.54650300	H	1.09339100	-11.64414000	1.87778000
C	-13.63499000	0.69206600	-0.07729400	C	4.21316700	-12.97495300	0.04586200
H	-11.85662600	-0.04617600	-1.00895200	H	4.22191200	-11.10927300	-0.99905600
C	-14.17990700	1.63394400	0.78835100	C	3.58986200	-13.75943300	1.01038000
H	-13.77832700	3.32174500	2.05766800	H	1.97600200	-13.87135400	2.42636500
H	-14.27316000	-0.05120100	-0.54304100	H	5.08479000	-13.35140500	-0.47996400
H	-15.24310600	1.62397600	1.00358500	H	3.97452400	-14.74641700	1.24528400
B	-3.41687400	9.32415000	0.14647600	B	-7.47704400	-6.54065500	0.29290900
H	6.59721300	2.49191400	1.61110600	H	-4.73189300	5.10594900	1.53211100
C	10.21232600	0.55146900	-0.92929200	C	-4.19290200	9.31281100	-0.77197100
C	9.29538500	-0.24470500	-0.23142700	C	-3.11896900	8.68516200	-0.12654900
C	8.12217000	0.32929200	0.27319000	C	-3.23469700	7.35660000	0.30752700
C	7.88922000	1.67622300	0.07148500	C	-4.41337900	6.67997300	0.07739500
C	8.81651400	2.47447000	-0.61697900	C	-5.49179900	7.30891300	-0.57297600
C	9.98386100	1.90590100	-1.11588800	C	-5.37634100	8.63230100	-0.99536500
H	11.11180600	0.09443700	-1.32434800	H	-4.08345900	10.33739800	-1.10594100
H	7.41604000	-0.28639100	0.81927800	H	-2.41011600	6.88503300	0.83028700
H	10.70921600	2.50388500	-1.65704100	H	-6.19545900	9.12918100	-1.50252800
C	6.72254300	2.51444200	0.52301900	C	-4.78917300	5.27136700	0.45084200
H	5.77787800	2.16251400	0.09369500	H	-4.12460800	4.53469400	-0.01327600
C	7.08560900	3.88795500	0.02372500	C	-6.19920800	5.15753600	-0.06359300
C	6.39665200	5.07648200	0.17093100	C	-7.06066900	4.08256200	0.00553000
C	6.92767900	6.24806000	-0.38299800	C	-8.34823700	4.19881400	-0.54220300
C	8.14776700	6.19842400	-1.06903400	C	-8.73473700	5.40954900	-1.14536300
C	8.84806700	5.00872600	-1.19459400	C	-7.87176300	6.48780200	-1.19979000
C	8.31679600	3.84464200	-0.64940200	C	-6.59306600	6.36721300	-0.66019400
H	5.45613100	5.11441600	0.70910700	H	-6.75954300	3.15705700	0.48183700
H	8.54500200	7.10819400	-1.50301000	H	-9.72264800	5.48910500	-1.58111400
H	9.79339700	4.99564700	-1.72625300	H	-8.19317300	7.40758500	-1.67608600
H	7.96013600	-6.53212300	1.22970900	H	3.27605500	9.97946200	1.03019700
C	3.84443100	-7.11139200	-1.21151500	C	5.19425900	6.04991000	-1.04510100
C	4.17199800	-7.93390200	-0.12596300	C	5.90186600	6.78465700	-0.06259700
C	5.22706000	-7.57243800	0.72100400	C	5.22320400	7.76812100	0.69066900
C	5.93848000	-6.41779900	0.46005500	C	3.88785800	7.98121800	0.46226500
C	5.60345300	-5.59032100	-0.62345500	C	3.18281000	7.23883000	-0.51319600
C	4.54519500	-5.93993000	-1.45611900	C	3.85630100	6.27182600	-1.27009300
H	3.03420900	-7.40348700	-1.86890500	H	5.72802200	5.32068600	-1.64001800

H	5.47829900	-8.20413700	1.56559300	H	5.75239400	8.31967400	1.45763000
H	4.27446400	-5.32079800	-2.30463800	H	3.34035700	5.70880800	-2.03923000
C	7.11055500	-5.84077500	1.20784600	C	2.95374200	8.93969200	1.14825100
H	6.86273500	-5.61906100	2.25187400	H	2.89193200	8.74958300	2.22480000
C	7.42269200	-4.59313000	0.42552000	C	1.64522200	8.67574100	0.45585100
C	8.41359500	-3.65693100	0.64638200	C	0.42214200	9.25864600	0.67168500
C	8.53363400	-2.56669700	-0.22499600	C	-0.68602100	8.83372300	-0.09134100
C	7.63364500	-2.43200600	-1.29025900	C	-0.51529500	7.82358200	-1.06609100
C	6.62707400	-3.36326800	-1.49682500	C	0.71418200	7.24376100	-1.27782000
C	6.52251900	-4.45674000	-0.64329200	C	1.81207400	7.66253800	-0.51611100
H	9.10165500	-3.75780800	1.47819600	H	0.29279400	10.01949100	1.43144700
H	7.73684000	-1.58931200	-1.96342600	H	-1.36313300	7.52629600	-1.66941600
H	5.94549900	-3.23970900	-2.33162600	H	0.82611400	6.48393800	-2.04261100
H	1.72077900	9.10220300	2.05300400	H	-9.11971100	-1.58435400	2.02042700
C	-1.70190700	7.83493000	-1.09087400	C	-6.68736500	-4.44235000	-1.00294700
C	-1.96210300	8.82574300	-0.12454900	C	-7.52435600	-5.00509900	-0.01993800
C	-0.86424700	9.35592000	0.58080900	C	-8.41551700	-4.14528100	0.64916300
C	0.41614000	8.90099900	0.33345100	C	-8.44830600	-2.79578500	0.35172800
C	0.64685200	7.92534200	-0.65140400	C	-7.61313000	-2.26783400	-0.64624500
C	-0.41780200	7.39245500	-1.37267900	C	-6.72971600	-3.09554500	-1.33229000
H	-2.53381500	7.41605200	-1.64699000	H	-5.99727600	-5.08975600	-1.53305800
H	-1.03313500	10.11859200	1.33473000	H	-9.07436000	-4.54955700	1.41155000
H	-0.25198300	6.64161800	-2.13809500	H	-6.08281100	-2.70048600	-2.10864600
C	1.71695300	9.30335300	0.97590500	C	-9.29798400	-1.70369900	0.94585100
H	1.92080400	10.37347400	0.85874900	H	-10.36878500	-1.90385500	0.82864900
C	2.72456000	8.45961500	0.24293700	C	-8.86963000	-0.48894100	0.16804000
C	4.09395500	8.40049500	0.41339300	C	-9.31250100	0.81538200	0.27604400
C	4.84942200	7.53970900	-0.39381400	C	-8.77399100	1.79075300	-0.57058600
C	4.20163200	6.74626500	-1.35274100	C	-7.79350400	1.43887400	-1.50669900
C	2.82581300	6.79330500	-1.50588500	C	-7.33610800	0.13377400	-1.59851800
C	2.07872400	7.65575700	-0.70918400	C	-7.87742400	-0.83780500	-0.76224500
H	4.58895900	9.00969000	1.16091500	H	-10.07050000	1.09162300	1.00042600
H	4.79291600	6.09308700	-1.98286900	H	-7.39831600	2.19959400	-2.16948500
H	2.34834800	6.17204100	-2.25598900	H	-6.57903500	-0.12097200	-2.33204500
H	-10.03779300	-3.76393600	0.62131000	H	7.26540700	-8.07724800	0.09438200
C	-8.04077800	0.23927500	-1.20610200	C	2.60559000	-7.46294500	-1.01049600
C	-9.12647600	0.32439000	-0.31285400	C	2.99598000	-8.58532700	-0.23615900
C	-9.50707700	-0.84475900	0.37162800	C	4.32696100	-8.57722900	0.25861800
C	-8.81677800	-2.02721400	0.18019400	C	5.17101800	-7.51791000	0.02012500
C	-7.74446700	-2.08274800	-0.72456500	C	4.74179400	-6.40704700	-0.74115900
C	-7.35806100	-0.94684000	-1.42988000	C	3.44441000	-6.39789900	-1.27059100
H	-7.73835600	1.12873800	-1.74828000	H	1.60762400	-7.45185300	-1.43576900
H	-10.34105600	-0.80896700	1.06555000	H	4.66987800	-9.41415400	0.85936400
H	-6.53637600	-0.98275600	-2.13731500	H	3.10129400	-5.56725800	-1.87956300
C	-9.03110700	-3.37582600	0.81244600	C	6.59257400	-7.29954800	0.47373700
H	-8.91393400	-3.33920000	1.90136700	H	6.68276300	-7.31865000	1.56575400
C	-7.96767900	-4.21953200	0.16300600	C	6.92378700	-5.94241700	-0.09452000
C	-7.68293800	-5.56189100	0.33165800	C	8.08095500	-5.20695300	0.00888400
C	-6.61643400	-6.15947200	-0.36486600	C	8.18344000	-3.91022000	-0.56301500
C	-5.86975300	-5.35117400	-1.24379800	C	7.04151500	-3.43838400	-1.26159500
C	-6.16478300	-4.01143900	-1.44750500	C	5.88391800	-4.17652000	-1.40017400
C	-7.21579300	-3.44349200	-0.73364900	C	5.80774700	-5.44299500	-0.80469400
H	-8.27597800	-6.16130400	1.01534100	H	8.92451200	-5.60524300	0.56480800
H	-5.04692700	-5.79689500	-1.79235900	H	7.09193500	-2.46064900	-1.72970100
H	-5.58245000	-3.42073000	-2.14669900	H	5.04432100	-3.77848800	-1.96164200
H	-1.14503900	-6.86769400	1.56349900	H	7.01730900	1.40784300	1.60123500
C	-4.48828300	-9.45221200	-0.75112200	C	10.39833100	-0.70816800	-1.09084500
C	-4.79935400	-8.16927500	-0.26067500	C	9.37300200	-1.48634300	-0.50916100
C	-3.73077100	-7.34991000	0.15335600	C	8.27050900	-0.79459900	0.03189100
C	-2.42884700	-7.80719700	0.09037100	C	8.20694400	0.58397500	-0.01434300
C	-2.14905100	-9.08662700	-0.41894300	C	9.23483500	1.32785300	-0.61894500
C	-3.18365100	-9.91224700	-0.84955100	C	10.33993300	0.67373000	-1.16359700
H	-5.29685500	-10.09736700	-1.07768400	H	11.26060900	-1.21722500	-1.50862400
H	-3.93955100	-6.35715700	0.53996300	H	7.47226500	-1.35924000	0.50310600
H	-2.97911100	-10.90013200	-1.24862600	H	11.14234000	1.23225700	-1.63502500
C	-1.15028700	-7.12381900	0.49818000	C	7.14292400	1.50677700	0.51723900
H	-0.99171100	-6.18705200	-0.04755500	H	6.16213000	1.30783500	0.07118900
C	-0.10211600	-8.15044000	0.16191500	C	7.66381800	2.86535600	0.13741300
C	1.26716900	-8.08283300	0.33414000	C	7.11051400	4.10565300	0.37181200
C	2.06071000	-9.17058700	-0.05319800	C	7.78690900	5.24685700	-0.08114900
C	1.45295100	-10.30876500	-0.60430100	C	9.01247700	5.12631100	-0.74567200
C	0.07858400	-10.37992100	-0.75786900	C	9.57165500	3.87918800	-0.96881700
C	-0.70715300	-9.29677500	-0.37657700	C	8.89801800	2.73908200	-0.52996200
H	1.73274700	-7.20458500	0.76668000	H	6.17380600	4.20998700	0.90823600
H	2.07448600	-11.14001200	-0.91414700	H	9.51779500	6.02021400	-1.09158600
H	-0.36840700	-11.27038700	-1.18653300	H	10.51951400	3.79934400	-1.48877000
H	-5.45670900	4.44628100	1.57828900	H	-2.17419600	-6.59720500	1.69935900
C	-5.87870300	8.78890800	-0.45906500	C	-6.09315400	-8.67624800	-0.16774700
C	-4.62447300	8.34009800	-0.00207300	C	-6.13148200	-7.31617300	0.20442000
C	-4.49237200	6.97771100	0.32322900	C	-4.90020800	-6.68325500	0.47872900
C	-5.57125700	6.12064200	0.20925800	C	-3.71301800	-7.38035000	0.39207800
C	-6.80432000	6.59378100	-0.26723900	C	-3.70493900	-8.72985700	-0.00886800
C	-6.95968800	7.93350400	-0.61106400	C	-4.90579400	-9.37946600	-0.29322900
H	-5.99850400	9.83634500	-0.71445200	H	-7.02686500	-9.18355200	-0.38669100
H	-3.53747900	6.60497100	0.68048700	H	-4.89436900	-5.63897600	0.77615900
H	-7.90753600	8.30691000	-0.98395200	H	-4.91431400	-10.41823700	-0.60650200
C	-5.66569800	4.65194500	0.52240100	C	-2.30587900	-6.91626600	0.65902900
H	-4.94815500	4.06047500	-0.05713500	H	-2.02943700	-6.05934400	0.03453000
C	-7.08918100	4.32645700	0.15932100	C	-1.48530300	-8.13749700	0.33795800
C	-7.75814600	3.11821900	0.21684400	C	-0.11562200	-8.30744700	0.37436100
C	-9.11146300	3.03082100	-0.15953300	C	0.47316100	-9.54195200	0.02736900
C	-9.74664200	4.20660900	-0.60486000	C	-0.40333600	-10.58773100	-0.33891700
C	-9.08063300	5.41980200	-0.69241800	C	-1.77859000	-10.43087600	-0.38845500
C	-7.74633700	5.47887300	-0.30086400	C	-2.32775900	-9.19485200	-0.04791200
H	-7.24352500	2.22928900	0.56776700	H	0.52204400	-7.48659400	0.68686300
H	-10.78831100	4.15778800	-0.90318100	H	0.02165900	-11.54941200	-0.60685400
H	-9.59471400	6.30350700	-1.05523300	H	-2.41375000	-11.25721700	-0.69152600
N	6.24646600	7.47628800	-0.24697100	N	-9.24011800	3.12326000	-0.49405600
N	9.55731900	-1.61712800	-0.03520700	N	-1.93008100	9.40822900	0.10499500
N	3.45742300	-9.12748800	0.10793000	N	7.24094500	6.53229100	0.15629300

Structure S68. The coordinates of both the optimized structures of B2N4L$_3$ at S$_0$ and S$_1$ in toluene.

S_0-optimized structure				S_1-optimized structure			
B	-4.19803000	8.96626800	0.05315000	B	-4.55182300	8.67131400	0.25553200
C	4.41288300	-10.06345100	0.47786900	C	4.77972400	-9.68958400	0.85226800
C	5.42093200	-10.26385800	1.42529000	C	5.73907100	-9.73515200	1.86556000
C	3.78226100	-11.17666400	-0.08524900	C	4.25875800	-10.88263200	0.34622000
C	5.79162100	-11.54935700	1.79207300	C	6.16897200	-10.95744500	2.36128400
H	5.91079400	-9.40634000	1.87126100	H	6.14280300	-8.81030100	2.26013200
C	4.14820300	-12.45787900	0.30122000	C	4.68467300	-12.09944700	0.85830800
H	3.00624900	-11.03147200	-0.82742700	H	3.52641900	-10.84950200	-0.45190900
C	5.15648700	-12.65479700	1.23778000	C	5.64250000	-12.14461100	1.86517200
H	6.57584300	-11.68497000	2.52891200	H	6.91368900	-10.97937700	3.14898400
H	3.64855100	-13.30954700	-0.14751300	H	4.27554900	-13.01889700	0.45467500
H	5.44409900	-13.65768900	1.53166500	H	5.97821100	-13.09745900	2.25758900
C	-6.19317500	-9.11323600	0.42144500	C	-5.79511500	-9.26086600	0.54015500
C	-5.75936300	-10.31578900	-0.14418800	C	-5.37797300	-10.40740400	-0.13696500
C	-7.23520900	-9.14105200	1.35292900	C	-6.71710300	-9.36340500	1.58259300
C	-6.35038300	-11.51577600	0.22383300	C	-5.88159400	-11.64601000	0.23102400
H	-4.95862500	-10.30369200	-0.87406100	H	-4.66873800	-10.31975100	-0.95152700
C	-7.83163200	-10.34427600	1.70069800	C	-7.22112000	-10.60617000	1.93679400
H	-7.57394900	-8.21466000	1.80146400	H	-7.02608400	-8.47107700	2.11426100
C	-7.39208600	-11.53988200	1.14399100	C	-6.80513300	-11.75054300	1.26557700
H	-6.00079000	-12.43837500	-0.22664400	H	-5.55867500	-12.53270000	-0.30226400
H	-8.63888500	-10.34559300	2.42504400	H	-7.93349400	-10.68038700	2.75036400
H	-7.85603800	-12.47878000	1.42339600	H	-7.19846500	-12.72004400	1.54797600
C	11.12692400	-0.88511600	0.02437500	C	11.30125200	-0.44910800	-0.57866000
C	11.95903300	0.00193500	-0.66332500	C	11.97555900	0.49917300	-1.35235100
C	11.68060000	-1.71957700	0.99841300	C	12.03044800	-1.24206700	0.31185600
C	13.31432600	0.05716900	-0.37173000	C	13.34917300	0.65040100	-1.23179200
H	11.53723800	0.64515900	-1.42667100	H	11.41634500	1.11558600	-2.04615100
C	13.04022600	-1.66984800	1.27047700	C	13.40620700	-1.09497100	0.41256200
H	11.03962300	-2.40626900	1.53868600	H	11.51312300	-1.97011700	0.92549900
C	13.86491300	-0.77966900	0.59232000	C	14.07451500	-0.14715500	-0.35405000
H	13.94636400	0.75087800	-0.91531100	H	13.85631300	1.39153100	-1.83970900
H	13.45390400	-2.32487700	2.02939100	H	13.95651300	-1.71736700	1.10971300
H	14.92557600	-0.73910800	0.81195700	H	15.14845000	-0.02954500	-0.26651100
C	6.85641400	9.12641900	0.43425900	C	6.53776000	9.23602000	0.66231200
C	8.16832800	9.06980900	-0.06389900	C	7.80095500	9.28549700	0.04820200
C	6.55047300	10.17384800	1.31831300	C	6.29940000	10.16892900	1.68587700
C	9.12163600	10.01561800	0.28893400	C	8.76058800	10.21987700	0.41510700
H	8.43908300	8.27550300	-0.75169100	H	8.02883200	8.57842300	-0.74334000
C	7.50571000	11.10698100	1.69856800	C	7.25976900	11.09462200	2.07398700
H	5.54705800	10.24794300	1.72436900	H	5.34133000	10.15701300	2.19548600
C	8.79291800	11.03296800	1.17797500	C	8.49408000	11.12798200	1.43442600
H	10.12308100	9.95740600	-0.12387100	H	9.72002200	10.23910700	-0.09195800
H	7.24625000	11.89631400	2.39589100	H	7.04498900	11.79294000	2.87652900
H	9.53850000	11.76707200	1.46436400	H	9.24393300	11.85441000	1.72989800
C	-11.10177100	1.15744000	0.02807100	C	-11.26703700	0.74163200	-0.51333100
C	-11.80396900	0.43887800	0.99872300	C	-12.08168200	0.13790300	0.44669200
C	-11.75160900	2.18610200	-0.65872500	C	-11.80756500	1.68926700	-1.38394700
C	-13.13085600	0.74151500	1.26856000	C	-13.42663100	0.46935500	0.51908500
H	-11.30381500	-0.35680800	1.53818300	H	-11.65447600	-0.57886100	1.13815400
C	-13.07341400	2.49294900	-0.36974000	C	-13.15127800	2.02024000	-1.29681000
H	-11.21520900	2.74083500	-1.41961900	H	-11.17128700	2.15760500	-2.12530900
C	-13.77292800	1.77172600	0.59116900	C	-13.96703700	1.41077700	-0.34965700
H	-13.66133500	0.17408600	2.02538300	H	-14.05115900	-0.00004300	1.27064400
H	-13.56337400	3.29383800	-0.91259500	H	-13.56384400	2.75414200	-1.97955600
H	-14.80778000	2.00945000	0.80904100	H	-15.01685000	1.67212400	-0.28552100
C	-5.06369700	10.20400600	0.47021100	C	-5.52005700	9.83942400	0.68507100
C	-4.57084300	11.18014100	1.35143200	C	-5.18107700	10.75913500	1.69342300
C	-6.36629300	10.38399500	-0.02300800	C	-6.78079600	10.00893100	0.08519800
C	-5.34096400	12.27053500	1.73323500	C	-6.03925900	11.78165900	2.07732000
H	-3.56894500	11.07278600	1.75380400	H	-4.22377800	10.65888300	2.19482900
C	-7.13236300	11.48642400	0.33140000	C	-7.63986700	11.03690200	0.45055300
H	-6.77823700	9.65094600	-0.70873400	H	-7.08654200	9.32024400	-0.69649100
C	-6.62234500	12.42914300	1.21716100	C	-7.27310200	11.92938900	1.45269000
H	-4.94096000	13.00089500	2.42839600	H	-5.74516700	12.46599500	2.86690000
H	-8.12934700	11.60912300	-0.07807700	H	-8.59884600	11.14378000	-0.04680900
H	-7.22223400	13.28616100	1.50443300	H	-7.94333100	12.73084800	1.74550800
B	5.78066000	8.06468900	0.02116800	B	5.45601000	8.17330100	0.23307700
H	5.81269700	-3.98250600	1.66739500	H	6.08777700	-3.55211100	1.55290500
C	6.21846100	-8.11403700	-0.79406000	C	6.49894200	-7.82973700	-0.64573200
C	5.04315600	-7.78197200	-0.10848900	C	5.33351500	-7.46498400	0.03820300
C	4.86636800	-6.47917700	0.37338000	C	5.14473200	-6.13727800	0.44189300
C	5.85684300	-5.54009900	0.15852200	C	6.11540400	-5.20160000	0.14628300
C	7.03846200	-5.87878100	-0.51989000	C	7.28806800	-5.56901100	-0.53691400
C	7.21782900	-7.17423700	-0.99345100	C	7.47808200	-6.89117100	-0.92944100
H	6.34048400	-9.12250700	-1.17107500	H	6.62725900	-8.85977400	-0.95656200
H	3.95917200	-6.22464200	0.91015200	H	4.24721000	-5.86281800	0.98525300
H	8.12014500	-7.45417100	-1.52641400	H	8.37372400	-7.19198500	-1.46170300
C	5.90123600	-4.09550000	0.58128600	C	6.14917600	-3.73293400	0.47420000
H	5.08206100	-3.51664300	0.14040400	H	5.31071700	-3.19384300	0.01973400
C	7.24370600	-3.64040900	0.07304900	C	7.47119800	-3.29518300	-0.09771800
C	7.84319800	-2.40127400	0.19113500	C	8.04805000	-2.04157700	-0.08084800
C	9.11090700	-2.19840000	-0.36849300	C	9.30257600	-1.85447500	-0.68026200
C	9.75306700	-3.25353500	-1.02887500	C	9.94752600	-2.94777200	-1.28222000
C	9.15910400	-4.50238700	-1.12470800	C	9.37219300	-4.20667900	-1.28063400
C	7.89664100	-4.70045300	-0.57558700	C	8.12519700	-4.38791300	-0.68907000
H	7.34796800	-1.58799000	0.70974200	H	7.54747300	-1.20413400	0.39084000
H	10.72927500	-3.08405900	-1.46724800	H	10.90795100	-2.79603500	-1.75919200
H	9.67748100	-5.30546200	-1.63738400	H	9.89136000	-5.03267100	-1.75473500
H	-0.94587400	-10.17700200	1.26963900	H	-0.57108300	-10.12184700	1.40747800
C	-3.68000500	-7.07605900	-1.21328800	C	-3.38721300	-7.05581400	-1.04282300
C	-4.20084100	-7.81755700	-0.14489900	C	-3.89144700	-7.84994400	0.00061600
C	-3.32737600	-8.49377900	0.71661300	C	-3.00766300	-8.52912500	0.85425700
C	-1.96740700	-8.43526300	0.48326300	C	-1.65204800	-8.40547000	0.64818500
C	-1.44762800	-7.68964400	-0.58690400	C	-1.14602800	-7.60658300	-0.39625800
C	-2.31253500	-7.00093100	-1.43100900	C	-2.02528700	-6.92788100	-1.24016600
H	-4.36219600	-6.56209900	-1.87990300	H	-4.07943700	-6.55992000	-1.71217400
H	-3.72720300	-9.06017500	1.55024200	H	-3.39808000	-9.13386300	1.66437900

H	-1.93407700	-6.42356400	-2.26771800
C	-0.84765000	-9.08606500	1.25094200
H	-0.82209000	-8.75795000	2.29605800
C	0.37598000	-8.64598100	0.49218600
C	1.70171700	-8.94634300	0.73599200
C	2.68880500	-8.43761700	-0.11820600
C	2.31671500	-7.61543900	-1.18992000
C	0.98633900	-7.29758900	-1.41804900
C	0.00577100	-7.82032500	-0.58130800
H	1.98690600	-9.57445500	1.57247100
H	3.08457700	-7.23159100	-1.85089700
H	0.72357100	-6.66242300	-2.25723200
H	8.54252700	3.74857900	1.95348900
C	5.54112800	5.79895200	-1.20725300
C	6.20768900	6.58708300	-0.24933900
C	7.27252600	5.99593800	0.45794100
C	7.62851500	4.68216700	0.22352900
C	6.95490500	3.92531600	-0.75022600
C	5.90959600	4.48903000	-1.47637800
H	4.72076600	6.23745400	-1.76547400
H	7.80416500	6.57754500	1.20473700
H	5.38542600	3.91683600	-2.23460100
C	8.70083500	3.84923000	0.87396200
H	9.69709700	4.28669900	0.74519700
C	8.57432700	2.52969600	0.16184300
C	9.29865200	1.36906100	0.35179700
C	9.01211600	0.24507800	-0.43405700
C	7.99047300	0.31516500	-1.39322500
C	7.25299500	1.47483400	-1.56633000
C	7.54445200	2.59217700	-0.78962100
H	10.08183000	1.31718000	1.09942600
H	7.78297300	-0.55328700	-2.00652800
H	6.46889000	1.50483200	-2.31525700
H	-8.73269200	5.96448800	0.78625900
C	-4.37643700	6.69645600	-1.17797400
C	-4.88533800	7.58993000	-0.21581600
C	-6.03765700	7.20027500	0.49405900
C	-6.62849300	5.97459100	0.25696800
C	-6.10810600	5.11139000	-0.72224800
C	-4.97893400	5.47687300	-1.44973900
H	-3.49161800	6.97969100	-1.73811900
H	-6.45205900	7.86675300	1.24416000
H	-4.57063600	4.82091600	-2.21137500
C	-7.83379300	5.34997500	0.90795900
H	-7.69276000	5.21521500	1.98617700
C	-7.95555200	4.03412000	0.18820500
C	-8.88299600	3.02728500	0.37299400
C	-8.81249800	1.87538600	-0.42136400
C	-7.79763100	1.76057300	-1.38332800
C	-6.85736600	2.76375100	-1.55125400
C	-6.93404800	3.91035900	-0.76632500
H	-9.66069800	3.11699600	1.12278500
H	-7.75766600	0.87335000	-2.00352300
H	-6.08327300	2.65284000	-2.30299500
H	-6.45442600	-2.88072700	1.65920100
C	-10.19070700	-1.42251300	-1.03129500
C	-9.36433900	-0.50718600	-0.36711400
C	-8.15609100	-0.94368500	0.19069100
C	-7.79629400	-2.27181500	0.06700300
C	-8.63376800	-3.18983400	-0.58619300
C	-9.83785900	-2.75922100	-1.13319500
H	-11.11871100	-1.07347100	-1.46810400
H	-7.51871500	-0.23820000	0.71188000
H	-10.49586600	-3.45013400	-1.64910200
C	-6.56137200	-2.96973100	0.57251200
H	-5.64897300	-2.54981400	0.13469400
C	-6.78426200	-4.39545100	0.14209900
C	-5.98409600	-5.50223600	0.35111600
C	-6.39717300	-6.74735800	-0.13873400
C	-7.61305800	-6.85288000	-0.82600500
C	-8.42228900	-5.74396900	-1.01865600
C	-8.00777600	-4.50653500	-0.53744100
H	-5.04592400	-5.42229800	0.88896400
H	-7.91873200	-7.81909000	-1.20933500
H	-9.36064100	-5.84994300	-1.55232500
H	0.64839100	6.87799700	1.50022000
C	3.91485800	9.77705900	-0.53172200
C	4.27898200	8.48494600	-0.10561500
C	3.24681200	7.58504900	0.21793500
C	1.92296600	7.97459100	0.13424200
C	1.59099400	9.26593800	-0.30566800
C	2.59054500	10.17135400	-0.64993100
H	4.69726200	10.48343500	-0.78764000
H	3.49814500	6.58330000	0.55195100
H	2.34346900	11.16930800	-0.99626200
C	0.67380200	7.19856500	0.45269100
H	0.58970700	6.28894500	-0.15246200
C	-0.41820900	8.18602300	0.14210900
C	-1.78985100	8.03998900	0.23480600
C	-2.64609900	9.11057100	-0.08232700
C	-2.05916000	10.31673200	-0.51173900
C	-0.68646100	10.46728100	-0.63915400
C	0.13688200	9.39724300	-0.30083900
H	-2.21451600	7.09927200	0.57097100
H	-2.70394800	11.15201800	-0.76308500
H	-0.26684400	11.40491000	-0.98795800
N	9.74579800	-0.94262200	-0.26468900
N	4.04432400	-8.75741100	0.09544400
N	-5.59383300	-7.89017000	0.05801000
N	-9.75483700	0.84417900	-0.25817700

H	-1.65576700	-6.32278400	-2.06017300
C	-0.51706900	-9.02804000	1.41448300
H	-0.51804300	-8.72127200	2.46600900
C	0.69472900	-8.51957700	0.68154700
C	2.02519500	-8.77316800	0.93299200
C	3.00345200	-8.18904200	0.11028300
C	2.60489500	-7.34709400	-0.94565300
C	1.26965400	-7.08801300	-1.18589800
C	0.30071700	-7.67777100	-0.37457300
H	2.32460800	-9.40951300	1.75713000
H	3.36133500	-6.91203400	-1.58648500
H	0.98944400	-6.44558500	-2.01331500
H	8.45680100	3.87257900	1.84959100
C	5.34056100	5.97675300	-1.16489300
C	5.95252100	6.72928500	-0.14141800
C	7.04829300	6.14160300	0.52082600
C	7.47983800	4.87150800	0.18907300
C	6.84949200	4.14926100	-0.83738800
C	5.77614600	4.71008700	-1.52474700
H	4.50615200	6.41468200	-1.70237500
H	7.54995400	6.69534100	1.30880300
H	5.28563800	4.17037000	-2.32869000
C	8.60139500	4.05742900	0.77923000
H	9.57089400	4.55831600	0.67906900
C	8.54726500	2.78483200	-0.02219000
C	9.33258200	1.65366700	0.08558300
C	9.10039700	0.57620500	-0.77694600
C	8.07961400	0.65479300	-1.73124700
C	7.27945000	1.78339000	-1.82483500
C	7.51199300	2.85727700	-0.96987800
H	10.12267200	1.58779000	0.82559900
H	7.92048800	-0.18152200	-2.40203400
H	6.49287000	1.82578600	-2.57049200
H	-9.01908300	5.50643300	0.74363000
C	-4.66193400	6.45335500	-1.11544800
C	-5.19271300	7.28162400	-0.10056200
C	-6.34622600	6.81616700	0.56491400
C	-6.90729200	5.59572100	0.24580800
C	-6.35586400	4.79882500	-0.77301700
C	-5.22530700	5.23707600	-1.46283800
H	-3.78692300	6.79673600	-1.65684900
H	-6.78679600	7.42672300	1.34725300
H	-4.79239100	4.64155300	-2.26048900
C	-8.10619800	4.90907200	0.84597600
H	-7.97832100	4.71817300	1.91734800
C	-8.18768100	3.63116100	0.05618900
C	-9.08749500	2.59292900	0.16946900
C	-8.96321200	1.49033400	-0.68599000
C	-7.94548700	1.45296500	-1.64614100
C	-7.03430100	2.49130600	-1.74910800
C	-7.14889100	3.58803100	-0.89517100
H	-9.88038700	2.61371400	0.90903200
H	-7.88282100	0.60448100	-2.31773100
H	-6.25326200	2.45045200	-2.50008300
H	-6.25319200	-2.90798800	1.40749200
C	-10.30410000	-1.92738700	-1.03174300
C	-9.45905000	-0.89256200	-0.56722500
C	-8.16851300	-1.21447000	-0.09605600
C	-7.76019900	-2.52550900	-0.10140400
C	-8.60883400	-3.55508600	-0.56191600
C	-9.88971900	-3.24012900	-1.02684100
H	-11.28220700	-1.67427800	-1.41907900
H	-7.52133400	-0.43021400	0.27595800
H	-10.55138600	-4.01259800	-1.40220400
C	-6.44772000	-3.10738300	0.34839100
H	-5.60359900	-2.68894500	-0.20947100
C	-6.62851100	-4.57623900	0.07839200
C	-5.74895900	-5.61023000	0.28977000
C	-6.14733900	-6.92419600	-0.02468800
C	-7.43887200	-7.15821000	-0.54074000
C	-8.31640300	-6.11729400	-0.75207400
C	-7.91792100	-4.81187800	-0.44758100
H	-4.76711300	-5.43207100	0.71166200
H	-7.72916600	-8.16968200	-0.79442100
H	-9.29791400	-6.31690700	-1.16631200
H	0.35615800	6.46550400	1.28088100
C	3.52848100	9.88610600	0.07078200
C	3.96008400	8.53945300	0.19100100
C	2.93730900	7.55546600	0.27329800
C	1.60855300	7.90283200	0.24494500
C	1.21342600	9.25588900	0.11648500
C	2.19843300	10.25043600	0.02326300
H	4.28157500	10.66292300	-0.01072100
H	3.21821100	6.51272100	0.38654800
H	1.92084000	11.29400000	-0.08718100
C	0.38365800	7.02802800	0.34055200
H	0.34697400	6.28606100	-0.46542200
C	-0.74851600	8.02025500	0.24554000
C	-2.10477700	7.80584400	0.27800200
C	-3.02883800	8.88544500	0.19870600
C	-2.46236300	10.18382800	0.07666200
C	-1.10390500	10.41425500	0.02695900
C	-0.22093500	9.32722300	0.11758000
H	-2.48577300	6.79581800	0.39598100
H	-3.13431700	11.03179400	-0.00484000
H	-0.72506900	11.42551000	-0.08482400
N	9.90127200	-0.58718700	-0.68740600
N	4.35693900	-8.44405500	0.33183600
N	-5.27740800	-7.99258500	0.17257700
N	-9.88922800	0.42176100	-0.58970900

Structure S69. The coordinates of both the optimized structures of $B1N5L_3$ at S_0 and S_1 in toluene.

S_0-optimized structure				S_1-optimized structure			
C	9.53741500	-5.60292700	0.44675500	C	8.30742700	-7.14082500	0.99049500
C	10.47809600	-5.18405300	1.39233900	C	9.23131700	-6.84862600	1.99806000
C	9.66899100	-6.87368300	-0.12101200	C	8.26433000	-8.43668900	0.46754200
C	11.52797400	-6.01640000	1.75200800	C	10.09298900	-7.83047800	2.46470100
H	10.38064800	-4.20330800	1.84262100	H	9.26916400	-5.84772100	2.41143400
C	10.71255700	-7.70523800	0.25861600	C	9.11865800	-9.41597700	0.95325700
H	8.95068400	-7.20400400	-0.86190100	H	7.56062400	-8.67084300	-0.32239200
C	11.65130300	-7.28326100	1.19308300	C	10.04065300	-9.12117100	1.95104200
H	12.24770200	-5.67344700	2.48730300	H	10.80265200	-7.58434500	3.24701600
H	10.79883100	-8.68721400	-0.19375600	H	9.07127900	-10.41531000	0.53433900
H	12.46923500	-7.93332700	1.48158900	H	10.71094400	-9.88759800	2.32274800
C	0.32468000	-10.95564400	0.36571500	C	-1.61319400	-10.87382100	-0.11928900
C	1.35959900	-11.70058300	-0.20685900	C	-0.69213500	-11.71756300	-0.74568000
C	-0.49580500	-11.56039000	1.32217000	C	-2.54700500	-11.42409800	0.76303400
C	1.57118800	-13.01628700	0.17895900	C	-0.70499200	-13.08062800	-0.48756700
H	1.99478900	-11.24245700	-0.95573300	H	0.03144700	-11.29887200	-1.43507200
C	-0.28717400	-12.88213500	1.68826100	C	-2.56303200	-12.79078200	1.00096300
H	-1.29646700	-10.98837100	1.77593400	H	-3.25743400	-10.77495200	1.26115900
C	0.74871300	-13.61809400	1.12448800	C	-1.64145700	-13.62764500	0.38203900
H	2.37854700	-13.57894000	-0.27700200	H	0.01667200	-13.72090100	-0.98290500
H	-0.93389600	-13.33442900	2.43221300	H	-3.29411200	-13.20071400	1.68921100
H	0.91276700	-14.64854600	1.41796500	H	-1.65214600	-14.69395500	0.57618300
C	9.51844100	5.79359200	0.07756800	C	10.41065600	4.05996200	-0.11218200
C	9.65710500	6.99945300	-0.61441100	C	10.68679600	5.16544500	-0.92125500
C	10.45950100	5.45834000	1.05410100	C	11.35052600	3.66848700	0.84528400
C	10.71086200	7.85415000	-0.32446000	C	11.87486500	5.86510200	-0.76736400
H	8.93588200	7.26075600	-1.37987000	H	9.96474300	5.47173900	-1.66885700
C	11.51987700	6.31125100	1.32478000	C	12.54327300	4.36399700	0.98006600
H	10.35440700	4.52667400	1.59746000	H	11.13882600	2.81762700	1.48212900
C	11.65062200	7.51533000	0.64243300	C	12.81251700	5.46823100	0.17932600
H	10.80433900	8.78607300	-0.87121700	H	12.07268500	6.72063300	-1.40387300
H	12.24145700	6.03592800	2.08610100	H	13.26074400	4.04666600	1.72895600
H	12.47653700	8.18229900	0.86131200	H	13.74204700	6.01418600	0.29271500
C	0.12544100	11.28877600	0.41378800	C	2.12087700	11.08827400	0.00131000
C	1.22249700	12.00764900	-0.08811900	C	3.31433100	11.52830700	-0.60244100
C	-0.74798300	11.97120900	1.27609500	C	1.45442900	12.02786300	0.81097400
C	1.43065600	13.34056400	0.24030900	C	3.80360600	12.81500200	-0.42351100
H	1.91633900	11.51301200	-0.75986700	H	3.86381600	10.84032900	-1.23748300
C	-0.53127300	13.29565700	1.63216700	C	1.94059400	13.31392200	1.00492700
H	-1.60563100	11.44655700	1.68402900	H	0.53771400	11.73157600	1.31114500
C	0.55638600	13.98544800	1.10823400	C	3.11884000	13.71743200	0.38468900
H	2.27722600	13.87642800	-0.17552200	H	4.72205100	13.11768000	-0.91697800
H	-1.21334800	13.79322500	2.31328700	H	1.40115400	14.00464400	1.64570500
H	0.72223300	15.02372200	1.37541600	H	3.50014800	14.72265600	0.53099700
C	-9.61954500	-5.44607300	0.17157600	C	-10.37225900	-3.70397800	0.16346300
C	-9.73224800	-6.46928600	1.11731900	C	-10.55147300	-4.65355200	1.17060000
C	-10.78059600	-4.94401100	-0.42353900	C	-11.48727900	-3.11120200	-0.43253700
C	-10.97816600	-6.98110700	1.44955700	C	-11.83227000	-5.00272300	1.57312500
H	-8.83867600	-6.86011100	1.58936400	H	-9.68565900	-5.11133500	1.63399300
C	-12.02317100	-5.45029900	-0.07115000	C	-12.76347400	-3.45794900	-0.01406800
H	-10.70219600	-4.15667900	-1.16389900	H	-11.34718500	-2.38671900	-1.22626900
C	-12.13203600	-6.47404500	0.86300700	C	-12.94292200	-4.40553600	0.98753600
H	-11.04557000	-7.77500200	2.18545500	H	-11.96085200	-5.73979300	2.35769700
H	-12.91253800	-5.04863500	-0.54446600	H	-13.62268800	-2.99477400	-0.48590400
H	-13.10409500	-6.87212600	1.13035500	H	-13.94171600	-4.67903200	1.30729800
C	-9.85799000	5.18406800	0.37290700	C	-8.72577700	6.80263600	0.85926500
C	-10.01356600	6.11139200	1.40638700	C	-8.53894500	7.62720400	1.97051900
C	-10.99415800	4.68857100	-0.27174600	C	-10.00014700	6.65323300	0.30686900
C	-11.28044400	6.53922100	1.77654700	C	-9.62387400	8.28587800	2.52754900
H	-9.13722800	6.49388700	1.91629200	H	-7.54747200	7.73871300	2.39204000
C	-12.25781400	5.10884500	0.11740400	C	-11.07818900	7.31469900	0.87489500
H	-10.87953400	3.97377700	-1.07813800	H	-10.13338100	6.03354900	-0.57201300
C	-12.41018100	6.03883400	1.13941500	C	-10.89576300	8.13188800	1.98534900
H	-11.38325500	7.25954300	2.58077100	H	-9.47445800	8.91835400	3.39491000
H	-13.12915300	4.71466000	-0.39404500	H	-12.06358400	7.20227800	0.43749000
H	-13.39868400	6.37012800	1.43613700	H	-11.74015900	8.65117900	2.42340900
B	-0.11366100	9.78797300	0.02943100	B	1.57685700	9.62656700	-0.21083100
H	7.09323000	0.12794100	1.70513300	H	7.01318100	-1.00628700	1.93946900
C	9.86043700	-2.94130800	-0.78754800	C	9.17393800	-4.65584800	-0.36482200
C	8.71359800	-3.37482700	-0.11060200	C	7.95202000	-4.83308300	0.29357100
C	7.80220200	-2.43307400	0.38231900	C	7.23039700	-3.71735200	0.73344600
C	8.04803800	-1.08807000	0.18432400	C	7.73167200	-2.45249000	0.49297000
C	9.20258000	-0.65600400	-0.48763700	C	8.95774300	-2.27681400	-0.16939200
C	10.11324200	-1.59043700	-0.96967600	C	9.68412900	-3.38605500	-0.59024700
H	10.55446000	-3.67880900	-1.17286200	H	9.71983300	-5.52842200	-0.70386600
H	6.91818400	-2.76939200	0.91267500	H	6.28809700	-3.85949400	1.25116600
H	11.00787600	-1.27775100	-1.49733100	H	10.63128200	-3.27042100	-1.10590800
C	7.22937100	0.09869700	0.61842300	C	7.14608600	-1.11393400	0.85718200
H	6.22560400	0.08422200	0.17950200	H	6.15926800	-0.96290200	0.40604800
C	8.04129200	1.26421500	0.11895500	C	8.16358500	-0.14577700	0.31379900
C	7.78718600	2.61639400	0.24296800	C	8.15312000	1.23476500	0.34964400
C	8.68576100	3.53548600	-0.31307600	C	9.20347800	1.94495600	-0.24804200
C	9.83064600	3.07137200	-0.97309900	C	10.25433400	1.24041100	-0.85364200
C	10.09524300	1.71430400	-1.07444800	C	10.27373700	-0.14504400	-0.86414300
C	9.19650300	0.80222000	-0.53131600	C	9.22176800	-0.84707500	-0.28510400
H	6.90443600	2.97381200	0.76121000	H	7.34134700	1.77453100	0.82360600
H	10.51522600	3.78953000	-1.40831800	H	11.05703500	1.79379500	-1.32557400
H	10.98970600	1.37890800	-1.58822700	H	11.09711900	-0.66564200	-1.34144800
H	5.22403700	-8.81001600	1.21021400	H	3.47529400	-9.60221400	1.25531800
C	1.21130100	-7.84953100	-1.28536600	C	-0.11644600	-7.88964700	-1.47360100
C	1.20792500	-8.75522900	-0.21650100	C	-0.35170900	-8.81370200	-0.44898100
C	2.30866700	-8.80605300	0.64842300	C	0.65354500	-9.08309200	0.48744700
C	3.38769600	-7.97512000	0.41939900	C	1.87216000	-8.44280700	0.37193500
C	3.38723000	-7.06548600	-0.64999800	C	2.10636000	-7.51515800	-0.65614600
C	2.28698900	-7.00077700	-1.49877900	C	1.10112600	-7.23357800	-1.57560700
H	0.35980900	-7.82180300	-1.95479900	H	-0.89856800	-7.69627500	-2.19832600
H	2.30495400	-9.49916800	1.48216300	H	0.46741600	-9.79395800	1.28489100
H	2.26711300	-6.31072900	-2.33551700	H	1.26390600	-6.52515400	-2.38070000

C	4.67441600	-7.86247700	1.19278800
H	4.50175900	-7.58362400	2.23826200
C	5.42257400	-6.79448100	0.44039600
C	6.67568000	-6.27201300	0.69355700
C	7.19012400	-5.28262700	-0.15418900
C	6.41809100	-4.82608800	-1.23043200
C	5.15126500	-5.33736200	-1.46851400
C	4.64927200	-6.33293400	-0.63682700
H	7.26688300	-6.62021000	1.53310800
H	6.82600100	-4.06624000	-1.88629300
H	4.57463100	-4.96979800	-2.31053800
H	4.67262800	7.96928900	1.99994200
C	1.04206700	7.80418500	-1.16144300
C	1.10723400	8.84381600	-0.21407500
C	2.31318500	9.00865400	0.49438600
C	3.38231400	8.16370200	0.26858100
C	3.29440900	7.14806900	-0.69864000
C	2.11918500	6.96996600	-1.42281200
H	0.12249100	7.66283900	-1.71933500
H	2.39316900	9.80001500	1.23333600
H	2.04026500	6.19169200	-2.17461900
C	4.73975700	8.14003800	0.91968300
H	5.27678400	9.08581900	0.78710100
C	5.42791000	7.00377400	0.21253500
C	6.70337700	6.50875200	0.40325500
C	7.14686600	5.43514700	-0.38013000
C	6.28682500	4.87797000	-1.33852300
C	5.00111700	5.36331000	-1.51132700
C	4.56498000	6.43346700	-0.73605600
H	7.36177300	6.93834400	1.14955500
H	6.64095900	4.05799500	-1.95135200
H	4.35509400	4.91615100	-2.25915000
H	-10.33802400	-0.15207600	1.08589100
C	-7.57775100	2.96199700	-1.34768400
C	-8.35458500	3.37316800	-0.25716800
C	-8.92641400	2.41259400	0.58568200
C	-8.72483300	1.07270300	0.31809500
C	-7.93673900	0.66220100	-0.76878400
C	-7.35754600	1.61616200	-1.59963500
H	-7.14835900	3.71181500	-2.00151100
H	-9.52321500	2.72930400	1.43370100
H	-6.75306500	1.32207100	-2.45093800
C	-9.24294500	-0.12757300	1.06378700
H	-8.91018300	-0.13763400	2.10763300
C	-8.67187400	-1.27808400	0.27923100
C	-8.81496000	-2.63362100	0.50077000
C	-8.19709700	-3.53944500	-0.37030500
C	-7.42857100	-3.05772100	-1.43775700
C	-7.27078700	-1.69566200	-1.64588500
C	-7.90106800	-0.79689200	-0.79110000
H	-9.40145800	-3.00512100	1.33361700
H	-6.95946400	-3.76597000	-2.11042700
H	-6.67434400	-1.34660800	-2.48197800
H	-3.43915300	-5.97640200	1.57139500
C	-7.41108700	-7.02940800	-0.96245100
C	-7.25333200	-5.79381600	-0.32120200
C	-5.99676000	-5.42932300	0.17858100
C	-4.93336200	-6.29932200	0.03184400
C	-5.09948800	-7.54515000	-0.59341800
C	-6.34674500	-7.90793700	-1.09094200
H	-8.38380700	-7.29705900	-1.35747600
H	-5.87451000	-4.47540400	0.67942800
H	-6.49463100	-8.86150100	-1.58635100
C	-3.50780300	-6.13915500	0.49023200
H	-3.02202800	-5.27970700	0.01478300
C	-2.87464900	-7.44022800	0.07263200
C	-1.57818700	-7.87747100	0.26506100
C	-1.20625100	-9.14361500	-0.20401700
C	-2.15129200	-9.94441300	-0.85819400
C	-3.45631600	-9.51079300	-1.03253000
C	-3.82394500	-8.25237100	-0.56788900
H	-0.84885600	-7.25924600	0.77656500
H	-1.85082000	-10.91762000	-1.22766700
H	-4.17168800	-10.15091700	-1.53753200
H	-3.59250400	5.83883600	1.53545100
C	-2.61792600	10.06013800	-0.56795100
C	-1.57055300	9.24049300	-0.10554400
C	-1.88312700	7.91116800	0.23909700
C	-3.17969000	7.44516800	0.13912700
C	-4.20046400	8.28167600	-0.34309800
C	-3.91740600	9.59519000	-0.70665600
H	-2.39745900	11.08715700	-0.83904600
H	-1.09703300	7.25646700	0.60253800
H	-4.69674800	10.24936400	-1.08323300
C	-3.74093000	6.08985800	0.47929000
H	-3.26614600	5.29012000	-0.09999500
C	-5.19877700	6.23757000	0.13560500
C	-6.21772100	5.31272400	0.25934100
C	-7.51687000	5.66846400	-0.12633600
C	-7.76023000	6.95592600	-0.62847000
C	-6.73984300	7.88656200	-0.73115300
C	-5.45000600	7.53029200	-0.34973800
H	-6.02583600	4.32126800	0.65327900
H	-8.76385100	7.21939400	-0.93924300
H	-6.95357400	8.87604600	-1.12067900
N	8.44514900	4.92179800	-0.20909900
N	8.47705400	-4.75386200	0.07127200
N	0.10952500	-9.61518100	-0.01609300
N	-8.35583700	-4.92708800	-0.17676800
N	-8.57212200	4.74595000	-0.01249000

C	3.09700000	-8.57425700	1.23751000
H	2.89451300	-8.29905400	2.27858900
C	4.07028100	-7.63011900	0.58278400
C	5.37107100	-7.33869600	0.94409000
C	6.11157500	-6.43233200	0.17247700
C	5.51070800	-5.82354900	-0.93855300
C	4.19719900	-6.10091200	-1.28295200
C	3.47032200	-7.01388600	-0.52659300
H	5.82648700	-7.80516900	1.81024000
H	6.08917900	-5.13046400	-1.53702900
H	3.75785100	-5.61769700	-2.14912200
H	6.05798800	7.14665700	1.79851700
C	2.33608100	7.36287800	-1.24237900
C	2.60284600	8.45669500	-0.38642200
C	3.85422900	8.45562000	0.27002600
C	4.75632700	7.42754500	0.08481700
C	4.46594800	6.36655200	-0.79180700
C	3.24229800	6.33831200	-1.46067000
H	1.38836800	7.34150800	-1.77054100
H	4.09839300	9.27514600	0.93968400
H	3.00192300	5.53106600	-2.14588900
C	6.11398500	7.22072000	0.70655800
H	6.79948400	8.04701500	0.48699000
C	6.57796300	5.93337800	0.07829300
C	7.75617800	5.24305000	0.27850500
C	7.99259400	4.06219500	-0.43948000
C	7.02654600	3.59913400	-1.34243500
C	5.82972600	4.27657400	-1.51750100
C	5.59675200	5.45243400	-0.80747300
H	8.50126500	5.60397800	0.97882400
H	7.22314000	2.69624600	-1.90831300
H	5.09552500	3.89586500	-2.21946600
H	-10.44242700	1.65679400	0.91679600
C	-6.90100100	4.27282700	-1.09105900
C	-7.69746700	4.79921400	-0.04464000
C	-8.55070200	3.93435900	0.67989000
C	-8.57225000	2.60058700	0.36690600
C	-7.75766800	2.07369900	-0.66420800
C	-6.92903300	2.93223800	-1.39799400
H	-6.27984900	4.94785500	-1.66504600
H	-9.14835200	4.32594600	1.49366900
H	-6.32021500	2.55554500	-2.21186700
C	-9.36333500	1.49297200	1.00774800
H	-9.14834300	1.40269600	2.07768300
C	-8.91318500	0.28026600	0.23989600
C	-9.28863800	-1.03115700	0.40439600
C	-8.70919600	-2.02094000	-0.41320000
C	-7.76000500	-1.64600900	-1.38801700
C	-7.38897900	-0.32894900	-1.55112100
C	-7.96242100	0.65335100	-0.73554500
H	-10.00464000	-1.31422200	1.16636100
H	-7.33450000	-2.40946000	-2.02705500
H	-6.66795700	-0.06514600	-2.31648500
H	-4.40963100	-5.32889200	1.37444400
C	-8.51025800	-5.54507100	-1.16447900
C	-8.12614100	-4.38855000	-0.47553000
C	-6.82405700	-4.27131000	0.02795700
C	-5.93455600	-5.30845900	-0.16712700
C	-6.32468500	-6.47614800	-0.84571100
C	-7.61883600	-6.58974300	-1.34660500
H	-9.51852300	-5.61669600	-1.55450300
H	-6.53596800	-3.38071200	0.57539500
H	-7.93453300	-7.47986300	-1.87940800
C	-4.50491300	-5.43013200	0.28796800
H	-3.87012500	-4.65424900	-0.15417200
C	-4.12050300	-6.80554300	-0.18844700
C	-2.92705900	-7.47915800	-0.01915600
C	-2.79313700	-8.77568700	-0.53491900
C	-3.86985600	-9.36212800	-1.21805200
C	-5.06942500	-8.68851900	-1.37296400
C	-5.20114100	-7.40244700	-0.85733100
H	-2.09977900	-7.02532700	0.51445900
H	-3.75369500	-10.35821600	-1.62733500
H	-5.88738800	-9.16492800	-1.90257800
H	-2.43660100	6.33076700	1.51397900
C	-0.88598100	10.34837500	-0.62819000
C	0.05664400	9.34980000	-0.24730200
C	-0.48472000	8.08178900	0.10619400
C	-1.83802900	7.85253700	0.08656500
C	-2.74458900	8.86284300	-0.31914000
C	-2.24408200	10.12604000	-0.68130600
H	-0.50863400	11.32445000	-0.91446700
H	0.19132000	7.29497200	0.42660000
H	-2.91583700	10.91653500	-1.00180200
C	-2.60177300	6.60933900	0.46708800
H	-2.30935800	5.74161500	-0.13493000
C	-4.03234100	7.00623400	0.21488100
C	-5.17886700	6.27707000	0.39871900
C	-6.42488600	6.86570100	0.08849600
C	-6.47710700	8.18548500	-0.39087000
C	-5.32379500	8.92434500	-0.55912900
C	-4.07900000	8.34461800	-0.25622500
H	-5.14347900	5.26809800	0.79460900
H	-7.43926400	8.61886400	-0.63824400
H	-5.38464000	9.94050200	-0.93165100
N	9.19869000	3.35185900	-0.25562100
N	7.44520100	-6.13592000	0.50748100
N	-1.59229000	-9.48497300	-0.36849800
N	-9.06737200	-3.35571600	-0.26294200
N	-7.61768900	6.13963500	0.27855800

Structure S70. The coordinates of both the optimized structures of N6L$_3$ at S$_0$ and S$_1$ in toluene.

S_0-optimized structure				S_1-optimized structure			
C	-4.81072300	9.92124000	0.31845900	C	9.63257400	-5.37340200	0.28475200
C	-5.80694700	10.10000000	1.28255400	C	10.54929500	-4.94783600	1.25019200
C	-4.23996900	11.04724400	-0.28182000	C	9.81088900	-6.62279500	-0.31604300
C	-6.22522900	11.37672100	1.62845700	C	11.62329300	-5.75412200	1.59781300
H	-6.25022200	9.23306400	1.75805000	H	10.41360400	-3.98359300	1.72549000
C	-4.65267100	12.32026400	0.08389800	C	10.87887100	-7.42866700	0.05050600
H	-3.47315200	10.91797100	-1.03642900	H	9.10934600	-6.95542200	-1.07189600
C	-5.64968800	12.49530100	1.03673100	C	11.79393700	-7.00025500	1.00549600
H	-6.99944700	11.49565300	2.37864700	H	12.32440700	-5.40836400	2.34953300
H	-4.19891600	13.18193400	-0.39358300	H	11.00270700	-8.39444400	-0.42716400
H	-5.97451200	13.49140600	1.31447600	H	12.63092300	-7.62996100	1.28436200
C	5.83130300	9.35574200	0.31701200	C	0.58019400	-10.98797900	0.40182000
C	5.38397900	10.53687600	-0.28197200	C	1.61322700	-11.71511600	-0.19650800
C	6.84014700	9.42615200	1.28196400	C	-0.19906000	-11.59953000	1.38788400
C	5.92957700	11.75825300	0.08575700	C	1.86300400	-13.02270900	0.19306800
H	4.60842000	10.49088500	-1.03731100	H	2.21432300	-11.24993700	-0.96872200
C	7.39171800	10.65061800	1.62988100	C	0.04914500	-12.91266000	1.75939100
H	7.18834600	8.51627100	1.75630000	H	-0.99649200	-11.03846900	1.86040000
C	6.93880000	11.82488800	1.03950100	C	1.08239600	-13.63181100	1.16918600
H	5.57051600	12.66401600	-0.39067400	H	2.66696600	-13.57355000	-0.28272700
H	8.17354200	10.68545800	2.38075500	H	-0.56389000	-13.37153300	2.52733800
H	7.36750600	12.78038500	1.31895300	H	1.27680300	-14.65593500	1.46623400
C	-11.02248500	0.37047900	0.30317500	C	9.24636700	6.01278600	0.24361800
C	-11.81987000	-0.60660800	-0.29963600	C	9.33308400	7.26338500	-0.37441900
C	-11.59027300	1.20647700	1.26885200	C	10.19754200	5.66394600	1.20650700
C	-13.15109200	-0.74638000	0.06500100	C	10.34584500	8.14562400	-0.02757600
H	-11.39016900	-1.25339900	-1.05541400	H	8.60422700	7.53659100	-1.12821500
C	-12.92711000	1.07035900	1.61373600	C	11.21600000	6.54685000	1.53447400
H	-10.97778500	1.96219600	1.74625200	H	10.13208000	4.69913000	1.69533200
C	-13.71583100	0.09194500	1.01941900	C	11.29575400	7.79368700	0.92461300
H	-13.75453800	-1.50949200	-0.41441500	H	10.39920500	9.11120900	-0.51852500
H	-13.35012700	1.72807200	2.36525300	H	11.94516400	6.26025200	2.28438800
H	-14.75820300	-0.01568800	1.29643200	H	12.08946900	8.48319500	1.18821600
C	-6.18729900	-9.12624800	0.31402700	C	-0.15657500	11.00100100	0.38820100
C	-7.44652700	-9.19773300	-0.28862000	C	0.81289500	11.79268800	-0.23354800
C	-5.84451500	-10.07616000	1.28046300	C	-0.95691900	11.56154100	1.38733100
C	-8.34192500	-10.19235000	0.07695000	C	0.97972900	13.11637600	0.14558600
H	-7.71738100	-8.47072600	-1.04509000	H	1.42827000	11.36485100	-1.01605200
C	-6.74029600	-11.07743900	1.62630500	C	-0.79142000	12.89014100	1.74942100
H	-4.87301200	-10.02437900	1.75759500	H	-1.70441200	10.94863800	1.87679100
C	-7.99549100	-11.14113100	1.03214800	C	0.17834200	13.67482700	1.13517200
H	-9.31400300	-10.23260100	-0.40238900	H	1.73405600	13.71905000	-0.34818400
H	-6.45663000	-11.80565000	2.37832400	H	-1.41834700	13.31018200	2.52823100
H	-8.69508200	-11.92106800	1.30986400	H	0.30770000	14.71145300	1.42427300
C	11.00164000	-0.79468200	0.30132300	C	-9.47570100	-5.63802100	0.22127000
C	11.65527100	-0.02120800	1.26483600	C	-9.60022700	-6.62999900	1.19777700
C	11.69121600	-1.85157900	-0.29990000	C	-10.62048600	-5.16973700	-0.42926700
C	12.97066100	-0.29665400	1.60903500	C	-10.84894700	-7.14719300	1.50818500
H	11.12632100	0.79589400	1.74097500	H	-8.71527000	-6.98604800	1.71147000
C	13.00059900	-2.13011800	0.06423600	C	-11.86564900	-5.68545200	-0.10286000
H	11.19527900	-2.45101500	-1.05412000	H	-10.52380900	-4.40733600	-1.19294100
C	13.65141400	-1.35399800	1.01640800	C	-11.98832000	-6.67754600	0.86391400
H	13.46134100	0.31450800	2.35870700	H	-10.93169400	-7.91448800	2.26991000
H	13.51983200	-2.95360700	-0.41388800	H	-12.74517300	-5.31662300	-0.61885300
H	14.67691000	-1.57026000	1.29292000	H	-12.96311800	-7.08066700	1.11315900
C	5.18743700	-9.72667900	0.32060000	C	-9.83621000	4.98617400	0.26944900
C	4.74334300	-10.63302000	1.28764400	C	-10.02446000	5.95091200	1.26252900
C	6.43382400	-9.93216400	-0.27796700	C	-10.94664800	4.45486100	-0.39150900
C	5.52728500	-11.72256400	1.63828400	C	-11.30462100	6.37881700	1.58069400
H	3.78136300	-10.47787900	1.76165000	H	-9.16412300	6.35457100	1.78283200
C	7.21804600	-11.01497200	0.09242900	C	-12.22300600	4.88230400	-0.05840700
H	6.78232400	-9.23958500	-1.03495400	H	-10.79879900	3.71419000	-1.16827400
C	6.77053500	-11.91972300	1.04834100	C	-12.41009400	5.84678800	0.92590600
H	5.16602000	-12.41509200	2.39075000	H	-11.43828200	7.12519400	2.35582000
H	8.18187700	-11.15869800	-0.38371900	H	-13.07655800	4.46663300	-0.58226500
H	7.38311900	-12.76850400	1.32973400	H	-13.40921900	6.18111200	1.18060800
H	-5.81290500	3.77500600	1.63993800	H	6.91472300	0.23432000	1.59133900
C	-6.56029700	7.85351300	-0.83258500	C	9.88341400	-2.67768800	-0.86154500
C	-5.33965700	7.59461700	-0.19586200	C	8.73034700	-3.16638800	-0.23100700
C	-5.07117700	6.30808500	0.28817500	C	7.76717400	-2.26519200	0.24459000
C	-6.01680000	5.31418400	0.12494800	C	7.97273200	-0.91005300	0.08204100
C	-7.24540000	5.58059300	-0.49992900	C	9.13628300	-0.42217200	-0.53761200
C	-7.51515000	6.85901000	-0.97700600	C	10.09451600	-1.31654700	-1.00811400
H	-6.75431300	8.84890600	-1.21396700	H	10.61594800	-3.38183800	-1.23774400
H	-4.12922900	6.10792600	0.78657000	H	6.87795600	-2.64124100	0.73805000
H	-8.45441000	7.08412300	-1.47068700	H	10.99546600	-0.96271200	-1.49766200
C	-5.96105700	3.87328800	0.55890300	C	7.09863700	0.23871500	0.51135300
H	-5.13391000	3.33627800	0.08139800	H	6.11574800	0.20459600	0.02831400
C	-7.30005900	3.34107500	0.12131700	C	7.89303500	1.44247900	0.07790300
C	-7.82543400	2.07329200	0.28055500	C	7.59601200	2.78102400	0.23549600
C	-9.10950000	1.79824900	-0.20636500	C	8.49496000	3.74396000	-0.24543000
C	-9.83994200	2.81063200	-0.84200200	C	9.67819600	3.33189300	-0.87538000
C	-9.31782100	4.08707800	-0.98231900	C	9.98147200	1.98776300	-1.01580100
C	-8.04070900	4.35769500	-0.50223300	C	9.08679500	1.03214800	-0.54058300
H	-7.26140800	1.29225400	0.77812200	H	6.68356500	3.09760100	0.72821200
H	-10.82779400	2.58533700	-1.22578500	H	10.36054600	4.08236200	-1.25625000
H	-9.90362200	4.85556100	-1.47515300	H	10.90410700	1.69408200	-1.50472100
H	0.54379700	10.26810100	1.09996500	H	5.45073200	-8.72198100	1.11157600
C	3.39693800	7.22998600	-1.32637200	C	1.34878500	-7.86387500	-1.28100400
C	3.88641000	7.99832400	-0.26208400	C	1.39737000	-8.77492700	-0.21509200
C	2.98624000	8.65818000	0.58421200	C	2.52409800	-8.80033700	0.62135800
C	1.63049000	8.55332100	0.34305700	C	3.57143900	-7.93956600	0.36888700
C	1.14185100	7.77825400	-0.72073900	C	3.52012500	-7.02462200	-0.69825900
C	2.03408800	7.10919800	-1.55234600	C	2.39531700	-6.98902300	-1.52045600
H	4.09995100	6.72937100	-1.98143900	H	0.47952300	-7.85747500	-1.92766800
H	3.36191400	9.24603800	1.41418100	H	2.56003000	-9.49464900	1.45325900
H	1.67976800	6.51019200	-2.38440900	H	2.33624400	-6.29907600	-2.35535200
C	0.48583700	9.17406500	1.09839900	C	4.87271400	-7.79129700	1.11151800

H	0.46969600	8.86180500	2.14852300
C	-0.71862200	8.67781800	0.34397500
C	-2.05549700	8.92551300	0.58603900
C	-3.02095000	8.36471400	-0.25967700
C	-2.61635100	7.54860500	-1.32403700
C	-1.27410500	7.28405800	-1.55079800
C	-0.31551700	7.85532300	-0.72007100
H	-2.36635300	9.55000400	1.41606900
H	-3.36889500	7.12534500	-1.97865500
H	-0.98569400	6.65095800	-2.38307700
H	-7.91732100	-4.02524900	2.13942600
C	-5.22870600	-6.03894500	-1.32803500
C	-5.73501200	-6.79792100	-0.26489200
C	-6.70546200	-6.24271700	0.57884700
C	-7.15932000	-4.96120000	0.33610700
C	-6.64651000	-4.20027700	-0.72652200
C	-5.67075300	-4.74438000	-1.55542700
H	-4.48432800	-6.47848000	-1.98122100
H	-7.09237400	-6.82436500	1.40801800
H	-5.26481700	-4.17786600	-2.38666200
C	-8.19324300	-4.16688600	1.08854600
H	-9.16955900	-4.66397600	1.08746600
C	-8.22650200	-2.86483400	0.33379300
C	-8.99596600	-1.74342800	0.57374400
C	-8.87293000	-0.63358800	-0.27178900
C	-7.96017600	-0.67242700	-1.33383700
C	-7.17336100	-1.79199100	-1.55860600
C	-7.30847900	-2.89968800	-0.72791000
H	-9.69494100	-1.71256400	1.40202900
H	-7.87683900	0.18714200	-1.98818100
H	-6.47560600	-1.79859100	-2.38913600
H	8.62604600	-5.60562500	1.08903000
C	4.56279500	-6.55737800	-1.32778800
C	4.98523200	-7.36474700	-0.26367400
C	6.00872900	-6.91494800	0.58014700
C	6.59594000	-5.68893700	0.33671800
C	6.16715900	-4.87887400	-0.72680900
C	5.13956800	-5.31709300	-1.55584000
H	3.77629900	-6.91616100	-1.98098900
H	6.33143800	-7.53369900	1.40994100
H	4.79669400	-4.71121400	-2.38772700
C	7.70784900	-5.00798300	1.08911000
H	7.44792300	-4.83702700	2.13970200
C	7.87925800	-3.71737400	0.33339800
C	8.76284300	-2.68340800	0.57307800
C	8.75813700	-1.56729200	-0.27315900
C	7.84715300	-1.51030500	-1.33589100
C	6.94646300	-2.54054600	-1.56031700
C	6.96325900	-3.65572400	-0.72883000
H	9.46064300	-2.72588000	1.40184000
H	7.85558100	-0.64724300	-1.99089600
H	6.25222600	-2.47420700	-2.39117100
H	6.17861800	3.13906900	1.63677700
C	10.08194900	1.75566400	-0.84463600
C	9.24924900	0.82604600	-0.20831300
C	8.00123400	1.23478800	0.27847200
C	7.61193800	2.55066300	0.11845300
C	8.45534900	3.48345300	-0.50560300
C	9.69691700	3.07986500	-0.98555500
H	11.04069100	1.42760200	-1.22814300
H	7.35833100	0.51758400	0.77646200
H	10.36036500	3.78221900	-1.47864800
C	6.33630900	3.22092600	0.55572600
H	5.45731700	2.77363700	0.07838000
C	6.54340400	4.64784300	0.12132900
C	5.70790800	5.73597900	0.28452600
C	6.11064200	6.98694600	-0.19961400
C	7.35161600	7.11560200	-0.83660700
C	8.19628800	6.02597200	-0.98087100
C	7.79320800	4.78322200	-0.50363400
H	4.75015900	5.63641200	0.78306200
H	7.64954200	8.08503200	-1.21788400
H	9.15408300	6.15094400	-1.47444500
H	-0.36692700	-6.91800800	1.64279500
C	-3.52049200	-9.60631700	-0.83428800
C	-3.90802600	-8.41994100	-0.19812200
C	-2.92925800	-7.54372800	0.28742000
C	-1.59532500	-7.86508100	0.12611700
C	-1.21034000	-9.06219000	-0.49809100
C	-2.18147900	-9.93550100	-0.97661000
H	-4.28466500	-10.27238900	-1.21675300
H	-3.22810700	-6.62804600	0.78537800
H	-1.90572400	-10.86144300	-1.46975600
C	-0.37630100	-7.09578300	0.56180300
H	-0.32431800	-6.11107400	0.08409000
C	0.75509300	-7.98902700	0.12625100
C	2.11541700	-7.80983500	0.28783900
C	2.99665100	-8.78429700	-0.19731000
C	2.48654800	-9.92334100	-0.83351600
C	1.12033200	-10.10969300	-0.97621500
C	0.24638300	-9.13903100	-0.49793200
H	2.50876500	-6.93068300	0.78589800
H	3.17650500	-10.66617400	-1.21573900
H	0.74873400	-11.00145900	-1.46943600
N	-5.27606800	-8.11278300	-0.04763300
N	4.38940100	-8.62349400	-0.04583500
N	9.66796100	-0.51211300	-0.05876400
N	5.27467500	8.11228900	-0.04657600
N	-4.38931100	8.62518400	-0.04316300
N	-9.66660600	0.51145100	-0.05744100
H	4.71708700	-7.52145500	2.16199500
C	5.56998200	-6.69827600	0.34614000
C	6.80997100	-6.13808400	0.57290100
C	7.27236600	-5.12525500	-0.28023100
C	6.46089300	-4.68902700	-1.33736100
C	5.20811800	-5.24067700	-1.55098000
C	4.75546400	-6.25711200	-0.71197800
H	7.43066900	-6.47058600	1.39734200
H	6.82932100	-3.91251200	-1.99713900
H	4.60176700	-4.88888000	-2.37870600
H	4.21281100	7.84212400	2.14418400
C	0.80293000	7.91795100	-1.27516400
C	0.79873700	8.84436900	-0.22009100
C	1.92889000	8.95579300	0.60696800
C	3.02829900	8.16367100	0.35647300
C	3.03067900	7.23361700	-0.70047600
C	1.90357800	7.11341800	-1.51356800
H	-0.06893900	7.84572900	-1.91426100
H	1.92424200	9.66078800	1.43062200
H	1.88557500	6.41178900	-2.34061000
C	4.34228400	8.11075400	1.08984500
H	4.85669200	9.07795200	1.07604800
C	5.10543900	7.05854300	0.32989100
C	6.38196800	6.58636500	0.55071000
C	6.90486600	5.59698700	-0.29621500
C	6.11510000	5.09480400	-1.34113800
C	4.82655800	5.55779500	-1.54903200
C	4.31261200	6.55146500	-0.71638400
H	6.98595400	6.96929000	1.36562800
H	6.52966600	4.33779200	-1.99620500
H	4.23824100	5.15715800	-2.36751700
H	-10.33776900	-0.35234800	0.99679200
C	-7.41912600	2.76108300	-1.28820100
C	-8.29997200	3.18346100	-0.27145200
C	-8.93560100	2.21650200	0.54049100
C	-8.69124200	0.88564800	0.31571900
C	-7.80811400	0.45906200	-0.70857400
C	-7.17081500	1.42280300	-1.50799800
H	-6.94777300	3.50864000	-1.91528800
H	-9.60093800	2.53708900	1.33430400
H	-6.50256000	1.12544000	-2.30878900
C	-9.24368600	-0.31461100	1.04010400
H	-8.97458500	-0.30962300	2.10243400
C	-8.61001500	-1.46791100	0.30606200
C	-8.76169300	-2.81468900	0.52075600
C	-8.06345200	-3.72891700	-0.29909600
C	-7.21733300	-3.23955900	-1.31403000
C	-7.05964600	-1.88528700	-1.52277200
C	-7.75955100	-0.97380600	-0.71485900
H	-9.40284900	-3.18618000	1.31230800
H	-6.69695000	-3.94816700	-1.94759600
H	-6.41355800	-1.53697500	-2.32119400
H	-3.24752600	-6.03749300	1.60462100
C	-7.25573000	-7.23357400	-0.81182400
C	-7.10438700	-5.96651900	-0.21837100
C	-5.83499000	-5.56338700	0.23968200
C	-4.76838600	-6.42241500	0.10575300
C	-4.92445700	-7.69767600	-0.47771000
C	-6.18383800	-8.09563900	-0.93644000
H	-8.23075600	-7.52743500	-1.18167600
H	-5.71715300	-4.59009300	0.70226900
H	-6.32656300	-9.06639800	-1.39869000
C	-3.33561500	-6.22828200	0.52938600
H	-2.87377600	-5.37341200	0.02304600
C	-2.69021900	-7.53027800	0.13194500
C	-1.38469000	-7.93715300	0.29793900
C	-1.00183100	-9.21601000	-0.14275900
C	-1.95485100	-10.05378500	-0.74470000
C	-3.26798800	-9.64862200	-0.89807300
C	-3.64908300	-8.37732500	-0.46121000
H	-0.65229700	-7.29119500	0.76912900
H	-1.64715800	-11.03179600	-1.09528200
H	-3.98449800	-10.31444500	-1.36682000
H	-3.63152800	5.79006100	1.60870100
C	-2.63941000	9.90691500	-0.71425800
C	-1.62276600	9.12779300	-0.13387700
C	-1.91479500	7.81911600	0.29688800
C	-3.19134800	7.32960800	0.14459400
C	-4.21527500	8.11722300	-0.42762400
C	-3.92332700	9.41762900	-0.85591000
H	-2.40190800	10.90634000	-1.05908100
H	-1.13504300	7.21816700	0.75118700
H	-4.68813700	10.03921200	-1.30869700
C	-3.74395700	5.98365100	0.53619800
H	-3.23157000	5.16595400	0.01729600
C	-5.19153700	6.08549600	0.13010400
C	-6.19444800	5.15549800	0.26289500
C	-7.49624200	5.47896000	-0.17504400
C	-7.73931300	6.74281700	-0.74875600
C	-6.73052800	7.67548400	-0.87477500
C	-5.43857000	7.35718100	-0.43606700
H	-6.00616600	4.18641200	0.71073700
H	-8.73619200	6.97475900	-1.10424400
H	-6.94369600	8.64000300	-1.32262800
N	-0.32832100	9.65224900	0.00891600
N	-8.53503500	4.54721000	-0.06182700
N	-8.20734500	-5.10839800	-0.10162000
N	0.32468100	-9.65581600	0.01394000
N	8.54336300	-4.55405100	-0.07790000
N	8.21308900	5.11592400	-0.09975100

Structure S71. The coordinates of both the optimized structures of B7L$_3$ at S$_0$ and S$_1$ in toluene.

S_0-optimized structure				S_1-optimized structure			
B	-10.11440800	4.45017400	-0.74724200	B	-9.89295000	4.85466500	-0.76880800
B	-2.82553800	10.32718500	2.81974400	B	-2.39573100	10.47867500	2.77158300
B	-9.64456800	-5.36314900	-2.71632600	B	-9.88757500	-4.98944600	-2.59410100
H	1.75455400	10.97932900	-0.13899100	H	2.21607700	10.92764400	-0.16870200
C	4.44399800	7.11427900	0.93931700	C	4.74563600	6.96463300	0.93875900
C	5.02225100	8.23877300	0.31708800	C	5.36915900	8.06080600	0.30959100
C	4.26609400	9.42606300	0.27952800	C	4.66155900	9.27742600	0.26376100
C	2.98644300	9.46302200	0.80350100	C	3.38418600	9.36930400	0.78627500
C	2.42926000	8.31576400	1.38988200	C	2.78088300	8.24944400	1.38016400
C	3.16715800	7.13934700	1.47773000	C	3.47079400	7.04477000	1.47635600
H	5.01931300	6.19743800	1.00322100	H	5.28345200	6.02574800	1.00895600
H	4.68787700	10.31175500	-0.18515700	H	5.11893200	10.14230400	-0.20642700
H	2.75296500	6.25522100	1.95052700	H	3.02090900	6.18123000	1.95463200
C	2.00112900	10.60046700	0.85913900	C	2.44522000	10.54562700	0.83235600
H	2.39273800	11.45230000	1.42649000	H	2.86963400	11.38464000	1.39520900
C	0.80651400	9.97439200	1.52884500	C	1.22530400	9.97232800	1.50356200
C	-0.41539800	10.53220700	1.86089100	C	0.02516200	10.58019800	1.82665100
C	-1.42612400	9.74031500	2.43515500	C	-1.01782000	9.83330800	2.40365800
C	-1.14661700	8.38174400	2.67536300	C	-0.79187100	8.46717100	2.65742400
C	0.09304400	7.82421700	2.40137300	C	0.42564600	7.85919500	2.39184700
C	1.06994600	8.62624500	1.81986600	C	1.43469100	8.61714400	1.80584400
H	-0.60778900	11.58196300	1.66059500	H	-0.12552100	11.63480300	1.61642700
H	-1.92209700	7.75633100	3.10483100	H	-1.59232100	7.87645600	3.09017400
H	0.28764300	6.78007300	2.62230300	H	0.57865700	6.81020700	2.62233000
B	6.43843800	8.16399300	-0.34613000	B	6.78161600	7.92457600	-0.35157000
B	-2.42548700	-10.16813400	2.13961600	B	-2.78880000	-10.17539400	2.02778600
H	10.78330900	5.30747100	-2.19245400	H	11.00531100	4.88060300	-2.18147000
C	8.98602300	0.85872100	-2.92911600	C	9.02451600	0.50660900	-2.89311100
C	10.37301500	1.05433500	-2.78725800	C	10.41863700	0.64403100	-2.74926700
C	10.82511300	2.31641200	-2.36298900	C	10.92249300	1.88928700	-2.33246000
C	9.91691200	3.32007300	-2.07025300	C	10.05697100	2.93191400	-2.04789800
C	8.54271600	3.10638300	-2.26270000	C	8.67495500	2.77488300	-2.24069700
C	8.07121600	1.87478300	-2.70432200	C	8.15286000	1.56114600	-2.67555400
H	8.62406100	-0.11665600	-3.23633500	H	8.62220700	-0.45410300	-3.19631400
H	11.88989600	2.49026500	-2.23803300	H	11.99335200	2.01887000	-2.20611600
H	7.00978800	1.70156900	-2.84612700	H	7.08534400	1.43156900	-2.81822400
C	10.14989700	4.70697500	-1.52969600	C	10.34726300	4.31106300	-1.51540800
H	10.65317300	4.68380600	-0.55676600	H	10.84879200	4.27284200	-0.54207100
C	8.75436300	5.26646600	-1.43091600	C	8.97617300	4.92866500	-1.42089600
C	8.32213600	6.49664200	-0.96864000	C	8.59511700	6.17790500	-0.96518000
C	6.94948100	6.81194300	-0.94599100	C	7.23655300	6.54978100	-0.94464300
C	6.04832900	5.85134200	-1.44684900	C	6.29644200	5.62468100	-1.44121200
C	6.46696300	4.61826900	-1.91999000	C	6.66372600	4.37315500	-1.90806000
C	7.82540600	4.31977500	-1.88978100	C	8.00882600	4.01874700	-1.87547400
H	9.04088200	7.22016700	-0.59695800	H	9.34294600	6.87301500	-0.59671100
H	4.98986900	6.08546000	-1.46494200	H	5.24861000	5.90241200	-1.46079000
H	5.74641500	3.90004100	-2.29651400	H	5.91431700	3.68333800	-2.28135200
H	2.79851700	-10.35315600	3.64775700	H	2.33812100	-10.36603100	3.70973000
C	5.04479100	-7.41358500	0.54021000	C	4.77615100	-7.95156300	0.29065900
C	5.75898800	-8.17769400	1.48445100	C	5.42680700	-8.52977900	1.41702800
C	5.07121900	-9.21061500	2.15179600	C	4.68007900	-9.45738900	2.19246000
C	3.72474000	-9.42039700	1.91840800	C	3.36144300	-9.70973600	1.90770000
C	3.02998600	-8.59856300	1.01537700	C	2.71899800	-9.04992700	0.82331500
C	3.69323500	-7.60529500	0.30353800	C	3.45752100	-8.19163000	-0.01296400
H	5.56284800	-6.62238800	0.01037500	H	5.33571600	-7.25620100	-0.32443500
H	5.60165300	-9.83046300	2.86762900	H	5.15697300	-9.94707300	3.03473100
H	3.16252100	-6.97518000	-0.40208400	H	2.98500600	-7.70138600	-0.85707300
C	2.77521400	-10.40269300	2.55413300	C	2.36200200	-10.57142200	2.63519000
H	3.02212600	-11.43615500	2.28393400	H	2.59518600	-11.63714100	2.52498200
C	1.44046200	-9.98507000	1.99323500	C	1.06536700	-10.21700500	1.95382900
C	0.16854500	-10.42636200	2.30907600	C	-0.22302600	-10.54850700	2.28634200
C	-0.96122500	-9.84397000	1.70116800	C	-1.32535800	-10.03295600	1.54642600
C	-0.74454100	-8.83908000	0.73710300	C	-1.01652200	-9.23300200	0.40497300
C	0.52302900	-8.38125400	0.41631000	C	0.26585200	-8.89371400	0.05343800
C	1.61574500	-8.94653300	1.06373000	C	1.32990800	-9.35562000	0.85209700
H	0.03391300	-11.21533300	3.04222700	H	-0.41533000	-11.19077900	3.13888100
H	-1.60225500	-8.38306200	0.25635000	H	-1.83659000	-8.83774100	-0.18307500
H	0.65370900	-7.58180500	-0.30506600	H	0.45489900	-8.25376900	-0.80149100
B	11.33272500	-0.14341100	-3.09962600	B	11.33014800	-0.59330200	-3.04962800
B	7.21431900	-7.74825000	1.85056100	B	6.82657300	-8.01651300	1.84312600
C	-10.71596200	-5.75761800	-3.78532500	C	-10.98461000	-5.33667900	-3.65466800
C	-11.28220300	-7.04306700	-3.79072400	C	-11.62547600	-6.58724300	-3.64263900
C	-11.14399900	-4.86260700	-4.77943400	C	-11.36387400	-4.43304500	-4.66157400
C	-12.24302000	-7.41029300	-4.72285000	C	-12.60701300	-6.91189100	-4.56881300
H	-10.96714800	-7.76182500	-3.04142800	H	-11.35392000	-7.31293800	-2.88276700
C	-12.08453600	-5.23091600	-5.73190600	C	-12.32688800	-4.75954400	-5.60672600
H	-10.71637500	-3.86627100	-4.81585700	H	-10.87888200	-3.46408900	-4.71440000
C	-12.64228300	-6.50430300	-5.69932000	C	-12.95716700	-5.99814000	-5.55696100
H	-12.67538000	-8.40465900	-4.69471200	H	-13.09554500	-7.87940700	-4.52575600
H	-12.38583200	-4.52562500	-6.49893700	H	-12.58841200	-4.04815200	-6.38276000
H	-13.38445000	-6.79163700	-6.43646800	H	-13.71664300	-6.25208800	-6.28865200
C	-11.32860700	5.10893200	-1.48633100	C	-11.05339000	5.55971500	-1.55008400
C	-11.80498600	4.61117000	-2.71090800	C	-11.53090100	5.05892100	-2.77312700
C	-11.94552500	6.26658100	-0.98421300	C	-11.61452300	6.76253900	-1.09021800
C	-12.85092600	5.22357100	-3.38849600	C	-12.52576300	5.71167600	-3.48879500
H	-11.32988600	3.74292500	-3.15388500	H	-11.09595400	4.15483500	-3.18456700
C	-13.01257900	6.86502300	-1.63806700	C	-12.63134500	7.40348100	-1.78265300
H	-11.57958500	6.70646400	-0.06302300	H	-11.24463400	7.20389400	-0.17128900
C	-13.46698800	6.34459500	-2.84512100	C	-13.08860000	6.87915600	-2.98697100
H	-13.18681300	4.82620200	-4.34021000	H	-12.86314900	5.30986700	-4.43812700
H	-13.48074100	7.74795900	-1.21659100	H	-13.05752700	8.32172700	-1.39329500
H	-14.29072900	6.81985300	-3.36690500	H	-13.87277400	7.38648100	-3.53867600
C	-2.93654300	11.75521900	3.44702100	C	-2.45442600	11.91973000	3.37617300
C	-4.09187200	12.53770100	3.28506400	C	-3.57308900	12.74796300	3.18676900

C	-1.88424600	12.30957000	4.19446100
C	-4.18878100	13.81122500	3.82878900
H	-4.92221100	12.14203700	2.70986900
C	-1.98288600	13.57235000	4.76232000
H	-0.97872400	11.73078500	4.34284500
C	-3.13497900	14.32819500	4.57446400
H	-5.08673300	14.40021800	3.67613300
H	-1.16131800	13.97071500	5.34789600
H	-3.21156700	15.31940700	5.00865700
C	7.34226100	9.44221700	-0.40865900
C	7.36065800	10.37612700	0.63977900
C	8.16863500	9.70130000	-1.51398200
C	8.17309100	11.50134800	0.59670200
H	6.73546700	10.20688300	1.51040700
C	8.96098100	10.83975000	-1.57827400
H	8.17758800	9.00196300	-2.34369100
C	8.97006800	11.73888900	-0.51765800
H	8.18074900	12.19757700	1.42850700
H	9.57676800	11.02420200	-2.45202500
H	9.59692200	12.62325900	-0.55938300
C	-2.75681700	-10.70577800	3.57100000
C	-1.98767100	-10.37871200	4.69959700
C	-3.87419200	-11.53275700	3.77235200
C	-2.31452100	-10.85324500	5.96288200
H	-1.13135200	-9.72240600	4.58705900
C	-4.19041500	-12.03516800	5.02699200
H	-4.49899800	-11.79124600	2.92365000
C	-3.41156600	-11.69168200	6.12664900
H	-1.71353700	-10.57162300	6.82086400
H	-5.04920000	-12.68595500	5.15120500
H	-3.66300000	-12.07182700	7.11105500
C	7.83365700	-8.04224200	3.25711800
C	7.06016200	-8.12605300	4.42627100
C	9.22184600	-8.20937100	3.39140300
C	7.64032200	-8.36877100	5.66422900
H	5.98776900	-7.97467000	4.36649300
C	9.80586000	-8.48228300	4.62049000
H	9.85026300	-8.13117000	2.51026500
C	9.01428300	-8.55845600	5.76130300
H	7.02204500	-8.41129500	6.55448500
H	10.87869900	-8.62458200	4.69289400
H	9.46865600	-8.75790100	6.72597500
C	12.65532100	0.05602400	-3.90125200
C	13.70359800	-0.87523300	-3.81391500
C	12.84784000	1.16740800	-4.73871400
C	14.88869600	-0.70130300	-4.51471700
H	13.58628800	-1.74373200	-3.17447200
C	14.02244000	1.33875500	-5.45766300
H	12.05388500	1.90021300	-4.83584000
C	15.04732700	0.40563900	-5.34168100
H	15.68802400	-1.42845200	-4.42113600
H	14.14222000	2.19898800	-6.10732200
H	15.96959700	0.54066200	-5.89672700
H	10.27840600	-3.21629100	1.31171100
C	8.26717000	-7.24827400	-0.49642400
C	8.03689800	-6.87501000	0.83880000
C	8.47883700	-5.60996900	1.25869900
C	9.06779500	-4.74163600	0.35774900
C	9.31786900	-5.14793800	-0.96211100
C	8.93258200	-6.41655800	-1.38772600
H	7.91647300	-8.21564600	-0.84216500
H	8.31647300	-5.29876400	2.28655400
H	9.11438400	-6.74101800	-2.40701200
C	9.49312600	-3.31301800	0.55302600
H	8.66127000	-2.68193800	0.88430800
C	9.99237800	-2.92876300	-0.81195000
C	10.42660900	-1.70077200	-1.27146900
C	10.89464300	-1.55778600	-2.58904700
C	10.89581800	-2.69390500	-3.41711400
C	10.39095400	-3.91586300	-2.98919400
C	9.93070500	-4.03073500	-1.68077500
H	10.40921400	-0.83721400	-0.61325000
H	11.26792900	-2.60531200	-4.43286900
H	10.35894000	-4.76212600	-3.66744300
H	-5.71932700	7.69822700	-0.97131300
C	-9.51849900	5.84992200	1.37086000
C	-9.15730500	5.37359100	0.10012500
C	-7.91238100	5.75976400	-0.42023400
C	-7.07701300	6.59131800	0.30564700
C	-7.46803800	7.07187300	1.56414600
C	-8.69265700	6.69374800	2.10341900
H	-10.46863200	5.54959800	1.80231400
H	-7.60303800	5.40997500	-1.40146700
H	-9.00411100	7.04599100	3.08128200
C	-5.70961500	7.11077100	-0.04634300
H	-4.99162800	6.29768300	-0.20203800
C	-5.35818700	7.95622600	1.14785000
C	-4.21065500	8.68266900	1.40322400
C	-4.10580900	9.46677900	2.56681000
C	-5.18745900	9.45602300	3.46712600
C	-6.32053100	8.68407600	3.25085200
C	-6.40902200	7.93772000	2.07928400
H	-3.38895200	8.66941200	0.69355900
H	-5.12572800	10.05682600	4.36858900
H	-7.12582700	8.67704500	3.97787000
H	-12.12544100	-0.69749100	-1.02587800
C	-8.21893400	-3.40730800	-1.84827000

C	-1.38896400	12.44084200	4.12885800
C	-3.62240000	14.03303000	3.70941000
H	-4.41219700	12.37901800	2.60655100
C	-1.44089400	13.71552200	4.67600300
H	-0.51071000	11.82675000	4.29789800
C	-2.55710800	14.51662900	4.46110000
H	-4.49227100	14.65721300	3.53544400
H	-0.61052100	14.08784100	5.26628900
H	-2.59667400	15.51704100	4.87881500
C	7.73783800	9.16385100	-0.41802900
C	7.79182200	10.10144500	0.62592800
C	8.57790500	9.38289900	-1.52171500
C	8.65043100	11.19172200	0.58018500
H	7.15742100	9.96272200	1.49528000
C	9.41758800	10.48675100	-1.58852300
H	8.56034900	8.67985600	-2.34814700
C	9.46057300	11.39012000	-0.53232600
H	8.68423500	11.89119600	1.40860200
H	10.04363500	10.64076200	-2.46087100
H	10.12411500	12.24720200	-0.57610500
C	-3.14463800	-10.58795900	3.50200200
C	-2.38982700	-10.18422000	4.61720100
C	-4.28354800	-11.37133800	3.76118000
C	-2.74259700	-10.54797300	5.91000300
H	-1.51970200	-9.55318100	4.46873400
C	-4.63288100	-11.75520000	5.04874300
H	-4.90119100	-11.69421400	2.92886400
C	-3.86230300	-11.34298800	6.13044100
H	-2.14435000	-10.20891800	6.74938400
H	-5.51003400	-12.37297000	5.21074300
H	-4.13536700	-11.63439200	7.13899900
C	7.35620300	-8.17807000	3.31144800
C	6.52045000	-8.12386900	4.44035900
C	8.73136200	-8.35268000	3.54781100
C	7.02425200	-8.24744500	5.72817400
H	5.45673700	-7.95790100	4.30619800
C	9.24007100	-8.50132300	4.83060100
H	9.41128500	-8.38211700	2.70238700
C	8.38626400	-8.44673300	5.92702900
H	6.35454500	-8.18718200	6.57965900
H	10.30431100	-8.65209800	4.97787200
H	8.78102300	-8.55215800	6.93191500
C	12.66798600	-0.45018900	-3.83802400
C	13.68318200	-1.41548100	-3.72908000
C	12.90815700	0.64472000	-4.68516700
C	14.88075900	-1.29028700	-4.41891200
H	13.53051600	-2.27185000	-3.08076800
C	14.09545600	0.76751400	-5.39303600
H	12.14125800	1.40349000	-4.79894400
C	15.08638900	-0.19886100	-5.25613100
H	15.65352900	-2.04332100	-4.30837900
H	14.25164100	1.61596500	-6.05043900
H	16.01851700	-0.10189200	-5.80255800
H	10.03695400	-3.57738600	1.35785300
C	7.97405000	-7.57131900	-0.47892600
C	7.69667100	-7.17524300	0.84687000
C	8.16783700	-5.91526300	1.26627400
C	8.81387300	-5.07771800	0.38064500
C	9.09678900	-5.50496600	-0.92994100
C	8.68857500	-6.77083000	-1.35531400
H	7.61927800	-8.53847700	-0.82090300
H	7.97369900	-5.58689400	2.28274600
H	8.89901200	-7.11278600	-2.36326500
C	9.27462600	-3.66000100	0.57461100
H	8.45155700	-3.00066100	0.87046900
C	9.82965000	-3.30724900	-0.77675200
C	10.31689500	-2.10034600	-1.23479700
C	10.83047100	-1.98677800	-2.53951500
C	10.82239200	-3.13382000	-3.35612900
C	10.26836500	-4.33359600	-2.93309200
C	9.76201400	-4.41997700	-1.63643200
H	10.30750800	-1.22918100	-0.58683500
H	11.22831100	-3.06673100	-4.36038100
H	10.23206200	-5.18657700	-3.60245100
H	-5.34796300	7.88148600	-0.99556200
C	-9.26143000	6.26937000	1.32975000
C	-8.90454400	5.74687500	0.07600400
C	-7.63434100	6.05990500	-0.43245400
C	-6.77140500	6.86834300	0.28716900
C	-7.15780400	7.39637900	1.52810100
C	-8.40694400	7.08935800	2.05641800
H	-10.23174900	6.02563600	1.75186100
H	-7.32768000	5.67282400	-1.40041400
H	-8.71582700	7.47886300	3.02083800
C	-5.37727300	7.31752600	-0.05651000
H	-4.69404100	6.46990100	-0.18137400
C	-5.00520200	8.17605800	1.12202000
C	-3.83128300	8.85957400	1.37554900
C	-3.70723800	9.66587700	2.52211600
C	-4.79934000	9.72148800	3.40829600
C	-5.96200200	8.99443800	3.19422100
C	-6.06800600	8.22546400	2.03874900
H	-3.00264400	8.79557600	0.67684300
H	-4.72279700	10.33948000	4.29688200
H	-6.77598500	9.03934300	3.91011300
H	-12.16392000	-0.19044500	-0.99891200
C	-8.38297400	-3.10579000	-1.68819100

C	-9.48847200	-3.88260000	-2.23328800	C	-9.66552300	-3.52318800	-2.10516900
C	-10.56297400	-2.97283100	-2.21795800	C	-10.69213700	-2.55633000	-2.12647000
C	-10.36260200	-1.65632900	-1.84344500	C	-10.43432300	-1.24894100	-1.76122400
C	-9.07866000	-1.21003100	-1.49012300	C	-9.13815200	-0.86269600	-1.37647400
C	-7.99960500	-2.08727000	-1.48820300	C	-8.10632800	-1.79684700	-1.33329600
H	-7.37972000	-4.09348200	-1.85717200	H	-7.57764500	-3.83139600	-1.67182100
H	-11.55431200	-3.31203900	-2.50189500	H	-11.69207900	-2.84756300	-2.43199300
H	-7.00489600	-1.74791700	-1.21963700	H	-7.10305300	-1.50641700	-1.04048800
C	-11.34231700	-0.51467700	-1.77062000	C	-11.35590300	-0.05820400	-1.72762600
H	-11.85363700	-0.34920700	-2.72509300	H	-11.83629300	0.11863600	-2.69593300
C	-10.46832000	0.64973600	-1.38478600	C	-10.43272900	1.06570800	-1.33683100
C	-10.79018600	1.98556900	-1.23219100	C	-10.68761800	2.41843500	-1.21731400
C	-9.80129000	2.92517800	-0.87845700	C	-9.65925800	3.31316300	-0.85707300
C	-8.49706800	2.44652000	-0.64442900	C	-8.38817600	2.77119400	-0.57769700
C	-8.15425800	1.11574800	-0.81763100	C	-8.11149400	1.42179600	-0.71527600
C	-9.14516700	0.21752500	-1.20151400	C	-9.13813300	0.56759100	-1.11064400
H	-11.81355700	2.31445200	-1.37902000	H	-11.68823700	2.79719600	-1.39671400
H	-7.73139200	3.14614100	-0.32950900	H	-7.59512900	3.43698900	-0.25702500
H	-7.13433800	0.78459800	-0.65437700	H	-7.11487000	1.04164500	-0.51845000
H	-6.34564700	-6.60345200	1.41985600	H	-6.56167400	-6.41930300	1.46449500
C	-3.59930600	-10.18729700	-0.19311000	C	-4.04397800	-10.12135800	-0.27856900
C	-3.60467100	-9.82415600	1.16562500	C	-3.96786400	-9.75361400	1.09097400
C	-4.65123600	-9.00989300	1.62964100	C	-4.97477300	-8.89561100	1.59473800
C	-5.60155600	-8.52468500	0.74887700	C	-5.92727100	-8.36672100	0.75621500
C	-5.57932500	-8.90913100	-0.60034300	C	-5.96984600	-8.74059200	-0.60816300
C	-4.58922800	-9.76828200	-1.07032700	C	-5.03165400	-9.65177500	-1.11725100
H	-2.79158600	-10.80732700	-0.56888400	H	-3.29295400	-10.79621600	-0.67554300
H	-4.68047400	-8.72007600	2.67582300	H	-4.94741700	-8.60915400	2.64101900
H	-4.56388000	-10.07240300	-2.11143500	H	-5.06263100	-9.95474300	-2.15817800
C	-6.71411300	-7.54132700	0.99078800	C	-6.98443500	-7.33387400	1.03636100
H	-7.46124000	-7.93169400	1.69178800	H	-7.73174500	-7.69714800	1.75125200
C	-7.29204200	-7.35206100	-0.38532200	C	-7.58640000	-7.10420300	-0.32233100
C	-8.25940500	-6.46527200	-0.81798900	C	-8.51470300	-6.17048200	-0.72597600
C	-8.65514300	-6.44463700	-2.16749100	C	-8.94042700	-6.11936300	-2.07024300
C	-8.05258900	-7.36387200	-3.04582000	C	-8.41107700	-7.07419100	-2.96992400
C	-7.04080900	-8.22147800	-2.63546000	C	-7.44060800	-7.98259200	-2.59464500
C	-6.64932200	-8.20230900	-1.30079600	C	-7.00703200	-7.98906500	-1.26298600
H	-8.71033200	-5.76857100	-0.11763800	H	-8.91623600	-5.45764900	-0.01262300
H	-8.35956100	-7.37323500	-4.08645200	H	-8.74995200	-7.06024000	-4.00012400
H	-6.56031300	-8.88316400	-3.34838800	H	-7.01569400	-8.66761100	-3.32005500

Structure S72. The coordinates of both the optimized structures of B6N1L_3 at S_0 and S_1 in toluene.

S_0-optimized structure				S_1-optimized structure			
H	0.13231000	10.93442100	-0.27819100	H	-0.73776800	10.84318700	-0.31575100
C	3.41330000	7.64230300	1.03096900	C	2.78404000	7.83807800	1.06139600
C	3.79383900	8.77615400	0.28615200	C	3.08089300	9.00214100	0.32450700
C	2.86091500	9.82607200	0.17471300	C	2.06989900	9.97076000	0.18998600
C	1.60833100	9.71946900	0.74910000	C	0.81789200	9.75881300	0.73883000
C	1.25191300	8.56516700	1.46558700	C	0.54759200	8.57770400	1.44729200
C	2.16569000	7.52747800	1.62449100	C	1.53859000	7.61677800	1.62665900
H	4.12360400	6.83145200	1.14961700	H	3.55777200	7.09019500	1.19316500
H	3.12582100	10.71619800	-0.38736700	H	2.27360300	10.88106800	-0.36455400
H	1.90809000	6.64012400	2.19307800	H	1.34309400	6.70962100	2.18822700
C	0.46005800	10.69425500	0.73924800	C	-0.40436600	10.63672400	0.70703100
H	0.72717500	11.64575500	1.21259900	H	-0.22534000	11.60878700	1.17950700
C	-0.60449300	9.96320800	1.51316100	C	-1.41523800	9.82324300	1.46719900
C	-1.89633000	10.35815100	1.80614300	C	-2.73148300	10.11620900	1.75879300
C	-2.72926000	9.49007400	2.52267600	C	-3.48988900	9.16695400	2.45405200
C	-2.24837700	8.23690800	2.92492300	C	-2.92582600	7.94967000	2.85740800
C	-0.95415700	7.84228600	2.62811900	C	-1.60023200	7.66850700	2.57807700
C	-0.12437600	8.70965300	1.92464000	C	-0.84177600	8.60715400	1.88287500
H	-2.27608800	11.32135800	1.48380000	H	-3.18782600	11.04785800	1.44484800
H	-2.90454600	7.57781700	3.48096100	H	-3.53081900	7.24444200	3.41447200
H	-0.59863200	6.87050400	2.95316800	H	-1.16485500	6.73287800	2.90912400
B	5.18323800	8.85530600	-0.42669300	B	4.48071000	9.20264200	-0.35831200
H	9.84970600	6.56763700	-2.25713900	H	9.30904100	7.30944800	-2.21042700
C	8.72934200	1.87054900	-2.71946500	C	8.55423200	2.54574700	-2.72969400
C	10.07425100	2.28302100	-2.65641200	C	9.86292100	3.06059800	-2.65846400
C	10.34183800	3.61718100	-2.29995600	C	10.02681300	4.40651400	-2.28556900
C	9.30235400	4.48197200	-2.00226100	C	8.92307300	5.18510500	-1.98070600
C	7.97091100	4.05047400	-2.11531900	C	7.62938400	4.65354100	-2.10315800
C	7.67817600	2.74176400	-2.48315100	C	7.43848800	3.33037600	-2.48616700
H	8.51038700	0.83977800	-2.97635700	H	8.41629400	1.50413100	-2.99870500
H	11.37081100	3.95854800	-2.23558600	H	11.02642200	4.82510600	-2.21525600
H	6.65145500	2.40122700	-2.56458900	H	6.44126500	2.91273600	-2.57484300
C	9.33957700	5.91531400	-1.53941900	C	8.84934300	6.61155100	-1.50163900
H	9.87468200	6.02110200	-0.58912300	H	9.37242800	6.74742200	-0.54851400
C	7.87931700	6.26206700	-1.40611200	C	7.36662900	6.84353700	-1.36919700
C	7.28074100	7.44175200	-1.00132700	C	6.67841300	7.96776300	-0.95101200
C	5.87780900	7.54843600	-0.93703400	C	5.27055800	7.96503400	-0.89005400
C	5.11673400	6.42954300	-1.32970500	C	4.59881100	6.79552600	-1.29929700
C	5.70189500	5.24454500	-1.74607300	C	5.27450700	5.66508200	-1.72887100
C	7.09016800	5.15869300	-1.76625500	C	6.66550000	5.68688000	-1.74518100
H	7.89302600	8.28998500	-0.71166000	H	7.22307100	8.85560000	-0.64587100
H	4.03497800	6.50003300	-1.31129200	H	3.51470000	6.78091500	-1.28449800
H	5.08681600	4.40189900	-2.04352500	H	4.72716900	4.78147900	-2.03908200
H	4.10217100	-10.07011100	3.57015100	H	4.94237800	-9.78537500	3.48713300
C	6.00068800	-6.80410200	0.55773400	C	6.55651200	-6.30087100	0.55106400
C	6.79568900	-7.50209600	1.48862600	C	7.41661400	-6.97076900	1.44396200
C	6.23188100	-8.62624000	2.12407900	C	6.94337000	-8.14931800	2.05509200
C	4.92136500	-8.98881600	1.87332600	C	5.65596400	-8.59362600	1.81674100
C	4.13915000	-8.23259700	0.98466300	C	4.80542800	-7.86513700	0.96829100
C	4.68315400	-7.14917300	0.30417500	C	5.25979400	-6.72617300	0.31342000
H	6.42367500	-5.94358800	0.05216500	H	6.91035100	-5.40000400	0.06291000
H	6.82814000	-9.19728400	2.82864500	H	7.59226900	-8.70022700	2.72860500
H	4.08509200	-6.56852600	-0.38983900	H	4.60942200	-6.16740100	-0.35085800
C	4.09156800	-10.09334100	2.47544000	C	4.91677400	-9.77434900	2.39240400
H	4.46139300	-11.08269600	2.18142500	H	5.35282400	-10.72417000	2.06128500
C	2.71988100	-9.82278500	1.91302800	C	3.52018500	-9.58597500	1.85758500
C	1.50717300	-10.41894200	2.20671200	C	2.35958900	-10.28402400	2.13913200
C	0.31997700	-9.95798600	1.60388100	C	1.13337700	-9.89283000	1.56635200
C	0.42218100	-8.91131300	0.66574200	C	1.14282200	-8.80063500	0.67578900
C	1.62860100	-8.29923300	0.36730700	C	2.29571600	-8.08814400	0.38879800
C	2.77631700	-8.74736900	1.01104500	C	3.48348900	-8.47640900	0.99739900
H	1.46228200	-11.23662000	2.91908300	H	2.38586300	-11.13072900	2.81788800
H	-0.48063500	-8.54803800	0.18860400	H	0.20831500	-8.48598600	0.22626400
H	1.66827800	-7.47207600	-0.33325700	H	2.26266400	-7.23240900	-0.27698000
C	-9.85208700	-6.94822500	-3.84319700	C	-9.26801800	-7.61691500	-3.75516300
C	-10.25502200	-8.29380400	-3.85418600	C	-9.58292600	-8.98603100	-3.76180300
C	-10.40636000	-6.10332500	-4.81870500	C	-9.80810200	-6.83895900	-4.79256500
C	-11.18023000	-8.76797900	-4.77393000	C	-10.41310000	-9.54300200	-4.72555400
H	-9.84011700	-8.97526100	-3.11881900	H	-9.17024600	-9.62415000	-2.98660700
C	-11.31185500	-6.57581700	-5.75898900	C	-10.62156100	-7.39100300	-5.77399800
H	-10.10615000	-5.06132800	-4.85013300	H	-9.56935200	-5.78154100	-4.83843000
C	-11.70687500	-7.90883100	-5.73220300	C	-10.93445600	-8.74536200	-5.73848200
H	-11.48605500	-9.80835300	-4.75022900	H	-10.64858200	-10.60181300	-4.69242800
H	-11.71270300	-5.90549000	-6.51170800	H	-11.01252100	-6.76405600	-6.56884300
H	-12.42162800	-8.27867900	-6.45963700	H	-11.57576300	-9.17799900	-6.49925300
C	-11.76890700	3.73966600	-1.31121300	C	-12.09627500	2.92470500	-1.42804200
C	-12.23435600	3.22249100	-2.53171400	C	-12.39922300	2.40841200	-2.70316400
C	-12.50066800	4.79471700	-0.74205600	C	-12.95941500	3.92882400	-0.94992800
C	-13.37690500	3.72032700	-3.14390300	C	-13.49837900	2.83824500	-3.43358700
H	-11.67656600	2.43283500	-3.02300300	H	-11.74040000	1.66630800	-3.14035800
C	-13.66129200	5.27545500	-1.33048200	C	-14.06872900	4.35497700	-1.66670300
H	-12.15094800	5.24467800	0.18092900	H	-12.76236500	4.38771400	0.01305000
C	-14.10074300	4.73961600	-2.53640400	C	-14.34857800	3.80939000	-2.91508300
H	-13.70432400	3.31312300	-4.09442000	H	-13.69008900	2.41668300	-4.41523000
H	-14.21605800	6.07934600	-0.85890500	H	-14.71674700	5.12084100	-1.25213400
H	-14.99908300	5.12421000	-3.00734900	H	-15.21165700	4.14385400	-3.48071000
C	-4.34803800	11.13390300	3.34468300	C	-5.15344000	10.69471500	3.35750500
C	-5.55893200	11.76304700	3.03587300	C	-6.28202700	11.39693500	2.92695800
C	-3.43891500	11.79564100	4.17759300	C	-4.32317100	11.23442300	4.34243800
C	-5.84988500	13.01633400	3.55428700	C	-6.58210300	12.62540700	3.49400500
H	-6.26935700	11.26591200	2.38644600	H	-6.90388000	10.98598100	2.14070200
C	-3.73404800	13.05578600	4.67610000	C	-4.63202700	12.46580100	4.89721300
H	-2.50278500	11.31549100	4.43600000	H	-3.45365100	10.68116200	4.67564300
C	-4.94099200	13.67558500	4.37356400	C	-5.76083700	13.16345400	4.47878200

H	-6.79447700	13.48575800	3.30156800
H	-3.01537300	13.54978500	5.32107700
H	-5.17016800	14.65741400	4.77126000
C	5.87641000	10.24482500	-0.63895000
C	5.77581400	11.26485800	0.32085400
C	6.62365900	10.52285800	-1.79481300
C	6.40099700	12.49230600	0.14593600
H	5.20706900	11.08408500	1.22712600
C	7.22727500	11.75788400	-1.99094300
H	6.72002200	9.75889700	-2.55940200
C	7.12277000	12.74346000	-1.01567400
H	6.32082200	13.25644400	0.91170300
H	7.78441800	11.95153800	-2.90133800
H	7.60278200	13.70543700	-1.16065100
C	-1.37714200	-11.06259500	3.43962500
C	-0.65069000	-10.68620000	4.58109600
C	-2.40268700	-12.00668600	3.61304000
C	-0.93021600	-11.22525700	5.82973700
H	0.13192000	-9.94053100	4.49108200
C	-2.66928900	-12.57155100	4.85251700
H	-2.99413700	-12.30669300	2.75419700
C	-1.93430100	-12.17750000	5.96531400
H	-0.36499100	-10.90477500	6.69831500
H	-3.45613100	-13.31108700	4.95496900
H	-2.14808200	-12.60725100	6.93815000
C	8.83304000	-7.15817500	3.27829600
C	8.07038400	-7.36717300	4.43888700
C	10.23053700	-7.15154700	3.42012900
C	8.67010800	-7.56415000	5.67557900
H	6.98779800	-7.35143300	4.37382800
C	10.83801400	-7.37706900	4.64743400
H	10.84841000	-6.97347100	2.54608200
C	10.05650900	-7.58015200	5.77962800
H	8.05761600	-7.70640800	6.55944400
H	11.91981700	-7.38354300	4.72548400
H	10.52745600	-7.74324100	6.74317500
C	12.44874300	1.62917800	-3.83190400
C	13.63185400	0.87552300	-3.75527900
C	12.44510900	2.73732300	-4.69528400
C	14.75892500	1.21735200	-4.48935400
H	13.66658200	0.01298700	-3.09812300
C	13.56140100	3.07386100	-5.44816900
H	11.54372800	3.33392700	-4.78648400
C	14.72283700	2.31627900	-5.34101500
H	15.66440700	0.62651200	-4.40319400
H	13.52924800	3.92677700	-6.11749500
H	15.59937000	2.58134600	-5.92269400
H	10.65863400	-2.01455100	1.40176200
C	9.17450200	-6.24664800	-0.45911400
C	8.90210400	-5.92705700	0.88210800
C	9.18075200	-4.62315700	1.32264800
C	9.64835200	-3.67145700	0.43451400
C	9.94090400	-4.02080700	-0.89280600
C	9.72309800	-5.32203600	-1.33801800
H	8.94880300	-7.24518300	-0.82008500
H	8.98269800	-4.35209500	2.35562100
H	9.94025100	-5.60342000	-2.36314800
C	9.88283000	-2.20203500	0.65031200
H	8.97946000	-1.69209100	1.00207400
C	10.30918100	-1.73291900	-0.71314200
C	10.56039400	-0.45007400	-1.15947400
C	10.98267400	-0.22540500	-2.48121300
C	11.13434500	-1.33958800	-3.32517100
C	10.81375100	-2.62608000	-2.90922300
C	10.39021300	-2.82082400	-1.59807500
H	10.42987100	0.39390200	-0.48871500
H	11.47480400	-1.18583800	-4.34421500
H	10.89177400	-3.45954700	-3.59942900
H	-6.71285300	7.19583000	-0.92248900
C	-9.90046200	4.53286500	1.54251800
C	-9.59071400	4.22987100	0.20784400
C	-8.48530600	4.86857400	-0.37530300
C	-7.73250800	5.77078900	0.35584000
C	-8.07103000	6.07308700	1.68338200
C	-9.15867000	5.44730000	2.28182200
H	-10.74260200	4.04023600	2.01946900
H	-8.21990300	4.65733000	-1.40780200
H	-9.42916300	5.66199000	3.31052400
C	-6.51419200	6.55107900	-0.05914700
H	-5.68540000	5.89394600	-0.34556200
C	-6.19482500	7.34995200	1.17531100
C	-5.18277400	8.26869200	1.37852900
C	-5.09000000	8.92075700	2.61228800
C	-6.01823300	8.63887500	3.62110800
C	-7.03589100	7.71918200	3.41368000
C	-7.12450700	7.06631400	2.18809400
H	-4.46647900	8.49870700	0.59736100
H	-5.93083900	9.14909400	4.57330400
H	-7.74352000	7.51115100	4.20895300
H	-11.81020800	-2.13250100	-0.97514400
C	-7.62337000	-4.32084300	-1.94261200
C	-8.83476100	-4.94725600	-2.29718000
C	-10.01286100	-4.17836500	-2.24298900
C	-9.96678800	-2.85035800	-1.85843300
C	-8.73826600	-2.24961100	-1.53846700
C	-7.55894200	-2.98560000	-1.57729000
H	-6.70659100	-4.89834000	-1.97946200

H	-7.45421900	13.17079800	3.15342400
H	-3.99191000	12.87832100	5.66801300
H	-5.99726400	14.12585300	4.91670800
C	5.06844200	10.64585300	-0.50501500
C	4.86462400	11.62154800	0.48437800
C	5.81772900	11.02043600	-1.63221700
C	5.39589000	12.89871300	0.36597300
H	4.29075100	11.36657800	1.36947000
C	6.32635700	12.30474100	-1.77225300
H	5.99034800	10.29355900	-2.41903600
C	6.12266900	13.24457400	-0.76796600
H	5.23949700	13.62753300	1.15399800
H	6.88730100	12.57250000	-2.66113900
H	6.52921800	14.24514600	-0.86911200
C	-0.44712400	-11.20371600	3.36559500
C	0.24544800	-10.79215400	4.51582800
C	-1.36816100	-12.25390900	3.51234700
C	0.03167100	-11.39641500	5.74757400
H	0.94869700	-9.96907100	4.44609200
C	-1.56726700	-12.88191100	4.73423800
H	-1.93074500	-12.58636200	2.64612800
C	-0.86891000	-12.45024400	5.85651600
H	0.56797600	-11.04778600	6.62373000
H	-2.27341100	-13.70129400	4.81526600
H	-1.03060400	-12.93043000	6.81578600
C	9.47259700	-6.53407100	3.19388800
C	8.75809100	-6.83199900	4.36565200
C	10.86876300	-6.42447800	3.30257800
C	9.40287900	-7.01651100	5.58143200
H	7.67614100	-6.89749800	4.32658800
C	11.52318300	-6.63635300	4.50794600
H	11.44851800	-6.17638900	2.41951700
C	10.78877300	-6.92947200	5.65191700
H	8.82605200	-7.23003700	6.47490400
H	12.60408500	-6.56265600	4.56004900
H	11.29549700	-7.08299400	6.59875300
C	12.27376300	2.60806600	-3.85231800
C	13.51429000	1.95152000	-3.79067700
C	12.17417400	3.71942700	-4.70586000
C	14.60413400	2.38850100	-4.53042600
H	13.62355200	1.08959400	-3.14101100
C	13.25360200	4.15046400	-5.46424600
H	11.22710600	4.24246500	-4.78533300
C	14.47285900	3.48757900	-5.37245900
H	15.55469600	1.87152100	-4.45609400
H	13.14769700	5.00300400	-6.12637800
H	15.32041400	3.82638500	-5.95876000
H	10.81763700	-1.20335600	1.36682600
C	9.68709200	-5.53334900	-0.51863200
C	9.39672100	-5.24753500	0.82617900
C	9.56641800	-3.92824700	1.27554200
C	9.94699500	-2.93397300	0.39222100
C	10.26069000	-3.24789400	-0.93903200
C	10.15056600	-4.55934100	-1.39327600
H	9.54347300	-6.54442800	-0.88650800
H	9.35203300	-3.68233600	2.31162200
H	10.38386100	-4.81398000	-2.42182700
C	10.05690400	-1.45124700	0.61746500
H	9.11505300	-1.02259100	0.97671000
C	10.43522700	-0.93820700	-0.74452700
C	10.57569600	0.36407900	-1.18289400
C	10.97314100	0.63215200	-2.50443200
C	11.21449200	-0.45981600	-3.35662400
C	11.00402500	-1.77129000	-2.94867700
C	10.60360900	-2.00952600	-1.63747400
H	10.37768200	1.18967100	-0.50573800
H	11.53714700	-0.27126300	-4.37559800
H	11.14874500	-2.59071600	-3.64485000
H	-6.85174700	6.23206300	-0.89243600
C	-10.54211800	4.09735400	1.39893900
C	-10.04002500	3.56857300	0.18289300
C	-8.81783100	4.08047700	-0.29746200
C	-8.14762500	5.06368100	0.39541300
C	-8.69172400	5.59715300	1.58722400
C	-9.90109500	5.10148100	2.09306600
H	-11.46253800	3.68960000	1.80219700
H	-8.40733000	3.68887000	-1.22244300
H	-10.31767400	5.48914900	3.01645900
C	-6.83807700	5.73146900	0.08122000
H	-6.00969500	5.01575200	0.05532800
C	-6.69368800	6.71456700	1.20785800
C	-5.69660300	7.62123900	1.44237100
C	-5.81746600	8.50452200	2.54123300
C	-6.94653100	8.42144900	3.39566800
C	-7.92541500	7.48550700	3.17448900
C	-7.81726300	6.62236200	2.07121300
H	-4.84612700	7.70671600	0.77742900
H	-7.00670200	9.08065800	4.25175200
H	-8.76628300	7.41213500	3.85350700
H	-11.84036200	-2.93339200	-1.34164600
C	-7.44436300	-4.92060800	-1.44682200
C	-8.51465400	-5.59214900	-2.09224200
C	-9.71886200	-4.85173000	-2.25251100
C	-9.81787100	-3.55058900	-1.82400200
C	-8.71950300	-2.90399900	-1.20420500
C	-7.52544800	-3.61484000	-1.01071400
H	-6.50626200	-5.44978000	-1.32033000

H	-10.96226500	-4.63637000	-2.50250000	H	-10.57444200	-5.33095000	-2.71861000
H	-6.60664700	-2.52696700	-1.33351200	H	-6.66817500	-3.13903000	-0.54512100
C	-11.07930000	-1.84235100	-1.73858300	C	-10.97571500	-2.58718400	-1.92017200
H	-11.63600900	-1.72989500	-2.67524700	H	-11.32303600	-2.46273500	-2.95159400
C	-10.34712800	-0.58217400	-1.35885500	C	-10.40204700	-1.31051700	-1.35409900
C	-10.83102400	0.69852300	-1.16671200	C	-10.96820800	-0.06598900	-1.25462200
C	-9.95751700	1.74990300	-0.82528800	C	-10.24540300	1.04096000	-0.70885700
C	-8.59412300	1.44075500	-0.65168200	C	-8.94419800	0.73981000	-0.20470600
C	-8.09125400	0.16757200	-0.86323400	C	-8.36089700	-0.50118800	-0.30719200
C	-8.97410900	-0.84470600	-1.22726600	C	-9.07675700	-1.54939400	-0.91096500
H	-11.89271000	0.89652400	-1.27059200	H	-11.98957000	0.08446800	-1.58857500
H	-7.91354000	2.23047600	-0.35383200	H	-8.37488100	1.53492500	0.26511600
H	-7.03184800	-0.03260000	-0.74411700	H	-7.35576500	-0.66589400	0.06897400
H	-5.37120800	-7.31137100	1.32363500	H	-4.88967300	-7.93929200	1.48230900
C	-2.26003100	-10.57080400	-0.31335500	C	-1.35400900	-10.58493300	-0.38743400
C	-2.30560500	-10.22463300	1.04891000	C	-1.45842800	-10.37974100	1.00069900
C	-3.43199400	-9.52886700	1.51862200	C	-2.67645100	-9.88849900	1.50459700
C	-4.42736400	-9.13803600	0.64066600	C	-3.70363900	-9.54763100	0.64378500
C	-4.36457800	-9.50645600	-0.71177300	C	-3.57404400	-9.76433100	-0.73752500
C	-3.28834400	-10.25060800	-1.18828100	C	-2.40354200	-10.31597700	-1.25357400
H	-1.39088000	-11.09804000	-0.69374500	H	-0.41918400	-10.95736000	-0.79456300
H	-3.49292600	-9.25448000	2.56762600	H	-2.78513100	-9.72459900	2.57273600
H	-3.23022100	-10.54068200	-2.23207000	H	-2.29426900	-10.49169600	-2.31865800
C	-5.63834100	-8.28106400	0.89014700	C	-5.02065400	-8.88203900	0.94034800
H	-6.33765500	-8.75267800	1.59045300	H	-5.66618700	-9.51166300	1.56417500
C	-6.23667500	-8.14728700	-0.48369900	C	-5.60451300	-8.67616300	-0.43196300
C	-7.29894700	-7.37209100	-0.90808000	C	-6.74805200	-7.99477300	-0.80787200
C	-7.70179000	-7.39115400	-2.25542400	C	-7.11029800	-7.89491600	-2.16311600
C	-7.00234900	-8.23080800	-3.14153100	C	-6.29306400	-8.54816000	-3.10522300
C	-5.89781000	-8.96991900	-2.73983300	C	-5.12153900	-9.20039200	-2.74751100
C	-5.50643200	-8.91533800	-1.40606300	C	-4.76633100	-9.24754000	-1.40268400
H	-7.82252600	-6.73417000	-0.20228600	H	-7.36617400	-7.51422700	-0.05520900
H	-7.31151400	-8.27030800	-4.18084900	H	-6.57039600	-8.50584900	-4.15366400
H	-5.34826200	-9.56890600	-3.45832300	H	-4.49024800	-9.65033500	-3.50705600
N	-4.05451600	9.85887600	2.83295400	N	-4.83784800	9.44315800	2.77993400
B	11.19261100	1.24006400	-2.99250700	B	11.06021400	2.11204000	-3.00712700
B	8.18872800	-6.91178200	1.87412700	B	8.77578300	-6.29966600	1.81316300
B	-1.09978800	-10.45694700	2.02401900	B	-0.23876000	-10.52994600	1.96759000
B	-8.81933200	-6.43338200	-2.78774900	B	-8.32500200	-7.01623600	-2.64744500
B	-10.45493800	3.21802000	-0.63861100	B	-10.81458200	2.45410300	-0.64424400

Structure S73. The coordinates of both the optimized structures of B5N2L$_3$ at S_0 and S_1 in toluene.

S_0-optimized structure				S_1-optimized structure			
B	-9.86900100	4.38841700	-0.57637700	B	-9.98297200	4.65184300	-0.40968400
B	-9.52143300	-5.38102900	-2.78275800	B	-9.76301200	-5.07374800	-2.83626300
H	1.38272200	10.57226400	-0.49539700	H	2.05562700	10.84091300	-0.08664400
C	4.47122300	7.22656100	1.13746900	C	4.55240500	6.77458500	0.73130500
C	4.86494900	8.21449800	0.22422500	C	5.15254700	7.92234200	0.15148700
C	4.01651900	9.30135300	-0.02254700	C	4.46410300	9.15739800	0.19115100
C	2.79940500	9.37112300	0.62830900	C	3.21174300	9.20655800	0.74558700
C	2.38779300	8.35446100	1.50328100	C	2.59276800	8.04420500	1.26508400
C	3.23509800	7.28189700	1.76153600	C	3.29078800	6.82830400	1.26917000
H	5.14193200	6.40170500	1.34487700	H	5.10751000	5.84700800	0.75824200
H	4.31810500	10.07142700	-0.72337400	H	4.91374000	10.03961900	-0.24573500
H	2.94797400	6.49643800	2.45227100	H	2.85466300	5.93792300	1.70611100
C	1.73426900	10.43121400	0.53251400	C	2.28216100	10.38129600	0.88062500
H	2.10221200	11.40586700	0.87215000	H	2.71257400	11.16705500	1.51017300
C	0.64951600	9.89615000	1.42963700	C	1.06132800	9.76430100	1.50635900
C	-0.59283100	10.43220500	1.71113000	C	-0.13018900	10.35351900	1.84111600
C	-1.46009300	9.73438200	2.55959200	C	-1.17076700	9.54945800	2.35816100
C	-1.06646900	8.50654900	3.10182000	C	-0.96323800	8.15883000	2.53167300
C	0.18002900	7.96879100	2.81763700	C	0.24373200	7.58076800	2.22386400
C	1.04659400	8.66843700	1.98445900	C	1.27353000	8.37820200	1.70461200
H	-0.90891200	11.37745800	1.28329100	H	-0.29892000	11.41089900	1.67879800
H	-1.75162600	7.97999200	3.75608400	H	-1.76248500	7.55888700	2.94661600
H	0.46869100	7.01743900	3.25129100	H	0.39279700	6.52063300	2.39131100
B	-2.36980500	-10.35224800	2.00059100	B	-2.65652100	-10.14717800	1.94965800
H	10.39076900	5.44913800	-2.19464300	H	10.78898500	5.09960200	-1.76755200
C	8.73403100	0.91785900	-2.70601100	C	9.01507200	0.75997600	-3.03362600
C	10.11749600	1.16692000	-2.62920200	C	10.37211800	0.90414700	-2.68904200
C	10.53500900	2.45959800	-2.25911600	C	10.79094500	2.12176300	-2.12871300
C	9.60234700	3.43767000	-1.96514600	C	9.87279600	3.13316900	-1.89902100
C	8.22886900	3.17043700	-2.09612400	C	8.53480100	2.97521800	-2.29230800
C	7.78991000	1.90624500	-2.47529700	C	8.10046200	1.78813400	-2.87298100
H	8.39674200	-0.07845200	-2.97126200	H	8.67737500	-0.18555400	-3.44390700
H	11.59628500	2.67697900	-2.18061500	H	11.83231900	2.25518600	-1.85103700
H	6.73125800	1.68822200	-2.56887700	H	7.06522700	1.65479300	-3.16813200
C	9.80473900	4.85263600	-1.48643200	C	10.06198600	4.47361800	-1.23816000
H	10.34227300	4.88672500	-0.53236600	H	10.42673700	4.37406400	-0.21014400
C	8.39488400	5.36883900	-1.36082600	C	8.68041800	5.06843800	-1.29114500
C	7.95391500	6.60376600	-0.92343800	C	8.22591300	6.27268700	-0.79248000
C	6.57796900	6.87336600	-0.90025300	C	6.87786100	6.61016400	-0.96718800
C	5.67656000	5.88944900	-1.33575800	C	6.01887300	5.75655200	-1.67160400
C	6.12075100	4.64226800	-1.73940400	C	6.47200900	4.53742300	-2.14458700
C	7.48534000	4.37320500	-1.74647300	C	7.80229200	4.18365400	-1.94082200
H	8.65695300	7.35986400	-0.59362900	H	8.88742500	6.94604600	-0.25962900
H	4.61661300	6.11028500	-1.34506200	H	4.99308200	6.05755500	-1.84645400
H	5.40261700	3.89491500	-2.05914300	H	5.79478500	3.88013100	-2.67768100
H	2.84632700	-10.52391400	3.53391600	H	2.44997400	-10.19929100	3.73327700
C	5.07889700	-7.49706400	0.50178200	C	4.94578900	-7.84033500	0.32579900
C	5.79551900	-8.27041200	1.43671400	C	5.59244500	-8.40769700	1.45744600
C	5.11599700	-9.32530900	2.07775300	C	4.82023600	-9.32242100	2.23100900
C	3.77406100	-9.54735300	1.82951200	C	3.50189400	-9.56605300	1.93699900
C	3.07640500	-8.71713600	0.93647700	C	2.86963800	-8.92124700	0.84487500
C	3.73226900	-7.70192400	0.24942100	C	3.62489200	-8.07746500	0.01468600
H	5.59050500	-6.68851800	-0.00757200	H	5.50932600	-7.15195900	-0.29516900
H	5.64873700	-9.95244500	2.78547300	H	5.28287400	-9.81939400	3.07807000
H	3.19909400	-7.06492000	-0.44810400	H	3.16639900	-7.58756700	-0.83853000
C	2.83110100	-10.55233800	2.43939500	C	2.49261000	-10.42302300	2.66221800
H	3.09133500	-11.57757900	2.15095300	H	2.73567900	-11.48925800	2.57706600
C	1.49552000	-10.13869000	1.87712700	C	1.20031400	-10.09094100	1.95597300
C	0.22591400	-10.59732800	2.17725700	C	-0.08839800	-10.44689500	2.26932300
C	-0.90561300	-10.01409600	1.57319400	C	-1.19472600	-9.96078500	1.51431600
C	-0.69252100	-8.99142600	0.62716800	C	-0.87663900	-9.15654600	0.38603600
C	0.57250100	-8.51651100	0.32205600	C	0.41099500	-8.79726400	0.05264800
C	1.66620600	-9.08200000	0.96741700	C	1.47264300	-9.23764600	0.85851600
H	0.09414500	-11.40011100	2.89578100	H	-0.27494900	-11.09700800	3.11861100
H	-1.55150100	-8.53505200	0.14911700	H	-1.69220600	-8.76761200	-0.21372800
H	0.70009500	-7.70369400	-0.38476300	H	0.59501500	-8.14848700	-0.79799100
B	7.24184500	-7.82716000	1.82258800	B	6.98527500	-7.91969800	1.86848700
C	-10.60985100	-5.74650200	-3.84444400	C	-10.84014900	-5.39375400	-3.92731900
C	-11.18561100	-7.02753200	-3.86863100	C	-11.45759300	-6.65446500	-3.98017700
C	-11.04552700	-4.82758200	-4.81317600	C	-11.22864200	-4.45014100	-4.89259300
C	-12.16173400	-7.36844000	-4.79479700	C	-12.42327200	-6.95343100	-4.93180500
H	-10.86581300	-7.76388500	-3.13870600	H	-11.17839000	-7.40875700	-3.25194900
C	-12.00209900	-5.16907600	-5.75958800	C	-12.17612500	-4.74864400	-5.86251900
H	-10.61153500	-3.83355900	-4.83421700	H	-10.76439600	-3.46943900	-4.89074800
C	-12.56817200	-6.43915900	-5.74611100	C	-12.78138000	-6.00063900	-5.87924400
H	-12.60089500	-8.36012800	-4.78151900	H	-12.89295300	-7.93123000	-4.93997000
H	-12.30956600	-4.44550500	-6.50688800	H	-12.44551300	-4.00545600	-6.60555200
H	-13.32278900	-6.70559700	-6.47846800	H	-13.52839400	-6.23401200	-6.63054800
C	-11.10343000	5.07689600	-1.25098400	C	-11.24214900	5.37645200	-0.99387700
C	-11.62545400	4.62665000	-2.47516700	C	-11.81863500	4.98681200	-2.21440100
C	-11.69668100	6.21595100	-0.68254100	C	-11.80599400	6.48783300	-0.34624700
C	-12.69193300	5.26798900	-3.09114000	C	-12.90824600	5.65937400	-2.75168600
H	-11.17091200	3.77334900	-2.96635400	H	-11.38957700	4.15682000	-2.76482900
C	-12.78349200	6.84250500	-1.27473100	C	-12.91489300	7.14437800	-0.85977300
H	-11.29623500	6.61647200	0.24253600	H	-11.36675100	6.84259800	0.58003600
C	-13.28249300	6.36993800	-2.48393500	C	-13.46782200	6.73199900	-2.06755400
H	-13.06393000	4.90807200	-4.04434400	H	-13.32310600	5.34532700	-3.70337600
H	-13.23300200	7.70982500	-0.80343400	H	-13.34121400	7.98777400	-0.32736900
H	-14.12204000	6.86734700	-2.95772600	H	-14.32588000	7.25233100	-2.47938000
C	-2.88238500	11.57014200	3.32988700	C	-2.49963600	11.43656700	3.13914800
C	-4.03695100	12.30980400	3.04742100	C	-3.53433000	12.24836200	2.67115100
C	-1.86936100	12.17146800	4.08640100	C	-1.59085800	11.93692700	4.07468600
C	-4.17138500	13.60782000	3.51740700	C	-3.64903500	13.55104700	3.13172400
H	-4.82680500	11.86407800	2.45516800	H	-4.23796700	11.85738300	1.94635300

C	-2.00956800	13.47564000	4.53689200
H	-0.97381100	11.60986000	4.32313400
C	-3.16054600	14.20476000	4.26157900
H	-5.07491900	14.16120300	3.28486100
H	-1.21161500	13.91960500	5.12242600
H	-3.26806500	15.22138800	4.62153000
C	6.85644700	9.28998600	-0.67954400
C	7.06058100	10.21276600	0.34846100
C	7.39777600	9.54459800	-1.94143600
C	7.78686000	11.37135500	0.11271400
H	6.64615200	10.01539900	1.33023100
C	8.13793500	10.69710800	-2.16375000
H	7.23465300	8.83488000	-2.74405900
C	8.33337000	11.61876200	-1.14141100
H	7.93699000	12.07863000	0.92096700
H	8.55192600	10.88111800	-3.14905700
H	8.90587000	12.52156600	-1.32067400
C	-2.70560400	-10.91921500	3.41946400
C	-1.94231700	-10.61324400	4.55790000
C	-3.82275700	-11.75143000	3.59914300
C	-2.27424800	-11.11328500	5.80998500
H	-1.08692800	-9.95302900	4.46254400
C	-4.14377800	-12.27937600	4.84200500
H	-4.44364500	-11.99327500	2.74267700
C	-3.37049700	-11.95671900	5.95187600
H	-1.67787800	-10.84756100	6.67621700
H	-5.00218000	-12.93367100	4.94929400
H	-3.62587400	-12.35679000	6.92732700
C	7.85831100	-8.14298200	3.22558100
C	7.08036200	-8.26856600	4.38801400
C	9.24860100	-8.28776700	3.36383000
C	7.65835100	-8.53008900	5.62315000
H	6.00574500	-8.13507800	4.32577500
C	9.83110200	-8.57893400	4.58944600
H	9.88006600	-8.17690200	2.48838100
C	9.03504700	-8.69689000	5.72355400
H	7.03624700	-8.60510500	6.50857500
H	10.90600900	-8.70300200	4.66463000
H	9.48792700	-8.91093600	6.68579300
C	12.42032400	0.25571200	-3.77381100
C	13.50760500	-0.62907100	-3.68249600
C	12.56099100	1.36325100	-4.62636700
C	14.68100100	-0.41314600	-4.39155100
H	13.43018700	-1.49511100	-3.03360300
C	13.72343200	1.57546300	-5.35447800
H	11.73563100	2.05970600	-4.72891700
C	14.78822500	0.68902500	-5.23294400
H	15.51075600	-1.10487200	-4.29399100
H	13.80220900	2.43140200	-6.01606700
H	15.70083100	0.85626500	-5.79518900
H	10.19011100	-3.20336000	1.38934100
C	8.29448900	-7.25131200	-0.50725300
C	8.05212600	-6.91508300	0.83561300
C	8.45899900	-5.64853800	1.28542000
C	9.02249200	-4.74288000	0.40502000
C	9.28244200	-5.11081000	-0.92408400
C	8.93554000	-6.38045300	-1.37864800
H	7.97091900	-8.21975900	-0.87571400
H	8.28670600	-5.36566900	2.31986300
H	9.12621000	-6.67554200	-2.40520900
C	9.40407800	-3.30646700	0.63222500
H	8.55486800	-2.70968400	0.98242300
C	9.88393600	-2.87454600	-0.72565100
C	10.27295900	-1.62206500	-1.15960500
C	10.72585000	-1.43302600	-2.47658500
C	10.76262900	-2.55008800	-3.32941300
C	10.30438000	-3.79847100	-2.92641800
C	9.85559800	-3.95817100	-1.61877700
H	10.22991700	-0.77440900	-0.48221400
H	11.12534400	-2.42560800	-4.34480700
H	10.29819500	-4.63017200	-3.62317700
H	-5.62543200	7.81651800	-0.86386100
C	-9.16946300	5.64525500	1.59641700
C	-8.88360200	5.28043400	0.27185400
C	-7.69390400	5.75572500	-0.30169700
C	-6.84031300	6.56564800	0.42680300
C	-7.15647400	6.93596600	1.74259700
C	-8.32447000	6.46747000	2.33324800
H	-10.07634600	5.27710400	2.06697600
H	-7.44352800	5.49319900	-1.32615500
H	-8.57993400	6.73492000	3.35337900
C	-5.52585400	7.17499900	0.01872000
H	-4.78231700	6.41071300	-0.23409800
C	-5.12944400	7.95919700	1.24029500
C	-4.01915300	8.75800100	1.43781400
C	-3.86936000	9.43179600	2.65424400
C	-4.83715500	9.28411600	3.65522500
C	-5.95192200	8.48352800	3.45418800
C	-6.10096100	7.81485900	2.24293400
H	-3.27029200	8.88182300	0.66311400
H	-4.70455100	9.80699400	4.59529100
H	-6.68983800	8.38061600	4.24255600
H	-11.91749100	-0.73438400	-0.93981600
C	-8.05667200	-3.45163800	-1.92203900
C	-9.33988600	-3.90938600	-2.28182000
C	-10.40568000	-2.99157200	-2.22229600
C	-10.18451900	-1.68431400	-1.82767200

C	-1.71032100	13.24415300	4.52060300
H	-0.80651300	11.29494500	4.45800700
C	-2.73798600	14.05590700	4.05292600
H	-4.45229000	14.17725900	2.76133800
H	-1.00558200	13.62408700	5.25121900
H	-2.83204500	15.07465800	4.40992200
C	7.21112600	8.98648200	-0.58277600
C	7.52203600	9.75787800	0.53629600
C	7.72256800	9.32560900	-1.83427300
C	8.33457400	10.87381400	0.39635300
H	7.13610900	9.47306700	1.50816800
C	8.53786700	10.44032200	-1.96239600
H	7.47474800	8.71834100	-2.69682400
C	8.84434100	11.21753100	-0.85060100
H	8.58119900	11.46785500	1.26867400
H	8.93076600	10.70525400	-2.93700800
H	9.48316600	12.08657100	-0.95510400
C	-3.05226800	-10.61060800	3.40338000
C	-2.33649200	-10.22887500	4.55103900
C	-4.18857700	-11.41149800	3.61151600
C	-2.72115700	-10.63034500	5.82417800
H	-1.46903100	-9.58641400	4.44204100
C	-4.57023100	-11.83466100	4.87774500
H	-4.78135700	-11.71299400	2.75339800
C	-3.83628000	-11.44388300	5.99279800
H	-2.15010400	-10.30629600	6.68850400
H	-5.44541200	-12.46542900	4.99733400
H	-4.13473900	-11.76525000	6.98523000
C	7.59330400	-8.15606700	3.30200500
C	6.81750100	-8.15841100	4.47386600
C	8.97818100	-8.33426000	3.46537500
C	7.38444600	-8.34288500	5.72866400
H	5.74817300	-7.99139600	4.39958700
C	9.55150300	-8.54102100	4.71267500
H	9.61661100	-8.31594200	2.58741200
C	8.75454000	-8.54541700	5.85277200
H	6.75568500	-8.32747800	6.61322600
H	10.62292500	-8.69167700	4.79855600
H	9.19890800	-8.69803100	6.83079200
C	12.70702400	-0.10668200	-3.65705600
C	13.75483800	-1.03118800	-3.51045800
C	12.94374900	1.01096200	-4.47480900
C	14.97709100	-0.84854600	-4.14198700
H	13.60708500	-1.90049500	-2.87880400
C	14.15684700	1.19180100	-5.12391500
H	12.15393000	1.74216200	-4.61195200
C	15.17769800	0.26227600	-4.95441300
H	15.77348800	-1.57185300	-4.00435600
H	14.31036100	2.05642900	-5.76067700
H	16.12968900	0.40386000	-5.45499300
H	9.78768500	-3.20844000	1.42117100
C	8.24941400	-7.43412100	-0.39189900
C	7.83238300	-7.01629700	0.88493800
C	8.14357400	-5.70269300	1.27288500
C	8.79090200	-4.84487900	0.40359600
C	9.22240200	-5.29485100	-0.85457900
C	8.95970100	-6.60370800	-1.25028900
H	8.00863600	-8.44163700	-0.71684200
H	7.83806000	-5.35121900	2.25447800
H	9.28018400	-6.96705400	-2.22165700
C	9.11512400	-3.38604200	0.57391900
H	8.21622600	-2.78767300	0.75940400
C	9.77063200	-3.03812100	-0.73391600
C	10.21859400	-1.81620900	-1.19100300
C	10.86297100	-1.70902300	-2.43852800
C	11.00995100	-2.88060400	-3.20392200
C	10.49388500	-4.10142500	-2.78846500
C	9.86750000	-4.18190600	-1.54676300
H	10.08681700	-0.92766100	-0.58045300
H	11.50949300	-2.82175800	-4.16580400
H	10.58250600	-4.97666700	-3.42347300
H	-5.65721200	7.96569100	-0.90816300
C	-9.11058100	5.86392700	1.73657100
C	-8.92806100	5.51636200	0.38796600
C	-7.76694600	5.96404800	-0.25943200
C	-6.83515800	6.72470600	0.42444300
C	-7.04548000	7.07300000	1.76690900
C	-8.18912100	6.63885800	2.42906200
H	-9.99373000	5.51342000	2.26157100
H	-7.59646300	5.71082400	-1.30205800
H	-8.36236900	6.89012300	3.47013500
C	-5.52583200	7.28381500	-0.06076900
H	-4.84265300	6.49599900	-0.39671200
C	-5.00362900	8.00028200	1.15316100
C	-3.83499600	8.71839400	1.30380100
C	-3.57788900	9.33782300	2.53132000
C	-4.48806800	9.23552600	3.58698500
C	-5.65528100	8.50329400	3.43534400
C	-5.91563300	7.88086500	2.21653500
H	-3.13021600	8.82913600	0.48701200
H	-4.26600400	9.72577800	4.52751500
H	-6.34981000	8.42019200	4.26335300
H	-12.10533500	-0.42917600	-0.90833200
C	-8.27707100	-3.17065800	-1.94936200
C	-9.56685800	-3.60825500	-2.31354400
C	-10.62042200	-2.67714800	-2.23768600
C	-10.38321100	-1.38028000	-1.81788900

C	-8.88770600	-1.25382900	-1.50262800	C	-9.08124700	-0.97250800	-1.48386400
C	-7.81629100	-2.13945600	-1.54657100	C	-8.02023300	-1.87005700	-1.54815400
H	-7.22443200	-4.14521400	-1.96306600	H	-7.45360700	-3.87332800	-2.00597800
H	-11.40704000	-3.31757600	-2.48598500	H	-11.62568700	-2.98598300	-2.50768200
H	-6.81178900	-1.81318500	-1.29912300	H	-7.01123400	-1.56175500	-1.29558800
C	-11.15396100	-0.53884300	-1.70144300	C	-11.33847900	-0.22530100	-1.66439300
H	-11.69019500	-0.34778700	-2.63733600	H	-11.87139600	-0.00423100	-2.59562200
C	-10.26182400	0.61082300	-1.31356800	C	-10.43258800	0.90422200	-1.24777800
C	-10.57219600	1.94323900	-1.11513500	C	-10.72526300	2.23311000	-1.01017600
C	-9.56798600	2.86822700	-0.76722800	C	-9.70747300	3.13803100	-0.64057600
C	-8.25817500	2.38006200	-0.59225700	C	-8.40329200	2.62554700	-0.48328600
C	-7.92794600	1.05256800	-0.81030500	C	-8.09157100	1.30072100	-0.73528400
C	-8.93596000	0.16798700	-1.18208100	C	-9.11163100	0.43924100	-1.12997600
H	-11.59808400	2.28046600	-1.21971500	H	-11.74771700	2.58498200	-1.09858000
H	-7.47960100	3.07055900	-0.28791300	H	-7.61294100	3.29616000	-0.16535300
H	-6.90479000	0.71299200	-0.69019600	H	-7.07341600	0.94326400	-0.62494200
H	-6.23006400	-6.72274100	1.32740900	H	-6.41695600	-6.39076900	1.22430000
C	-3.54794700	-10.33244800	-0.33180200	C	-3.89529200	-10.14405700	-0.36713400
C	-3.54641300	-9.98600000	1.03113500	C	-3.83186600	-9.74263200	0.98238700
C	-4.57925200	-9.16106700	1.50650700	C	-4.83051500	-8.86461300	1.44235800
C	-5.52163100	-8.64874500	0.63252800	C	-5.78265200	-8.36077900	0.57697300
C	-5.50711800	-9.01692300	-0.72129300	C	-5.81915700	-8.77531700	-0.76497400
C	-4.53193400	-9.88677800	-1.20265500	C	-4.88137500	-9.69396000	-1.23312800
H	-2.75016000	-10.95990400	-0.71642200	H	-3.13870300	-10.82570100	-0.74317400
H	-4.60299500	-8.88379000	2.55625500	H	-4.81508000	-8.54166900	2.47939600
H	-4.51234300	-10.17834900	-2.24748200	H	-4.89793800	-10.02910300	-2.26545700
C	-6.61596500	-7.64849200	0.88745800	C	-6.84026300	-7.31900500	0.82562800
H	-7.36950600	-8.03358800	1.58450100	H	-7.58693100	-7.65778000	1.55373300
C	-7.19158800	-7.43235800	-0.48557800	C	-7.44645300	-7.13024300	-0.53872700
C	-8.14378900	-6.52371000	-0.90622400	C	-8.37944300	-6.20778600	-0.96403500
C	-8.54293300	-6.48216000	-2.25418000	C	-8.81936700	-6.19332700	-2.30344600
C	-7.95782600	-7.40118400	-3.14433300	C	-8.28844800	-7.16519600	-3.17405200
C	-6.95941200	-8.27994200	-2.74601300	C	-7.30867500	-8.06253200	-2.77301900
C	-6.56537600	-8.28310500	-1.41191700	C	-6.87060400	-8.03487600	-1.45070000
H	-8.58082900	-5.82684300	-0.19729700	H	-8.77611500	-5.47692000	-0.26541100
H	-8.26757100	-7.39406400	-4.18419000	H	-8.62308200	-7.18150000	-4.20630300
H	-6.49149300	-8.94140900	-3.46746500	H	-6.88267900	-8.76191900	-3.48485600
N	-2.73965600	10.25483200	2.86455900	N	-2.39117200	10.10026500	2.68854700
N	6.10381400	8.11644000	-0.44379000	N	6.39016900	7.83278900	-0.44632000
B	11.10944700	0.00612900	-2.96367500	B	11.34046200	-0.31095900	-2.93340300

Structure S74. The coordinates of both the optimized structures of B4N3L$_3$ at S_0 and S_1 in toluene.

S_0-optimized structure				S_1-optimized structure			
B	-9.32805700	5.57666700	-0.65667800	B	-10.65938200	3.05651400	-0.32921400
B	-10.26548100	-4.18686500	-2.73180400	B	-8.91182400	-6.56213500	-2.53118100
H	2.91916700	10.26933100	-0.37267500	H	0.12443500	10.73837700	-0.62364100
C	5.33254700	6.32110600	1.02696500	C	3.37518500	7.58504100	1.09390400
C	5.94574900	7.32817200	0.26888400	C	3.73744300	8.64643300	0.25077800
C	5.29385300	8.55682300	0.10106000	C	2.82369700	9.67753500	-0.01250600
C	4.05558400	8.75117600	0.68272300	C	1.56425900	9.61139600	0.54280900
C	3.42923200	7.72787600	1.41060400	C	1.18244200	8.51770000	1.34254400
C	4.07691000	6.50893600	1.58279100	C	2.10066400	7.50889900	1.62632600
H	5.84938400	5.37998200	1.17019300	H	4.10617500	6.82281200	1.33435300
H	5.76341400	9.33961500	-0.48378600	H	3.10933800	10.50219900	-0.65496600
H	3.61992400	5.70992200	2.15667400	H	1.83638500	6.67868600	2.27119800
C	3.17607400	9.97319700	0.65033300	C	0.42043400	10.58120800	0.41905800
H	3.66664500	10.83801800	1.11074400	H	0.68139000	11.56584100	0.82201800
C	1.96500000	9.53349200	1.43012500	C	-0.66701800	9.91439200	1.21833100
C	0.81082300	10.23733800	1.71600100	C	-1.96141100	10.33626700	1.44254000
C	-0.20404100	9.61508800	2.45332600	C	-2.82451800	9.52344000	2.19257300
C	-0.04323800	8.29026300	2.87510400	C	-2.36240900	8.29446000	2.69272000
C	1.11231500	7.58265900	2.57965700	C	-1.06177000	7.87952300	2.47365500
C	2.12702300	8.20689700	1.86181700	C	-0.20299500	8.69404000	1.73890600
H	0.67841400	11.25951600	1.37862200	H	-2.32357000	11.27413900	1.03736200
H	-0.83667000	7.82177600	3.44559900	H	-3.03916800	7.67662000	3.27025100
H	1.21795100	6.55830000	2.92033800	H	-0.72267400	6.93338400	2.88084900
B	-3.68884900	-9.82960200	2.12528700	B	-0.87615400	-10.53782400	1.85359600
H	11.18714700	3.87091900	-1.84590700	H	9.65844900	6.71907700	-2.07291900
C	8.88934600	-0.16345800	-3.20775700	C	8.66258700	1.98709900	-2.52954500
C	10.24025700	-0.18916200	-2.84616700	C	9.99611400	2.45320100	-2.42625500
C	10.81830200	0.92540400	-2.22733700	C	10.23973000	3.79775600	-2.06785600
C	10.03566900	2.03850900	-1.98115800	C	9.17212600	4.62218300	-1.81497800
C	8.67880200	2.05876900	-2.34406000	C	7.84228200	4.15591200	-1.93983500
C	8.10425800	0.95146100	-2.95762600	C	7.59882300	2.82574600	-2.30332000
H	8.45914800	-1.03585800	-3.68566800	H	8.49035000	0.95897600	-2.81902000
H	11.86364500	0.89524700	-1.93913900	H	11.25837800	4.14896100	-1.95717800
H	7.05771500	0.95060800	-3.24240800	H	6.58661000	2.45411700	-2.41126100
C	10.40411700	3.33160100	-1.30110100	C	9.16607800	6.05521200	-1.35461300
H	10.78707700	3.16725600	-0.28803300	H	9.68960900	6.17889900	-0.40119000
C	9.10328100	4.09175400	-1.29999400	C	7.69673300	6.35756100	-1.23562800
C	8.81989300	5.33192900	-0.75980300	C	7.08103500	7.50875700	-0.81414200
C	7.52376500	5.85189400	-0.87801400	C	5.66974900	7.55866600	-0.77036600
C	6.54226400	5.11012000	-1.55036200	C	4.92093600	6.43543500	-1.19703800
C	6.82245400	3.85415000	-2.06335400	C	5.54427100	5.27885000	-1.59964300
C	8.10669000	3.33614900	-1.93552500	C	6.94342800	5.22062100	-1.60889000
H	9.58309000	5.90161600	-0.24190500	H	7.66096100	8.36344400	-0.48966000
H	5.54804500	5.52609400	-1.65814700	H	3.84122100	6.49844200	-1.21730500
H	6.04187100	3.29473000	-2.56765900	H	4.95074500	4.43189800	-1.92326000
H	1.50697700	-10.52918300	3.61188800	H	4.29745400	-9.94685600	3.39815300
C	3.99882900	-7.71473000	0.57794900	C	6.18720200	-6.93541800	0.12048600
C	4.64823800	-8.58154500	1.47936900	C	7.00443800	-7.53165600	1.12327500
C	3.87030400	-9.56336600	2.12441300	C	6.41751200	-8.61713700	1.84210800
C	2.50694600	-9.63401100	1.90410900	C	5.11984300	-9.00181900	1.61965200
C	1.88529800	-8.71979100	1.03761700	C	4.31974000	-8.34384100	0.65110400
C	2.63256400	-7.76686000	0.35485200	C	4.88723500	-7.31576000	-0.12126000
H	4.58384000	-6.95713500	0.06933500	H	6.59814400	-6.11156300	-0.45320100
H	4.34654600	-10.25685400	2.81024000	H	7.01160100	-9.13738900	2.58694900
H	2.15730600	-7.06246200	-0.31929700	H	4.29779800	-6.80384300	-0.87568500
C	1.47381800	-10.54386700	2.51741800	C	4.29364100	-10.06160900	2.30899000
H	1.62316000	-11.58716000	2.21548000	H	4.67535200	-11.06878300	2.10189200
C	0.18002700	-9.98888100	1.97931700	C	2.91959200	-9.85756900	1.71668200
C	-1.12598000	-10.32578300	2.28505200	C	1.72237800	-10.43969300	2.05040600
C	-2.19973100	-9.62734500	1.69812400	C	0.51073300	-10.06650000	1.39836100
C	-1.89535300	-8.61554400	0.76554800	C	0.63128200	-9.11495700	0.34923100
C	-0.59250200	-8.25938000	0.45809700	C	1.82385900	-8.51892200	0.00344500
C	0.44575100	-8.93943100	1.08411800	C	2.98927900	-8.86379200	0.70755100
H	-1.32993500	-11.12358100	2.99224500	H	1.69021500	-11.19383200	2.83091500
H	-2.70976700	-8.07307900	0.29954400	H	-0.26890300	-8.81154700	-0.17425600
H	-0.39143800	-7.45227300	-0.23812900	H	1.85447900	-7.76910600	-0.78090300
B	6.15244400	-8.32807900	1.81686800	B	8.37141400	-6.93729900	1.46290500
C	-11.40234100	-4.43916900	-3.77491800	C	-9.98184500	-7.07961700	-3.55074800
C	-12.12468800	-5.64386900	-3.76928700	C	-10.38292300	-8.42569700	-3.54731200
C	-11.73911000	-3.49218300	-4.75602000	C	-10.57600900	-6.23575200	-4.50325000
C	-13.14546100	-5.88360700	-4.67882600	C	-11.34124300	-8.90162700	-4.43194500
H	-11.88454300	-6.39996800	-3.02908600	H	-9.93907400	-9.10620800	-2.82820200
C	-12.74109200	-3.73499300	-5.68574900	C	-11.51604300	-6.70937300	-5.40860200
H	-11.19125200	-2.55688600	-4.80027000	H	-10.28086000	-5.19249100	-4.54409000
C	-13.45199500	-4.92937600	-5.64281300	C	-11.90646300	-8.04347100	-5.36877600
H	-13.69786200	-6.81636300	-4.64277300	H	-11.64299000	-9.94295400	-4.39709500
H	-12.97064500	-2.99331700	-6.44322200	H	-11.94727300	-6.03904100	-6.14451400
H	-14.24174100	-5.11777600	-6.36227600	H	-12.64763100	-8.41447800	-6.06876100
C	-10.47433600	6.42086100	-1.30848000	C	-12.03015700	3.53232600	-0.92050600
C	-11.07967300	6.04255400	-2.51855200	C	-12.54808700	2.99204400	-2.10947100
C	-10.89889500	7.62843900	-0.73047100	C	-12.76222100	4.56780100	-0.31699100
C	-12.06634800	6.81870700	-3.11195500	C	-13.73952800	3.44831400	-2.65782500
H	-10.75323200	5.13644200	-3.01693800	H	-11.99431100	2.21705500	-2.62779400
C	-11.90684500	8.39285200	-1.29976600	C	-13.97000100	5.00613300	-0.84000000
H	-10.42781100	7.97254200	0.18398200	H	-12.37584000	5.03808300	0.58073400
C	-12.49178500	7.98953500	-2.49539600	C	-14.46073500	4.44765000	-2.01546300
H	-12.50501600	6.51043200	-4.05484100	H	-14.10716300	3.02303900	-3.58544200
H	-12.22680800	9.31217400	-0.82135800	H	-14.52269700	5.79501700	-0.34133700
H	-13.26926000	8.59299300	-2.95155700	H	-15.39711700	4.79901900	-2.43547400
C	-1.34252100	11.61301700	3.29382300	C	-4.47894100	11.25175600	2.71898300
C	-2.35591100	12.53502500	3.00814500	C	-5.68102600	11.79632700	2.25732600
C	-0.28172300	12.01362300	4.11413000	C	-3.62223700	12.05146400	3.48304100
C	-2.30799200	13.81657900	3.53639900	C	-6.01386600	13.10944500	2.55638100
H	-3.17959100	12.24271200	2.36809400	H	-6.35109200	11.18684400	1.66319500

C	-0.23660000	13.30325400	4.62277300
H	0.50464000	11.30821800	4.35399200
C	-1.24838800	14.21450600	4.34325400
H	-3.10439900	14.51428900	3.30084900
H	0.59472700	13.59115700	5.25730600
H	-1.21186500	15.21913700	4.74831800
C	8.15484800	8.15277100	-0.35998700
C	8.38184700	8.94288800	0.76951500
C	8.87672800	8.41253200	-1.52748800
C	9.30617700	9.97667500	0.72495300
H	7.82958000	8.74104200	1.67986200
C	9.81163200	9.43733100	-1.55756300
H	8.69909600	7.80756900	-2.40890800
C	10.02971400	10.22824200	-0.43521600
H	9.47091500	10.58040700	1.61068400
H	10.36289600	9.62666100	-2.47214500
H	10.75582100	11.03251700	-0.46443300
C	-4.07936000	-10.36636100	3.54140100
C	-3.28106900	-10.15778200	4.67790000
C	-5.28255600	-11.06867300	3.72104200
C	-3.66118800	-10.62782700	5.92782700
H	-2.35728500	-9.59727400	4.58296000
C	-5.65576400	-11.56639900	4.96172200
H	-5.93053700	-11.23186200	2.86610400
C	-4.84551100	-11.34244300	6.06950700
H	-3.03450100	-10.43840500	6.79267600
H	-6.58250600	-12.11962500	5.06919400
H	-5.14009300	-11.71871400	7.04338800
C	6.76063100	-8.72020100	3.20456700
C	6.00327600	-8.73180200	4.38716100
C	8.12007400	-9.05795000	3.30888800
C	6.57131500	-9.06422500	5.60977500
H	4.95557400	-8.45333800	4.34983800
C	8.68853500	-9.41981300	4.52233700
H	8.73712800	-9.04340700	2.41644100
C	7.91402700	-9.41905800	5.67729200
H	5.96780100	-9.04885900	6.51098300
H	9.73719300	-9.69263100	4.57141600
H	8.35778500	-9.68864900	6.62976700
C	12.19376100	-1.33546500	-3.80282200
C	13.14216200	-2.35445200	-3.64526200
C	12.47360900	-0.29409600	-4.69671400
C	14.32680800	-2.32930300	-4.36556300
H	12.94823800	-3.16360900	-2.95168200
C	13.66922700	-0.27456600	-5.39947800
H	11.74818700	0.49662800	-4.84319800
C	14.60559800	-1.28997600	-5.24545700
H	15.04491800	-3.13011700	-4.22484700
H	13.86145700	0.54242600	-6.08680300
H	15.53560000	-1.27218600	-5.80160500
H	9.93142700	-4.35994800	1.22074400
C	7.09800600	-7.86970100	-0.57498300
C	7.04528800	-7.54726200	0.79257000
C	7.73726100	-6.40472400	1.22889200
C	8.39091600	-5.59404300	0.31926100
C	8.44748100	-5.94844600	-1.03766600
C	7.81678500	-7.10651500	-1.48500400
H	6.55838200	-8.74155600	-0.93119900
H	7.71602900	-6.13515200	2.28070400
H	7.85194700	-7.39159100	-2.53126700
C	9.07623800	-4.27345400	0.54061300
H	8.40108700	-3.53236100	0.98189300
C	9.50690500	-3.88882000	-0.84745300
C	10.13168800	-2.73341300	-1.27602800
C	10.43720300	-2.58500200	-2.63114200
C	10.14233100	-3.61427000	-3.53233500
C	9.50875500	-4.77158400	-3.10269000
C	9.17022000	-4.90222800	-1.75865300
H	10.37667500	-1.93405800	-0.58520600
H	10.39422600	-3.48475200	-4.57867200
H	9.26268900	-5.54909700	-3.81778900
H	-4.66011900	8.41409800	-0.96811400
C	-8.43559400	6.70658600	1.51367700
C	-8.22146400	6.32661900	0.17988900
C	-6.98691100	6.64489000	-0.40742200
C	-6.01909400	7.31699300	0.31855200
C	-6.26137200	7.70316700	1.64540200
C	-7.47479000	7.39219900	2.24823400
H	-9.37739900	6.45827800	1.99404600
H	-6.78984500	6.36676000	-1.43936000
H	-7.67586700	7.67480700	3.27639600
C	-4.63808600	7.74430900	-0.10126500
H	-4.01400800	6.88920300	-0.38439100
C	-4.11551100	8.43624500	1.12876200
C	-2.89748300	9.05903400	1.32518700
C	-2.63396500	9.67193100	2.55460400
C	-3.60039700	9.64505100	3.56712500
C	-4.82360300	9.02271800	3.36660700
C	-5.08429400	8.41156000	2.14420600
H	-2.14827400	9.09003400	0.54160800
H	-3.37991200	10.11910800	4.51646000
H	-5.55828400	9.01064100	4.16453600
H	-12.02382000	0.75995300	-0.95026300
C	-8.55677300	-2.44336000	-1.93421000
C	-9.89369700	-2.73924100	-2.26704800
C	-10.83249800	-1.69155100	-2.21194600
C	-10.43973400	-0.41671600	-1.84511700
C	-3.95828100	13.36762400	3.76171000
H	-2.69766600	11.63420700	3.86380300
C	-5.15562900	13.90649100	3.30441000
H	-6.95077300	13.51383100	2.18937400
H	-3.28303400	13.97133000	4.35820600
H	-5.41784900	14.93320700	3.53169700
C	5.66675600	9.96180100	-0.41762100
C	5.77030200	10.77519700	0.70962000
C	6.17265700	10.39304900	-1.64329200
C	6.38236800	12.01571100	0.60678300
H	5.37639200	10.42887800	1.65774600
C	6.78889700	11.63274700	-1.73402000
H	6.07202500	9.75981600	-2.51703700
C	6.89445400	12.44714600	-0.61202400
H	6.46576800	12.64416500	1.48577900
H	7.17620800	11.96816000	-2.68905700
H	7.37232000	13.41680600	-0.68793500
C	-1.13593500	-11.19531300	3.26083800
C	-0.42985800	-10.82511700	4.41812000
C	-2.13629000	-12.16982300	3.42005400
C	-0.69572300	-11.39896500	5.65492700
H	0.33106700	-10.05505700	4.34772700
C	-2.39589300	-12.76402600	4.64778100
H	-2.71884500	-12.46982700	2.55449300
C	-1.67523800	-12.37888400	5.77343900
H	-0.13840100	-11.07997100	6.52994700
H	-3.16626300	-13.52409900	4.73011800
H	-1.88042200	-12.83394000	6.73682500
C	9.16556900	-7.27887200	2.78002500
C	8.54040500	-7.51674700	4.01666100
C	10.57130300	-7.31981400	2.77291100
C	9.26797400	-7.79253800	5.16746400
H	7.45879600	-7.46363800	4.08017100
C	11.30694300	-7.61273400	3.91329100
H	11.09675700	-7.12324700	1.84317800
C	10.65655500	-7.85151600	5.11956900
H	8.74978700	-7.96083400	6.10628100
H	12.39063800	-7.65108100	3.86404300
H	11.22685300	-8.07456600	6.01524900
C	12.27834900	1.98105100	-3.17247400
C	13.45293300	1.38238600	-2.71007600
C	12.34667600	2.95547400	-4.17299300
C	14.67709700	1.76612100	-3.23517400
H	13.39981600	0.62426200	-1.93835200
C	13.57778100	3.33748800	-4.68264200
H	11.43485400	3.39457500	-4.55986300
C	14.74797900	2.74708800	-4.21800600
H	15.58252700	1.29795700	-2.86671300
H	13.61980100	4.08894900	-5.46286900
H	15.70750600	3.04372700	-4.62479300
H	10.75411700	-1.95678900	1.35307600
C	9.22933800	-5.99665400	-0.84639800
C	8.99973500	-5.82098100	0.53222600
C	9.27986900	-4.55755200	1.07965000
C	9.70026400	-3.51682900	0.27340900
C	9.92493600	-3.72097600	-1.09763500
C	9.70549200	-4.97674400	-1.65884500
H	9.01795400	-6.96451100	-1.28983700
H	9.12072100	-4.39213700	2.14136000
H	9.87529800	-5.15352700	-2.71625300
C	9.93751600	-2.07501600	0.63140900
H	9.05343600	-1.61190700	1.08263100
C	10.28272800	-1.45943300	-0.69524400
C	10.52823700	-0.13817600	-1.00479200
C	10.83859800	0.19919000	-2.32579000
C	10.92769900	-0.78376300	-3.31303500
C	10.65895400	-2.10931500	-3.00323200
C	10.31574800	-2.44944500	-1.69536900
H	10.48494800	0.63874400	-0.24884600
H	11.17727900	-0.49536700	-4.32774400
H	10.69924100	-2.86332600	-3.78115700
H	-6.91042700	6.97155000	-1.01354100
C	-10.01974500	4.50892900	1.74361400
C	-9.76045500	4.11328900	0.42200700
C	-8.67322300	4.70236300	-0.24173400
C	-7.89202100	5.64872700	0.39784600
C	-8.18242700	6.04456600	1.71170300
C	-9.24853000	5.46666400	2.39152500
H	-10.84396000	4.05341700	2.28409800
H	-8.44495200	4.41796200	-1.26535200
H	-9.48025400	5.75144800	3.41254500
C	-6.68573200	6.38895500	-0.11323400
H	-5.86993100	5.70716800	-0.37851700
C	-6.32246900	7.27253200	1.04860000
C	-5.30477600	8.20145600	1.15082700
C	-5.17894800	8.94532700	2.32748500
C	-6.07252800	8.74624800	3.38400300
C	-7.09245500	7.81097400	3.28100900
C	-7.21785800	7.06796700	2.11089500
H	-4.61431600	8.37450100	0.33239500
H	-5.95724300	9.33323500	4.28788200
H	-7.77597000	7.66465700	4.11014300
H	-11.90976200	-2.32761400	-0.56240100
C	-7.72633000	-4.42260600	-1.73410800
C	-8.93862100	-5.07682000	-2.03072100
C	-10.12971300	-4.33529400	-1.91806000
C	-10.09590600	-3.00663200	-1.53369300

C	-9.09349400	-0.15117000	-1.54577700	C	-8.86775200	-2.37849100	-1.27073500
C	-8.14446000	-1.16651500	-1.58832100	C	-7.67512200	-3.08720800	-1.36742900
H	-7.82111400	-3.23873100	-1.97211800	H	-6.79961500	-4.97874100	-1.81812200
H	-11.87149400	-1.89148400	-2.45497500	H	-11.07943000	-4.81454100	-2.13481500
H	-7.10280000	-0.96709800	-1.36065100	H	-6.72266600	-2.60772000	-1.16819500
C	-11.25207600	0.84585600	-1.72379700	C	-11.22414400	-2.02365200	-1.36170900
H	-11.77232300	1.09222100	-2.65581400	H	-11.82888800	-1.92673200	-2.26987700
C	-10.21422600	1.87637700	-1.36353900	C	-10.50334900	-0.74594600	-1.02022400
C	-10.34803800	3.23932400	-1.17386200	C	-11.00496500	0.52490200	-0.81240600
C	-9.22815400	4.03015600	-0.84876000	C	-10.13965000	1.59838900	-0.51862400
C	-7.98876100	3.37954300	-0.68996600	C	-8.76385100	1.31689600	-0.40174400
C	-7.83479400	2.01916900	-0.89961100	C	-8.24425800	0.05324200	-0.62795800
C	-8.95414500	1.26859000	-1.24590800	C	-9.12011800	-0.97915400	-0.95030200
H	-11.32383400	3.70463200	-1.26554100	H	-12.07433500	0.69914900	-0.86815900
H	-7.12348000	3.96670300	-0.40368000	H	-8.08646400	2.12101900	-0.13754700
H	-6.86199700	1.55189000	-0.79077700	H	-7.17692300	-0.12503100	-0.55317400
H	-7.14393200	-5.82070400	1.40021900	H	-5.17466300	-7.32683600	1.34483100
C	-4.86350900	-9.71772000	-0.20533500	C	-2.19457000	-10.64825000	-0.40956100
C	-4.82308300	-9.35357900	1.15220700	C	-2.13748400	-10.26532000	0.94395000
C	-5.76191300	-8.41738300	1.61584400	C	-3.22777500	-9.54552600	1.46247500
C	-6.64698800	-7.82183100	0.73459300	C	-4.28051100	-9.17430100	0.64592500
C	-6.67558200	-8.21080900	-0.61320100	C	-4.31371000	-9.57516300	-0.69929100
C	-5.79809800	-9.18510600	-1.08195000	C	-3.27266900	-10.33522200	-1.22593400
H	-4.13599000	-10.42997300	-0.58155200	H	-1.36055600	-11.20159200	-0.82985300
H	-5.75373300	-8.12306300	2.66123100	H	-3.21603100	-9.24191400	2.50550200
H	-5.81134600	-9.49287300	-2.12219500	H	-3.28438000	-10.65254000	-2.26389200
C	-7.62742800	-6.70637000	0.97442800	C	-5.47132500	-8.30750300	0.95729100
H	-8.41685300	-6.99791000	1.67714300	H	-6.12030300	-8.75846400	1.71731200
C	-8.17932000	-6.45095100	-0.40157200	C	-6.16651700	-8.20778300	-0.37388300
C	-9.02601200	-5.44956000	-0.83667100	C	-7.25815300	-7.44694200	-0.74109300
C	-9.42185100	-5.38655800	-2.18472300	C	-7.76112100	-7.50163300	-2.05589000
C	-8.94592800	-6.38006900	-3.05951300	C	-7.12305000	-8.36573600	-2.96579200
C	-8.04826200	-7.35620000	-2.64757000	C	-5.98989700	-9.09154200	-2.62532800
C	-7.65195500	-7.37975300	-1.31438900	C	-5.49991000	-9.00100300	-1.32541800
H	-9.38002500	-4.69595600	-0.13954600	H	-7.73352700	-6.79397400	-0.01492400
H	-9.25657900	-6.35662100	-4.09891800	H	-7.50468100	-8.43346400	-3.97940900
H	-7.65751200	-8.07663100	-3.35841900	H	-5.49195500	-9.70909900	-3.36552100
N	-1.39168700	10.31175600	2.76830400	N	-4.14815500	9.91379300	2.43592600
N	7.20682000	7.10680300	-0.32182600	N	5.02605200	8.69609400	-0.31880600
N	10.99692800	-1.36313300	-3.08030400	N	11.03264400	1.57064900	-2.65566800

Structure S75. The coordinates of both the optimized structures of B3N4L$_3$ at S$_0$ and S$_1$ in toluene.

S_0-optimized structure				S_1-optimized structure			
B	-10.26109800	3.92488600	-0.63846200	B	-11.22139000	1.65759300	-0.16267300
B	-9.32897300	-5.84435700	-2.68403300	B	-7.93828800	-7.48603200	-2.52772700
H	0.97266200	10.62244100	-0.42624600	H	-1.24229800	10.84914900	-0.46416100
C	4.02299600	7.13190900	0.94922500	C	2.22616400	7.72089500	0.78324200
C	4.45450400	8.22647400	0.18705500	C	2.52173700	8.93925900	0.12613100
C	3.60436100	9.32822500	0.02315400	C	1.52329200	9.93218100	0.02980500
C	2.35612200	9.31431000	0.61552100	C	0.27706200	9.68153700	0.54727200
C	1.91476500	8.20196400	1.34842000	C	-0.02639100	8.44596200	1.16236600
C	2.75773300	7.10819900	1.51440200	C	0.97094200	7.47104400	1.28638300
H	4.69074500	6.29073600	1.08986500	H	3.00798100	6.98256600	0.90138300
H	3.93146700	10.17790500	-0.56528000	H	1.73850600	10.86621100	-0.47364700
H	2.44548400	6.24457800	2.09176000	H	0.77111200	6.53146400	1.78835300
C	1.28643000	10.37418700	0.59365900	C	-0.93955700	10.56547300	0.54903400
H	1.63250100	11.30879400	1.04891000	H	-0.76439800	11.49675600	1.09775300
C	0.17328500	9.74140700	1.38659200	C	-1.97240300	9.70224300	1.21964800
C	-1.07730000	10.24588500	1.68798000	C	-3.28889800	9.97951600	1.48494300
C	-1.96743000	9.46435400	2.43454400	C	-4.09229900	8.98292400	2.08059900
C	-1.58678700	8.18333400	2.85022000	C	-3.52737000	7.72572000	2.40125900
C	-0.33406300	7.67561300	2.54013100	C	-2.20087700	7.46311700	2.15322400
C	0.55605500	8.45928000	1.81333800	C	-1.40585000	8.45013300	1.55634800
H	-1.38032300	11.23279900	1.35564600	H	-3.72721400	10.93185600	1.21324000
H	-2.28637100	7.59016000	3.42749000	H	-4.14905700	6.97907800	2.87798800
H	-0.05760200	6.68199900	2.87610700	H	-1.78140600	6.50308000	2.43013300
B	-1.78425400	-10.18381300	2.12971700	B	0.57370900	-10.44606700	1.74322000
H	10.23152600	5.68796700	-1.80679900	H	8.79373800	7.75074800	-1.52976700
C	8.68782600	1.30844900	-3.15936200	C	8.40928200	3.08485100	-2.76429600
C	10.01449600	1.51839500	-2.76858900	C	9.63835300	3.63525800	-2.36986300
C	10.37836200	2.72110900	-2.15230700	C	9.67440700	4.91078600	-1.78978500
C	9.41136400	3.68560700	-1.93734200	C	8.49221300	5.60470200	-1.61972800
C	8.07930700	3.46961900	-2.32725800	C	7.26585500	5.05271400	-2.02585800
C	7.71777500	2.27418000	-2.93889200	C	7.22727900	3.78383900	-2.59652100
H	8.42544300	0.37210800	-3.63793500	H	8.39748800	2.09888400	-3.21276000
H	11.40656900	2.87446500	-1.84270500	H	10.62205800	5.32821800	-1.46867300
H	6.69394200	2.09155100	-3.24680700	H	6.28979300	3.34196700	-2.91563300
C	9.53987800	5.02956300	-1.26930100	C	8.26653900	6.95495400	-0.99226900
H	9.92313800	4.94413600	-0.24665400	H	8.62126200	6.98976300	0.04337600
C	8.12927900	5.55665200	-1.30485900	C	6.77301900	7.11895000	-1.08492800
C	7.62872900	6.73701500	-0.78925700	C	5.98173000	8.15882700	-0.64323900
C	6.26619500	7.02773100	-0.93867100	C	4.59725700	8.09562500	-0.85218400
C	5.43799400	6.12141800	-1.61529700	C	4.03747500	6.99804700	-1.52051800
C	5.93764400	4.92533100	-2.10472300	C	4.83181000	5.94386100	-1.93735200
C	7.28880100	4.63503500	-1.94730500	C	6.20497300	5.99666900	-1.71366600
H	8.27339600	7.43475500	-0.26687300	H	6.41278600	9.01355200	-0.13512600
H	4.38970400	6.36097200	-1.74585500	H	2.97245900	6.98137200	-1.71696500
H	5.27352000	4.23524700	-2.61392900	H	4.38092900	5.10092800	-2.44864900
H	3.48901600	-10.04653100	3.55433800	H	5.77844500	-9.45146000	3.10048400
C	5.44230600	-6.74730000	0.63115400	C	6.96673500	-5.54400700	0.53661300
C	6.22941800	-7.57639300	1.44823000	C	7.92427100	-6.28933000	1.24294600
C	5.64080000	-8.70284700	2.04748200	C	7.58253200	-7.56475700	1.72177200
C	4.29101000	-8.93709100	1.87152500	C	6.29555000	-8.03380600	1.54189000
C	3.50761500	-8.08974200	1.07282200	C	5.33778200	-7.27130100	0.85549200
C	4.09589400	-7.00328500	0.43752600	C	5.68624700	-6.03153100	0.33577600
H	5.89108300	-5.88203400	0.16161600	H	7.22664100	-4.56367100	0.15962600
H	6.24218300	-9.36527000	2.65885800	H	8.32116000	-8.15980500	2.24612500
H	3.50957400	-6.33604300	-0.18510300	H	4.96213200	-5.42711500	-0.19987300
C	3.42985700	-10.02467300	2.46101400	C	5.67651600	-9.32332700	2.01760400
H	3.73567900	-11.01808300	2.11291300	H	6.15208700	-10.19596000	1.55507800
C	2.05038500	-9.66166200	1.97117600	C	4.23632000	-9.18059300	1.59230600
C	0.82684500	-10.22891700	2.26883600	C	3.15273700	-10.00117300	1.84157600
C	-0.36061600	-9.70079400	1.71992000	C	1.86860500	-9.65411700	1.37389600
C	-0.24333300	-8.60855600	0.83760500	C	1.74451400	-8.46799400	0.62424500
C	0.97510500	-8.01685900	0.54540600	C	2.81845600	-7.62590800	0.38245500
C	2.12486900	-8.53742100	1.12924200	C	4.06637800	-7.97895700	0.88277100
H	0.77238800	-11.08389400	2.93577400	H	3.28423100	-10.91767000	2.40877400
H	-1.14374600	-8.19439200	0.39824100	H	0.76601000	-8.18756900	0.25146800
H	1.02426200	-7.15527400	-0.11196300	H	2.67900300	-6.70350500	-0.17132300
C	-10.39598400	-6.30582700	-3.72969300	C	-8.86554200	-8.15716700	-3.60932100
C	-10.87599900	-7.62590800	-3.72734300	C	-9.02593600	-9.55262600	-3.65434300
C	-10.90605400	-5.43808100	-4.70923400	C	-9.54197600	-7.41476300	-4.59207100
C	-11.83242300	-8.05314300	-4.63812300	C	-9.83385700	-10.16997500	-4.60008600
H	-10.49642100	-8.32424300	-2.98874500	H	-8.50700300	-10.16437800	-2.92288600
C	-11.84333000	-5.86470700	-5.64026100	C	-10.33718100	-8.02400600	-5.55435700
H	-10.54581400	-4.41568800	-4.75136000	H	-9.42547400	-6.33619300	-4.61008400
C	-12.31446300	-7.17244400	-5.60030400	C	-10.49316900	-9.40586400	-5.55710700
H	-12.19729200	-9.07402700	-4.60441700	H	-9.94570900	-11.24955100	-4.59588000
H	-12.20945400	-5.17836500	-6.39623700	H	-10.83613200	-7.41988200	-6.30541000
H	-13.05367800	-7.50616600	-6.32070100	H	-11.11893900	-9.88434800	-6.30329000
C	-11.54224600	4.53660700	-1.29842000	C	-12.64179800	1.97351400	-0.77235500
C	-12.05938600	4.04683500	-2.50935100	C	-13.02464700	1.47564200	-2.03124100
C	-12.18735600	5.64655800	-0.72897900	C	-13.56550400	2.82112000	-0.13569900
C	-13.16944900	4.62348000	-3.11213900	C	-14.25311300	1.77900900	-2.60388700
H	-11.56688400	3.21527100	-3.00125400	H	-12.33039200	0.85264900	-2.58468100
C	-13.31635300	6.20772500	-1.30766100	C	-14.80362000	3.11493000	-0.69029800
H	-11.79396600	6.07639600	0.18594600	H	-13.30970200	3.26231200	0.82179900
C	-13.80873700	5.69756300	-2.50423000	C	-15.15668500	2.59316800	-1.93035300
H	-13.53734500	4.23509000	-4.05570500	H	-14.50502100	1.38017800	-3.58147200
H	-13.80457100	7.05365100	-0.83599100	H	-15.49426600	3.76065900	-0.15702600
H	-14.68180800	6.14432800	-2.96778700	H	-16.12029700	2.82676900	-2.37068600
C	-3.40612900	11.24428600	3.30225100	C	-5.88428300	10.52203100	2.66672500
C	-4.55351800	11.99687900	3.02716100	C	-7.06088200	11.00465600	2.09048600
C	-2.41888200	11.80029300	4.12369700	C	-5.17485000	11.31440000	3.57240500
C	-4.70689600	13.26515700	3.56726300	C	-7.51573500	12.27311400	2.41575800
H	-5.32267500	11.58368400	2.38586400	H	-7.60578500	10.38746000	1.38658000
C	-2.57645700	13.07616200	4.64446200	C	-5.63592300	12.58410700	3.88407000

H	-1.53004800	11.22578500	4.35501400
C	-3.72070100	13.81829700	4.37573400
H	-5.60427200	13.83041900	3.33972800
H	-1.79896300	13.48637500	5.27995600
H	-3.84212500	14.81231300	4.79033900
C	6.48651000	9.41159800	-0.46812600
C	6.57049400	10.25373200	0.64342900
C	7.16026800	9.76428600	-1.64008200
C	7.30600300	11.42833100	0.57628800
H	6.05607500	9.98194800	1.55770400
C	7.90831600	10.93166500	-1.69213600
H	7.09290600	9.11840000	-2.50769600
C	7.98281600	11.77319300	-0.58806800
H	7.36067200	12.07099000	1.44818000
H	8.42499500	11.19088600	-2.60977300
H	8.56214500	12.68813600	-0.63437000
C	-2.07149600	-10.84188400	3.52010200
C	-1.31608500	-10.54563100	4.66632100
C	-3.13300500	-11.74980500	3.66741200
C	-1.60187000	-11.12685000	5.89450800
H	-0.50389000	-9.83007900	4.59625400
C	-3.40816400	-12.35599100	4.88535000
H	-3.74644200	-11.98831300	2.80468700
C	-2.64358400	-12.04135600	6.00354300
H	-1.01254000	-10.86841700	6.76778500
H	-4.22413300	-13.06598700	4.96721400
H	-2.86324700	-12.50367400	6.96005100
C	8.24558900	-7.57736000	2.86507900
C	7.59292000	-7.51660700	4.09958400
C	9.60448000	-7.90123800	2.83424000
C	8.28467800	-7.79501200	5.27011800
H	6.54453200	-7.24690100	4.14040800
C	10.29123500	-8.16220100	4.01080700
H	10.11708000	-7.94537600	1.88037200
C	9.63608700	-8.11792700	5.23591300
H	7.76186600	-7.74219200	6.21893000
H	11.34553800	-8.41227500	3.96577300
H	10.17289600	-8.32955800	6.15347500
C	12.17887000	0.75330400	-3.63404600
C	13.31789200	-0.01396500	-3.36138700
C	12.27526600	1.78576700	-4.57452100
C	14.51129500	0.24297700	-4.01947800
H	13.26419700	-0.80986200	-2.62846000
C	13.47915300	2.04237300	-5.21375700
H	11.40133600	2.38239500	-4.80590500
C	14.60614800	1.27389500	-4.94702000
H	15.38027000	-0.36467900	-3.79081300
H	13.52889000	2.84698500	-5.93957700
H	15.54282900	1.47477500	-5.45385800
H	10.02536600	-2.50558400	1.33853300
C	8.56270200	-6.73367900	-0.51858200
C	8.21988300	-6.35229600	0.77956800
C	8.45436300	-5.04667800	1.21586300
C	9.00820500	-4.13499800	0.33811300
C	9.35142600	-4.51528100	-0.97002900
C	9.13319300	-5.82153200	-1.39746800
H	8.36672500	-7.75248900	-0.83280200
H	8.17375800	-4.76240600	2.22442400
H	9.39313500	-6.12987500	-2.40423900
C	9.28753500	-2.67219200	0.54556600
H	8.38705400	-2.12043200	0.83608600
C	9.80541700	-2.24178000	-0.79877000
C	10.18505600	-0.98192600	-1.21827700
C	10.64089600	-0.80569900	-2.52828100
C	10.73016100	-1.90685000	-3.38865600
C	10.34874000	-3.17171400	-2.96518400
C	9.86927300	-3.34071200	-1.66952800
H	10.12851100	-0.12685300	-0.55371900
H	11.08602900	-1.75611400	-4.40121200
H	10.40657500	-4.00873900	-3.65261600
H	-6.17220000	7.55118900	-0.96994300
C	-9.59000300	5.21830600	1.52198600
C	-9.31231700	4.87291500	0.19065200
C	-8.15447800	5.40165900	-0.40119400
C	-7.31908100	6.23702700	0.31940100
C	-7.62338600	6.58068600	1.64520900
C	-8.76390500	6.06628400	2.25103200
H	-10.47259100	4.81029200	2.00568500
H	-7.91180200	5.15547900	-1.43155400
H	-9.01015000	6.31448400	3.27827500
C	-6.03281500	6.89425000	-0.10413800
H	-5.27245900	6.15903100	-0.39047400
C	-5.63276200	7.66481700	1.12492800
C	-4.53396100	8.47956000	1.32078300
C	-4.37312500	9.12614100	2.55070100
C	-5.32224900	8.94153800	3.56328800
C	-6.42662200	8.12660500	3.36302200
C	-6.58328500	7.47987400	2.14110600
H	-3.80030200	8.63276700	0.53688900
H	-5.18191200	9.44419100	4.51313900
H	-7.14859200	7.99289000	4.16145100
H	-12.00353600	-1.31651400	-0.91807000
C	-7.98741800	-3.81038800	-1.87370500
C	-9.24218400	-4.35391300	-2.21405700
C	-10.36278200	-3.50292900	-2.16565400
C	-10.22057700	-2.17637100	-1.79958500
C	-8.95061500	-1.66103500	-1.49298600

H	-4.27794400	10.92468000	4.03919000
C	-6.80599600	13.06828500	3.30907400
H	-8.42669800	12.64441400	1.96108600
H	-5.08603700	13.19143200	4.59370700
H	-7.16600200	14.05904400	3.56016000
C	4.26186100	10.48549500	-0.57134400
C	4.28371900	11.35444500	0.51905000
C	4.73265700	10.90964800	-1.81310000
C	4.76600800	12.64578800	0.36059400
H	3.93070200	11.01107100	1.48449300
C	5.22027700	12.20008700	-1.95952100
H	4.70998800	10.22798900	-2.65521800
C	5.23656100	13.07194900	-0.87633100
H	4.78563700	13.31684100	1.21164400
H	5.58175000	12.52763700	-2.92739500
H	5.61769400	14.07947900	-0.99508800
C	0.46563500	-11.26214800	3.07580900
C	1.17986300	-10.91432100	4.23382200
C	-0.38251400	-12.37877900	3.15665700
C	1.05559700	-11.64117800	5.41038700
H	1.82855000	-10.04500400	4.21536600
C	-0.49343800	-13.12597900	4.32136300
H	-0.95876300	-12.66676100	2.28355200
C	0.22389800	-12.75472100	5.45328400
H	1.60691100	-11.34087700	6.29514800
H	-1.14567200	-13.99222600	4.35107000
H	0.13086700	-13.32912200	6.36892900
C	9.93507600	-6.01873600	2.64277700
C	9.30493300	-6.20960300	3.87588700
C	11.33130800	-6.04664500	2.58643200
C	10.05926100	-6.44254400	5.01735500
H	8.22417400	-6.17264500	3.93888000
C	12.07681000	-6.26410100	3.73562900
H	11.82625300	-5.89754600	1.63382800
C	11.44752500	-6.47034200	4.95774700
H	9.55254000	-6.58879500	5.96511300
H	13.15917300	-6.28377000	3.67022200
H	12.03165400	-6.64899300	5.85312300
C	11.99841900	3.45210900	-3.02873300
C	13.24169000	2.94391600	-2.63727500
C	11.96095200	4.52312600	-3.92899000
C	14.41242100	3.49417000	-3.13796600
H	13.28601300	2.11753000	-1.93828500
C	13.13909600	5.07457100	-4.40964200
H	11.00582000	4.91472900	-4.25748100
C	14.37335200	4.56547600	-4.02230700
H	15.36538900	3.08451100	-2.82139100
H	13.08752000	5.90233500	-5.10863600
H	15.29083800	4.99536600	-4.40680800
H	10.64376200	-0.58091700	1.52712500
C	10.00051300	-4.85502600	-0.66429000
C	9.62810700	-4.65800500	0.66682700
C	9.61367200	-3.37246400	1.21179400
C	9.94736800	-2.29949600	0.40802600
C	10.32024300	-2.49516800	-0.93177000
C	10.35375700	-3.77885400	-1.46798100
H	9.99873100	-5.86302100	-1.06281000
H	9.31108800	-3.23387400	2.24418300
H	10.63881300	-3.94566500	-2.50093000
C	9.92888600	-0.83229400	0.73546400
H	8.94510600	-0.50349000	1.08718200
C	10.30336300	-0.19816600	-0.57495400
C	10.39821100	1.14207100	-0.89575700
C	10.77048700	1.50675100	-2.19193300
C	11.06748000	0.52355100	-3.14071900
C	10.96651200	-0.82278800	-2.81910400
C	10.56714600	-1.18676400	-1.53626500
H	10.18501900	1.91423300	-0.16460800
H	11.35985900	0.82709700	-4.13935200
H	11.17864300	-1.57417900	-3.57169400
H	-7.75796500	5.81291200	-0.97880000
C	-10.82667800	3.35307300	1.82262900
C	-10.46365400	2.85737700	0.55528400
C	-9.38368700	3.47629600	-0.09606100
C	-8.71663500	4.53592500	0.49045200
C	-9.11440800	5.02190800	1.74622600
C	-10.17558700	4.42252700	2.42019500
H	-11.63678000	2.87369400	2.36290300
H	-9.07192800	3.11274600	-1.07090300
H	-10.48569900	4.77480200	3.39883500
C	-7.54123400	5.32208300	-0.02359300
H	-6.66410300	4.68724800	-0.19092200
C	-7.30812700	6.32248200	1.07392900
C	-6.36624500	7.32499400	1.15182700
C	-6.36945500	8.17148900	2.26874900
C	-7.31451500	8.00215500	3.28757700
C	-8.25154400	6.98591000	3.21084900
C	-8.25312100	6.13916900	2.10239300
H	-5.64625500	7.48532500	0.35689500
H	-7.29574800	8.66623000	4.14364700
H	-8.97155800	6.85505600	4.01046700
H	-11.75593500	-3.79586600	-0.92979700
C	-7.20654300	-5.34062600	-1.31921200
C	-8.23914500	-6.10412500	-1.92867600
C	-9.51701900	-5.47772400	-2.00944000
C	-9.71694300	-4.20095500	-1.54764100
C	-8.65252100	-3.45642500	-0.97746200

C	-7.82644900	-2.47857100	-1.52700300	C	-7.38713200	-4.05517200	-0.85808100
H	-7.11426500	-4.45203100	-1.90660000	H	-6.21764600	-5.78028200	-1.24741600
H	-11.34357000	-3.89622400	-2.41447100	H	-10.34756700	-6.02973100	-2.43926400
H	-6.84295600	-2.08520700	-1.29318700	H	-6.55540000	-3.50641500	-0.42728400
C	-11.25718100	-1.08913700	-1.68779900	C	-10.96822700	-3.35606500	-1.55298700
H	-11.80868600	-0.94810900	-2.62369400	H	-11.39124000	-3.25176500	-2.55811800
C	-10.43434100	0.11980700	-1.32637000	C	-10.48938500	-2.03640800	-0.99623100
C	-10.82295900	1.43424900	-1.14522700	C	-11.16848600	-0.85758800	-0.83073100
C	-9.87350300	2.42279300	-0.81843900	C	-10.52396900	0.30872700	-0.31189100
C	-8.53522500	2.01683800	-0.64809300	C	-9.16543400	0.13747600	0.08748100
C	-8.12721600	0.70873900	-0.84894700	C	-8.47020900	-1.03794800	-0.07370000
C	-9.08329300	-0.23982600	-1.19810800	C	-9.11949700	-2.14816400	-0.64375600
H	-11.86815300	1.70758800	-1.24531100	H	-12.22028300	-0.80618800	-1.09265700
H	-7.79749000	2.75706900	-0.35990300	H	-8.64977200	0.98395800	0.52859800
H	-7.08476400	0.43279900	-0.73194300	H	-7.43005700	-1.10510000	0.23038900
H	-5.94572600	-6.87593000	1.43991100	H	-4.30652400	-8.23352300	1.47532100
C	-2.95172100	-10.23826200	-0.20061400	C	-0.58097300	-10.43234900	-0.58650600
C	-2.98585300	-9.89382600	1.16209100	C	-0.67762700	-10.32423200	0.81277400
C	-4.08745000	-9.16139000	1.63353600	C	-1.92499400	-9.97565100	1.36190400
C	-5.06937500	-8.73582700	0.75609800	C	-2.99542900	-9.68146800	0.53746000
C	-5.02087300	-9.10651600	-0.59637700	C	-2.87607200	-9.81068200	-0.85618400
C	-3.96990100	-9.88434300	-1.07453700	C	-1.66847000	-10.21559900	-1.42020900
H	-2.09916800	-10.79102100	-0.58227800	H	0.37710700	-10.68844500	-1.02806000
H	-4.13869500	-8.88652000	2.68307400	H	-2.02801800	-9.88593200	2.43954200
H	-3.92147800	-10.17422200	-2.11892300	H	-1.56419300	-10.31875300	-2.49533500
C	-6.24821800	-7.83467900	1.00534000	C	-4.36199200	-9.15826600	0.89083000
H	-6.96546200	-8.28045100	1.70451100	H	-4.93115600	-9.87389900	1.49598700
C	-6.84044300	-7.67778400	-0.36893000	C	-4.99546500	-8.94500200	-0.45843900
C	-7.86751700	-6.85788500	-0.79549800	C	-6.21142300	-8.36788500	-0.77788400
C	-8.26951700	-6.86147200	-2.14333000	C	-6.62015500	-8.24882300	-2.11839900
C	-7.60897600	-7.73423600	-3.02688200	C	-5.76557200	-8.77463800	-3.10626400
C	-6.53601000	-8.51798600	-2.62347800	C	-4.52353900	-9.31534500	-2.80509700
C	-6.14132000	-8.47552600	-1.29034700	C	-4.12872400	-9.38396200	-1.47185500
H	-8.36180000	-6.19477600	-0.09172600	H	-6.85509100	-7.98502600	0.00878100
H	-7.91972500	-7.76290200	-4.06614600	H	-6.07482800	-8.71812500	-4.14525900
H	-6.01199000	-9.14094300	-3.34067600	H	-3.87053200	-9.66498800	-3.59850600
N	-3.24984900	9.95653600	2.76463900	N	-5.42635000	9.22173900	2.34991100
N	5.73165700	8.21950700	-0.40920100	N	3.77645900	9.15826400	-0.41400700
N	10.96927500	0.49402100	-2.97607900	N	10.81366000	2.88190500	-2.53775900
N	7.58317400	-7.28043700	1.65295800	N	9.20672700	-5.76215200	1.46050400

Structure S76. The coordinates of both the optimized structures of B2N5L$_3$ at S$_0$ and S$_1$ in toluene.

S_0-optimized structure				S_1-optimized structure			
H	-1.42957900	10.66491100	0.37592500	H	-1.32283000	10.98346200	-0.44011200
C	-4.31877400	7.05307400	-1.03183200	C	2.19046900	7.89861600	0.79061000
C	-4.79919900	8.12510900	-0.26720700	C	2.46332400	9.11266400	0.11605100
C	-3.99898300	9.26232700	-0.09642700	C	1.45534400	10.09657200	0.02709100
C	-2.74795300	9.30342800	-0.68193200	C	0.21873600	9.83792000	0.56376500
C	-2.25625700	8.21302100	-1.41579000	C	-0.06338500	8.60398100	1.19231400
C	-3.05118900	7.08516700	-1.59114000	C	0.94546700	7.64088400	1.31389100
H	-4.94949300	6.18448900	-1.17700800	H	2.98149200	7.16934500	0.90349100
H	-4.36534000	10.09456600	0.49390900	H	1.65458900	11.02760400	-0.48847300
H	-2.69914800	6.23789500	-2.16976500	H	0.76185800	6.70424200	1.82732400
C	-1.72223300	10.40562200	-0.64746100	C	-1.00902200	10.70671300	0.57157500
H	-2.10111600	11.32613100	-1.10538700	H	-0.84100000	11.64186800	1.11603700
C	-0.57625300	9.81989900	-1.42976300	C	-2.02624800	9.83328900	1.25347300
C	0.65877400	10.37214500	-1.71149400	C	-3.34694500	10.09061600	1.51957900
C	1.58766800	9.62742100	-2.44838300	C	-4.13274100	9.08356900	2.12098900
C	1.25976800	8.33620900	-2.87711300	C	-3.54590000	7.83886000	2.45080300
C	0.02278800	7.78076500	-2.58686800	C	-2.21571100	7.59676200	2.20295500
C	-0.90474500	8.52631200	-1.86694200	C	-1.43935600	8.59252700	1.59656100
H	0.92083500	11.36750800	-1.36965600	H	-3.80199300	11.03341600	1.24199900
H	1.98877500	7.77247100	-3.44741100	H	-4.15498000	7.08498000	2.93237300
H	-0.21196500	6.77941800	-2.93144200	H	-1.77954900	6.64574500	2.48516200
H	-10.41606400	5.29513200	1.79848100	H	8.69417900	7.86576800	-1.65636300
C	-8.61398300	1.01998500	3.16958000	C	8.23137400	3.18177700	-2.79808700
C	-9.95526800	1.15266600	2.79548700	C	9.47397000	3.72152700	-2.43150300
C	-10.39333500	2.32751600	2.17348400	C	9.53658800	5.00799600	-1.87745600
C	-9.48434900	3.34239800	1.93792400	C	8.36681900	5.72188800	-1.70442700
C	-8.13791000	3.20449500	2.31367100	C	7.12733700	5.18005500	-2.08377100
C	-7.70221800	2.03643100	2.92933400	C	7.06238400	3.90128500	-2.62915200
H	-8.29349600	0.10335500	3.65093100	H	8.19883800	2.18691500	-3.22516200
H	-11.43230600	2.42027500	1.87581900	H	10.49426800	5.41739400	-1.57630800
H	-6.66588300	1.91370500	3.22488100	H	6.11416200	3.46695600	-2.92608000
C	-9.69465000	4.67088300	1.25934200	C	8.16755100	7.08528800	-1.09623800
H	-10.08357500	4.55438300	0.24191400	H	8.54064400	7.13358500	-0.06764800
C	-8.31520300	5.27610300	1.27424300	C	6.67450300	7.26556500	-1.16609400
C	-7.88624200	6.47743200	0.74250000	C	5.90149500	8.31780200	-0.72128300
C	-6.54056500	6.84498100	0.87557800	C	4.51264200	8.26416700	-0.90278100
C	-5.65696600	5.99300500	1.55291600	C	3.93047800	7.16641300	-1.55186400
C	-6.08417200	4.77554700	2.05756900	C	4.70655100	6.10058800	-1.97201400
C	-7.41821000	4.40878400	1.91570100	C	6.08368600	6.14160400	-1.77082300
H	-8.57391800	7.13272600	0.22008300	H	6.34931000	9.17297000	-0.22858600
H	-4.62259500	6.29184600	1.67127300	H	2.86189300	7.15825200	-1.72851300
H	-5.37794300	4.12816400	2.56622100	H	4.23882300	5.25705200	-2.46683700
H	-3.00169500	-10.34891500	-3.40637200	H	5.74265900	-9.57341300	3.01365200
C	-4.97441900	-6.72168800	-0.91070500	C	6.65363200	-5.31362400	0.93746900
C	-5.76022800	-7.70704900	-1.52417900	C	7.67893300	-6.11341400	1.45732000
C	-5.14849400	-8.88341000	-1.98563900	C	7.40930100	-7.45013500	1.78764200
C	-3.77878200	-9.02357000	-1.87332600	C	6.12503700	-7.93759500	1.64016200
C	-2.99608700	-8.02341000	-1.27510800	C	5.09722300	-7.12445700	1.13546300
C	-3.60373300	-6.87869300	-0.77689500	C	5.37184600	-5.81428000	0.76673900
H	-5.44528300	-5.81689100	-0.54821700	H	6.86408900	-4.28372800	0.67780500
H	-5.75203600	-9.66091000	-2.44022100	H	8.20481900	-8.07973800	2.17056400
H	-3.01920200	-6.09482800	-0.30718500	H	4.59333800	-5.17105500	0.37065500
C	-2.89615500	-10.15675600	-2.33323900	C	5.58100100	-9.30644600	1.96370500
H	-3.13986300	-11.09593600	-1.82362800	H	6.06347300	-10.08828500	1.36619200
C	-1.51306300	-9.66973800	-1.98046000	C	4.11518500	-9.17187900	1.63478600
C	-0.29004600	-10.26914900	-2.21342500	C	3.10124000	-10.10010000	1.77456400
C	0.88532500	-9.60306200	-1.83347500	C	1.78605200	-9.73977600	1.43915100
C	0.79263200	-8.36601000	-1.18162600	C	1.53862700	-8.45792200	0.92561900
C	-0.43759200	-7.78060900	-0.92672500	C	2.56319700	-7.53938300	0.76489200
C	-1.59684500	-8.42239700	-1.34139700	C	3.85618800	-7.88686300	1.13355000
H	-0.22679000	-11.23642900	-2.69918700	H	3.30743200	-11.09600000	2.14990700
H	1.69860500	-7.85361400	-0.88401300	H	0.52692600	-8.17958900	0.66031900
H	-0.47938200	-6.81774800	-0.42890400	H	2.33919200	-6.55289700	0.37315200
C	10.45808800	-5.94901700	3.66003100	C	-8.59460400	-8.22063600	-3.57138400
C	11.00182400	-7.24320000	3.61034100	C	-8.77584000	-9.61422900	-3.56995000
C	10.93662200	-5.08894200	4.66199500	C	-9.27251300	-7.50008100	-4.56929200
C	11.98733200	-7.65222200	4.49825200	C	-9.60269700	-10.24935900	-4.48726900
H	10.64955400	-7.93526300	2.85255000	H	-8.25843800	-10.21041500	-2.82469100
C	11.90297300	-5.49903800	5.57049000	C	-10.08690000	-8.12770100	-5.50332100
H	10.52894100	-4.08658900	4.73949700	H	-9.14279300	-6.42404000	-4.62105300
C	12.43613700	-6.78044500	5.48430300	C	-10.26173900	-9.50662800	-5.46116500
H	12.40105400	-8.65240400	4.42774400	H	-9.73011600	-11.32648100	-4.44718200
H	12.24369600	-4.82000200	6.34470400	H	-10.58637300	-7.54019400	-6.26710300
H	13.19818300	-7.10053600	6.18685700	H	-10.90232500	-9.99944600	-6.18511500
C	11.24497500	4.96037500	1.40732500	C	-12.48597300	1.87074100	-0.77457300
C	11.77355500	4.46967200	2.61299400	C	-12.86857400	1.36443300	-2.03025900
C	11.85816900	6.09674800	0.85481900	C	-13.42174100	2.69993300	-0.13116200
C	12.86460000	5.07055500	3.22660800	C	-14.10736700	1.64255200	-2.59337200
H	11.30489600	3.61703300	3.09192600	H	-12.16630900	0.75533100	-2.58902000
C	12.96840800	6.68335300	1.44440700	C	-14.66980100	2.96834900	-0.67617900
H	11.45435300	6.52706300	-0.05532100	H	-13.16709700	3.14717700	0.82382900
C	13.47295900	6.17149100	2.63517900	C	-15.02193400	2.43872800	-1.91317200
H	13.24178300	4.68029600	4.16571800	H	-14.35878700	1.23812300	-3.56878400
H	13.43235500	7.54988800	0.98584400	H	-15.36911400	3.60045500	-0.13790000
H	14.33122900	6.63762800	3.10723200	H	-15.99343700	2.65262800	-2.34611900
C	2.98147600	11.45842300	-3.28474000	C	-5.96303700	10.58416600	2.69529900
C	4.10267000	12.24401400	-2.99401600	C	-7.15769300	11.03060100	2.12666700
C	1.98730200	11.98804300	-4.11530800	C	-5.26830100	11.40257900	3.58945400
C	4.22397600	13.51843700	-3.52771400	C	-7.64459700	12.28820000	2.44755900
H	4.87664500	11.85167400	-2.34548300	H	-7.69203500	10.39380100	1.43222000
C	2.11246100	13.27012400	-4.62957500	C	-5.76130200	12.66141600	3.89619000
H	1.11869200	11.38828500	-4.35898400	H	-4.35727400	11.04126800	4.05172800
C	3.23085800	14.04512100	-4.34532000	C	-6.94929500	13.10931600	3.32862800
H	5.10147400	14.10928600	-3.28797100	H	-8.56969200	12.63070700	1.99880700

H	1.33013200	13.65936000	-5.27229300
H	3.32717500	15.04396600	-4.75486200
C	-6.89224600	9.20819700	0.37175000
C	-7.00964000	10.03659900	-0.74717900
C	-7.59388000	9.53484800	1.53502000
C	-7.80635400	11.17138800	-0.69626200
H	-6.47365500	9.78502200	-1.65482000
C	-8.40267800	10.66160800	1.57061100
H	-7.50079000	8.90061800	2.40879800
C	-8.51113200	11.48948100	0.45906000
H	-7.88636300	11.80342800	-1.57393300
H	-8.94029200	10.90067600	2.48168200
H	-9.13809800	12.37304800	0.49266100
C	2.45142500	-10.80044200	-3.31307000
C	1.78631000	-10.47633800	-4.50023700
C	3.46252500	-11.76700800	-3.35200600
C	2.11684300	-11.11777500	-5.68561000
H	1.01368400	-9.71746400	-4.49394400
C	3.79540300	-12.38948200	-4.54547900
H	3.98368900	-12.02768600	-2.43836500
C	3.12281200	-12.07582500	-5.72096800
H	1.58874500	-10.85014600	-6.59454200
H	4.58219800	-13.13606200	-4.55098100
H	3.37980200	-12.56986700	-6.65081200
C	-7.84248400	-7.90367300	-2.82674400
C	-7.22119000	-7.96196300	-4.07857500
C	-9.20366900	-8.21415400	-2.73711300
C	-7.94371300	-8.33839000	-5.20201300
H	-6.17267500	-7.70680600	-4.17091000
C	-9.91952300	-8.57393200	-3.86892100
H	-9.69505900	-8.17194000	-1.77219700
C	-9.29595100	-8.64524800	-5.10928200
H	-7.44239600	-8.37571400	-6.16320500
H	-10.97373000	-8.81113500	-3.77507200
H	-9.85618000	-8.93390300	-5.99109700
C	-12.05092000	0.26096300	3.71511800
C	-13.12904100	-0.60942400	3.51058700
C	-12.20523600	1.31543000	4.62354900
C	-14.31801700	-0.42930100	4.20117300
H	-13.03213500	-1.42490500	2.80419500
C	-13.40539400	1.49276500	5.29584100
H	-11.37911700	1.99202200	4.80468400
C	-14.47106000	0.62320900	5.09609700
H	-15.13831200	-1.11679800	4.02460100
H	-13.49954100	2.31648700	5.99540700
H	-15.40458700	0.76316300	5.62853600
H	-9.98484700	-2.96447600	-1.28019400
C	-7.99183400	-6.98028000	0.56066900
C	-7.79466000	-6.61685800	-0.77294200
C	-8.18561500	-5.35354000	-1.22334500
C	-8.74741000	-4.46528600	-0.32637600
C	-8.94741400	-4.82996200	1.01536200
C	-8.57233000	-6.09440400	1.45889700
H	-7.67674500	-7.96546400	0.88486600
H	-8.01623800	-5.08047400	-2.25942900
H	-8.71953000	-6.38969500	2.49207200
C	-9.17377300	-3.04006900	-0.54708000
H	-8.35290000	-2.42109000	-0.92488100
C	-9.61979900	-2.61306000	0.82403700
C	-10.07627800	-1.37858200	1.24347500
C	-10.44390300	-1.20440700	2.58064500
C	-10.37013000	-2.28174700	3.47105700
C	-9.90822400	-3.52031400	3.04942200
C	-9.51539300	-3.68478400	1.72433800
H	-10.14689900	-0.54093400	0.55823300
H	-10.66208400	-2.13106100	4.50395100
H	-9.83731600	-4.33839100	3.75811600
H	5.79788600	7.84075500	1.02028900
C	9.30646200	5.59818700	-1.42785300
C	9.01985200	5.24367900	-0.10069400
C	7.84142000	5.74222300	0.47632300
C	6.99491300	6.55743800	-0.25446300
C	7.30811200	6.91085500	-1.57561500
C	8.46923800	6.42636700	-2.16676900
H	10.20560100	5.21354500	-1.90009700
H	7.59155500	5.48908800	1.50326600
H	8.72298100	6.68248200	-3.19022800
C	5.68675700	7.18104700	0.15249200
H	4.94189100	6.42631700	0.42856500
C	5.28316600	7.94208400	-1.08132700
C	4.16522600	8.72642700	-1.29213200
C	4.00340300	9.36887600	-2.52405700
C	4.97141900	9.21202300	-3.52325800
C	6.09492500	8.42747700	-3.30806500
C	6.25198400	7.78368700	-2.08462800
H	3.41645000	8.85840900	-0.51874100
H	4.83038400	9.71139600	-4.47474800
H	6.83120100	8.31418700	-4.09653400
H	11.86982700	-0.87050800	0.93812100
C	7.93780800	-3.49302300	1.89974900
C	9.21260000	-4.00644100	2.21096400
C	10.30696100	-3.12198600	2.16455900
C	10.12156600	-1.79378300	1.82505100
C	8.83354900	-1.30995300	1.54357700
C	7.73380800	-2.16032100	1.57958300
H	7.08373800	-4.15994400	1.93359400
H	11.30204400	-3.49098700	2.39293700
H	-5.22199300	13.28860700	4.59664600
H	-7.33440600	14.09146000	3.57621300
C	4.18803700	10.65744500	-0.62503600
C	4.21654000	11.53688800	0.45694700
C	4.64858600	11.07088500	-1.87441900
C	4.69489500	12.82753900	0.28243200
H	3.87256400	11.20211300	1.42866000
C	5.13248600	12.36073800	-2.03666900
H	4.62040500	10.38167500	-2.71016000
C	5.15519500	13.24294200	-0.96199500
H	4.71979900	13.50659000	1.12695000
H	5.48580700	12.67974700	-3.01040200
H	5.53343100	14.25002000	-1.09306300
C	0.64378000	-11.53693800	2.68161800
C	1.20911600	-11.23906400	3.92633100
C	-0.04978700	-12.74281100	2.53124800
C	1.09656400	-12.13519800	4.97980500
H	1.73025600	-10.30040800	4.06918500
C	-0.16880500	-13.62399700	3.59524500
H	-0.49545200	-12.98229600	1.57305300
C	0.40774700	-13.33240700	4.82609000
H	1.54069500	-11.88246200	5.93671600
H	-0.71117500	-14.55264900	3.45428900
H	0.31818900	-14.02607600	5.65411300
C	9.71844200	-5.82525800	2.80679300
C	9.10924700	-6.13993100	4.02609100
C	11.11428300	-5.73822900	2.75619200
C	9.88174300	-6.37531500	5.15465700
H	8.02957100	-6.19612900	4.08909300
C	11.87501900	-5.96013700	3.89400900
H	11.59766700	-5.49876100	1.81629700
C	11.26748700	-6.28578300	5.10130400
H	9.38812000	-6.61733400	6.08976200
H	12.95545300	-5.88905400	3.83002200
H	11.86507800	-6.46717000	5.98714100
C	11.82584500	3.47592700	-3.10806400
C	13.05815300	2.92254400	-2.74331300
C	11.80934700	4.55077200	-4.00488800
C	14.23762200	3.43393100	-3.26425200
H	13.08726000	2.09152500	-2.04913000
C	12.99677100	5.06237700	-4.50606800
H	10.86294700	4.97664100	-4.31530400
C	14.21981700	4.50954500	-4.14391400
H	15.18129600	2.98909800	-2.96786300
H	12.96062400	5.89381400	-5.20170000
H	15.14434800	4.90865200	-4.54434800
H	10.61105400	-0.45396800	1.52733300
C	9.62516200	-4.72291100	-0.54597400
C	9.36669500	-4.49650800	0.80820900
C	9.44746300	-3.20396500	1.33266500
C	9.76330700	-2.15611000	0.48919400
C	10.02614600	-2.38352600	-0.87135500
C	9.96087000	-3.67313900	-1.38982600
H	9.55273800	-5.73453700	-0.92816300
H	9.23205000	-3.03970000	2.38297900
H	10.15913000	-3.86447300	-2.43874800
C	9.83043100	-0.68496900	0.79368800
H	8.88948700	-0.30964300	1.21004600
C	10.13561400	-0.08414800	-0.55029500
C	10.26344600	1.24614700	-0.90040500
C	10.56085900	1.57505400	-2.22483600
C	10.75178700	0.56788600	-3.17487300
C	10.61315800	-0.76831300	-2.82512000
C	10.28709700	-1.09578900	-1.51208800
H	10.13298900	2.03831900	-0.17101900
H	10.98981000	0.84582100	-4.19522900
H	10.74078000	-1.53922100	-3.57706800
H	-7.72519600	5.85569400	-0.96908800
C	-10.69370200	3.26735200	1.82331500
C	-10.32398700	2.79832100	0.54784600
C	-9.26857300	3.45850400	-0.10279500
C	-8.63186500	4.53231200	0.49196500
C	-9.03682000	4.99153200	1.75531500
C	-10.07329000	4.35045900	2.42912900
H	-11.48482000	2.75631700	2.36281800
H	-8.95231500	3.11714600	-1.08421900
H	-10.38819600	4.68158200	3.41363000
C	-7.48606400	5.36168200	-0.02087700
H	-6.58997000	4.75758500	-0.20111300
C	-7.27802800	6.35704500	1.08620200
C	-6.36727000	7.38759400	1.16985000
C	-6.38911300	8.22075300	2.29613200
C	-7.32213700	8.01202600	3.31805800
C	-8.22792700	6.96774200	3.23552600
C	-8.20973600	6.13291600	2.11835100
H	-5.65683500	7.57829000	0.37303600
H	-7.31844800	8.66716500	4.18126100
H	-8.93870700	6.80619100	4.03780700
H	-11.48222300	-3.87247800	-0.92647900
C	-6.91192300	-5.33353400	-1.38849700
C	-7.94470500	-6.12619600	-1.95936800
C	-9.23376100	-5.52135500	-2.02487700
C	-9.44656600	-4.24186800	-1.57721800
C	-8.38478700	-3.47079700	-1.03808600
C	-7.10540400	-4.04441000	-0.94260600
H	-5.91345100	-5.75398000	-1.33382900
H	-10.06347100	-6.09397800	-2.42849900

H	6.73603800	-1.79154600	1.36699000	H	-6.27392200	-3.47420500	-0.53998400
C	11.12554400	-0.67609100	1.71878200	C	-10.71382300	-3.42163200	-1.56559500
H	11.68277900	-0.53340900	2.65105600	H	-11.15585500	-3.33206200	-2.56399700
C	10.26494200	0.51392200	1.38432300	C	-10.25111800	-2.08955800	-1.02573400
C	10.61560400	1.84061700	1.21513100	C	-10.95289900	-0.92590500	-0.84885700
C	9.63643700	2.80610700	0.90847700	C	-10.32524500	0.25524900	-0.34345100
C	8.30787200	2.36593200	0.74850600	C	-8.95486500	0.11589800	0.02641900
C	7.93749500	1.04530300	0.94089700	C	-8.23597100	-1.04326300	-0.14952100
C	8.92283000	0.11876200	1.26758600	C	-8.87164600	-2.17001400	-0.70269900
H	11.65396900	2.14137700	1.30779900	H	-12.01086400	-0.89914800	-1.08888200
H	7.54728800	3.08843400	0.47493700	H	-8.44958400	0.97454600	0.45600500
H	6.90150900	0.74267800	0.83265300	H	-7.18819500	-1.08549100	0.13212200
H	5.91136100	-6.54103900	-1.43350300	H	-3.87892400	-8.08297800	1.38920000
C	3.25235900	-10.25331500	0.07813200	C	-0.37740700	-10.73612400	-0.57976200
C	3.22610900	-9.79735700	-1.24215100	C	-0.43406200	-10.46408900	0.78790600
C	4.21443200	-8.92637700	-1.70724800	C	-1.60128000	-9.94741500	1.35635300
C	5.19446600	-8.49613800	-0.83239500	C	-2.68391700	-9.67924900	0.54014800
C	5.21421800	-8.94542800	0.49809600	C	-2.62262700	-9.93714900	-0.84026200
C	4.24587300	-9.83573700	0.95221400	C	-1.46870300	-10.47855100	-1.39903500
H	2.47260400	-10.92809200	0.41193700	H	0.54087700	-11.13572600	-0.99499200
H	4.17468800	-8.57148100	-2.73148500	H	-1.62705500	-9.73201700	2.41940900
H	4.25036500	-10.18985100	1.97723500	H	-1.40855900	-10.68394300	-2.46229700
C	6.29482300	-7.49622900	-1.05960100	C	-4.00496800	-9.04966200	0.88982200
H	7.01989700	-7.85249500	-1.80054800	H	-4.58494200	-9.67958600	1.57446300
C	6.91377100	-7.37091000	0.30610300	C	-4.67576200	-8.91843900	-0.45180200
C	7.89535100	-6.50552500	0.74887500	C	-5.87731000	-8.31531800	-0.77654400
C	8.33708300	-6.55079500	2.08367400	C	-6.32992400	-8.28399000	-2.10817100
C	7.75971500	-7.50930400	2.93642100	C	-5.52983900	-8.91880100	-3.07794800
C	6.73384000	-8.34648600	2.51868300	C	-4.30535800	-9.49671800	-2.77339700
C	6.30097100	-8.26565800	1.19918200	C	-3.86918900	-9.48375300	-1.45164900
H	8.32519500	-5.77718300	0.06781100	H	-6.47831300	-7.84932100	-0.00125500
H	8.09967000	-7.56841500	3.96509600	H	-5.87069800	-8.92699400	-4.10840600
H	6.27555000	-9.04097800	3.21481200	H	-3.69813200	-9.93928800	-3.55672900
N	2.85812700	10.16470600	-2.75370500	N	-5.47279800	9.29528300	2.38382800
N	2.14929500	-10.16910900	-2.09252000	N	0.72591400	-10.64911700	1.59371600
N	-7.14720200	-7.51650600	-1.66499100	N	8.96976300	-5.57624200	1.64173400
N	-10.84775400	0.07768900	3.02382100	N	10.63301000	2.94404800	-2.59702000
N	-6.07562900	8.05741800	0.32805900	N	3.70603800	9.33172400	-0.45171800
B	9.98462200	4.31897400	0.73603100	B	-11.05369400	1.58576800	-0.17746700
B	9.35816100	-5.50425400	2.64109700	B	-7.64658700	-7.52304000	-2.52414300

Structure S77. The coordinates of both the optimized structures of B1N6L$_3$ at S$_0$ and S$_1$ in toluene.

S_0-optimized structure				S_1-optimized structure			
H	1.94034100	10.57069200	-0.44517000	H	-1.11510400	10.82061600	0.52806800
C	4.67696000	6.85449800	0.99666000	C	-3.97002800	7.07170600	-0.55801600
C	5.20318200	7.90155500	0.22770900	C	-4.47241900	8.21917700	0.09330900
C	4.45010800	9.06891700	0.04631000	C	-3.67757100	9.38048600	0.15130400
C	3.19916700	9.16378900	0.62581500	C	-2.42097300	9.36445900	-0.40921500
C	2.66093500	8.09836300	1.36373300	C	-1.90901800	8.20217400	-1.02030700
C	3.40922300	6.94070500	1.54970100	C	-2.70257000	7.05516500	-1.09827700
H	5.27180900	5.96234900	1.15006600	H	-4.59660600	6.19304500	-0.63618500
H	4.85252500	9.88208000	-0.54715700	H	-4.05194700	10.26634800	0.64939200
H	3.02095400	6.11198200	2.13197500	H	-2.34186200	6.15992000	-1.59202000
C	2.21878100	10.30618800	0.58085300	C	-1.39764100	10.46671700	-0.46903500
H	2.63295300	11.21349700	1.03454200	H	-1.77022000	11.33844600	-1.01738500
C	1.04727100	9.77189100	1.36232600	C	-0.24303400	9.80834200	-1.17532300
C	-0.16483800	10.37577500	1.63730800	C	0.98210700	10.33096100	-1.51040500
C	-1.12541800	9.67415400	2.37625300	C	1.93034100	9.49819200	-2.13834100
C	-0.85200200	8.37237800	2.81161600	C	1.61288400	8.15122000	-2.41188900
C	0.36187000	7.76515000	2.52775700	C	0.37521800	7.63868500	-2.09100200
C	1.32147800	8.46840200	1.80749400	C	-0.56696300	8.46510300	-1.47128500
H	-0.38456100	11.37921800	1.28934000	H	1.23926600	11.35729600	-1.27663200
H	-1.60483400	7.84168400	3.38262500	H	2.34620600	7.53078200	-2.91134000
H	0.55414600	6.75672800	2.87793600	H	0.13954100	6.60870400	-2.33237500
H	10.70325200	4.81362500	-1.78212800	H	-10.31826100	5.65284300	1.74857900
C	8.71827500	0.61153500	-3.12649800	C	-8.84322400	1.21948800	3.01790000
C	10.06138000	0.68630500	-2.74327900	C	-10.15002500	1.44464200	2.56632300
C	10.54843100	1.84594800	-2.12915000	C	-10.48370700	2.67305300	1.98207500
C	9.68522400	2.90366600	-1.91028900	C	-9.50804900	3.64431300	1.85810600
C	8.33671900	2.82371900	-2.29534700	C	-8.20015300	3.41407900	2.31529800
C	7.85235300	1.67096900	-2.90316400	C	-7.86754500	2.19456300	2.89555800
H	8.35941100	-0.29426800	-3.60118100	H	-8.60325000	0.26344400	3.46783700
H	11.58836700	1.89364300	-1.82428200	H	-11.49287900	2.83808000	1.62085100
H	6.81364800	1.59297700	-3.20550200	H	-6.86198300	2.00055600	3.25245100
C	9.95067900	4.22777500	-1.24246200	C	-9.59984100	5.00954700	1.22829600
H	10.32704900	4.10363000	-0.22122700	H	-9.92654300	4.95856900	0.18413900
C	8.60006100	4.89452300	-1.27291300	C	-8.19352500	5.53188300	1.35862500
C	8.22147200	6.11812400	-0.75372900	C	-7.65952900	6.72235900	0.90601100
C	6.89427600	6.54360700	-0.89895700	C	-6.30649500	6.99451700	1.14322600
C	5.97844000	5.72682700	-1.57683100	C	-5.52241400	6.07762500	1.85492700
C	6.35430300	4.48760900	-2.06900200	C	-6.05368900	4.87183000	2.28163700
C	7.66953400	4.06289500	-1.91382200	C	-7.39176500	4.58990700	2.02415500
H	8.93384000	6.74630200	-0.23109700	H	-8.26620600	7.43847400	0.36338600
H	4.95925600	6.07061700	-1.70447400	H	-4.48676500	6.31672000	2.06475800
H	5.62350100	3.86822500	-2.57780400	H	-5.42773500	4.16849200	2.81939600
H	2.46531700	-10.43884000	3.38787400	H	-3.20150800	-10.17484500	-3.42371700
C	4.66294100	-6.81857800	1.07199700	C	-5.20220700	-6.41928200	-1.14552200
C	5.38774700	-7.87890700	1.63244500	C	-5.98279300	-7.41398200	-1.74878500
C	4.70718500	-9.03625900	2.04150000	C	-5.37807600	-8.61666300	-2.14563700
C	3.33139000	-9.08882900	1.92624300	C	-4.01623200	-8.77702800	-1.97750000
C	2.61048200	-8.01490100	1.38031800	C	-3.23701400	-7.76761800	-1.38949700
C	3.28500200	-6.88475300	0.93774000	C	-3.83988900	-6.59387500	-0.95720600
H	5.18884000	-5.92857000	0.75046500	H	-5.67041500	-5.49434900	-0.83338400
H	5.26439700	-9.86940600	2.45503300	H	-5.98108200	-9.39926300	-2.59244600
H	2.74805900	-6.04551600	0.50872400	H	-3.25723300	-5.80374500	-0.49580100
C	2.38271400	-10.19169800	2.32393100	C	-3.13947800	-9.94476700	-2.35458600
H	2.58040500	-11.11829100	1.77321100	H	-3.42684500	-10.85874400	-1.82254300
C	1.03242300	-9.61244000	1.98405000	C	-1.76271100	-9.47314100	-1.95863000
C	-0.22349800	-10.16086000	2.16231700	C	-0.54602700	-10.11258500	-2.10155400
C	-1.35584000	-9.41518500	1.80118500	C	0.62626700	-9.45977500	-1.68962200
C	-1.18958300	-8.14468400	1.23353700	C	0.53421400	-8.18781000	-1.10677500
C	0.07267700	-7.60956300	1.03203000	C	-0.69054600	-7.56140400	-0.94275700
C	1.19056200	-8.33700300	1.41912200	C	-1.84533100	-8.19665400	-1.38055100
H	-0.34468200	-11.15125200	2.58670400	H	-0.48545700	-11.10407600	-2.53600900
H	-2.06426700	-7.56996800	0.95647400	H	1.43827500	-7.68450400	-0.78861100
H	0.17261500	-6.62141100	0.59584200	H	-0.73101900	-6.57428400	-0.49480000
C	-10.56020100	-5.38242600	-3.47622500	C	9.82114600	-5.96639500	3.98440900
C	-11.21773500	-6.59537400	-3.25122400	C	10.35188900	-7.26202300	3.99042200
C	-10.93625600	-4.60265700	-4.57399700	C	10.21554700	-5.07373800	4.98733200
C	-12.22536700	-7.01560900	-4.10680300	C	11.24271900	-7.65158100	4.97872600
H	-10.93390000	-7.20480300	-2.40132900	H	10.06418700	-7.95857900	3.21184300
C	-11.95727500	-5.02408400	-5.41361300	C	11.11980400	-5.47018100	5.96169500
H	-10.42303700	-3.66869900	-4.76903300	H	9.80830000	-4.07021900	4.99950500
C	-12.60710900	-6.23231300	-5.18981700	C	11.63810200	-6.75999100	5.96989900
H	-12.72377000	-7.95960000	-3.91524800	H	11.64226900	-8.65990400	4.96276200
H	-12.23434100	-4.40620700	-6.26085200	H	11.41097100	-4.76225800	6.73021900
H	-13.39975300	-6.56020100	-5.85234800	H	12.34125800	-7.06553400	6.73608500
C	-11.07172300	5.34672400	-1.22400000	C	11.83456500	4.24153000	0.39730400
C	-11.68383600	4.88977900	-2.40318600	C	12.56773000	3.74250200	1.49812500
C	-11.63234700	6.47874100	-0.61017400	C	12.47640700	5.26935100	-0.32873500
C	-12.80357300	5.51746100	-2.93268500	C	13.83922300	4.19362400	1.81946000
H	-11.25920200	4.04124900	-2.92822100	H	12.10850100	2.99883100	2.13965700
C	-12.76941200	7.09256600	-1.11501400	C	13.75186400	5.71893000	-0.02244500
H	-11.16556700	6.88475100	0.28070300	H	11.95824800	5.73736800	-1.15756600
C	-13.35695000	6.61329600	-2.28102900	C	14.45282300	5.17976600	1.05246400
H	-13.24588700	5.15183600	-3.85314800	H	14.35284600	3.77876500	2.68159800
H	-13.19020000	7.95522100	-0.60978400	H	14.20225900	6.50286600	-0.62405700
H	-14.23708500	7.10053100	-2.68692500	H	15.44923100	5.53209100	1.29685600
C	-2.44939900	11.56636500	3.19494700	C	3.32779200	11.31385200	-2.96087500
C	-3.55427700	12.37891500	2.91536600	C	4.42377300	12.07972400	-2.55442300
C	-1.42726600	12.07808300	4.00277000	C	2.38843900	11.85582000	-3.84293600
C	-3.63199000	13.66183900	3.43659800	C	4.57187400	13.37417200	-3.02681700
H	-4.35005200	12.00061500	2.28516600	H	5.14399400	11.65978400	-1.86292300
C	-1.50915100	13.36860200	4.50448700	C	2.54399500	13.15410700	-4.30175300
H	-0.57095900	11.45810600	4.23907000	H	1.55387500	11.25057900	-4.17588100
C	-2.61090500	14.17068700	4.23067000	C	3.63431300	13.91806600	-3.89832800
H	-4.49769400	14.27320700	3.20554900	H	5.42117100	13.96437600	-2.70293900

H	-0.70556400	13.74324300	5.12943600
H	-2.67315400	15.17620200	4.63034500
C	7.34307000	8.89296900	-0.40616700
C	7.48676900	9.72360600	0.70808500
C	8.06703000	9.18157300	-1.56586800
C	8.33119100	10.82328600	0.65601600
H	6.93375500	9.50122900	1.61311800
C	8.92277300	10.27304700	-1.60254800
H	7.95397900	8.54580200	-2.43615500
C	9.05754300	11.10343900	-0.49575900
H	8.43112300	11.45743500	1.53011300
H	9.47679400	10.48295400	-2.51097100
H	9.72150700	11.95951300	-0.53030900
C	-3.01424500	-10.62992600	3.15887000
C	-2.38141600	-10.39603300	4.38424900
C	-4.04970700	-11.56958300	3.10564300
C	-2.76772500	-11.09823700	5.51708200
H	-1.58978000	-9.65964000	4.44827800
C	-4.43817500	-12.25342900	4.24768500
H	-4.54529100	-11.76108300	2.16116200
C	-3.79818400	-12.02899900	5.46121400
H	-2.26410600	-10.90047500	6.45724400
H	-5.24271000	-12.97794800	4.18234600
H	-4.09895400	-12.57081100	6.35043300
C	7.45743600	-8.22847900	2.92235600
C	6.82812800	-8.28862400	4.17035300
C	8.80268000	-8.60267100	2.83014300
C	7.52629300	-8.72728100	5.28647900
H	5.79288000	-7.98519200	4.26578800
C	9.49443100	-9.02497300	3.95527400
H	9.30080200	-8.56072800	1.86871900
C	8.86252800	-9.09699100	5.19137900
H	7.01879800	-8.76426400	6.24445600
H	10.53633100	-9.31072300	3.85905500
H	9.40392700	-9.43419400	6.06768600
C	12.11367100	-0.31450900	-3.64986600
C	13.14302200	-1.24274900	-3.44759900
C	12.32304300	0.72850200	-4.56065600
C	14.33788600	-1.12915800	-4.14210600
H	13.00372000	-2.05088600	-2.73985800
C	13.52894300	0.83838000	-5.23706700
H	11.53498400	1.44927200	-4.74076400
C	14.54594700	-0.08795000	-5.03920600
H	15.11964100	-1.86057400	-3.96711500
H	13.66585200	1.65456800	-5.93841700
H	15.48402800	-0.00036800	-5.57480400
H	9.92157700	-3.42596600	1.35999200
C	7.64278300	-7.29714300	-0.46105600
C	7.48549300	-6.92478500	0.87543500
C	7.96686400	-5.69228800	1.32406100
C	8.57614200	-4.84128800	0.42176400
C	8.73580900	-5.21533800	-0.92283200
C	8.27187700	-6.45059600	-1.36425700
H	7.25790500	-8.25782000	-0.78340600
H	7.82785300	-5.41106600	2.36249100
H	8.38671400	-6.75251000	-2.39961000
C	9.09672600	-3.44704700	0.63870000
H	8.32324400	-2.77730100	1.02962700
C	9.55026400	-3.04617100	-0.73786800
C	10.07521300	-1.84120900	-1.16331600
C	10.43482200	-1.68866200	-2.50514200
C	10.28581400	-2.75889200	-3.39449100
C	9.75398200	-3.96719500	-2.96733200
C	9.36786300	-4.10792300	-1.63753500
H	10.20503200	-1.00945400	-0.47954900
H	10.57385800	-2.62522000	-4.43085700
H	9.62409200	-4.77885600	-3.67502300
H	-5.49318900	8.07964600	-1.03617200
C	-8.98378200	5.89814100	1.49243100
C	-8.75185300	5.55666900	0.15079000
C	-7.57911700	6.03129700	-0.45693000
C	-6.68639900	6.81132000	0.25760100
C	-6.94669900	7.15308600	1.59305200
C	-8.10039400	6.69093800	2.21563800
H	-9.87694900	5.53028100	1.98880400
H	-7.37057900	5.78842700	-1.49544100
H	-8.31309200	6.93713700	3.25080200
C	-5.37411900	7.40370600	-0.18201000
H	-4.66062600	6.63226100	-0.49257900
C	-4.90987100	8.13441700	1.04898500
C	-3.76365700	8.88337700	1.23645700
C	-3.54782900	9.50423400	2.47088200
C	-4.48959800	9.36280900	3.49653700
C	-5.64046900	8.61211100	3.30610800
C	-5.85141700	7.98926600	2.07996000
H	-3.03386500	9.00482500	0.44336900
H	-4.30601100	9.84718400	4.44860300
H	-6.35617500	8.50919200	4.11464400
H	-11.76425100	-0.47010800	-0.81119800
C	-7.98168600	-3.15785400	-2.15052600
C	-9.29562900	-3.62838900	-2.30573100
C	-10.36850000	-2.73988000	-2.14179500
C	-10.10969000	-1.41513400	-1.84533500
C	-8.79406400	-0.95450500	-1.68027700
C	-7.72870600	-1.83557400	-1.82996500
H	-7.15528100	-3.84293700	-2.29132600
H	-11.38598400	-3.09747800	-2.25053600

H	1.81689800	13.56533000	-4.99246300
H	3.75421300	14.93091700	-4.26439000
C	-6.48713000	9.39608600	0.80132900
C	-6.65535000	10.24557300	-0.29298300
C	-7.07581900	9.71868000	2.02418500
C	-7.39427400	11.41201600	-0.15677900
H	-6.20962700	9.98412300	-1.24573700
C	-7.82397800	10.88054100	2.14735300
H	-6.94135000	9.05704000	2.87180800
C	-7.98305000	11.73360100	1.06104900
H	-7.52195000	12.06526700	-1.01252200
H	-8.27598200	11.12463200	3.10201100
H	-8.56455800	12.64258600	1.16211000
C	2.23042200	-10.80848400	-2.98620000
C	1.65886000	-10.54360700	-4.23535000
C	3.19076100	-11.82140500	-2.88804800
C	2.02968500	-11.28663200	-5.34702300
H	0.92847500	-9.74999400	-4.33451400
C	3.56468500	-12.54718000	-4.00888000
H	3.64034100	-12.03542300	-1.92560800
C	2.98430100	-12.29160400	-5.24593900
H	1.57516800	-11.06302600	-6.30624800
H	4.31072000	-13.32808200	-3.90844700
H	3.27381000	-12.86525300	-6.11879000
C	-8.01020100	-7.57501300	-3.13855300
C	-7.32713800	-7.66399200	-4.35613200
C	-9.38401500	-7.84039900	-3.11835900
C	-8.00273600	-8.02481300	-5.51334600
H	-6.26712700	-7.44546700	-4.39536700
C	-10.05150900	-8.18491700	-4.28387400
H	-9.92340600	-7.77647600	-2.18067000
C	-9.36742000	-8.28612300	-5.48982200
H	-7.45338100	-8.08660200	-6.44663900
H	-11.11632000	-8.38744400	-4.24340400
H	-9.89043800	-8.56331100	-6.39776700
C	-12.37661100	0.62624700	3.21317500
C	-13.44976700	-0.19634100	2.85015500
C	-12.60362200	1.65920900	4.13101700
C	-14.70835500	0.01121800	3.39470000
H	-13.29361600	-0.99675200	2.13722200
C	-13.87070700	1.86485700	4.65579500
H	-11.78177200	2.29460800	4.43777700
C	-14.93359100	1.04411200	4.29684000
H	-15.52409300	-0.63920400	3.09796600
H	-14.02164300	2.67009700	5.36678000
H	-15.92023100	1.20513100	4.71527400
H	-10.15737400	-2.61856200	-1.62665400
C	-8.27669100	-6.67262600	0.25205000
C	-8.03096800	-6.30417400	-1.07245300
C	-8.39316000	-5.03410700	-1.52816100
C	-8.97363800	-4.14447700	-0.64447500
C	-9.22392300	-4.51514800	0.68692200
C	-8.87828300	-5.78619300	1.13531500
H	-7.98311800	-7.66294000	0.58055800
H	-8.18582000	-4.75701900	-2.55621300
H	-9.06348300	-6.08579300	2.16108600
C	-9.37082600	-2.71152800	-0.86916900
H	-8.52897300	-2.10318100	-1.21682700
C	-9.85416000	-2.28663400	0.48974000
C	-10.29963100	-1.04693500	0.90611600
C	-10.70973300	-0.87978900	2.23104500
C	-10.69311700	-1.96509500	3.11285700
C	-10.23790600	-3.20833300	2.69635700
C	-9.79979100	-3.36677600	1.38465800
H	-10.33023700	-0.20058500	0.22846700
H	-11.02211300	-1.81685300	4.13502800
H	-10.20837300	-4.03482700	3.39802400
H	6.21605300	7.28396200	0.92102600
C	9.58600800	5.27131300	-1.89079000
C	9.36776400	4.77476800	-0.58122600
C	8.20794300	5.23247800	0.08212100
C	7.34157200	6.11762400	-0.52352500
C	7.60337100	6.60938800	-1.81875100
C	8.73996900	6.17383200	-2.50697900
H	10.44292500	4.90648900	-2.44719100
H	8.00923800	4.89311000	1.09420000
H	8.94873100	6.52155100	-3.51373000
C	6.06021400	6.70861600	0.00173600
H	5.31886100	5.93744500	0.23804400
C	5.61230200	7.58886900	-1.13268700
C	4.49774700	8.38595800	-1.22740700
C	4.31417500	9.16714500	-2.38358900
C	5.25904800	9.12774700	-3.42300200
C	6.36616800	8.30818800	-3.33404700
C	6.55363100	7.52684500	-2.18691000
H	3.77707000	8.44701000	-0.41949000
H	5.09434800	9.72921700	-4.30905200
H	7.07644500	8.27076400	-4.15187400
H	11.62060200	-1.57969600	0.65306500
C	7.60029700	-3.60891200	2.41232300
C	8.84543500	-4.23385700	2.54885100
C	10.01555500	-3.51676100	2.24994500
C	9.91807500	-2.20294900	1.84242200
C	8.66157700	-1.57847300	1.68835100
C	7.49993100	-2.29889500	1.97231000
H	6.70299300	-4.16519100	2.65741600
H	10.98115600	-4.00086900	2.35127900

H	-6.70418900	-1.49712900	-1.71896700
C	-11.07949800	-0.28020600	-1.64538900
H	-11.70461400	-0.11868900	-2.53040000
C	-10.17146900	0.89040300	-1.36923500
C	-10.48373600	2.21665400	-1.14364400
C	-9.46664000	3.16185900	-0.89575300
C	-8.13834800	2.69521200	-0.85636100
C	-7.80746700	1.37467900	-1.11292900
C	-8.83086600	0.46895100	-1.37747500
H	-11.52123800	2.53471000	-1.14177800
H	-7.34437400	3.39681100	-0.62625400
H	-6.77098600	1.05491900	-1.09669200
H	-6.39835500	-6.26274800	1.45429300
C	-3.62432200	-9.81279100	-0.23692200
C	-3.67896500	-9.48418300	1.12001500
C	-4.69940100	-8.66064400	1.60351200
C	-5.62608200	-8.14926500	0.71459600
C	-5.55848600	-8.46301100	-0.65256300
C	-4.56068200	-9.30826100	-1.12787200
H	-2.82423600	-10.45542900	-0.58576500
H	-4.72600800	-8.40489400	2.65728400
H	-4.49871900	-9.56271700	-2.18033900
C	-6.74867400	-7.18005500	0.96951000
H	-7.51397900	-7.60779700	1.62735500
C	-7.28316500	-6.92260100	-0.41310900
C	-8.26989300	-6.04324700	-0.81627400
C	-8.57971000	-5.94527600	-2.17594200
C	-7.92805100	-6.76383400	-3.10385500
C	-6.94277800	-7.65162300	-2.69729000
C	-6.60148500	-7.71549400	-1.35018400
H	-8.78931500	-5.41181000	-0.10372000
H	-8.18459300	-6.67716700	-4.15326400
H	-6.42929200	-8.26080300	-3.43316200
N	-2.37275700	10.26444100	2.67660100
N	-2.65195000	-9.93395700	1.99163600
N	6.78557900	-7.78118800	1.77000400
N	10.90522400	-0.43087500	-2.95414600
N	6.47835500	7.77819400	-0.36159600
B	-9.77755600	4.67068600	-0.65559800
N	-9.52161400	-4.98048100	-2.61304500
H	6.52276000	-1.83813500	1.87443100
C	11.01393700	-1.22696900	1.49518700
H	11.70437000	-1.07854900	2.33294700
C	10.25148500	0.03085600	1.15254100
C	10.72809100	1.27019600	0.79416100
C	9.84966700	2.35947800	0.51345900
C	8.46077600	2.03951500	0.54847500
C	7.96997600	0.80608700	0.92059100
C	8.86612400	-0.21718300	1.25172200
H	11.79862400	1.41922100	0.70265700
H	7.74564300	2.80638000	0.27494300
H	6.89791300	0.63403800	0.95025700
H	5.81306900	-6.61854800	-1.15588600
C	2.81510600	-10.04223200	0.41115300
C	2.91673200	-9.69413900	-0.93747800
C	3.99021100	-8.91906300	-1.38545700
C	4.92403900	-8.47359100	-0.46940400
C	4.81131600	-8.80710900	0.89114700
C	3.75911500	-9.60536300	1.32994600
H	1.97352800	-10.64546300	0.73197100
H	4.05255300	-8.64762400	-2.43390400
H	3.66134300	-9.87400100	2.37616900
C	6.10294000	-7.56281700	-0.68312500
H	6.85676900	-8.01916400	-1.33498200
C	6.62256300	-7.36041300	0.71460000
C	7.64683400	-6.54382700	1.15050700
C	7.94092900	-6.48504000	2.51845000
C	7.22160900	-7.28456400	3.41763200
C	6.19723800	-8.10742500	2.97641100
C	5.87864700	-8.13202000	1.62212200
H	8.21090900	-5.92898200	0.45815400
H	7.45589500	-7.22993200	4.47409900
H	5.63453100	-8.69589300	3.69322300
N	3.18184700	9.99076300	-2.49280000
N	1.88480300	-10.07110500	-1.83985700
N	-7.36235900	-7.20594600	-1.94513600
N	-11.10250600	0.40900500	2.67471300
N	-5.73751300	8.19958500	0.67037700
B	10.36534100	3.76059300	0.11052100
N	8.91588600	-5.57962900	2.98331300

Structure S78. The coordinates of both the optimized structures of N7L$_3$ at S$_0$ and S$_1$ in toluene.

S$_0$-optimized structure				S$_1$-optimized structure			
H	4.36070900	9.81000500	0.00801900	H	6.14993000	8.96816200	0.12861100
C	6.22641200	5.47169600	1.05404300	C	7.19799600	4.32369500	1.03910700
C	6.95855400	6.44052000	0.35317200	C	8.11537300	5.17993400	0.38901000
C	6.48053800	7.75656900	0.29462200	C	7.87819100	6.57743600	0.38280000
C	5.28814500	8.07109400	0.91947100	C	6.74650300	7.06461300	0.98571900
C	4.53733000	7.08698200	1.57977400	C	5.80226600	6.19767700	1.59122200
C	5.01577200	5.78329400	1.65038700	C	6.05590400	4.81645500	1.62703100
H	6.61222400	4.46160700	1.11593100	H	7.41033500	3.26220100	1.07367300
H	7.04364800	8.51068900	-0.24345400	H	8.57647100	7.23937700	-0.11649200
H	4.46146700	5.01197700	2.17418500	H	5.37098400	4.13858000	2.12411700
C	4.57860800	9.39802000	0.99942000	C	6.26902300	8.49002900	1.10701200
H	5.18199900	10.14851300	1.52229300	H	6.97597300	9.10818900	1.67160100
C	3.32059900	9.06022400	1.75723300	C	4.95028700	8.34292000	1.82335300
C	2.26138800	9.87804000	2.10497900	C	4.04794500	9.31267900	2.19449700
C	1.17136800	9.32715300	2.78972000	C	2.84495500	8.92717400	2.82011300
C	1.16290800	7.96434500	3.10374600	C	2.58619100	7.56550000	3.05004800
C	2.22384900	7.14395300	2.75200900	C	3.49698000	6.59236400	2.69147200
C	3.31077100	7.69381800	2.08243600	C	4.69863600	6.97317900	2.07667700
H	2.25601900	10.93292000	1.85210100	H	4.23630800	10.36302400	1.99970300
H	0.30941200	7.55352500	3.63028200	H	1.65495300	7.28716300	3.53001400
H	2.19880800	6.08962300	3.00522000	H	3.28053400	5.54866000	2.89103400
H	11.53351000	2.22601500	-1.89208000	H	11.79706600	0.27377700	-2.08905300
C	8.55002500	-1.29283100	-3.35453600	C	8.11382000	-2.66302600	-3.27036400
C	9.86104900	-1.57891600	-2.96048800	C	9.39292200	-3.17818200	-2.98644100
C	10.62742300	-0.59940700	-2.31862200	C	10.37813900	-2.33315800	-2.42511200
C	10.06606500	0.64111600	-2.07716800	C	10.05975000	-1.02569300	-2.15419900
C	8.74724800	0.92238600	-2.47019400	C	8.77316400	-0.50237800	-2.45178500
C	7.98713900	-0.04958400	-3.11103700	C	7.79907100	-1.34558200	-3.02022800
H	7.97347300	-2.06471200	-3.85099600	H	7.37347500	-3.32606800	-3.70352600
H	11.64001900	-0.83174600	-2.00657100	H	11.35683000	-2.73695600	-2.18894300
H	6.96690100	0.15190200	-3.41904200	H	6.81052100	-0.96960000	-3.25951500
C	10.65540600	1.83243600	-1.36764500	C	10.89879000	0.04599300	-1.50381800
H	10.98457300	1.58414800	-0.35287500	H	11.24404200	-0.25309200	-0.50826000
C	9.51784400	2.82017000	-1.37096600	C	9.95670000	1.22295500	-1.44739000
C	9.45086900	4.07428300	-0.79297500	C	10.14950500	2.46083200	-0.89507800
C	8.27116700	4.82003500	-0.91180700	C	9.09183900	3.40853500	-0.93783000
C	7.19017000	4.29408100	-1.63230700	C	7.88266600	3.07026100	-1.58882600
C	7.25004700	3.02537200	-2.18498300	C	7.68881800	1.82314400	-2.13265000
C	8.41465500	2.27760400	-2.04694000	C	8.71813900	0.86331000	-2.04711200
H	10.29019000	4.47957800	-0.23908500	H	11.07868200	2.71539100	-0.39789700
H	6.29014100	4.88741000	-1.73871600	H	7.10111000	3.81776900	-1.65543400
H	6.39378700	2.63238300	-2.72243300	H	6.75381900	1.59079500	-2.63010800
H	-0.11746800	-10.49850100	3.47952500	H	-1.99590000	-10.30564400	3.45552100
C	2.76855000	-7.55505200	0.96947800	C	1.30664400	-7.84315500	0.93889900
C	3.25249700	-8.72816600	1.56431600	C	1.60777200	-9.06762500	1.55086200
C	2.34067800	-9.68689900	2.03323100	C	0.56119300	-9.87735900	2.02022700
C	0.98556900	-9.43371800	1.94359000	C	-0.74076500	-9.42750300	1.91799500
C	0.50851900	-8.24759400	1.36353600	C	-1.03295900	-8.18844400	1.32542000
C	1.40740200	-7.31633100	0.86033400	C	-0.00314700	-7.40639000	0.81802400
H	3.47118900	-6.81795100	0.60196800	H	2.11368400	-7.22270000	0.57021100
H	2.70733100	-10.60714200	2.47392700	H	0.78562100	-10.83668900	2.47291100
H	1.06073100	-6.39518700	0.40421800	H	-0.20702800	-6.44853700	0.35144400
C	-0.17423200	-10.27842800	2.40800500	C	-2.01590300	-10.08525100	2.38278800
H	-0.20637700	-11.24533200	1.89318200	H	-2.19195500	-11.03903200	1.87250400
C	-1.36940900	-9.42261300	2.07117600	C	-3.06758000	-9.06089000	2.03755200
C	-2.71011200	-9.66470900	2.30282600	C	-4.42906900	-9.09433100	2.27143500
C	-3.65685900	-8.69716700	1.93331600	C	-5.21803400	-7.99514000	1.89802000
C	-3.22709400	-7.52251100	1.30124600	C	-4.61414800	-6.90389900	1.25783400
C	-1.88365500	-7.29551200	1.04744300	C	-3.25262400	-6.88671600	1.00084500
C	-0.94594300	-8.23972400	1.44454900	C	-2.46905100	-7.96058800	1.40299300
H	-3.03738100	-10.58376500	2.77606500	H	-4.89107500	-9.95023500	2.75054900
H	-3.95806100	-6.77671000	1.01568200	H	-5.22185300	-6.05600200	0.96870200
H	-1.57836800	-6.37456800	0.56229400	H	-2.81120200	-6.02578000	0.51030900
C	-11.69450800	-2.68157500	-3.31932900	C	-12.02954500	-0.61671000	-3.29508200
C	-12.65810700	-3.65775400	-3.04631300	C	-13.17105000	-1.38129200	-3.03441500
C	-11.86270300	-1.86298700	-4.44049000	C	-12.01497600	0.21164700	-4.42137500
C	-13.75844800	-3.80981600	-3.87654000	C	-14.26690600	-1.31817900	-3.88197400
H	-12.53770800	-4.29492500	-2.17808500	H	-13.19180900	-2.02399300	-2.16212000
C	-12.97623900	-2.01244400	-5.25486900	C	-13.12328900	0.28024300	-5.25340800
H	-11.11655500	-1.11313500	-4.67380100	H	-11.13133800	0.79714300	-4.64506400
C	-13.93019800	-2.98605900	-4.98312300	C	-14.25513000	-0.48347200	-4.99363700
H	-14.49394900	-4.57299300	-3.64661600	H	-15.14246800	-1.91902900	-3.66140000
H	-13.08780500	-1.36829500	-6.12037200	H	-13.09223900	0.92797500	-6.12286300
H	-14.79508900	-3.10223700	-5.62593900	H	-15.11604700	-0.43044800	-5.64998500
C	-8.53749400	7.54557900	-2.00904700	C	-6.95359000	8.79174300	-2.06184500
C	-8.95699600	7.16182500	-3.28833800	C	-7.41948600	8.45918500	-3.33953700
C	-8.77256900	8.86241200	-1.59487400	C	-6.92528900	10.14179900	-1.69076300
C	-9.60376400	8.06834200	-4.11576600	C	-7.85532200	9.45005700	-4.20720600
H	-8.76974000	6.15332100	-3.63558400	H	-7.43300700	7.42311500	-3.65419200
C	-9.40694200	9.76273500	-2.43739300	C	-7.35055000	11.12353500	-2.57284200
H	-8.45749900	9.17453200	-0.60639100	H	-6.57026800	10.41583100	-0.70455200
C	-9.83340100	9.37509700	-3.70224300	C	-7.82433000	10.78899900	-3.83603600
H	-9.91779700	7.74761400	-5.10336800	H	-8.20995300	9.16722600	-5.19264200
H	-9.57806400	10.77683100	-2.09245000	H	-7.31977300	12.16187500	-2.26052700
H	-10.33465500	10.07998000	-4.35532000	H	-8.16199200	11.55875000	-4.52021400
C	0.20372500	11.29625900	3.90206800	C	2.22019000	11.02711800	3.92504700
C	-0.80065100	12.27329800	3.89308500	C	1.43155000	12.18184500	3.84066000
C	1.34364800	11.51863700	4.68567600	C	3.34744500	11.04110900	4.75700700
C	-0.66402900	13.42954900	4.64630100	C	1.76264800	13.31115000	4.57346900
H	-1.68727100	12.12418500	3.28907600	H	0.56251800	12.19015800	3.19416800
C	1.47231400	12.68639600	5.42285200	C	3.67409200	12.18240900	5.47288800
H	2.12588200	10.77056400	4.72035600	H	3.95749500	10.15071900	4.84604900
C	0.47263200	13.65184800	5.41455300	C	2.88652500	13.32546300	5.39181400
H	-1.45646100	14.16999400	4.62051100	H	1.13772300	14.19398200	4.49255600

H	2.36464800	12.83314600	6.02211000
H	0.57685300	14.55993300	5.99679300
C	9.25323300	6.97284200	-0.30691600
C	9.59850000	7.69126200	0.84087000
C	10.01110800	7.14446300	-1.46832200
C	10.67307700	8.56865000	0.81956000
H	9.02084800	7.55669100	1.74779000
C	11.09483200	8.01063900	-1.47464000
H	9.74432000	6.59594300	-2.36405700
C	11.43075700	8.73174300	-0.33454500
H	10.92737400	9.11792900	1.71949400
H	11.67155300	8.13205900	-2.38504900
H	12.27374300	9.41302300	-0.34526600
C	-5.49805500	-9.44371500	3.38994900
C	-4.78531100	-9.30939500	4.58606800
C	-6.72268900	-10.12034400	3.40960600
C	-5.28236800	-9.85304700	5.76210100
H	-3.84420400	-8.77341500	4.59358200
C	-7.21693800	-10.64593300	4.59374900
H	-7.28224900	-10.23221300	2.48836600
C	-6.49993900	-10.52279300	5.77841200
H	-4.71415300	-9.73587800	6.67864000
H	-8.16784800	-11.16756800	4.58471700
H	-6.88533500	-10.94128600	6.70093000
C	5.21533700	-9.50985600	2.83062600
C	4.62591300	-9.38572800	4.09310900
C	6.42738700	-10.19989700	2.71997500
C	5.22915600	-9.95348100	5.20644000
H	3.69702200	-8.83941900	4.20136200
C	7.02914100	-10.74974600	3.84179600
H	6.89277300	-10.30265300	1.74675700
C	6.43370200	-10.63726700	5.09286600
H	4.75551300	-9.84388800	6.17612900
H	7.96826400	-11.28106800	3.73151400
H	6.90258000	-11.07444900	5.96681400
C	11.58050800	-3.07535100	-3.87691300
C	12.33222100	-4.24117100	-3.68188800
C	12.05703000	-2.11529100	-4.77853100
C	13.51710100	-4.43717700	-4.37522300
H	11.98568100	-4.99076200	-2.98094200
C	13.25146400	-2.31811400	-5.45367700
H	11.48462100	-1.21247700	-4.95278500
C	13.99150700	-3.47908600	-5.26347700
H	14.08077600	-5.34841700	-4.20588800
H	13.59836300	-1.55999200	-6.14772500
H	14.92125200	-3.63469900	-5.79817100
H	8.71475500	-5.47389600	1.17291600
C	5.54014800	-8.69159800	-0.57425700
C	5.49989200	-8.28040400	0.75969900
C	6.26997300	-7.19481300	1.18432400
C	7.05345300	-6.52604300	0.26332200
C	7.09756400	-6.94086500	-1.07799200
C	6.34001600	-8.03028900	-1.49690800
H	4.93112500	-9.53502500	-0.87871100
H	6.22083600	-6.87855200	2.22077800
H	6.36215400	-8.36176000	-2.52930100
C	7.90349900	-5.30063000	0.45684800
H	7.32207600	-4.45619100	0.84238700
C	8.42786200	-5.04068800	-0.92829200
C	9.23581000	-4.01183100	-1.37207700
C	9.60958300	-3.96841800	-2.71803400
C	9.18438400	-4.97407100	-3.59359900
C	8.36906000	-6.00474200	-3.14788200
C	7.97490200	-6.03155600	-1.81317400
H	9.57899100	-3.23385900	-0.69886900
H	9.48614800	-4.92765400	-4.63364800
H	8.03109700	-6.76406900	-3.84476500
H	-3.50132300	9.74164600	-0.80427800
C	-7.01857300	7.00154800	1.10111000
C	-6.87121500	7.14631500	-0.28024000
C	-5.72497700	7.74859700	-0.80605000
C	-4.73951400	8.18587800	0.05921000
C	-4.89623100	8.05580300	1.44882200
C	-6.03894400	7.45919700	1.97165600
H	-7.91590800	6.52797900	1.48241900
H	-5.61855100	7.84523600	-1.88128400
H	-6.17049100	7.34735600	3.04234500
C	-3.40194300	8.79761300	-0.25689200
H	-2.78894600	8.13823500	-0.88097000
C	-2.80213500	9.00487600	1.10689500
C	-1.56442700	9.51852900	1.44672200
C	-1.22694200	9.64524200	2.79596700
C	-2.14089400	9.27077500	3.78621900
C	-3.37781700	8.74187600	3.44569100
C	-3.70478000	8.59589700	2.10076800
H	-0.85117200	9.82578600	0.68959700
H	-1.86201800	9.38635800	4.82741500
H	-4.06875700	8.43713900	4.22417400
H	-11.29068200	2.52333300	-0.92711400
C	-8.54138700	-1.25788800	-2.16697400
C	-9.93917900	-1.31849500	-2.26102300
C	-10.68845500	-0.14039300	-2.12523900
C	-10.03227400	1.05861600	-1.91880900
C	-8.63422100	1.10815600	-1.80624000
C	-7.89039000	-0.05894300	-1.92678700
H	-7.96328200	-2.16537800	-2.28703700
H	-11.76997700	-0.17923500	-2.19015300

H	4.54927900	12.16981900	6.11359900
H	3.14417100	14.21287500	5.95811800
C	10.44390400	5.32447200	-0.30141100
C	10.91342800	5.95524700	0.85365200
C	11.20517800	5.36920400	-1.47218100
C	12.12601200	6.62657900	0.83006000
H	10.32836400	5.90435400	1.76399900
C	12.42011600	6.03677100	-1.48169100
H	10.83120700	4.88868500	-2.36820600
C	12.88583500	6.66980700	-0.33424100
H	12.48491600	7.10879400	1.73228900
H	13.00101300	6.07124800	-2.39641300
H	13.83493000	7.19304700	-0.34687200
C	-7.15504800	-8.46387000	3.34920000
C	-6.43370100	-8.46107600	4.54767400
C	-8.47145800	-8.93807400	3.35818000
C	-7.01401600	-8.93655000	5.71510700
H	-5.41979200	-8.08066400	4.56386500
C	-9.04628900	-9.39606300	4.53394800
H	-9.03889900	-8.94631200	2.43501300
C	-8.32230900	-9.40531000	5.72068800
H	-6.43710300	-8.92413500	6.63360900
H	-10.06792100	-9.75988300	4.51661500
H	-8.77173500	-9.77103300	6.63664600
C	3.42274700	-10.09246600	2.85939000
C	2.84332800	-9.85081300	4.10958200
C	4.52379000	-10.95257300	2.78387900
C	3.34644200	-10.46856500	5.24566600
H	2.00201000	-9.17352300	4.18998100
C	5.02730900	-11.55280700	3.92797000
H	4.97995500	-11.14694100	1.82033700
C	4.44081000	-11.32163000	5.16699900
H	2.88319300	-10.26561700	6.20529800
H	5.88080600	-12.21698500	3.84520800
H	4.83230300	-11.79773500	6.05852800
C	10.80591700	-4.96335600	-3.88673200
C	11.36102600	-6.22202200	-3.62275600
C	11.42903800	-4.13241100	-4.82680900
C	12.50313700	-6.63519500	-4.29065200
H	10.89709100	-6.86788600	-2.88736800
C	12.57851800	-4.55326600	-5.47673700
H	10.99903100	-3.16372000	-5.04988000
C	13.12416200	-5.80610100	-5.21854600
H	12.91825900	-7.61293000	-4.07164400
H	13.04371500	-3.89719100	-6.20439200
H	14.02039600	-6.13194400	-5.73352100
H	7.49224000	-6.66684200	1.16404400
C	3.89559500	-9.42191800	-0.56218900
C	3.90315700	-8.97522700	0.76189300
C	4.82400400	-8.00471100	1.17041100
C	5.70857400	-7.48657100	0.24608500
C	5.71079500	-7.94319600	-1.08517200
C	4.79845500	-8.91777900	-1.48755900
H	3.16954900	-10.17175800	-0.85503000
H	4.81144800	-7.65671100	2.19779600
H	4.78336200	-9.27909000	-2.51002400
C	6.73093700	-6.39758300	0.42318200
H	6.27650100	-5.46272500	0.76875900
C	7.31607700	-6.26676600	-0.95620900
C	8.28119500	-5.39367700	-1.40817600
C	8.70155600	-5.46902000	-2.74355100
C	8.14438800	-6.43181800	-3.59902300
C	7.16011700	-7.29524400	-3.14648800
C	6.72969800	-7.20952200	-1.82237200
H	8.72537200	-4.65272600	-0.75266500
H	8.47828200	-6.47591900	-4.62924700
H	6.72400700	-8.01751500	-3.82811800
H	-1.58562200	9.93269700	-0.84596500
C	-5.63012600	8.08919200	1.09581900
C	-5.42989200	8.12675700	-0.28617200
C	-4.17256800	8.44981200	-0.80587700
C	-3.13498800	8.72026600	0.06544900
C	-3.34257200	8.70630500	1.45585900
C	-4.59532500	8.38513500	1.97208100
H	-6.61342400	7.83179900	1.47255100
H	-4.02785700	8.46528400	-1.88093100
H	-4.76912200	8.36347300	3.04237300
C	-1.69393600	9.02577700	-0.24063400
H	-1.21664000	8.21620100	-0.80358600
C	-1.09323300	9.19370100	1.12840500
C	0.21249500	9.47293200	1.47770200
C	0.53769400	9.63069200	2.82944200
C	-0.45557000	9.50997000	3.80966300
C	-1.76214400	9.20655700	3.45889900
C	-2.08357600	9.03645200	2.11405800
H	0.98912200	9.58156500	0.72842600
H	-0.18357600	9.63930700	4.85096100
H	-2.51611000	9.09502200	4.23058500
H	-10.65798100	4.42927100	-0.92656400
C	-8.67823300	0.16974600	-2.08708200
C	-10.05941600	0.38043600	-2.20851000
C	-10.56771500	1.68305000	-2.09262400
C	-9.69489800	2.73374700	-1.88092500
C	-8.31612000	2.51205700	-1.74014900
C	-7.81147400	1.22150400	-1.83940700
H	-8.28424500	-0.83304700	-2.19259500
H	-11.63463800	1.85505000	-2.17919600

H	-6.80804500	-0.04162000	-1.85678100	H	-6.74787800	1.02861700	-1.74868800
C	-10.60599700	2.44594100	-1.77912800	C	-9.98969900	4.20802000	-1.76635600
H	-11.17831500	2.74049800	-2.66557700	H	-10.48135800	4.59565500	-2.66524500
C	-9.37778600	3.30009800	-1.59134800	C	-8.62158300	4.80971300	-1.56667500
C	-9.28026200	4.66915000	-1.43163800	C	-8.26046100	6.13620800	-1.42889500
C	-8.01430400	5.25347000	-1.28292300	C	-6.90693200	6.46503100	-1.26510400
C	-6.87613600	4.43885300	-1.25868500	C	-5.95138200	5.44407100	-1.19868900
C	-6.97786000	3.06399500	-1.40592300	C	-6.31783500	4.11296800	-1.32521700
C	-8.22994000	2.49108300	-1.59015600	C	-7.65415200	3.79227600	-1.52947000
H	-10.16395500	5.29787600	-1.42847900	H	-9.00334800	6.92589100	-1.45650400
H	-5.90225800	4.89644400	-1.13414100	H	-4.90924400	5.70483500	-1.05991100
H	-6.08077400	2.45430100	-1.39395100	H	-5.55788100	3.34012100	-1.28312100
H	-7.78503300	-4.45356800	1.57604400	H	-8.54885100	-3.12715900	1.60142800
C	-6.04662400	-8.64768300	-0.01748500	C	-7.58613900	-7.54679900	-0.03835400
C	-5.95791900	-8.25538300	1.32029700	C	-7.41994600	-7.18520300	1.30046700
C	-6.72468000	-7.18651800	1.79239500	C	-7.98633200	-6.00348000	1.78623100
C	-7.53677800	-6.50190700	0.90796100	C	-8.67130900	-5.18017200	0.91261800
C	-7.60865100	-6.88252300	-0.44199500	C	-8.81620100	-5.53130400	-0.43960400
C	-6.87124000	-7.96843800	-0.90313800	C	-8.28542100	-6.72718100	-0.91307500
H	-5.44304900	-9.48143100	-0.35723900	H	-7.13832300	-8.46972500	-0.38836900
H	-6.63973200	-6.88555300	2.83106000	H	-7.84160700	-5.72976600	2.82582500
H	-6.92003800	-8.27593000	-1.94202600	H	-8.39353900	-7.01277300	-1.95375900
C	-8.37103100	-5.27199200	1.14500900	C	-9.27169000	-3.82313900	1.16324400
H	-9.19413000	-5.46725300	1.84210100	H	-10.11676200	-3.87494000	1.85956600
C	-8.87543300	-4.94436100	-0.23415600	C	-9.70893400	-3.39679900	-0.21185100
C	-9.61010100	-3.85319400	-0.65728500	C	-10.22656700	-2.18396600	-0.62472900
C	-9.92818700	-3.73097100	-2.01308600	C	-10.51229500	-1.99159400	-1.97968500
C	-9.55208900	-4.73314600	-2.91342300	C	-10.33034500	-3.03906900	-2.88869300
C	-8.82128200	-5.83230800	-2.48654400	C	-9.81932700	-4.25942900	-2.47186500
C	-8.45761100	-5.92584400	-1.14711600	C	-9.47960600	-4.43076300	-1.13379100
H	-9.91545100	-3.07511000	0.03374100	H	-10.38067900	-1.36849500	0.07345700
H	-9.81174400	-4.62427600	-3.96001600	H	-10.56209900	-2.87443300	-3.93451400
H	-8.51091000	-6.58489500	-3.20321100	H	-9.65413700	-5.05010400	-3.19566400
N	0.06074000	10.12874800	3.14671300	N	1.88253800	9.88284100	3.19389800
N	-7.88097400	6.65152400	-1.14958800	N	-6.50487300	7.81301800	-1.16193900
N	-10.57337600	-2.55744100	-2.47735200	N	-10.91879900	-0.71232700	-2.43474600
N	-5.02910400	-8.90072700	2.18013600	N	-6.60430900	-7.98384700	2.14712500
N	4.63888600	-8.94028000	1.67951300	N	2.94709400	-9.47690800	1.68636800
N	10.38166200	-2.87687600	-3.18425400	N	9.65039800	-4.53913400	-3.21980600
N	8.15793000	6.08412700	-0.29577800	N	9.21048200	4.63751800	-0.28583600
